# Supplementary material for: Common allotypes of ER aminopeptidase 1 have substrate-dependent and highly variable enzymatic properties
Source: J Biol Chem. 2021 Feb 20;296:100443. doi: 10.1016/j.jbc.2021.100443 (PMC8024916; doi:10.1016/j.jbc.2021.100443)
Supplement: Supplemental Figures S1–S4 and Tables S1–S8 [file mmc1.pdf]

Supporting Information

Common allotypes of ER aminopeptidase 1 have substrate-dependent and highly variable enzymatic properties

Jonathan P. Hutchinson[1,#], Ioannis Temponeras[2,#], Jonas Kuiper[3], Adrian Cortes[4], Justyna Korczynska[1], Semra Kitchen[5] and Efstratios Stratikos[2,6,*]

Affiliations:
[1]Medicinal Science and Technology, GlaxoSmithKline, Stevenage, Hertfordshire SG1 2NY, U.K.
[2]National Centre for Scientific Research "Demokritos", Athens 15341, Greece.
[3]Department of Ophthalmology, University Medical Center Utrecht, Utrecht University, Utrecht, The Netherlands. Center for Translational Immunology, University Medical Center Utrecht, University of Utrecht, Utrecht, Netherlands.
[4] Human Genetics, GlaxoSmithKline, Stevenage, Hertfordshire SG1 2NY, U.K.
[5] Adaptive Immunity Research Unit, GlaxoSmithKline, Stevenage, Hertfordshire SG1 2NY, U.K.
[6] Laboratory of Biochemistry, Department of Chemistry, National and Kapodistrian University of Athens, Panepistimiopolis Zografou 157 84, Greece.

[#] These authors contributed equally to the manuscript
[*] E-mail: stratos@rrp.demokritos.gr or estratikos@chem.uoa.gr.

Supporting Table 1: Frequency of ERAP1 SNPs in different populations based on analysis of 2,504 Human Genomes from the 1000 Genomes Project.

SNP ID / Amino acid	Population Frequency of SNP %											
	ALL		AFR		AMR		EUR		EAS		SAS	
rs3734016	C	T	C	T	C	T	C	T	C	T	C	T
E56K	91.4	8.6	91.9	8.1	91.4	8.6	95.9	4.1	82.8	17.2	95.0	5.0
rs26653	G	C	G	C	G	C	G	C	G	C	G	C
P127R	56.3	43.8	48.2	51.8	58.8	41.2	71.8	28.2	52.1	47.9	53.7	46.3
rs26618	T	C	T	C	T	C	T	C	T	C	T	C
I276M	77.3	22.7	80.4	19.6	85.6	14.4	78.1	21.9	71.4	34.6	72.6	27.4
rs27895	C	T	C	T	C	T	C	T	C	T	C	T
G346D	89.6	10.4	76.2	23.8	95.2	4.8	93.6	6.4	99.9	0.1	89.1	10.9
rs2287987	T	C	T	C	T	C	T	C	T	C	T	C
M349V	89.5	10.5	93.4	6.6	87.3	12.7	77.5	22.5	94.2	5.8	93.1	6.9
rs30187	C	T	T	C	C	T	T	C	C	T	C	T
K528R	59.6	40.4	40.2	59.8	59.5	40.5	35.0	65.0	54.7	45.3	59.1	40.9
rs10050860	C	T	C	T	C	T	C	T	C	T	C	T
D575N	89.4	10.6	93.5	6.5	86.9	13.1	77.1	22.9	94.2	5.8	93.1	6.9
rs17482078	C	T	C	T	C	T	C	T	C	T	C	T
R725Q	89.9	10.1	94.6	5.4	87.6	12.4	77.6	22.4	94.2	5.8	93.5	6.5
rs27044	G	C	G	C	G	C	G	C	G	C	G	C
Q730E	32.1	67.9	28.9	71.1	34.9	65.1	28.5	71.5	42.9	57.1	26.9	73.1

Supporting Table 2: Correlation of coding SNPs in the 1000 Genomes Project data set suggesting linkage disequilibrium between particular SNPs. Number correspond to percentage of frequency of the two SNPs exist in an individual. Color coding indicates level of correlation (green=0, red=100).

	56E	56K	127R	127P	276I	276M	346G	346D	349M	349V	528K	528R	575D	575N	725R	725Q
**127R**	43.8	0.0														
**127P**	47.7	8.6														
**276I**	68.8	8.6	43.6	33.8												
**276M**	22.7	0.0	0.2	22.5												
**346G**	82.5	7.2	37.5	52.1	67.0	22.7										
**346D**	9.0	1.4	6.2	4.2	10.4	0.0										
**349M**	80.9	8.6	43.7	45.8	66.8	22.7	79.1	10.4								
**349V**	10.5	0.0	0.0	10.5	10.5	0.0	10.5	0.0								
**528K**	40.4	0.0	34.2	6.2	40.4	0.0	40.3	0.0	40.4	0.0						
**528R**	51.0	8.6	9.6	50.1	37.0	22.7	49.3	10.3	49.1	10.5						
**575D**	80.8	8.6	43.7	45.7	66.9	22.5	79.0	10.4	89.4	0.0	40.4	49.0				
**575N**	10.6	0.0	0.0	10.6	10.5	0.1	10.6	0.0	0.1	10.5	0.0	10.6				
**725R**	81.4	8.6	43.7	46.7	67.7	22.2	79.6	10.4	89.0	9.6	40.3	49.6	88.9	1.0		
**725Q**	10.1	0.0	0.1	10.0	9.6	0.4	10.1	0.0	0.5	0.9	0.0	10.0	0.5	9.6		
**730Q**	32.1	0.0	25.7	6.4	32.0	0.1	32.0	0.1	32.0	0.1	31.8	0.3	32.0	0.1	32.1	0.0
**730E**	59.4	8.6	18.1	49.9	45.4	22.5	57.7	10.3	57.6	10.4	8.6	59.3	57.4	10.5	57.9	10.1

Supporting Table 3: Frequency of ERAP1 allotypes in samples from the UK biobank stratified by self-reported ethnic background.

	Population allotype frequency %												
Allotype	**British**	**Irish**	**Any other white background**	**White and Black Caribbean**	**White and Black African**	**White and Asian**	**Any other mixed background**	**Indian**	**Bangladeshi**	**Any other Asian background**	**Caribbean**	**African**	**Any other Black background**
1	13.0	13.0	13.9	8.4	9.0	10.2	9.4	8.2	8.6	10.9	6.7	2.5	0.7
2	13.6	12.6	14.0	18.8	21.0	19.6	20.2	20.6	19.0	23.3	25.0	26.6	28.7
3	7.0	6.8	8.4	10.0	8.8	9.9	9.6	13.6	13.3	13.1	12.0	10.8	12.1
4	0.6	0.5	0.8	1.6	1.4	1.1	1.8	1.5	1.9	2.5	1.8	2.6	2.8
5	6.7	6.8	6.9	6.4	9.2	7.1	6.2	11.1	12.2	8.4	8.2	7.7	8.5
6	8.5	7.6	9.2	7.6	6.2	8.5	7.6	7.9	7.6	5.4	6.8	5.7	5.7
7	4.5	5.9	3.3	3.6	2.9	4.6	4.7	4.9	4.0	5.2	6.2	2.4	2.2
8	22.9	22.2	23.6	20.4	18.7	22.4	22.7	21.3	22.2	21.0	24.3	17.5	16.4
9	0.2	0.5	0.1	0.2	0.0	0.2	0.1	0.0	0.0	0.0	0.0	0.0	0.0
10	22.0	23.1	18.9	14.8	13.2	15.3	14.3	9.5	9.9	8.6	8.2	5.3	3.7

Supporting Table 4: Distribution of combinations of ERAP1 allotypes in the global population

	% in population (global)									
Allotype	***1***	***2***	***3***	***4***	***5***	***6***	***7***	***8***	***9***	***10***
1	0.9									
2	2.2	8.2								
3	1.1	3.5	1.1							
4	0.5	1.7	0.5	0.1						
5	0.6	2.4	1.4	0.4	0.6					
6	1.0	2.4	0.9	0.4	0.9	0.4				
7	0.7	4.1	1.0	0.6	0.6	0.6	1.1			
8	2.4	11.0	3.2	1.1	2.6	2.0	3.3	5.8		
9	0.0	0.1	0.1	0.0	0.0	0.0	0.0	0.0	0.0	
10	1.7	4.0	1.8	0.6	1.3	1.6	1.0	4.1	0.0	1.2

Supporting Table 5: Distribution of combinations of ERAP1 allotypes in the sub-populations

					% in population (EUR)					
Allotype	***1***	***2***	***3***	***4***	***5***	***6***	***7***	***8***	***9***	***10***
1	3.0									
2	3.6	2.4								
3	2.0	1.0	1.0							
4	0.4	0.2	0.0	0.0						
5	1.6	1.8	0.4	0.0	0.6					
6	2.0	4.0	1.2	0.0	1.4	0.8				
7	1.6	0.8	0.2	0.0	0.6	0.8	0.2			
8	6.2	5.6	1.8	1.0	2.4	2.6	2.0	6.2		
9	0.2	0.2	0.2	0.0	0.0	0.2	0.0	0.0	0.0	
10	4.6	6.8	4.4	0.4	3.2	4.8	1.8	9.2	0.0	4.8

					% in population (AMR)					
Allotype	***1***	***2***	***3***	***4***	***5***	***6***	***7***	***8***	***9***	***10***
1	1.4									
2	5.8	5.2								
3	1.4	2.3	1.4							
4	2.0	4.6	1.4	0.6						
5	0.3	1.4	0.6	0.3	0.6					
6	2.3	4.6	0.9	2.0	0.3	1.2				
7	1.2	3.5	0.3	2.3	0.9	1.4	2.3			
8	3.5	6.6	0.9	2.3	1.7	2.9	1.2	2.3		
9	0.0	0.3	0.3	0.0	0.0	0.0	0.0	0.3	0.0	
10	4.6	4.9	0.6	1.7	0.9	3.7	1.7	4.0	0.0	0.9

					% in population (AFR)					
Allotype	***1***	***2***	***3***	***4***	***5***	***6***	***7***	***8***	***9***	***10***
1	0.0									
2	0.2	8.6								
3	0.2	6.5	0.9							
4	0.0	2.0	0.8	0.0						
5	0.2	4.2	2.1	0.9	0.3					
6	0.2	1.7	0.8	0.3	1.1	0.2				
7	0.2	1.7	1.2	0.5	0.2	0.2	0.2			
8	0.2	8.9	3.3	0.5	3.5	1.4	0.9	3.6		
9	0.0	0.0	0.0	0.0	0.0	0.0	0.0	0.0	0.0	
10	0.0	2.3	1.2	0.0	0.9	0.2	0.2	1.1	0.0	0.0

% in population (EAS)										
Allotype	***1***	***2***	***3***	***4***	***5***	***6***	***7***	***8***	***9***	***10***
1	0									
2	0	19								
3	0	2.6	0.2							
4	0	2.2	0	0						
5	0	0	0	0	0					
6	0	0.4	0	0	0	0				
7	0	13.4	1.2	0.8	0	0.4	3.6			
8	0	23.4	1.2	1.2	0	0.6	10	8.2		
9	0	0	0	0	0	0	0	0	0	
10	0	5.4	0	0.6	0	0	1.2	4.4	0	0

% in population (SAS)										
Allotype	***1***	***2***	***3***	***4***	***5***	***6***	***7***	***8***	***9***	***10***
1	0.4									
2	3.5	4.9								
3	2.2	3.7	2.2							
4	0.8	0.4	0.6	0.0						
5	1.2	3.5	3.3	0.8	1.6					
6	1.0	2.5	1.6	0.2	1.6	0.4				
7	0.8	1.8	1.8	0.0	1.4	0.4	0.0			
8	3.5	9.8	8.4	1.0	5.1	3.1	2.7	8.4		
9	0.0	0.0	0.0	0.0	0.0	0.0	0.0	0.0	0.0	
10	0.8	1.6	2.5	0.8	1.6	0.6	0.6	2.9	0.0	0.8

Supporting Table 6: Frequency of ERAP1 1-10 allotype combinations in genetic samples that carry the [A,A],[A,B] and [B,B] ERAP1 allotypes. Numbers indicate % values. Cells are color coded (red=high, yellow=medium, green=low).

[A,A] ERAP2 (495 samples)

ERAP1 allotype	1	2	3	4	5	6	7	8	9	10
10	1.6	2.0	1.8	0.0	0.2	1.8	1.4	8.7	0.0	2.2
9	0.0	0.0	0.0	0.0	0.0	0.0	0.0	0.0	0.0	0.0
8	3.2	8.5	9.1	1.6	2.4	3.0	9.1	19.2	0.0	8.7
7	1.0	1.8	2.2	0.6	0.6	0.4	1.8	9.1	0.0	1.4
6	0.4	0.8	0.4	0.6	0.2	0.2	0.4	3.0	0.0	1.8
5	0.4	0.8	0.4	0.4	0.4	0.2	0.6	2.4	0.0	0.2
4	0.0	1.4	0.6	0.0	0.4	0.6	0.6	1.6	0.0	0.0
3	1.2	1.6	2.2	0.6	0.4	0.4	2.2	9.1	0.0	1.8
2	1.0	1.6	1.6	1.4	0.8	0.8	1.8	8.5	0.0	2.0
1	0.6	1.0	1.2	0.0	0.4	0.4	1.0	3.2	0.0	1.6

[A,B] ERAP2 (1086 samples)

ERAP1 allotype	1	2	3	4	5	6	7	8	9	10
10	2.0	5.2	2.8	0.6	1.7	2.1	1.6	4.9	0.0	1.4
9	0.1	0.2	0.1	0.0	0.0	0.1	0.0	0.1	0.0	0.0
8	3.1	17.2	3.1	1.6	4.1	2.8	3.4	3.8	0.1	4.9
7	1.1	5.4	0.9	0.6	0.7	1.0	1.6	3.4	0.0	1.6
6	0.4	1.9	1.7	0.2	1.0	0.3	1.0	2.8	0.1	2.1
5	0.6	2.9	1.7	0.5	0.4	1.0	0.7	4.1	0.0	1.7
4	0.3	1.1	0.9	0.0	0.5	0.2	0.6	1.6	0.0	0.6
3	1.4	3.7	0.7	0.9	1.7	1.7	0.9	3.1	0.1	2.8
2	1.4	4.9	3.7	1.1	2.9	1.9	5.4	17.2	0.2	5.2
1	0.9	1.4	1.4	0.3	0.6	0.4	1.1	3.1	0.1	2.0

[B,B] ERAP2 (650 samples)

ERAP1 allotype	1	2	3	4	5	6	7	8	9	10
10	2.0	5.4	0.8	1.4	2.0	1.4	0.2	1.1	0.0	0.8
9	0.0	0.0	0.2	0.0	0.0	0.0	0.0	0.0	0.0	0.0
8	1.7	7.1	0.3	0.3	1.4	0.8	0.2	1.4	0.0	1.1
7	0.0	5.4	0.6	0.8	0.5	0.2	0.3	0.2	0.0	0.2
6	2.8	5.5	0.3	0.8	1.7	1.1	0.2	0.8	0.0	1.4
5	1.2	3.7	2.0	0.6	1.4	1.7	0.5	1.4	0.0	2.0
4	1.5	3.7	0.0	0.3	0.6	0.8	0.8	0.3	0.0	1.4
3	0.9	6.0	1.4	0.0	2.0	0.3	0.6	0.3	0.2	0.8
2	5.5	22.3	6.0	3.7	3.7	5.5	5.4	7.1	0.0	5.4
1	1.4	5.5	0.9	1.5	1.2	2.8	0.0	1.7	0.0	2.0

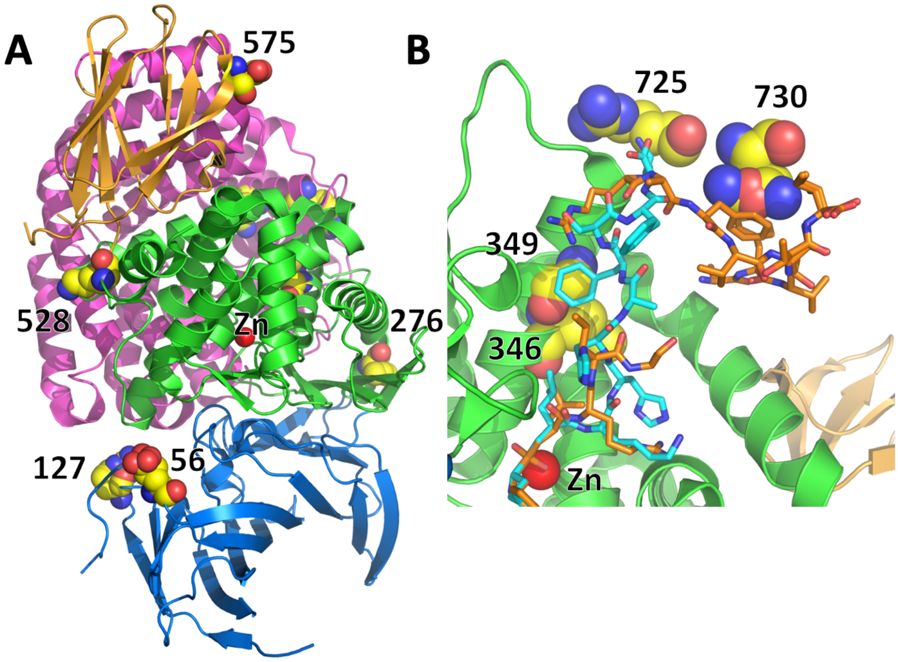

Supporting Figure 1: Schematic representations of ERAP1 crystal structure (PDB codes 6RYF and 6RQX) indicating the positions of 9 SNPs. Structure is colored by domain (domain I in blue, domain II in green, domain III in orange and domain IV in magenta). Active site Zn(II) atom is shown as a red sphere). Panel A, polymorphic residues that lie on the outside of the protein are indicated by spheres (carbon=yellow, oxygen=red, nitrogen=blue). Panel B, polymorphic residues that lie in the inside of the substrate binding cavity of the enzyme are shown as spheres (carbon=yellow, oxygen=red, nitrogen=blue). Peptide substrates co-crystallized with ERAP1 are shown in stick representation (blue=10mer, orange=15mer).

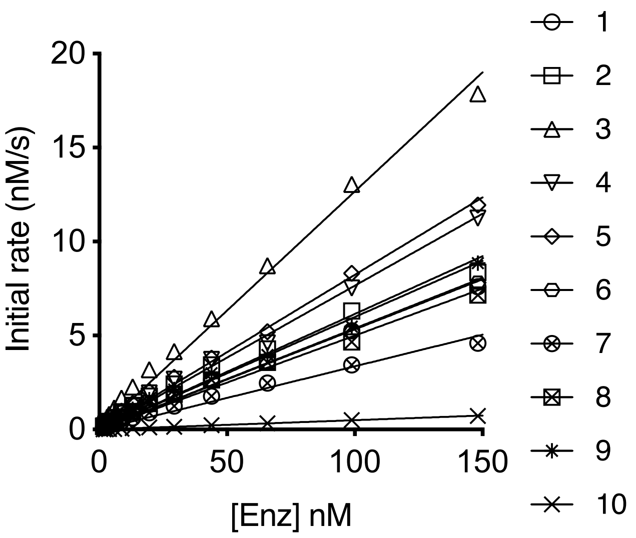

Supporting Figure 2: Initial rates of hydrolysis of Leu-AMC by ERAP1 allotypes plotted against enzyme concentration.

Supporting Table 7: Enzymatic parameters of each ERAP1 allotype versus small dipeptide substrates.

Leu-AMC substrate				Leu-pNA substrate		
**Allotype**	**Sp. Act. (s^{-1})**	**k_{cat}/K_M ($M^{-1} s^{-1}$)**	**V_{max} (s^{-1})**	**Hill coeff**	**K_{half} (mM)**	**K_{prime} (mM)**
**1**	0.0505±0.0011	2638 ± 32	2.95 ± 0.1	1.58 ± 0.09	0.85 ± 0.05	0.77 ± 0.08
**2**	0.0558±0.0025	2529 ± 18	2.8 ± 0.48	1.35 ± 0.31	0.95 ± 0.31	0.94 ± 0.42
**3**	0.1202±0.0026	4296 ± 49	4.98 ± 0.16	1.49 ± 0.08	0.8 ± 0.05	0.72 ± 0.08
**4**	0.0736±0.0019	3130 ± 15	10.47 ± 0.56	1.53 ± 0.07	1.7 ± 0.13	2.26 ± 0.19
**5**	0.0795±0.0014	3519 ± 29	8.07 ± 0.3	1.51 ± 0.06	1.4 ± 0.08	1.65 ± 0.12
**6**	0.0513±0.001	2273 ± 12	9.94 ± 0.79	1.43 ± 0.09	1.94 ± 0.23	2.57 ± 0.31
**7**	0.0311±0.0013	1706 ± 33	6.13 ± 0.35	1.46 ± 0.06	2.12 ± 0.17	2.99 ± 0.24
**8**	0.0463±0.0016	2492 ± 17	9.45 ± 0.35	1.6 ± 0.05	1.87 ± 0.1	2.72 ± 0.15
**9**	0.0568±0.0017	2747 ± 18	9.33 ± 0.32	1.55 ± 0.05	1.81 ± 0.09	2.52 ± 0.13
**10**	0.0049±0.0001	240 ± 5	nd	1.53 ± 0.08	nd	nd

Supporting Table 8: Calculated parameters from Michaelis-Menten analysis of trimming of the 9mer peptide YTAFTIPSI by ERAP1 allotypes.

	9mer peptide		
**Allotype**	**k_{cat} (s^{-1})**	**K_M (μM)**	**k_{cat}/K_M ($M^{-1} s^{-1}$)**
**1**	0.527 ± 0.014	6.5 ± 0.6	80523 ± 7251
**2**	0.553 ± 0.016	4.9 ± 0.5	113718 ± 12448
**3**	0.761 ± 0.021	13 ± 1	58480 ± 4601
**4**	0.655 ± 0.022	22.2 ± 1.8	29463 ± 2537
**5**	0.4 ± 0.015	14 ± 1.3	28478 ± 2938
**6**	0.403 ± 0.01	16.8 ± 1.1	23915 ± 1648
**7**	0.389 ± 0.027	25 ± 3.6	15567 ± 2466
**8**	0.489 ± 0.013	16 ± 1.1	30614 ± 2207
**9**	0.589 ± 0.018	23.8 ± 1.6	24717 ± 1826
**10**	0.051 ± 0.002	23.8 ± 1.4	2134 ± 144

Supporting Figure 3: Bubble chart showing the frequency of allotype combinations in the five human populations analyzed color-coded by their estimated activity. Allotypes have been clustered based on activity (highest activity top right of each panel, lowest bottom left).

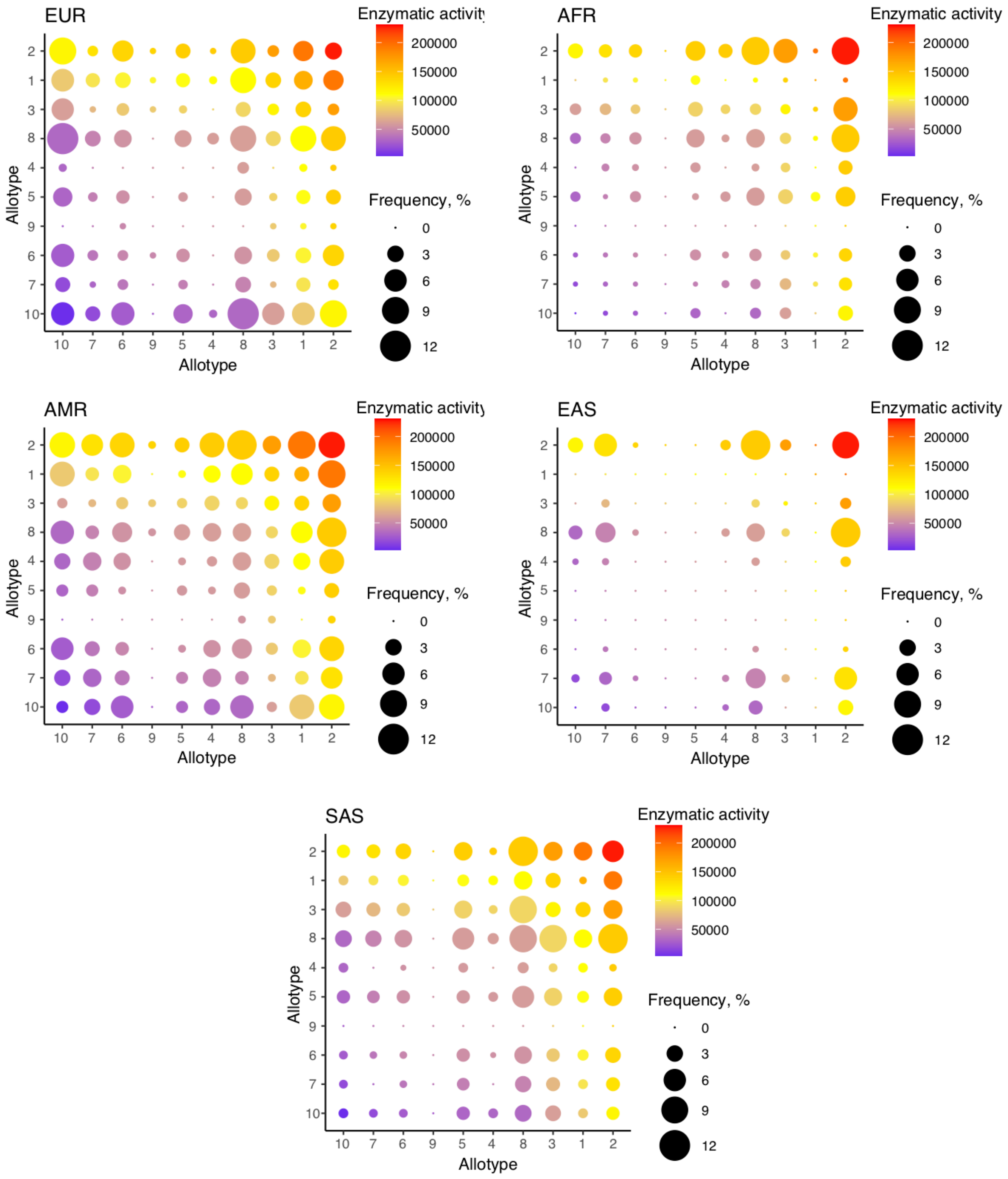

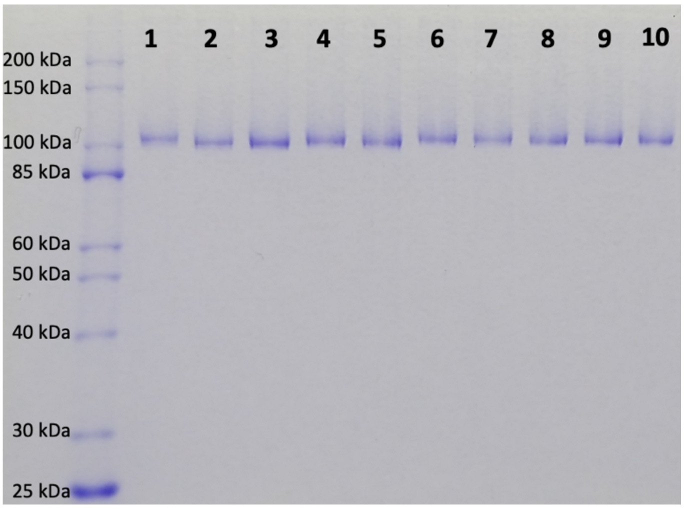

Supporting Figure 4: SDS-PAGE analysis of ERAP1 variants. 1μg of each ERAP1 allotype was analyzed on 8% SDS-PAGE under denaturing conditions and stained with Coomassie blue.

Haplotype 1

SEC-HPLC method details:
Injection: 20 ul
Flowrate: 0.7 ml/min
Column Name: TSKgel G3000SWxl
Column Size: 7.8 mm × 300 mm
Mobile Phase: 0.1 M Phosphate pH 6.7, 0.1 M Na2SO4

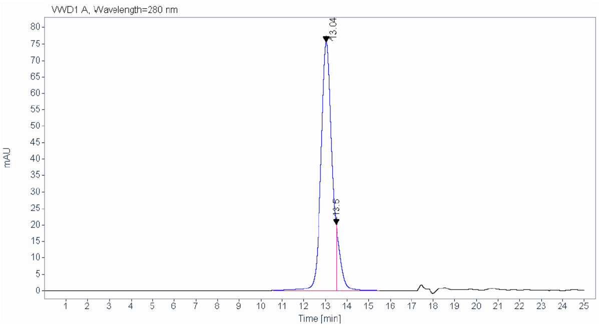

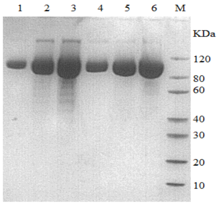

Lane1: 2µg reduced
Lane2: 5µg reduced
Lane3: 1µug reduced
Lane4: 2µg non-reduced
Lane5: 5µg non-reduced
Lane6: 10µg non-reduced

Signal: VWD1 A, Wavelength=280 nm

RT [min]	Height	Width [min]	Area	Area%
13.040	75.5494	0.5661	2566.0020	89.8514
13.500	19.9134	0.2426	289.8279	10.1486
		Sum	2855.8298	

Haplotype 2

SEC-HPLC method details:
Injection: 2 ul
Flowrate: 0.3 ml/min
Column Name: Agilent AdvanceBio SEC 300A
Column Size: 4.6 mm × 300 mm
Mobile Phase: 0.1 M Phosphate pH 6.7, 0.1 M Na2SO4

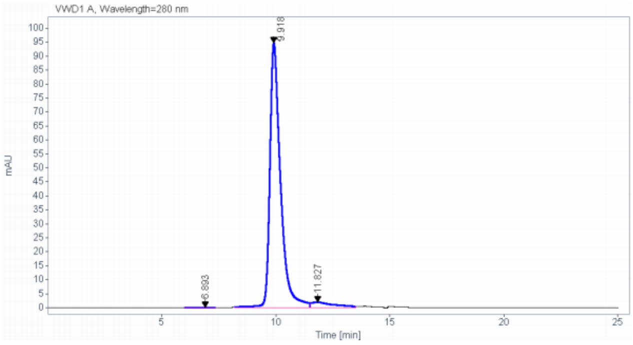

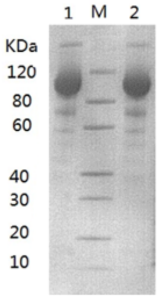

Lane1: 5µg reduced
Lane2: 5µg non-reduced

Signal: VWD1 A, Wavelength=280 nm

RT [min]	Height	Width [min]	Area	Area%
6.89	0.10	0.69	5.08	0.16
9.92	94.79	0.48	3086.78	95.25
11.83	2.00	0.98	148.97	4.60
		Sum	3240.8392	

Haplotype 3

SEC-HPLC method details (the same method conditions were used for all the remaining haplotypes):
Injection: 10 ul
Flowrate: 0.3 ml/min
Column Name: Agilent AdvanceBio SEC 300A
Column Size: 4.6 mm × 300 mm
Mobile Phase: 0.1 M Phosphate pH 6.7, 0.1 M Na2SO4

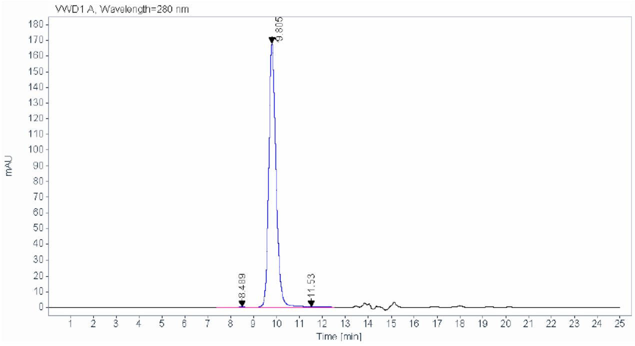

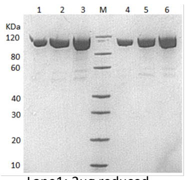

Lane1: 2µg reduced
Lane2: 5µg reduced
Lane3: 10µg reduced
Lane4: 2µg non-reduced
Lane5: 5µg non-reduced
Lane6: 10µg non-reduced

Signal: VWD1 A, Wavelength=280 nm

RT [min]	Height	Width [min]	Area	Area%
8.489	0.3415	0.4976	11.7558	0.3125
9.805	168.0251	0.3382	3718.0522	98.8410
11.530	0.6491	0.6683	31.8405	0.8464
		Sum	3761.6485	

Haplotype 4

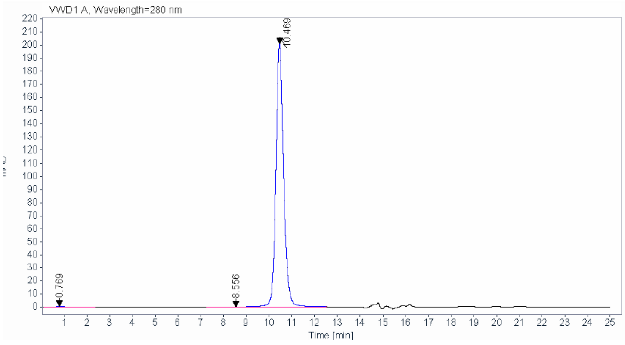

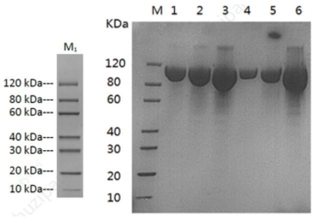

Lane1: 2µg reduced
Lane2: 5µg reduced
Lane3: 10µg reduced
Lane4: 2µg non-reduced
Lane5: 5µg non-reduced
Lane6: 10µg non-reduced

Signal: VWD1 A, Wavelength=280 nm

RT [min]	Height	Width [min]	Area	Area%
0.769	0.6721	0.4531	23.1060	0.4814
8.556	0.1879	0.8419	12.4032	0.2584
10.469	200.9883	0.3566	4764.4243	99.2602
		Sum	4799.9335	

Haplotype 5

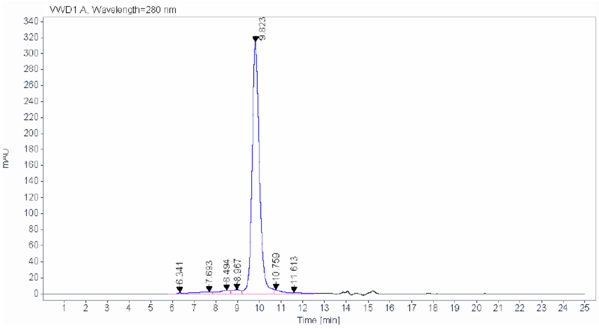

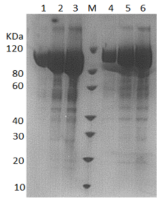

Lane1: 2μg reduced
Lane2: 5μg reduced
Lane3: 10μg reduced
Lane4: 2μg non-reduced
Lane5: 5μg non-reduced
Lane6: 10μg non-reduced

Signal: VWD1 A, Wavelength=280 nm

RT [min]	Height	Width [min]	Area	Area%
6.341	1.4471	0.2741	27.0561	0.3343
7.693	2.5107	0.8773	152.6399	1.8859
8.494	4.6340	0.5153	172.9045	2.1362
8.967	5.3442	0.3908	146.0127	1.8040
9.823	313.7916	0.3619	7367.9595	91.0306
10.759	5.0124	0.4010	148.2059	1.8311
11.613	1.8140	0.5971	79.1576	0.9780
		Sum	8093.9362	

Haplotype 6

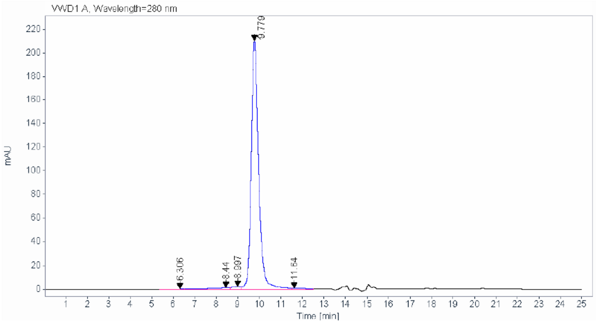

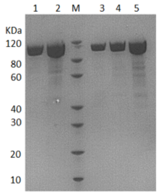

Lane1: 5μg reduced
Lane2: 10μg reduced
Lane3: 2μg non-reduced
Lane4: 5μg non-reduced
Lane5: 10μg non-reduced

Signal: VWD1 A, Wavelength=280 nm

RT [min]	Height	Width [min]	Area	Area%
6.306	0.1050	0.3904	3.0830	0.0595
8.440	1.3304	0.7971	82.6871	1.5949
8.997	2.1103	0.3722	54.2910	1.0472
9.779	210.1648	0.3658	5003.4678	96.5118
11.640	0.9541	0.5946	40.7794	0.7866
		Sum	5184.3082	

Haplotype 7

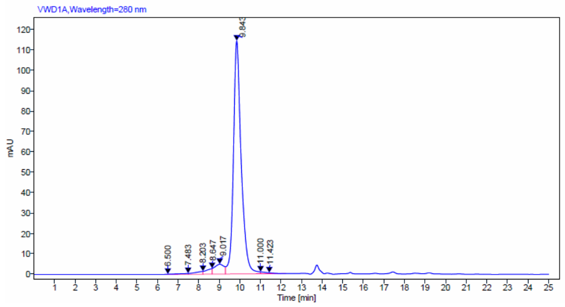

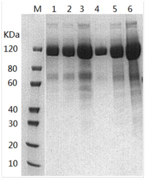

Lane1: 2μg reduced
Lane2: 5μg reduced
Lane3: 10μg reduced
Lane4: 2μg non-reduced
Lane5: 5μg non-reduced
Lane6: 10μg non-reduced

Signal: VWD1A,Wavelength=280 nm

RT [min]	Height	Width [min]	Area	Area%
6.500	0.14	0.29	2.46	0.07
7.483	0.41	0.56	13.67	0.42
8.203	1.36	0.45	37.01	1.13
8.647	2.78	0.33	54.48	1.66
9.017	4.80	0.47	154.42	4.71
9.843	114.32	0.38	2994.04	91.27
11.000	0.91	0.31	17.17	0.52
11.423	0.42	0.28	7.13	0.22
		Sum	3280.38	

Haplotype 8

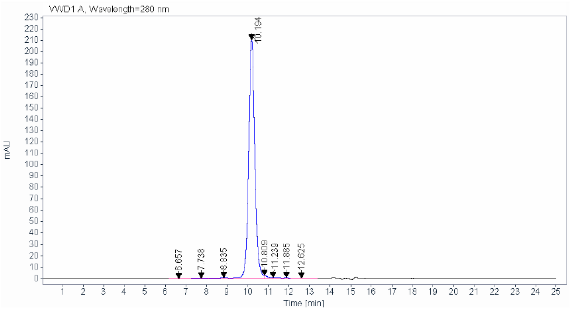

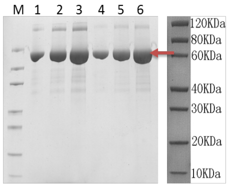

Lane1: 2μg reduced
Lane2: 5μg reduced
Lane3: 10μg reduced
Lane4: 2μg non-reduced
Lane5: 5μg non-reduced
Lane6: 10μg non-reduced

Signal: VWD1 A, Wavelength=280 nm

RT [min]	Height	Width [min]	Area	Area%
6.657	0.1027	0.4620	2.8461	0.0631
7.738	0.2536	0.7723	11.7513	0.2607
8.835	0.8666	0.5356	27.8489	0.6178
10.194	210.3143	0.3481	4392.2275	97.4432
10.809	2.5075	0.2251	33.8657	0.7513
11.239	0.6793	0.3882	15.8229	0.3510
11.885	0.5261	0.5204	16.4279	0.3645
12.625	0.1973	0.5649	6.6855	0.1483
		Sum	4507.4758	

Haplotype 9

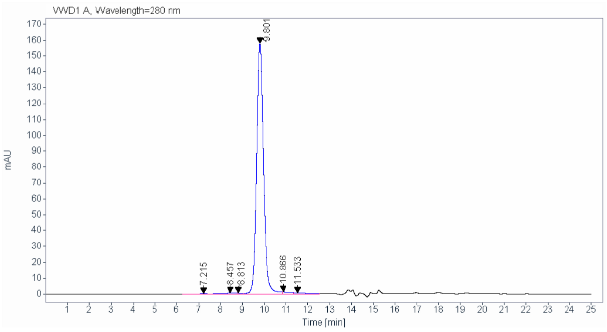

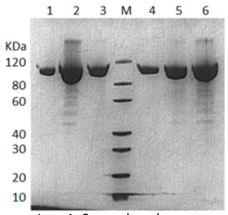

Lane1: 2μg reduced
Lane2: 10μg reduced
Lane3: 5μg reduced
Lane4: 2μg non-reduced
Lane5: 5μg non-reduced
Lane6: 10μg non-reduced

Signal: VWD1 A, Wavelength=280 nm

RT [min]	Height	Width [min]	Area	Area%
7.215	0.1311	0.5748	5.4714	0.1555
8.457	0.7014	0.4833	24.2507	0.6894
8.813	0.4861	0.2305	7.6036	0.2161
9.801	157.8495	0.3403	3413.2371	97.0249
10.866	1.2397	0.3655	33.7368	0.9590
11.533	0.7542	0.5837	33.5972	0.9550
		Sum	3517.8967	

Haplotype 10

0 cycles freeze-thaw SEC-HPLC

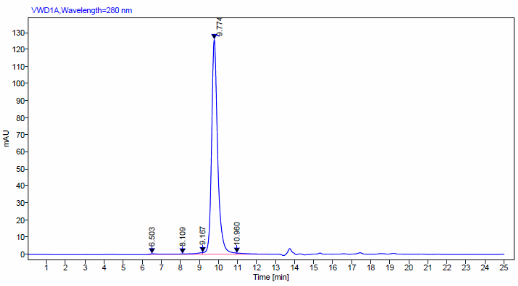

Signal: VWD1A,Wavelength=280 nm

RT [min]	Height	Width [min]	Area	Area%
6.503	0.47	0.60	22.50	0.78
8.109	0.30	0.30	7.48	0.26
9.167	1.02	0.50	30.48	1.06
9.774	126.03	0.33	2794.84	97.02
10.960	0.71	0.59	25.28	0.88
		Sum	2880.58	

1 cycles freeze-thaw SEC-HPLC

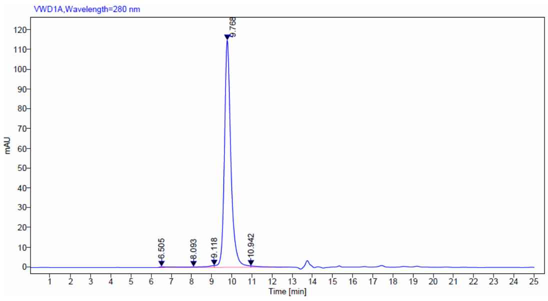

Signal: VWD1A,Wavelength=280 nm

RT [min]	Height	Width [min]	Area	Area%
6.505	0.39	0.62	18.80	0.71
8.093	0.26	0.30	6.42	0.24
9.118	0.86	0.48	24.60	0.93
9.768	114.66	0.33	2561.44	97.23
10.942	0.64	0.60	23.16	0.88
		Sum	2634.43	

Haplotype 10

3 cycles freeze-thaw SEC-HPLC

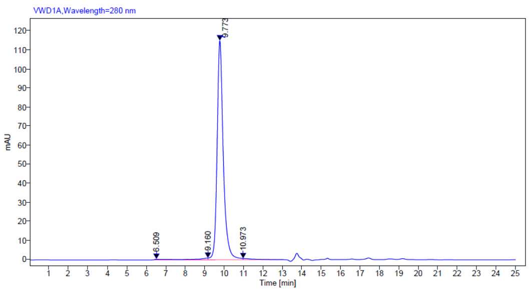

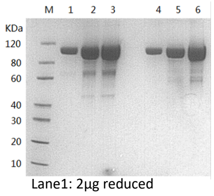

Lane1: 2µg reduced
Lane2: 5µg reduced
Lane3: 10µg reduced
Lane4: 2µg non-reduced
Lane5: 5µg non-reduced
Lane6: 10µg non-reduced

Signal: VWD1A,Wavelength=280 nm

RT [min]	Height	Width [min]	Area	Area%
6.509	0.38	0.63	19.31	0.74
9.160	0.90	0.62	33.49	1.28
9.773	114.97	0.33	2548.51	97.03
10.973	0.64	0.66	25.19	0.96
		Sum	2626.50	

Supporting Figure 5: Size-exclusion chromatographic analysis (SEC) and SDS-PAGE analysis of ERAP1 allotypes. Proteins were loaded up to 10µg to reveal purity. For allotype 10 the sample was analyzed by SEC and after 3 freeze-thaw cycles to test stability versus aggregation.

Supporting Figure 6: DNA sequencing results for all ERAP1 allotypes; forward and reverse sequencing primers are indicated with green and red arrows respectively. SNPs defining different ERAP1 allotypes are highlighted in yellow in the consensus sequences with the corresponding allotype 1 amino acid substitutions written above each SNP**.**

10 20 30 40 50 60 70 80 90 100

GGAGATCGGAAGACCTCGGCCGTCGCGGCGCTTGCCGGTGGTGCTGACCCCGGATGAAGTGGTTCGCATCCTCGGTTTTCTGGAAGGCGAGCATCGTTTGTTCGCCC

A09-SEQ1R.ab1(1>820)

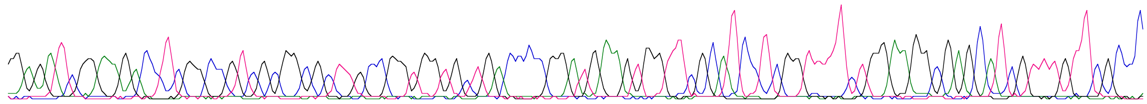

GGAGATCGGAAGACCTCGGCCGTCGCGGCGCTTGCCGGTGGTGCTGACCCCGGATGAAGTGGTTCGCATCCTCGGTTTTCTGGAAGGCGAGCATCGTTTGTTCGCCC

110 120 130 140 150 160 170 180 190 200 210

AGGACTCTAGCTATAGTTCTAGTGGTTGGCTACGTATACTCCGGAATATTAATAGATCATGGAGATAATTAAAATGATAACCATCTCGCAAATAAATAAGTATTTTA

A09-SEQ1R.ab1(1>820)

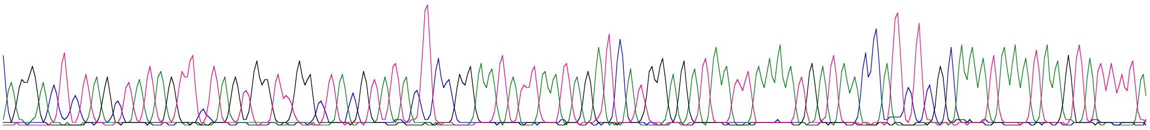

AGGACTCTAGCTATAGTTCTAGTGGTTGGCTACGTATACTCCGGAATATTAATAGATCATGGAGATAATTAAAATGATAACCATCTCGCAAATAAATAAGTATTTTA

220 230 240 250 260 270 280 290 300 310 320

CTGTTTTCGTAACAGTTTTGTAATAAAAAAACCTATAAATATTCCGGATTATTCATACCGTCCCACCATCGGGCGCGGATCCAACTCCTAAAAAACCGCCACCATGG

U3823DH140-8.seq(1>2878)
A09-SEQ1R.ab1(1>820)

GGATCCAACTCCTAAAAAACCGCCACCATGG

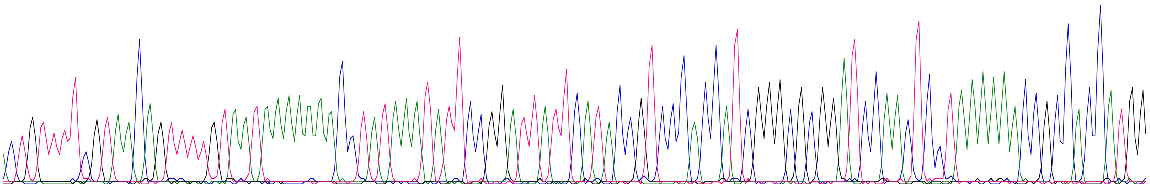

CTGTTTTCGTAACAGTTTTGTAATAAAAAAACCTATAAATATTCCGGATTATTCATACCGTCCCACCATCGGGCGCGGATCCAACTCCTAAAAAACCGCCACCATGG

D03-pFastbac-F.ab1(1>819)

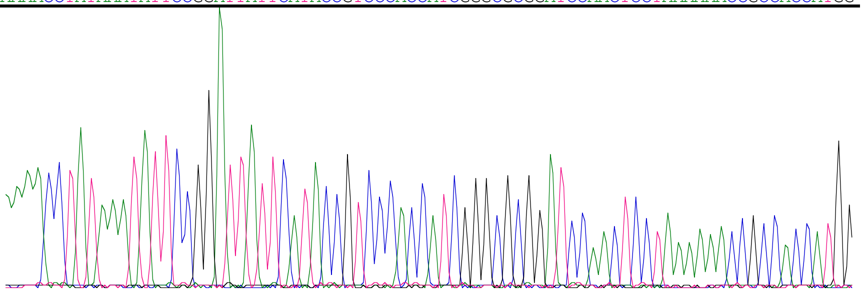

AAAACCTATAAATATTCCGGATTATTCATACCGTCCCACCATCGGGCGCGGATCCAACTCCTAAAAAACCGCCACCATGG

330 340 350 360 370 380 390 400 410 420

TGTTTCTGCCCCTCAAATGGTCCCTTGCAACCATGTCATTTCTACTTTCCTCACTGTTGGCTCTCTTAACTGTGTCCACTCCTTCATGGTGTCAGAGCACTGAAGCA

U3823DH140-8.seq(1>2878)
A09-SEQ1R.ab1(1>820)

TGTTTCTGCCCCTCAAATGGTCCCTTGCAACCATGTCATTTCTACTTTCCTCACTGTTGGCTCTCTTAACTGTGTCCACTCCTTCATGGTGTCAGAGCACTGAAGCA

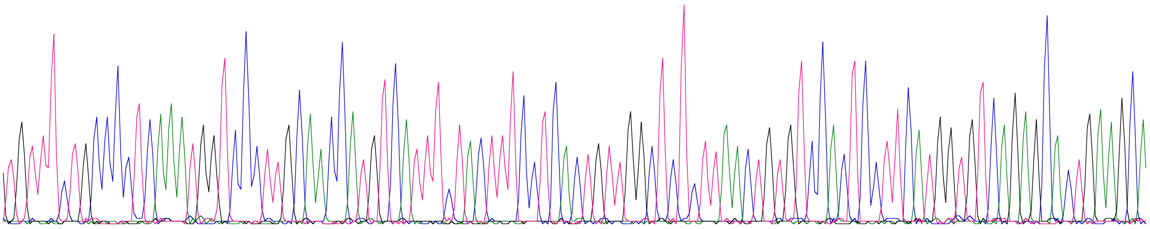

TGTTTCTGCCCCTCAAATGGTCCCTTGCAACCATGTCATTTCTACTTTCCTCACTGTTGGCTCTCTTAACTGTGTCCACTCCTTCATGGTGTCAGAGCACTGAAGCA

330 340 350 360 370 380 390 400 410 420

TGTTTCTGCCCCTCAAATGGTCCCTTGCAACCATGTCATTTCTACTTTCCTCACTGTTGGCTCTCTTAACTGTGTCCACTCCTTCATGGTGTCAGAGCACTGAAGCA

D03-pFastbac-F.ab1(1>819)

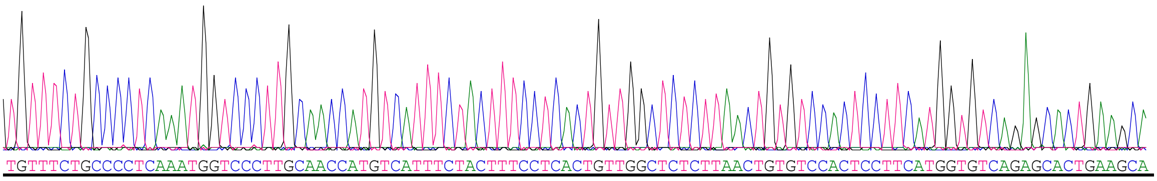

430 440 450 460 470 480 490 500 510 520 530

TCTCCAAAACGTAGTGATGGGACACCATTTCCTTGGAATAAAATACGACTTCCTGAGTACGTCATCCCAGTTCATTATGATCTCTTGATCCATGCAAACCTTACCAC

U3823DH140-8.seq(1>2878)
A09-SEQ1R.ab1(1>820)

TCTCCAAAACGTAGTGATGGGACACCATTTCCTTGGAATAAAATACGACTTCCTGAGTACGTCATCCCAGTTCATTATGATCTCTTGATCCATGCAAACCTTACCAC

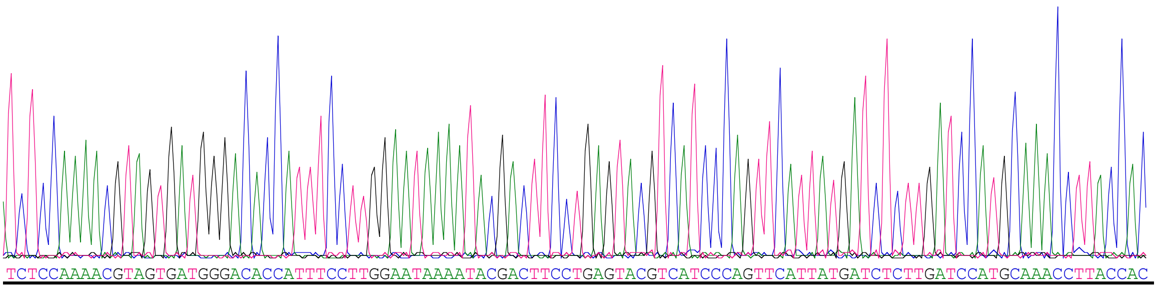

D03-pFastbac-F.ab1(1>819)

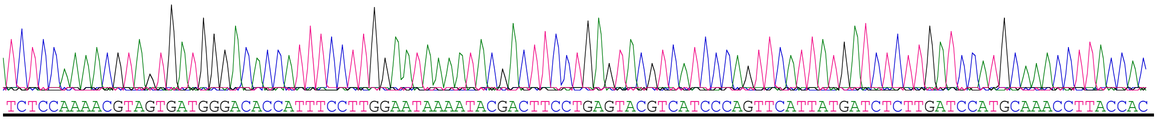

540 550 560 570 580 590 600 610 620 630 640

GCTGACCTTCTGGGGAACCACGAAAGTAGAAATCACAGCCAGTCAGCCCACCAGCACCATCATCCTGCATAGTCACCACCTGCAGATATCTAGGGCCACCCTCAGGA

U3823DH140-8.seq(1>2878)
A09-SEQ1R.ab1(1>820)

GCTGACCTTCTGGGGAACCACGAAAGTAGAAATCACAGCCAGTCAGCCCACCAGCACCATCATCCTGCATAGTCACCACCTGCAGATATCTAGGGCCACCCTCAGGA

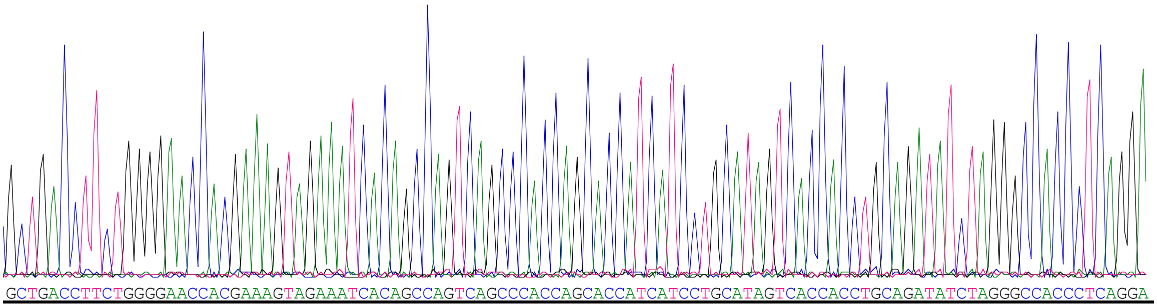

D03-pFastbac-F.ab1(1>819)

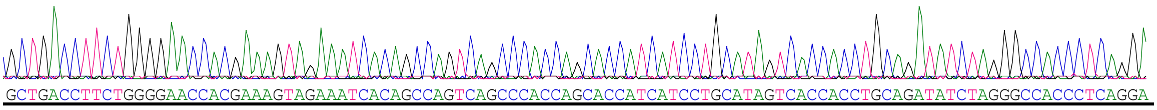

650 660 670 680 690 700 710 720 730 740

AGGGAGCTGGAGAGAGGCTATCGGAAGAACCCCTGCAGGTCCTGGAACACCCCCCGCAGGAGCAAATTGCACTGCTGGCTCCCGAGCCCCTCCTTGTCGGGCTCCCG

U3823DH140-8.seq(1>2878) AGGGAGCTGGAGAGAGGCTATCGGAAGAACCCCTGCAGGTCCTGGAACACCCCCCGCAGGAGCAAATTGCACTGCTGGCTCCCGAGCCCCTCCTTGTCGGGCTCCCG

A09-SEQ1R.ab1(1>820)

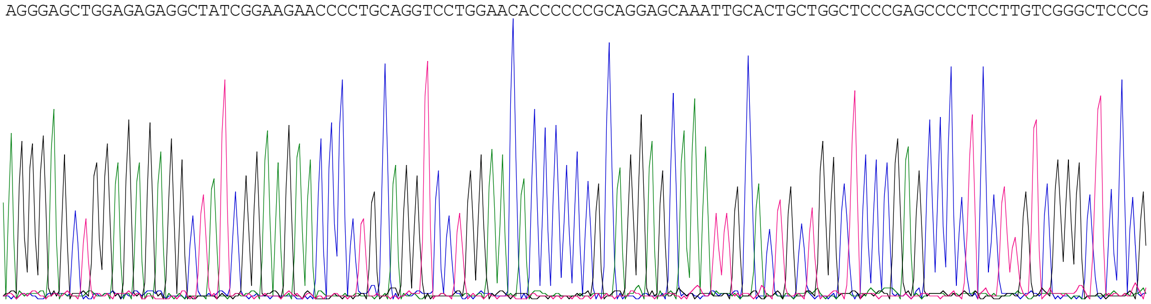

AGGGAGCTGGAGAGAGGCTATCGGAAGAACCCCTGCAGGTCCTGGAACACCCCCCGCAGGAGCAAATTGCACTGCTGGCTCCCGAGCCCCTCCTTGTCGGGCTCCCG

D03-pFastbac-F.ab1(1>819)

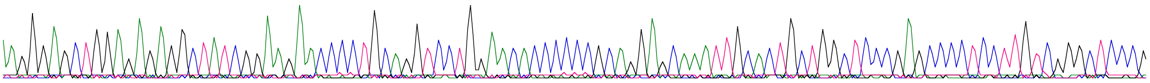

AGGGAGCTGGAGAGAGGCTATCGGAAGAACCCCTGCAGGTCCTGGAACACCCCCCGCAGGAGCAAATTGCACTGCTGGCTCCCGAGCCCCTCCTTGTCGGGCTCCCG

A06-SEQR.ab1(1>745)

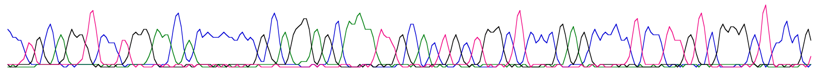

CCTGCAGGTCCTGGAACACCCCCCGCAGGAGCAAATTGCACTGCTGGCTCCCGAGCCCCTCCTTGTCGGGCTCCCG

750 760 770 780 790 800 810 820 830 840 850

TACACAGTTGTCATTCACTATGCTGGCAATCTTTCGGAGACTTTCCACGGATTTTACAAAAGCACCTACAGAACCAAGGAAGGGGAACTGAGGATACTAGCATCAAC

U3823DH140-8.seq(1>2878) TACACAGTTGTCATTCACTATGCTGGCAATCTTTCGGAGACTTTCCACGGATTTTACAAAAGCACCTACAGAACCAAGGAAGGGGAACTGAGGATACTAGCATCAAC

A09-SEQ1R.ab1(1>820)

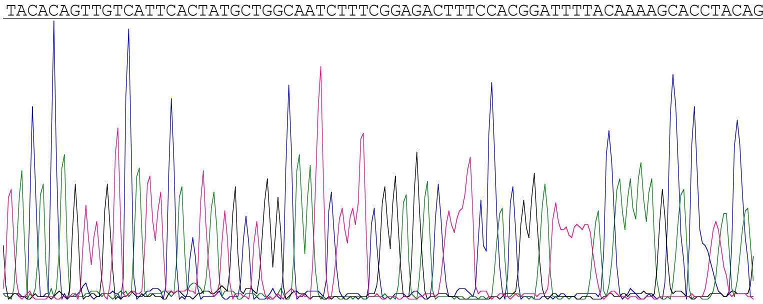

TACACAGTTGTCATTCACTATGCTGGCAATCTTTCGGAGACTTTCCACGGATTTTACAAAAGCACCtACAG

D03-pFastbac-F.ab1(1>819)

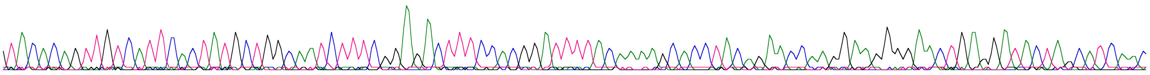

TACACAGTTGTCATTCACTATGCTGGCAATCTTTCGGAGACTTTCCACGGATTTTACAAAAGCACCTACAGAACCAAGGAAGGGGAACTGAGGATACTAGCATCAAC

A06-SEQR.ab1(1>745)

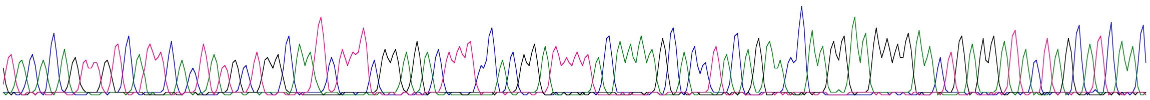

TACACAGTTGTCATTCACTATGCTGGCAATCTTTCGGAGACTTTCCACGGATTTTACAAAAGCACCTACAGAACCAAGGAAGGGGAACTGAGGATACTAGCATCAAC

750 760 770 780 790 800 810 820 830 840 850

TACACAGTTGTCATTCACTATGCTGGCAATCTTTCGGAGACTTTCCACGGATTTTACAAAAGCACCTACAGAACCAAGGAAGGGGAACTGAGGATACTAGCATCAAC

D05-SEQ1.ab1(1>819) →

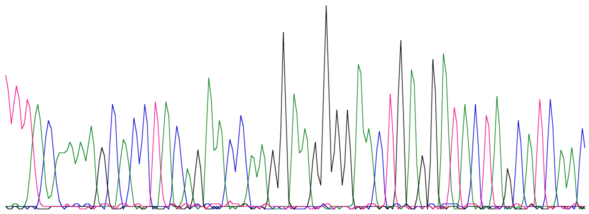

TTTACAAAAGCACCTACAGAACCAAGGAAGGGGAACTGAGGATACTAGCATCAAC

860 870 880 890 900 910 920 930 940 950 960

ACAATTTGAACCCACTGCAGCTAGAATGGCCTTTCCCTGCTTTGATGAACCTGCCTTCAAAGCAAGTTTCTCAATCAAAATTAGAAGAGAGCCAAGGCACCTAGCCA

U3823DH140-8.seq(1>2878) → ACAATTTGAACCCACTGCAGCTAGAATGGCCTTTCCCTGCTTTGATGAACCTGCCTTCAAAGCAAGTTTCTCAATCAAAATTAGAAGAGAGCCAAGGCACCTAGCCA

D03-pFastbac-F.ab1(1>819) →

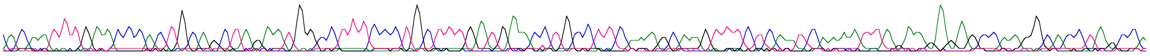

ACAATTTGAACCCACTGCAGCTAGAATGGCCTTTCCCTGCTTTGATGAACCTGCCTTCAAAGCAAGTTTCTCAATCAAAATTAGAAGAGAGCCAAGGCACCTAGCCA

A06-SEQR.ab1(1>745) ←

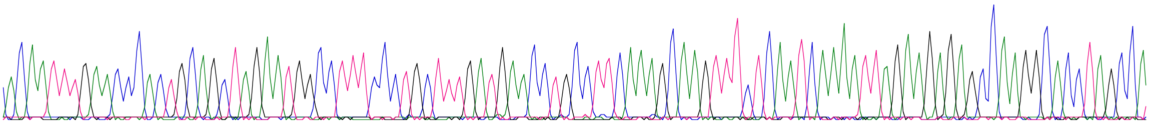

ACAATTTGAACCCACTGCAGCTAGAATGGCCTTTCCCTGCTTTGATGAACCTGCCTTCAAAGCAAGTTTCTCAATCAAAATTAGAAGAGAGCCAAGGCACCTAGCCA

D05-SEQ1.ab1(1>819) →

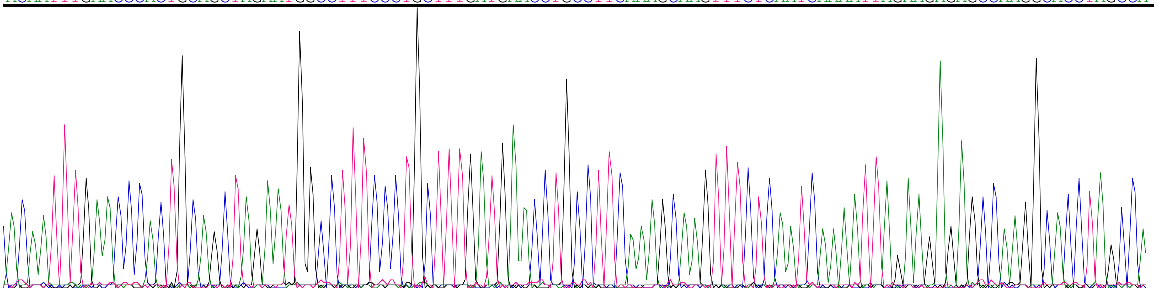

ACAATTTGAACCCACTGCAGCTAGAATGGCCTTTCCCTGCTTTGATGAACCTGCCTTCAAAGCAAGTTTCTCAATCAAAATTAGAAGAGAGCCAAGGCACCTAGCCA

970 980 990 1,000 1,010 1,020 1,030 1,040 1,050 1,060 1,070

TCTCCAATATGCCATTGGTGAAATCTGTGACTGTTGCTGAAGGACTCATAGAAGACCATTTTGATGTCACTGTGAAGATGAGCACCTATCTGGTGGCCTTCATCATT

U3823DH140-8.seq(1>2878) → TCTCCAATATGCCATTGGTGAAATCTGTGACTGTTGCTGAAGGACTCATAGAAGACCATTTTGATGTCACTGTGAAGATGAGCACCTATCTGGTGGCCTTCATCATT

D03-pFastbac-F.ab1(1>819) →

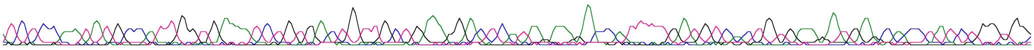

TCTCCAATATGCCATTGGTGAAATCTGTGACTGTTGCTGAAGGACTCATAGAAGACCATTTTGATGTCACTGTGAAGATGAGCACCTATCTGGTGGC

970 980 990 1,000 1,010 1,020 1,030 1,040 1,050 1,060 1,070

TCTCCAATATGCCATTGGTGAAATCTGTGACTGTTGCTGAAGGACTCATAGAAGACCATTTTGATGTCACTGTGAAGATGAGCACCTATCTGGTGGCCTTCATCATT

A06-SEQR.ab1(1>745) ←

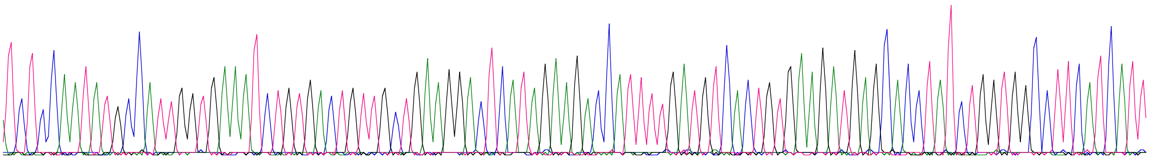

TCTCCAATATGCCATTGGTGAAATCTGTGACTGTTGCTGAAGGACTCATAGAAGACCATTTTGATGTCACTGTGAAGATGAGCACCTATCTGGTGGCCTTCATCATT

D05-SEQ1.ab1(1>819) →

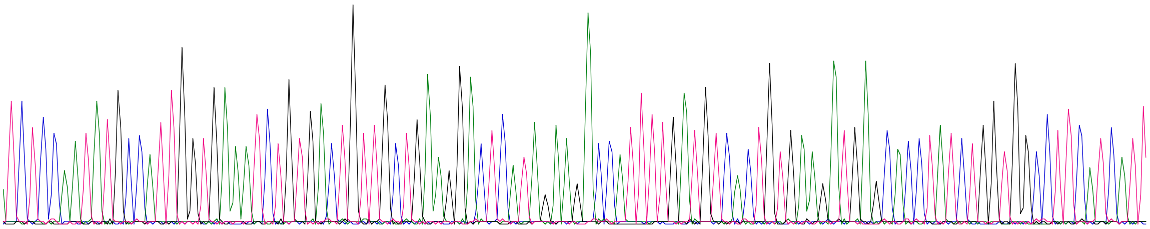

TCTCCAATATGCCATTGGTGAAATCTGTGACTGTTGCTGAAGGACTCATAGAAGACCATTTTGATGTCACTGTGAAGATGAGCACCTATCTGGTGGCCTTCATCATT

1,080 1,090 1,100 1,110 1,120 1,130 1,140 1,150 1,160 1,170

TCAGATTTTGAGTCTGTCAGCAAGATAACCAAGAGTGGAGTCAAGGTTTCTGTTTATGCTGTGCCAGACAAGATAAATCAAGCAGATTATGCACTGGATGCTGCGGT

U3823DH140-8.seq(1>2878) → TCAGATTTTGAGTCTGTCAGCAAGATAACCAAGAGTGGAGTCAAGGTTTCTGTTTATGCTGTGCCAGACAAGATAAATCAAGCAGATTATGCACTGGATGCTGCGGT

A06-SEQR.ab1(1>745) ←

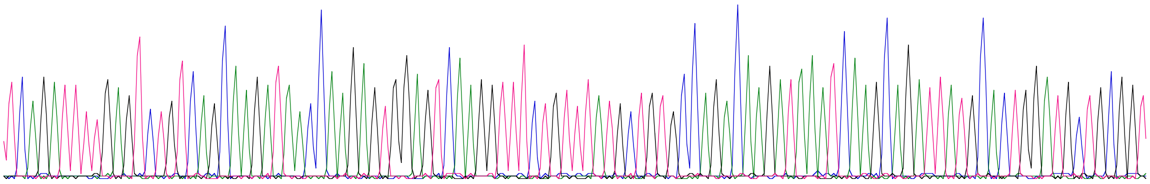

TCAGATTTTGAGTCTGTCAGCAAGATAACCAAGAGTGGAGTCAAGGTTTCTGTTTATGCTGTGCCAGACAAGATAAATCAAGCAGATTATGCACTGGATGCTGCGGT

D05-SEQ1.ab1(1>819) →

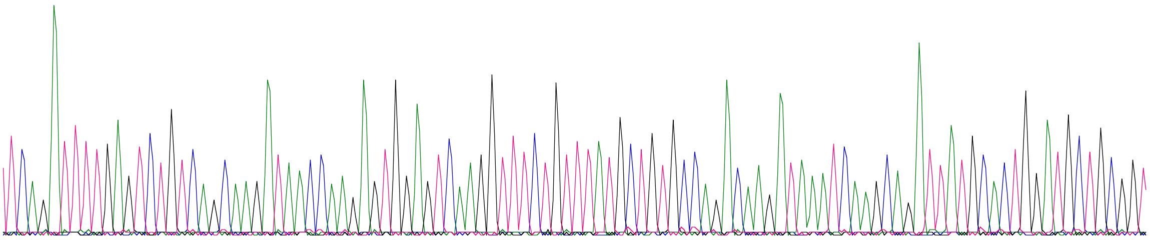

TCAGATTTTGAGTCTGTCAGCAAGATAACCAAGAGTGGAGTCAAGGTTTCTGTTTATGCTGTGCCAGACAAGATAAATCAAGCAGATTATGCACTGGATGCTGCGGT

1,180 1,190 1,200 1,210 1,220 1,230 1,240 1,250 1,260 1,270 1,280

GACTCTTCTAGAATTTTATGAGGATTATTTCAGCATACCGTATCCCCTACCCAAACAAGATCTTGCTGCTATTCCCGACTTTCAGTCTGGTGCTATGGAAAACTGGG

U3823DH140-8.seq(1>2878) → GACTCTTCTAGAATTTTATGAGGATTATTTCAGCATACCGTATCCCCTACCCAAACAAGATCTTGCTGCTATTCCCGACTTTCAGTCTGGTGCTATGGAAAACTGGG

A06-SEQR.ab1(1>745) ←

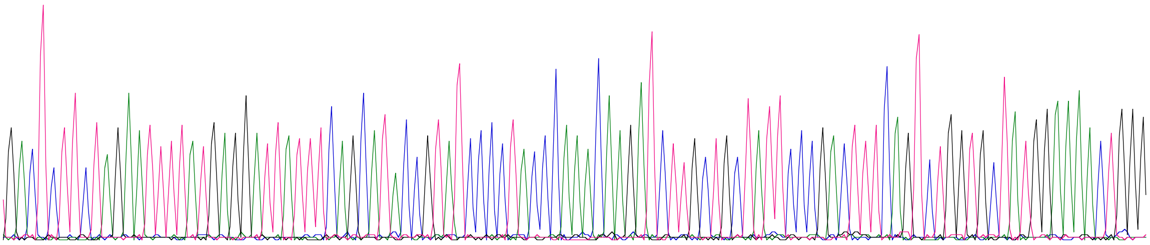

GACTCTTCTAGAATTTTATGAGGATTATTTCAGCATACCGTATCCCCTACCCAAACAAGATCTTGCTGCTATTCCCGACTTTCAGTCTGGTGCTATGGAAAACTGGG

1,180 1,190 1,200 1,210 1,220 1,230 1,240 1,250 1,260 1,270 1,280

GACTCTTCTAGAATTTTATGAGGATTATTTCAGCATACCGTATCCCCTACCCAAACAAGATCTTGCTGCTATTCCCGACTTTCAGTCTGGTGCTATGGAAAACTGGG

D05-SEQ1.ab1(1>819)

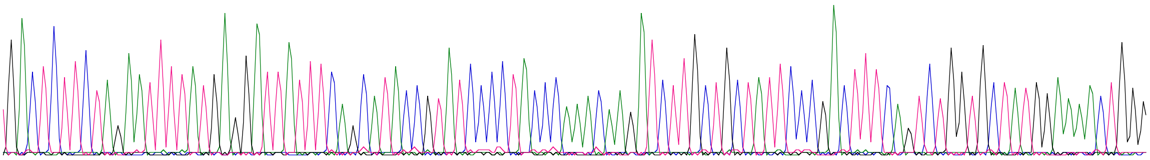

GACTCTTCTAGAATTTTATGAGGATTATTTCAGCATACCGTATCCCCTACCCAAACAAGATCTTGCTGCTATTCCCGACTTTCAGTCTGGTGCTATGGAAAACTGGG

A11-SEQ3R.ab1(1>814)

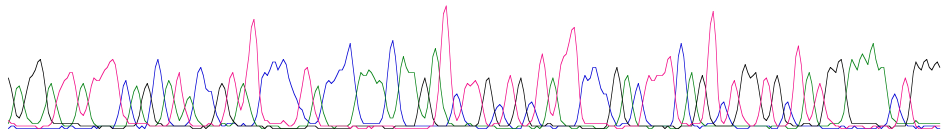

GAGGATTATTTCAGCATACCGTATCCCCTACCCAAACAAGATCTTGCTGCTATTCCCGACTTTCAGTCTGGTGCTATGGAAAACTGGG

B01-SEQ2.ab1(1>819)

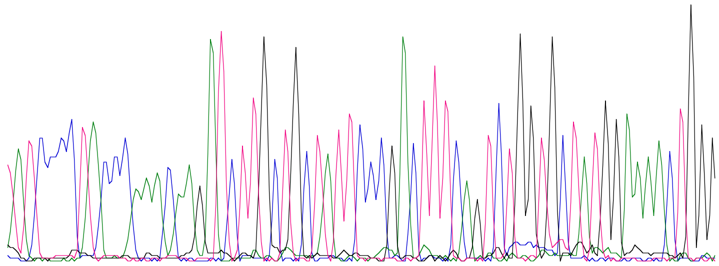

TATCCCCTACCCAAACAAGATCTTGcTGCTATTCCCGACTTTCAGTCTGGTGCTATGGAAAACTGGG

1,290 1,300 1,310 1,320 1,330 1,340 1,350 1,360 1,370 1,380 1,390

GACTGACAACATATAGAGAATCTGCTCTGTTGTTTGATGCAGAAAAGTCTTCTGCATCAAGTAAGCTTGGCATCACAATGACTGTGGCCCATGAACTGGCTCACCAG

U3823DH140-8.seq(1>2878) GACTGACAACATATAGAGAATCTGCTCTGTTGTTTGATGCAGAAAAGTCTTCTGCATCAAGTAAGCTTGGCATCACAATGACTGTGGCCCATGAACTGGCTCACCAG

A06-SEQR.ab1(1>745)

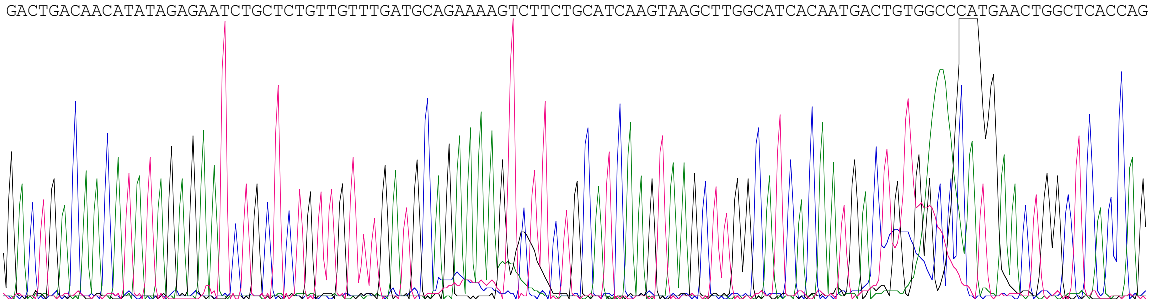

GACTGACAACATATAGAGAATCTGCTCTGTTGTTTGATGCAGAAAAGTCTTCTGCATCAAGTAAGCTTGGCATCACAATGACTGTGGCCCATGAACTGGCTCACCAG

D05-SEQ1.ab1(1>819)

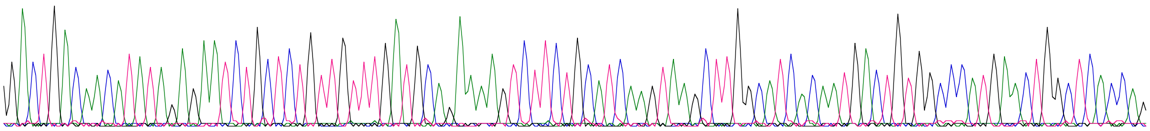

GACTGACAACATATAGAGAATCTGCTCTGTTGTTTGATGCAGAAAAGTCTTCTGCATCAAGTAAGCTTGGCATCACAATGACTGTGGCCCATGAACTGGCTCACCAG

A11-SEQ3R.ab1(1>814)

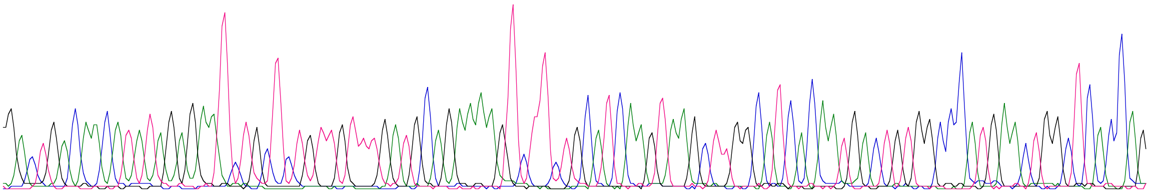

GACTGACAACATATAGAGAATCTGCTCTGTTGTTTGATGCAGAAAAGTCTTCTGCATCAAGTAAGCTTGGCATCACAATGACTGTGGCCCATGAACTGGCTCACCAG

1,290 1,300 1,310 1,320 1,330 1,340 1,350 1,360 1,370 1,380 1,390

GACTGACAACATATAGAGAATCTGCTCTGTTGTTTGATGCAGAAAAGTCTTCTGCATCAAGTAAGCTTGGCATCACAATGACTGTGGCCCATGAACTGGCTCACCAG

B01-SEQ2.ab1(1>819)

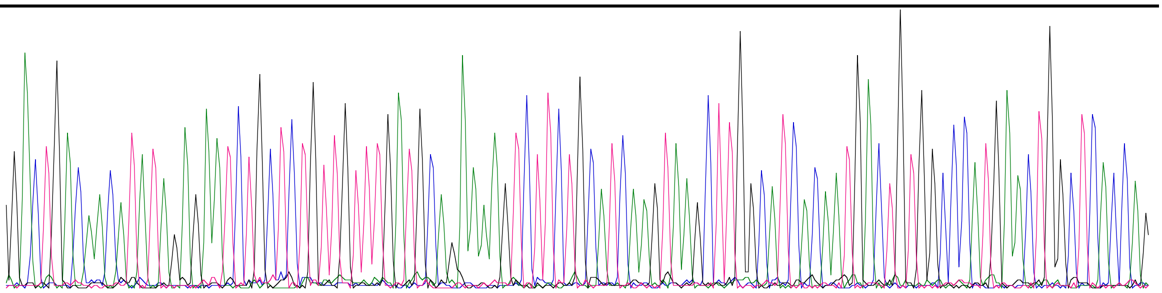

GACTGACAACATATAGAGAATCTGCTCTGTTGTTTGATGCAGAAAAGTCTTCTGCATCAAGTAAGCTTGGCATCACAATGACTGTGGCCCATGAACTGGCTCACCAG

1,400 1,410 1,420 1,430 1,440 1,450 1,460 1,470 1,480 1,490

TGGTTTGGGAACCTGGTCACTATGGAATGGTGGAATGATCTTTGGCTAAATGAAGGATTTGCCAAATTTATGGAGTTTGTGTCTGTCAGTGTGACCCATCCTGAACT

U3823DH140-8.seq(1>2878) TGGTTTGGGAACCTGGTCACTATGGAATGGTGGAATGATCTTTGGCTAAATGAAGGATTTGCCAAATTTATGGAGTTTGTGTCTGTCAGTGTGACCCATCCTGAACT

A06-SEQR.ab1(1>745)

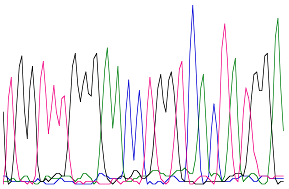

TGGTTTGGGAACCTGGTCACTATGGAA

D05-SEQ1.ab1(1>819)

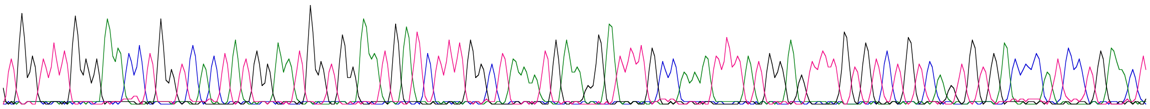

TGGTTTGGGAACCTGGTCACTATGGAATGGTGGAATGATCTTTGGCTAAATGAAGGATTTGCCAAATTTATGGAGTTTGTGTCTGTCAGTGTGACCCATCCTGAACT

A11-SEQ3R.ab1(1>814)

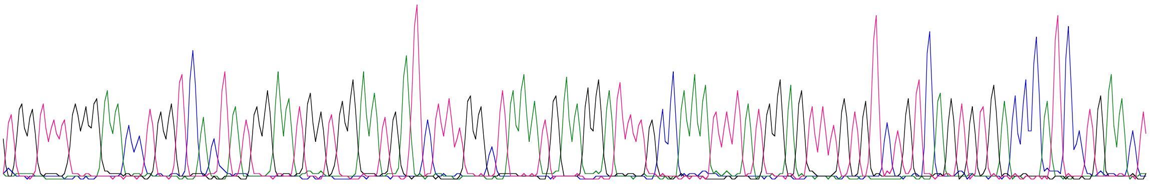

TGGTTTGGGAACCTGGTCACTATGGAATGGTGGAATGATCTTTGGCTAAATGAAGGATTTGCCAAATTTATGGAGTTTGTGTCTGTCAGTGTGACCCATCCTGAACT

B01-SEQ2.ab1(1>819)

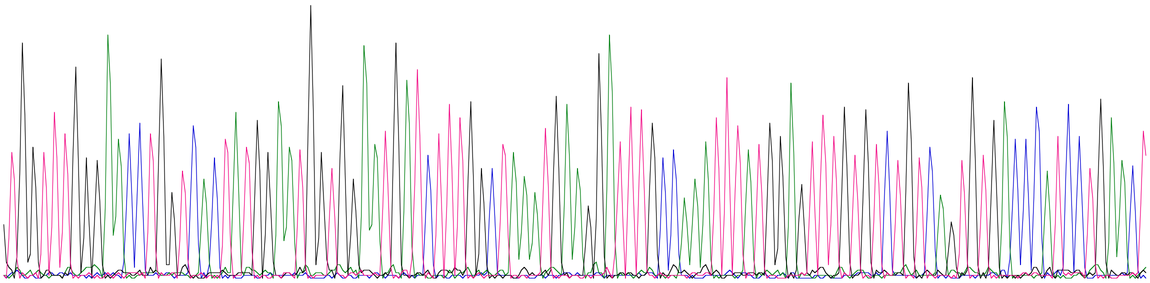

TGGTTTGGGAACCTGGTCACTATGGAATGGTGGAATGATCTTTGGCTAAATGAAGGATTTGCCAAATTTATGGAGTTTGTGTCTGTCAGTGTGACCCATCCTGAACT

1,500 1,510 1,520 1,530 1,540 1,550 1,560 1,570 1,580 1,590 1,600

GAAAGTTGGAGATTATTTCTTTGGCAAATGTTTTGACGCAATGGAGGTAGATGCTTTAAATTCCTCACACCCTGTGTCTACACCTGTGGAAAATCCTGCTCAGATCC

U3823DH140-8.seq(1>2878) → GAAAGTTGGAGATTATTTCTTTGGCAAATGTTTTGACGCAATGGAGGTAGATGCTTTAAATTCCTCACACCCTGTGTCTACACCTGTGGAAAATCCTGCTCAGATCC

D05-SEQ1.ab1(1>819) →

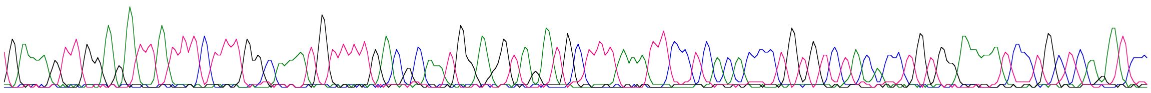

GAAAGTTGGAGATTATTTCTTTGGCAAATGTTTTGACGCAATGGAGGTAGATGCTTTAAATTCCTCACACCCTGTGTCTACACCTGTGGAAAATCCTGCTCAGATCC

A11-SEQ3R.ab1(1>814) ←

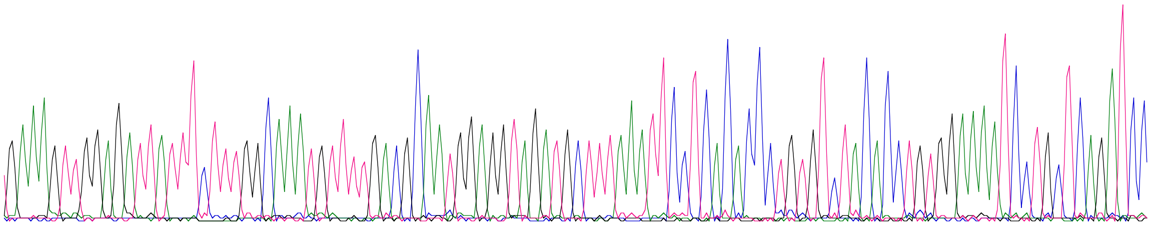

GAAAGTTGGAGATTATTTCTTTGGCAAATGTTTTGACGCAATGGAGGTAGATGCTTTAAATTCCTCACACCCTGTGTCTACACCTGTGGAAAATCCTGCTCAGATCC

B01-SEQ2.ab1(1>819) →

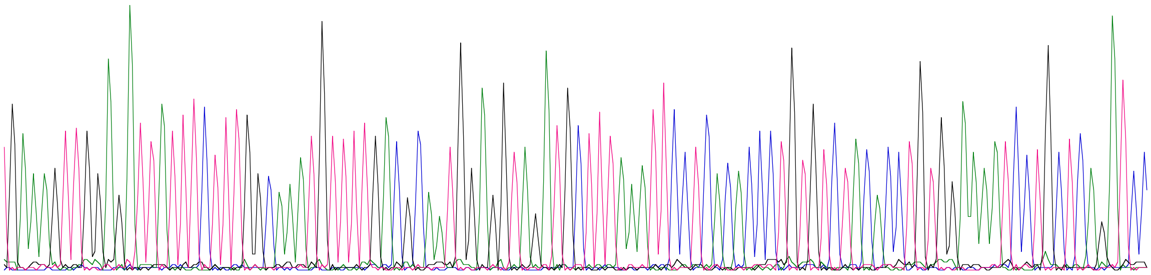

GAAAGTTGGAGATTATTTCTTTGGCAAATGTTTTGACGCAATGGAGGTAGATGCTTTAAATTCCTCACACCCTGTGTCTACACCTGTGGAAAATCCTGCTCAGATCC

1,610 1,620 1,630 1,640 1,650 1,660 1,670 1,680 1,690 1,700 1,710

GGGAGATGTTTGATGATGTTTCTTATGATAAGGGAGCTTGTATTCTGAATATGCTAAGGGAGTATCTTAGTGCTGACGCATTTAAAAGTGGTATTGTACAGTATCTC

U3823DH140-8.seq(1>2878) → GGGAGATGTTTGATGATGTTTCTTATGATAAGGGAGCTTGTATTCTGAATATGCTAAGGGAGTATCTTAGTGCTGACGCATTTAAAAGTGGTATTGTACAGTATCTC

D05-SEQ1.ab1(1>819) →

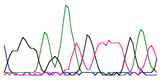

GGGAGATGTTTGATG

A11-SEQ3R.ab1(1>814) ←

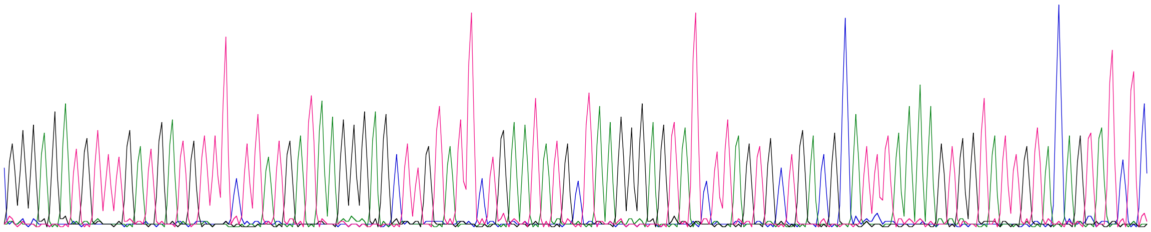

GGGAGATGTTTGATGATGTTTCTTATGATAAGGGAGCTTGTATTCTGAATATGCTAAGGGAGTATCTTAGTGCTGACGCATTTAAAAGTGGTATTGTACAGTATCTC

B01-SEQ2.ab1(1>819) →

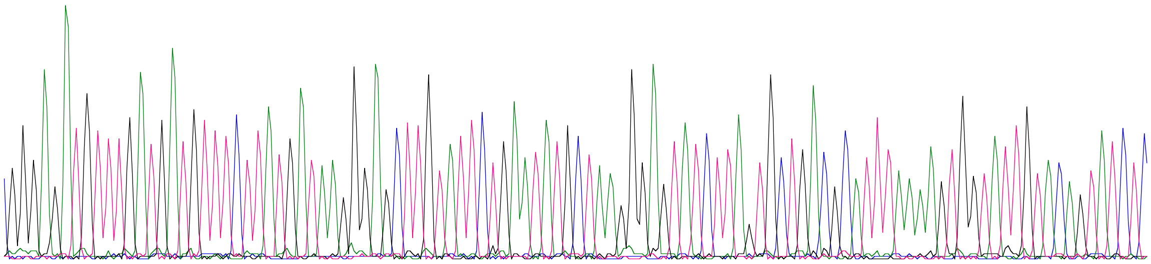

GGGAGATGTTTGATGATGTTTCTTATGATAAGGGAGCTTGTATTCTGAATATGCTAAGGGAGTATCTTAGTGCTGACGCATTTAAAAGTGGTATTGTACAGTATCTC

1,720 1,730 1,740 1,750 1,760 1,770 1,780 1,790 1,800 1,810

CAGAAGCATAGCTATAAAAATACAAAAAACGAGGACCTGTGGGATAGTATGGCAAGTATTTGCCCTACAGATGGTGTAAAAGGGATGGATGGCTTTTGCTCTAGAAG

U3823DH140-8.seq(1>2878) → CAGAAGCATAGCTATAAAAATACAAAAAACGAGGACCTGTGGGATAGTATGGCAAGTATTTGCCCTACAGATGGTGTAAAAGGGATGGATGGCTTTTGCTCTAGAAG

A11-SEQ3R.ab1(1>814) ←

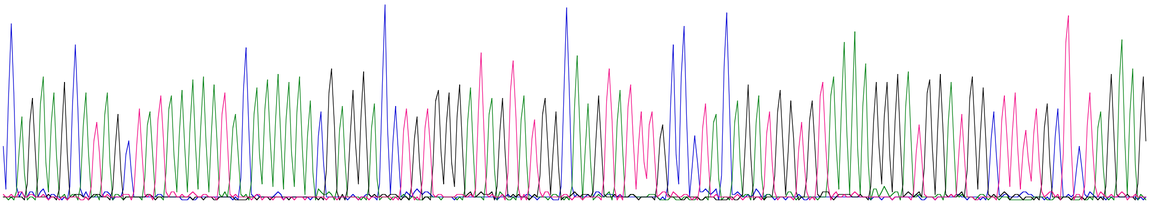

CAGAAGCATAGCTATAAAAATACAAAAAACGAGGACCTGTGGGATAGTATGGCAAGTATTTGCCCTACAGATGGTGTAAAAGGGATGGATGGCTTTTGCTCTAGAAG

B01-SEQ2.ab1(1>819) →

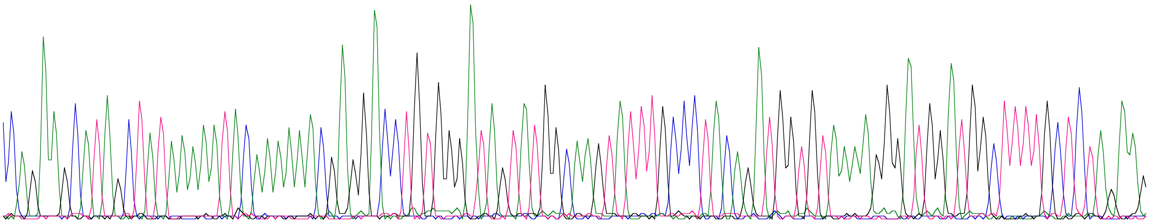

CAGAAGCATAGCTATAAAAATACAAAAAACGAGGACCTGTGGGATAGTATGGCAAGTATTTGCCCTACAGATGGTGTAAAAGGGATGGATGGCTTTTGCTCTAGAAG

B07-SEQ4RN.ab1(1>815) ←

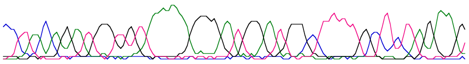

CCTACAGATGGTGTAAAAGGGATGGATGGCTTTTGCTCTAGAAG

E08-SEQ4RN.ab1(1>785) ←

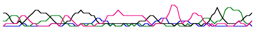

GATGGATGGCTTTTGCTCTAGAAG

D03-SEQ3.ab1(1>802) →

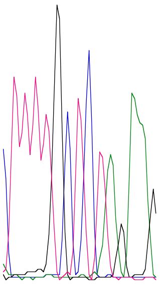

CTTTTGCTCTAGAAG

1,820 1,830 1,840 1,850 1,860 1,870 1,880 1,890 1,900 1,910 1,920

TCAACATTCATCTTCATCCTCACATTGGCATCAGGAAGGGGTGGATGTGAAAACCATGATGAACACTTGGACACTGCAGAAGGGTTTTCCCCTAATAACCATCACAG

U3823DH140-8.seq(1>2878) → TCAACATTCATCTTCATCCTCACATTGGCATCAGGAAGGGGTGGATGTGAAAACCATGATGAACACTTGGACACTGCAGAAGGGTTTTCCCCTAATAACCATCACAG

A11-SEQ3R.ab1(1>814) ←

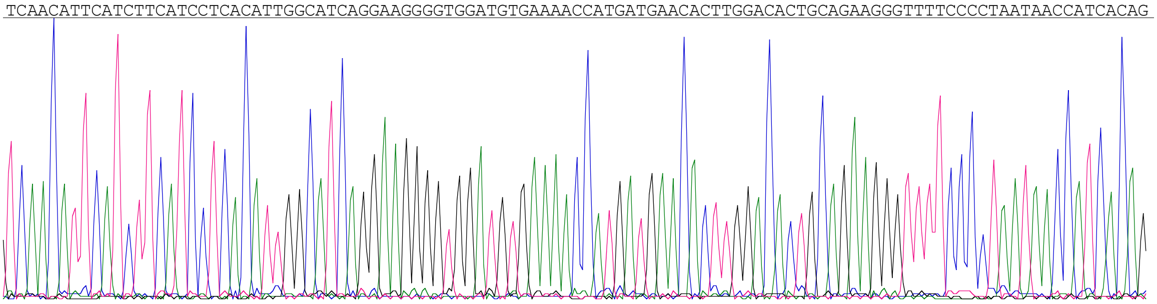

TCAACATTCATCTTCATCCTCACATTGGCATCAGGAAGGGGTGGATGTGAAAACCATGATGAACACTTGGACACTGCAGAAGGGTTTTCCCCTAATAACCATCACAG

1,820 1,830 1,840 1,850 1,860 1,870 1,880 1,890 1,900 1,910 1,920

TCAACATTCATCTTCATCCTCACATTGGCATCAGGAAGGGGTGGATGTGAAAACCATGATGAACACTTGGACACTGCAGAAGGGTTTTCCCCTAATAACCATCACAG

B01-SEQ2.ab1(1>819)

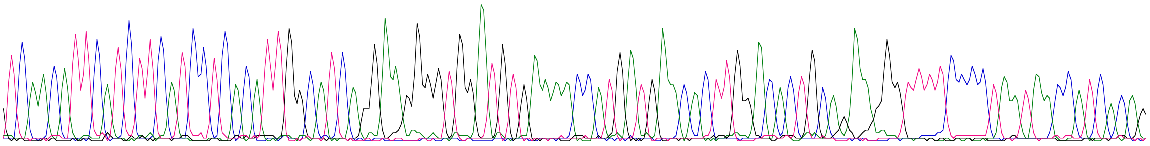

TCAACATTCATCTTCATCCTCACATTGGCATCAGGAAGGGGTGGATGTGAAAACCATGATGAACACTTGGACACTGCAGAAGGGTTTTCCCCTAATAACCATCACAG

B07-SEQ4RN.ab1(1>815)

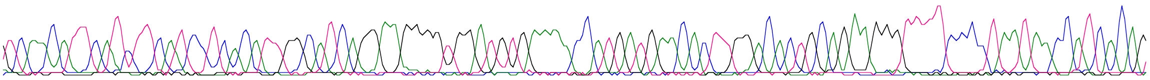

TCAACATTCATCTTCATCCTCACATTGGCATCAGGAAGGGGTGGATGTGAAAACCATGATGAACACTTGGACACTGCAGAAGGGTTTTCCCCTAATAACCATCACAG

E08-SEQ4RN.ab1(1>785)

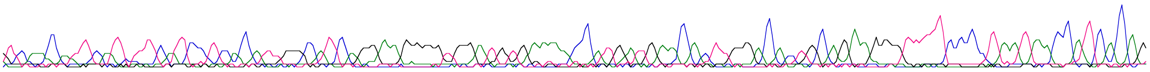

TCAACATTCATCTTCATCCTCACATTGGCATCAGGAAGGGGTGGATGTGAAAACCATGATGAACACTTGGACACTGCAGAAGGGTTTTCCCCTAATAACCATCACAG

D03-SEQ3.ab1(1>802)

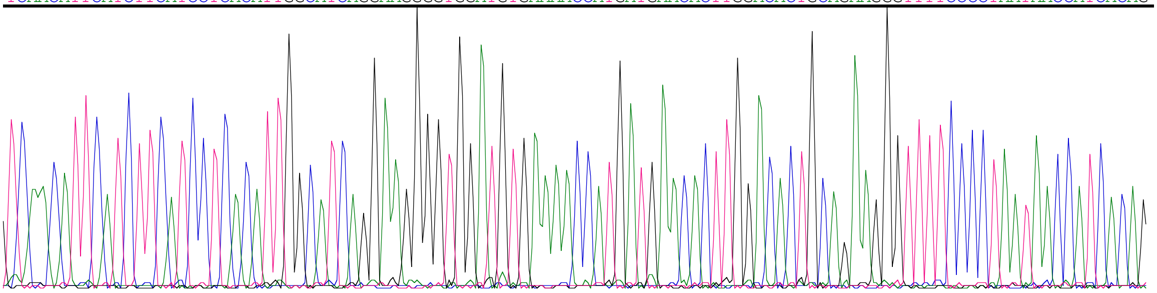

TCAACATTCATCTTCATCCTCACATTGGCATCAGGAAGGGGTGGATGTGAAAACCATGATGAACACTTGGACACTGCAGAAGGGTTTTCCCCTAATAACCATCACAG

A08-SEQ3.ab1(1>808)

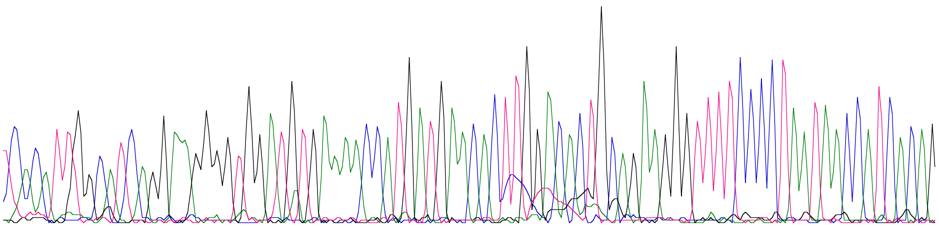

TCaCATTGGCATCAGGAAGGGGTGGATGTGAAAACCATGATGAACACTTGGACACTGCAGAAGGGTTTTCCCCTAATAACCATCACAG

1,930 1,940 1,950 1,960 1,970 1,980 1,990 2,000 2,010 2,020 2,030

TGAGGGGGAGGAATGTACACATGAAGCAAGAGCACTACATGAAGGGCTCTGACGGCGCCCCGGACACTGGGTACCTGTGGCATGTTCCATTGACATTCATCACCAGC

U3823DH140-8.seq(1>2878)

TGAGGGGGAGGAATGTACACATGAAGCAAGAGCACTACATGAAGGGCTCTGACGGCGCCCCGGACACTGGGTACCTGTGGCATGTTCCATTGACATTCATCACCAGC

A11-SEQ3R.ab1(1>814)

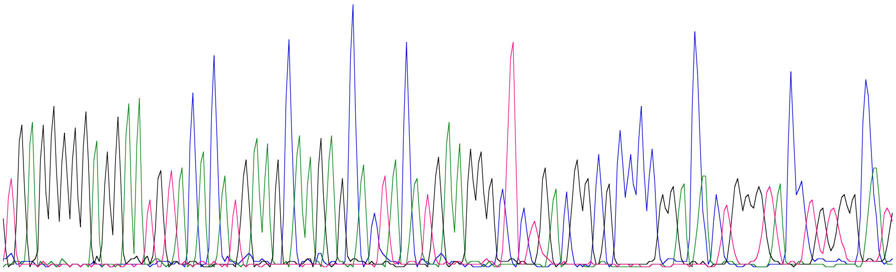

TGAGGGGGaGGAATGTACACATGAAGCAAGAGCACTACATGAAGGGCTCTGACGGCGCCCCGGACACTGGGTACCTGTGGCATG

1,930 1,940 1,950 1,960 1,970 1,980 1,990 2,000 2,010 2,020 2,030

TGAGGGGGAGGAATGTACACATGAAGCAAGAGCACTACATGAAGGGCTCTGACGGCGCCCCGGACACTGGGTACCTGTGGCATGTTCCATTGACATTCATCACCAGC

B01-SEQ2.ab1(1>819)

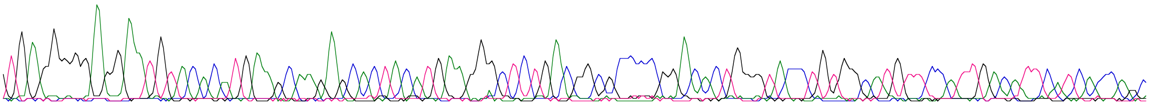

TGAGGGGGAGGAATGTACACATGAAGCAAGAGCACTACATGAAGGGCTCTGACGGCGCCCCGGACACTGGGTACCTGTGGCATGTTCCATTGACATTCATCACCAGC

B07-SEQ4RN.ab1(1>815)

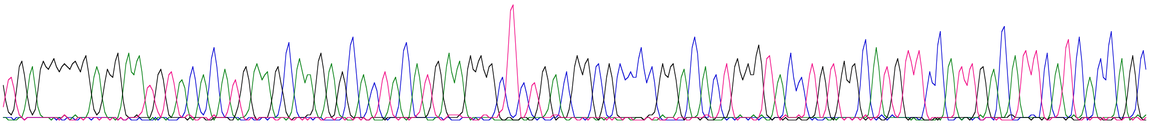

TGAGGGGGAGGAATGTACACATGAAGCAAGAGCACTACATGAAGGGCTCTGACGGCGCCCCGGACACTGGGTACCTGTGGCATGTTCCATTGACATTCATCACCAGC

E08-SEQ4RN.ab1(1>785)

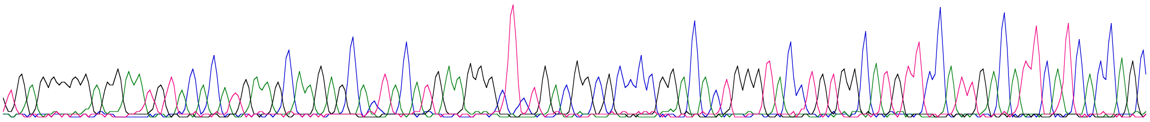

TGAGGGGGAGGAATGTACACATGAAGCAAGAGCACTACATGAAGGGCTCTGACGGCGCCCCGGACACTGGGTACCTGTGGCATGTTCCATTGACATTCATCACCAGC

D03-SEQ3.ab1(1>802)

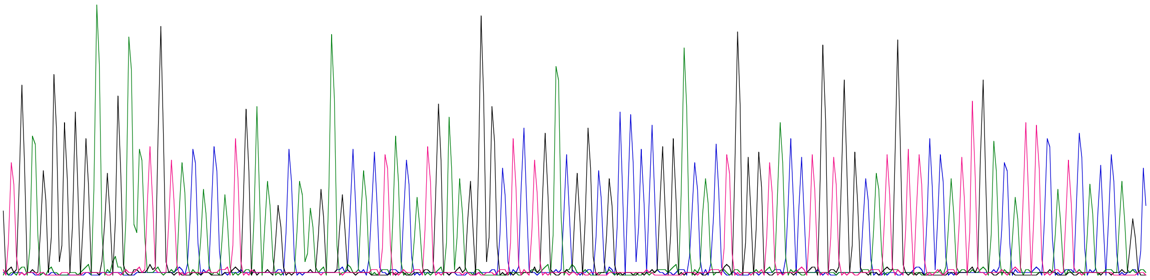

TGAGGGGGAGGAATGTACACATGAAGCAAGAGCACTACATGAAGGGCTCTGACGGCGCCCCGGACACTGGGTACCTGTGGCATGTTCCATTGACATTCATCACCAGC

A08-SEQ3.ab1(1>808)

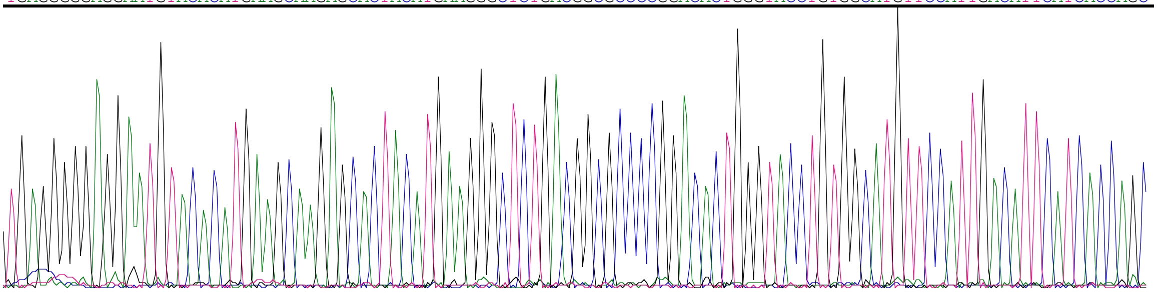

TGAGGGGGAGGAATGTACACATGAAGCAAGAGCACTACATGAAGGGCTCTGACGGCGCCCCGGACACTGGGTACCTGTGGCATGTTCCATTGACATTCATCACCAGC

2,040 2,050 2,060 2,070 2,080 2,090 2,100 2,110 2,120 2,130 2,140

AAATCCGACATGGTCCATCGATTTTTGCTAAAAACAAAAACAGATGTGCTCATCCTCCCAGAAGAGGTGGAATGGATCAAATTTAATGTGGGCATGAATGGCTATTA

U3823DH140-8.seq(1>2878)
B01-SEQ2.ab1(1>819)

AAATCCGACATGGTCCATCGATTTTTGCTAAAAACAAAAACAGATGTGCTCATCCTCCCAGAAGAGGTGGAATGGATCAAATTTAATGTGGGCATGAATGGCTATTA

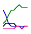

AAA

2,040 2,050 2,060 2,070 2,080 2,090 2,100 2,110 2,120 2,130 2,140

AAATCCGACATGGTCCATCGATTTTTGCTAAAAACAAAAACAGATGTGCTCATCCTCCCAGAAGAGGTGGAATGGATCAAATTTAATGTGGGCATGAATGGCTATTA

B07-SEQ4RN.ab1(1>815) ←

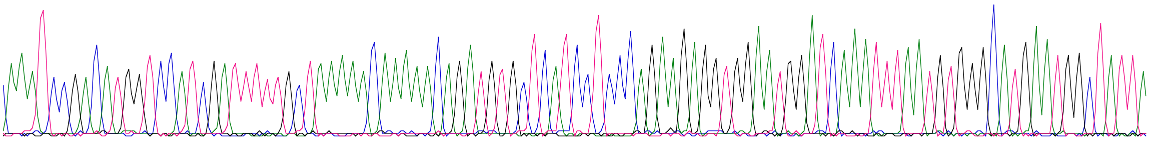

AAATCCGACATGGTCCATCGATTTTTGCTAAAAACAAAAACAGATGTGCTCATCCTCCCAGAAGAGGTGGAATGGATCAAATTTAATGTGGGCATGAATGGCTATTA

E08-SEQ4RN.ab1(1>785) ←

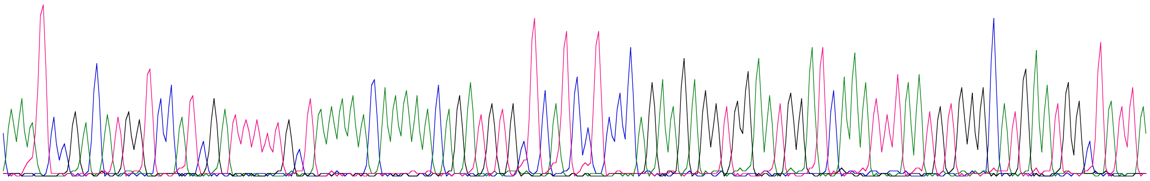

AAATCCGACATGGTCCATCGATTTTTGCTAAAAACAAAAACAGATGTGCTCATCCTCCCAGAAGAGGTGGAATGGATCAAATTTAATGTGGGCATGAATGGCTATTA

D03-SEQ3.ab1(1>802) →

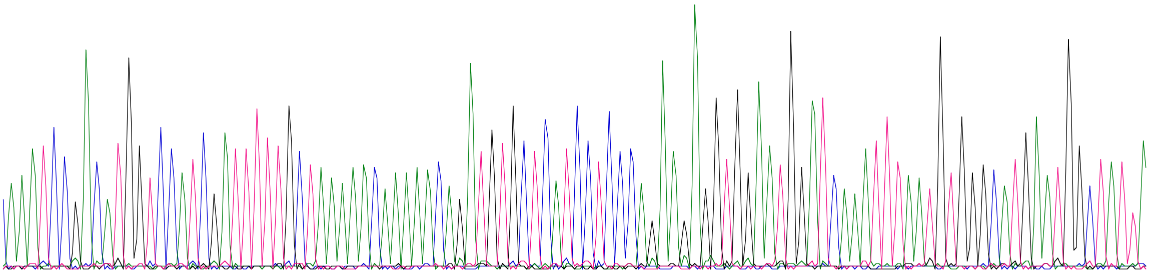

AAATCCGACATGGTCCATCGATTTTTGCTAAAAACAAAAACAGATGTGCTCATCCTCCCAGAAGAGGTGGAATGGATCAAATTTAATGTGGGCATGAATGGCTATTA

A08-SEQ3.ab1(1>808) →

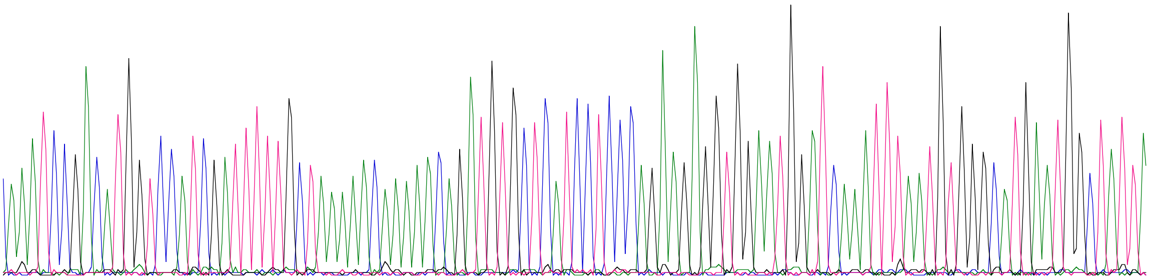

AAATCCGACATGGTCCATCGATTTTTGCTAAAAACAAAAACAGATGTGCTCATCCTCCCAGAAGAGGTGGAATGGATCAAATTTAATGTGGGCATGAATGGCTATTA

2,150 2,160 2,170 2,180 2,190 2,200 2,210 2,220 2,230 2,240

CATTGTGCATTACGAGGATGATGGATGGGACTCTTTGACTGGCCTTTTAAAAGGAACACACACAGCAGTCAGCAGTAATGATCGGGCGAGTCTCATTAACAATGCAT

U3823DH140-8.seq(1>2878) → CATTGTGCATTACGAGGATGATGGATGGGACTCTTTGACTGGCCTTTTAAAAGGAACACACACAGCAGTCAGCAGTAATGATCGGGCGAGTCTCATTAACAATGCAT

B07-SEQ4RN.ab1(1>815) ←

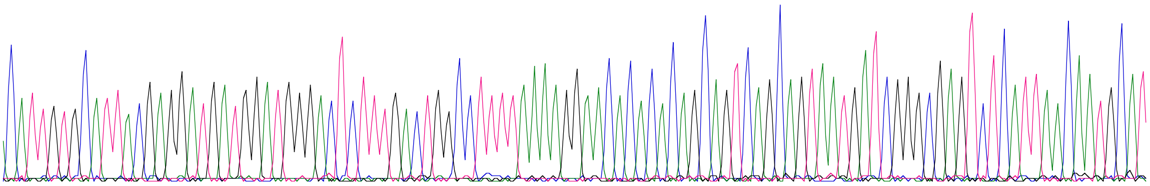

CATTGTGCATTACGAGGATGATGGATGGGACTCTTTGACTGGCCTTTTAAAAGGAACACACACAGCAGTCAGCAGTAATGATCGGGCGAGTCTCATTAACAATGCAT

E08-SEQ4RN.ab1(1>785) ←

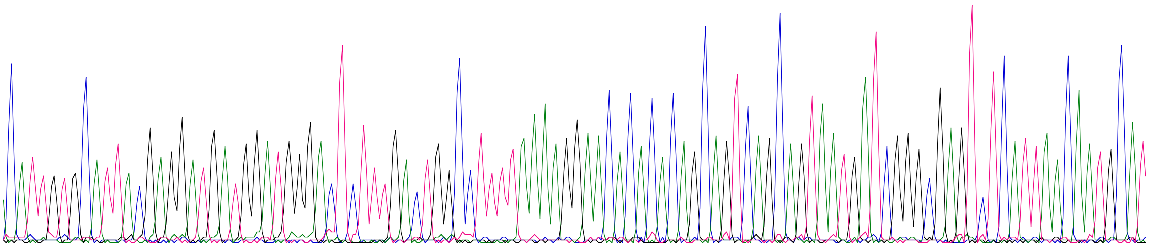

CATTGTGCATTACGAGGATGATGGATGGGACTCTTTGACTGGCCTTTTAAAAGGAACACACACAGCAGTCAGCAGTAATGATCGGGCGAGTCTCATTAACAATGCAT

2,150 2,160 2,170 2,180 2,190 2,200 2,210 2,220 2,230 2,240

CATTGTGCATTACGAGGATGATGGATGGGACTCTTTGACTGGCCTTTTAAAAGGAACACACACAGCAGTCAGCAGTAATGATCGGGCGAGTCTCATTAACAATGCAT

D03-SEQ3.ab1(1>802)

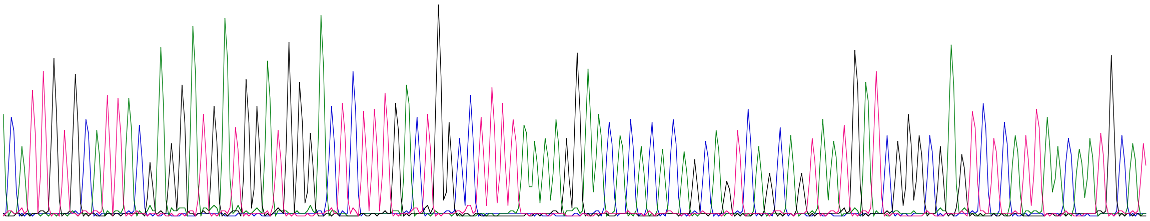

CATTGTGCATTACGAGGATGATGGATGGGACTCTTTGACTGGCCTTTTAAAAGGAACACACACAGCAGTCAGCAGTAATGATCGGGCGAGTCTCATTAACAATGCAT

A08-SEQ3.ab1(1>808)

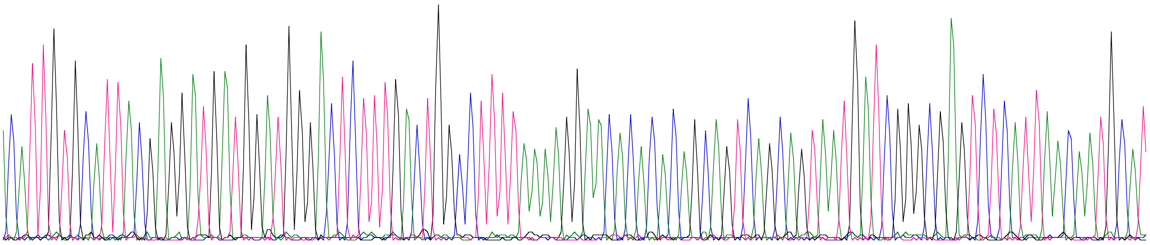

CATTGTGCATTACGAGGATGATGGATGGGACTCTTTGACTGGCCTTTTAAAAGGAACACACACAGCAGTCAGCAGTAATGATCGGGCGAGTCTCATTAACAATGCAT

2,250 2,260 2,270 2,280 2,290 2,300 2,310 2,320 2,330 2,340 2,350

TTCAGCTCGTCAGCATTGGGAAGCTGTCCATTGAAAAGGCCTTGGATTTATCCCTGTACTTGAAACATGAAACTGAAATTATGCCCGTGTTTCAAGGTTTGAATGAG

U3823DH140-8.seq(1>2878) TTCAGCTCGTCAGCATTGGGAAGCTGTCCATTGAAAAGGCCTTGGATTTATCCCTGTACTTGAAACATGAAACTGAAATTATGCCCGTGTTTCAAGGTTTGAATGAG

B07-SEQ4RN.ab1(1>815)

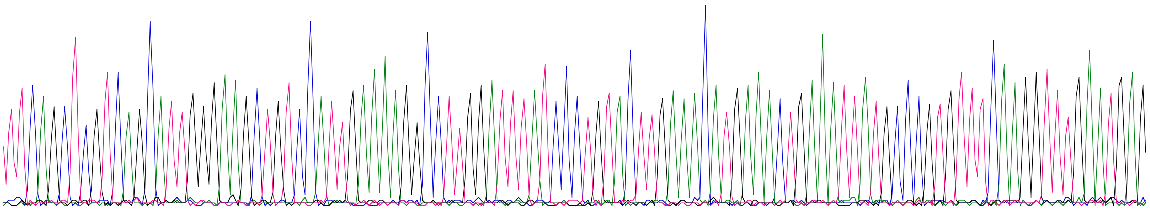

TTCAGCTCGTCAGCATTGGGAAGCTGTCCATTGAAAAGGCCTTGGATTTATCCCTGTACTTGAAACATGAAACTGAAATTATGCCCGTGTTTCAAGGTTTGAATGAG

E08-SEQ4RN.ab1(1>785)

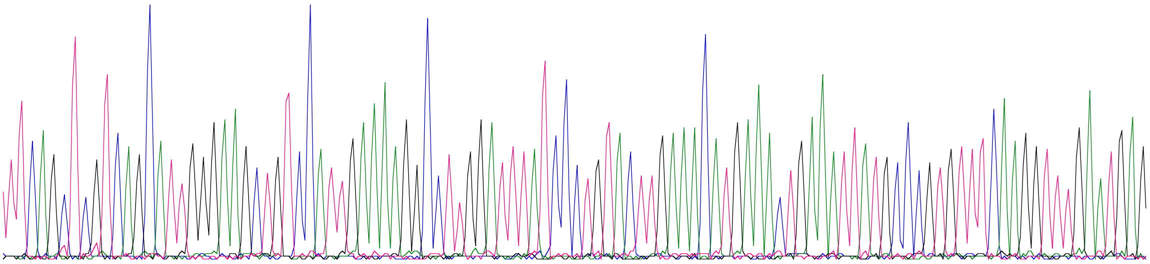

TTCAGCTCGTCAGCATTGGGAAGCTGTCCATTGAAAAGGCCTTGGATTTATCCCTGTACTTGAAACATGAAACTGAAATTATGCCCGTGTTTCAAGGTTTGAATGAG

D03-SEQ3.ab1(1>802)

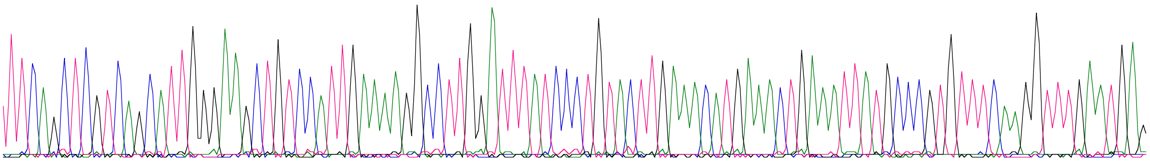

TTCAGCTCGTCAGCATTGGGAAGCTGTCCATTGAAAAGGCCTTGGATTTATCCCTGTACTTGAAACATGAAACTGAAATTATGCCCGTGTTTCAAGGTTTGAATGAG

A08-SEQ3.ab1(1>808)

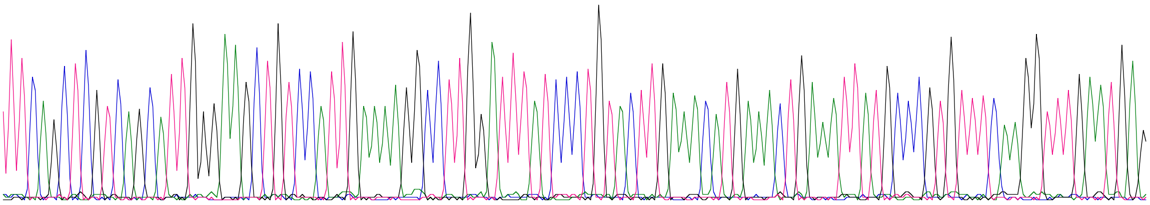

TTCAGCTCGTCAGCATTGGGAAGCTGTCCATTGAAAAGGCCTTGGATTTATCCCTGTACTTGAAACATGAAACTGAAATTATGCCCGTGTTTCAAGGTTTGAATGAG

2,250 2,260 2,270 2,280 2,290 2,300 2,310 2,320 2,330 2,340 2,350

TTCAGCTCGTCAGCATTGGGAAGCTGTCCATTGAAAAGGCCTTGGATTTATCCCTGTACTTGAAACATGAAACTGAAATTATGCCCGTGTTTCAAGGTTTGAATGAG

D08-SEQ4.ab1(1>819)

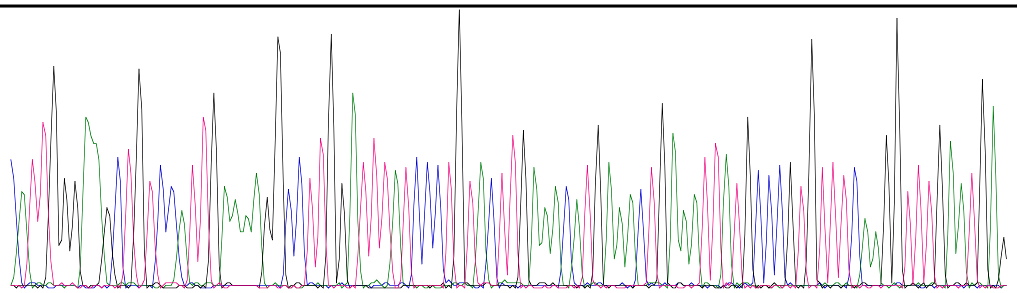

CATTGGGAAGCTGTCCATTGAAAAGGCCTTGGATTTATCCCTGTACTTGAAACATGAAACTGAAATTATGCCCGTGTTTCAAGGTTTGAATGAG

2,360 2,370 2,380 2,390 2,400 2,410 2,420 2,430 2,440 2,450 2,460

CTGATTCCTATGTATAAGTTAATGGAGAAAAGAGATATGAATGAAGTGGAAACTCAATTCAAGGCCTTCCTCATCAGGCTGCTAAGGGACCTCATTGATAAGCAGAC

U3823DH140-8.seq(1>2878)

CTGATTCCTATGTATAAGTTAATGGAGAAAAGAGATATGAATGAAGTGGAAACTCAATTCAAGGCCTTCCTCATCAGGCTGCTAAGGGACCTCATTGATAAGCAGAC

B07-SEQ4RN.ab1(1>815)

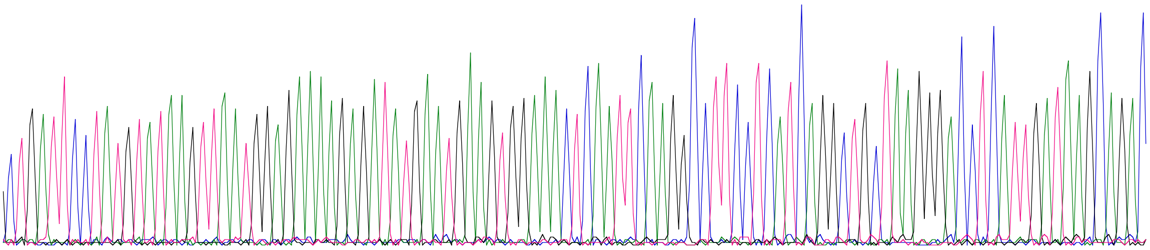

CTGATTCCTATGTATAAGTTAATGGAGAAAAGAGATATGAATGAAGTGGAAACTCAATTCAAGGCCTTCCTCATCAGGCTGCTAAGGGACCTCATTGATAAGCAGAC

E08-SEQ4RN.ab1(1>785)

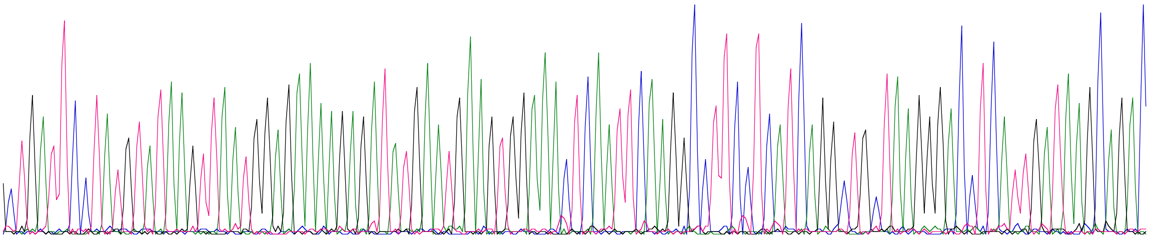

CTGATTCCTATGTATAAGTTAATGGAGAAAAGAGATATGAATGAAGTGGAAACTCAATTCAAGGCCTTCCTCATCAGGCTGCTAAGGGACCTCATTGATAAGCAGAC

D03-SEQ3.ab1(1>802)

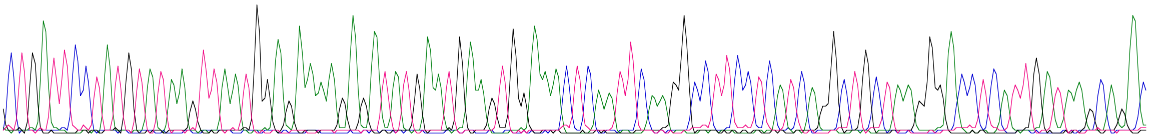

CTGATTCCTATGTATAAGTTAATGGAGAAAAGAGATATGAATGAAGTGGAAACTCAATTCAAGGCCTTCCTCATCAGGCTGCTAAGGGACCTCATTGATAAGCAGAC

A08-SEQ3.ab1(1>808)

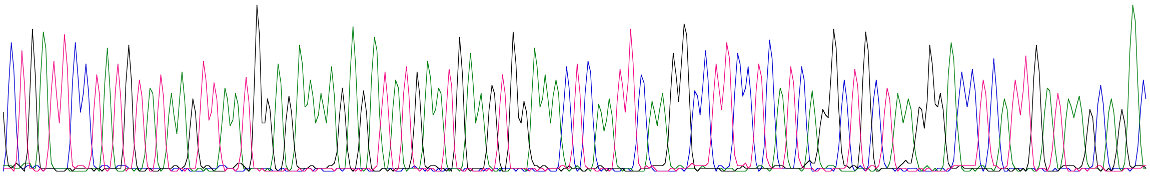

CTGATTCCTATGTATAAGTTAATGGAGAAAAGAGATATGAATGAAGTGGAAACTCAATTCAAGGCCTTCCTCATCAGGCTGCTAAGGGACCTCATTGATAAGCAGAC

D08-SEQ4.ab1(1>819)

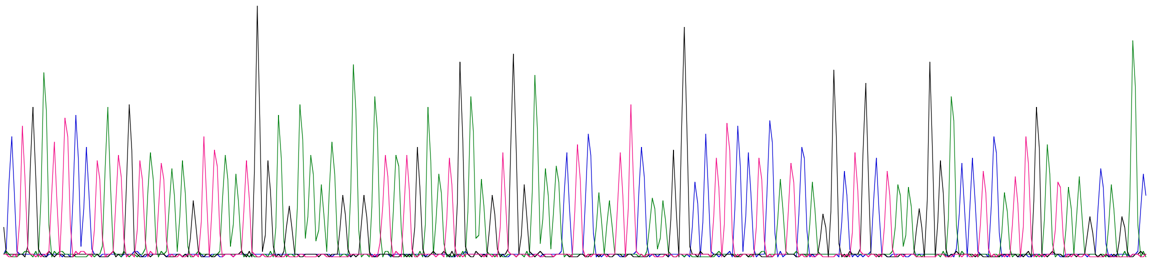

CTGATTCCTATGTATAAGTTAATGGAGAAAAGAGATATGAATGAAGTGGAAACTCAATTCAAGGCCTTCCTCATCAGGCTGCTAAGGGACCTCATTGATAAGCAGAC

2,360 2,370 2,380 2,390 2,400 2,410 2,420 2,430 2,440 2,450 2,460

CTGATTCCTATGTATAAGTTAATGGAGAAAAGAGATATGAATGAAGTGGAAACTCAATTCAAGGCCTTCCTCATCAGGCTGCTAAGGGACCTCATTGATAAGCAGAC

D04-PFastBac1-R.ab1(1>824)

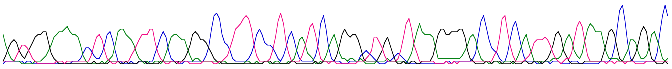

AGTGGAAACTCAATTCAAGGCCTTCCTCATCAGGCTGCTAAGGGACCTCATTGATAAGCAGAC

2,470 2,480 2,490 2,500 2,510 2,520 2,530 2,540 2,550 2,560

ATGGACAGACGAGGGCTCAGTCTCAGAGCGAATGCTGCGGAGTCAACTACTACTCCTCGCCTGTGTGCACAACTATCAGCCGTGCGTACAGAGGGCAGAAGGCTATT

U3823DH140-8.seq(1>2878) ATGGACAGACGAGGGCTCAGTCTCAGAGCGAATGCTGCGGAGTCAACTACTACTCCTCGCCTGTGTGCACAACTATCAGCCGTGCGTACAGAGGGCAGAAGGCTATT

B07-SEQ4RN.ab1(1>815)

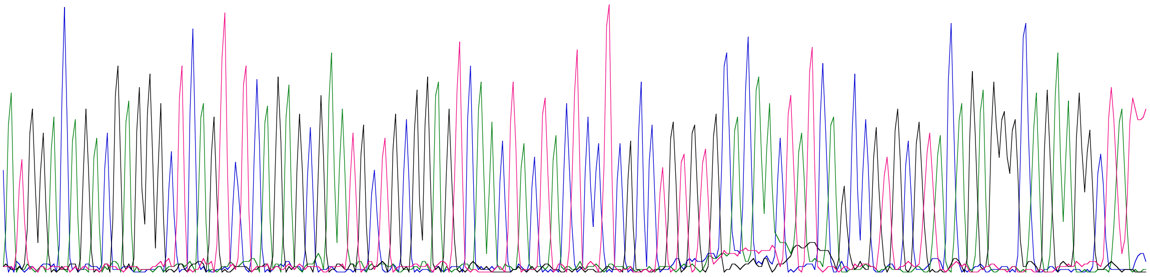

ATGGACAGACGAGGGCTCAGTCTCAGAGCGAATGCTGCGGAGTCAACTACTACTCCTCGCCTGTGTGCACAACTATCAGCCGTGCGTACAGAGGGCAGAAGGCTATt

E08-SEQ4RN.ab1(1>785)

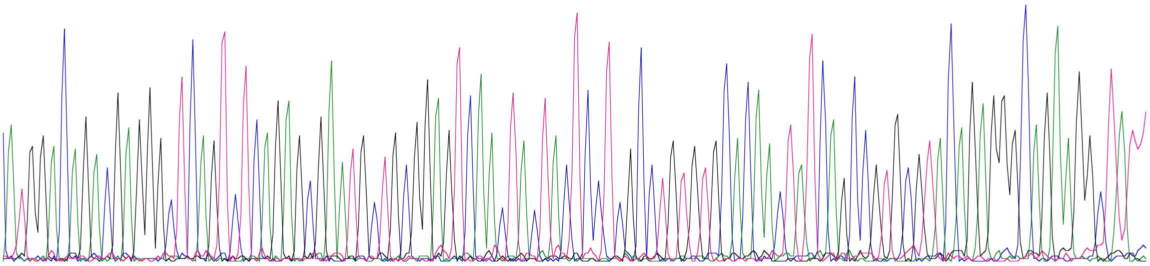

ATGGACAGACGAGGGCTCAGTCTCAGAGCGAATGCTGCGGAGTCAACTACTACTCCTCGCCTGTGTGCACAACTATCAGCCGTGCGTACAGAGGGCAGAAGGCTATt

D03-SEQ3.ab1(1>802)

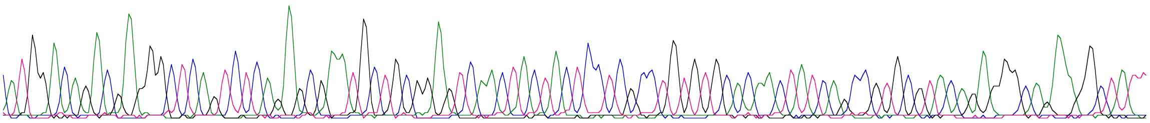

ATGGACAGACGAGGGCTCAGTCTCAGAGCGAATGCTGCGGAGTCAACTACTACTCCTCGCCTGTGTGCACAACTATCAGCCGTGCGTACAGAGGGCAGAAGGCTATT

A08-SEQ3.ab1(1>808)

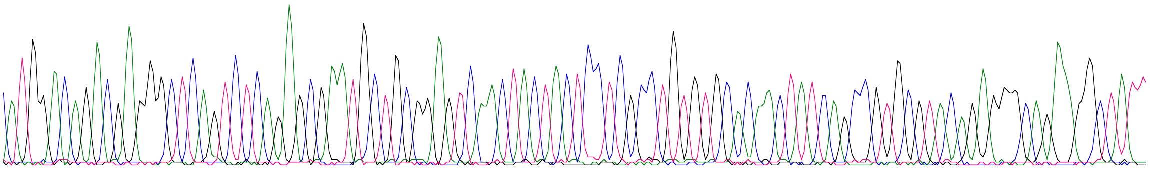

ATGGACAGACGAGGGCTCAGTCTCAGAGCGAATGCTGCGGAGTCAACTACTACTCCTCGCCTGTGTGCACAACTATCAGCCGTGCGTACAGAGGGCAGAAGGCTATT

D08-SEQ4.ab1(1>819)

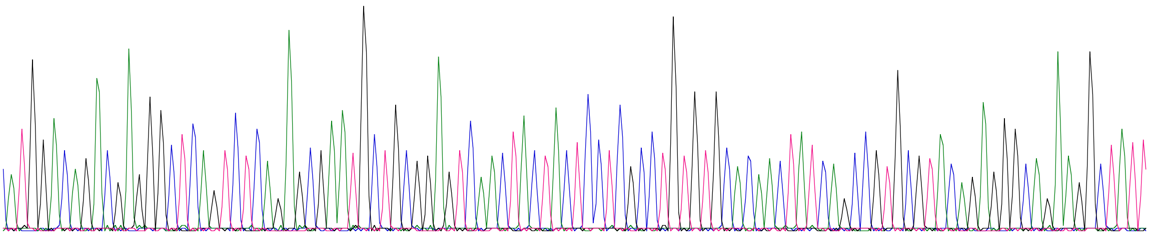

ATGGACAGACGAGGGCTCAGTCTCAGAGCGAATGCTGCGGAGTCAACTACTACTCCTCGCCTGTGTGCACAACTATCAGCCGTGCGTACAGAGGGCAGAAGGCTATT

2,470 2,480 2,490 2,500 2,510 2,520 2,530 2,540 2,550 2,560

ATGGACAGACGAGGGCTCAGTCTCAGAGCGAATGCTGCGGAGTCAACTACTACTCCTCGCCTGTGTGCACAACTATCAGCCGTGCGTACAGAGGGCAGAAGGCTATT

D04-PFastBac1-R.ab1(1>824)

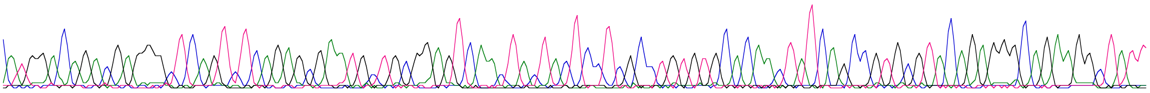

ATGGACAGACGAGGGCTCAGTCTCAGAGCGAATGCTGCGGAGTCAACTACTACTCCTCGCCTGTGTGCACAACTATCAGCCGTGCGTACAGAGGGCAGAAGGCTATT

2,570 2,580 2,590 2,600 2,610 2,620 2,630 2,640 2,650 2,660 2,670

TCAGAAAGTGGAAGGAATCCAATGGAAACTTGAGCCTGCCTGTCGACGTGACCTTGGCAGTGTTTGCTGTGGGGGCCCAGAGCACAGAAGGCTGGGATTTTCTTTAT

U3823DH140-8.seq(1>2878) TCAGAAAGTGGAAGGAATCCAATGGAAACTTGAGCCTGCCTGTCGACGTGACCTTGGCAGTGTTTGCTGTGGGGGCCCAGAGCACAGAAGGCTGGGATTTTCTTTAT

B07-SEQ4RN.ab1(1>815)

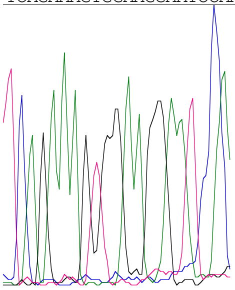

TCAGAAAGTGGAAGGAATCCAA

E08-SEQ4RN.ab1(1>785)

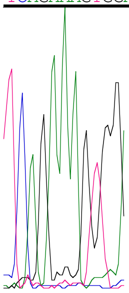

TCAGAAAGTGGA

D03-SEQ3.ab1(1>802)

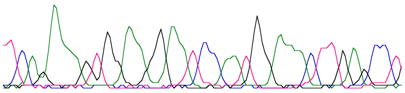

TCAGAAAGTGGAAGGAATCCAATGGAAACTTGAGCCTG

A08-SEQ3.ab1(1>808)

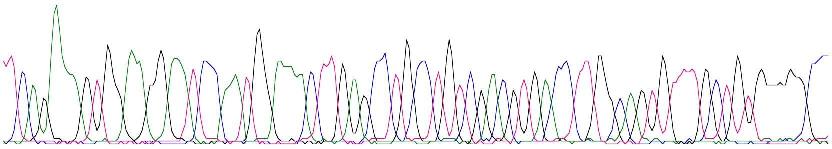

TCAGAAAGTGGAAGGAATCCAATGGAAACTTGAGCCTGCCTGTCGACGTGACCTTGGCAGTGTTTGCTGTGGGGGCCC

D08-SEQ4.ab1(1>819)

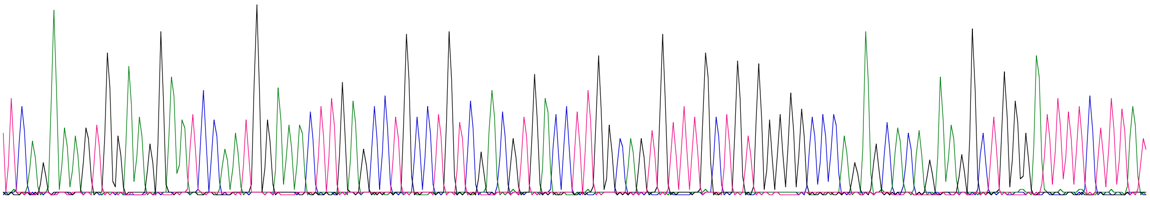

TCAGAAAGTGGAAGGAATCCAATGGAAACTTGAGCCTGCCTGTCGACGTGACCTTGGCAGTGTTTGCTGTGGGGGCCCAGAGCACAGAAGGCTGGGATTTTCTTTAT

2,570 2,580 2,590 2,600 2,610 2,620 2,630 2,640 2,650 2,660 2,670

TCAGAAAGTGGAAGGAATCCAATGGAAACTTGAGCCTGCCTGTCGACGTGACCTTGGCAGTGTTTGCTGTGGGGGCCCAGAGCACAGAAGGCTGGGATTTTCTTTAT

D04-PFastBac1-R.ab1(1>824)

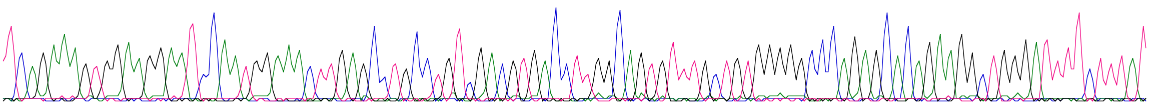

TCAGAAAGTGGAAGGAATCCAATGGAAACTTGAGCCTGCCTGTCGACGTGACCTTGGCAGTGTTTGCTGTGGGGGCCCAGAGCACAGAAGGCTGGGATTTTCTTTAT

2,680 2,690 2,700 2,710 2,720 2,730 2,740 2,750 2,760 2,770 2,780

AGTAAATATCAGTTTTCTTTGTCCAGTACTGAGAAAAGCCAAATTGAATTTGCCCTCTGCAGAACCCAAAATAAGGAAAAGCTTCAATGGCTACTAGATGAAAGCTT

U3823DH140-8.seq(1>2878)
D08-SEQ4.ab1(1>819)

AGTAAATATCAGTTTTCTTTGTCCAGTACTGAGAAAAGCCAAATTGAATTTGCCCTCTGCAGAACCCAAAATAAGGAAAAGCTTCAATGGCTACTAGATGAAAGCTT

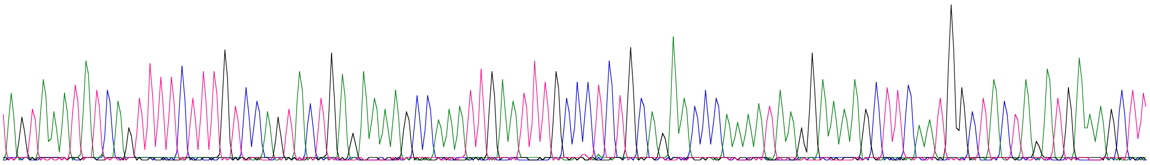

AGTAAATATCAGTTTTCTTTGTCCAGTACTGAGAAAAGCCAAATTGAATTTGCCCTCTGCAGAACCCAAAATAAGGAAAAGCTTCAATGGCTACTAGATGAAAGCTT

D04-PFastBac1-R.ab1(1>824)

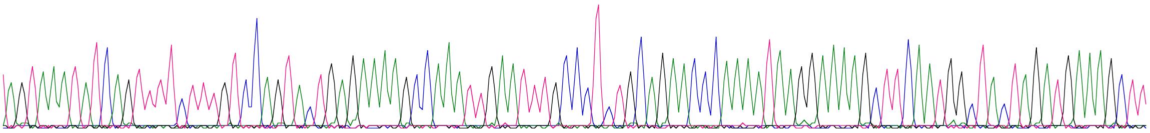

AGTAAATATCAGTTTTCTTTGTCCAGTACTGAGAAAAGCCAAATTGAATTTGCCCTCTGCAGAACCCAAAATAAGGAAAAGCTTCAATGGCTACTAGATGAAAGCTT

2,790 2,800 2,810 2,820 2,830 2,840 2,850 2,860 2,870 2,880

TAAGGGAGATAAAATAAAAACTCAGGAGTTTCCACAAATTCTTACACTCATTGGCAGGAACCCAGTAGGATACCCACTGGCCTGGCAATTTCTGAGGAAAAACTGGA

U3823DH140-8.seq(1>2878)
D08-SEQ4.ab1(1>819)

TAAGGGAGATAAAATAAAAACTCAGGAGTTTCCACAAATTCTTACACTCATTGGCAGGAACCCAGTAGGATACCCACTGGCCTGGCAATTTCTGAGGAAAAACTGGA

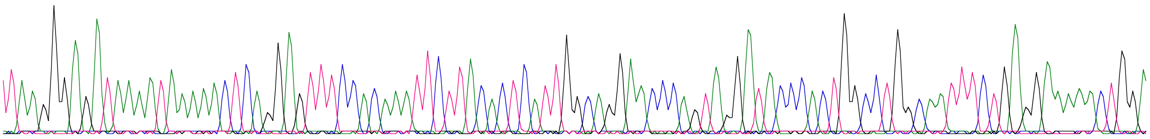

TAAGGGAGATAAAATAAAAACTCAGGAGTTTCCACAAATTCTTACACTCATTGGCAGGAACCCAGTAGGATACCCACTGGCCTGGCAATTTCTGAGGAAAAACTGGA

D04-PFastBac1-R.ab1(1>824)

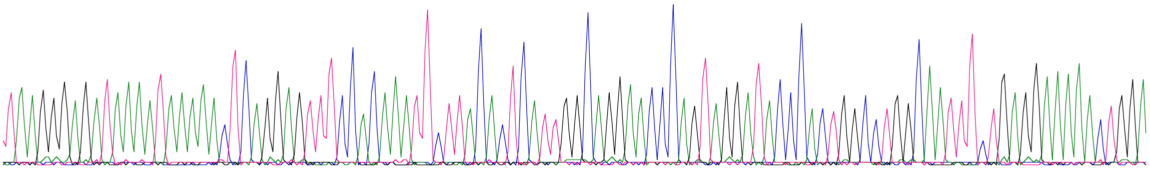

TAAGGGAGATAAAATAAAAACTCAGGAGTTTCCACAAATTCTTACACTCATTGGCAGGAACCCAGTAGGATACCCACTGGCCTGGCAATTTCTGAGGAAAAACTGGA

2,890 2,900 2,910 2,920 2,930 2,940 2,950 2,960 2,970 2,980 2,990

ACAAACTTGTACAAAAGTTTGAACTTGGCTCATCTTCCATAGCCCACATGGTAATGGGTACAACAAATCAATTCTCCACAAGAACACGGCTTGAAGAGGTAAAAGGA

U3823DH140-8.seq(1>2878) → ACAAACTTGTACAAAAGTTTGAACTTGGCTCATCTTCCATAGCCCACATGGTAATGGGTACAACAAATCAATTCTCCACAAGAACACGGCTTGAAGAGGTAAAAGGA

D08-SEQ4.ab1(1>819) →

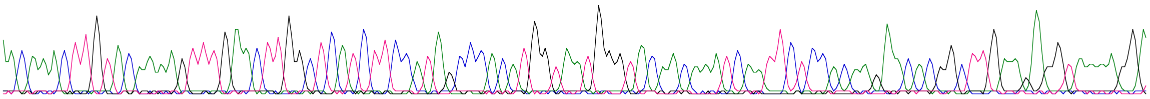

ACAAACTTGTACAAAAGTTTGAACTTGGCTCATCTTCCATAGCCCACATGGTAATGGGTACAACAAATCAATTCTCCACAAGAACACGGCTTGAAGAGGTAAAAGGA

D04-PFastBac1-R.ab1(1>824) ←

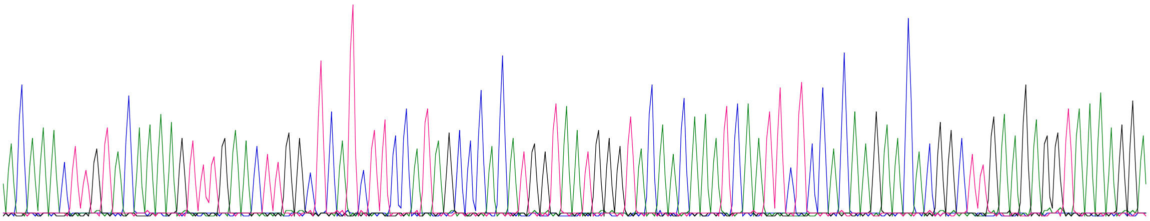

ACAAACTTGTACAAAAGTTTGAACTTGGCTCATCTTCCATAGCCCACATGGTAATGGGTACAACAAATCAATTCTCCACAAGAACACGGCTTGAAGAgGTAAAAGGA

A03-SEQ5.ab1(1>383) →

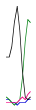

GGA

3,000 3,010 3,020 3,030 3,040 3,050 3,060 3,070 3,080 3,090 3,100

TTCTTCAGCTCTTTGAAAGAAAATGGTTCTCAGCTCCGTTGTGTCCAACAGACAATTGAAACCATTGAAGAAAACATCGGTTGGATGGATAAGAATTTTGATAAAAT

U3823DH140-8.seq(1>2878) → TTCTTCAGCTCTTTGAAAGAAAATGGTTCTCAGCTCCGTTGTGTCCAACAGACAATTGAAACCATTGAAGAAAACATCGGTTGGATGGATAAGAATTTTGATAAAAT

D08-SEQ4.ab1(1>819) →

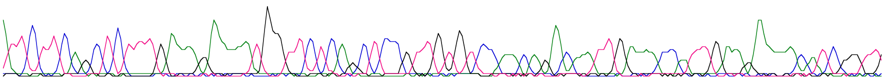

TTCTTCAGCTCTTTGAAAGAAAATGGTTCTCAGCTCCGTTGTGTCCAACAGACAATTGAAACCATTGAAGAAAACATCGGTTG

D04-PFastBac1-R.ab1(1>824) ←

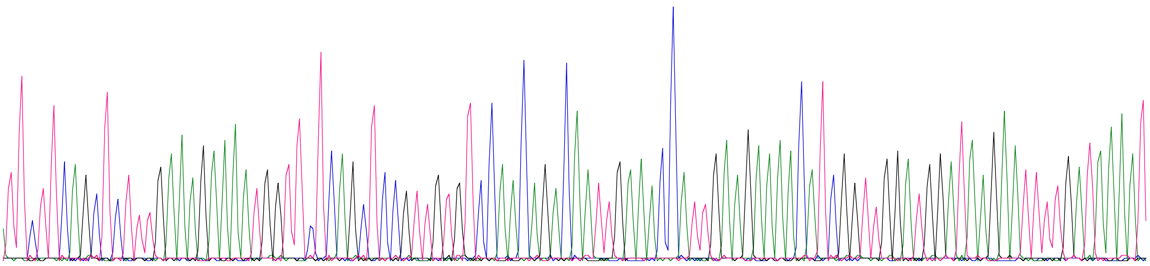

TTCTTCAGCTCTTTGAAAGAAAATGGTTcTCAGCTCCGTTGTGTCCAACAGACAATTGAAACCATTGAAGAAAACATCGGTTGGATGGATAAGAATTTTGATAAAAT

A03-SEQ5.ab1(1>383) →

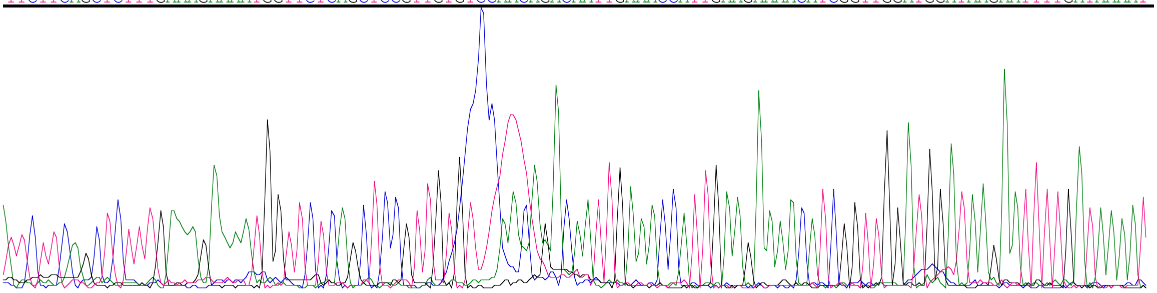

TTCTTCAGCTCTTTGAaAGAAAATGGTTCTCAGCTCCGTTGTGTCCAACAgACAATTGAAACCATTGAAGAAAACATCGGTTGGATGGATAAgAATTTTGATAAAAT

3,110 3,120 3,130 3,140 3,150 3,160 3,170 3,180 3,190 3,200 3,210

CAGAGTGTGGCTGCAAAGTGAAAAGCTTGAACGCATGCATCATCACCATCACCATTAACTCGAGGCATGCGGTACCAAGCTTGTCGAGAAGTACTAGAGGATCATAA

U3823DH140-8.seq(1>2878) → CAGAGTGTGGCTGCAAAGTGAAAAGCTTGAACGCATGCATCATCACCATCACCATTAACTCGAG

D04-PFastBac1-R.ab1(1>824) ←

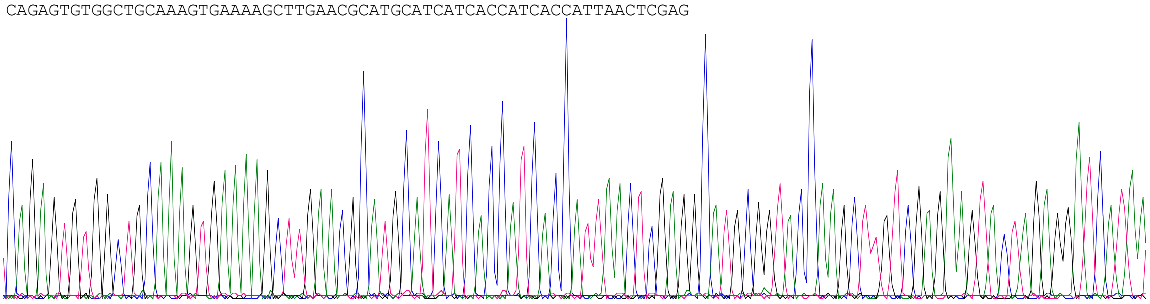

CAGAGTGTGGCTGCAAAGTGAAAAGCTTGAACGCATGCATCATCACCATCACCATTAACTCGAGGCATGCGGTACCAAGCTTGTCGAGAAGTACTAGAGGATCATAA

A03-SEQ5.ab1(1>383) →

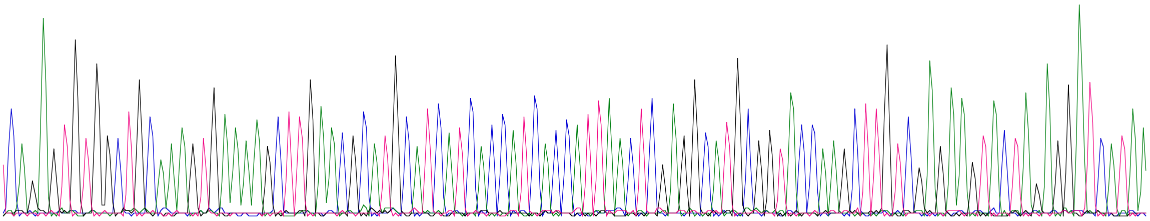

CAGAGTGTGGCTGCAAAGTGAAAAGCTTGAACGCATGCATCATCACCATCACCATTAACTCGAGGCATGCGGTACCAAGCTTGTCGAGAAGTACTAGAGGATCATAA

3,220 3,230 3,240 3,250 3,260 3,270 3,280 3,290 3,300 3,310

TCAGCCATACCACATTTGTAGAGGTTTTACTTGCTTTAAAAAACCTCCCACACCTCCCCCTGAACCTGAAACATAAAATGAATGCAATTGTTGTTGTTAACTTGTTT

D04-PFastBac1-R.ab1(1>824) ←

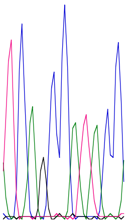

TCAGCCATACCA

A03-SEQ5.ab1(1>383) →

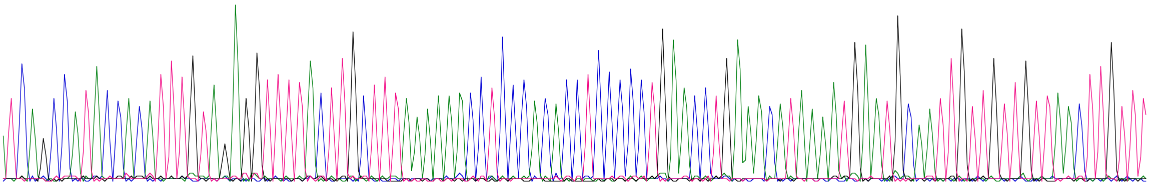

TCAGCCATACCACATTTGTAGAGGTTTTACTTGCTTTAAAAAACCTCCCACACCTCCCCCTGAACCTGAAACATAAAATGAATGCAATTGTTGTTGTTAACTTGTTT

3,320 3,330 3,340 3,350 3,360 3,370

ATTGCAGCTTATAATGGTTACAAATAAAGCAATAGCATCACAAATTTCACAAATAAAGC

A03-SEQ5.ab1(1>383) →

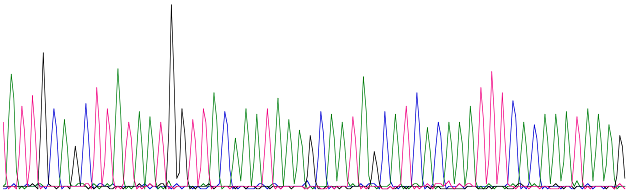

ATTGCAGCTTATAATGGTTACAAATAAAGCAATAGCATCACAAATTTCACAAATAAAGC

10 20 30 40 50 60 70 80

GAGATAATTAAAATGATAACCATCTCGCAAATAAATAAGTATTTTACTGTTTTCGTAACAGTTTTGTAATAAAAAAAACCT

E03-seq4r.ab1(1>808)

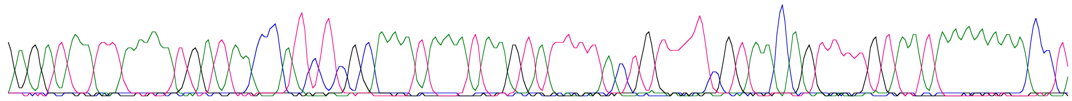

GAGATAATTAAAATGATAACCATCTCGCAAATAAATAAGTATTTTACTGTTTTCGTAACAGTTTTGTAATAAAAAAAACCT

A05-PFastBac-seqF.ab1(1>814)

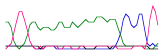

ATAAAAAAACCT

90 100 110 120 130 140 150 160

ATAAATATTCCGGATTATTCATACCGTCCCACCATCGGGCGCGGATCCAACTCCTAAAAAACCGCCACCATGGTGTTTCT

775569-3.seq(1>2877) GGATCCAACTCCTAAAAAACCGCCACCatggtgtttct
E03-seq4r.ab1(1>808)

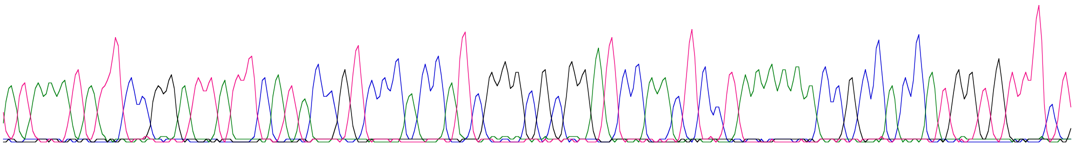

ATAAATATTCCGGATTATTCATACCGTCCCACCATCGGGCGCGGATCCAACTCCTAAAAAACCGCCACCATGGTGTTTCT

A05-PFastBac-seqF.ab1(1>814)

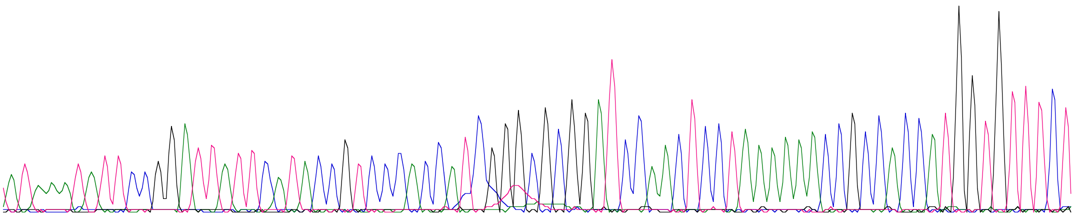

ATAAATATTCCGGATTATTCATACCGTCCCACCATCGGGCGCGGATCCAACTCCTAAAAAACCGCCACCATGGTGTTTCT

170 180 190 200 210 220 230 240

GCCCCTCAAATGGTCCCTTGCAACCATGTCATTTCTACTTTCCTCACTGTTGGCTCTCTTAACTGTGTCCACTCCTTCAT

775569-3.seq(1>2877) gcccctcaaatggtcccttgcaaccatgtcatttctactttcctcactgttggctctcttaactgtgtccactccttcat
E03-seq4r.ab1(1>808)

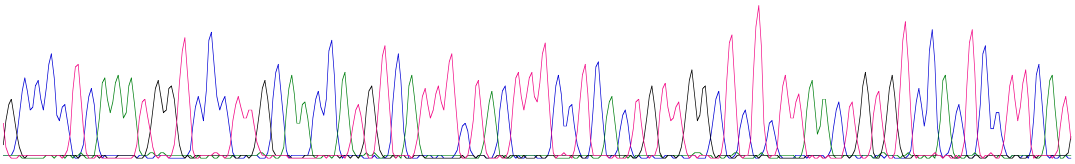

GCCCCTCAAATGGTCCCTTGCAACCATGTCATTTCTACTTTCCTCACTGTTGGCTCTCTTAACTGTGTCCACTCCTTCAT

170 180 190 200 210 220 230 240

GCCCCTCAAATGGTCCCTTGCAACCATGTCATTTCTACTTTCCTCACTGTTGGCTCTCTTAACTGTGTCCACTCCTTCAT

A05-PFastBac-seqF.ab1(1>814) →

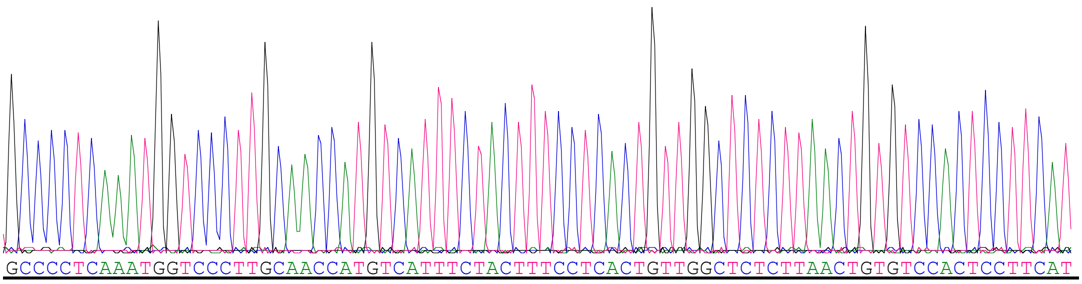

250 260 270 280 290 300 310 320

GGTGTCAGAGCACTGAAGCATCTCCAAAACGTAGTGATGGGACACCATTTCCTTGGAATAAAATACGACTTCCTGAGTAC

775569-3.seq(1>2877) → ggtgtcagagcactgaagcatctccaaaacgtagtgatgggacaccatttccttggaataaaatacgacttcctgagtac
E03-seq4r.ab1(1>808) ←

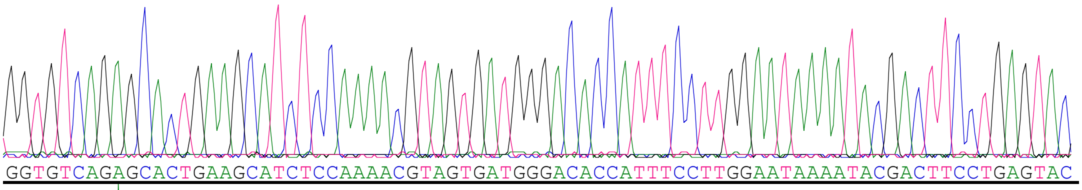

A05-PFastBac-seqF.ab1(1>814) →

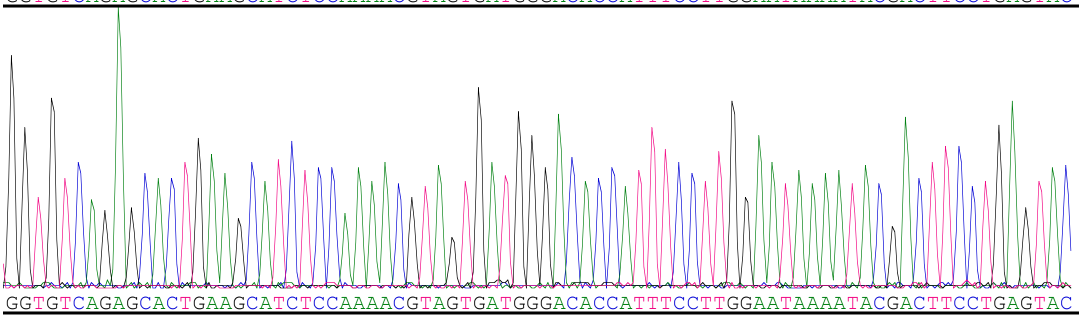

330 340 350 360 370 380 390 400

GTCATCCCAGTTCATTATGATCTCTTGATCCATGCAAACCTTACCACGCTGACCTTCTGGGGAACCACGAAAGTAGAAAT

775569-3.seq(1>2877) → gtcatcccagttcattatgatctcttgatccatgcaaaccttaccacgctgaccttctggggaaccacgaaagtagaaat
E03-seq4r.ab1(1>808) ←

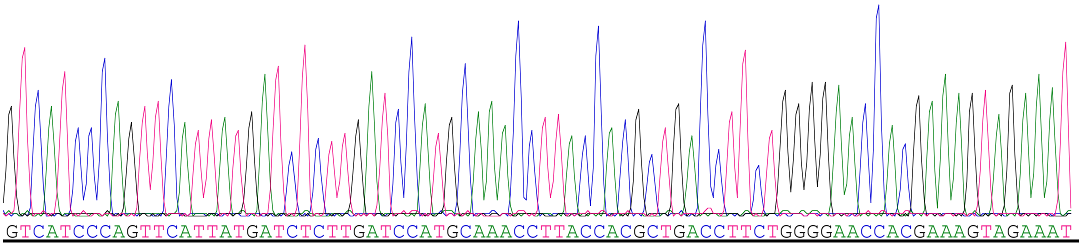

A05-PFastBac-seqF.ab1(1>814) →

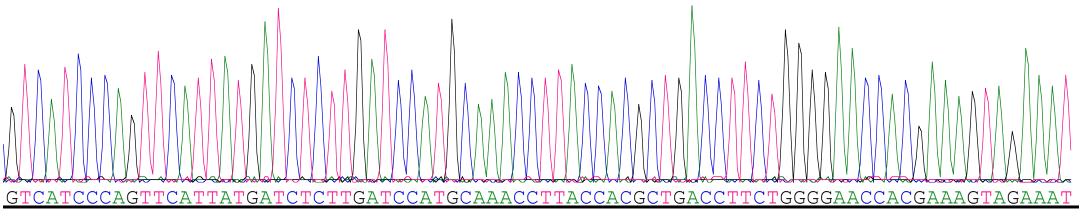

410 420 430 440 450 460 470 480

CACAGCCAGTCAGCCCACCAGCACCATCATCCTGCATAGTCACCACCTGCAGATATCTAGGGCCACCCTCAGGAAGGGAG

775569-3.seq(1>2877) cacagccagtcagcccaccagcaccatcatcctgcatagtcaccacctgcagatatctagggccaccctcaggaagggag
E03-seq4r.ab1(1>808)

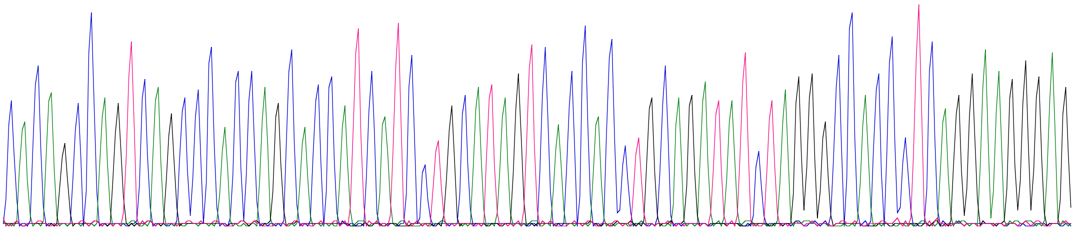

CACAGCCAGTCAGCCCACCAGCACCATCATCCTGCATAGTCACCACCTGCAGATATCTAGGGCCACCCTCAGGAAGGGAG

A05-PFastBac-seqF.ab1(1>814)

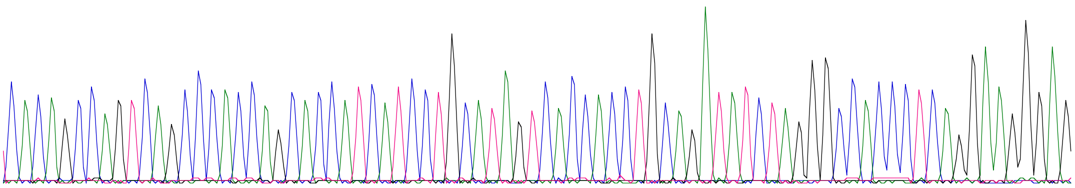

CACAGCCAGTCAGCCCACCAGCACCATCATCCTGCATAGTCACCACCTGCAGATATCTAGGGCCACCCTCAGGAAGGGAG

P127R

490 500 510 520 530 540 550 560

CTGGAGAGAGGCTATCGGAAGAACCCCTGCAGGTCCTGGAACACCCCCGTCAGGAGCAAATTGCACTGCTGGCTCCCGAG

775569-3.seq(1>2877) ctggagagaggctatcggaagaacccctgcaggtcctggaacacccccgtcaggagcaaattgcactgctggctcccgag
E03-seq4r.ab1(1>808)

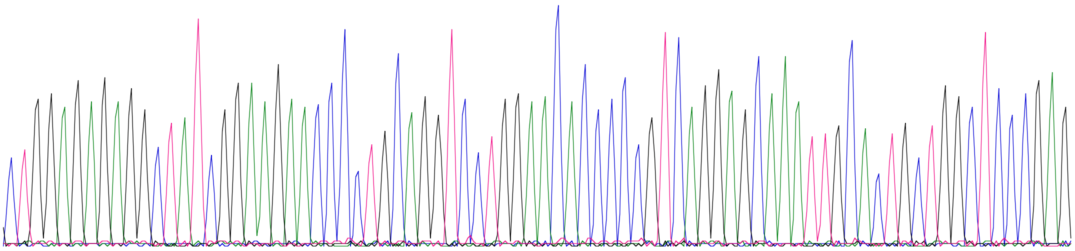

CTGGAGAGAGGCTATCGGAAGAACCCCTGCAGGTCCTGGAACACCCCCGTCAGGAGCAAATTGCACTGCTGGCTCCCGAG

A05-PFastBac-seqF.ab1(1>814)

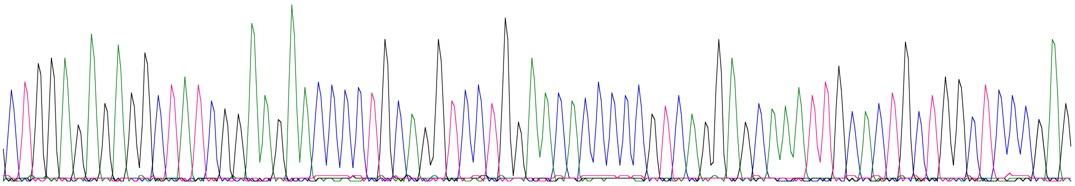

CTGGAGAGAGGCTATCGGAAGAACCCCTGCAGGTCCTGGAACACCCCCGTCAGGAGCAAATTGCACTGCTGGCTCCCGAG

570 580 590 600 610 620 630 640

CCCCTCCTTGTCGGGCTCCCGTACACAGTTGTCATTCACTATGCTGGCAATCTTTCGGAGACTTTCCACGGATTTTACAA

775569-3.seq(1>2877) cccctccttgtcgggctcccgtacacagttgtcattcactatgctggcaatctttcggagactttccacggattttacaa
E03-seq4r.ab1(1>808)

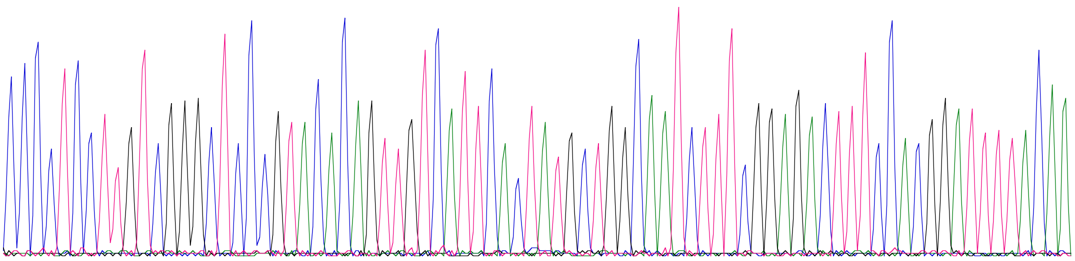

CCCCTCCTTGTCGGGCTCCCGTACACAGTTGTCATTCACTATGCTGGCAATCTTTCGGAGACTTTCCACGGATTTTACAA

570 580 590 600 610 620 630 640

CCCCTCCTTGTCGGGCTCCCGTACACAGTTGTCATTCACTATGCTGGCAATCTTTCGGAGACTTTCCACGGATTTTACAA

A05-PFastBac-seqF.ab1(1>814) →

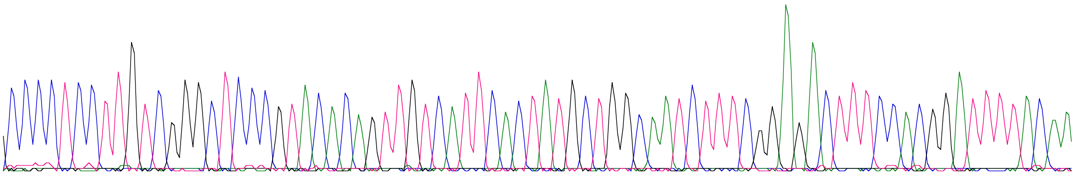

CCCCTCCTTGTCGGGCTCCCGTACACAGTTGTCATTCACTATGCTGGCAATCTTTCGGAGACTTTCCACGGATTTTACAA

650 660 670 680 690 700 710 720

AAGCACCTACAGAACCAAGGAAGGGGAACTGAGGATACTAGCATCAACACAATTTGAACCCACTGCAGCTAGAATGGCCT

775569-3.seq(1>2877) → aagcacctacagaaccaaggaaggggaactgaggatactagcatcaacacaatttgaacccactgcagctagaatggcct
E03-seq4r.ab1(1>808) ←

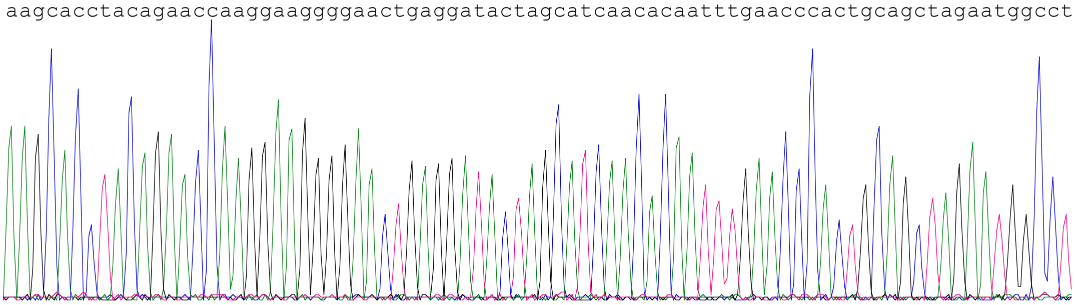

AAGCACCTACAGAACCAAGGAAGGGGAACTGAGGATACTAGCATCAACACAATTTGAACCCACTGCAGCTAGAATGGCCT

A05-PFastBac-seqF.ab1(1>814) →

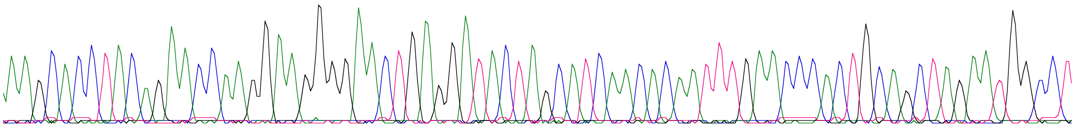

AAGCACCTACAGAACCAAGGAAGGGGAACTGAGGATACTAGCATCAACACAATTTGAACCCACTGCAGCTAGAATGGCCT

730 740 750 760 770 780 790 800

TTCCCTGCTTTGATGAACCTGCCTTCAAAGCAAGTTTCTCAATCAAAATTAGAAGAGAGCCAAGGCACCTAGCCATCTCC

775569-3.seq(1>2877) → ttccctgctttgatgaacctgccttcaaagcaagtttctcaatcaaaattagaagagagccaaggcacctagccatctcc
E03-seq4r.ab1(1>808) ←

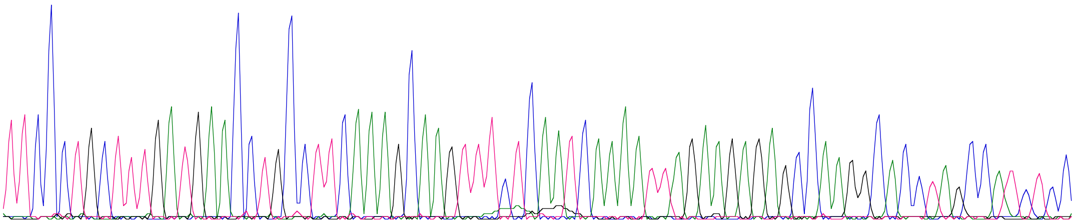

TTCCCTGCTTTGATGAACCTGCCTTCAAAGCAAGTTTCTCAATCAAAATTAGAAGAGAGCCAAGGCACCTAGCCATCTCC

A05-PFastBac-seqF.ab1(1>814) →

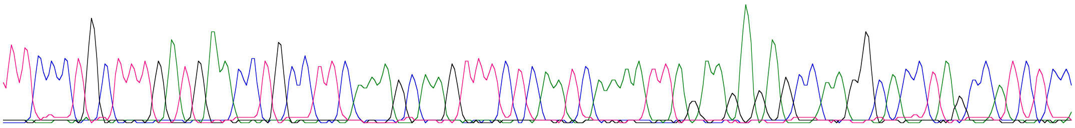

TTCCCTGCTTTGATGAACCTGCCTTCAAAGCAAGTTTCTCAATCAAAATTAGAAGAGAGCCAAGGCACCTAGCCATCTCC

730 740 750 760 770 780 790 800

TTCCCTGCTTTGATGAACCTGCCTTCAAAGCAAGTTTCTCAATCAAAATTAGAAGAGAGCCAAGGCACCTAGCCATCTCC

E04-seq5r.ab1(1>818) ←

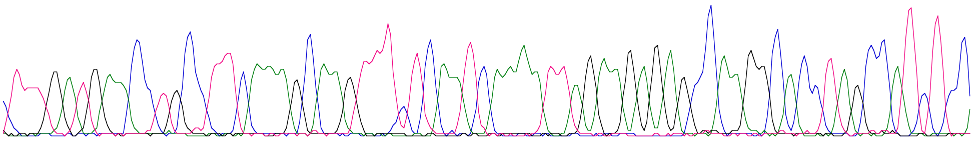

CTTTGATGAACCTGCCTTCAAAGCAAGTTTCTCAATCAAAATTAGAAGAGAGCCAAGGCACCTAGCCATCTCC

810 820 830 840 850 860 870 880

AATATGCCATTGGTGAAATCTGTGACTGTTGCTGAAGGACTCATAGAAGACCATTTTGATGTCACTGTGAAGATGAGCAC

775569-3.seq(1>2877) → aatatgccattggtgaaatctgtgactgttgctgaaggactcatagaagaccattttgatgtcactgtgaagatgagcac

E03-seq4r.ab1(1>808) ←

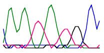

AATATGCC

A05-PFastBac-seqF.ab1(1>814) →

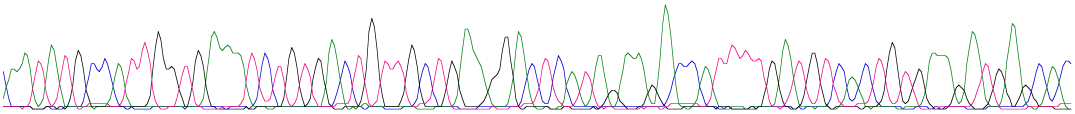

AATATGCCATTGGTGAAATCTGTGACTGTTGCTGAAGGACTCATAGAAGACCATTTTGATGTCACTGTGAAGATGAGCAC

E04-seq5r.ab1(1>818) ←

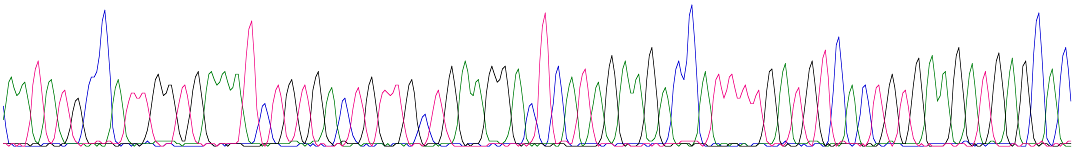

AATATGCCATTGGTGAAATCTGTGACTGTTGCTGAAGGACTCATAGAAGACCATTTTGATGTCACTGTGAAGATGAGCAC

A07-seq1.ab1(1>794) →

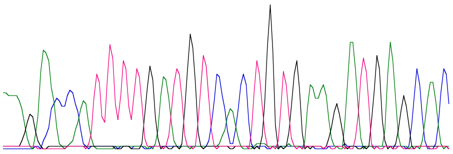

AAGACCATTTTGATGTCACTGTGAAGATGAGCAC

890 900 910 920 930 940 950 960

CTATCTGGTGGCCTTCATCATTTCAGATTTTGAGTCTGTCAGCAAGATAACCAAGAGTGGAGTCAAGGTTTCTGTTTATG

775569-3.seq(1>2877) → ctatctggtggccttcatcatttcagattttgagtctgtcagcaagataaccaagagtggagtcaaggtttctgtttatg
A05-PFastBac-seqF.ab1(1>814) →

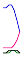
CT

E04-seq5r.ab1(1>818) ←

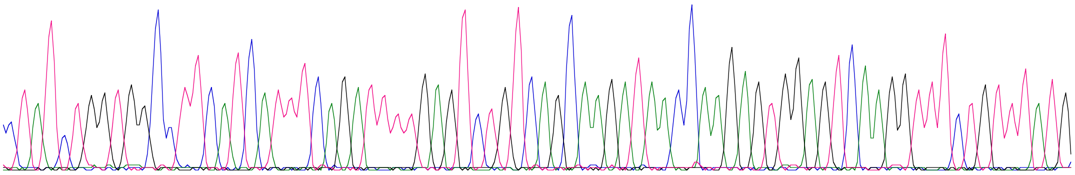
CTATCTGGTGGCCTTCATCATTTCAGATTTTGAGTCTGTCAGCAAGATAACCAAGAGTGGAGTCAAGGTTTCTGTTTATG

A07-seq1.ab1(1>794) →

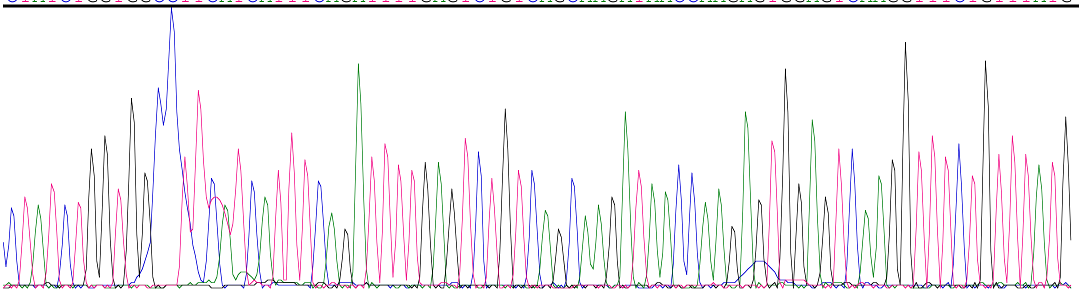
CTATCTGGTGGCCTTCATCATTTCAGATTTTGAGTCTGTCAGCAAGATAACCAAGAGTGGAGTCAAGGTTTCTGTTTATG

970 980 990 1,000 1,010 1,020 1,030 1,040

CTGTGCCAGACAAGATAAATCAAGCAGATTATGCACTGGATGCTGCGGTGACTCTTCTAGAATTTTATGAGGATTATTTC

775569-3.seq(1>2877) → ctgtgccagacaagataaatcaagcagattatgcactggatgctgcggtgactcttctagaattttatgaggattatttc
E04-seq5r.ab1(1>818) ←

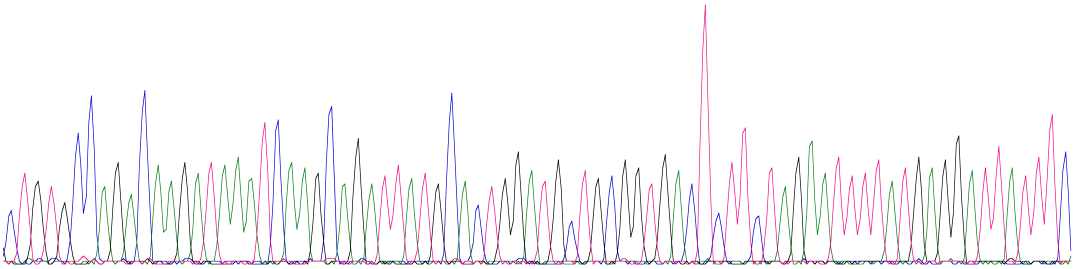
CTGTGCCAGACAAGATAAATCAAGCAGATTATGCACTGGATGCTGCGGTGACTCTTCTAGAATTTTATGAGGATTATTTC

A07-seq1.ab1(1>794) →

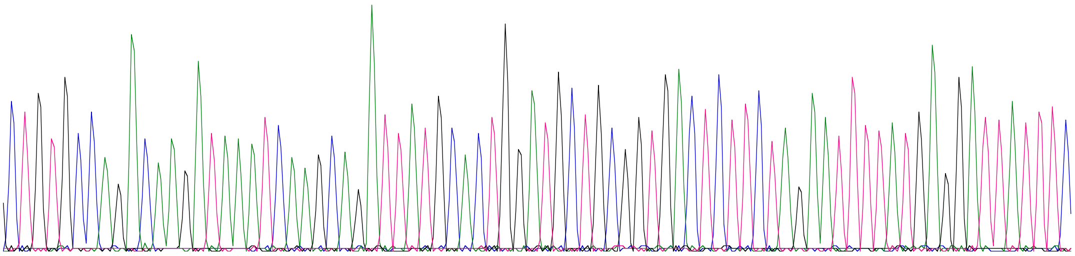
CTGTGCCAGACAAGATAAATCAAGCAGATTATGCACTGGATGCTGCGGTGACTCTTCTAGAATTTTATGAGGATTATTTC

1,050 1,060 1,070 1,080 1,090 1,100 1,110 1,120

AGCATACCGTATCCCCTACCCAAACAAGATCTTGCTGCTATTCCCGACTTTCAGTCTGGTGCTATGGAAAACTGGGGACT

775569-3.seq(1>2877) agcataccgtatcccctacccaaacaagatcttgctgctattcccgactttcagtctggtgctatggaaaactggggact
E04-seq5r.ab1(1>818)

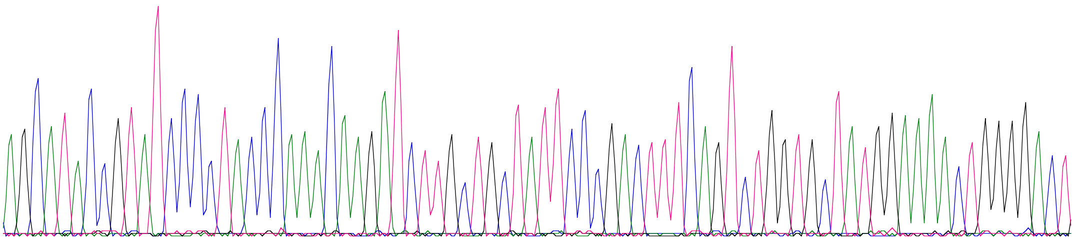

AGCATACCGTATCCCCTACCCAAACAAGATCTTGCTGCTATTCCCGACTTTCAGTCTGGTGCTATGGAAAACTGGGGACT

A07-seq1.ab1(1>794)

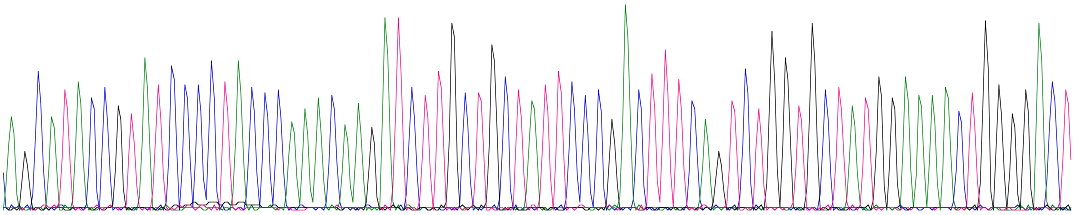

AGCATACCGTATCCCCTACCCAAACAAGATCTTGCTGCTATTCCCGACTTTCAGTCTGGTGCTATGGAAAACTGGGGACT

1,130 1,140 1,150 1,160 1,170 1,180 1,190 1,200

GACAACATATAGAGAATCTGCTCTGTTGTTTGATGCAGAAAAGTCTTCTGCATCAAGTAAGCTTGGCATCACAATGACTG

775569-3.seq(1>2877) gacaacatatagagaatctgctctgttgtttgatgcagaaaagtcttctgcatcaagtaagcttggcatcacaatgactg
E04-seq5r.ab1(1>818)

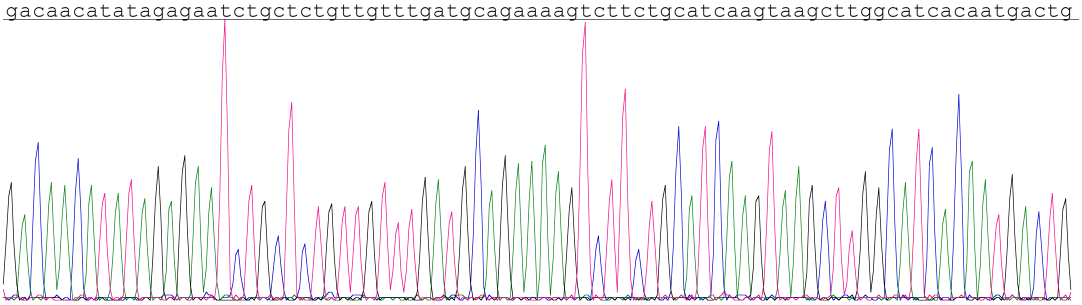

GACAACATATAGAGAATCTGCTCTGTTGTTTGATGCAGAAAAGTCTTCTGCATCAAGTAAGCTTGGCATCACAATGACTG

A07-seq1.ab1(1>794)

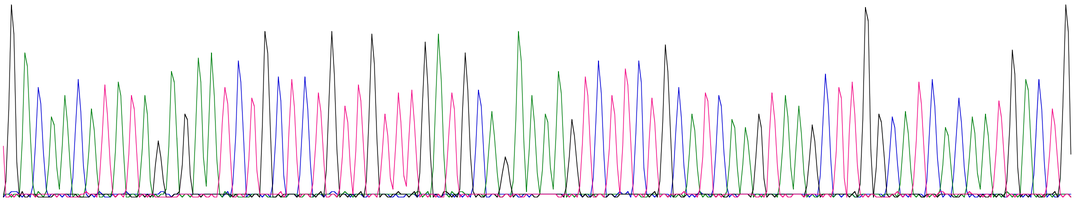

GACAACATATAGAGAATCTGCTCTGTTGTTTGATGCAGAAAAGTCTTCTGCATCAAGTAAGCTTGGCATCACAATGACTG

1,210 1,220 1,230 1,240 1,250 1,260 1,270 1,280

TGGCCCATGAACTGGCTCACCAGTGGTTTGGGAACCTGGTCACTATGGAATGGTGGAATGATCTTTGGCTAAATGAAGGA

775569-3.seq(1>2877) tggcccatgaactggctcaccagtggtttgggaacctggtcactatggaatggtggaatgatctttggctaaatgaagga
E04-seq5r.ab1(1>818)

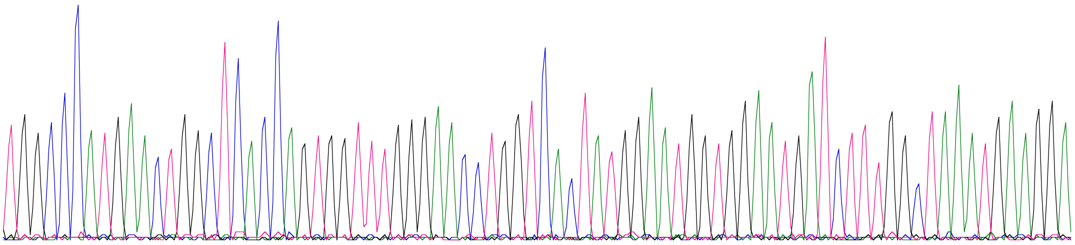

TGGCCCATGAACTGGCTCACCAGTGGTTTGGGAACCTGGTCACTATGGAATGGTGGAATGATCTTTGGCTAAATGAAGGA

1,210 1,220 1,230 1,240 1,250 1,260 1,270 1,280

TGGCCCATGAACTGGCTCACCAGTGGTTTGGGAACCTGGTCACTATGGAATGGTGGAATGATCTTTGGCTAAATGAAGGA

A07-seq1.ab1(1>794) →

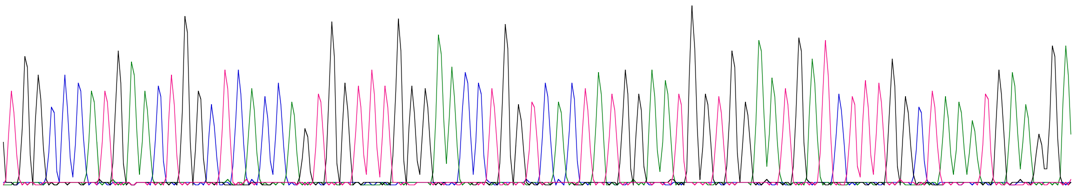

TGGCCCATGAACTGGCTCACCAGTGGTTTGGGAACCTGGTCACTATGGAATGGTGGAATGATCTTTGGCTAAATGAAGGA

1,290 1,300 1,310 1,320 1,330 1,340 1,350 1,360

TTTGCCAAATTTATGGAGTTTGTGTCTGTCAGTGTGACCCATCCTGAACTGAAAGTTGGAGATTATTTCTTTGGCAAATG

775569-3.seq(1>2877) → tttgccaaatttatggagtttgtgtctgtcagtgtgacccatcctgaactgaaagttggagattatttctttggcaaatg

E04-seq5r.ab1(1>818) ←

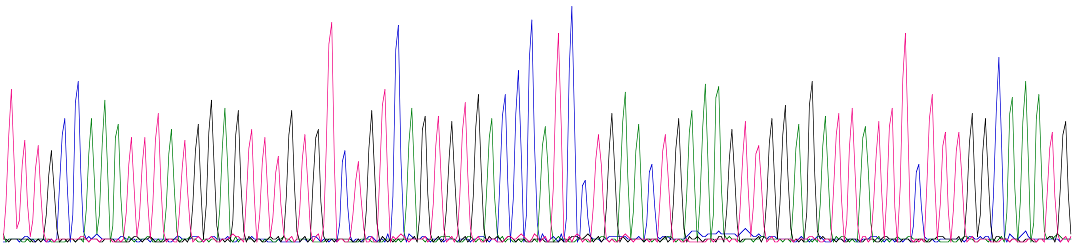

TTTGCCAAATTTATGGAGTTTGTGTCTGTCAGTGTGACCCATCCTGAACTGAAAGTTGGAGATTATTTCTTTGGCAAATG

A07-seq1.ab1(1>794) →

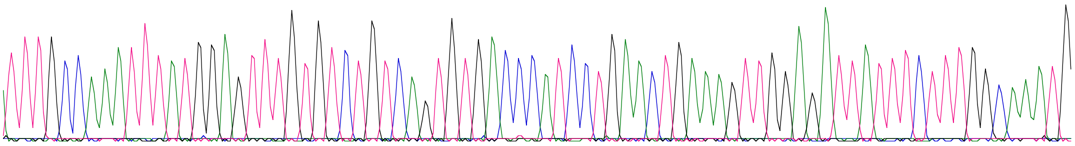

TTTGCCAAATTTATGGAGTTTGTGTCTGTCAGTGTGACCCATCCTGAACTGAAAGTTGGAGATTATTTCTTTGGCAAATG

1,370 1,380 1,390 1,400 1,410 1,420 1,430 1,440

TTTTGACGCAATGGAGGTAGATGCTTTAAATTCCTCACACCCTGTGTCTACACCTGTGGAAAATCCTGCTCAGATCCGGG

775569-3.seq(1>2877) → ttttgacgcaatggaggtagatgctttaaattcctcacaccctgtgtctacacctgtggaaaatcctgctcagatccggg

E04-seq5r.ab1(1>818) ←

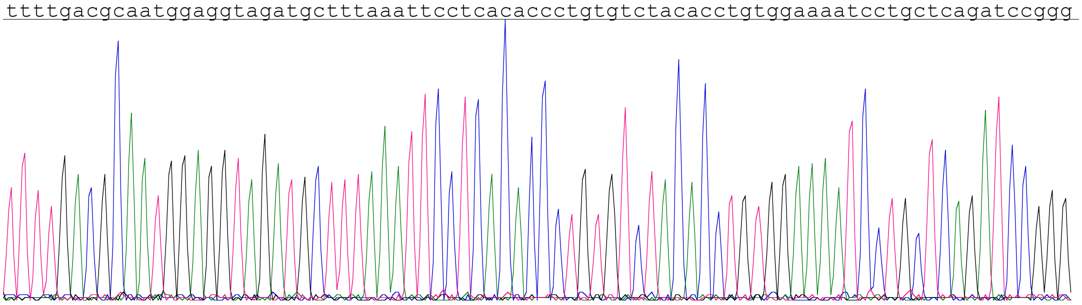

TTTTGACGCAATGGAGGTAGATGCTTTAAATTCCTCACACCCTGTGTCTACACCTGTGGAAAATCCTGCTCAGATCCGGG

A07-seq1.ab1(1>794) →

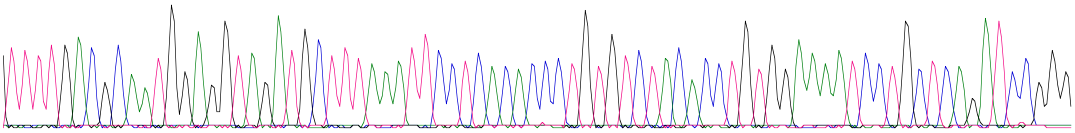

TTTTGACGCAATGGAGGTAGATGCTTTAAATTCCTCACACCCTGTGTCTACACCTGTGGAAAATCCTGCTCAGATCCGGG

1,450 1,460 1,470 1,480 1,490 1,500 1,510 1,520

AGATGTTTGATGATGTTTCTTATGATAAGGGAGCTTGTATTCTGAATATGCTAAGGGAGTATCTTAGTGCTGACGCATTT

775569-3.seq(1>2877) → agatgtttgatgatgtttcttatgataagggagcttgtattctgaatatgctaagggagtatcttagtgctgacgcattt

E04-seq5r.ab1(1>818) ←

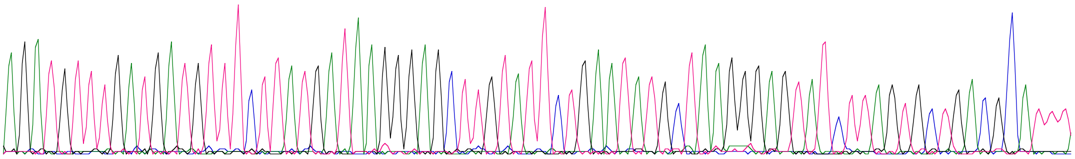

AGATGTTTGATGATGTTTCTTATGATAAGGGAGCTTGTATTCTGAATATGCTAAGGGAGTATCTTAGTGCTGACGCATTT

A07-seq1.ab1(1>794) →

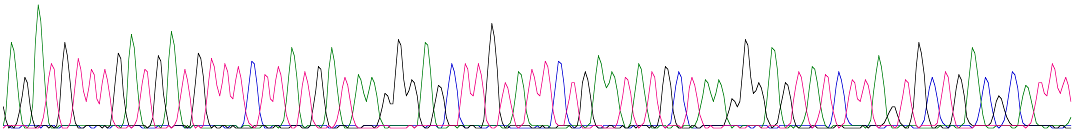

AGATGTTTGATGATGTTTCTTATGATAAGGGAGCTTGTATTCTGAATATGCTAAGGGAGTATCTTAGTGCTGACGCATTT

E05-seq6r.ab1(1>817) ←

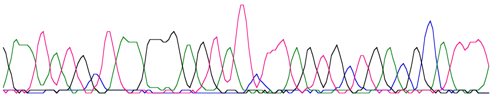

GAATATGCTAAGGGAGTATCTTAGTGCTGACGCATTT

1,530 1,540 1,550 1,560 1,570 1,580 1,590 1,600

AAAAGTGGTATTGTACAGTATCTCCAGAAGCATAGCTATAAAAATACAAAAAACGAGGACCTGTGGGATAGTATGGCAAG

775569-3.seq(1>2877) → aaaagtggtattgtacagtatctccagaagcatagctataaaaatacaaaaaacgaggacctgtgggatagtatggcaag

E04-seq5r.ab1(1>818) ←

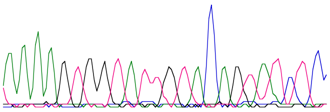

AAAAGTGGTATTGTACAGTATCTCC

A07-seq1.ab1(1>794) →

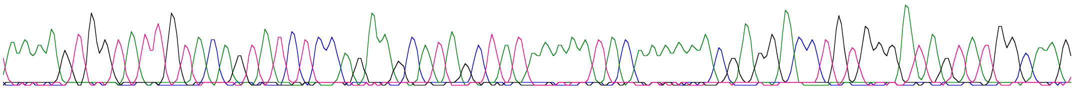

AAAAGTGGTATTGTACAGTATCTCCAGAAGCATAGCTATAAAAATACAAAAAACGAGGACCTGTGGGATAGTATGGCAAG

E05-seq6r.ab1(1>817) ←

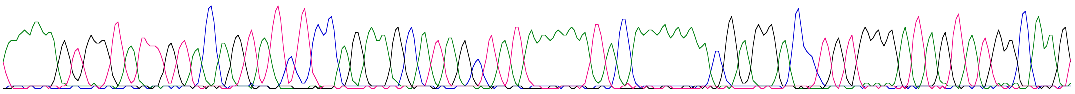

AAAAGTGGTATTGTACAGTATCTCCAGAAGCATAGCTATAAAAATACAAAAAACGAGGACCTGTGGGATAGTATGGCAAG

1,530 1,540 1,550 1,560 1,570 1,580 1,590 1,600

AAAAGTGGTATTGTACAGTATCTCCAGAAGCATAGCTATAAAAATACAAAAAACGAGGACCTGTGGGATAGTATGGCAAG

A08-seq2.ab1(1>797) →

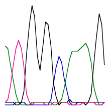

ATGGCAAG

1,610 1,620 1,630 1,640 1,650 1,660 1,670 1,680

TATTTGCCCTACAGATGGTGTAAAAGGGATGGATGGCTTTTGCTCTAGAAGTCAACATTCATCTTCATCCTCACATTGGC

775569-3.seq(1>2877) → tatttgccctacagatggtgtaaaagggatggatggcttttgctctagaagtcaacattcatcttcatcctcacattggc
A07-seq1.ab1(1>794) →

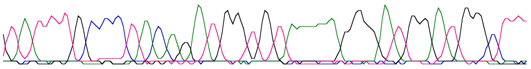

TATTTGCCCTACAGATGGTGTAAAAGGGATGGATGGCTTT

E05-seq6r.ab1(1>817) ←

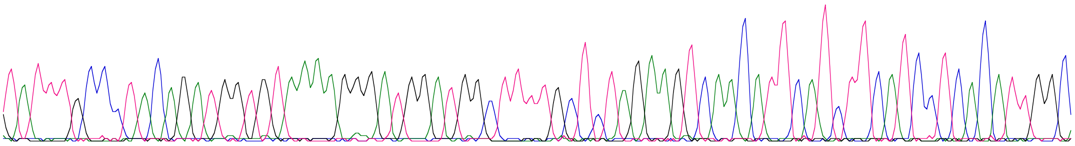

TATTTGCCCTACAGATGGTGTAAAAGGGATGGATGGCTTTTGCTCTAGAAGTCAACATTCATCTTCATCCTCACATTGGC

A08-seq2.ab1(1>797) →

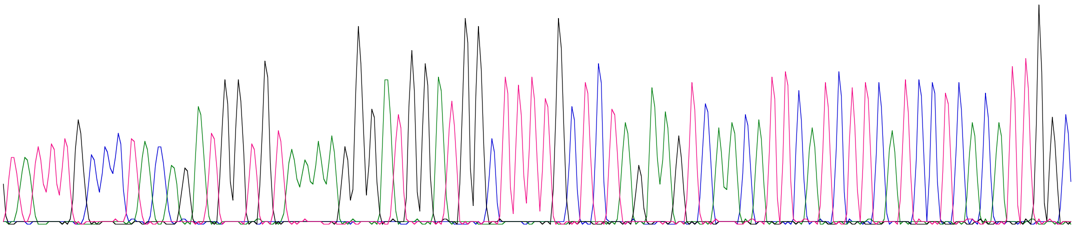

TATTTGCCCTACAGATGGTGTAAAAGGGATGGATGGCTTTTGCTCTAGAAGTCAACATTCATCTTCATCCTCACATTGGC

1,690 1,700 1,710 1,720 1,730 1,740 1,750 1,760

ATCAGGAAGGGGTGGATGTGAAAACCATGATGAACACTTGGACACTGCAGAAGGGTTTTCCCCTAATAACCATCACAGTG

775569-3.seq(1>2877) → atcaggaaggggtggatgtgaaaaccatgatgaacacttggacactgcagaagggttttcccctaataaccatcacagtg
E05-seq6r.ab1(1>817) ←

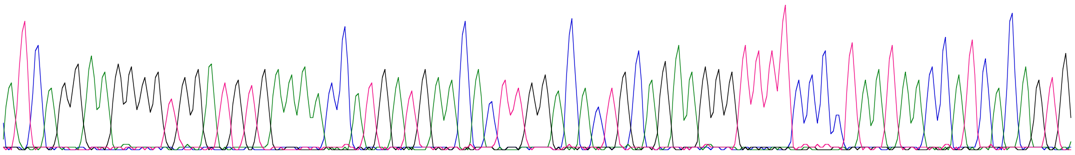

ATCAGGAAGGGGTGGATGTGAAAACCATGATGAACACTTGGACACTGCAGAAGGGTTTTCCCCTAATAACCATCACAGTG

1,690 1,700 1,710 1,720 1,730 1,740 1,750 1,760

ATCAGGAAGGGGTGGATGTGAAAACCATGATGAACACTTGGACACTGCAGAAGGGTTTTCCCCTAATAACCATCACAGTG

A08-seq2.ab1(1>797) →

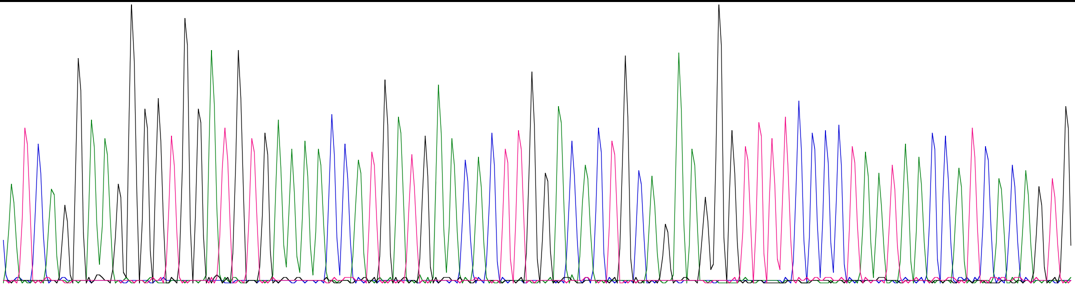

ATCAGGAAGGGGTGGATGTGAAAACCATGATGAACACTTGGACACTGCAGAAGGGTTTTCCCCTAATAACCATCACAGTG

1,770 1,780 1,790 1,800 1,810 1,820 1,830 1,840

AGGGGGAGGAATGTACACATGAAGCAAGAGCACTACATGAAGGGCTCTGACGGCGCCCCGGACACTGGGTACCTGTGGCA

775569-3.seq(1>2877) → agggggaggaatgtacacatgaagcaagagcactacatgaagggctctgacggcgccccggacactgggtacctgtggca

E05-seq6r.ab1(1>817) ←

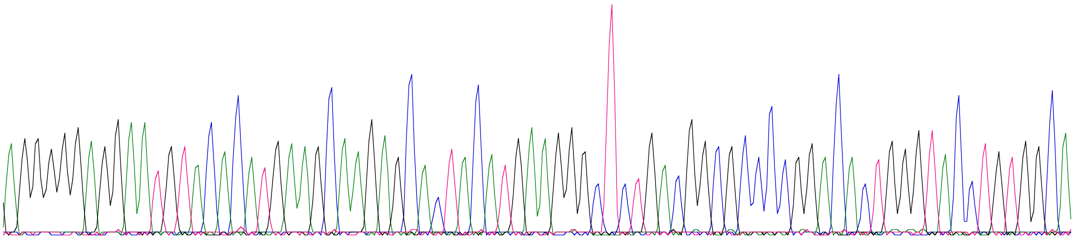

AGGGGGAGGAATGTACACATGAAGCAAGAGCACTACATGAAGGGCTCTGACGGCGCCCCGGACACTGGGTACCTGTGGCA

A08-seq2.ab1(1>797) →

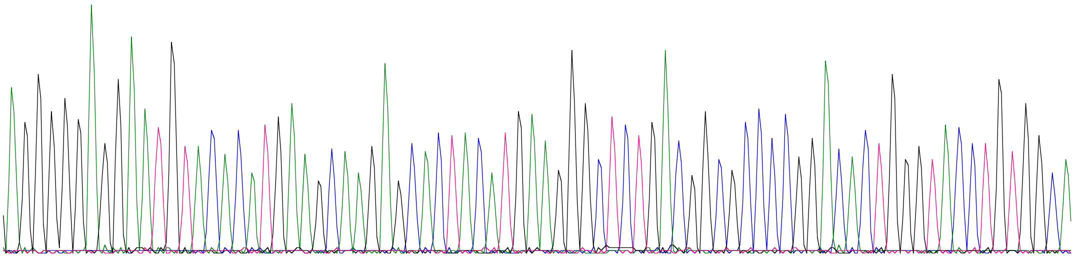

AGGGGGAGGAATGTACACATGAAGCAAGAGCACTACATGAAGGGCTCTGACGGCGCCCCGGACACTGGGTACCTGTGGCA

1,850 1,860 1,870 1,880 1,890 1,900 1,910 1,920

TGTTCCATTGACATTCATCACCAGCAAATCCGACATGGTCCATCGATTTTTGCTAAAAACAAAAACAGATGTGCTCATCC

775569-3.seq(1>2877) → tgttccattgacattcatcaccagcaaatccgacatggtccatcgatttttgctaaaaacaaaaacagatgtgctcatcc

E05-seq6r.ab1(1>817) ←

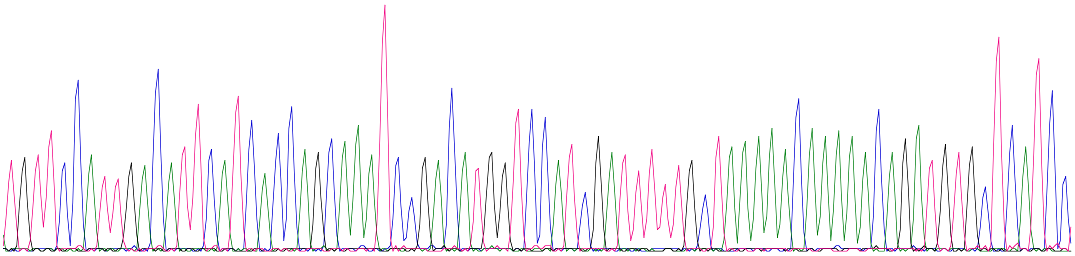

TGTTCCATTGACATTCATCACCAGCAAATCCGACATGGTCCATCGATTTTTGCTAAAAACAAAAACAGATGTGCTCATCC

A08-seq2.ab1(1>797) →

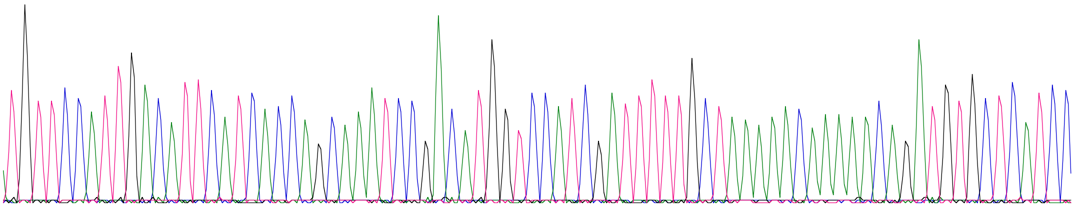

TGTTCCATTGACATTCATCACCAGCAAATCCGACATGGTCCATCGATTTTTGCTAAAAACAAAAACAGATGTGCTCATCC

1,930 1,940 1,950 1,960 1,970 1,980 1,990 2,000

TCCCAGAAGAGGTGGAATGGATCAAATTTAATGTGGGCATGAATGGCTATTACATTGTGCATTACGAGGATGATGGATGG

775569-3.seq(1>2877) tcccagaagaggtggaatggatcaaatttaatgtgggcatgaatggctattacattgtgcattacgaggatgatggatgg
E05-seq6r.ab1(1>817)

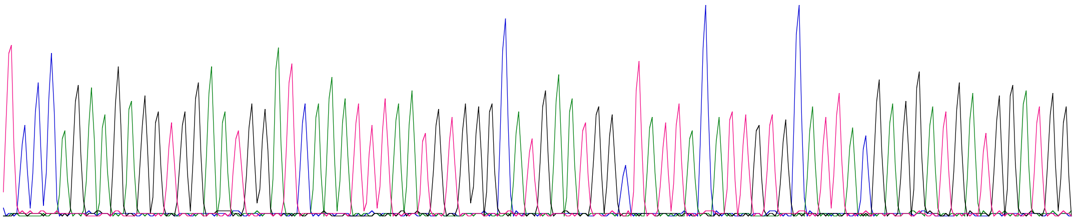

TCCCAGAAGAGGTGGAATGGATCAAATTTAATGTGGGCATGAATGGCTATTACATTGTGCATTACGAGGATGATGGATGG

A08-seq2.ab1(1>797)

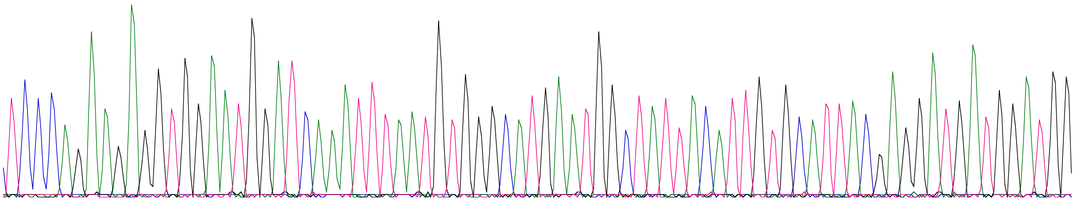

TCCCAGAAGAGGTGGAATGGATCAAATTTAATGTGGGCATGAATGGCTATTACATTGTGCATTACGAGGATGATGGATGG

2,010 2,020 2,030 2,040 2,050 2,060 2,070 2,080

GACTCTTTGACTGGCCTTTTAAAAGGAACACACACAGCAGTCAGCAGTAATGATCGGGCGAGTCTCATTAACAATGCATT

775569-3.seq(1>2877) gactctttgactggccttttaaaaggaacacacacagcagtcagcagtaatgatcgggcgagtctcattaacaatgcatt
E05-seq6r.ab1(1>817)

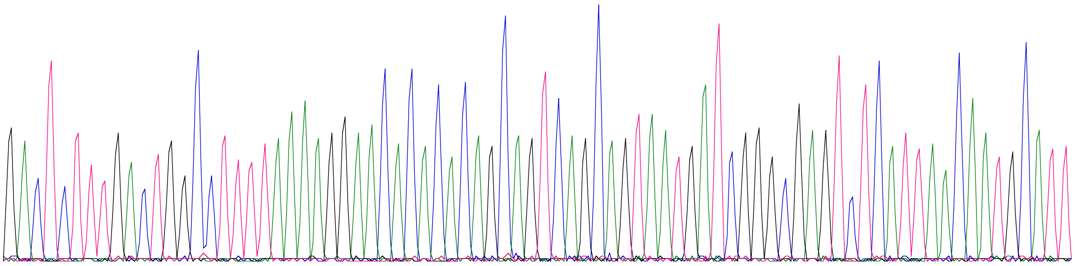

GACTCTTTGACTGGCCTTTTAAAAGGAACACACACAGCAGTCAGCAGTAATGATCGGGCGAGTCTCATTAACAATGCATT

A08-seq2.ab1(1>797)

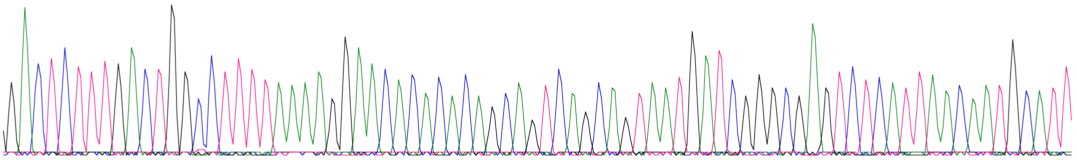

GACTCTTTGACTGGCCTTTTAAAAGGAACACACACAGCAGTCAGCAGTAATGATCGGGCGAGTCTCATTAACAATGCATT

2,090 2,100 2,110 2,120 2,130 2,140 2,150 2,160

TCAGCTCGTCAGCATTGGGAAGCTGTCCATTGAAAAGGCCTTGGATTTATCCCTGTACTTGAAACATGAAACTGAAATTA

775569-3.seq(1>2877) tcagctcgtcagcattgggaagctgtccattgaaaaggccttggatttatccctgtacttgaaacatgaaactgaaatta
E05-seq6r.ab1(1>817)

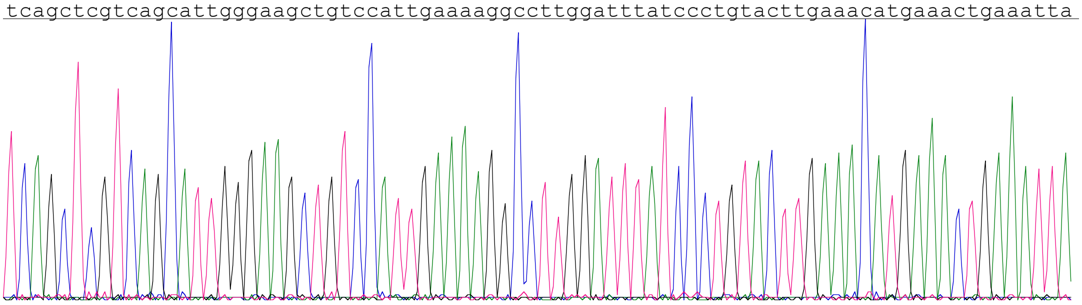

TCAGCTCGTCAGCATTGGGAAGCTGTCCATTGAAAAGGCCTTGGATTTATCCCTGTACTTGAAACATGAAACTGAAATTA

2,090 2,100 2,110 2,120 2,130 2,140 2,150 2,160

TCAGCTCGTCAGCATTGGGAAGCTGTCCATTGAAAAGGCCTTGGATTTATCCCTGTACTTGAAACATGAAACTGAAATTA

A08-seq2.ab1(1>797) →

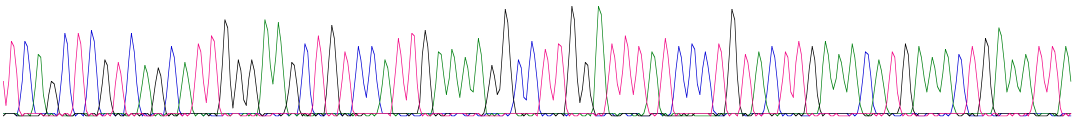

TCAGCTCGTCAGCATTGGGAAGCTGTCCATTGAAAAGGCCTTGGATTTATCCCTGTACTTGAAACATGAAACTGAAATTA

2,170 2,180 2,190 2,200 2,210 2,220 2,230 2,240

TGCCCGTGTTTCAAGGTTTGAATGAGCTGATTCCTATGTATAAGTTAATGGAGAAAAGAGATATGAATGAAGTGGAAACT

775569-3.seq(1>2877) → tgcccgtgtttcaaggtttgaatgagctgattcctatgtataagttaatggagaaaagagatatgaatgaagtggaaact
E05-seq6r.ab1(1>817) ←

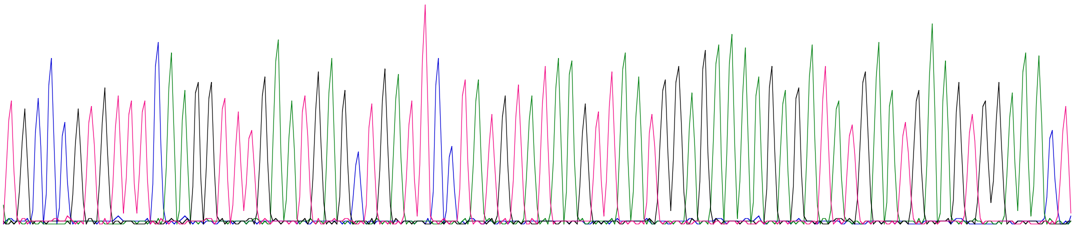

TGCCCGTGTTTCAAGGTTTGAATGAGCTGATTCCTATGTATAAGTTAATGGAGAAAAGAGATATGAATGAAGTGGAAACT

A08-seq2.ab1(1>797) →

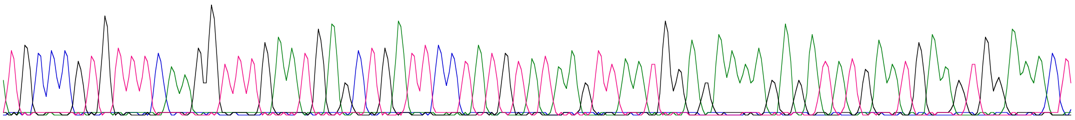

TGCCCGTGTTTCAAGGTTTGAATGAGCTGATTCCTATGTATAAGTTAATGGAGAAAAGAGATATGAATGAAGTGGAAACT

2,250 2,260 2,270 2,280 2,290 2,300 2,310 2,320

CAATTCAAGGCCTTCCTCATCAGGCTGCTAAGGGACCTCATTGATAAGCAGACATGGACAGACGAGGGCTCAGTCTCAGA

775569-3.seq(1>2877) → caattcaaggccttcctcatcaggctgctaagggacctcattgataagcagacatggacagacgagggctcagtctcaga
E05-seq6r.ab1(1>817) ←

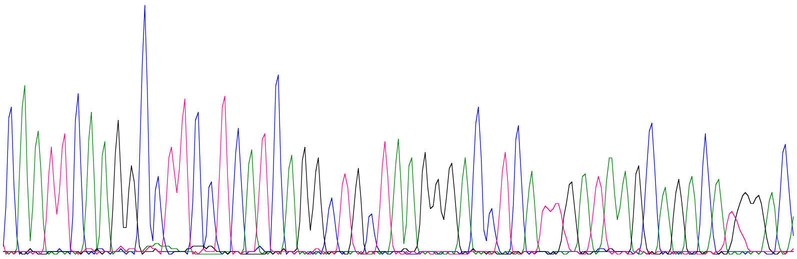

CAATTCAAGGCCTTCCTCATCAGGCTGCTAAGGGACCTCATTGATAAGCAGACATGGACA

A08-seq2.ab1(1>797) →

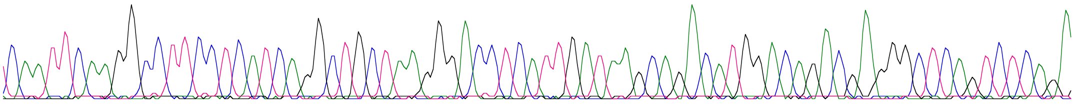

CAATTCAAGGCCTTCCTCATCAGGCTGCTAAGGGACCTCATTGATAAGCAGACATGGACAGACGAGGGCTCAGTCTCAGA

2,250 2,260 2,270 2,280 2,290 2,300 2,310 2,320

CAATTCAAGGCCTTCCTCATCAGGCTGCTAAGGGACCTCATTGATAAGCAGACATGGACAGACGAGGGCTCAGTCTCAGA

A06-pfastbac1-R.ab1(1>809) ←

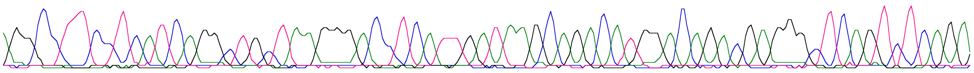

AGGCCTTCCTCATCAGGCTGCTAAGGGACCTCATTGATAAGCAGACATGGACAGACGAGGGCTCAGTCTCAGA

2,330 2,340 2,350 2,360 2,370 2,380 2,390 2,400

GCGAATGCTGCGGAGTCAACTACTACTCCTCGCCTGTGTGCACAACTATCAGCCGTGCGTACAGAGGGCAGAAGGCTATT

775569-3.seq(1>2877) → gcgaatgctgcggagtcaactactactcctcgcctgtgtgcacaactatcagccgtgcgtacagagggcagaaggctatt

A08-seq2.ab1(1>797) →

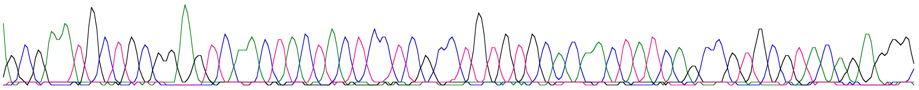

GCGAATGCTGCGGAGTCAACTACTACTCCTCGCCTGTGTGCACAACTATCAGCCGTGCGTACAGAGGGC

A06-pfastbac1-R.ab1(1>809) ←

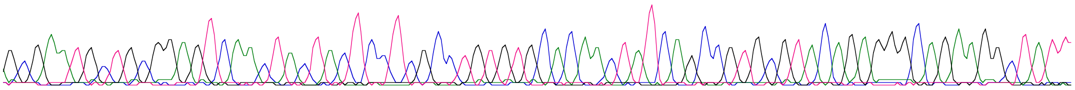

GCGAATGCTGCGGAGTCAACTACTACTCCTCGCCTGTGTGCACAACTATCAGCCGTGCGTACAGAGGGCAGAAGGCTATT

E02-seq3.ab1(1>802) →

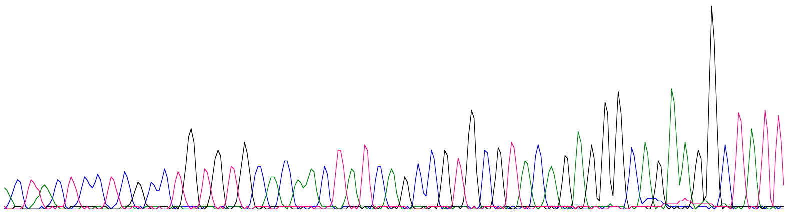

ACTACTCCTCGCCTGTGTGCACAACTATCAGCCGTGCGTACAGAGGGCAGAAGGCTATT

2,410 2,420 2,430 2,440 2,450 2,460 2,470 2,480

TCAGAAAGTGGAAGGAATCCAATGGAAACTTGAGCCTGCCTGTCGACGTGACCTTGGCAGTGTTTGCTGTGGGGGCCCAG

775569-3.seq(1>2877) → tcagaaagtggaaggaatccaatggaaacttgagcctgcctgtcgacgtgaccttggcagtgtttgctgtgggggcccag

A06-pfastbac1-R.ab1(1>809) ←

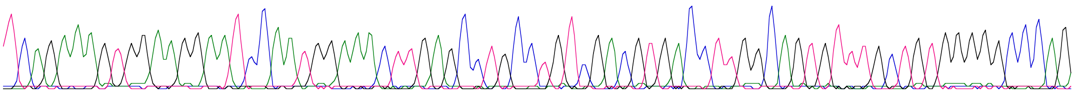

TCAGAAAGTGGAAGGAATCCAATGGAAACTTGAGCCTGCCTGTCGACGTGACCTTGGCAGTGTTTGCTGTGGGGGCCCAG

2,410 2,420 2,430 2,440 2,450 2,460 2,470 2,480

TCAGAAAGTGGAAGGAATCCAATGGAAACTTGAGCCTGCCTGTCGACGTGACCTTGGCAGTGTTTGCTGTGGGGGCCCAG

E02-seq3.ab1(1>802) →

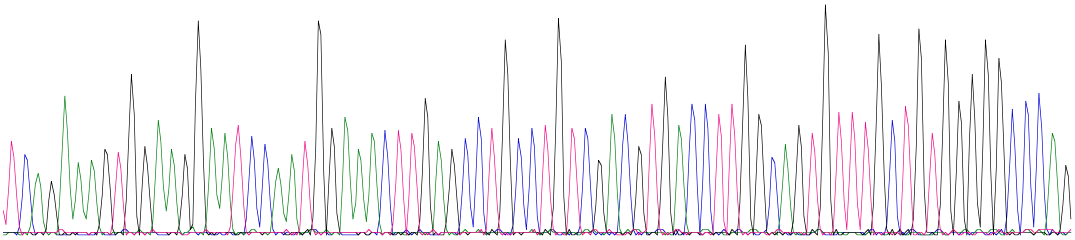

TCAGAAAGTGGAAGGAATCCAATGGAAACTTGAGCCTGCCTGTCGACGTGACCTTGGCAGTGTTTGCTGTGGGGGCCCAG

2,490 2,500 2,510 2,520 2,530 2,540 2,550 2,560

AGCACAGAAGGCTGGGATTTTCTTTATAGTAAATATCAGTTTTCTTTGTCCAGTACTGAGAAAAGCCAAATTGAATTTGC

775569-3.seq(1>2877) →
A06-pfastbac1-R.ab1(1>809) ←

agcacagaaggctgggattttctttatagtaaatatcagttttctttgtccagtactgagaaaagccaaattgaatttgc

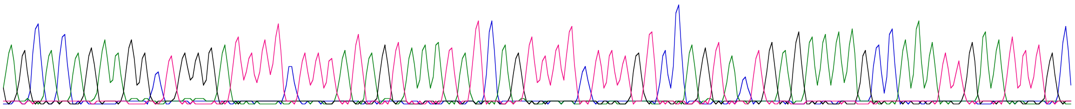

AGCACAGAAGGCTGGGATTTTCTTTATAGTAAATATCAGTTTTCTTTGTCCAGTACTGAGAAAAGCCAAATTGAATTTGC

E02-seq3.ab1(1>802) →

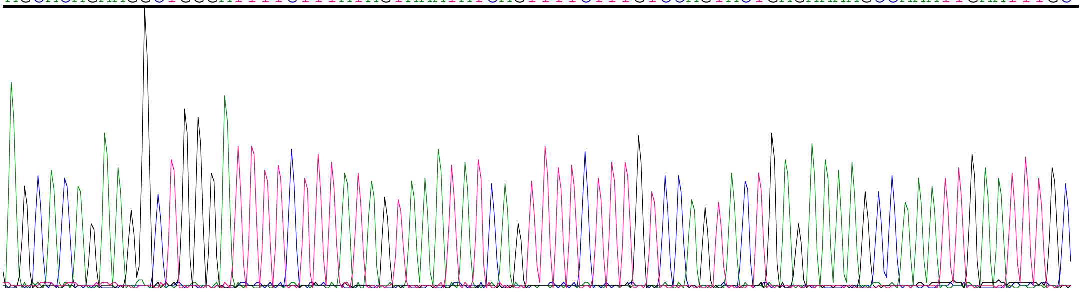

AGCACAGAAGGCTGGGATTTTCTTTATAGTAAATATCAGTTTTCTTTGTCCAGTACTGAGAAAAGCCAAATTGAATTTGC

2,570 2,580 2,590 2,600 2,610 2,620 2,630 2,640

CCTCTGCAGAACCCAAAATAAGGAAAAGCTTCAATGGCTACTAGATGAAAGCTTTAAGGGAGATAAAATAAAAACTCAGG

775569-3.seq(1>2877) →
A06-pfastbac1-R.ab1(1>809) ←

cctctgcagaacccaaaataaggaaaagcttcaatggctactagatgaaagctttaagggagataaaataaaaactcagg

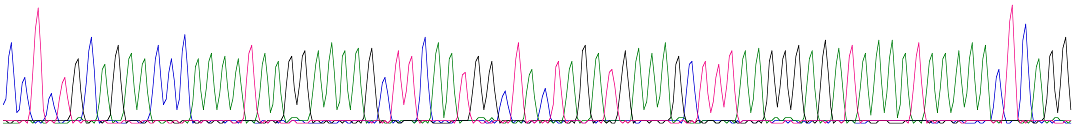

CCTCTGCAGAACCCAAAATAAGGAAAAGCTTCAATGGCTACTAGATGAAAGCTTTAAGGGAGATAAAATAAAAACTCAGG

E02-seq3.ab1(1>802) →

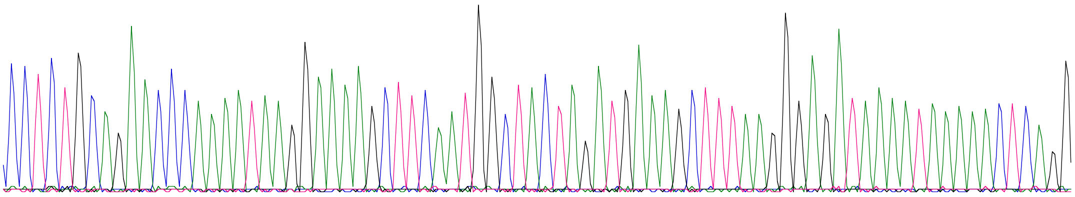

CCTCTGCAGAACCCAAAATAAGGAAAAGCTTCAATGGCTACTAGATGAAAGCTTTAAGGGAGATAAAATAAAAACTCAGG

2,650 2,660 2,670 2,680 2,690 2,700 2,710 2,720

AGTTTCCACAAATTCTTACACTCATTGGCAGGAACCCAGTAGGATACCCACTGGCCTGGCAATTTCTGAGGAAAAACTGG

775569-3.seq(1>2877) → agtttccacaaattcttacactcattggcaggaacccagtaggatacccactggcctggcaatttctgaggaaaaactgg
A06-pfastbac1-R.ab1(1>809) ←

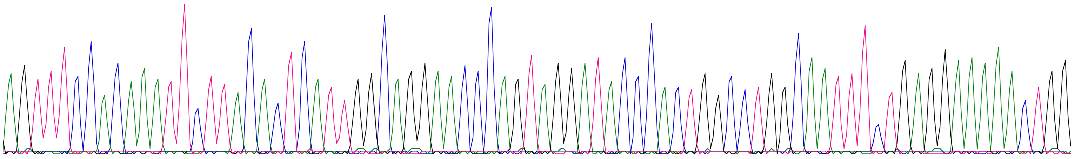

AGTTTCCACAAATTCTTACACTCATTGGCAGGAACCCAGTAGGATACCCACTGGCCTGGCAATTTCTGAGGAAAAACTGG

E02-seq3.ab1(1>802) →

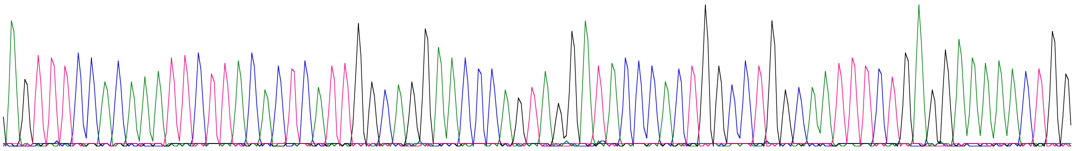

AGTTTCCACAAATTCTTACACTCATTGGCAGGAACCCAGTAGGATACCCACTGGCCTGGCAATTTCTGAGGAAAAACTGG

2,730 2,740 2,750 2,760 2,770 2,780 2,790 2,800

AACAAACTTGTACAAAAGTTTGAACTTGGCTCATCTTCCATAGCCCACATGGTAATGGGTACAACAAATCAATTCTCCAC

775569-3.seq(1>2877) → aacaaacttgtacaaaagtttgaacttggctcatcttccatagcccacatggtaatgggtacaacaaatcaattctccac
A06-pfastbac1-R.ab1(1>809) ←

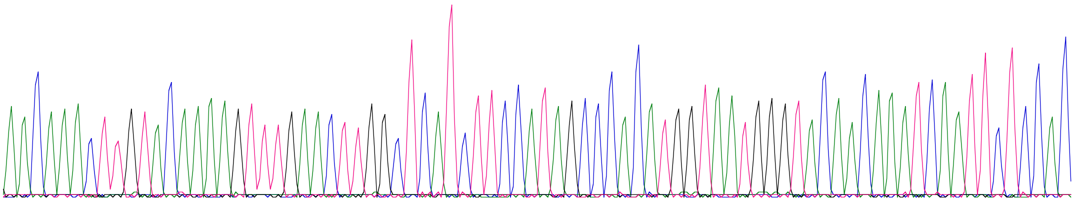

AACAAACTTGTACAAAAGTTTGAACTTGGCTCATCTTCCATAGCCCACATGGTAATGGGTACAACAAATCAATTCTCCAC

E02-seq3.ab1(1>802) →

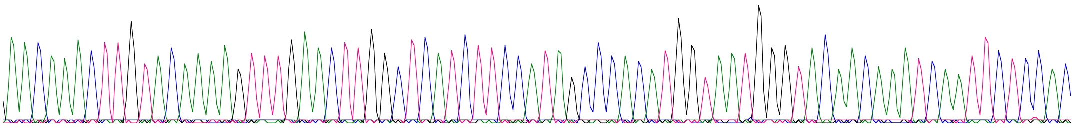

AACAAACTTGTACAAAAGTTTGAACTTGGCTCATCTTCCATAGCCCACATGGTAATGGGTACAACAAATCAATTCTCCAC

2,810 2,820 2,830 2,840 2,850 2,860 2,870 2,880

AAGAACACGGCTTGAAGAGGTAAAAGGATTCTTCAGCTCTTTGAAGAAAATGGTTCTCAGCTCCGTTGTGTCCAACAGA

775569-3.seq(1>2877) → aagaacacggcttgaagaggtaaaaggattcttcagctctttgaagaaaatggttctcagctccgttgtgtccaacaga
A06-pfastbac1-R.ab1(1>809) ←

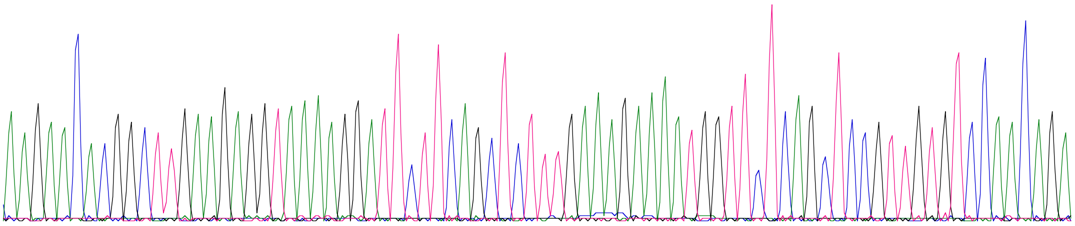

AAGAACACGGCTTGAAGAGGTAAAAGGATTCTTCAGCTCTTTGAAGAAAATGGTTCTCAGCTCCGTTGTGTCCAACAGA

2,810 2,820 2,830 2,840 2,850 2,860 2,870 2,880

AAGAACACGGCTTGAAGAGGTAAAAGGATTCTTCAGCTCTTTGAAAGAAAATGGTTCTCAGCTCCGTTGTGTCCAACAGA

E02-seq3.ab1(1>802) →

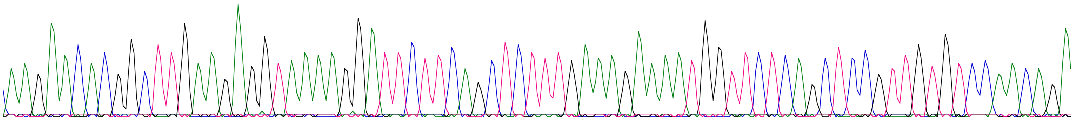

AAGAACACGGCTTGAAGAGGTAAAAGGATTCTTCAGCTCTTTGAAAGAAAATGGTTCTCAGCTCCGTTGTGTCCAACAGA

2,890 2,900 2,910 2,920 2,930 2,940 2,950 2,960

CAATTGAAACCATTGAAGAAAACATCGGTTGGATGGATAAGAATTTTGATAAAATCAGAGTGTGGCTGCAAAGTGAAAAG

775569-3.seq(1>2877) →
A06-pfastbac1-R.ab1(1>809) ←

caattgaaaccattgaagaaaacatcggttggatggataagaattttgataaaatcagagtgtggctgcaaagtgaaaag

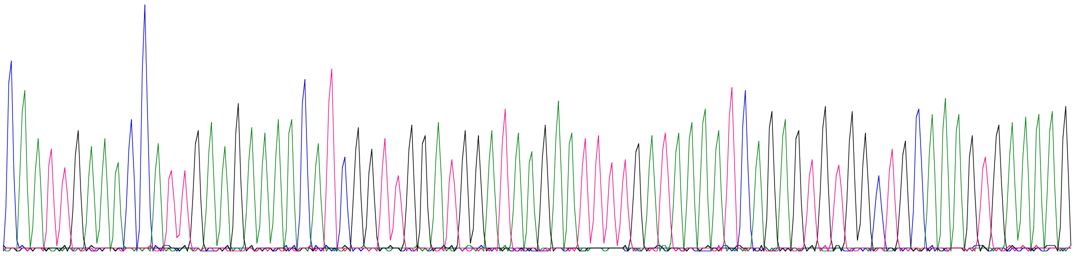

CAATTGAAACCATTGAAGAAAACATCGGTTGGATGGATAAGAATTTTGATAAAATCAGAGTGTGGCTGCAAAGTGAAAAG

E02-seq3.ab1(1>802) →

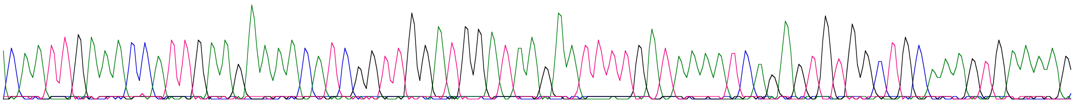

CAATTGAAACCATTGAAGAAAACATCGGTTGGATGGATAAGAATTTTGATAAAATCAGAGTGTGGCTGCAAAGTGAAAAG

2,970 2,980 2,990 3,000 3,010 3,020 3,030 3,040

CTTGAACGCATGCATCATCACCATCACCATTAACTCGAGGCATGCGGTACCAAGCTTGTCGAGAAGTACTAGAGGATCAT

775569-3.seq(1>2877) →
A06-pfastbac1-R.ab1(1>809) ←

cttgaaCGCATGCATCATCACCATCACCATtaaCTCGAG

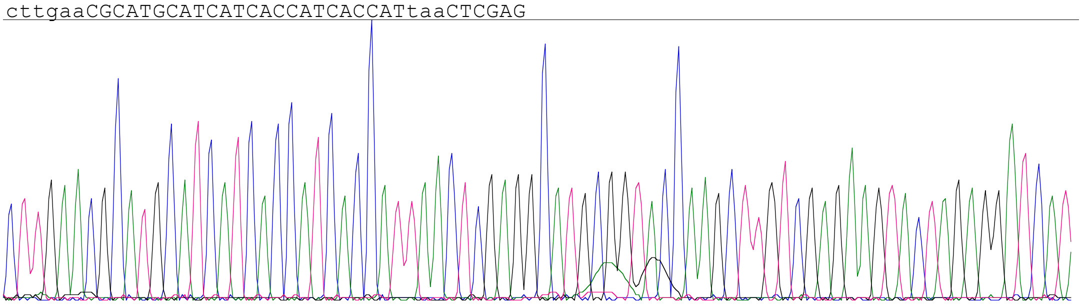

CTTGAACGCATGCATCATCACCATCACCATTAACTCGAGGCATGCGGTACCAAGCTTGTCGAGAAGTACTAGAGGATCAT

E02-seq3.ab1(1>802) →

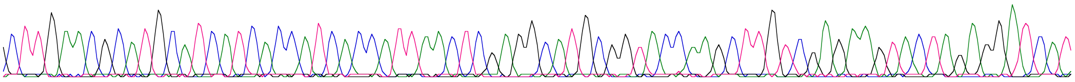

CTTGAACGCATGCATCATCACCATCACCATTAACTCGAGGCATGCGGTACCAAGCTTGTCGAGAAGTACTAGAGGATCAT

3,050 3,060 3,070 3,080 3,090 3,100 3,110 3,120

AATCAGCCATACCACATTTGTAGAGGTTTTACTTGCTTTAAAAAACCTCCCACACCTCCCCCTGAACCTGAAACATAAAA

A06-pfastbac1-R.ab1(1>809)

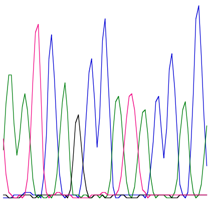

AATCAGCCATACCACA

E02-seq3.ab1(1>802)

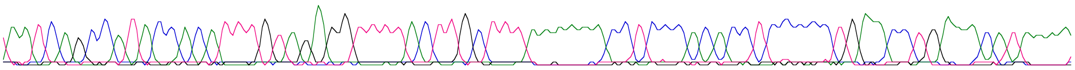

AATCAGCCATACCACATTTGTAGAGGTTTTACTTGCTTTAAAAAACCTCCCACACCTCCCCCTGAACCTGAAACATAAAA

3,130 3,140

TGAATGCAATTGTTGTTGTTAAC

E02-seq3.ab1(1>802)

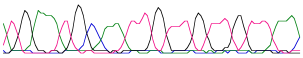

TGAATGCAATTGTTGTTGTTAAC

10 20 30 40 50 60 70 80 90 100

TAAAAAAACCTATAAATATTCCGGATTATTCATACCGTCCCACCATCGGGCGCGGATCCAACTCCTAAAAAAACCGCCACCATGGTGTTTCTGCCCCTCAAATGGTCCC

U4947DH210-2.seq(1>2878) GGATCCAACTCCTAAAAAAACCGCCACCATGGTGTTTCTGCCCCTCAAATGGTCCC

H03-pFastbac-F.ab1(1>822)

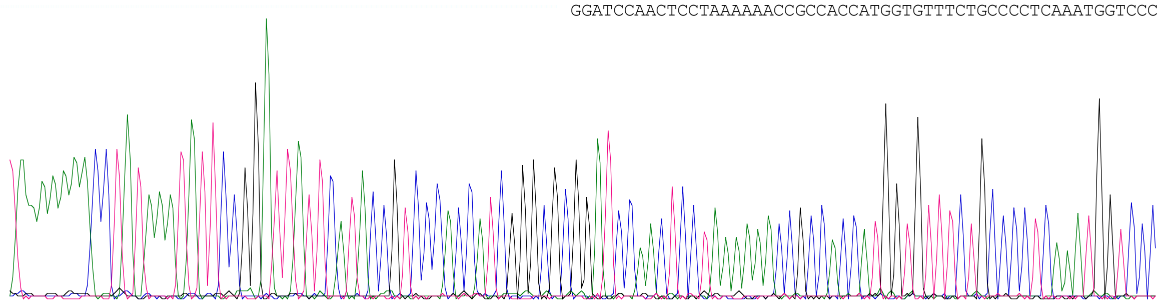

TAAAAAAACCTATAAATATTCCGGATTATTCATACCGTCCCACCATCGGGCGCGGATCCAACTCCTAAAAAAACCGCCACCATGGTGTTTCTGCCCCTCAAATGGTCCC

H05-SEQ3R.ab1(1>794)

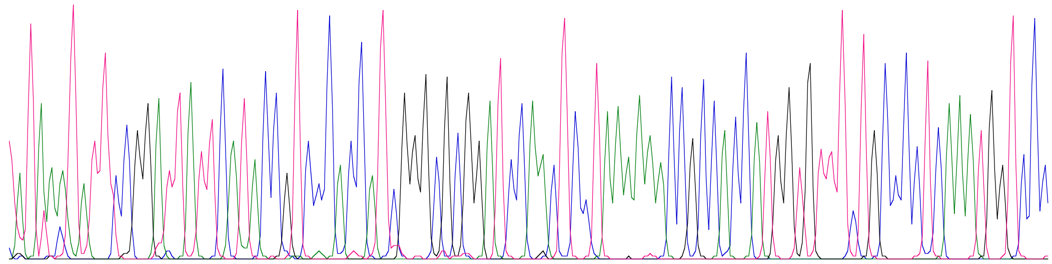

TATAAATATTCCGGATTATTCATACCGTCCCACCATCGGGCGCGGATCCAACTCCTAAAAAAACCGCCACCATGGTGTTTCTGCCCCTCAAATGGTCCC

110 120 130 140 150 160 170 180 190 200 210

TTGCAACCATGTCATTTCTACTTTCCTCACTGTTGGCTCTCTTAACTGTGTCCACTCCTTCATGGTGTCAGAGCACTGAAGCATCTCCAAAACGTAGTGATGGGACAC

U4947DH210-2.seq(1>2878) TTGCAACCATGTCATTTCTACTTTCCTCACTGTTGGCTCTCTTAACTGTGTCCACTCCTTCATGGTGTCAGAGCACTGAAGCATCTCCAAAACGTAGTGATGGGACAC

H03-pFastbac-F.ab1(1>822)

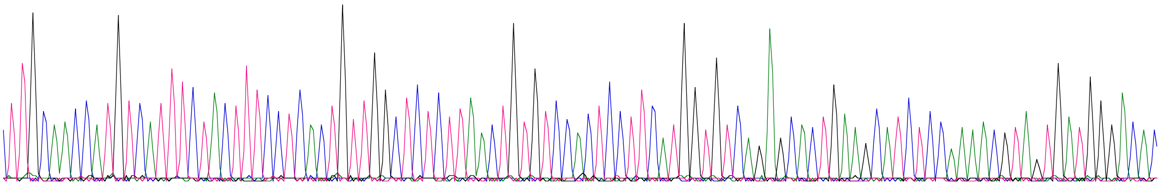

TTGCAACCATGTCATTTCTACTTTCCTCACTGTTGGCTCTCTTAACTGTGTCCACTCCTTCATGGTGTCAGAGCACTGAAGCATCTCCAAAACGTAGTGATGGGACAC

H05-SEQ3R.ab1(1>794)

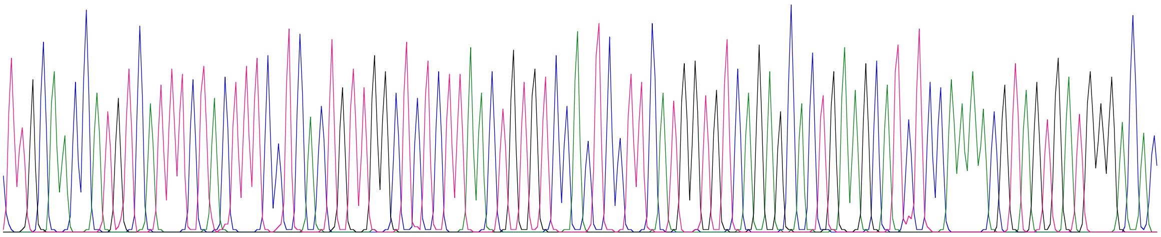

TTGCAACCATGTCATTTCTACTTTCCTCACTGTTGGCTCTCTTAACTGTGTCCACTCCTTCATGGTGTCAGAGCACTGAAGCATCTCCAAAACGTAGTGATGGGACAC

220 230 240 250 260 270 280 290 300 310 320

CATTTCCTTGGAATAAAATACGACTTCCTGAGTACGTCATCCCAGTTCATTATGATCTCTTGATCCATGCAAACCTTACCACGCTGACCTTCTGGGGAACCACGAAAG

U4947DH210-2.seq(1>2878) CATTTCCTTGGAATAAAATACGACTTCCTGAGTACGTCATCCCAGTTCATTATGATCTCTTGATCCATGCAAACCTTACCACGCTGACCTTCTGGGGAACCACGAAAG

H03-pFastbac-F.ab1(1>822)

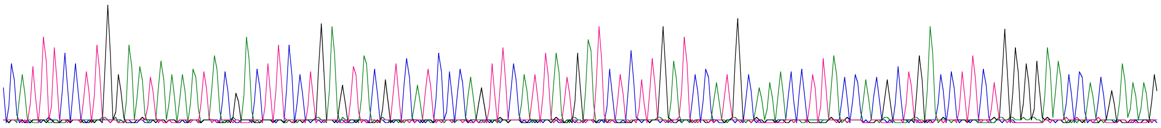

CATTTCCTTGGAATAAAATACGACTTCCTGAGTACGTCATCCCAGTTCATTATGATCTCTTGATCCATGCAAACCTTACCACGCTGACCTTCTGGGGAACCACGAAAG

220 230 240 250 260 270 280 290 300 310 320

CATTTCCTTGGAATAAAATACGACTTCCTGAGTACGTCATCCCAGTTCATTATGATCTCTTGATCCATGCAAACCTTACCACGCTGACCTTCTGGGGAACCACGAAAG

H05-SEQ3R.ab1(1>794)

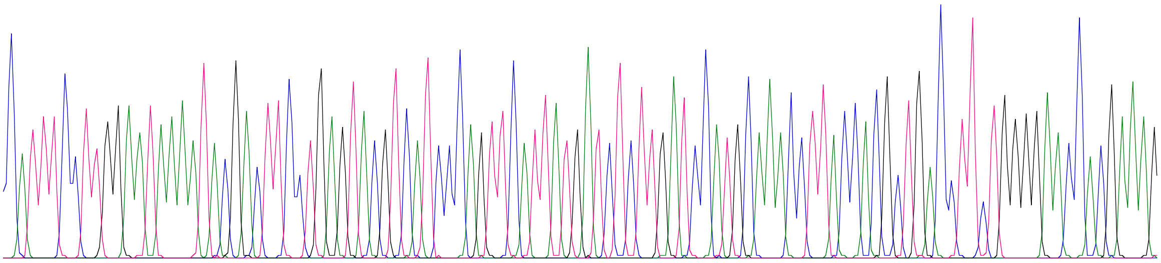

CATTTCCTTGGAATAAAATACGACTTCCTGAGTACGTCATCCCAGTTCATTATGATCTCTTGATCCATGCAAACCTTACCACGCTGACCTTCTGGGGAACCACGAAAG

330 340 350 360 370 380 390 400 410 420 430

TAGAAATCACAGCCAGTCAGCCCACCAGCACCATCATCCTGCATAGTCACCACCTGCAGATATCTAGGGCCACCCTCAGGAAGGGAGCTGGAGAGAGGCTATCGGAAG

U4947DH210-2.seq(1>2878)
TAGAAATCACAGCCAGTCAGCCCACCAGCACCATCATCCTGCATAGTCACCACCTGCAGATATCTAGGGCCACCCTCAGGAAGGGAGCTGGAGAGAGGCTATCGGAAG

H03-pFastbac-F.ab1(1>822)

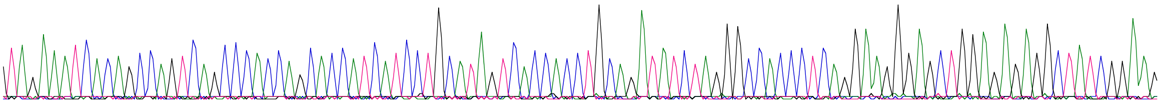

TAGAAATCACAGCCAGTCAGCCCACCAGCACCATCATCCTGCATAGTCACCACCTGCAGATATCTAGGGCCACCCTCAGGAAGGGAGCTGGAGAGAGGCTATCGGAAG

H05-SEQ3R.ab1(1>794)

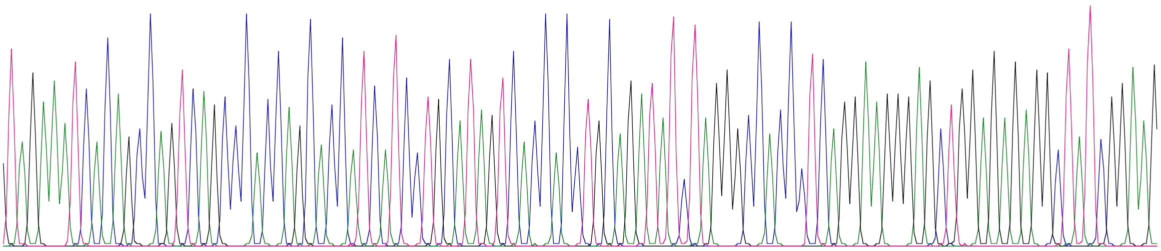

TAGAAATCACAGCCAGTCAGCCCACCAGCACCATCATCCTGCATAGTCACCACCTGCAGATATCTAGGGCCACCCTCAGGAAGGGAGCTGGAGAGAGGCTATCGGAAG

P127R

440 450 460 470 480 490 500 510 520 530 540

AACCCCTGCAGGTCCTGGAACACCCGCGTCAGGAGCAAATTGCACTGCTGGCTCCCGAGCCCCTCCTTGTCGGGCTCCCGTACACAGTTGTCATTCACTATGCTGGCA

U4947DH210-2.seq(1>2878)
AACCCCTGCAGGTCCTGGAACACCCGCGTCAGGAGCAAATTGCACTGCTGGCTCCCGAGCCCCTCCTTGTCGGGCTCCCGTACACAGTTGTCATTCACTATGCTGGCA

H03-pFastbac-F.ab1(1>822)

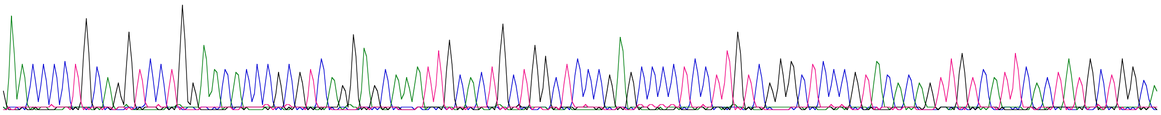

AACCCCTGCAGGTCCTGGAACACCCGCGTCAGGAGCAAATTGCACTGCTGGCTCCCGAGCCCCTCCTTGTCGGGCTCCCGTACACAGTTGTCATTCACTATGCTGGCA

H05-SEQ3R.ab1(1>794)

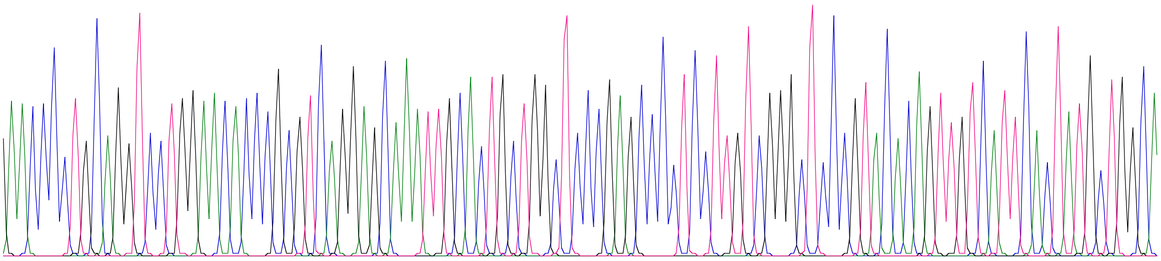

AACCCCTGCAGGTCCTGGAACACCCGCGTCAGGAGCAAATTGCACTGCTGGCTCCCGAGCCCCTCCTTGTCGGGCTCCCGTACACAGTTGTCATTCACTATGCTGGCA

550 560 570 580 590 600 610 620 630 640

ATCTTTCGGAGACTTTCCACGGATTTTACAAAAGCACCTACAGAACCAAGGAAGGGGAACTGAGGATACTAGCATCAACACAATTTGAACCCACTGCAGCTAGAATGG

U4947DH210-2.seq(1>2878) → ATCTTTCGGAGACTTTCCACGGATTTTACAAAAGCACCTACAGAACCAAGGAAGGGGAACTGAGGATACTAGCATCAACACAATTTGAACCCACTGCAGCTAGAATGG

H03-pFastbac-F.ab1(1>822) →

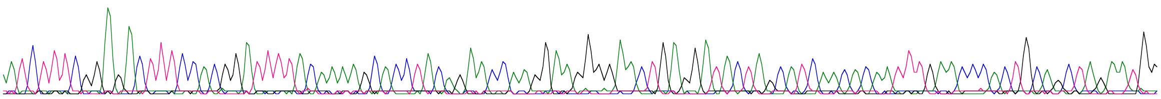

ATCTTTCGGAGACTTTCCACGGATTTTACAAAAGCACCTACAGAACCAAGGAAGGGGAACTGAGGATACTAGCATCAACACAATTTGAACCCACTGCAGCTAGAATGG

H05-SEQ3R.ab1(1>794) ←

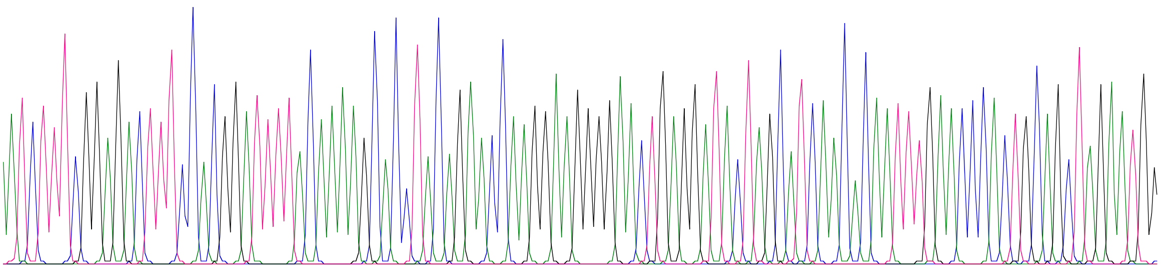

ATCTTTCGGAGACTTTCCACGGATTTTACAAAAGCACCTACAGAACCAAGGAAGGGGAACTGAGGATACTAGCATCAACACAATTTGAACCCACTGCAGCTAGAATGG

650 660 670 680 690 700 710 720 730 740 750

CCTTTCCCTGCTTTGATGAACCTGCCTTCAAAGCAAGTTTCTCAATCAAAATTAGAAGAGAGCCAAGGCACCTAGCCATCTCCAATATGCCATTGGTGAAATCTGTGA

U4947DH210-2.seq(1>2878) → CCTTTCCCTGCTTTGATGAACCTGCCTTCAAAGCAAGTTTCTCAATCAAAATTAGAAGAGAGCCAAGGCACCTAGCCATCTCCAATATGCCATTGGTGAAATCTGTGA

H03-pFastbac-F.ab1(1>822) →

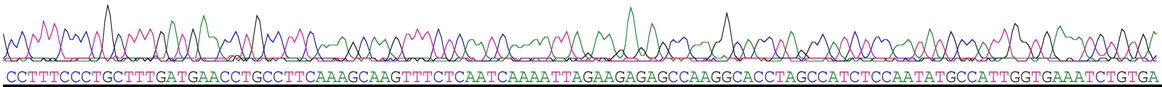

CCTTTCCCTGCTTTGATGAACCTGCCTTCAAAGCAAGTTTCTCAATCAAAATTAGAAGAGAGCCAAGGCACCTAGCCATCTCCAATATGCCATTGGTGAAATCTGTGA

H05-SEQ3R.ab1(1>794) ←

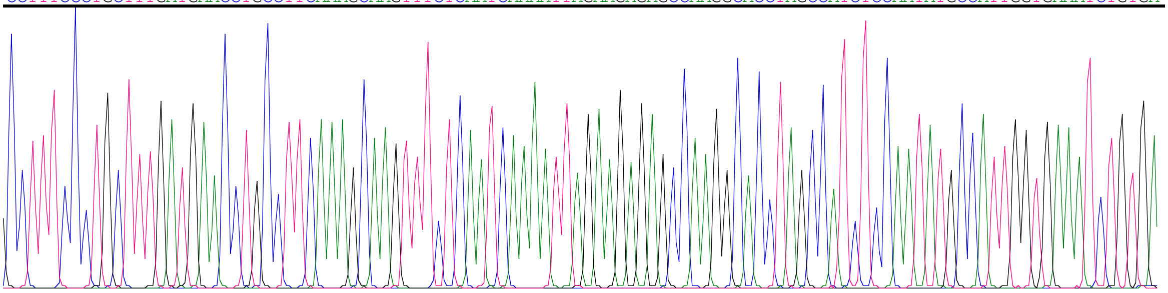

CCTTTCCCTGCTTTGATGAACCTGCCTTCAAAGCAAGTTTCTCAATCAAAATTAGAAGAGAGCCAAGGCACCTAGCCATCTCCAATATGCCATTGGTGAAATCTGTGA

H04-SEQ2R.ab1(1>827) ←

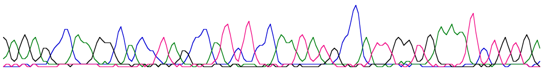

GAGAGCCAAGGCACCTAGCCATCTCCAATATGCCATTGGTGAAATCTGTGA

H05-SEQ1.ab1(1>817) →

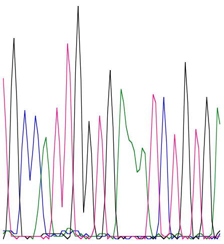

TGCCATTGGTGAAATCTGTGA

760 770 780 790 800 810 820 830 840 850 860

CTGTTGCTGAAGGACTCATAGAAGACCATTTTGATGTCACTGTGAAGATGAGCACCTATCTGGTGGCCTTCATCATTTCAGATTTTGAGTCTGTCAGCAAGATAACCA

U4947DH210-2.seq(1>2878) CTGTTGCTGAAGGACTCATAGAAGACCATTTTGATGTCACTGTGAAGATGAGCACCTATCTGGTGGCCTTCATCATTTCAGATTTTGAGTCTGTCAGCAAGATAACCA
H03-pFastbac-F.ab1(1>822)

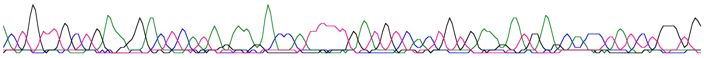

CTGTTGCTGAAGGACTCATAGAAGACCATTTTGATGTCACTGTGAAGATGAGCACCTATCTGGTGG

H05-SEQ3R.ab1(1>794)

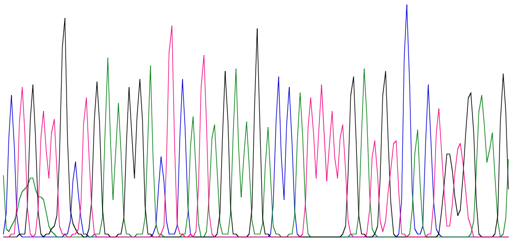

CTGTTGCTGAAGGACTCATAGAAGACCATTTTGATGTCACTGTGAAGA

H04-SEQ2R.ab1(1>827)

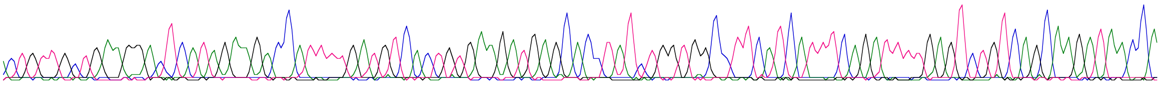

CTGTTGCTGAAGGACTCATAGAAGACCATTTTGATGTCACTGTGAAGATGAGCACCTATCTGGTGGCCTTCATCATTTCAGATTTTGAGTCTGTCAGCAAGATAACCA

H05-SEQ1.ab1(1>817)

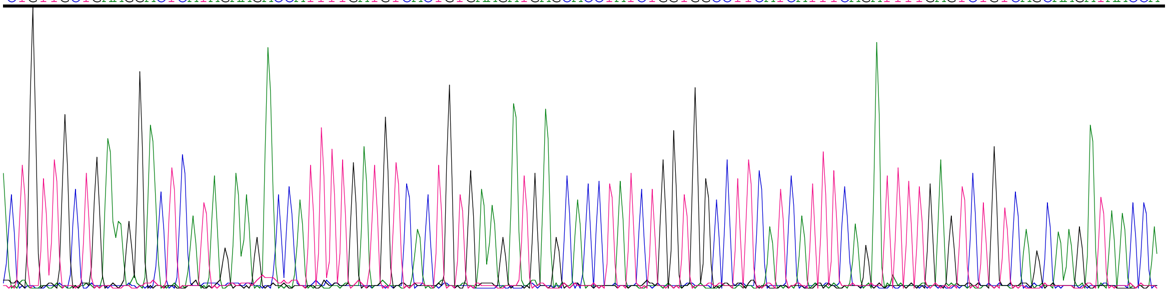

CTGTTGCTGAAGGACTCATAGAAGACCATTTTGATGTCACTGTGAAGATGAGCACCTATCTGGTGGCCTTCATCATTTCAGATTTTGAGTCTGTCAGCAAGATAACCA

870 880 890 900 910 920 930 940 950 960 970

AGAGTGGAGTCAAGGTTTCTGTTTATGCTGTGCCAGACAAGATAAATCAAGCAGATTATGCACTGGATGCTGCGGTGACTCTTCTAGAATTTTATGAGGATTATTTCA

U4947DH210-2.seq(1>2878) AGAGTGGAGTCAAGGTTTCTGTTTATGCTGTGCCAGACAAGATAAATCAAGCAGATTATGCACTGGATGCTGCGGTGACTCTTCTAGAATTTTATGAGGATTATTTCA
H04-SEQ2R.ab1(1>827)

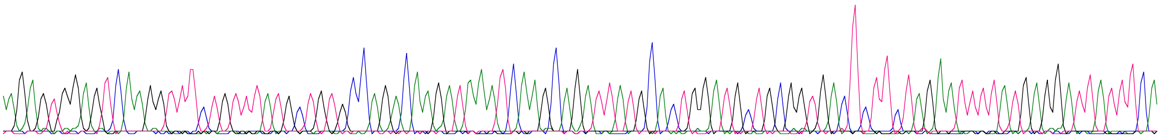

AGAGTGGAGTCAAGGTTTCTGTTTATGCTGTGCCAGACAAGATAAATCAAGCAGATTATGCACTGGATGCTGCGGTGACTCTTCTAGAATTTTATGAGGATTATTTCA

H05-SEQ1.ab1(1>817)

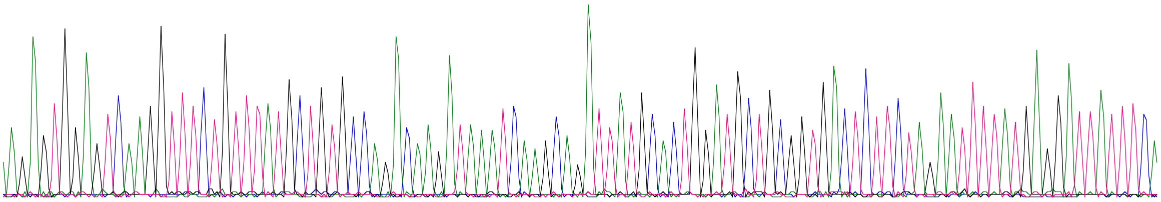

AGAGTGGAGTCAAGGTTTCTGTTTATGCTGTGCCAGACAAGATAAATCAAGCAGATTATGCACTGGATGCTGCGGTGACTCTTCTAGAATTTTATGAGGATTATTTCA

980 990 1,000 1,010 1,020 1,030 1,040 1,050 1,060 1,070 1,080

GCATACCGTATCCCCTACCCAAACAAGATCTTGCTGCTATTCCCGACTTTCAGTCTGGTGCTATGGAAAACTGGGGACTGACAACATATAGAGAATCTGCTCTGTTGT

U4947DH210-2.seq(1>2878) → GCATACCGTATCCCCTACCCAAACAAGATCTTGCTGCTATTCCCGACTTTCAGTCTGGTGCTATGGAAAACTGGGGACTGACAACATATAGAGAATCTGCTCTGTTGT

H04-SEQ2R.ab1(1>827) ←

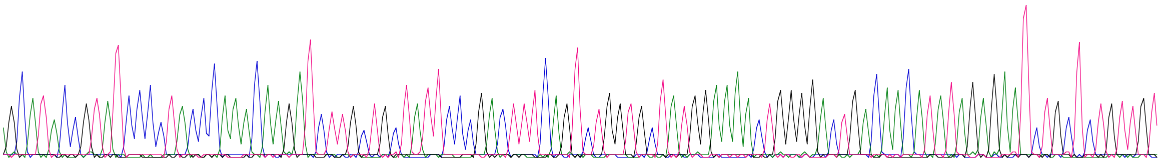

GCATACCGTATCCCCTACCCAAACAAGATCTTGCTGCTATTCCCGACTTTCAGTCTGGTGCTATGGAAAACTGGGGACTGACAACATATAGAGAATCTGCTCTGTTGT

H05-SEQ1.ab1(1>817) →

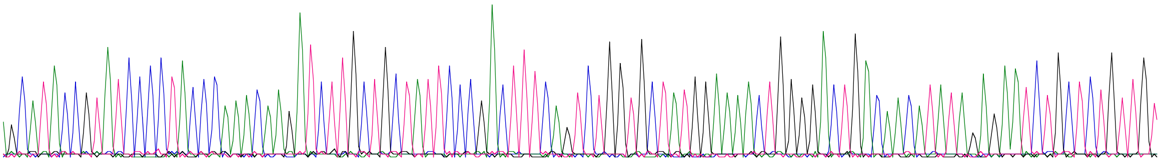

GCATACCGTATCCCCTACCCAAACAAGATCTTGCTGCTATTCCCGACTTTCAGTCTGGTGCTATGGAAAACTGGGGACTGACAACATATAGAGAATCTGCTCTGTTGT

1,090 1,100 1,110 1,120 1,130 1,140 1,150 1,160 1,170 1,180

TTGATGCAGAAAAGTCTTCTGCATCAAGTAAGCTTGGCATCACAATGACTGTGGCCCATGAACTGGCTCACCAGTGGTTTGGGAACCTGGTCACTATGGAATGGTGGA

U4947DH210-2.seq(1>2878) → TTGATGCAGAAAAGTCTTCTGCATCAAGTAAGCTTGGCATCACAATGACTGTGGCCCATGAACTGGCTCACCAGTGGTTTGGGAACCTGGTCACTATGGAATGGTGGA

H04-SEQ2R.ab1(1>827) ←

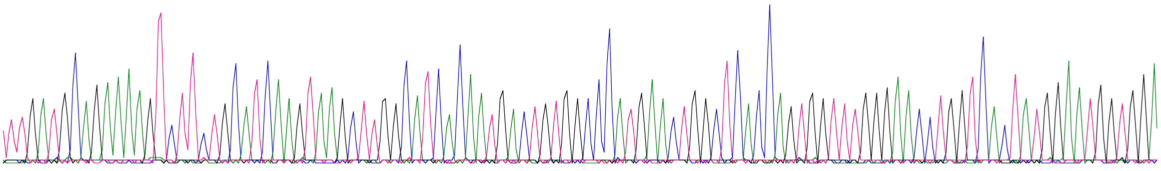

TTGATGCAGAAAAGTCTTCTGCATCAAGTAAGCTTGGCATCACAATGACTGTGGCCCATGAACTGGCTCACCAGTGGTTTGGGAACCTGGTCACTATGGAATGGTGGA

H05-SEQ1.ab1(1>817) →

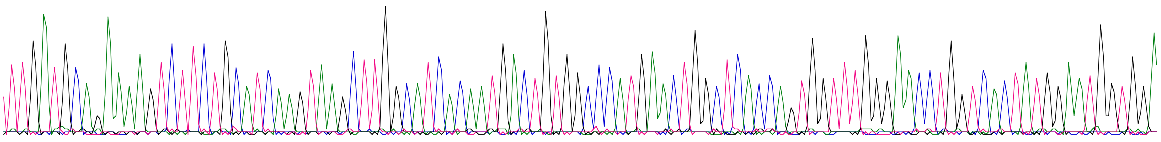

TTGATGCAgAAAAGTCTTCTGCATCAAGTAAGCTTGGCATCACAATGACTGTGGCCCATGAACTGGCTCACCAGTGGTTTGGGAACCTGGTCACTATGGAATGGTGGA

1,190 1,200 1,210 1,220 1,230 1,240 1,250 1,260 1,270 1,280 1,290

ATGATCTTTGGCTAAATGAAGGATTTGCCAAATTTATGGAGTTTGTGTCTGTCAGTGTGACCCATCCTGAACTGAAAGTTGGAGATTATTTCTTTGGCAAATGTTTTG

U4947DH210-2.seq(1>2878) → ATGATCTTTGGCTAAATGAAGGATTTGCCAAATTTATGGAGTTTGTGTCTGTCAGTGTGACCCATCCTGAACTGAAAGTTGGAGATTATTTCTTTGGCAAATGTTTTG

H04-SEQ2R.ab1(1>827) ←

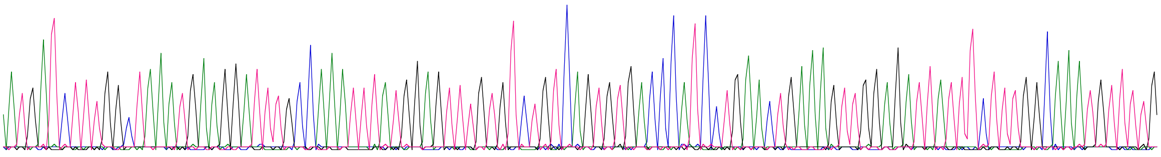

ATGATCTTTGGCTAAATGAAGGATTTGCCAAATTTATGGAGTTTGTGTCTGTCAGTGTGACCCATCCTGAACTGAAAGTTGGAGATTATTTCTTTGGCAAATGTTTTG

1,190 1,200 1,210 1,220 1,230 1,240 1,250 1,260 1,270 1,280 1,290

ATGATCTTTGGCTAAATGAAGGATTTGCCAAATTTATGGAGTTTGTGTCTGTCAGTGTGACCCATCCTGAACTGAAAGTTGGAGATTATTTCTTTGGCAAATGTTTTG

H05-SEQ1.ab1(1>817) →

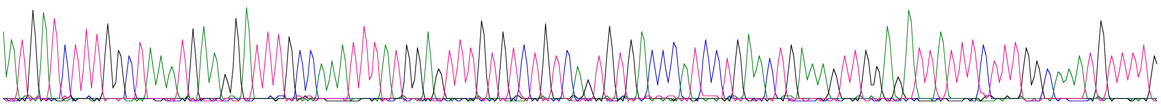

ATGATCTTTGGCTAAATGAAGGATTTGCCAAATTTATGGAGTTTGTGTCTGTCAGTGTGACCCATCCTGAACTGAAAGTTGGAGATTATTTCTTTGGCAAATGTTTTG

1,300 1,310 1,320 1,330 1,340 1,350 1,360 1,370 1,380 1,390 1,400

ACGCAATGGAGGTAGATGCTTTAAATTCCTCACACCCTGTGTCTACACCTGTGGAAAATCCTGCTCAGATCCGGGAGATGTTTGATGATGTTTCTTATGATAAGGGAG

U4947DH210-2.seq(1>2878) → ACGCAATGGAGGTAGATGCTTTAAATTCCTCACACCCTGTGTCTACACCTGTGGAAAATCCTGCTCAGATCCGGGAGATGTTTGATGATGTTTCTTATGATAAGGGAG

H04-SEQ2R.ab1(1>827) ←

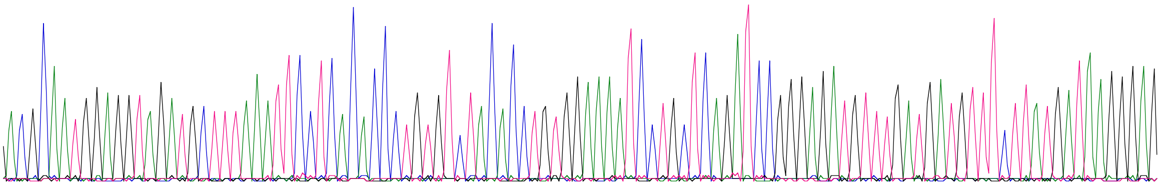

ACGCAATGGAGGTAGATGCTTTAAATTCCTCACACCCTGTGTCTACACCTGTGGAAAATCCTGCTCAGATCCGGGAGATGTTTGATGATGTTTCTTATGATAAGGGAG

H05-SEQ1.ab1(1>817) →

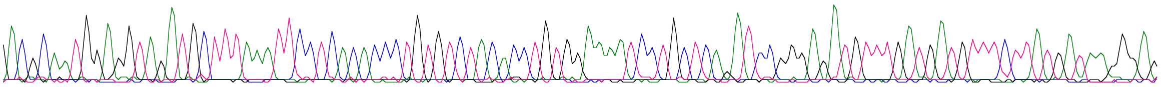

ACGCAATGGAGGTAGATGCTTTAAATTCCTCACACCCTGTGTCTACACCTGTGGAAAATCCTGCTCAGATCCGGGAGATGTTTGATGATGTTTCTTATGATAAGGGAG

1,410 1,420 1,430 1,440 1,450 1,460 1,470 1,480 1,490 1,500 1,510

CTTGTATTCTGAATATGCTAAGGGAGTATCTTAGTGCTGACGCATTTAAAAGTGGTATTGTACAGTATCTCCAGAAGCATAGCTATAAAAATACAAAAAACGAGGACC

U4947DH210-2.seq(1>2878) → CTTGTATTCTGAATATGCTAAGGGAGTATCTTAGTGCTGACGCATTTAAAAGTGGTATTGTACAGTATCTCCAGAAGCATAGCTATAAAAATACAAAAAACGAGGACC

H04-SEQ2R.ab1(1>827) ←

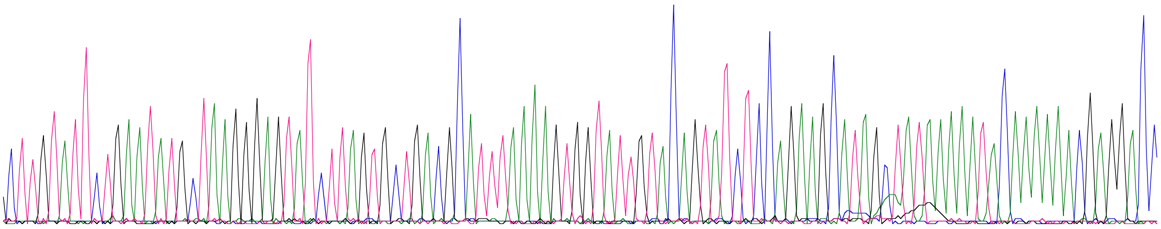

CTTGTATTCTGAATATGCTAAGGGAGTATCTTAGTGCTGACGCATTTAAAAGTGGTATTGTACAGTATCTCCAGAAGCATAGctATAAAAATACAAAAAACGAGGACC

H05-SEQ1.ab1(1>817) →

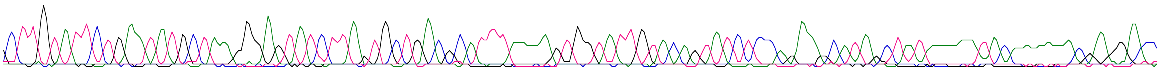

CTTGTATTCTGAATATGCTAAGGGAGTATCTTAGTGCTGACGCATTTAAAAGTGGTATTGTACAGTATCTCCAGAAGCATAGCTATAAAAATACAAAAAACGAGGACC

1,410 1,420 1,430 1,440 1,450 1,460 1,470 1,480 1,490 1,500 1,510

CTTGTATTCTGAATATGCTAAGGGAGTATCTTAGTGCTGACGCATTTAAAAGTGGTATTGTACAGTATCTCCAGAAGCATAGCTATAAAAATACAAAAAACGAGGACC

B11-SEQ1R.ab1(1>824) ←

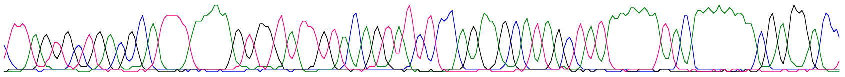

CTTAGTGCTGACGCATTTAAAAGTGGTATTGTACAGTATCTCCAGAAGCATAGCTATAAAAATACAAAAAACGAGGACC

F12-SEQ2.ab1(1>819) →

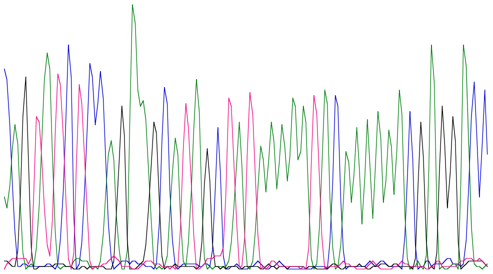

CAGTATCTCCAGAAGCATAGCTATAAAAATACAAAAAACGAGGACC

1,520 1,530 1,540 1,550 1,560 1,570 1,580 1,590 1,600 1,610 1,620

TGTGGGATAGTATGGCAAGTATTTGCCCTACAGATGGTGTAAAAGGGATGGATGGCTTTTGCTCTAGAAGTCAACATTCATCTTCATCCTCACATTGGCATCAGGAAG

U4947DH210-2.seq(1>2878) → TGTGGGATAGTATGGCAAGTATTTGCCCTACAGATGGTGTAAAAGGGATGGATGGCTTTTGCTCTAGAAGTCAACATTCATCTTCATCCTCACATTGGCATCAGGAAG

H04-SEQ2R.ab1(1>827) ←

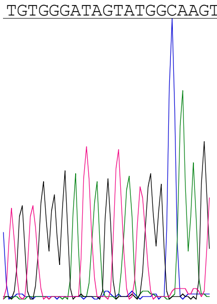

TGTGGGATAGTATGGCAAGT

H05-SEQ1.ab1(1>817) →

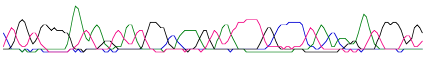

TGTGGGATAGTATGGCAAGTATTTGCCCTACAGATGGTGT

B11-SEQ1R.ab1(1>824) ←

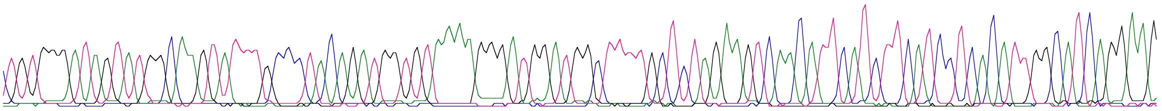

TGTGGGATAGTATGGCAAGTATTTGCCCTACAGATGGTGTAAAAGGGATGGATGGCTTTTGCTCTAGAAGTCAACATTCATCTTCATCCTCACATTGGCATCAGGAAG

F12-SEQ2.ab1(1>819) →

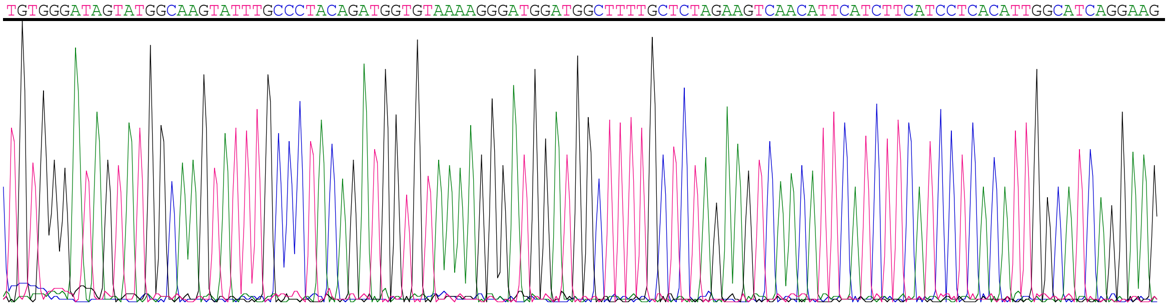

TGTGGGATAGTATGGCAAGTATTTGCCCTACAGATGGTGTAAAAGGGATGGATGGCTTTTGCTCTAGAAGTCAACATTCATCTTCATCCTCACATTGGCATCAGGAAG

1,630 1,640 1,650 1,660 1,670 1,680 1,690 1,700 1,710 1,720

GGGTGGATGTGAAAACCATGATGAACACTTGGACACTGCAGAAGGGTTTTCCCCTAATAACCATCACAGTGAGGGGGAGGAATGTACACATGAAGCAAGAGCACTACA

U4947DH210-2.seq(1>2878) → GGGTGGATGTGAAAACCATGATGAACACTTGGACACTGCAGAAGGGTTTTCCCCTAATAACCATCACAGTGAGGGGGAGGAATGTACACATGAAGCAAGAGCACTACA
B11-SEQ1R.ab1(1>824) ←

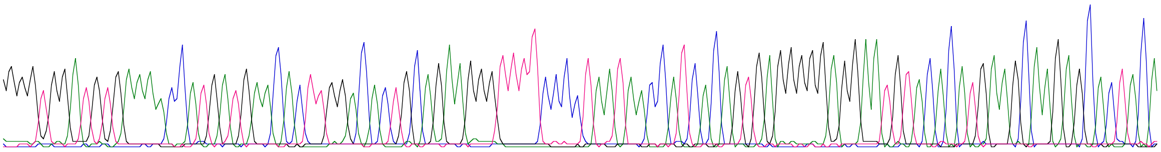

GGGTGGATGTGAAAACCATGATGAACACTTGGACACTGCAGAAGGGTTTTCCCCTAATAACCATCACAGTGAGGGGGAGGAATGTACACATGAAGCAAGAGCACTACA

F12-SEQ2.ab1(1>819) →

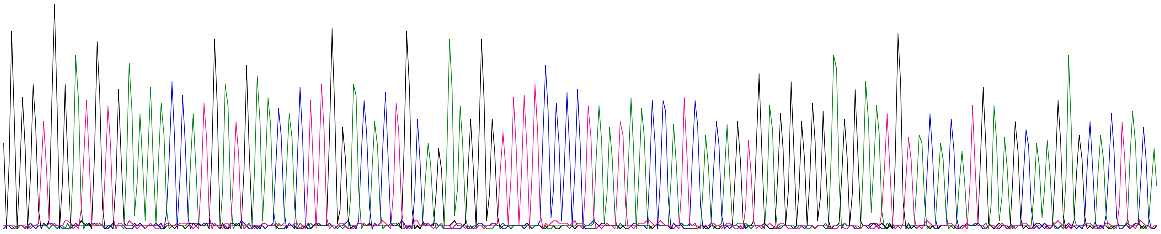

GGGTGGATGTGAAAACCATGATGAACACTTGGACACTGCAGAAGGGTTTTCCCCTAATAACCATCACAGTGAGGGGGAGGAATGTACACATGAAGCAAGAGCACTACA

1,730 1,740 1,750 1,760 1,770 1,780 1,790 1,800 1,810 1,820 1,830

TGAAGGGCTCTGACGGCGCCCCGGACACTGGGTACCTGTGGCATGTTCCATTGACATTCATCACCAGCAAATCCGACATGGTCCATCGATTTTTGCTAAAAACAAAAA

U4947DH210-2.seq(1>2878) → TGAAGGGCTCTGACGGCGCCCCGGACACTGGGTACCTGTGGCATGTTCCATTGACATTCATCACCAGCAAATCCGACATGGTCCATCGATTTTTGCTAAAAACAAAAA
B11-SEQ1R.ab1(1>824) ←

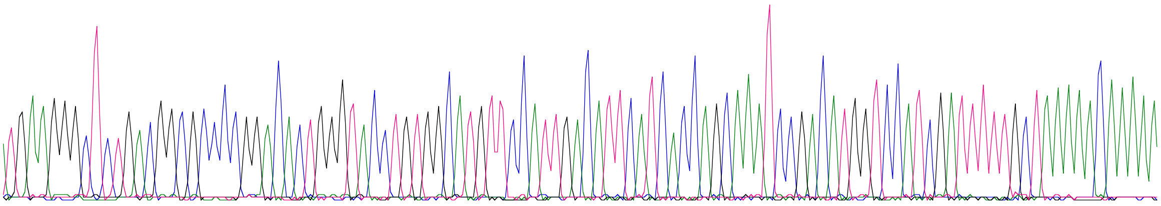

TGAAGGGCTCTGACGGCGCCCCGGACACTGGGTACCTGTGGCATGTTCCATTGACATTCATCACCAGCAAATCCGACATGGTCCATCGATTTTTGCTAAAAACAAAAA

F12-SEQ2.ab1(1>819) →

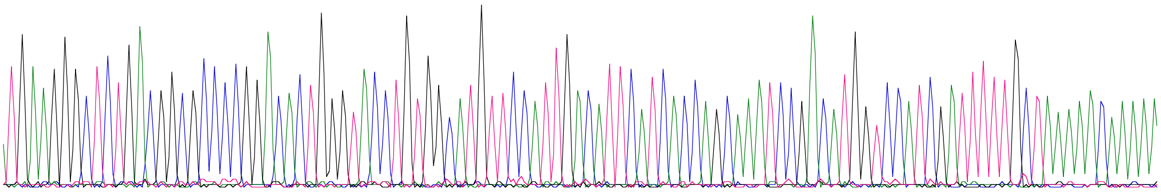

TGAAGGGCTCTGACGGCGCCCCGGACACTGGGTACCTGTGGCATGTTCCATTGACATTCATCACCAGCAAATCCGACATGGTCCATCGATTTTTGCTAAAAACAAAAA

1,840 1,850 1,860 1,870 1,880 1,890 1,900 1,910 1,920 1,930 1,940

CAGATGTGCTCATCCTCCCAGAAGAGGTGGAATGGATCAAATTTAATGTGGGCATGAATGGCTATTACATTGTGCATTACGAGGATGATGGATGGGACTCTTTGACTG

U4947DH210-2.seq(1>2878) → CAGATGTGCTCATCCTCCCAGAAGAGGTGGAATGGATCAAATTTAATGTGGGCATGAATGGCTATTACATTGTGCATTACGAGGATGATGGATGGGACTCTTTGACTG
B11-SEQ1R.ab1(1>824) ←

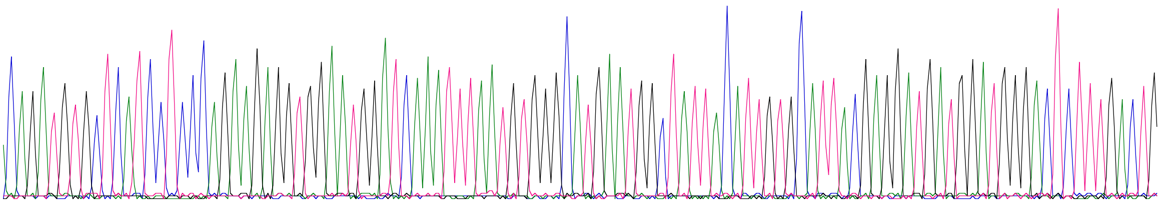

CAGATGTGCTCATCCTCCCAGAAGAGGTGGAATGGATCAAATTTAATGTGGGCATGAATGGCTATTACATTGTGCATTACGAGGATGATGGATGGGACTCTTTGACTG

1,840 1,850 1,860 1,870 1,880 1,890 1,900 1,910 1,920 1,930 1,940

CAGATGTGCTCATCCTCCCAGAAGAGGTGGAATGGATCAAATTTAATGTGGGCATGAATGGCTATTACATTGTGCATTACGAGGATGATGGATGGGACTCTTTGACTG

F12-SEQ2.ab1(1>819)

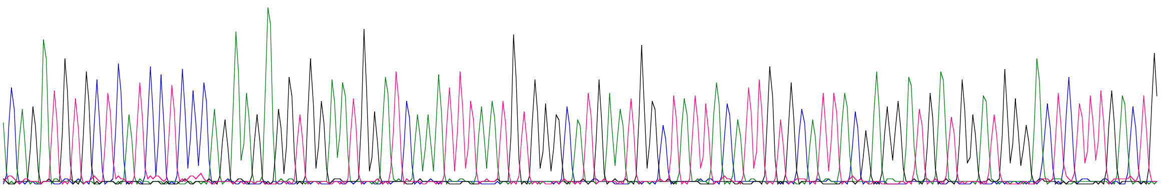

CAGATGTGCTCATCCTCCCAGAAGAGGTGGAATGGATCAAATTTAATGTGGGCATGAATGGCTATTACATTGTGCATTACGAGGATGATGGATGGGACTCTTTGACTG

1,950 1,960 1,970 1,980 1,990 2,000 2,010 2,020 2,030 2,040 2,050

GCCTTTTAAAAGGAACACACACAGCAGTCAGCAGTAATGATCGGGCGAGTCTCATTAACAATGCATTTCAGCTCGTCAGCATTGGGAAGCTGTCCATTGAAAAGGCCT

U4947DH210-2.seq(1>2878) GCCTTTTAAAAGGAACACACACAGCAGTCAGCAGTAATGATCGGGCGAGTCTCATTAACAATGCATTTCAGCTCGTCAGCATTGGGAAGCTGTCCATTGAAAAGGCCT

B11-SEQ1R.ab1(1>824)

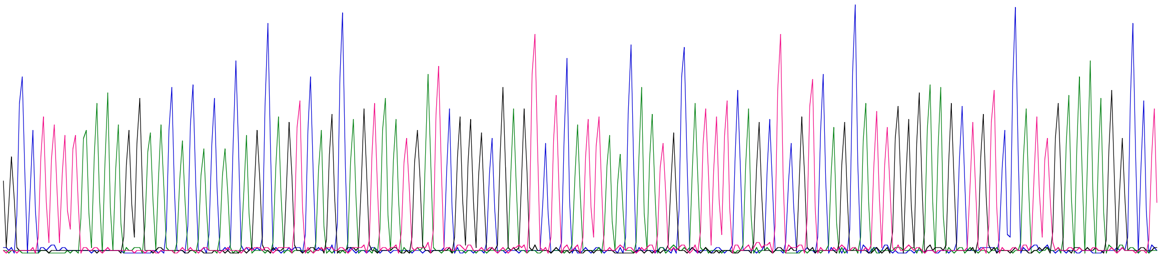

GCCTTTTAAAAGGAACACACACAGCAGTCAGCAGTAATGATCGGGCGAGTCTCATTAACAATGCATTTCAGCTCGTCAGCATTGGGAAGCTGTCCATTGAAAAGGCCT

F12-SEQ2.ab1(1>819)

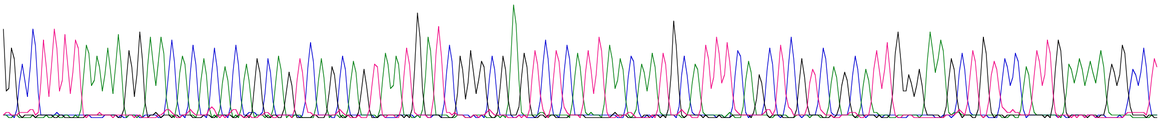

GCCTTTTAAAAGGAACACACACAGCAGTCAGCAGTAATGATCGGGCGAGTCTCATTAACAATGCATTTCAGCTCGTCAGCATTGGGAAGCTGTCCATTGAAAAGGCCT

2,060 2,070 2,080 2,090 2,100 2,110 2,120 2,130 2,140 2,150 2,160

TGGATTTATCCCTGTACTTGAAACATGAAACTGAAATTATGCCCGTGTTTCAAGGTTTGAATGAGCTGATTCCTATGTATAAGTTAATGGAGAAAAGAGATATGAATG

U4947DH210-2.seq(1>2878) TGGATTTATCCCTGTACTTGAAACATGAAACTGAAATTATGCCCGTGTTTCAAGGTTTGAATGAGCTGATTCCTATGTATAAGTTAATGGAGAAAAGAGATATGAATG

B11-SEQ1R.ab1(1>824)

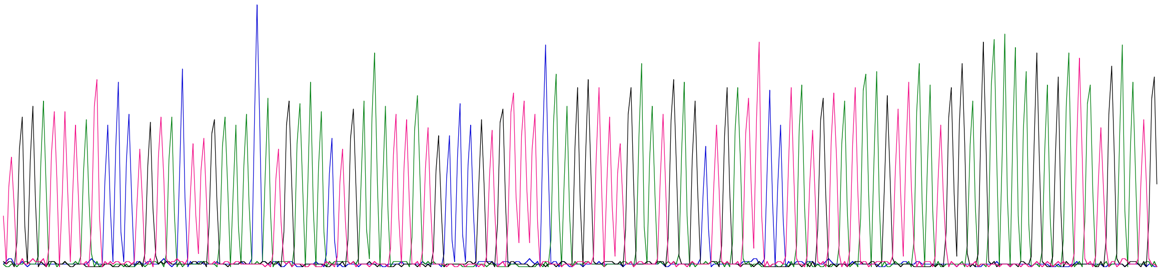

TGGATTTATCCCTGTACTTGAAACATGAAACTGAAATTATGCCCGTGTTTCAAGGTTTGAATGAGCTGATTCCTATGTATAAGTTAATGGAGAAAAGAGATATGAATG

F12-SEQ2.ab1(1>819)

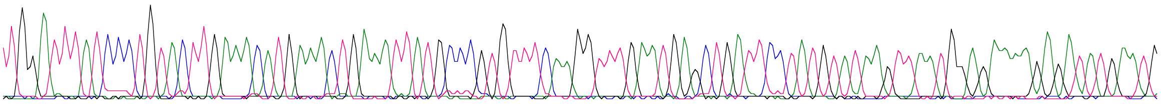

TGGATTTATCCCTGTACTTGAAACATGAAACTGAAATTATGCCCGTGTTTCAAGGTTTGAATGAGCTGATTCCTATGTATAAGTTAATGGAGAAAAGAGATATGAATG

2,170 2,180 2,190 2,200 2,210 2,220 2,230 2,240 2,250 2,260

AAGTGGAAACTCAATTCAAGGCCTTCCTCATCAGGCTGCTAAGGGACCTCATTGATAAGCAGACATGGACAGACGAGGGCTCAGTCTCAGAGCGAATGCTGCGGAGTG

U4947DH210-2.seq(1>2878) AAGTGGAAACTCAATTCAAGGCCTTCCTCATCAGGCTGCTAAGGGACCTCATTGATAAGCAGACATGGACAGACGAGGGCTCAGTCTCAGAGCGAATGCTGCGGAGTG

B11-SEQ1R.ab1(1>824)

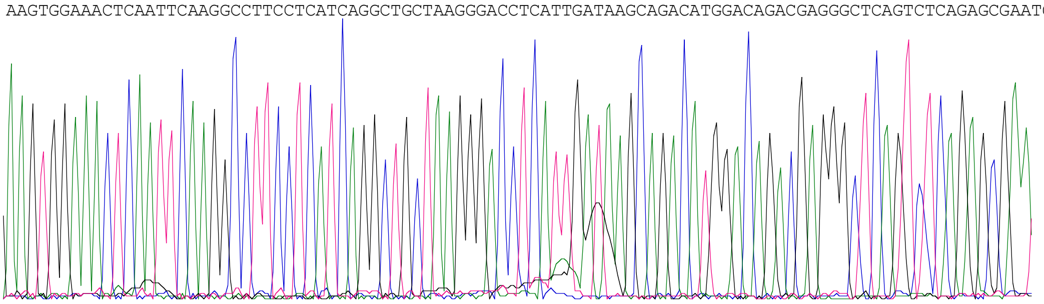

AAGTGGAAACTCAATTCAAGGCCTTCCTCATCAGGCTGCTAAGGGACCTCATTGATAAGCAGACATGGACAGACGAGGGCTCAGTCTCAGAGCGAAT

F12-SEQ2.ab1(1>819)

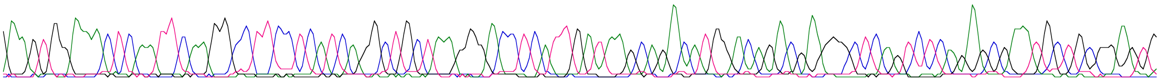

AAGTGGAAACTCAATTCAAGGCCTTCCTCATCAGGCTGCTAAGGGACCTCATTGATAAGCAGACATGGACAGACGAGGGCTCAGTCTCAGAGCGAATGCTGCGGAGTG

H04-pFastbac-R.ab1(1>825)

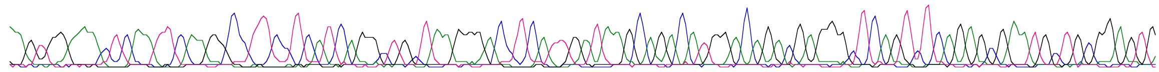

AAGTGGAAACTCAATTCAAGGCCTTCCTCATCAGGCTGCTAAGGGACCTCATTGATAAGCAGACATGGACAGACGAGGGCTCAGTCTCAGAGCGAATGCTGCGGAGTG

H07-SEQ3.ab1(1>827)

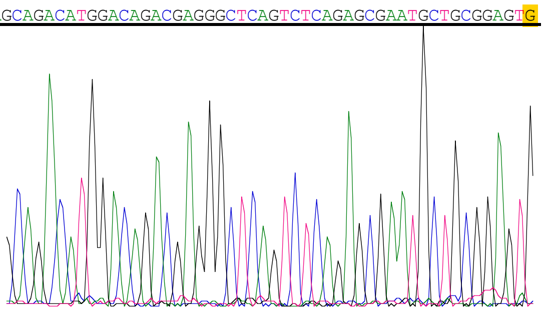

GCAGACATGGACAGACGAGGGCTCAGTCTCAGAGCGAATGCTGCGGAGTG

Q730E

2,270 2,280 2,290 2,300 2,310 2,320 2,330 2,340 2,350 2,360 2,370

AGCTACTACTCCTCGCCTGTGTGCACAACTATCAGCCGTGCGTACAGAGGGCAGAAGGCTATTTCAGAAAGTGGAAGGAATCCAATGGAAACTTGAGCCTGCCTGTCG

U4947DH210-2.seq(1>2878) AGCTACTACTCCTCGCCTGTGTGCACAACTATCAGCCGTGCGTACAGAGGGCAGAAGGCTATTTCAGAAAGTGGAAGGAATCCAATGGAAACTTGAGCCTGCCTGTCG

F12-SEQ2.ab1(1>819)

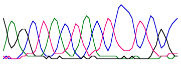

AGCTACTACTCCTCGCC

H04-pFastbac-R.ab1(1>825)

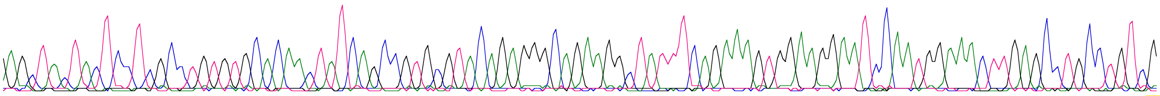

AGCTACTACTCCTCGCCTGTGTGCACAACTATCAGCCGTGCGTACAGAGGGCAGAAGGCTATTTCAGAAAGTGGAAGGAATCCAATGGAAACTTGAGCCTGCCTGTCG

Q730E

2,270 2,280 2,290 2,300 2,310 2,320 2,330 2,340 2,350 2,360 2,370

AGCTACTACTCCTCGCCTGTGTGCACAACTATCAGCCGTGCGTACAGAGGGCAGAAGGCTATTTCAGAAAGTGGAAGGAATCCAATGGAAACTTGAGCCTGCCTGTCG

H07-SEQ3.ab1(1>827)

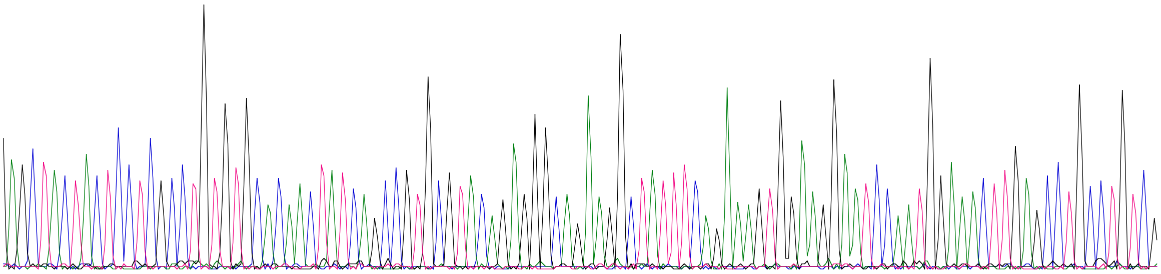

AGCTACTACTCCTCGCCTGTGTGCACAACTATCAGCCGTGCGTACAGAGGGCAGAAGGCTATTTCAGAAAGTGGAAGGAATCCAATGGAAACTTGAGCCTGCCTGTCG

2,380 2,390 2,400 2,410 2,420 2,430 2,440 2,450 2,460 2,470 2,480

ACGTGACCTTGGCAGTGTTTGCTGTGGGGGCCCAGAGCACAGAAGGCTGGGATTTTCTTTATAGTAAATATCAGTTTTCTTTGTCCAGTACTGAGAAAAGCCAAATTG

U4947DH210-2.seq(1>2878) ACGTGACCTTGGCAGTGTTTGCTGTGGGGGCCCAGAGCACAGAAGGCTGGGATTTTCTTTATAGTAAATATCAGTTTTCTTTGTCCAGTACTGAGAAAAGCCAAATTG
H04-pFastbac-R.ab1(1>825)

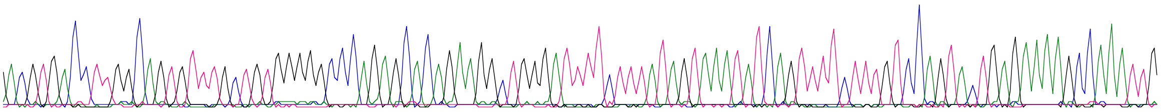

ACGTGACCTTGGCAGTGTTTGCTGTGGGGGCCCAGAGCACAGAAGGCTGGGATTTTCTTTATAGTAAATATCAGTTTTCTTTGTCCAGTACTGAGAAAAGCCAAATTG

H07-SEQ3.ab1(1>827)

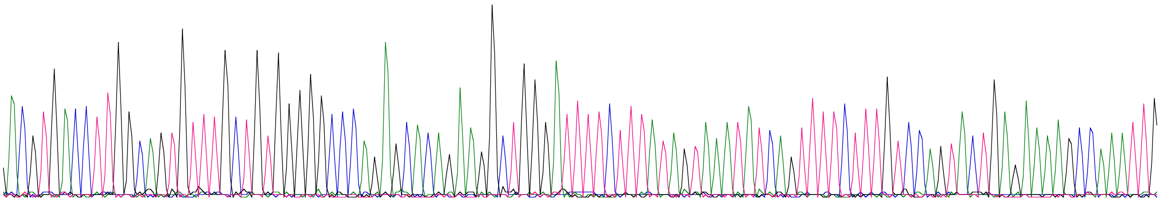

ACGTGACCTTGGCAGTGTTTGCTGTGGGGGCCCaGAGCACAGAAGGCTGGGATTTTCTTTATAGTAAATATCAGTTTTCTTTGTCCAGTACTGAGAAAAGCCAAATTG

2,490 2,500 2,510 2,520 2,530 2,540 2,550 2,560 2,570 2,580 2,590

AATTTGCCCTCTGCAGAACCCAAAATAAGGAAAAGCTTCAATGGCTACTAGATGAAAGCTTTAAGGGAGATAAAATAAAAACTCAGGAGTTTCCACAAATTCTTACAC

U4947DH210-2.seq(1>2878) AATTTGCCCTCTGCAGAACCCAAAATAAGGAAAAGCTTCAATGGCTACTAGATGAAAGCTTTAAGGGAGATAAAATAAAAACTCAGGAGTTTCCACAAATTCTTACAC
H04-pFastbac-R.ab1(1>825)

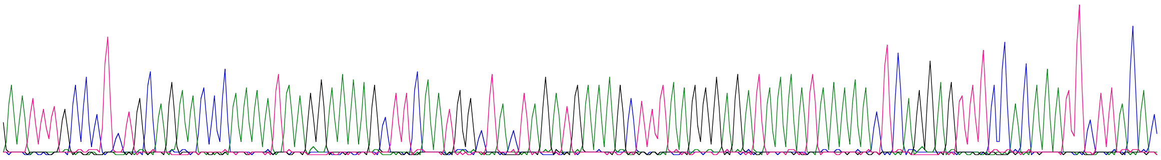

AATTTGCCCTCTGCAGAACCCAAAATAAGGAAAAGCTTCAATGGCTACTAGATGAAAGCTTTAAGGGAGATAAAATAAAAACTCAGGAGTTTCCACAAATTCTTACAC

H07-SEQ3.ab1(1>827)

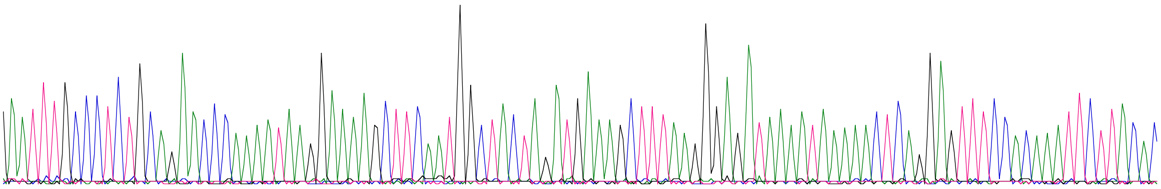

AATTTGCCCTCTGCAGAACCCAAAATAAGGAAAAGCTTCAATGGCTACTAGATGAAAGCTTTAAGGGAGATAAAATAAAAACTCAGGAGTTTCCACAAATTCTTACAC

2,600 2,610 2,620 2,630 2,640 2,650 2,660 2,670 2,680 2,690 2,700

TCATTGGCAGGAACCCAGTAGGATACCCACTGGCCTGGCAATTTCTGAGGAAAAACTGGAACAAACTTGTACAAAAGTTTGAACTTGGCTCATCTTCCATAGCCCACA

U4947DH210-2.seq(1>2878) → TCATTGGCAGGAACCCAGTAGGATACCCACTGGCCTGGCAATTTCTGAGGAAAAACTGGAACAAACTTGTACAAAAGTTTGAACTTGGCTCATCTTCCATAGCCCACA
H04-pFastbac-R.ab1(1>825) ←

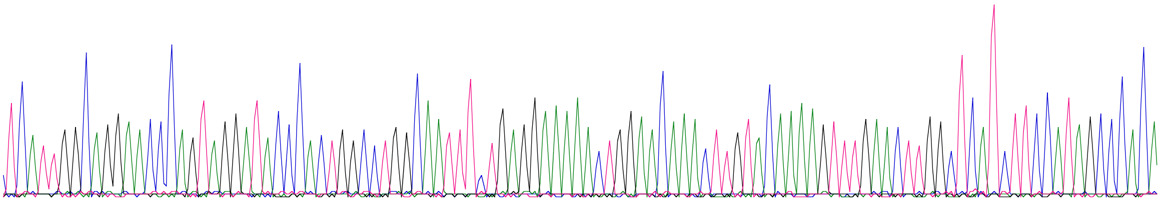

TCATTGGCAGGAACCCAGTAGGATACCCACTGGCCTGGCAATTTCTGAGGAAAAACTGGAACAAACTTGTACAAAAGTTTGAACTTGGCTCATCTTCCATAGCCCACA

H07-SEQ3.ab1(1>827) →

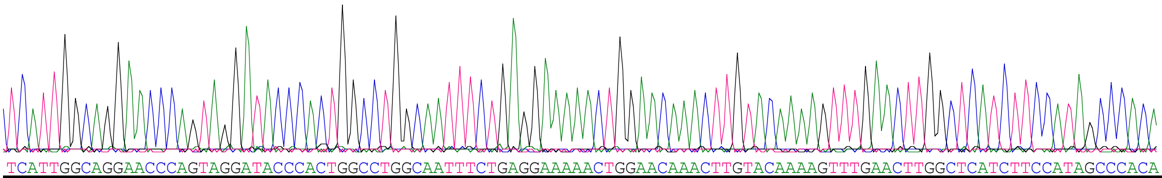

TCATTGGCAGGAACCCAGTAGGATACCCACTGGCCTGGCAATTTCTGAGGAAAAACTGGAACAAACTTGTACAAAAGTTTGAACTTGGCTCATCTTCCATAGCCCACA

2,710 2,720 2,730 2,740 2,750 2,760 2,770 2,780 2,790 2,800

TGGTAATGGGTACAACAAATCAATTCTCCACAAGAACACGGCTTGAAGAGGTAAAAGGATTCTTCAGCTCTTTGAAAGAAAATGGTTCTCAGCTCCGTTGTGTCCAAC

U4947DH210-2.seq(1>2878) → TGGTAATGGGTACAACAAATCAATTCTCCACAAGAACACGGCTTGAAGAGGTAAAAGGATTCTTCAGCTCTTTGAAAGAAAATGGTTCTCAGCTCCGTTGTGTCCAAC
H04-pFastbac-R.ab1(1>825) ←

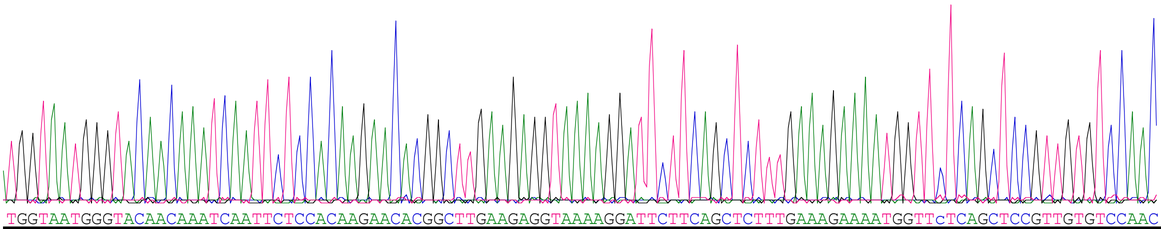

TGGTAATGGGTACAACAAATCAATTCTCCACAAGAACACGGCTTGAAGAGGTAAAAGGATTCTTCAGCTCTTTGAAAGAAAATGGTTcTCAGCTCCGTTGTGTCCAAC

H07-SEQ3.ab1(1>827) →

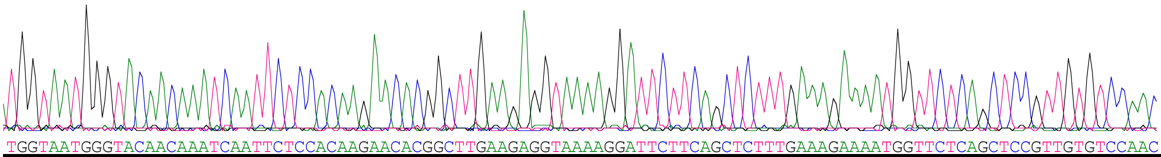

TGGTAATGGGTACAACAAATCAATTCTCCACAAGAACACGGCTTGAAGAGGTAAAAGGATTCTTCAGCTCTTTGAAAGAAAATGGTTCTCAGCTCCGTTGTGTCCAAC

2,810 2,820 2,830 2,840 2,850 2,860 2,870 2,880 2,890 2,900 2,910

AGACAATTGAAACCATTGAAGAAAACATCGGTTGGATGGATAAGAATTTTGATAAAATCAGAGTGTGGCTGCAAAGTGAAAAGCTTGAACGCATGCATCATCACCATC

U4947DH210-2.seq(1>2878) → AGACAATTGAAACCATTGAAGAAAACATCGGTTGGATGGATAAGAATTTTGATAAAATCAGAGTGTGGCTGCAAAGTGAAAAGCTTGAACGCATGCATCATCACCATC
H04-pFastbac-R.ab1(1>825) ←

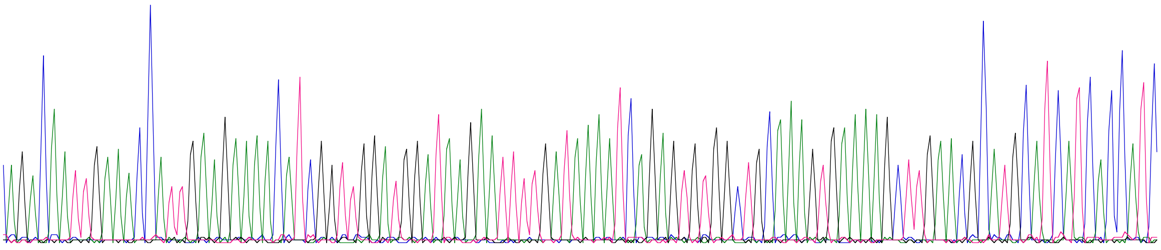

AGACAATTGAAACCATTGAAGAAAACATCGGTTGGATGGATAAGAATTTTGATAAAATCAGAGTGTGGCTGCAAAGTGAAAAGCTTGAACGCATGCATCATCACCATC

2,810 2,820 2,830 2,840 2,850 2,860 2,870 2,880 2,890 2,900 2,910

AGACAATTGAAACCATTGAAGAAAACATCGGTTGGATGGATAAGAATTTTGATAAAATCAGAGTGTGGCTGCAAAGTGAAAAGCTTGAACGCATGCATCATCACCATC

H07-SEQ3.ab1(1>827)

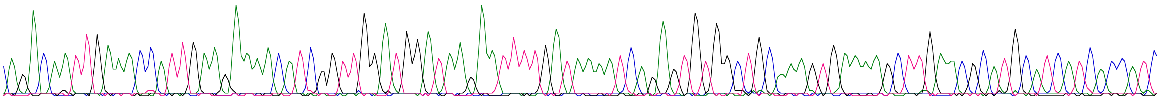

AGACAATTGAAACCATTGAAGAAAACATCGGTTGGATGGATAAGAATTTTGATAAAATCAGAGTGTGGCTGCAAAGTGAAAAGCTTGAACGCATGCATCATCACCATC

2,920 2,930 2,940 2,950 2,960 2,970 2,980 2,990 3,000 3,010 3,020

ACCATTAACTCGAGGCATGCGGTACCAAGCTTGTCGAGAAGTACTAGAGGATCATAATCAGCCATACCACATTTGTAGAGGTTTTACTTGCTTTAAAAAACCTCCCAC

U4947DH210-2.seq(1>2878)
H04-pFastbac-R.ab1(1>825)

ACCATTAACTCGAG

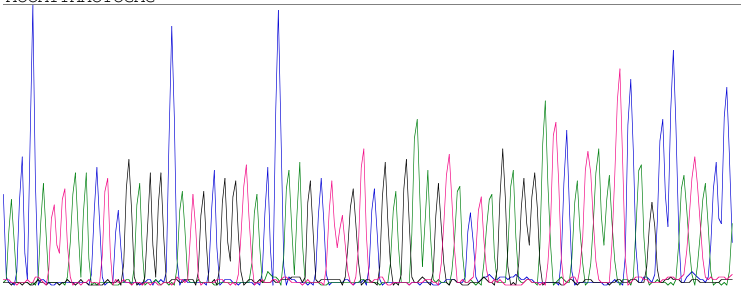

ACCATTAACTCGAGGCATGCGGTACCAAGCTTGTCGAGAAGTACTAGAGGATCATAATCAGCCATACCA

H07-SEQ3.ab1(1>827)

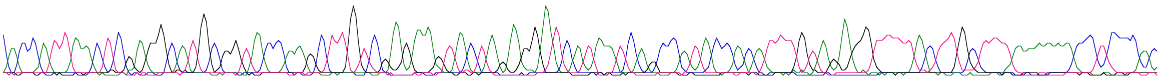

ACCATTAACTCGAGGCATGCGGTACCAAGCTTGTCGAGAAGTACTAGAGGATCATAATCAGCCATACCACATTTGTAGAGGTTTTACTTGCTTTAAAAAACCTCCCAC

3,030 3,040

ACCTCCCCCTGAACCTGAAAC

H07-SEQ3.ab1(1>827)

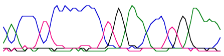

ACCTCCCCCTGAACCTGAAAC

Haplotype 4

10 20 30 40 50 60 70 80 90 100

TTTTGTAATAAAAAAAACCTATAAATATTCCGGATTATTCATACCGTCCCACCATCGGGCGCGGATCCAACTCCTAAAAAAACCGCCACCATGGTGTTTCTGCCCCTCAA

U4947DH210-4.seq(1>2878) GGATCCAACTCCTAAAAAAACCGCCACCATGGTGTTTCTGCCCCTCAA

G01-pFastbac-F.ab1(1>821)

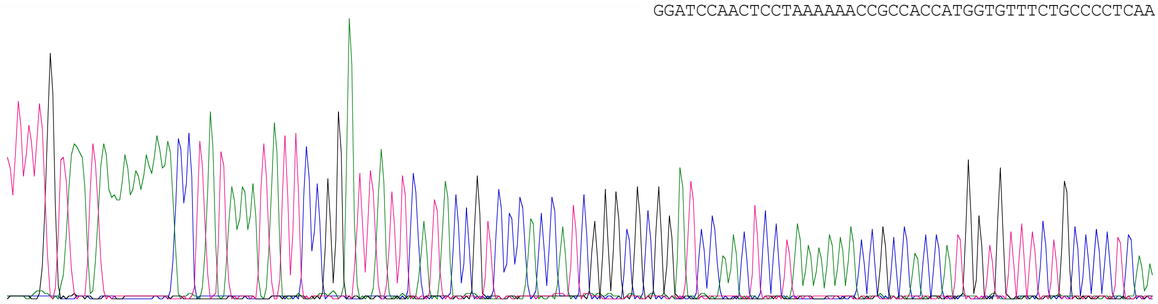

TTTTGTaATAAAAAAAACCTATAAATATTCCGGATTATTCATACCGTCCCACCATCGGGCGCGGATCCAACTCCTAAAAAAACCGCCACCATGGTGTTTCTGCCCCTCAA

F11-SEQ3R.ab1(1>811)

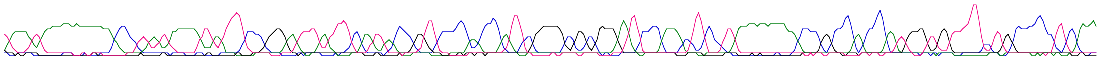

TAATAAAAAAAACCTATAAATATTCCGGATTATTCATACCGTCCCACCATCGGGCGCGGATCCAACTCCTAAAAAAACCGCCACCATGGTGTTTCTGCCCCTCAA

110 120 130 140 150 160 170 180 190 200 210

ATGGTCCCTTGCAACCATGTCATTTCTACTTTCCTCACTGTTGGCTCTCTTAACTGTGTCCACTCCTTCATGGTGTCAGAGCACTGAAGCATCTCCAAAACGTAGTGA

U4947DH210-4.seq(1>2878) ATGGTCCCTTGCAACCATGTCATTTCTACTTTCCTCACTGTTGGCTCTCTTAACTGTGTCCACTCCTTCATGGTGTCAGAGCACTGAAGCATCTCCAAAACGTAGTGA

G01-pFastbac-F.ab1(1>821)

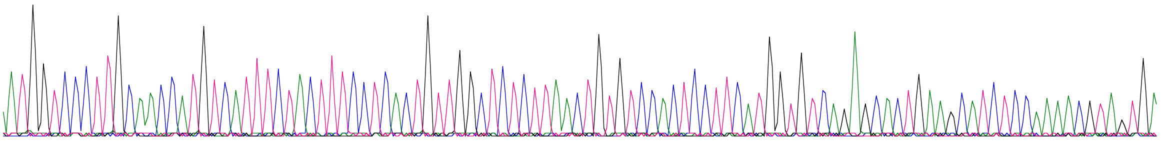

ATGGTCCCTTGCAACCATGTCATTTCTACTTTCCTCACTGTTGGCTCTCTTAACTGTGTCCACTCCTTCATGGTGTCAGAGCACTGAAGCATCTCCAAAACGTAGTGA

F11-SEQ3R.ab1(1>811)

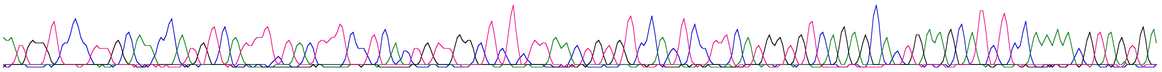

ATGGTCCCTTGCAACCATGTCATTTCTACTTTCCTCACTGTTGGCTCTCTTAACTGTGTCCACTCCTTCATGGTGTCAGAGCACTGAAGCATCTCCAAAACGTAGTGA

220 230 240 250 260 270 280 290 300 310 320

TGGGACACCATTTCCTTGGAATAAAATACGACTTCCTGAGTACGTCATCCCAGTTCATTATGATCTCTTGATCCATGCAAACCTTACCACGCTGACCTTCTGGGGAAC

U4947DH210-4.seq(1>2878) TGGGACACCATTTCCTTGGAATAAAATACGACTTCCTGAGTACGTCATCCCAGTTCATTATGATCTCTTGATCCATGCAAACCTTACCACGCTGACCTTCTGGGGAAC

G01-pFastbac-F.ab1(1>821)

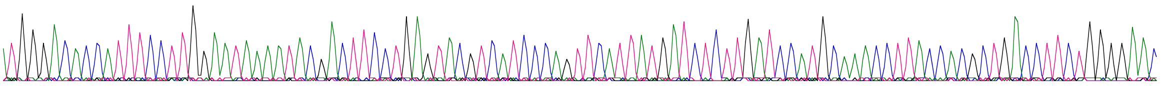

TGGGACACCATTTCCTTGGAATAAAATACGACTTCCTGAGTACGTCATCCCAGTTCATTATGATCTCTTGATCCATGCAAACCTTACCACGCTGACCTTCTGGGGAAC

220 230 240 250 260 270 280 290 300 310 320

TGGGACACCATTTCCTTGGAATAAAATACGACTTCCTGAGTACGTCATCCCAGTTCATTATGATCTCTTGATCCATGCAAACCTTACCACGCTGACCTTCTGGGGAAC

F11-SEQ3R.ab1(1>811)

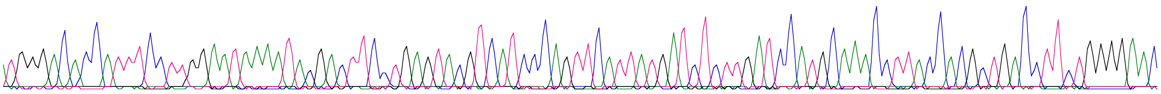

TGGGACACCATTTCCTTGGAATAAAATACGACTTCCTGAGTACGTCATCCCAGTTCATTATGATCTCTTGATCCATGCAAACCTTACCACGCTGACCTTCTGGGGAAC

330 340 350 360 370 380 390 400 410 420 430

CACGAAAGTAGAAATCACAGCCAGTCAGCCCACCAGCACCATCATCCTGCATAGTCACCACCTGCAGATATCTAGGGCCACCCTCAGGAAGGGAGCTGGAGAGAGGCT

U4947DH210-4.seq(1>2878)
G01-pFastbac-F.ab1(1>821)

CACGAAAGTAGAAATCACAGCCAGTCAGCCCACCAGCACCATCATCCTGCATAGTCACCACCTGCAGATATCTAGGGCCACCCTCAGGAAGGGAGCTGGAGAGAGGCT

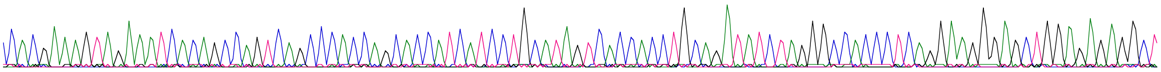

CACGAAAGTAGAAATCACAGCCAGTCAGCCCACCAGCACCATCATCCTGCATAGTCACCACCTGCAGATATCTAGGGCCACCCTCAGGAAGGGAGCTGGAGAGAGGCT

F11-SEQ3R.ab1(1>811)

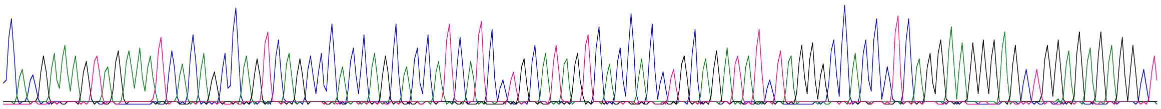

CACGAAAGTAGAAATCACAGCCAGTCAGCCCACCAGCACCATCATCCTGCATAGTCACCACCTGCAGATATCTAGGGCCACCCTCAGGAAGGGAGCTGGAGAGAGGCT

P127R

440 450 460 470 480 490 500 510 520 530 540

ATCGGAAGAACCCCTGCAGGTCCTGGAACACCCGCGTCAGGAGCAAATTGCACTGCTGGCTCCCGAGCCCCTCCTTGTCGGGCTCCCGTACACAGTTGTCATTCACTA

U4947DH210-4.seq(1>2878)
G01-pFastbac-F.ab1(1>821)

ATCGGAAGAACCCCTGCAGGTCCTGGAACACCCGCGTCAGGAGCAAATTGCACTGCTGGCTCCCGAGCCCCTCCTTGTCGGGCTCCCGTACACAGTTGTCATTCACTA

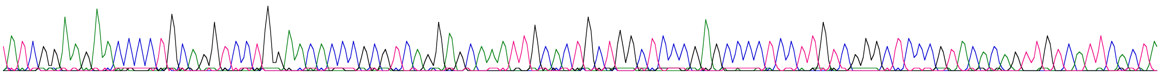

ATCGGAAGAACCCCTGCAGGTCCTGGAACACCCGCGTCAGGAGCAAATTGCACTGCTGGCTCCCGAGCCCCTCCTTGTCGGGCTCCCGTACACAGTTGTCATTCACTA

F11-SEQ3R.ab1(1>811)

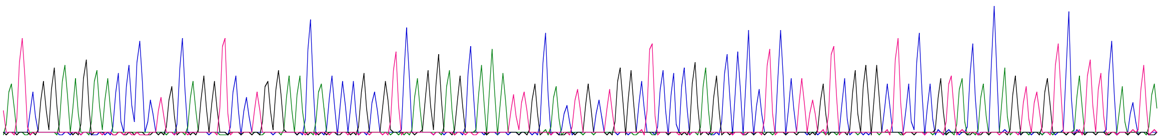

ATCGGAAGAACCCCTGCAGGTCCTGGAACACCCGCGTCAGGAGCAAATTGCACTGCTGGCTCCCGAGCCCCTCCTTGTCGGGCTCCCGTACACAGTTGTCATTCACTA

550 560 570 580 590 600 610 620 630 640

TGCTGGCAATCTTTCGGAGACTTTCCACGGATTTTACAAAAGCACCTACAGAACCAAGGAAGGGGAACTGAGGATACTAGCATCAACACAATTTGAACCCACTGCAGC

U4947DH210-4.seq(1>2878) TGCTGGCAATCTTTCGGAGACTTTCCACGGATTTTACAAAAGCACCTACAGAACCAAGGAAGGGGAACTGAGGATACTAGCATCAACACAATTTGAACCCACTGCAGC
G01-pFastbac-F.ab1(1>821)

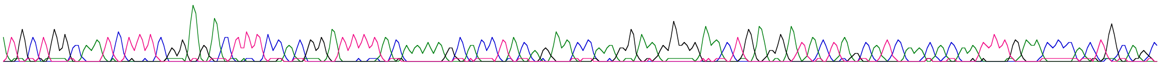

TGCTGGCAATCTTTCGGAGACTTTCCACGGATTTTACAAAAGCACCTACAGAACCAAGGAAGGGGAACTGAGGATACTAGCATCAACACAATTTGAACCCACTGCAGC

F11-SEQ3R.ab1(1>811)

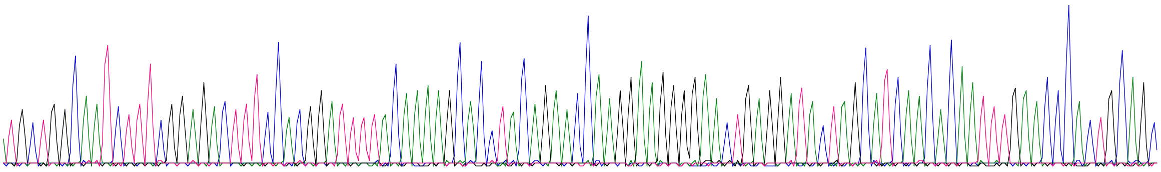

TGCTGGCAATCTTTCGGAGACTTTCCACGGATTTTACAAAAGCACCTACAGAACCAAGGAAGGGGAACTGAGGATACTAGCATCAACACAATTTGAACCCACTGCAGC

650 660 670 680 690 700 710 720 730 740 750

TAGAATGGCCTTTCCCTGCTTTGATGAACCTGCCTTCAAAGCAAGTTTCTCAATCAAAATTAGAAGAGAGCCAAGGCACCTAGCCATCTCCAATATGCCATTGGTGAA

U4947DH210-4.seq(1>2878) TAGAATGGCCTTTCCCTGCTTTGATGAACCTGCCTTCAAAGCAAGTTTCTCAATCAAAATTAGAAGAGAGCCAAGGCACCTAGCCATCTCCAATATGCCATTGGTGAA
G01-pFastbac-F.ab1(1>821)

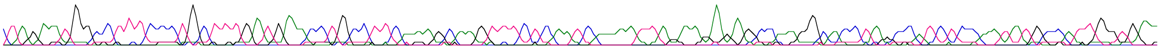

TAGAATGGCCTTTCCCTGCTTTGATGAACCTGCCTTCAAAGCAAGTTTCTCAATCAAAATTAGAAGAGAGCCAAGGCACCTAGCCATCTCCAATATGCCATTGGTGAA

F11-SEQ3R.ab1(1>811)

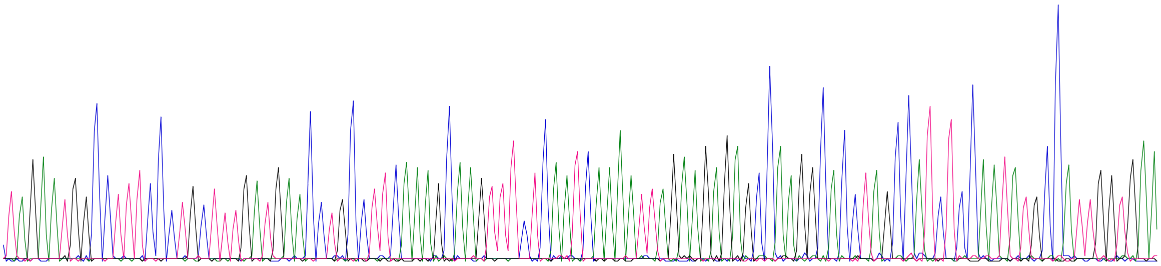

TAGAATGGCCTTTCCCTGCTTTGATGAACCTGCCTTCAAAGCAAGTTTCTCAATCAAAATTAGAAGAGAGCCAAGGCACCTAGCCATCTCCAATATGCCATTGGTGAA

F10-SEQ2R.ab1(1>825)

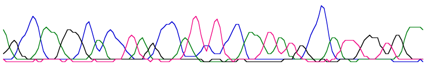

AGCCAAGGCACCTAGCCATCTCCAATATGCCATTGGTGAA

G03-SEQ1.ab1(1>825)

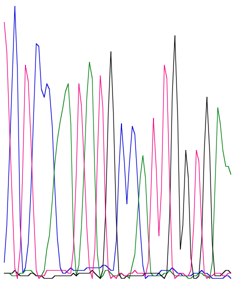

TCTCCaATATGCCATTGGTGAA

760 770 780 790 800 810 820 830 840 850 860

ATCTGTGACTGTTGCTGAAGGACTCATAGAAGACCATTTTGATGTCACTGTGAAGATGAGCACCTATCTGGTGGCCTTCATCATTTCAGATTTTGAGTCTGTCAGCAA

U4947DH210-4.seq(1>2878) ATCTGTGACTGTTGCTGAAGGACTCATAGAAGACCATTTTGATGTCACTGTGAAGATGAGCACCTATCTGGTGGCCTTCATCATTTCAGATTTTGAGTCTGTCAGCAA
G01-pFastbac-F.ab1(1>821)

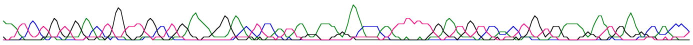

ATCTGTGACTGTTGCTGAAGGACTCATAGAAGACCATTTTGATGTCACTGTGAAGATGAGCACCT

F11-SEQ3R.ab1(1>811)

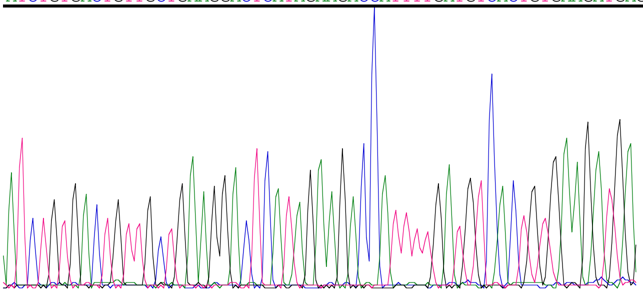

ATCTGTGACTGTTGCTGAAGGACTCATAGAAGACCATTTTGATGTCACTGTGAAGATGAG

F10-SEQ2R.ab1(1>825)

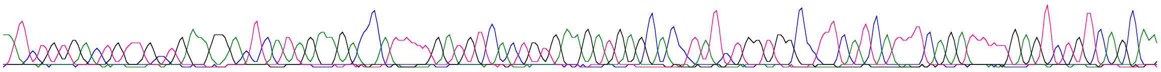

ATCTGTGACTGTTGCTGAAGGACTCATAGAAGACCATTTTGATGTCACTGTGAAGATGAGCACCTATCTGGTGGCCTTCATCATTTCAGATTTTGAGTCTGTCAGCAA

G03-SEQ1.ab1(1>825)

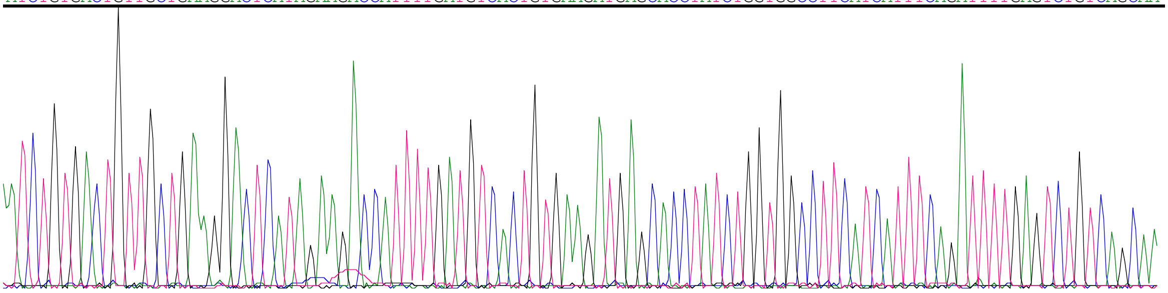

ATCTGTGACTGTTGCTGAAGGACTCATAGAAGACCATTTTGATGTCACTGTGAAGATGAGCACCTATCTGGTGGCCTTCATCATTTCAGATTTTGAGTCTGTCAGCAA

870 880 890 900 910 920 930 940 950 960 970

GATAACCAAGAGTGGAGTCAAGGTTTCTGTTTATGCTGTGCCAGACAAGATAAATCAAGCAGATTATGCACTGGATGCTGCGGTGACTCTTCTAGAATTTTATGAGGA

U4947DH210-4.seq(1>2878) GATAACCAAGAGTGGAGTCAAGGTTTCTGTTTATGCTGTGCCAGACAAGATAAATCAAGCAGATTATGCACTGGATGCTGCGGTGACTCTTCTAGAATTTTATGAGGA
F10-SEQ2R.ab1(1>825)

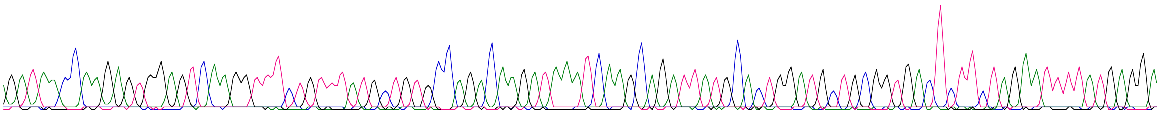

GATAACCAAGAGTGGAGTCAAGGTTTCTGTTTATGCTGTGCCAGACAAGATAAATCAAGCAGATTATGCACTGGATGCTGCGGTGACTCTTCTAGAATTTTATGAGGA

G03-SEQ1.ab1(1>825)

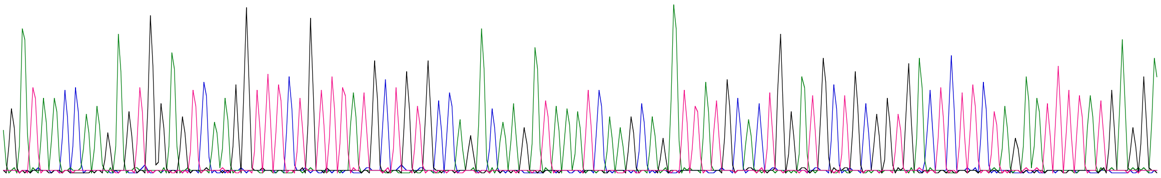

GATAACCAAGAGTGGAGTCAAGGTTTCTGTTTATGCTGTGCCAGACAAGATAAATCAAGCAGATTATGCACTGGATGCTGCGGTGACTCTTCTAGAATTTTATGAGGA

980 990 1,000 1,010 1,020 1,030 1,040 1,050 1,060 1,070 1,080

TTATTTCAGCATACCGTATCCCCTACCCAAACAAGATCTTGCTGCTATTCCCGACTTTCAGTCTGGTGCTATGGAAAACTGGGGACTGACAACATATAGAGAATCTGC

U4947DH210-4.seq(1>2878) TTATTTCAGCATACCGTATCCCCTACCCAAACAAGATCTTGCTGCTATTCCCGACTTTCAGTCTGGTGCTATGGAAAACTGGGGACTGACAACATATAGAGAATCTGC

F10-SEQ2R.ab1(1>825)

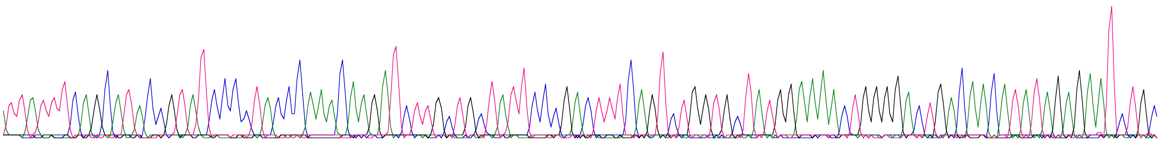

TTATTTCAGCATACCGTATCCCCTACCCAAACAAGATCTTGCTGCTATTCCCGACTTTCAGTCTGGTGCTATGGAAAACTGGGGACTGACAACATATAGAGAATCTGC

G03-SEQ1.ab1(1>825)

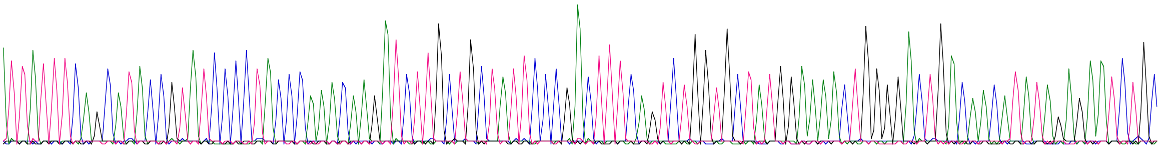

TTATTTCAGCATACCGTATCCCCTACCCAAACAAGATCTTGCTGCTATTCCCGACTTTCAGTCTGGTGCTATGGAAAACTGGGGACTGACAACATATAGAGAATCTGC

1,090 1,100 1,110 1,120 1,130 1,140 1,150 1,160 1,170 1,180

TCTGTTGTTTGATGCAGAAAAGTCTTCTGCATCAAGTAAGCTTGGCATCACAATGACTGTGGCCCATGAACTGGCTCACCAGTGGTTTGGGAACCTGGTCACTATGGA

U4947DH210-4.seq(1>2878) TCTGTTGTTTGATGCAGAAAAGTCTTCTGCATCAAGTAAGCTTGGCATCACAATGACTGTGGCCCATGAACTGGCTCACCAGTGGTTTGGGAACCTGGTCACTATGGA

F10-SEQ2R.ab1(1>825)

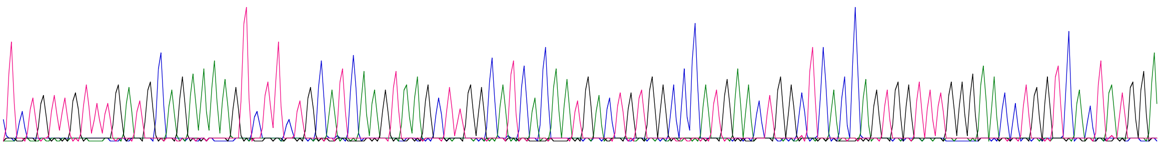

TCTGTTGTTTGATGCAGAAAAGTCTTCTGCATCAAGTAAGCTTGGCATCACAATGACTGTGGCCCATGAACTGGCTCACCAGTGGTTTGGGAACCTGGTCACTATGGA

G03-SEQ1.ab1(1>825)

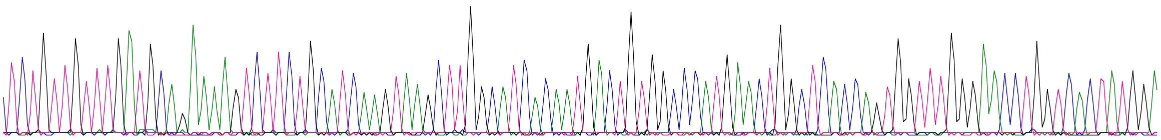

TCTGTTGTTTGATGCAgAAAAGTCTTCTGCATCAAGTAAGCTTGGCATCACAATGACTGTGGCCCATGAACTGGCTCACCAGTGGTTTGGGAACCTGGTCACTATGGA

1,190 1,200 1,210 1,220 1,230 1,240 1,250 1,260 1,270 1,280 1,290

ATGGTGGAATGATCTTTGGCTAAATGAAGGATTTGCCAAATTTATGGAGTTTGTGTCTGTCAGTGTGACCCATCCTGAACTGAAAGTTGGAGATTATTTCTTTGGCAA

U4947DH210-4.seq(1>2878) ATGGTGGAATGATCTTTGGCTAAATGAAGGATTTGCCAAATTTATGGAGTTTGTGTCTGTCAGTGTGACCCATCCTGAACTGAAAGTTGGAGATTATTTCTTTGGCAA

F10-SEQ2R.ab1(1>825)

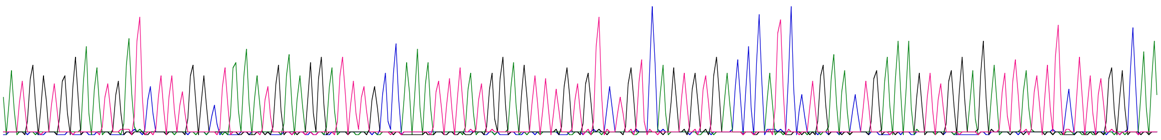

ATGGTGGAATGATCTTTGGCTAAATGAAGGATTTGCCAAATTTATGGAGTTTGTGTCTGTCAGTGTGACCCATCCTGAACTGAAAGTTGGAGATTATTTCTTTGGCAA

1,190 1,200 1,210 1,220 1,230 1,240 1,250 1,260 1,270 1,280 1,290

ATGGTGGAATGATCTTTGGCTAAATGAAGGATTTGCCAAATTTATGGAGTTTGTGTCTGTCAGTGTGACCCATCCTGAACTGAAAGTTGGAGATTATTTCTTTGGCAA

G03-SEQ1.ab1(1>825)

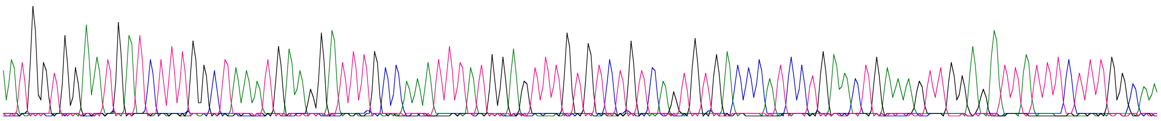

ATGGTGGAATGATCTTTGGCTAAATGAAGGATTTGCCAAATTTATGGAGTTTGTGTCTGTCAGTGTGACCCATCCTGAACTGAAAGTTGGAGATTATTTCTTTGGCAA

1,300 1,310 1,320 1,330 1,340 1,350 1,360 1,370 1,380 1,390 1,400

ATGTTTTGACGCAATGGAGGTAGATGCTTTAAATTCCTCACACCCTGTGTCTACACCTGTGGAAAATCCTGCTCAGATCCGGGAGATGTTTGATGATGTTTCTTATGA

U4947DH210-4.seq(1>2878) ATGTTTTGACGCAATGGAGGTAGATGCTTTAAATTCCTCACACCCTGTGTCTACACCTGTGGAAAATCCTGCTCAGATCCGGGAGATGTTTGATGATGTTTCTTATGA

F10-SEQ2R.ab1(1>825)

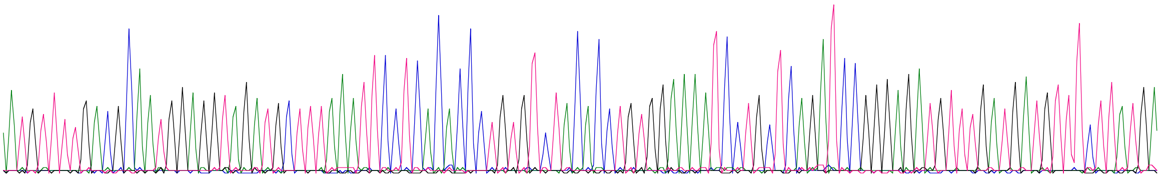

ATGTTTTGACGCAATGGAGGTAGATGCTTTAAATTCCTCACACCCTGTGTCTACACCTGTGGAAAATCCTGCTCAGATCCGGGAGATGTTTGATGATGTTTCTTATGA

G03-SEQ1.ab1(1>825)

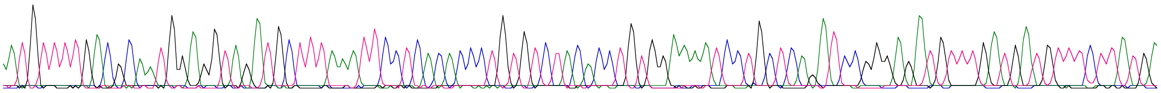

ATGTTTTGACGCAATGGAGGTAGATGCTTTAAATTCCTCACACCCTGTGTCTACACCTGTGGAAAATCCTGCTCAGATCCGGGAGATGTTTGATGATGTTTCTTATGA

1,410 1,420 1,430 1,440 1,450 1,460 1,470 1,480 1,490 1,500 1,510

TAAGGGAGCTTGTATTCTGAATATGCTAAGGGAGTATCTTAGTGCTGACGCATTTAAAAGTGGTATTGTACAGTATCTCCAGAAGCATAGCTATAAAAATACAAAAAA

U4947DH210-4.seq(1>2878) TAAGGGAGCTTGTATTCTGAATATGCTAAGGGAGTATCTTAGTGCTGACGCATTTAAAAGTGGTATTGTACAGTATCTCCAGAAGCATAGCTATAAAAATACAAAAAA

F10-SEQ2R.ab1(1>825)

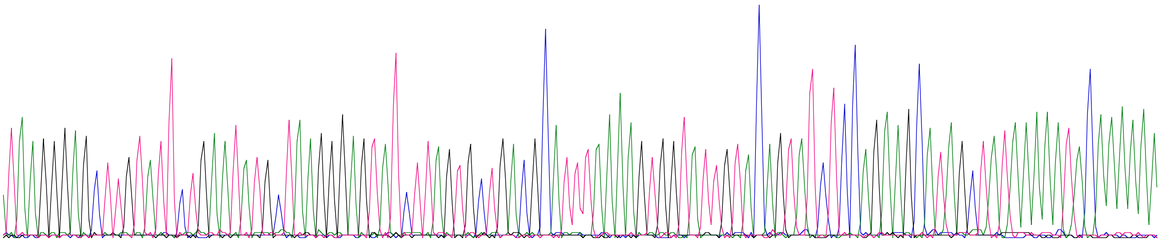

TAAGGGAGCTTGTATTCTGAATATGCTAAGGGAGTATCTTAGTGCTGACGCATTTAAAAGTGGTATTGTACAGTATCTCCAGAAGCATAGCTATAAAAATACAAAAAA

G03-SEQ1.ab1(1>825)

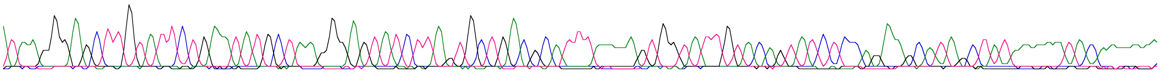

TAAGGGAGCTTGTATTCTGAATATGCTAAGGGAGTATCTTAGTGCTGACGCATTTAAAAGTGGTATTGTACAGTATCTCCAGAAGCATAGCTATAAAAATACAAAAAA

1,410 1,420 1,430 1,440 1,450 1,460 1,470 1,480 1,490 1,500 1,510

TAAGGGAGCTTGTATTCTGAATATGCTAAGGGAGTATCTTAGTGCTGACGCATTTAAAAGTGGTATTGTACAGTATCTCCAGAAGCATAGCTATAAAAATACAAAAAA

G06-SEQ1R.ab1(1>824)

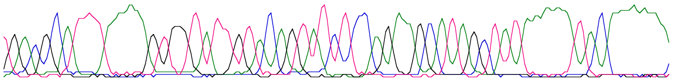

TGACGCATTTAAAAGTGGTATTGTACAGTATCTCCAGAAGCATAGCTATAAAAATACAAAAAA

G04-SEQ2.ab1(1>821)

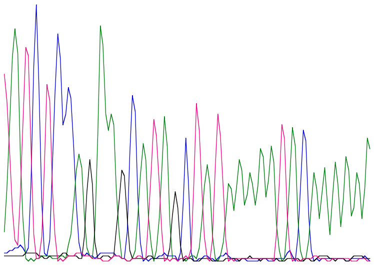

TATCTCCAGAAGCATAGCTATAAAAATACAAAAAA

1,520 1,530 1,540 1,550 1,560 1,570 1,580 1,590 1,600 1,610 1,620

CGAGGACCTGTGGGATAGTATGGCAAGTATTTGCCCTACAGATGGTGTAAAAGGGATGGATGGCTTTTGCTCTAGAAGTCAACATTCATCTTCATCCTCACATTGGCA

U4947DH210-4.seq(1>2878) CGAGGACCTGTGGGATAGTATGGCAAGTATTTGCCCTACAGATGGTGTAAAAGGGATGGATGGCTTTTGCTCTAGAAGTCAACATTCATCTTCATCCTCACATTGGCA

F10-SEQ2R.ab1(1>825)

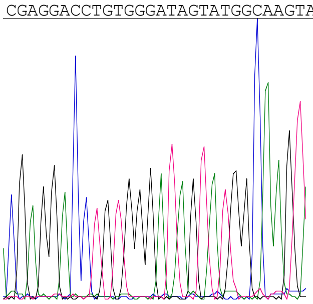

CGAGGACCTGTGGGATAGTATGGCAAGTA

G03-SEQ1.ab1(1>825)

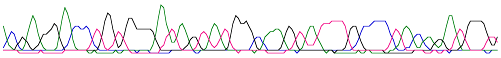

CGAGGACCTGTGGGATAGTATGGCAAGTATTTGCCCTACAGATGGTG

G06-SEQ1R.ab1(1>824)

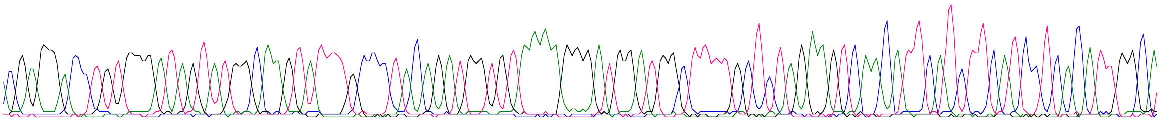

CGAGGACCTGTGGGATAGTATGGCAAGTATTTGCCCTACAGATGGTGTAAAAGGGATGGATGGCTTTTGCTCTAGAAGTCAACATTCATCTTCATCCTCACATTGGCA

G04-SEQ2.ab1(1>821)

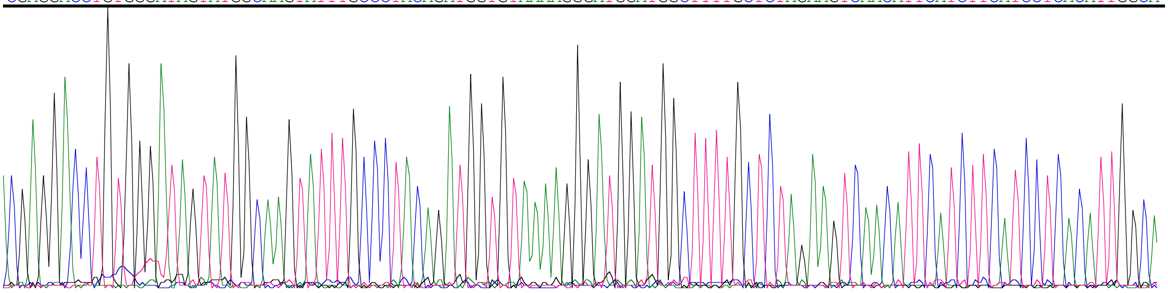

CGAGGACCTGTGGGATAGTATGGCAAGTATTTGCCCTACAGATGGTGTAAAAGGGATGGATGGCTTTTGCTCTAGAAGTCAACATTCATCTTCATCCTCACATTGGCA

K528R

1,630 1,640 1,650 1,660 1,670 1,680 1,690 1,700 1,710 1,720

TCAGGAAGGGGTGGATGTGAAAACCATGATGAACACTTGGACACTGCAGCGTGGTTTTCCCCTAATAACCATCACAGTGAGGGGGAGGAATGTACACATGAAGCAAGA

U4947DH210-4.seq(1>2878) TCAGGAAGGGGTGGATGTGAAAACCATGATGAACACTTGGACACTGCAGCGTGGTTTTCCCCTAATAACCATCACAGTGAGGGGGAGGAATGTACACATGAAGCAAGA
G06-SEQ1R.ab1(1>824)

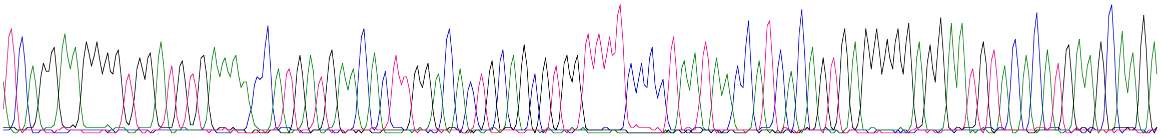

TCAGGAAGGGGTGGATGTGAAAACCATGATGAACACTTGGACACTGCAGCGTGGTTTTCCCCTAATAACCATCACAGTGAGGGGGAGGAATGTACACATGAAGCAAGA

G04-SEQ2.ab1(1>821)

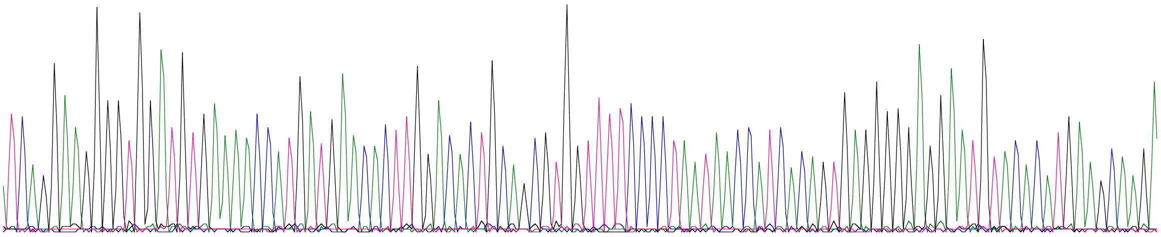

TCAGGAAGGGGTGGATGTGAAAACCATGATGAACACTTGGACACTGCAGCGTGGTTTTCCCCTAATAACCATCACAGTGAGGGGGAGGAATGTACACATGAAGCAAGA

1,730 1,740 1,750 1,760 1,770 1,780 1,790 1,800 1,810 1,820 1,830

GCACTACATGAAGGGCTCTGACGGCGCCCCGGACACTGGGTACCTGTGGCATGTTCCATTGACATTCATCACCAGCAAATCCGACATGGTCCATCGATTTTTGCTAAA

U4947DH210-4.seq(1>2878) GCACTACATGAAGGGCTCTGACGGCGCCCCGGACACTGGGTACCTGTGGCATGTTCCATTGACATTCATCACCAGCAAATCCGACATGGTCCATCGATTTTTGCTAAA
G06-SEQ1R.ab1(1>824)

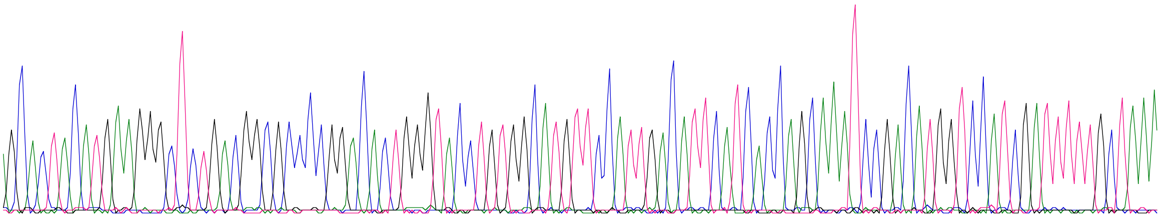

GCACTACATGAAGGGCTCTGACGGCGCCCCGGACACTGGGTACCTGTGGCATGTTCCATTGACATTCATCACCAGCAAATCCGACATGGTCCATCGATTTTTGCTAAA

G04-SEQ2.ab1(1>821)

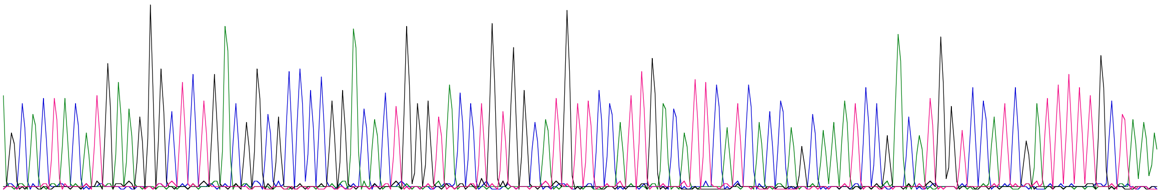

GCACTACATGAAGGGCTCTGACGGCGCCCCGGACACTGGGTACCTGTGGCATGTTCCATTGACATTCATCACCAGCAAATCCGACATGGTCCATCGaTTTTTGCTAAA

1,840 1,850 1,860 1,870 1,880 1,890 1,900 1,910 1,920 1,930 1,940

AACAAAAACAGATGTGCTCATCCTCCCAGAAGAGGTGGAATGGATCAAATTTAATGTGGGCATGAATGGCTATTACATTGTGCATTACGAGGATGATGGATGGGACTC

U4947DH210-4.seq(1>2878) AACAAAAACAGATGTGCTCATCCTCCCAGAAGAGGTGGAATGGATCAAATTTAATGTGGGCATGAATGGCTATTACATTGTGCATTACGAGGATGATGGATGGGACTC
G06-SEQ1R.ab1(1>824)

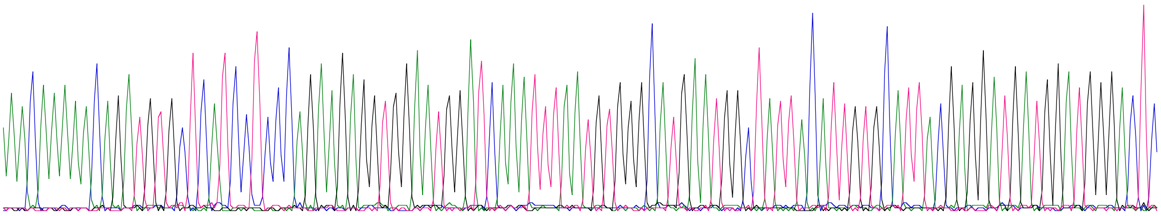

AACAAAAACAGATGTGCTCATCCTCCCAGAAGAGGTGGAATGGATCAAATTTAATGTGGGCATGAATGGCTATTACATTGTGCATTACGAGGATGATGGATGGGACTC

1,840 1,850 1,860 1,870 1,880 1,890 1,900 1,910 1,920 1,930 1,940

AACAAAAACAGATGTGCTCATCCTCCCAGAAGAGGTGGAATGGATCAAATTTAATGTGGGCATGAATGGCTATTACATTGTGCATTACGAGGATGATGGATGGGACTC

G04-SEQ2.ab1(1>821)

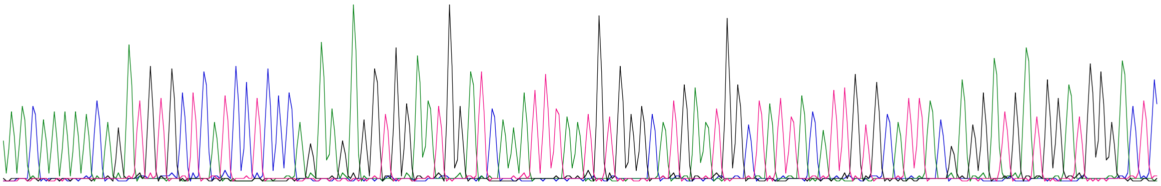

AACAAAAACAGATGTGCTCATCCTCCCAGAAGAGGTGGAATGGATCAAATTTAATGTGGGCATGAATGGCTATTACATTGTGCATTACGAGGATGATGGATGGGACTC

1,950 1,960 1,970 1,980 1,990 2,000 2,010 2,020 2,030 2,040 2,050

TTTGACTGGCCTTTTAAAAGGAACACACACAGCAGTCAGCAGTAATGATCGGGCGAGTCTCATTAACAATGCATTTCAGCTCGTCAGCATTGGGAAGCTGTCCATTGA

U4947DH210-4.seq(1>2878) TTTGACTGGCCTTTTAAAAGGAACACACACAGCAGTCAGCAGTAATGATCGGGCGAGTCTCATTAACAATGCATTTCAGCTCGTCAGCATTGGGAAGCTGTCCATTGA
G06-SEQ1R.ab1(1>824)

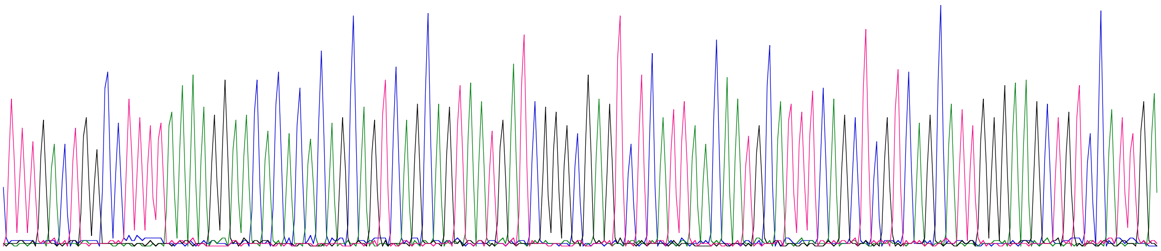

TTTGACTGGCCTTTTAAAAGGAACACACACAGCAGTCAGCAGTAATGATCGGGCGAGTCTCATTAACAATGCATTTCAGCTCGTCAGCATTGGGAAGCTGTCCATTGA

G04-SEQ2.ab1(1>821)

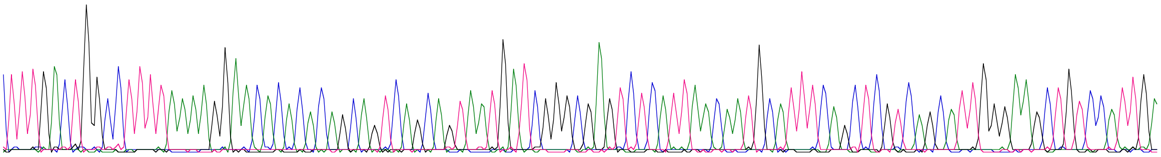

TTTGACTGGCCTTTTAAAAGGAACACACACAGCAGTCAGCAGTAATGATCGGGCGAGTCTCATTAACAATGCATTTCAGCTCGTCAGCATTGGGAAGCTGTCCATTGA

2,060 2,070 2,080 2,090 2,100 2,110 2,120 2,130 2,140 2,150 2,160

AAAGGCCTTGGATTTATCCCTGTACTTGAAACATGAAACTGAAATTATGCCCGTGTTTCAAGGTTTGAATGAGCTGATTCCTATGTATAAGTTAATGGAGAAAAGAGA

U4947DH210-4.seq(1>2878) AAAGGCCTTGGATTTATCCCTGTACTTGAAACATGAAACTGAAATTATGCCCGTGTTTCAAGGTTTGAATGAGCTGATTCCTATGTATAAGTTAATGGAGAAAAGAGA
G06-SEQ1R.ab1(1>824)

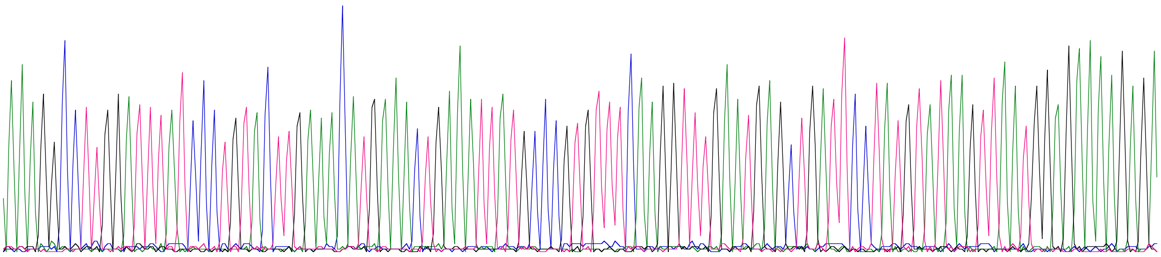

AAAGGCCTTGGATTTATCCCTGTACTTGAAACATGAAACTGAAATTATGCCCGTGTTTCAAGGTTTGAATGAGCTGATTCCTATGTATAAGTTAATGGAGAAAAGAGA

G04-SEQ2.ab1(1>821)

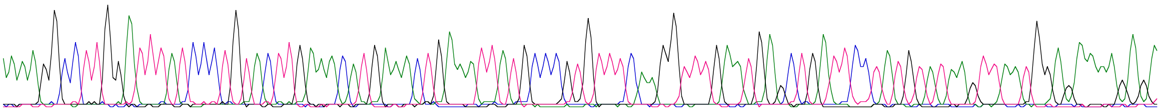

AAAGGCCTTGGATTTATCCCTGTACTTGAAACATGAAACTGAAATTATGCCCGTGTTTCAAGGTTTGAATGAGCTGATTCCTATGTATAAGTTAATGGAGAAAAGAGA

2,170 2,180 2,190 2,200 2,210 2,220 2,230 2,240 2,250 2,260

TATGAATGAAGTGGAAACTCAATTCAAGGCCTTCCTCATCAGGCTGCTAAGGGACCTCATTGATAAGCAGACATGGACAGACGAGGGCTCAGTCTCAGAGCGAATGCT

U4947DH210-4.seq(1>2878) TATGAATGAAGTGGAAACTCAATTCAAGGCCTTCCTCATCAGGCTGCTAAGGGACCTCATTGATAAGCAGACATGGACAGACGAGGGCTCAGTCTCAGAGCGAATGCT

G06-SEQ1R.ab1(1>824)

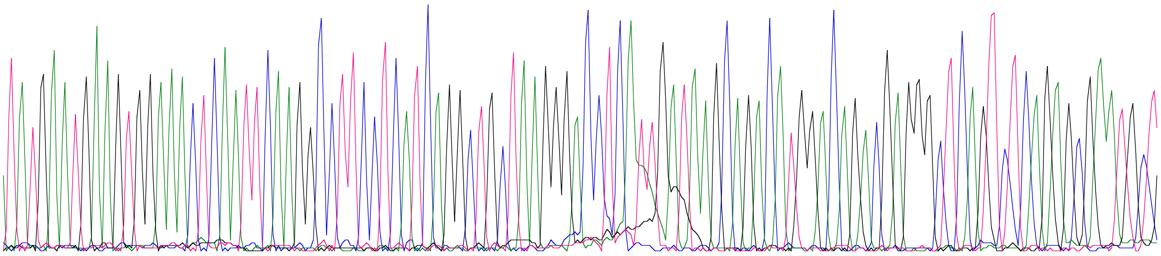

TATGAATGAAGTGGAAACTCAATTCAAGGCCTTCCTCATCAGGCTGCTAAGGGACCTCATTGATAAGCAGACATGGACAGACGAGGGCTCAGTCTCAGAGCGAATGCT

G04-SEQ2.ab1(1>821)

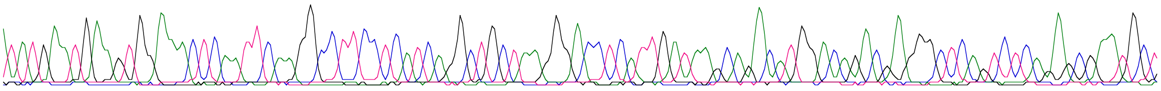

TATGAATGAAGTGGAAACTCAATTCAAGGCCTTCCTCATCAGGCTGCTAAGGGACCTCATTGATAAGCAGACATGGACAGACGAGGGCTCAGTCTCAGAGCGAATGCT

G02-pFastbac-R.ab1(1>826)

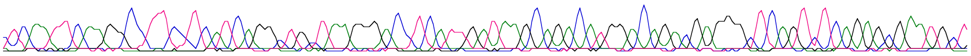

CTCAATTCAAGGCCTTCCTCATCAGGCTGCTAAGGGACCTCATTGATAAGCAGACATGGACAGACGAGGGCTCAGTCTCAGAGCGAATGCT

G05-SEQ3.ab1(1>826)

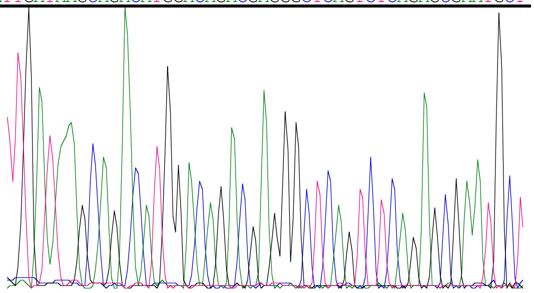

TTGATAAGCAGACATGGACAGACGAGGGCTCAGTCTCAGAGCGAATGCT

2,270 2,280 2,290 2,300 2,310 2,320 2,330 2,340 2,350 2,360 2,370

GCGGAGTGAGCTACTACTCCTCGCCTGTGTGCACAACTATCAGCCGTGCGTACAGAGGGCAGAAGGCTATTTCAGAAAGTGGAAGGAATCCAATGGAAACTTGAGCCT

U4947DH210-4.seq(1>2878) GCGGAGTGAGCTACTACTCCTCGCCTGTGTGCACAACTATCAGCCGTGCGTACAGAGGGCAGAAGGCTATTTCAGAAAGTGGAAGGAATCCAATGGAAACTTGAGCCT

G06-SEQ1R.ab1(1>824)

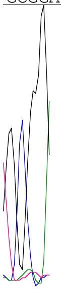

GCGGA

G04-SEQ2.ab1(1>821)

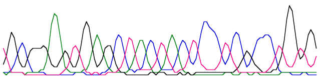

GCGGAGTGAGCTACTACTCCTCGCCTGTGT

Q730E

2,270 2,280 2,290 2,300 2,310 2,320 2,330 2,340 2,350 2,360 2,370

GCGGAGTGAGCTACTACTCCTCGCCTGTGTGCACAACTATCAGCCGTGCGTACAGAGGGCAGAAGGCTATTTCAGAAAGTGGAAGGAATCCAATGGAAACTTGAGCCT

G02-pFastbac-R.ab1(1>826)

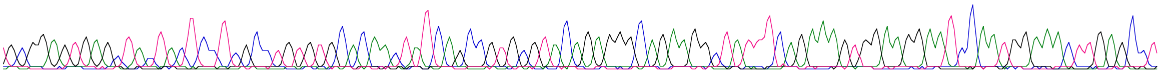

GCGGAGTGAGCTACTACTCCTCGCCTGTGTGCACAACTATCAGCCGTGCGTACAGAGGGCAGAAGGCTATTTCAGAAAGTGGAAGGAATCCAATGGAAACTTGAGCCT

G05-SEQ3.ab1(1>826)

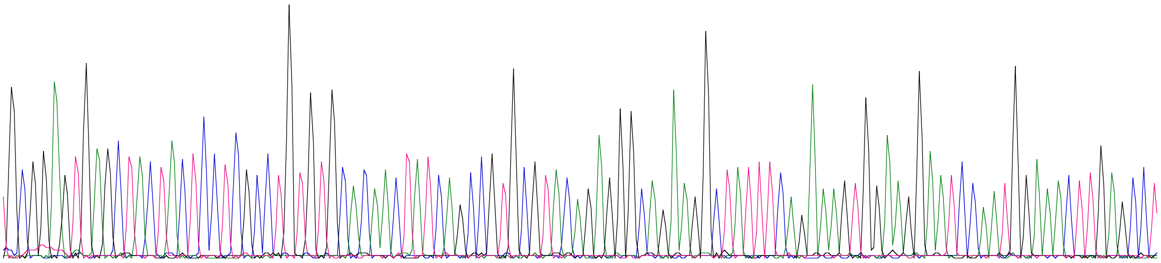

GCGGAGTGAGCTACTACTCCTCGCCTGTGTGCACAACTATCAGCCGTGCGTACAGAGGGCAGAAGGCTATTTCAGAAAGTGGAAGGAATCCAATGGAAACTTGAGCCT

2,380 2,390 2,400 2,410 2,420 2,430 2,440 2,450 2,460 2,470 2,480

GCCTGTCGACGTGACCTTGGCAGTGTTTGCTGTGGGGGCCCAGAGCACAGAAGGCTGGGATTTTCTTTATAGTAAATATCAGTTTTCTTTGTCCAGTACTGAGAAAAG

U4947DH210-4.seq(1>2878) GCCTGTCGACGTGACCTTGGCAGTGTTTGCTGTGGGGGCCCAGAGCACAGAAGGCTGGGATTTTCTTTATAGTAAATATCAGTTTTCTTTGTCCAGTACTGAGAAAAG

G02-pFastbac-R.ab1(1>826)

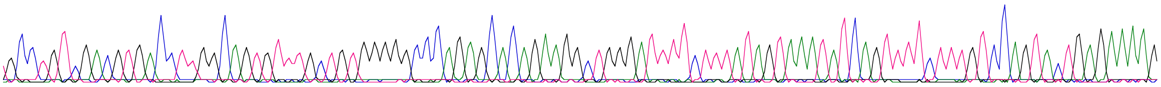

GCCTGTCGACGTGACCTTGGCAGTGTTTGCTGTGGGGGCCCAGAGCACAGAAGGCTGGGATTTTCTTTATAGTAAATATCAGTTTTCTTTGTCCAGTACTGAGAAAAG

G05-SEQ3.ab1(1>826)

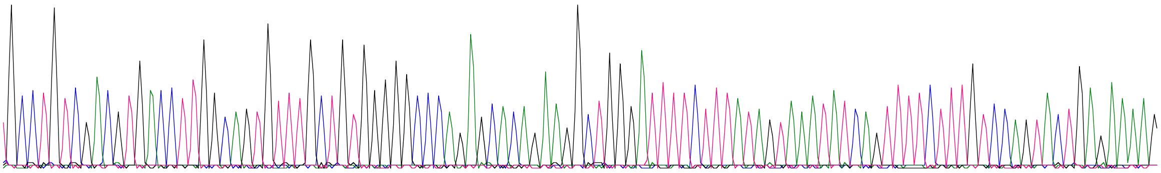

GCCTGTCGACGTGACCTTGGCAGTGTTTGCTGTGGGGGCCCAGAGCACAGAAGGCTGGGATTTTCTTTATAGTAAATATCAGTTTTCTTTGTCCAGTACTGAGAAAAG

2,490 2,500 2,510 2,520 2,530 2,540 2,550 2,560 2,570 2,580 2,590

CCAAATTGAATTTGCCCTCTGCAGAACCCAAAATAAGGAAAAGCTTCAATGGCTACTAGATGAAAGCTTTAAGGGAGATAAAATAAAAACTCAGGAGTTTCCACAAAT

U4947DH210-4.seq(1>2878) CCAAATTGAATTTGCCCTCTGCAGAACCCAAAATAAGGAAAAGCTTCAATGGCTACTAGATGAAAGCTTTAAGGGAGATAAAATAAAAACTCAGGAGTTTCCACAAAT

G02-pFastbac-R.ab1(1>826)

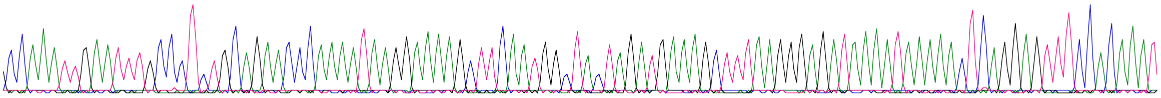

CCAAATTGAATTTGCCCTCTGCAGAACCCAAAATAAGGAAAAGCTTCAATGGCTACTAGATGAAAGCTTTAAGGGAGATAAAATAAAAACTCAGGAGTTTCCACAAAT

2,490 2,500 2,510 2,520 2,530 2,540 2,550 2,560 2,570 2,580 2,590

CCAAATTGAATTTGCCCTCTGCAGAACCCAAAATAAGGAAAAGCTTCAATGGCTACTAGATGAAAGCTTTAAGGGAGATAAAATAAAAACTCAGGAGTTTCCACAAAT

G05-SEQ3.ab1(1>826)

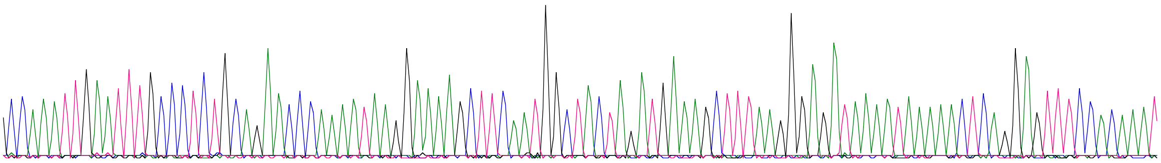

CCAAATTGAATTTGCCCTCTGCAGAACCCAAAATAAGGAAAAGCTTCAATGGCTACTAGATGAAAGCTTTAAGGGAGATAAAATAAAAACTCAGGAGTTTCCACAAAT

2,600 2,610 2,620 2,630 2,640 2,650 2,660 2,670 2,680 2,690 2,700

TCTTACACTCATTGGCAGGAACCCAGTAGGATACCCACTGGCCTGGCAATTTCTGAGGAAAAACTGGAACAAACTTGTACAAAAGTTTGAACTTGGCTCATCTTCCAT

U4947DH210-4.seq(1>2878) TCTTACACTCATTGGCAGGAACCCAGTAGGATACCCACTGGCCTGGCAATTTCTGAGGAAAAACTGGAACAAACTTGTACAAAAGTTTGAACTTGGCTCATCTTCCAT

G02-pFastbac-R.ab1(1>826)

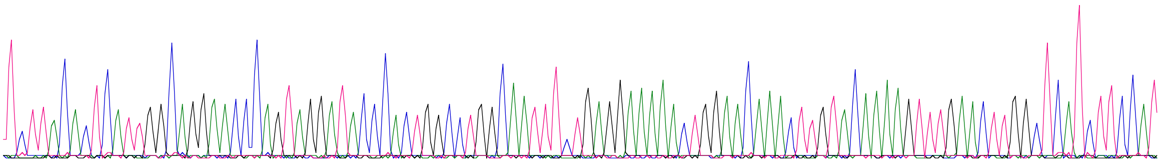

TCTTACACTCATTGGCAGGAACCCAGTAGGATACCCACTGGCCTGGCAATTTCTGAGGAAAAACTGGAACAAACTTGTACAAAAGTTTGAACTTGGCTCATCTTCCAT

G05-SEQ3.ab1(1>826)

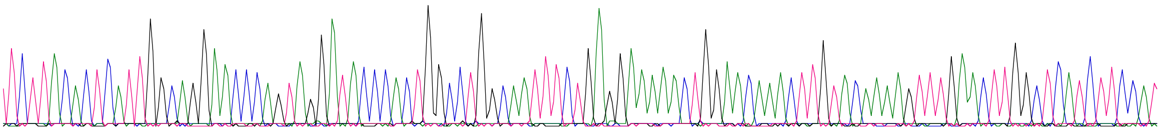

TCTTACACTCATTGGCAGGAACCCAGTAGGATACCCACTGGCCTGGCAATTTCTGAGGAAAAACTGGAACAAACTTGTACAAAAGTTTGAACTTGGCTCATCTTCCAT

2,710 2,720 2,730 2,740 2,750 2,760 2,770 2,780 2,790 2,800

AGCCCACATGGTAATGGGTACAACAAATCAATTCTCCACAAGAACACGGCTTGAAGAGGTAAAAGGATTCTTCAGCTCTTTGAAAGAAAATGGTTCTCAGCTCCGTTG

U4947DH210-4.seq(1>2878) AGCCCACATGGTAATGGGTACAACAAATCAATTCTCCACAAGAACACGGCTTGAAGAGGTAAAAGGATTCTTCAGCTCTTTGAAAGAAAATGGTTCTCAGCTCCGTTG

G02-pFastbac-R.ab1(1>826)

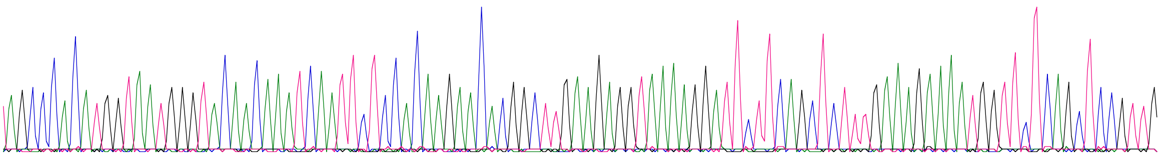

AGCCCACATGGTAATGGGTACAACAAATCAATTCTCCACAAGAACACGGCTTGAAGAGGTAAAAGGATTCTTCAGCTCTTTGAAAGAAAATGGTTCTCAGCTCCGTTG

G05-SEQ3.ab1(1>826)

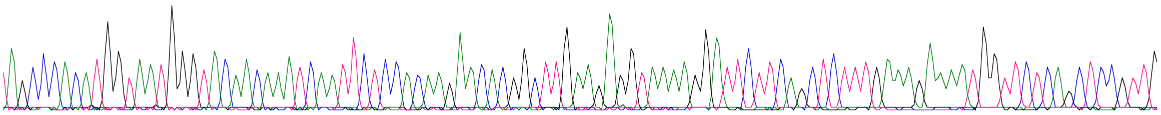

AGCCCACATGGTAATGGGTACAACAAATCAATTCTCCACAAGAACACGGCTTGAAGAGGTAAAAGGATTCTTCAGCTCTTTGAAAGAAAATGGTTCTCAGCTCCGTTG

2,810 2,820 2,830 2,840 2,850 2,860 2,870 2,880 2,890 2,900 2,910

TGTCCAACAGACAATTGAAACCATTGAAGAAAACATCGGTTGGATGGATAAGAATTTTGATAAAATCAGAGTGTGGCTGCAAAGTGAAAAGCTTGAACGCATGCATCA

U4947DH210-4.seq(1>2878) TGTCCAACAGACAATTGAAACCATTGAAGAAAACATCGGTTGGATGGATAAGAATTTTGATAAAATCAGAGTGTGGCTGCAAAGTGAAAAGCTTGAACGCATGCATCA

G02-pFastbac-R.ab1(1>826)

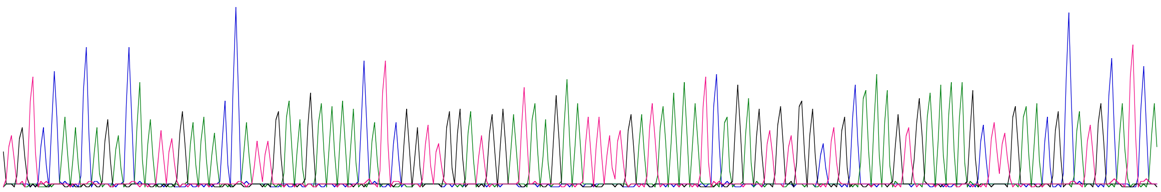

TGTCCAACAGACAATTGAAACCATTGAAGAAAACATCGGTTGGATGGATAAGAATTTTGATAAAATCAGAGTGTGGCTGCAAAGTGAAAAGCTTGAACGCATGCATCA

G05-SEQ3.ab1(1>826)

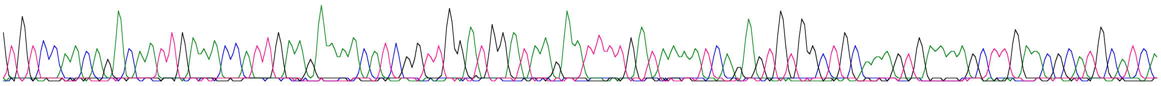

TGTCCAACAGACAATTGAAACCATTGAAGAAAACATCGGTTGGATGGATAAGAATTTTGATAAAATCAGAGTGTGGCTGCAAAGTGAAAAGCTTGAACGCATGCATCA

2,920 2,930 2,940 2,950 2,960 2,970 2,980 2,990 3,000 3,010 3,020

TCACCATCACCATTAACTCGAGGCATGCGGTACCAAGCTTGTCGAGAAGTACTAGAGGATCATAATCAGCCATACCACATTTGTAGAGGTTTTACTTGCTTTAAAAAA

U4947DH210-4.seq(1>2878) TCACCATCACCATTAACTCGAG

G02-pFastbac-R.ab1(1>826)

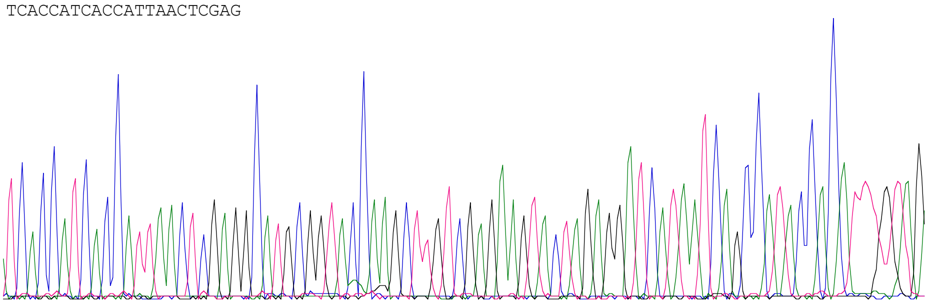

TCACCATCACCATTAACTCGAGGCATGCGGTACCAAGCTTGTCGAGAAGTACTAGAGGATCATAATCAGCCATACCACATTtGtAGA

G05-SEQ3.ab1(1>826)

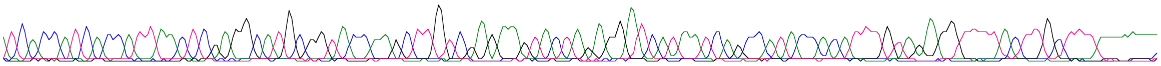

TCACCATCACCATTAACTCGAGGCATGCGGTACCAAGCTTGTCGAGAAGTACTAGAGGATCATAATCAGCCATACCACATTTGTAGAGGTTTTACTTGCTTTAAAAAA

3,030 3,040

CCTCCCACACCTCCCCCTGAA

G05-SEQ3.ab1(1>826)

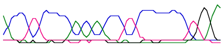

CCTCCCACACCTCCCCCTGAA

10 20 30 40 50 60 70 80 90 100

TTTTGTAATAAAAAAAACCTATAAATATTCCGGATTATTCATACCGTCCCACCATCGGGCGCGGATCCAACTCCTAAAAAAACCGCCACCATGGTGTTTCTGCCCCTCAA

U4947DH210-6.seq(1>2878) GGATCCAACTCCTAAAAAAACCGCCACCATGGTGTTTCTGCCCCTCAA
G10-pFastbac-F.ab1(1>824)

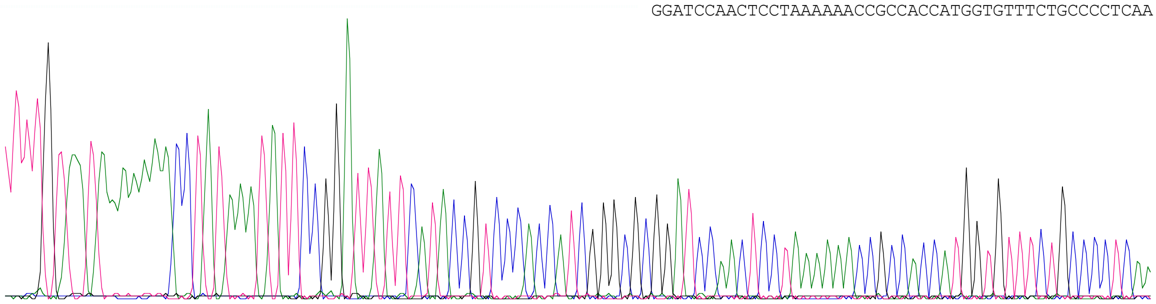

TTTTGTaATAAAAAAAACCTATAAATATTCCGGATTATTCATACCGTCCCACCATCGGGCGCGGATCCAACTCCTAAAAAAACCGCCACCATGGTGTTTCTGCCCCTCAA

G02-SEQ3R.ab1(1>811)

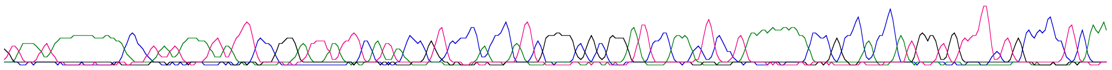

GTAATAAAAAAAACCTATAAATATTCCGGATTATTCATACCGTCCCACCATCGGGCGCGGATCCAACTCCTAAAAAAACCGCCACCATGGTGTTTCTGCCCCTCAA

110 120 130 140 150 160 170 180 190 200 210

ATGGTCCCTTGCAACCATGTCATTTCTACTTTCCTCACTGTTGGCTCTCTTAACTGTGTCCACTCCTTCATGGTGTCAGAGCACTGAAGCATCTCCAAAACGTAGTGA

U4947DH210-6.seq(1>2878) ATGGTCCCTTGCAACCATGTCATTTCTACTTTCCTCACTGTTGGCTCTCTTAACTGTGTCCACTCCTTCATGGTGTCAGAGCACTGAAGCATCTCCAAAACGTAGTGA
G10-pFastbac-F.ab1(1>824)

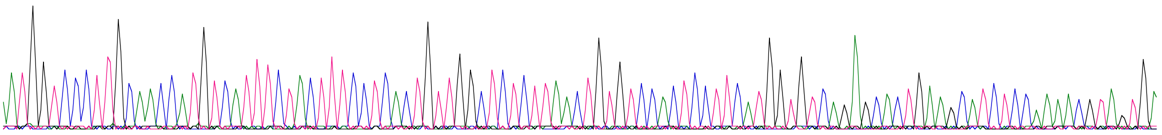

ATGGTCCCTTGCAACCATGTCATTTCTACTTTCCTCACTGTTGGCTCTCTTAACTGTGTCCACTCCTTCATGGTGTCAGAGCACTGAAGCATCTCCAAAACGTAGTGA

G02-SEQ3R.ab1(1>811)

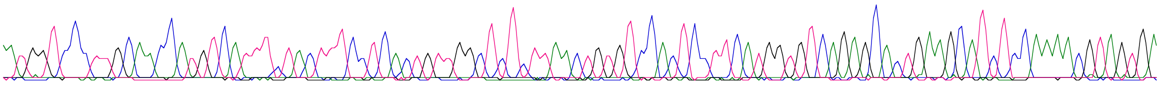

ATGGTCCCTTGCAACCATGTCATTTCTACTTTCCTCACTGTTGGCTCTCTTAACTGTGTCCACTCCTTCATGGTGTCAGAGCACTGAAGCATCTCCAAAACGTAGTGA

220 230 240 250 260 270 280 290 300 310 320

TGGGACACCATTTCCTTGGAATAAAATACGACTTCCTGAGTACGTCATCCCAGTTCATTATGATCTCTTGATCCATGCAAACCTTACCACGCTGACCTTCTGGGGAAC

U4947DH210-6.seq(1>2878) TGGGACACCATTTCCTTGGAATAAAATACGACTTCCTGAGTACGTCATCCCAGTTCATTATGATCTCTTGATCCATGCAAACCTTACCACGCTGACCTTCTGGGGAAC
G10-pFastbac-F.ab1(1>824)

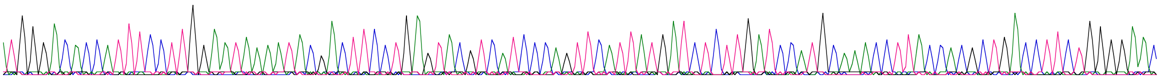

TGGGACACCATTTCCTTGGAATAAAATACGACTTCCTGAGTACGTCATCCCAGTTCATTATGATCTCTTGATCCATGCAAACCTTACCACGCTGACCTTCTGGGGAAC

220 230 240 250 260 270 280 290 300 310 320

TGGGACACCATTTCCTTGGAATAAAATACGACTTCCTGAGTACGTCATCCCAGTTCATTATGATCTCTTGATCCATGCAAACCTTACCACGCTGACCTTCTGGGGAAC

G02-SEQ3R.ab1(1>811)

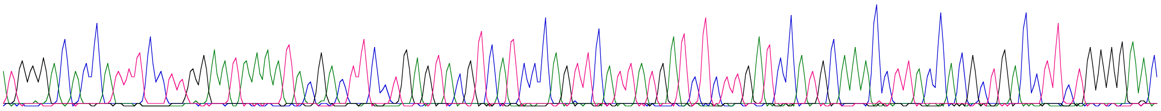

TGGGACACCATTTCCTTGGAATAAAATACGACTTCCTGAGTACGTCATCCCAGTTCATTATGATCTCTTGATCCATGCAAACCTTACCACGCTGACCTTCTGGGGAAC

330 340 350 360 370 380 390 400 410 420 430

CACGAAAGTAGAAATCACAGCCAGTCAGCCCACCAGCACCATCATCCTGCATAGTCACCACCTGCAGATATCTAGGGCCACCCTCAGGAAGGGAGCTGGAGAGAGGCT

U4947DH210-6.seq(1>2878)
G10-pFastbac-F.ab1(1>824)

CACGAAAGTAGAAATCACAGCCAGTCAGCCCACCAGCACCATCATCCTGCATAGTCACCACCTGCAGATATCTAGGGCCACCCTCAGGAAGGGAGCTGGAGAGAGGCT

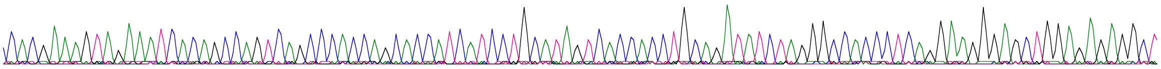

CACGAAAGTAGAAATCACAGCCAGTCAGCCCACCAGCACCATCATCCTGCATAGTCACCACCTGCAGATATCTAGGGCCACCCTCAGGAAGGGAGCTGGAGAGAGGCT

G02-SEQ3R.ab1(1>811)

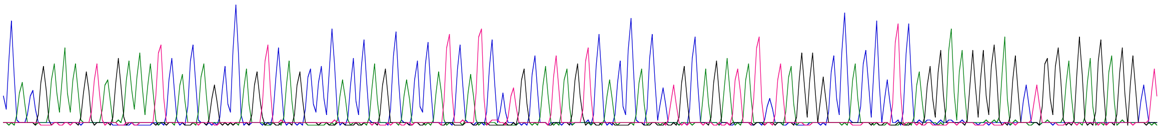

CACGAAAGTAGAAATCACAGCCAGTCAGCCCACCAGCACCATCATCCTGCATAGTCACCACCTGCAGATATCTAGGGCCACCCTCAGGAAGGGAGCTGGAGAGAGGCT

P127R

440 450 460 470 480 490 500 510 520 530 540

ATCGGAAGAACCCCTGCAGGTCCTGGAACACCCGCGTCAGGAGCAAATTGCACTGCTGGCTCCCGAGCCCCTCCTTGTCGGGCTCCCGTACACAGTTGTCATTCACTA

U4947DH210-6.seq(1>2878)
G10-pFastbac-F.ab1(1>824)

ATCGGAAGAACCCCTGCAGGTCCTGGAACACCCGCGTCAGGAGCAAATTGCACTGCTGGCTCCCGAGCCCCTCCTTGTCGGGCTCCCGTACACAGTTGTCATTCACTA

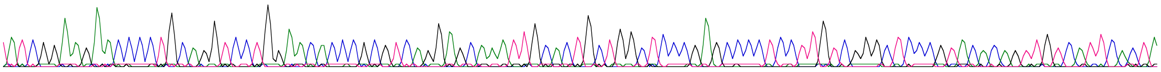

ATCGGAAGAACCCCTGCAGGTCCTGGAACACCCGCGTCAGGAGCAAATTGCACTGCTGGCTCCCGAGCCCCTCCTTGTCGGGCTCCCGTACACAGTTGTCATTCACTA

G02-SEQ3R.ab1(1>811)

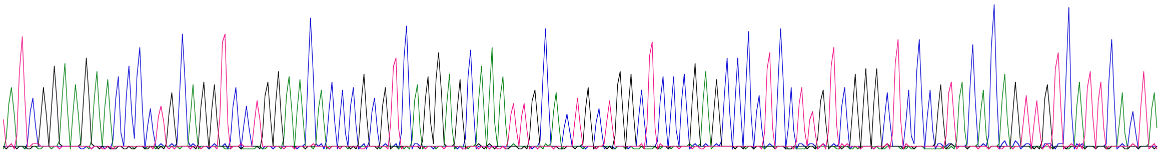

ATCGGAAGAACCCCTGCAGGTCCTGGAACACCCGCGTCAGGAGCAAATTGCACTGCTGGCTCCCGAGCCCCTCCTTGTCGGGCTCCCGTACACAGTTGTCATTCACTA

550 560 570 580 590 600 610 620 630 640

TGCTGGCAATCTTTCGGAGACTTTCCACGGATTTTACAAAAGCACCTACAGAACCAAGGAAGGGGAACTGAGGATACTAGCATCAACACAATTTGAACCCACTGCAGC

U4947DH210-6.seq(1>2878) → TGCTGGCAATCTTTCGGAGACTTTCCACGGATTTTACAAAAGCACCTACAGAACCAAGGAAGGGGAACTGAGGATACTAGCATCAACACAATTTGAACCCACTGCAGC

G10-pFastbac-F.ab1(1>824) →

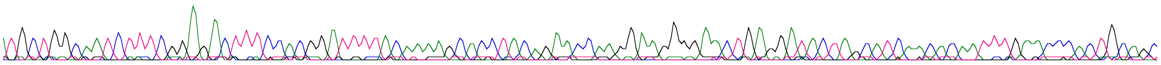

TGCTGGCAATCTTTCGGAGACTTTCCACGGATTTTACAAAAGCACCTACAGAACCAAGGAAGGGGAACTGAGGATACTAGCATCAACACAATTTGAACCCACTGCAGC

G02-SEQ3R.ab1(1>811) ←

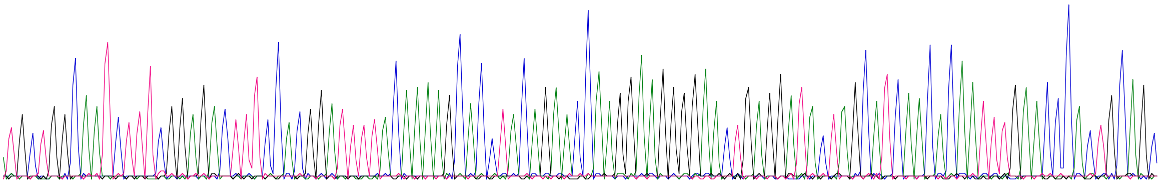

TGCTGGCAATCTTTCGGAGACTTTCCACGGATTTTACAAAAGCACCTACAGAACCAAGGAAGGGGAACTGAGGATACTAGCATCAACACAATTTGAACCCACTGCAGC

650 660 670 680 690 700 710 720 730 740 750

TAGAATGGCCTTTCCCTGCTTTGATGAACCTGCCTTCAAAGCAAGTTTCTCAATCAAAATTAGAAGAGAGCCAAGGCACCTAGCCATCTCCAATATGCCATTGGTGAA

U4947DH210-6.seq(1>2878) → TAGAATGGCCTTTCCCTGCTTTGATGAACCTGCCTTCAAAGCAAGTTTCTCAATCAAAATTAGAAGAGAGCCAAGGCACCTAGCCATCTCCAATATGCCATTGGTGAA

G10-pFastbac-F.ab1(1>824) →

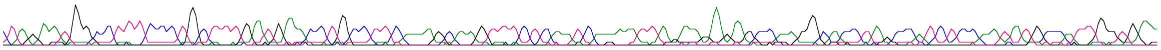

TAGAATGGCCTTTCCCTGCTTTGATGAACCTGCCTTCAAAGCAAGTTTCTCAATCAAAATTAGAAGAGAGCCAAGGCACCTAGCCATCTCCAATATGCCATTGGTGAA

G02-SEQ3R.ab1(1>811) ←

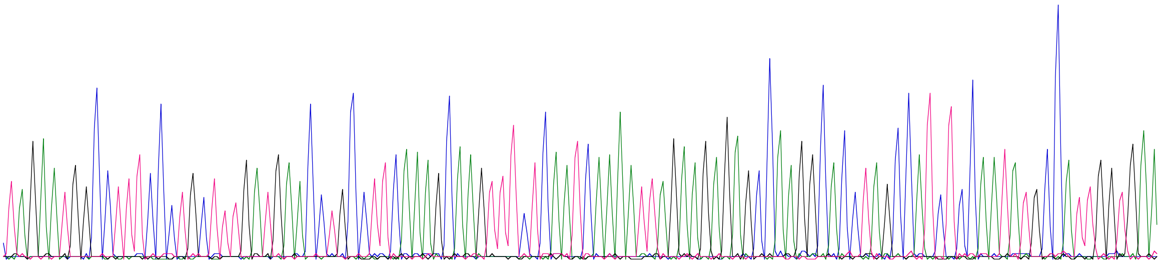

TAGAATGGCCTTTCCCTGCTTTGATGAACCTGCCTTCAAAGCAAGTTTCTCAATCAAAATTAGAAGAGAGCCAAGGCACCTAGCCATCTCCAATATGCCATTGGTGAA

G12-SEQ1.ab1(1>821) →

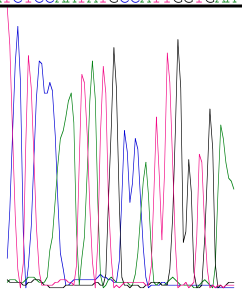

TCTCCaATATGCCATTGGTGAA

G01-SEQ2R.ab1(1>804) ←

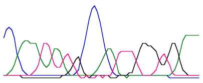

CCAATATGCCATTGGTGAA

760 770 780 790 800 810 820 830 840 850 860

ATCTGTGACTGTTGCTGAAGGACTCATAGAAGACCATTTTGATGTCACTGTGAAGATGAGCACCTATCTGGTGGCCTTCATCATTTCAGATTTTGAGTCTGTCAGCAA

U4947DH210-6.seq(1>2878) ATCTGTGACTGTTGCTGAAGGACTCATAGAAGACCATTTTGATGTCACTGTGAAGATGAGCACCTATCTGGTGGCCTTCATCATTTCAGATTTTGAGTCTGTCAGCAA

G10-pFastbac-F.ab1(1>824)

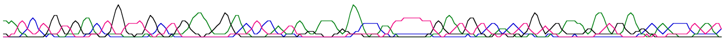

ATCTGTGACTGTTGCTGAAGGACTCATAGAAGACCATTTTGATGTCACTGTGAAGATGAGCACCTATC

G02-SEQ3R.ab1(1>811)

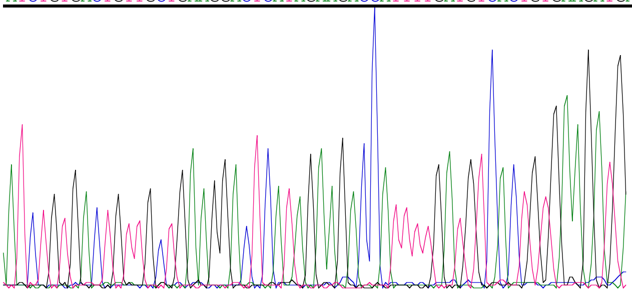

ATCTGTGACTGTTGCTGAAGGACTCATAGAAGACCATTTTGATGTCACTGTGAAGATGA

G12-SEQ1.ab1(1>821)

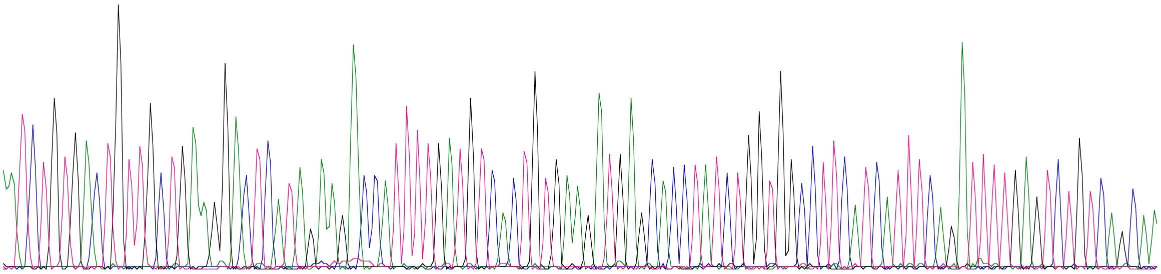

ATCTGTGACTGTTGCTGAAGGACTCATAGAAGACCATTTTGATGTCACTGTGAAGATGAGCACCTATCTGGTGGCCTTCATCATTTCAGATTTTGAGTCTGTCAGCAA

G01-SEQ2R.ab1(1>804)

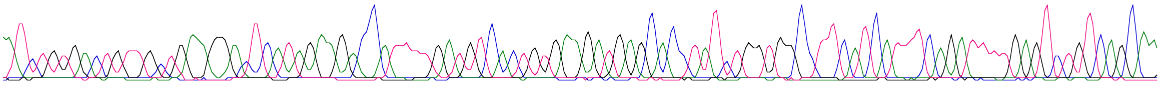

ATCTGTGACTGTTGCTGAAGGACTCATAGAAGACCATTTTGATGTCACTGTGAAGATGAGCACCTATCTGGTGGCCTTCATCATTTCAGATTTTGAGTCTGTCAGCAA

870 880 890 900 910 920 930 940 950 960 970

GATAACCAAGAGTGGAGTCAAGGTTTCTGTTTATGCTGTGCCAGACAAGATAAATCAAGCAGATTATGCACTGGATGCTGCGGTGACTCTTCTAGAATTTTATGAGGA

U4947DH210-6.seq(1>2878) GATAACCAAGAGTGGAGTCAAGGTTTCTGTTTATGCTGTGCCAGACAAGATAAATCAAGCAGATTATGCACTGGATGCTGCGGTGACTCTTCTAGAATTTTATGAGGA

G12-SEQ1.ab1(1>821)

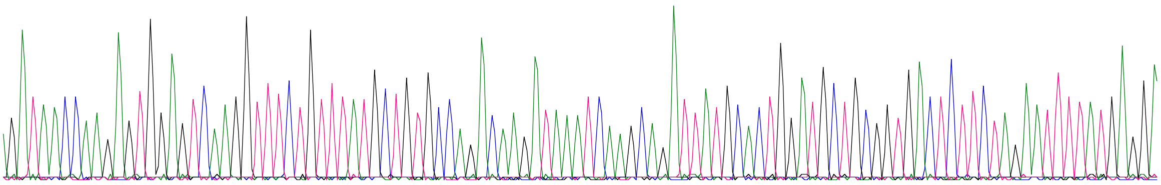

GATAACCAAGAGTGGAGTCAAGGTTTCTGTTTATGCTGTGCCAGACAAGATAAATCAAGCAGATTATGCACTGGATGCTGCGGTGACTCTTCTAGAATTTTATGAGGA

G01-SEQ2R.ab1(1>804)

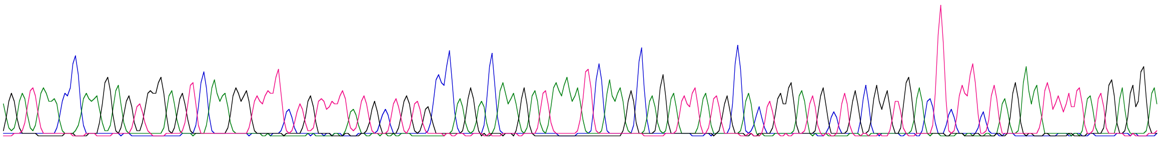

GATAACCAAGAGTGGAGTCAAGGTTTCTGTTTATGCTGTGCCAGACAAGATAAATCAAGCAGATTATGCACTGGATGCTGCGGTGACTCTTCTAGAATTTTATGAGGA

980 990 1,000 1,010 1,020 1,030 1,040 1,050 1,060 1,070 1,080

TTATTTCAGCATACCGTATCCCCTACCCAAACAAGATCTTGCTGCTATTCCCGACTTTCAGTCTGGTGCTATGGAAAACTGGGGACTGACAACATATAGAGAATCTGC

U4947DH210-6.seq(1>2878) → TTATTTCAGCATACCGTATCCCCTACCCAAACAAGATCTTGCTGCTATTCCCGACTTTCAGTCTGGTGCTATGGAAAACTGGGGACTGACAACATATAGAGAATCTGC
G12-SEQ1.ab1(1>821) →

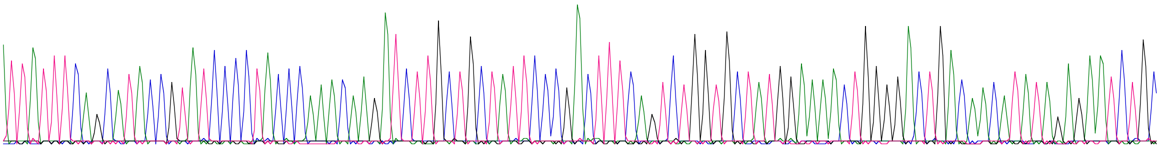

TTATTTCAGCATACCGTATCCCCTACCCAAACAAGATCTTGCTGCTATTCCCGACTTTCAGTCTGGTGCTATGGAAAACTGGGGACTGACAACATATAGAGAATCTGC

G01-SEQ2R.ab1(1>804) ←

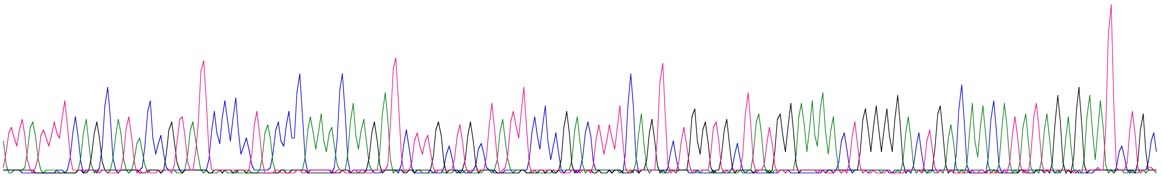

TTATTTCAGCATACCGTATCCCCTACCCAAACAAGATCTTGCTGCTATTCCCGACTTTCAGTCTGGTGCTATGGAAAACTGGGGACTGACAACATATAGAGAATCTGC

G346D

1,090 1,100 1,110 1,120 1,130 1,140 1,150 1,160 1,170 1,180

TCTGTTGTTTGATGCAGAAAAGTCTTCTGCATCAAGTAAGCTTGACATCACAATGACTGTGGCCCATGAACTGGCTCACCAGTGGTTTGGGAACCTGGTCACTATGGA

U4947DH210-6.seq(1>2878) → TCTGTTGTTTGATGCAGAAAAGTCTTCTGCATCAAGTAAGCTTGACATCACAATGACTGTGGCCCATGAACTGGCTCACCAGTGGTTTGGGAACCTGGTCACTATGGA
G12-SEQ1.ab1(1>821) →

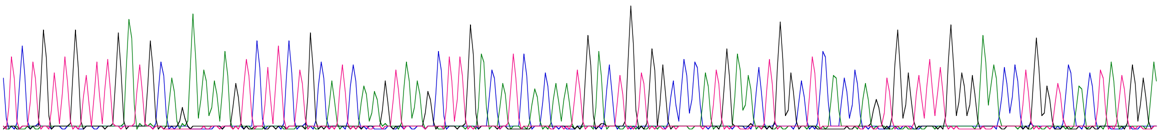

TCTGTTGTTTGATGCAGAAAAGTCTTCTGCATCAAGTAAGCTTGACATCACAATGACTGTGGCCCATGAACTGGCTCACCAGTGGTTTGGGAACCTGGTCACTATGGA

G01-SEQ2R.ab1(1>804) ←

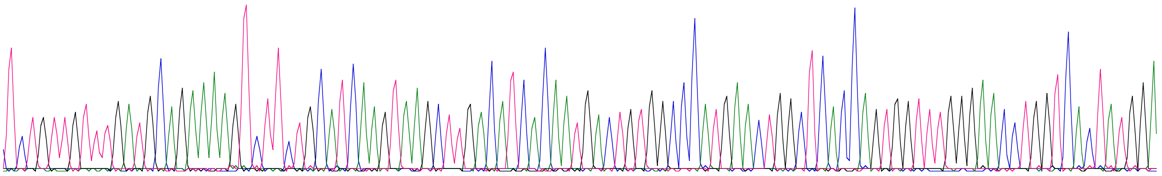

TCTGTTGTTTGATGCAGAAAAGTCTTCTGCATCAAGTAAGCTTGACATCACAATGACTGTGGCCCATGAACTGGCTCACCAGTGGTTTGGGAACCTGGTCACTATGGA

1,190 1,200 1,210 1,220 1,230 1,240 1,250 1,260 1,270 1,280 1,290

ATGGTGGAATGATCTTTGGCTAAATGAAGGATTTGCCAAATTTATGGAGTTTGTGTCTGTCAGTGTGACCCATCCTGAACTGAAAGTTGGAGATTATTTCTTTGGCAA

U4947DH210-6.seq(1>2878) → ATGGTGGAATGATCTTTGGCTAAATGAAGGATTTGCCAAATTTATGGAGTTTGTGTCTGTCAGTGTGACCCATCCTGAACTGAAAGTTGGAGATTATTTCTTTGGCAA
G12-SEQ1.ab1(1>821) →

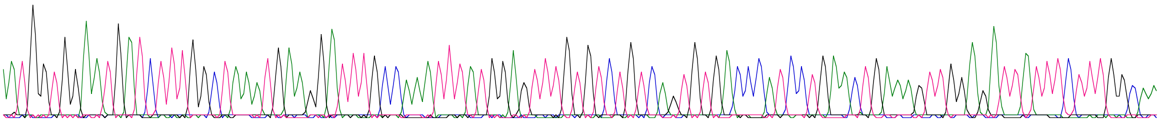

ATGGTGGAATGATCTTTGGCTAAATGAAGGATTTGCCAAATTTATGGAGTTTGTGTCTGTCAGTGTGACCCATCCTGAACTGAAAGTTGGAGATTATTTCTTTGGCAA

1,190 1,200 1,210 1,220 1,230 1,240 1,250 1,260 1,270 1,280 1,290

ATGGTGGAATGATCTTTGGCTAAATGAAGGATTTGCCAAATTTATGGAGTTTGTGTCTGTCAGTGTGACCCATCCTGAACTGAAAGTTGGAGATTATTTCTTTGGCAA

G01-SEQ2R.ab1(1>804) ←

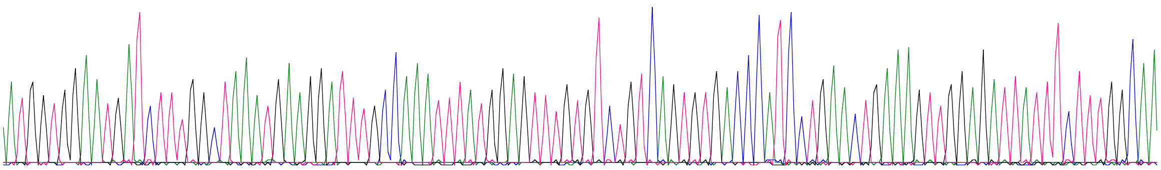

ATGGTGGAATGATCTTTGGCTAAATGAAGGATTTGCCAAATTTATGGAGTTTGTGTCTGTCAGTGTGACCCATCCTGAACTGAAAGTTGGAGATTATTTCTTTGGCAA

1,300 1,310 1,320 1,330 1,340 1,350 1,360 1,370 1,380 1,390 1,400

ATGTTTTGACGCAATGGAGGTAGATGCTTTAAATTCCTCACACCCTGTGTCTACACCTGTGGAAAATCCTGCTCAGATCCGGGAGATGTTTGATGATGTTTCTTATGA

U4947DH210-6.seq(1>2878) →
G12-SEQ1.ab1(1>821) →

ATGTTTTGACGCAATGGAGGTAGATGCTTTAAATTCCTCACACCCTGTGTCTACACCTGTGGAAAATCCTGCTCAGATCCGGGAGATGTTTGATGATGTTTCTTATGA

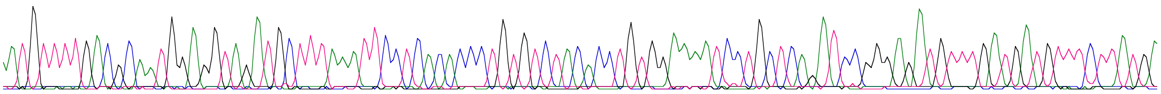

ATGTTTTGACGCAATGGAGGTAGATGCTTTAAATTCCTCACACCCTGTGTCTACACCTGTGGAAAATCCTGCTCAGATCCGGGAGATGTTTGATGATGTTTCTTATGA

G01-SEQ2R.ab1(1>804) ←

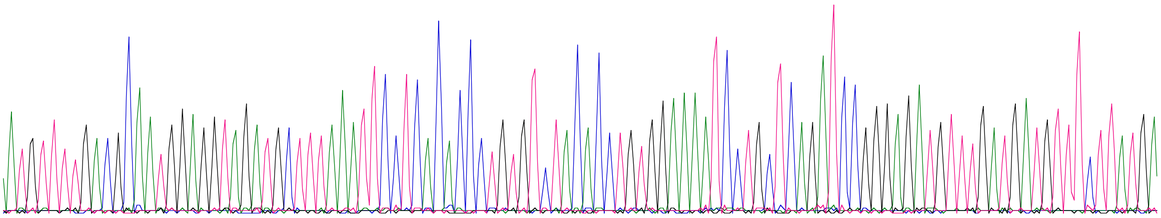

ATGTTTTGACGCAATGGAGGTAGATGCTTTAAATTCCTCACACCCTGTGTCTACACCTGTGGAAAATCCTGCTCAGATCCGGGAGATGTTTGATGATGTTTCTTATGA

1,410 1,420 1,430 1,440 1,450 1,460 1,470 1,480 1,490 1,500 1,510

TAAGGGAGCTTGTATTCTGAATATGCTAAGGGAGTATCTTAGTGCTGACGCATTTAAAAGTGGTATTGTACAGTATCTCCAGAAGCATAGCTATAAAAATACAAAAAA

U4947DH210-6.seq(1>2878) →
G12-SEQ1.ab1(1>821) →

TAAGGGAGCTTGTATTCTGAATATGCTAAGGGAGTATCTTAGTGCTGACGCATTTAAAAGTGGTATTGTACAGTATCTCCAGAAGCATAGCTATAAAAATACAAAAAA

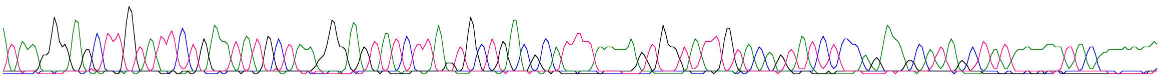

TAAGGGAGCTTGTATTCTGAATATGCTAAGGGAGTATCTTAGTGCTGACGCATTTAAAAGTGGTATTGTACAGTATCTCCAGAAGCATAGCTATAAAAATACAAAAAA

G01-SEQ2R.ab1(1>804) ←

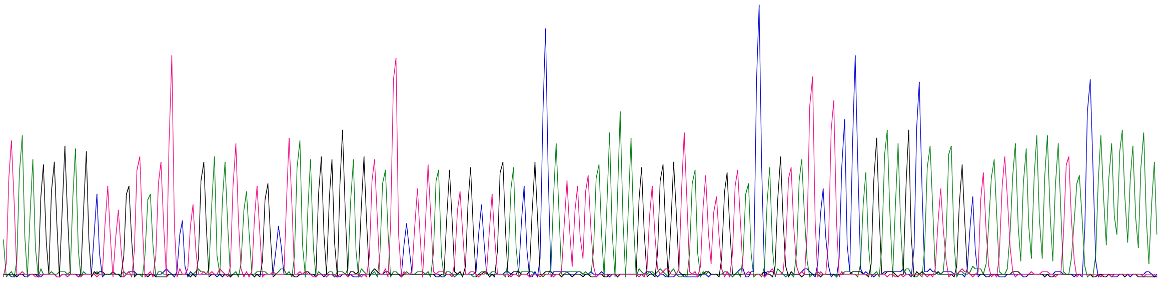

TAAGGGAGCTTGTATTCTGAATATGCTAAGGGAGTATCTTAGTGCTGACGCATTTAAAAGTGGTATTGTACAGTATCTCCAGAAGCATAGCTATAAAAATACAAAAAA

1,410 1,420 1,430 1,440 1,450 1,460 1,470 1,480 1,490 1,500 1,510

TAAGGGAGCTTGTATTCTGAATATGCTAAGGGAGTATCTTAGTGCTGACGCATTTAAAAGTGGTATTGTACAGTATCTCCAGAAGCATAGCTATAAAAATACAAAAAA

G07-SEQ1R.ab1(1>824)

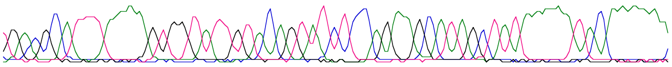

TGACGCATTTAAAAGTGGTATTGTACAGTATCTCCAGAAGCATAGCTATAAAAATACAAAAAA

H01-SEQ2.ab1(1>817)

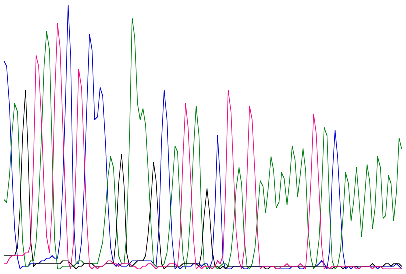

CAGTATCTCCAGAAGCATAGCTATAAAAATACAAAAAA

1,520 1,530 1,540 1,550 1,560 1,570 1,580 1,590 1,600 1,610 1,620

CGAGGACCTGTGGGATAGTATGGCAAGTATTTGCCCTACAGATGGTGTAAAAGGGATGGATGGCTTTTGCTCTAGAAGTCAACATTCATCTTCATCCTCACATTGGCA

U4947DH210-6.seq(1>2878) CGAGGACCTGTGGGATAGTATGGCAAGTATTTGCCCTACAGATGGTGTAAAAGGGATGGATGGCTTTTGCTCTAGAAGTCAACATTCATCTTCATCCTCACATTGGCA
G12-SEQ1.ab1(1>821)

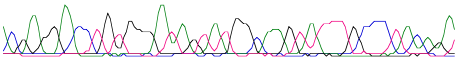

CGAGGACCTGTGGGATAGTATGGCAAGTATTTGCCCTACAGAT

G01-SEQ2R.ab1(1>804)

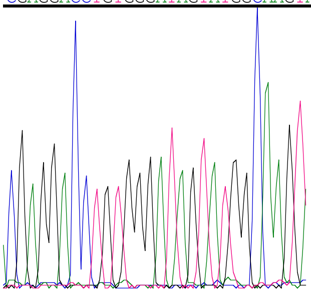

CGAGGACCTGTGGGATAGTATGGCAAGTA

G07-SEQ1R.ab1(1>824)

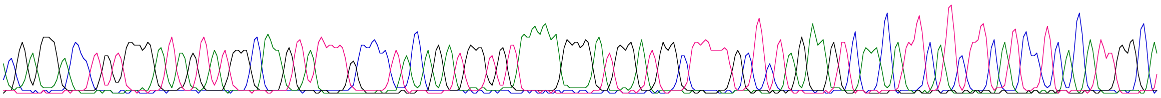

CGAGGACCTGTGGGATAGTATGGCAAGTATTTGCCCTACAGATGGTGTAAAAGGGATGGATGGCTTTTGCTCTAGAAGTCAACATTCATCTTCATCCTCACATTGGCA

H01-SEQ2.ab1(1>817)

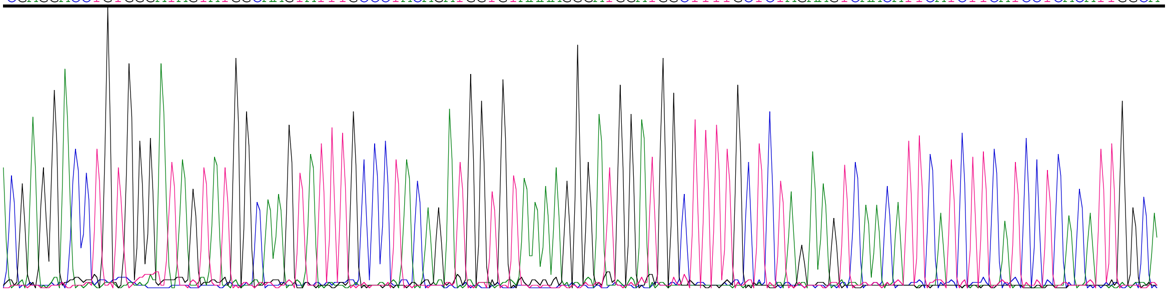

CGAGGACCTGTGGGATAGTATGGCAAGTATTTGCCCTACAGATGGTGTAAAAGGGATGGATGGcTTTTGCTCTAGAAGTCAACATTCATCTTCATCCTCACATTGGCA

K528R

1,630 1,640 1,650 1,660 1,670 1,680 1,690 1,700 1,710 1,720

TCAGGAAGGGGTGGATGTGAAAACCATGATGAACACTTGGACACTGCAGCGTGGTTTTCCCCTAATAACCATCACAGTGAGGGGGAGGAATGTACACATGAAGCAAGA

U4947DH210-6.seq(1>2878) TCAGGAAGGGGTGGATGTGAAAACCATGATGAACACTTGGACACTGCAGCGTGGTTTTCCCCTAATAACCATCACAGTGAGGGGGAGGAATGTACACATGAAGCAAGA

G07-SEQ1R.ab1(1>824)

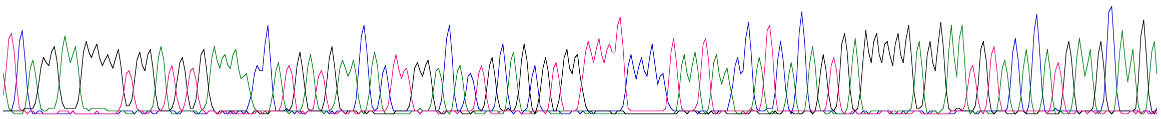

TCAGGAAGGGGTGGATGTGAAAACCATGATGAACACTTGGACACTGCAGCGTGGTTTTCCCCTAATAACCATCACAGTGAGGGGGAGGAATGTACACATGAAGCAAGA

H01-SEQ2.ab1(1>817)

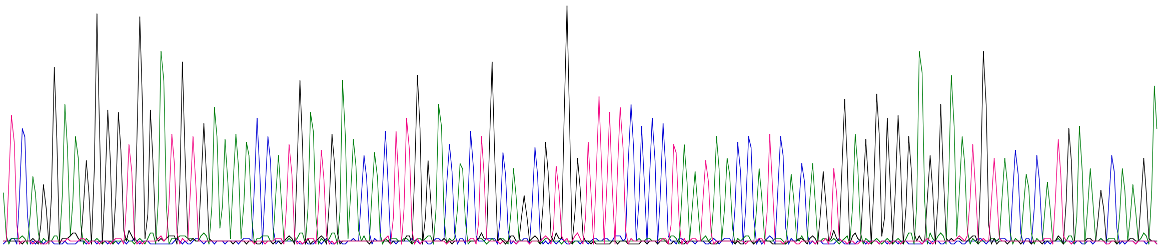

TCAGGAAGGGGTGGATGTGAAAACCATGATGAACACTTGGACACTGCAGCGTGGTTTTCCCCTAATAACCATCACAGTGAGGGGGAGGAATGTACACATGAAGCAAGA

1,730 1,740 1,750 1,760 1,770 1,780 1,790 1,800 1,810 1,820 1,830

GCACTACATGAAGGGCTCTGACGGCGCCCCGGACACTGGGTACCTGTGGCATGTTCCATTGACATTCATCACCAGCAAATCCGACATGGTCCATCGATTTTTGCTAAA

U4947DH210-6.seq(1>2878) GCACTACATGAAGGGCTCTGACGGCGCCCCGGACACTGGGTACCTGTGGCATGTTCCATTGACATTCATCACCAGCAAATCCGACATGGTCCATCGATTTTTGCTAAA

G07-SEQ1R.ab1(1>824)

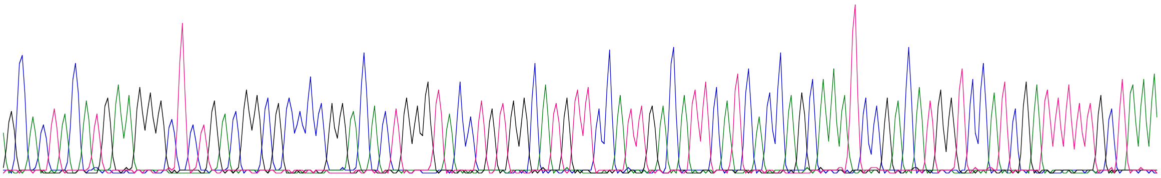

GCACTACATGAAGGGCTCTGACGGCGCCCCGGACACTGGGTACCTGTGGCATGTTCCATTGACATTCATCACCAGCAAATCCGACATGGTCCATCGATTTTTGCTAAA

H01-SEQ2.ab1(1>817)

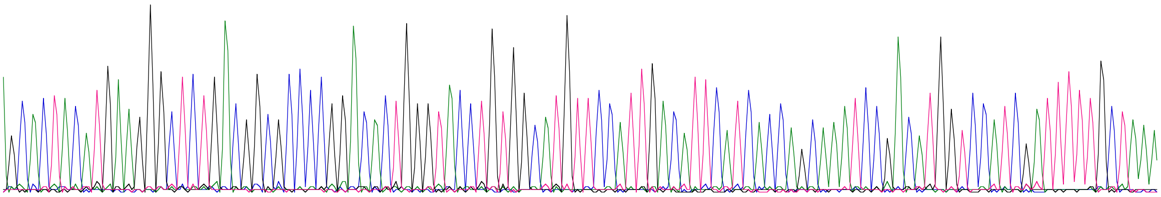

GCACTACATGAAGGGCTCTGACGGCGCCCCGGACACTGGGTACCTGTGGCATGTTCCATTGACATTCATCACCAGCAAATCCGACATGGTCCATCGaTTTTTGCTAAA

1,840 1,850 1,860 1,870 1,880 1,890 1,900 1,910 1,920 1,930 1,940

AACAAAAACAGATGTGCTCATCCTCCCAGAAGAGGTGGAATGGATCAAATTTAATGTGGGCATGAATGGCTATTACATTGTGCATTACGAGGATGATGGATGGGACTC

U4947DH210-6.seq(1>2878) AACAAAAACAGATGTGCTCATCCTCCCAGAAGAGGTGGAATGGATCAAATTTAATGTGGGCATGAATGGCTATTACATTGTGCATTACGAGGATGATGGATGGGACTC

G07-SEQ1R.ab1(1>824)

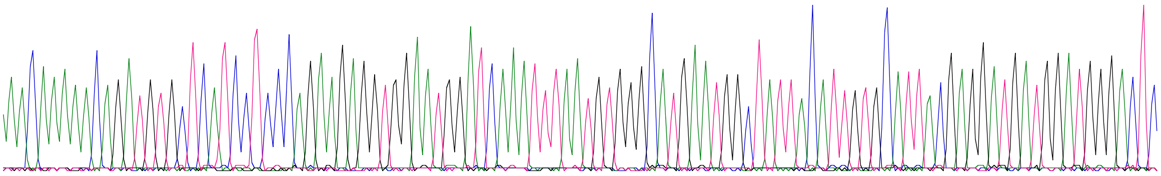

AACAAAAACAGATGTGCTCATCCTCCCAGAAGAGGTGGAATGGATCAAATTTAATGTGGGCATGAATGGCTATTACATTGTGCATTACGAGGATGATGGATGGGACTC

1,840 1,850 1,860 1,870 1,880 1,890 1,900 1,910 1,920 1,930 1,940

AACAAAAACAGATGTGCTCATCCTCCCAGAAGAGGTGGAATGGATCAAATTTAATGTGGGCATGAATGGCTATTACATTGTGCATTACGAGGATGATGGATGGGACTC

H01-SEQ2.ab1(1>817)

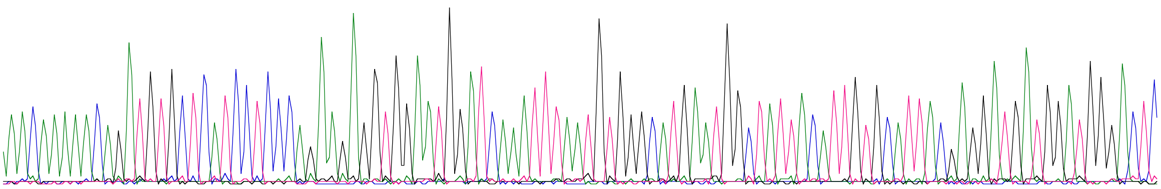

AACAAAAACAGATGTGCTCATCCTCCCAGAAGAGGTGGAATGGATCAAATTTAATGTGGGCATGAATGGCTATTACATTGTGCATTACGAGGATGATGGATGGGACTC

1,950 1,960 1,970 1,980 1,990 2,000 2,010 2,020 2,030 2,040 2,050

TTTGACTGGCCTTTTAAAAGGAACACACACAGCAGTCAGCAGTAATGATCGGGCGAGTCTCATTAACAATGCATTTCAGCTCGTCAGCATTGGGAAGCTGTCCATTGA

U4947DH210-6.seq(1>2878) TTTGACTGGCCTTTTAAAAGGAACACACACAGCAGTCAGCAGTAATGATCGGGCGAGTCTCATTAACAATGCATTTCAGCTCGTCAGCATTGGGAAGCTGTCCATTGA

G07-SEQ1R.ab1(1>824)

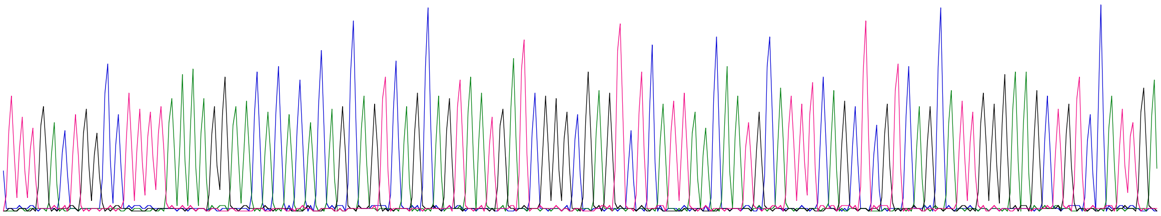

TTTGACTGGCCTTTTAAAAGGAACACACACAGCAGTCAGCAGTAATGATCGGGCGAGTCTCATTAACAATGCATTTCAGCTCGTCAGCATTGGGAAGCTGTCCATTGA

H01-SEQ2.ab1(1>817)

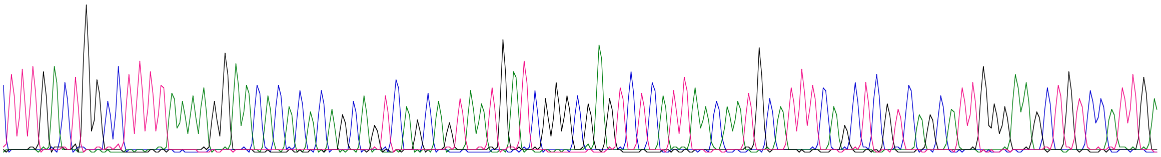

TTTGACTGGCCTTTTAAAAGGAACACACACAGCAGTCAGCAGTAATGATCGGGCGAGTCTCATTAACAATGCATTTCAGCTCGTCAGCATTGGGAAGCTGTCCATTGA

2,060 2,070 2,080 2,090 2,100 2,110 2,120 2,130 2,140 2,150 2,160

AAAGGCCTTGGATTTATCCCTGTACTTGAAACATGAAACTGAAATTATGCCCGTGTTTCAAGGTTTGAATGAGCTGATTCCTATGTATAAGTTAATGGAGAAAAGAGA

U4947DH210-6.seq(1>2878) AAAGGCCTTGGATTTATCCCTGTACTTGAAACATGAAACTGAAATTATGCCCGTGTTTCAAGGTTTGAATGAGCTGATTCCTATGTATAAGTTAATGGAGAAAAGAGA

G07-SEQ1R.ab1(1>824)

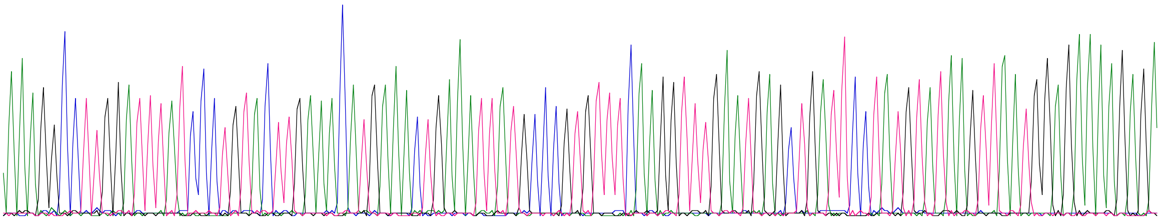

AAAGGCCTTGGATTTATCCCTGTACTTGAAACATGAAACTGAAATTATGCCCGTGTTTCAAGGTTTGAATGAGCTGATTCCTATGTATAAGTTAATGGAGAAAAGAGA

H01-SEQ2.ab1(1>817)

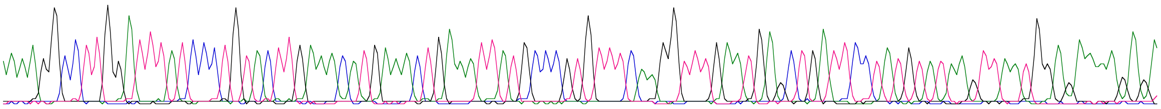

AAAGGCCTTGGATTTATCCCTGTACTTGAAACATGAAACTGAAATTATGCCCGTGTTTCAAGGTTTGAATGAGCTGATTCCTATGTATAAGTTAATGGAGAAAAGAGA

2,170 2,180 2,190 2,200 2,210 2,220 2,230 2,240 2,250 2,260

TATGAATGAAGTGGAAACTCAATTCAAGGCCTTCCTCATCAGGCTGCTAAGGGACCTCATTGATAAGCAGACATGGACAGACGAGGGCTCAGTCTCAGAGCGAATGCT

U4947DH210-6.seq(1>2878) TATGAATGAAGTGGAAACTCAATTCAAGGCCTTCCTCATCAGGCTGCTAAGGGACCTCATTGATAAGCAGACATGGACAGACGAGGGCTCAGTCTCAGAGCGAATGCT

G07-SEQ1R.ab1(1>824)

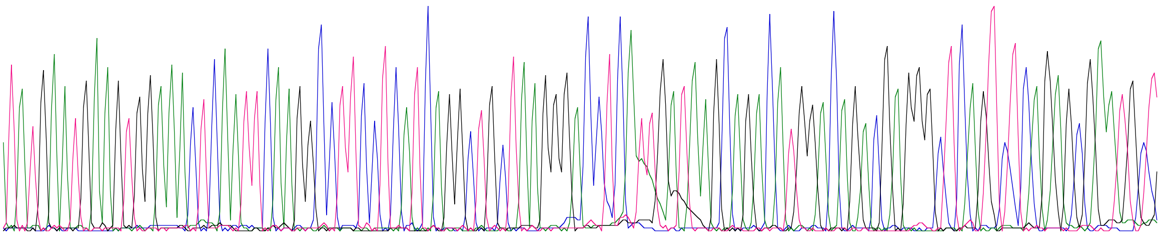

TATGAATGAAGTGGAAACTCAATTCAAGGCCTTCCTCATCAGGCTGCTAAGGGACCTCAtTGaTAAGCAGACATGGACAGACGAGGGCTCAGTCTCAGAGCGAATGCT

H01-SEQ2.ab1(1>817)

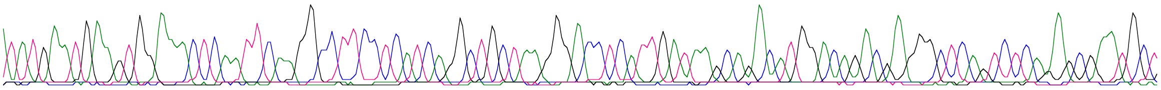

TATGAATGAAGTGGAAACTCAATTCAAGGCCTTCCTCATCAGGCTGCTAAGGGACCTCATTGATAAGCAGACATGGACAGACGAGGGCTCAGTCTCAGAGCGAATGCT

G11-pFastbac-R.ab1(1>813)

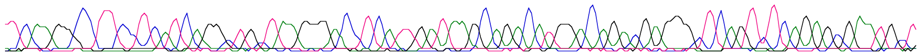

TTCAAGGCCTTCCTCATCAGGCTGCTAAGGGACCTCATTGATAAGCAGACATGGACAGACGAGGGCTCAGTCTCAGAGCGAATGCT

H02-SEQ3.ab1(1>825)

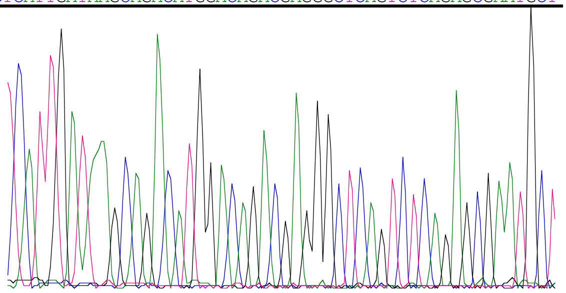

TCATTGATAAGCAGACATGGACAGACGAGGGCTCAGTCTCAGAGCGAATGCT

2,270 2,280 2,290 2,300 2,310 2,320 2,330 2,340 2,350 2,360 2,370

GCGGAGTGAGCTACTACTCCTCGCCTGTGTGCACAACTATCAGCCGTGCGTACAGAGGGCAGAAGGCTATTTCAGAAAGTGGAAGGAATCCAATGGAAACTTGAGCCT

U4947DH210-6.seq(1>2878) GCGGAGTGAGCTACTACTCCTCGCCTGTGTGCACAACTATCAGCCGTGCGTACAGAGGGCAGAAGGCTATTTCAGAAAGTGGAAGGAATCCAATGGAAACTTGAGCCT

G07-SEQ1R.ab1(1>824)

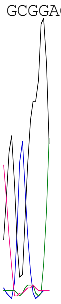

GCGGA

H01-SEQ2.ab1(1>817)

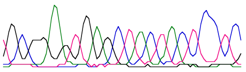

GCGGAGTGAGCTACTACTCCTCG

Q730E

2,270 2,280 2,290 2,300 2,310 2,320 2,330 2,340 2,350 2,360 2,370

GCGGAGTGAGCTACTACTCCTCGCCTGTGTGCACAACTATCAGCCGTGCGTACAGAGGGCAGAAGGCTATTTCAGAAAGTGGAAGGAATCCAATGGAAACTTGAGCCT

G11-pFastbac-R.ab1(1>813)

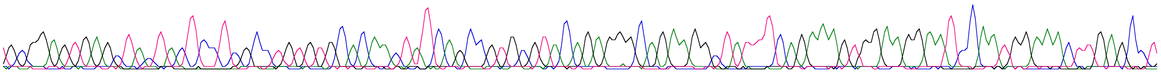

GCGGAGTGAGCTACTACTCCTCGCCTGTGTGCACAACTATCAGCCGTGCGTACAGAGGGCAGAAGGCTATTTCAGAAAGTGGAAGGAATCCAATGGAAACTTGAGCCT

H02-SEQ3.ab1(1>825)

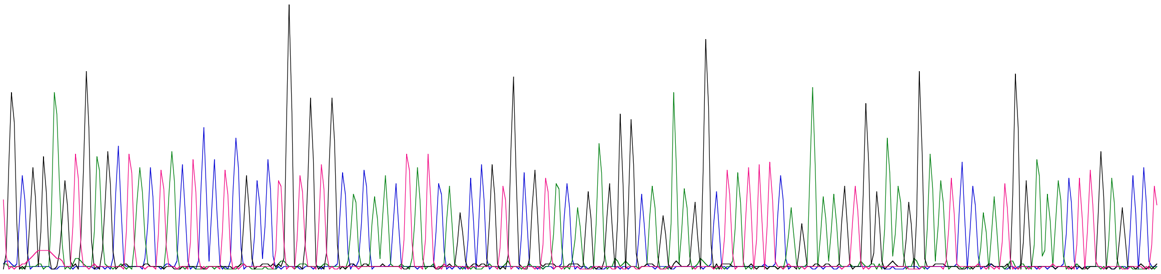

GCGGAGTGAGCTACTACTCCTCGCCTGTGTGCACAACTATCAGCCGTGCGTACAGAGGGCAGAAGGCTATTTCAGAAAGTGGAAGGAATCCAATGGAAACTTGAGCCT

2,380 2,390 2,400 2,410 2,420 2,430 2,440 2,450 2,460 2,470 2,480

GCCTGTCGACGTGACCTTGGCAGTGTTTGCTGTGGGGGCCCAGAGCACAGAAGGCTGGGATTTTCTTTATAGTAAATATCAGTTTTCTTTGTCCAGTACTGAGAAAAG

U4947DH210-6.seq(1>2878)

GCCTGTCGACGTGACCTTGGCAGTGTTTGCTGTGGGGGCCCAGAGCACAGAAGGCTGGGATTTTCTTTATAGTAAATATCAGTTTTCTTTGTCCAGTACTGAGAAAAG

G11-pFastbac-R.ab1(1>813)

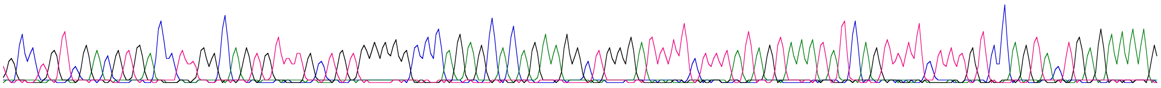

GCCTGTCGACGTGACCTTGGCAGTGTTTGCTGTGGGGGCCCAGAGCACAGAAGGCTGGGATTTTCTTTATAGTAAATATCAGTTTTCTTTGTCCAGTACTGAGAAAAG

H02-SEQ3.ab1(1>825)

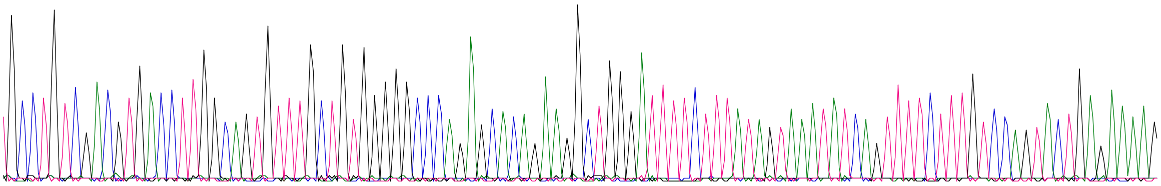

GCCTGTCGACGTGACCTTGGCAGTGTTTGCTGTGGGGGCCCAGAGCACAGAAGGCTGGGATTTTCTTTATAGTAAATATCAGTTTTCTTTGTCCAGTACTGAGAAAAG

2,490 2,500 2,510 2,520 2,530 2,540 2,550 2,560 2,570 2,580 2,590

CCAAATTGAATTTGCCCTCTGCAGAACCCAAAATAAGGAAAAGCTTCAATGGCTACTAGATGAAAGCTTTAAGGGAGATAAAATAAAAACTCAGGAGTTTCCACAAAT

U4947DH210-6.seq(1>2878)

CCAAATTGAATTTGCCCTCTGCAGAACCCAAAATAAGGAAAAGCTTCAATGGCTACTAGATGAAAGCTTTAAGGGAGATAAAATAAAAACTCAGGAGTTTCCACAAAT

G11-pFastbac-R.ab1(1>813)

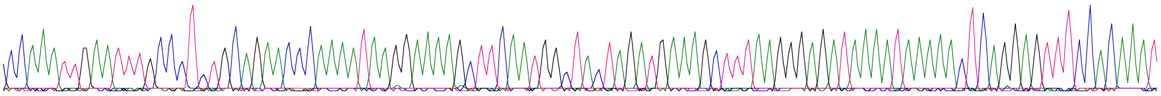

CCAAATTGAATTTGCCCTCTGCAGAACCCAAAATAAGGAAAAGCTTCAATGGCTACTAGATGAAAGCTTTAAGGGAGATAAAATAAAAACTCAGGAGTTTCCACAAAT

2,490 2,500 2,510 2,520 2,530 2,540 2,550 2,560 2,570 2,580 2,590

CCAAATTGAATTTGCCCTCTGCAGAACCCAAAATAAGGAAAAGCTTCAATGGCTACTAGATGAAAGCTTTAAGGGAGATAAAATAAAAACTCAGGAGTTTCCACAAAT

H02-SEQ3.ab1(1>825)

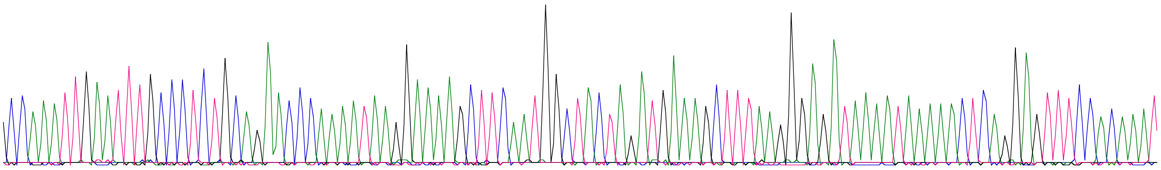

2,600 2,610 2,620 2,630 2,640 2,650 2,660 2,670 2,680 2,690 2,700

TCTTACACTCATTGGCAGGAACCCAGTAGGATACCCACTGGCCTGGCAATTTCTGAGGAAAAACTGGAACAAACTTGTACAAAAGTTTGAACTTGGCTCATCTTCCAT

U4947DH210-6.seq(1>2878) TCTTACACTCATTGGCAGGAACCCAGTAGGATACCCACTGGCCTGGCAATTTCTGAGGAAAAACTGGAACAAACTTGTACAAAAGTTTGAACTTGGCTCATCTTCCAT

G11-pFastbac-R.ab1(1>813)

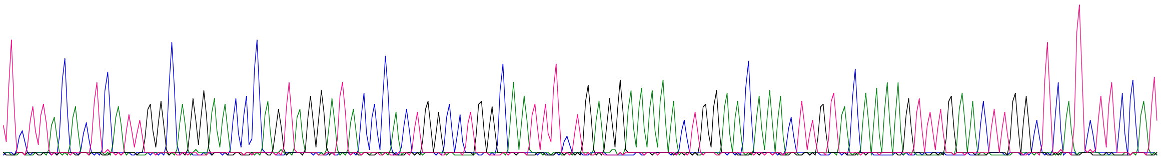

H02-SEQ3.ab1(1>825)

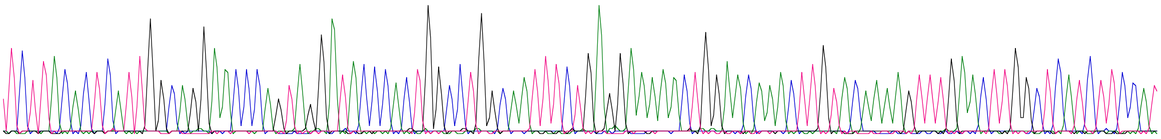

2,710 2,720 2,730 2,740 2,750 2,760 2,770 2,780 2,790 2,800

AGCCCACATGGTAATGGGTACAACAAATCAATTCTCCACAAGAACACGGCTTGAAGAGGTAAAAGGATTCTTCAGCTCTTTGAAAGAAAATGGTTCTCAGCTCCGTTG

U4947DH210-6.seq(1>2878) AGCCCACATGGTAATGGGTACAACAAATCAATTCTCCACAAGAACACGGCTTGAAGAGGTAAAAGGATTCTTCAGCTCTTTGAAAGAAAATGGTTCTCAGCTCCGTTG

G11-pFastbac-R.ab1(1>813)

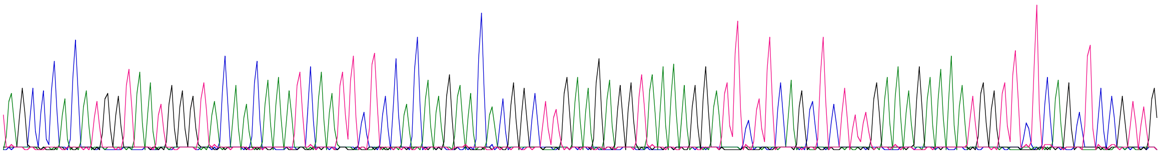

H02-SEQ3.ab1(1>825)

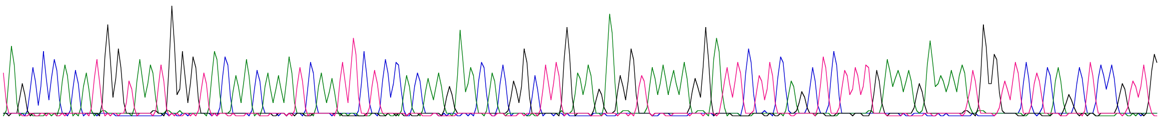

2,810 2,820 2,830 2,840 2,850 2,860 2,870 2,880 2,890 2,900 2,910

TGTCCAACAGACAATTGAAACCATTGAAGAAAACATCGGTTGGATGGATAAGAATTTTGATAAAATCAGAGTGTGGCTGCAAAGTGAAAAGCTTGAACGCATGCATCA

U4947DH210-6.seq(1>2878) TGTCCAACAGACAATTGAAACCATTGAAGAAAACATCGGTTGGATGGATAAGAATTTTGATAAAATCAGAGTGTGGCTGCAAAGTGAAAAGCTTGAACGCATGCATCA
G11-pFastbac-R.ab1(1>813)

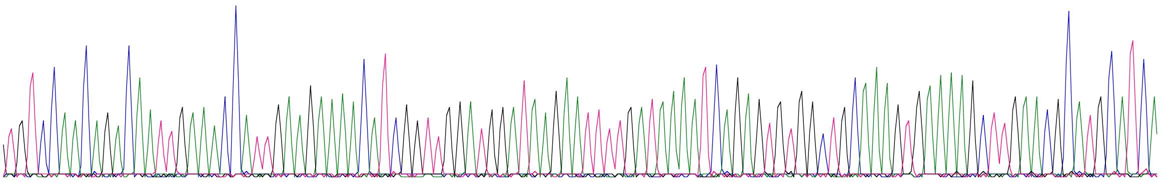

TGTCCAACAGACAATTGAAACCATTGAAGAAAACATCGGTTGGATGGATAAGAATTTTGATAAAATCAGAGTGTGGCTGCAAAGTGAAAAGCTTGAACGCATGCATCA

H02-SEQ3.ab1(1>825)

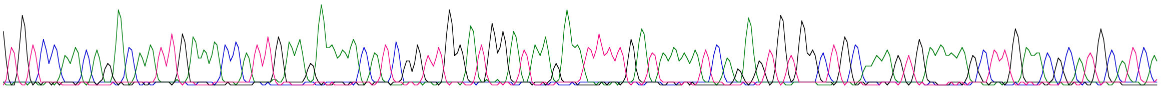

TGTCCAACAGACAATTGAAACCATTGAAGAAAACATCGGTTGGATGGATAAGAATTTTGATAAAATCAGAGTGTGGCTGCAAAGTGAAAAGCTTGAACGCATGCATCA

2,920 2,930 2,940 2,950 2,960 2,970 2,980 2,990 3,000 3,010 3,020

TCACCATCACCATTAACTCGAGGCATGCGGTACCAAGCTTGTCGAGAAGTACTAGAGGATCATAATCAGCCATACCACATTTGTAGAGGTTTTACTTGCTTTAAAAAA

U4947DH210-6.seq(1>2878) TCACCATCACCATTAACTCGAG
G11-pFastbac-R.ab1(1>813)

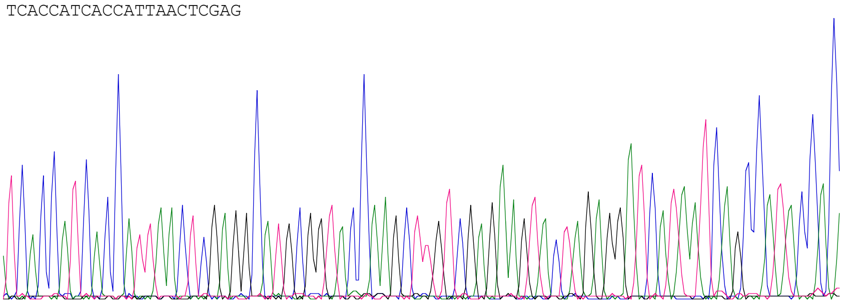

TCACCATCACCATTAACTCGAGGCATGCGGTACCAAGCTTGTCGAGAAGTACTAGAGGATCATAATCAGCCATACCACA

H02-SEQ3.ab1(1>825)

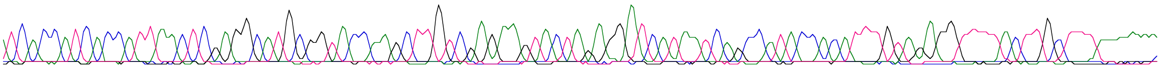

TCACCATCACCATTAACTCGAGGCATGCGGTACCAAGCTTGTCGAGAAGTACTAGAGGATCATAATCAGCCATACCACATTTGTAGAGGTTTTACTTGCTTTAAAAAA

3,030 3,040

CCTCCCACACCTCCCCC

H02-SEQ3.ab1(1>825)

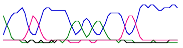

CCTCCCACACCTCCCCC

10 20 30 40 50 60 70 80 90 100

TTTTGTAATAAAAAAAACCTATAAATATTCCGGATTATTCATACCGTCCCACCATCGGGCGCGGATCCAACTCCTAAAAAAACCGCCACCATGGTGTTTCTGCCCCTCAA

U4947DH210-8.seq(1>2878) GGATCCAACTCCTAAAAAAACCGCCACCATGGTGTTTCTGCCCCTCAA
H07-pFastbac-F.ab1(1>825)

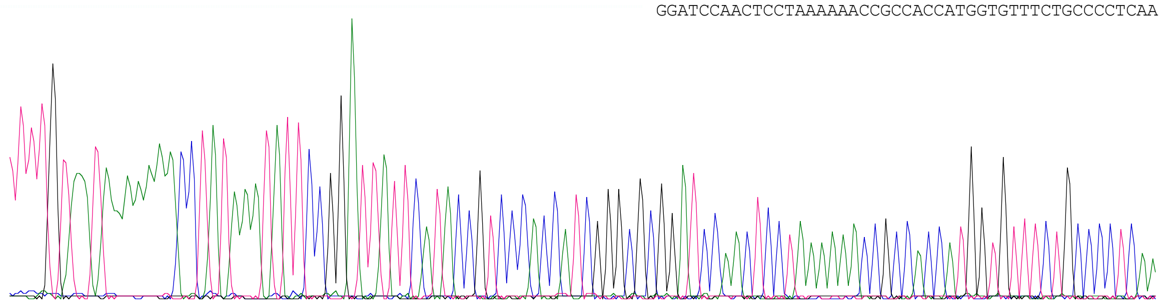

TTTTGTaATAAAAAAAACCTATAAATATTCCGGATTATTCATACCGTCCCACCATCGGGCGCGGATCCAACTCCTAAAAAAACCGCCACCATGGTGTTTCTGCCCCTCAA

G05-SEQ3R.ab1(1>808)

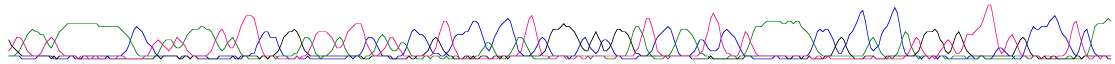

GTAATAAAAAAAACCTATAAATATTCCGGATTATTCATACCGTCCCACCATCGGGCGCGGATCCAACTCCTAAAAAAACCGCCACCATGGTGTTTCTGCCCCTCAA

110 120 130 140 150 160 170 180 190 200 210

ATGGTCCCTTGCAACCATGTCATTTCTACTTTCCTCACTGTTGGCTCTCTTAACTGTGTCCACTCCTTCATGGTGTCAGAGCACTGAAGCATCTCCAAAACGTAGTGA

U4947DH210-8.seq(1>2878) ATGGTCCCTTGCAACCATGTCATTTCTACTTTCCTCACTGTTGGCTCTCTTAACTGTGTCCACTCCTTCATGGTGTCAGAGCACTGAAGCATCTCCAAAACGTAGTGA
H07-pFastbac-F.ab1(1>825)

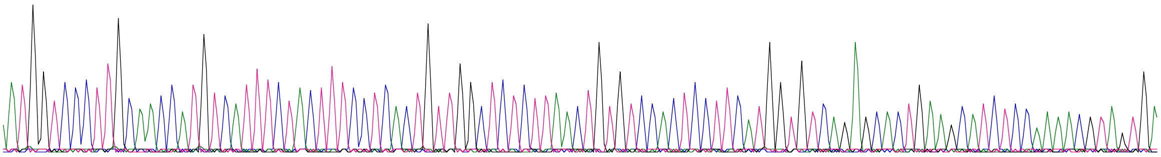

ATGGTCCCTTGCAACCATGTCATTTCTACTTTCCTCACTGTTGGCTCTCTTAACTGTGTCCACTCCTTCATGGTGTCAGAGCACTGAAGCATCTCCAAAACGTAGTGA

G05-SEQ3R.ab1(1>808)

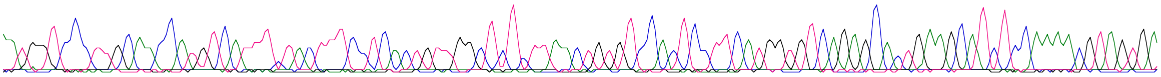

ATGGTCCCTTGCAACCATGTCATTTCTACTTTCCTCACTGTTGGCTCTCTTAACTGTGTCCACTCCTTCATGGTGTCAGAGCACTGAAGCATCTCCAAAACGTAGTGA

220 230 240 250 260 270 280 290 300 310 320

TGGGACACCATTTCCTTGGAATAAAATACGACTTCCTGAGTACGTCATCCCAGTTCATTATGATCTCTTGATCCATGCAAACCTTACCACGCTGACCTTCTGGGGAAC

U4947DH210-8.seq(1>2878) TGGGACACCATTTCCTTGGAATAAAATACGACTTCCTGAGTACGTCATCCCAGTTCATTATGATCTCTTGATCCATGCAAACCTTACCACGCTGACCTTCTGGGGAAC
H07-pFastbac-F.ab1(1>825)

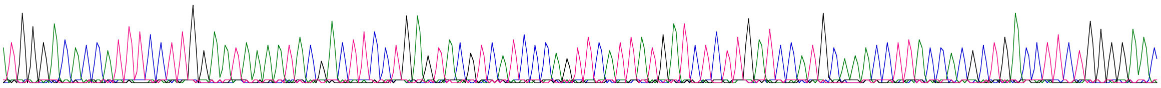

TGGGACACCATTTCCTTGGAATAAAATACGACTTCCTGAGTACGTCATCCCAGTTCATTATGATCTCTTGATCCATGCAAACCTTACCACGCTGACCTTCTGGGGAAC

220 230 240 250 260 270 280 290 300 310 320

TGGGACACCATTTCCTTGGAATAAAATACGACTTCCTGAGTACGTCATCCCAGTTCATTATGATCTCTTGATCCATGCAAACCTTACCACGCTGACCTTCTGGGGAAC

G05-SEQ3R.ab1(1>808)

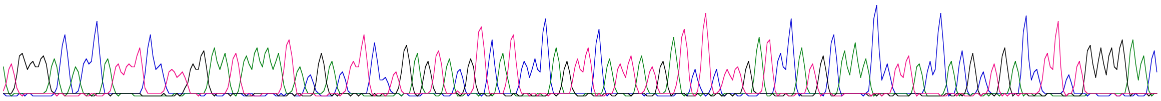

TGGGACACCATTTCCTTGGAATAAAATACGACTTCCTGAGTACGTCATCCCAGTTCATTATGATCTCTTGATCCATGCAAACCTTACCACGCTGACCTTCTGGGGAAC

330 340 350 360 370 380 390 400 410 420 430

CACGAAAGTAGAAATCACAGCCAGTCAGCCCACCAGCACCATCATCCTGCATAGTCACCACCTGCAGATATCTAGGGCCACCCTCAGGAAGGGAGCTGGAGAGAGGCT

U4947DH210-8.seq(1>2878)
H07-pFastbac-F.ab1(1>825)

CACGAAAGTAGAAATCACAGCCAGTCAGCCCACCAGCACCATCATCCTGCATAGTCACCACCTGCAGATATCTAGGGCCACCCTCAGGAAGGGAGCTGGAGAGAGGCT

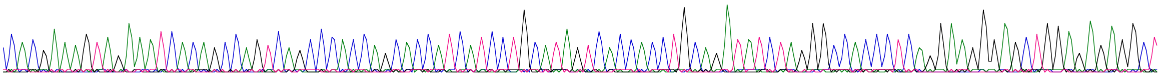

CACGAAAGTAGAAATCACAGCCAGTCAGCCCACCAGCACCATCATCCTGCATAGTCACCACCTGCAGATATCTAGGGCCACCCTCAGGAAGGGAGCTGGAGAGAGGCT

G05-SEQ3R.ab1(1>808)

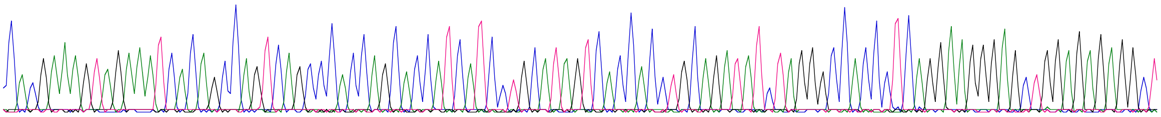

CACGAAAGTAGAAATCACAGCCAGTCAGCCCACCAGCACCATCATCCTGCATAGTCACCACCTGCAGATATCTAGGGCCACCCTCAGGAAGGGAGCTGGAGAGAGGCT

440 450 460 470 480 490 500 510 520 530 540

ATCGGAAGAACCCCTGCAGGTCCTGGAACACCCCCCGCAGGAGCAAATTGCACTGCTGGCTCCCGAGCCCCTCCTTGTCGGGCTCCCGTACACAGTTGTCATTCACTA

U4947DH210-8.seq(1>2878)
H07-pFastbac-F.ab1(1>825)

ATCGGAAGAACCCCTGCAGGTCCTGGAACACCCCCCGCAGGAGCAAATTGCACTGCTGGCTCCCGAGCCCCTCCTTGTCGGGCTCCCGTACACAGTTGTCATTCACTA

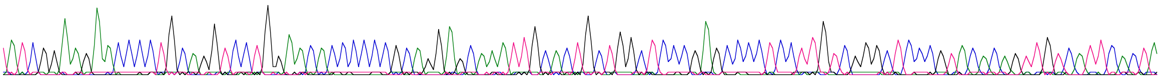

ATCGGAAGAACCCCTGCAGGTCCTGGAACACCCCCCGCAGGAGCAAATTGCACTGCTGGCTCCCGAGCCCCTCCTTGTCGGGCTCCCGTACACAGTTGTCATTCACTA

G05-SEQ3R.ab1(1>808)

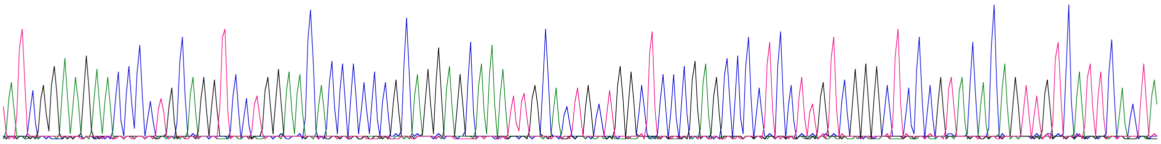

ATCGGAAGAACCCCTGCAGGTCCTGGAACACCCCCCGCAGGAGCAAATTGCACTGCTGGCTCCCGAGCCCCTCCTTGTCGGGCTCCCGTACACAGTTGTCATTCACTA

550 560 570 580 590 600 610 620 630 640

TGCTGGCAATCTTTCGGAGACTTTCCACGGATTTTACAAAAGCACCTACAGAACCAAGGAAGGGGAACTGAGGATACTAGCATCAACACAATTTGAACCCACTGCAGC

U4947DH210-8.seq(1>2878) → TGCTGGCAATCTTTCGGAGACTTTCCACGGATTTTACAAAAGCACCTACAGAACCAAGGAAGGGGAACTGAGGATACTAGCATCAACACAATTTGAACCCACTGCAGC

H07-pFastbac-F.ab1(1>825) →

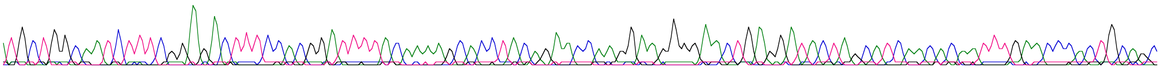

TGCTGGCAATCTTTCGGAGACTTTCCACGGATTTTACAAAAGCACCTACAGAACCAAGGAAGGGGAACTGAGGATACTAGCATCAACACAATTTGAACCCACTGCAGC

G05-SEQ3R.ab1(1>808) ←

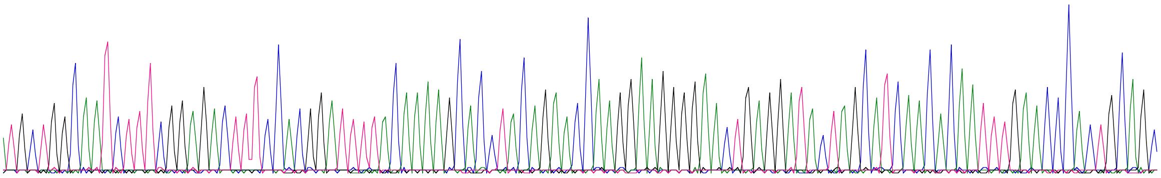

TGCTGGCAATCTTTCGGAGACTTTCCACGGATTTTACAAAAGCACCTACAGAACCAAGGAAGGGGAACTGAGGATACTAGCATCAACACAATTTGAACCCACTGCAGC

650 660 670 680 690 700 710 720 730 740 750

TAGAATGGCCTTTCCCTGCTTTGATGAACCTGCCTTCAAAGCAAGTTTCTCAATCAAAATTAGAAGAGAGCCAAGGCACCTAGCCATCTCCAATATGCCATTGGTGAA

U4947DH210-8.seq(1>2878) → TAGAATGGCCTTTCCCTGCTTTGATGAACCTGCCTTCAAAGCAAGTTTCTCAATCAAAATTAGAAGAGAGCCAAGGCACCTAGCCATCTCCAATATGCCATTGGTGAA

H07-pFastbac-F.ab1(1>825) →

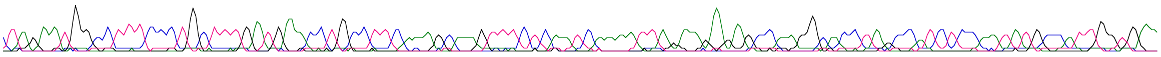

TAGAATGGCCTTTCCCTGCTTTGATGAACCTGCCTTCAAAGCAAGTTTCTCAATCAAAATTAGAAGAGAGCCAAGGCACCTAGCCATCTCCAATATGCCATTGGTGAA

G05-SEQ3R.ab1(1>808) ←

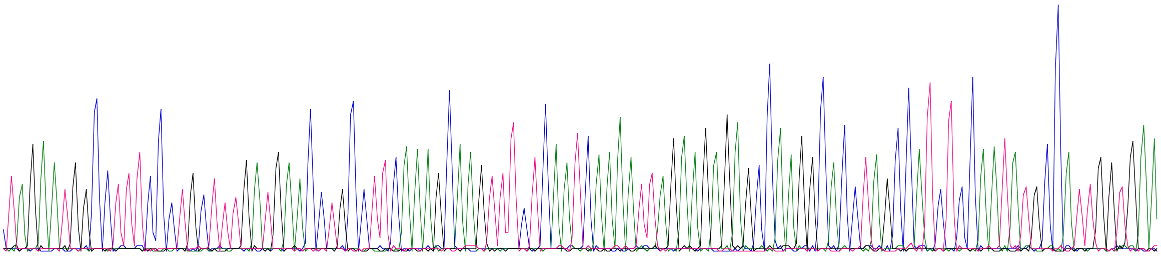

TAGAATGGCCTTTCCCTGCTTTGATGAACCTGCCTTCAAAGCAAGTTTCTCAATCAAAATTAGAAGAGAGCCAAGGCACCTAGCCATCTCCAATATGCCATTGGTGAA

G04-SEQ2R.ab1(1>826) ←

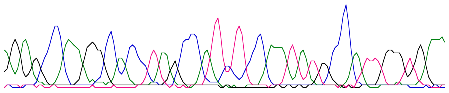

AGAGCCAAGGCACCTAGCCATCTCCAATATGCCATTGGTGAA

H09-SEQ1.ab1(1>826) →

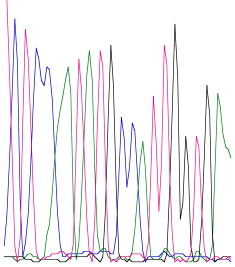

TCTCCaATATGCCATTGGTGAA

760 770 780 790 800 810 820 830 840 850 860

ATCTGTGACTGTTGCTGAAGGACTCATAGAAGACCATTTTGATGTCACTGTGAAGATGAGCACCTATCTGGTGGCCTTCATCATTTCAGATTTTGAGTCTGTCAGCAA

U4947DH210-8.seq(1>2878) ATCTGTGACTGTTGCTGAAGGACTCATAGAAGACCATTTTGATGTCACTGTGAAGATGAGCACCTATCTGGTGGCCTTCATCATTTCAGATTTTGAGTCTGTCAGCAA

H07-pFastbac-F.ab1(1>825)

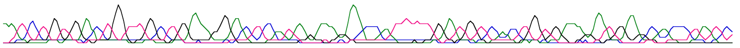

ATCTGTGACTGTTGCTGAAGGACTCATAGAAGACCATTTTGATGTCACTGTGAAGATGAGCACCTATCT

G05-SEQ3R.ab1(1>808)

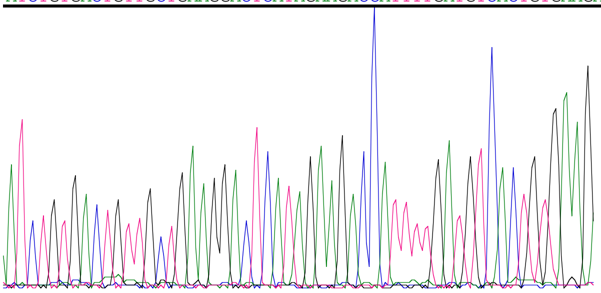

ATCTGTGACTGTTGCTGAAGGACTCATAGAAGACCATTTTGATGTCACTGTGAAGA

G04-SEQ2R.ab1(1>826)

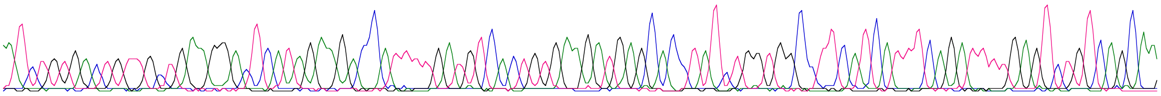

ATCTGTGACTGTTGCTGAAGGACTCATAGAAGACCATTTTGATGTCACTGTGAAGATGAGCACCTATCTGGTGGCCTTCATCATTTCAGATTTTGAGTCTGTCAGCAA

H09-SEQ1.ab1(1>826)

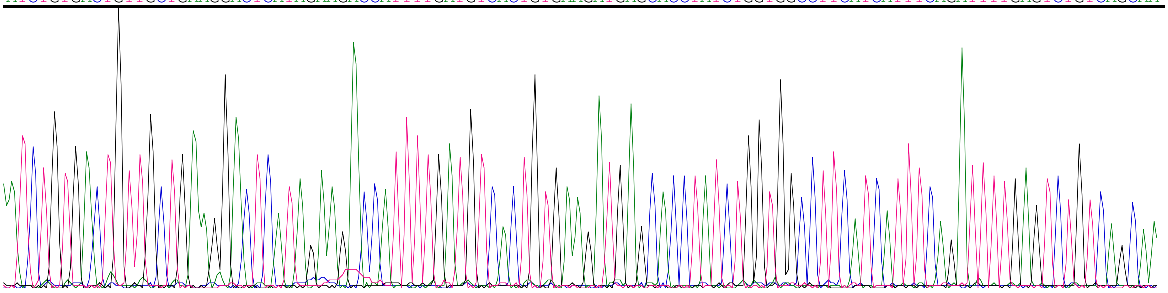

ATCTGTGACTGTTGCTGAAGGACTCATAGAAGACCATTTTGATGTCACTGTGAAGATGAGCACCTATCTGGTGGCCTTCATCATTTCAGATTTTGAGTCTGTCAGCAA

870 880 890 900 910 920 930 940 950 960 970

GATAACCAAGAGTGGAGTCAAGGTTTCTGTTTATGCTGTGCCAGACAAGATAAATCAAGCAGATTATGCACTGGATGCTGCGGTGACTCTTCTAGAATTTTATGAGGA

U4947DH210-8.seq(1>2878) GATAACCAAGAGTGGAGTCAAGGTTTCTGTTTATGCTGTGCCAGACAAGATAAATCAAGCAGATTATGCACTGGATGCTGCGGTGACTCTTCTAGAATTTTATGAGGA

G04-SEQ2R.ab1(1>826)

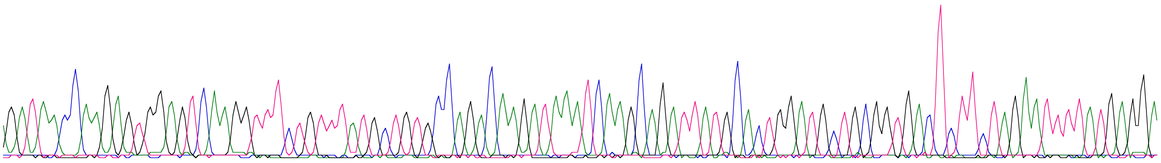

GATAACCAAGAGTGGAGTCAAGGTTTCTGTTTATGCTGTGCCAGACAAGATAAATCAAGCAGATTATGCACTGGATGCTGCGGTGACTCTTCTAGAATTTTATGAGGA

H09-SEQ1.ab1(1>826)

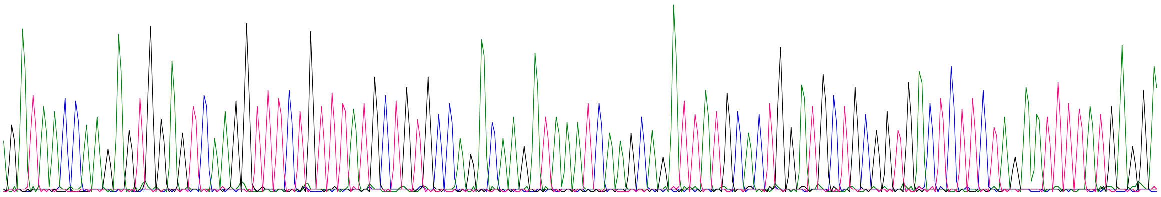

GATAACCAAGAGTGGAGTCAAGGTTTCTGTTTATGCTGTGCCAGACAAGATAAATCAAGCAGATTATGCACTGGATGCTGCGGTGACTCTTCTAGAATTTTATGAGGA

980 990 1,000 1,010 1,020 1,030 1,040 1,050 1,060 1,070 1,080

TTATTTCAGCATACCGTATCCCCTACCCAAACAAGATCTTGCTGCTATTCCCGACTTTCAGTCTGGTGCTATGGAAAACTGGGGACTGACAACATATAGAGAATCTGC

U4947DH210-8.seq(1>2878) TTATTTCAGCATACCGTATCCCCTACCCAAACAAGATCTTGCTGCTATTCCCGACTTTCAGTCTGGTGCTATGGAAAACTGGGGACTGACAACATATAGAGAATCTGC
G04-SEQ2R.ab1(1>826)

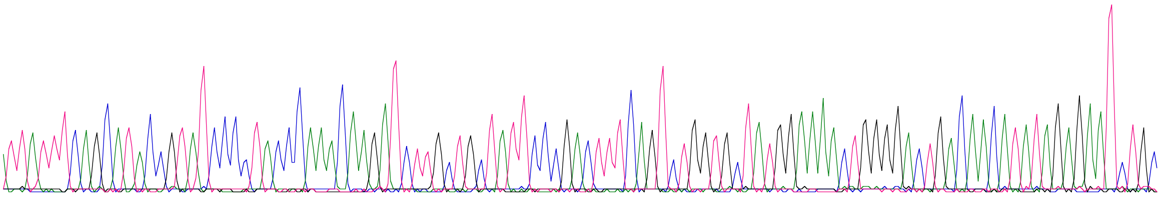

TTATTTCAGCATACCGTATCCCCTACCCAAACAAGATCTTGCTGCTATTCCCGACTTTCAGTCTGGTGCTATGGAAAACTGGGGACTGACAACATATAGAGAATCTGC

H09-SEQ1.ab1(1>826)

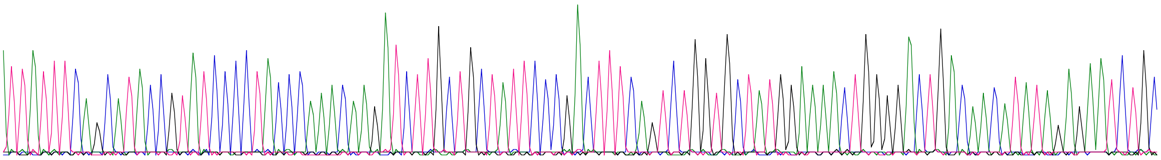

TTATTTCAGCATACCGTATCCCCTACCCAAACAAGATCTTGCTGCTATTCCCGACTTTCAGTCTGGTGCTATGGAAAACTGGGGACTGACAACATATAGAGAATCTGC

1,090 1,100 1,110 1,120 1,130 1,140 1,150 1,160 1,170 1,180

TCTGTTGTTTGATGCAGAAAAGTCTTCTGCATCAAGTAAGCTTGGCATCACAATGACTGTGGCCCATGAACTGGCTCACCAGTGGTTTGGGAACCTGGTCACTATGGA

U4947DH210-8.seq(1>2878) TCTGTTGTTTGATGCAGAAAAGTCTTCTGCATCAAGTAAGCTTGGCATCACAATGACTGTGGCCCATGAACTGGCTCACCAGTGGTTTGGGAACCTGGTCACTATGGA
G04-SEQ2R.ab1(1>826)

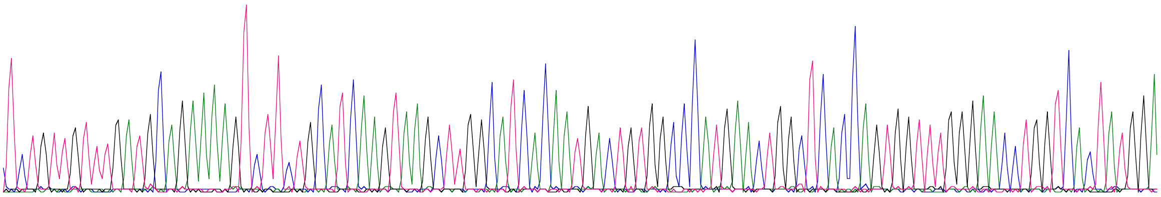

TCTGTTGTTTGATGCAGAAAAGTCTTCTGCATCAAGTAAGCTTGGCATCACAATGACTGTGGCCCATGAACTGGCTCACCAGTGGTTTGGGAACCTGGTCACTATGGA

H09-SEQ1.ab1(1>826)

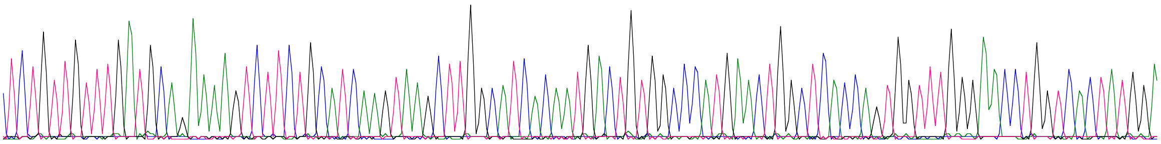

TCTGTTGTTTGATGCAGAAAAGTCTTCTGCATCAAGTAAGCTTGGCATCACAATGACTGTGGCCCATGAACTGGCTCACCAGTGGTTTGGGAACCTGGTCACTATGGA

1,190 1,200 1,210 1,220 1,230 1,240 1,250 1,260 1,270 1,280 1,290

ATGGTGGAATGATCTTTGGCTAAATGAAGGATTTGCCAAATTTATGGAGTTTGTGTCTGTCAGTGTGACCCATCCTGAACTGAAAGTTGGAGATTATTTCTTTGGCAA

U4947DH210-8.seq(1>2878) ATGGTGGAATGATCTTTGGCTAAATGAAGGATTTGCCAAATTTATGGAGTTTGTGTCTGTCAGTGTGACCCATCCTGAACTGAAAGTTGGAGATTATTTCTTTGGCAA
G04-SEQ2R.ab1(1>826)

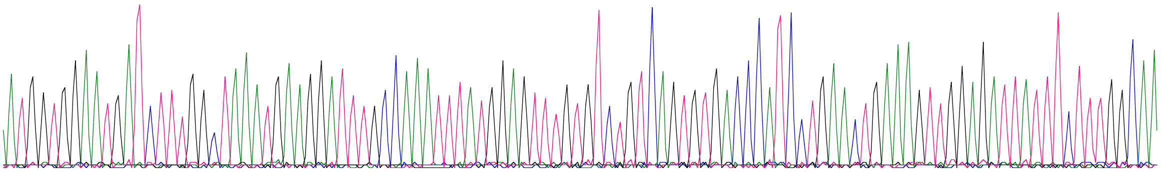

ATGGTGGAATGATCTTTGGCTAAATGAAGGATTTGCCAAATTTATGGAGTTTGTGTCTGTCAGTGTGACCCATCCTGAACTGAAAGTTGGAGATTATTTCTTTGGCAA

1,190 1,200 1,210 1,220 1,230 1,240 1,250 1,260 1,270 1,280 1,290

ATGGTGGAATGATCTTTGGCTAAATGAAGGATTTGCCAAATTTATGGAGTTTGTGTCTGTCAGTGTGACCCATCCTGAACTGAAAGTTGGAGATTATTTCTTTGGCAA

H09-SEQ1.ab1(1>826)

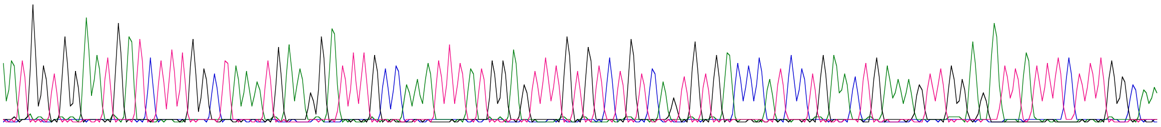

ATGGTGGAATGATCTTTGGCTAAATGAAGGATTTGCCAAATTTATGGAGTTTGTGTCTGTCAGTGTGACCCATCCTGAACTGAAAGTTGGAGATTATTTCTTTGGCAA

1,300 1,310 1,320 1,330 1,340 1,350 1,360 1,370 1,380 1,390 1,400

ATGTTTTGACGCAATGGAGGTAGATGCTTTAAATTCCTCACACCCTGTGTCTACACCTGTGGAAAATCCTGCTCAGATCCGGGAGATGTTTGATGATGTTTCTTATGA

U4947DH210-8.seq(1>2878) ATGTTTTGACGCAATGGAGGTAGATGCTTTAAATTCCTCACACCCTGTGTCTACACCTGTGGAAAATCCTGCTCAGATCCGGGAGATGTTTGATGATGTTTCTTATGA
G04-SEQ2R.ab1(1>826)

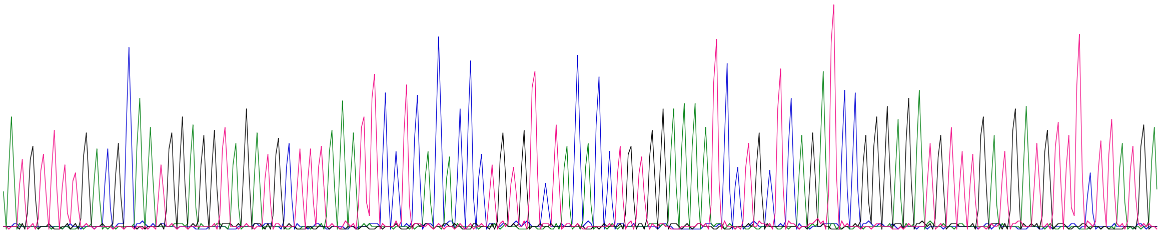

ATGTTTTGACGCAATGGAGGTAGATGCTTTAAATTCCTCACACCCTGTGTCTACACCTGTGGAAAATCCTGCTCAGATCCGGGAGATGTTTGATGATGTTTCTTATGA

H09-SEQ1.ab1(1>826)

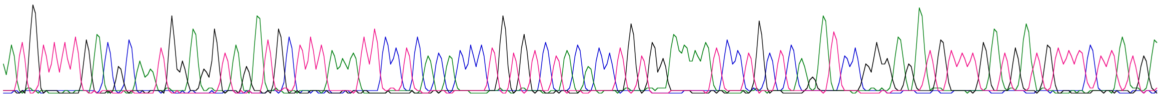

ATGTTTTGACGCAATGGAGGTAGATGCTTTAAATTCCTCACACCCTGTGTCTACACCTGTGGAAAATCCTGCTCAGATCCGGGAGATGTTTGATGATGTTTCTTATGA

1,410 1,420 1,430 1,440 1,450 1,460 1,470 1,480 1,490 1,500 1,510

TAAGGGAGCTTGTATTCTGAATATGCTAAGGGAGTATCTTAGTGCTGACGCATTTAAAAGTGGTATTGTACAGTATCTCCAGAAGCATAGCTATAAAAATACAAAAAA

U4947DH210-8.seq(1>2878) TAAGGGAGCTTGTATTCTGAATATGCTAAGGGAGTATCTTAGTGCTGACGCATTTAAAAGTGGTATTGTACAGTATCTCCAGAAGCATAGCTATAAAAATACAAAAAA
G04-SEQ2R.ab1(1>826)

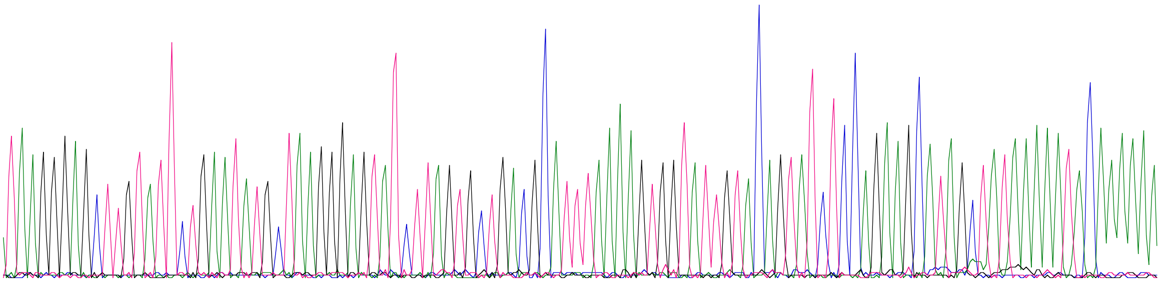

TAAGGGAGCTTGTATTCTGAATATGCTAAGGGAGTATCTTAGTGCTGACGCATTTAAAAGTGGTATTGTACAGTATCTCCAGAAGCATAGcTATAAAAATACAAAAAA

H09-SEQ1.ab1(1>826)

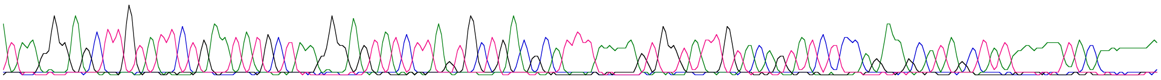

TAAGGGAGCTTGTATTCTGAATATGCTAAGGGAGTATCTTAGTGCTGACGCATTTAAAAGTGGTATTGTACAGTATCTCCAGAAGCATAGCTATAAAAATACAAAAAA

1,410 1,420 1,430 1,440 1,450 1,460 1,470 1,480 1,490 1,500 1,510

TAAGGGAGCTTGTATTCTGAATATGCTAAGGGAGTATCTTAGTGCTGACGCATTTAAAAGTGGTATTGTACAGTATCTCCAGAAGCATAGCTATAAAAATACAAAAAA

G03-SEQ1R.ab1(1>825)

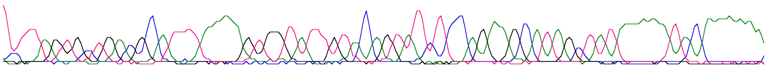

TCTTAGTGCTGACGCATTTAAAAGTGGTATTGTACAGTATCTCCAGAAGCATAGCTATAAAAATACAAAAAA

H10-SEQ2.ab1(1>823)

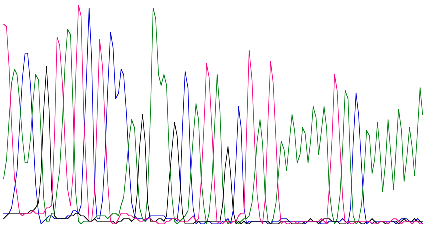

TaCAGTATCTCCAGAAGCATAGCTATAAAAATACAAAAAA

1,520 1,530 1,540 1,550 1,560 1,570 1,580 1,590 1,600 1,610 1,620

CGAGGACCTGTGGGATAGTATGGCAAGTATTTGCCCTACAGATGGTGTAAAAGGGATGGATGGCTTTTGCTCTAGAAGTCAACATTCATCTTCATCCTCACATTGGCA

U4947DH210-8.seq(1>2878) CGAGGACCTGTGGGATAGTATGGCAAGTATTTGCCCTACAGATGGTGTAAAAGGGATGGATGGCTTTTGCTCTAGAAGTCAACATTCATCTTCATCCTCACATTGGCA

G04-SEQ2R.ab1(1>826)

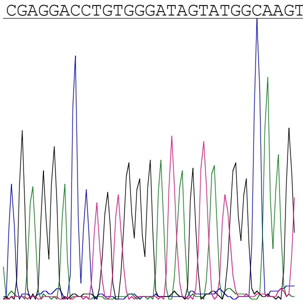

CGAGGACCTGTGGGATAGTATGGCAAGT

H09-SEQ1.ab1(1>826)

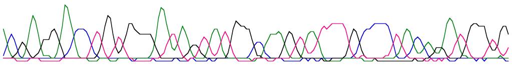

CGAGGACCTGTGGGATAGTATGGCAAGTATTTGCCCTACAGATGGTGT

G03-SEQ1R.ab1(1>825)

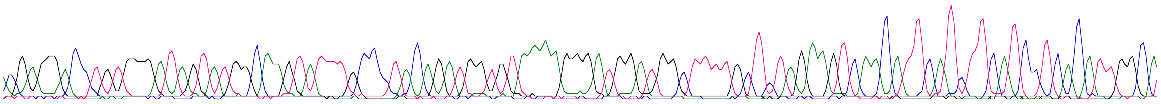

CGAGGACCTGTGGGATAGTATGGCAAGTATTTGCCCTACAGATGGTGTAAAAGGGATGGATGGCTTTTGCTCTAGAAGTCAACATTCATCTTCATCCTCACATTGGCA

H10-SEQ2.ab1(1>823)

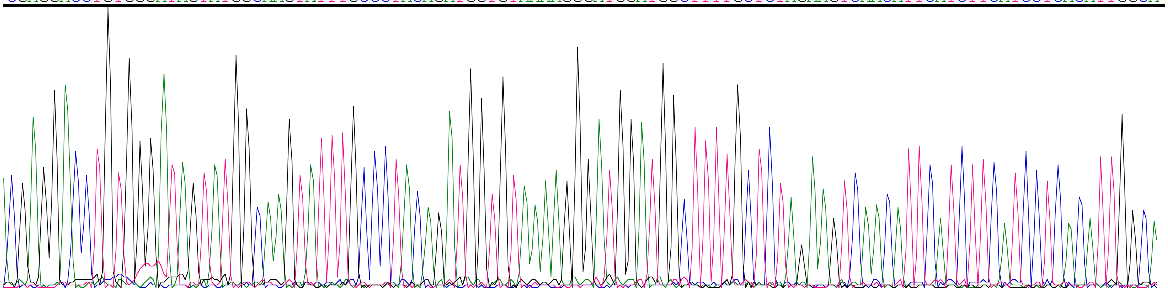

CGAGGACCTGTGGGaTAGTATGGCAAGTATTTGCCCTACAGATGGTGTAAAAGGGATGGATGGcTTTTGCTCTAGAAGTCAACATTCATCTTCATCCTCACATTGGCA

K528R

1,630 1,640 1,650 1,660 1,670 1,680 1,690 1,700 1,710 1,720

TCAGGAAGGGGTGGATGTGAAAACCATGATGAACACTTGGACACTGCAGCGTGGTTTTCCCCTAATAACCATCACAGTGAGGGGGAGGAATGTACACATGAAGCAAGA

U4947DH210-8.seq(1>2878) TCAGGAAGGGGTGGATGTGAAAACCATGATGAACACTTGGACACTGCAGCGTGGTTTTCCCCTAATAACCATCACAGTGAGGGGGAGGAATGTACACATGAAGCAAGA

G03-SEQ1R.ab1(1>825)

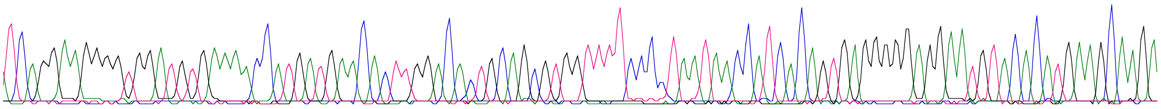

TCAGGAAGGGGTGGATGTGAAAACCATGATGAACACTTGGACACTGCAGCGTGGTTTTCCCCTAATAACCATCACAGTGAGGGGGAGGAATGTACACATGAAGCAAGA

H10-SEQ2.ab1(1>823)

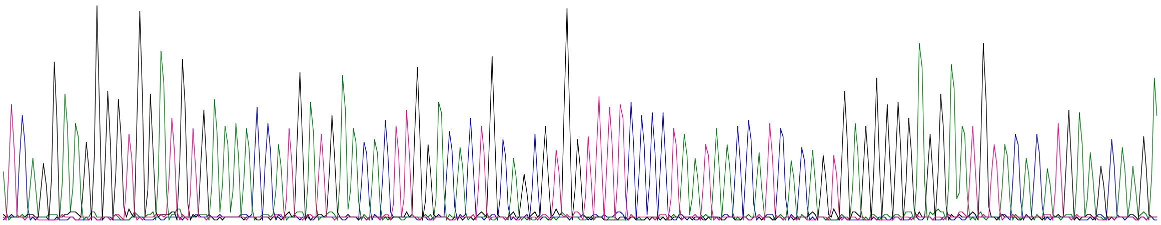

TCAGGAAGGGGTGGATGTGAAAACCATGATGAACACTTGGACACTGCAGCGTGGTTTTCCCCTAATAACCATCACAGTGAGGGGGAGGAATGTACACATGAAGCAAGA

1,730 1,740 1,750 1,760 1,770 1,780 1,790 1,800 1,810 1,820 1,830

GCACTACATGAAGGGCTCTGACGGCGCCCCGGACACTGGGTACCTGTGGCATGTTCCATTGACATTCATCACCAGCAAATCCGACATGGTCCATCGATTTTTGCTAAA

U4947DH210-8.seq(1>2878) GCACTACATGAAGGGCTCTGACGGCGCCCCGGACACTGGGTACCTGTGGCATGTTCCATTGACATTCATCACCAGCAAATCCGACATGGTCCATCGATTTTTGCTAAA

G03-SEQ1R.ab1(1>825)

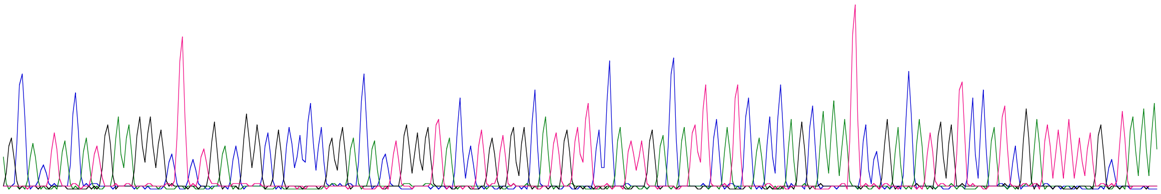

GCACTACATGAAGGGCTCTGACGGCGCCCCGGACACTGGGTACCTGTGGCATGTTCCATTGACATTCATCACCAGCAAATCCGACATGGTCCATCGATTTTTGCTAAA

H10-SEQ2.ab1(1>823)

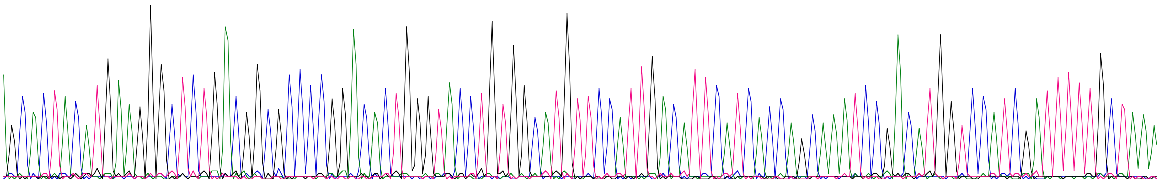

GCACTACATGAAGGGCTCTGACGGCGCCCCGGACACTGGGTACCTGTGGCATGTTCCATTGACATTCATCACCAGCAAATCCGACATGGTCCATCGATTTTTGCTAAA

1,840 1,850 1,860 1,870 1,880 1,890 1,900 1,910 1,920 1,930 1,940

AACAAAAACAGATGTGCTCATCCTCCCAGAAGAGGTGGAATGGATCAAATTTAATGTGGGCATGAATGGCTATTACATTGTGCATTACGAGGATGATGGATGGGACTC

U4947DH210-8.seq(1>2878) AACAAAAACAGATGTGCTCATCCTCCCAGAAGAGGTGGAATGGATCAAATTTAATGTGGGCATGAATGGCTATTACATTGTGCATTACGAGGATGATGGATGGGACTC

G03-SEQ1R.ab1(1>825)

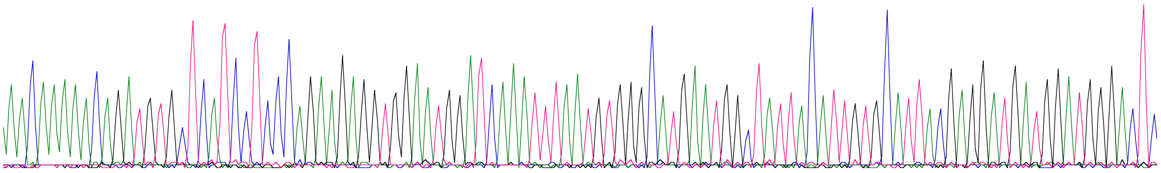

AACAAAAACAGATGTGCTCATCCTCCCAGAAGAGGTGGAATGGATCAAATTTAATGTGGGCATGAATGGCTATTACATTGTGCATTACGAGGATGATGGATGGGACTC

1,840 1,850 1,860 1,870 1,880 1,890 1,900 1,910 1,920 1,930 1,940

AACAAAAACAGATGTGCTCATCCTCCCAGAAGAGGTGGAATGGATCAAATTTAATGTGGGCATGAATGGCTATTACATTGTGCATTACGAGGATGATGGATGGGACTC

H10-SEQ2.ab1(1>823)

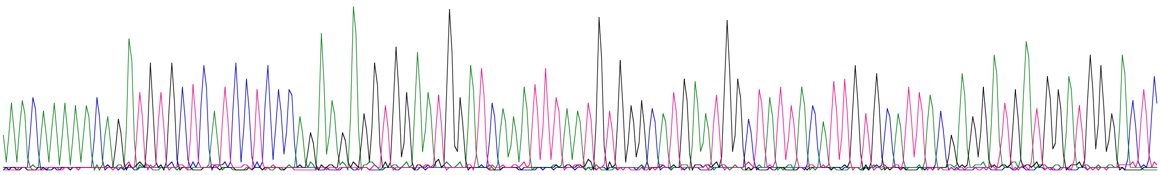

AACAAAAACAGATGTGCTCATCCTCCCAGAAGAGGTGGAATGGATCAAATTTAATGTGGGCATGAATGGCTATTACATTGTGCATTACGAGGATGATGGATGGGACTC

1,950 1,960 1,970 1,980 1,990 2,000 2,010 2,020 2,030 2,040 2,050

TTTGACTGGCCTTTTAAAAGGAACACACACAGCAGTCAGCAGTAATGATCGGGCGAGTCTCATTAACAATGCATTTCAGCTCGTCAGCATTGGGAAGCTGTCCATTGA

U4947DH210-8.seq(1>2878) TTTGACTGGCCTTTTAAAAGGAACACACACAGCAGTCAGCAGTAATGATCGGGCGAGTCTCATTAACAATGCATTTCAGCTCGTCAGCATTGGGAAGCTGTCCATTGA

G03-SEQ1R.ab1(1>825)

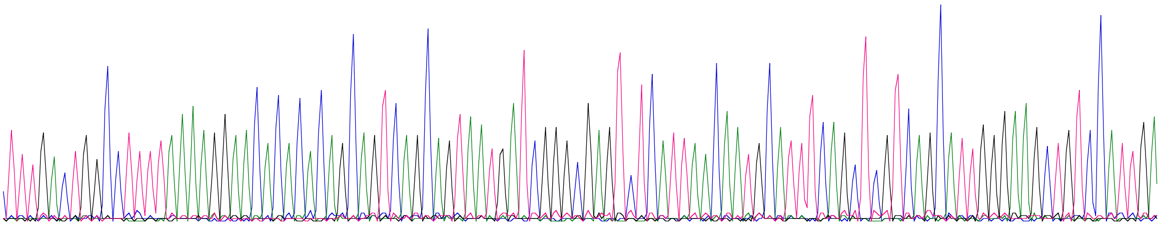

TTTGACTGGCCTTTTAAAAGGAACACACACAGCAGTCAGCAGTAATGATCGGGCGAGTcTCATTAACAATGCATTTCAGCTCGTCAGCATTGGGAAGCTGTCCATTGA

H10-SEQ2.ab1(1>823)

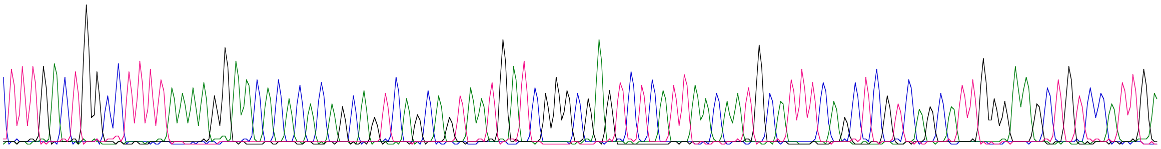

TTTGACTGGCCTTTTAAAAGGAACACACACAGCAGTCAGCAGTAATGATCGGGCGAGTCTCATTAACAATGCATTTCAGCTCGTCAGCATTGGGAAGCTGTCCATTGA

2,060 2,070 2,080 2,090 2,100 2,110 2,120 2,130 2,140 2,150 2,160

AAAGGCCTTGGATTTATCCCTGTACTTGAAACATGAAACTGAAATTATGCCCGTGTTTCAAGGTTTGAATGAGCTGATTCCTATGTATAAGTTAATGGAGAAAAGAGA

U4947DH210-8.seq(1>2878) AAAGGCCTTGGATTTATCCCTGTACTTGAAACATGAAACTGAAATTATGCCCGTGTTTCAAGGTTTGAATGAGCTGATTCCTATGTATAAGTTAATGGAGAAAAGAGA

G03-SEQ1R.ab1(1>825)

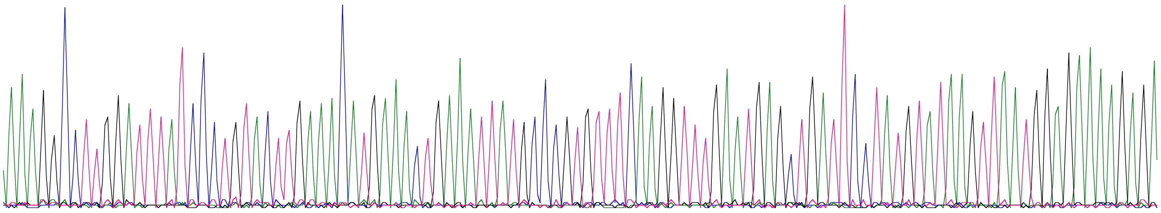

AAAGGCCTTGGATTTATCCCTGTACTTGAAACATGAAACTGAAATTATGCCCGTGTTTCAAGGTTTGAATGAGCTGATTCCTATGTATAAGTTAATGGAGAAAAGAGA

H10-SEQ2.ab1(1>823)

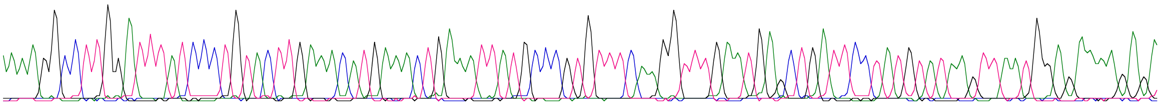

AAAGGCCTTGGATTTATCCCTGTACTTGAAACATGAAACTGAAATTATGCCCGTGTTTCAAGGTTTGAATGAGCTGATTCCTATGTATAAGTTAATGGAGAAAAGAGA

2,170 2,180 2,190 2,200 2,210 2,220 2,230 2,240 2,250 2,260

TATGAATGAAGTGGAAACTCAATTCAAGGCCTTCCTCATCAGGCTGCTAAGGGACCTCATTGATAAGCAGACATGGACAGACGAGGGCTCAGTCTCAGAGCGAATGCT

U4947DH210-8.seq(1>2878) TATGAATGAAGTGGAAACTCAATTCAAGGCCTTCCTCATCAGGCTGCTAAGGGACCTCATTGATAAGCAGACATGGACAGACGAGGGCTCAGTCTCAGAGCGAATGCT

G03-SEQ1R.ab1(1>825)

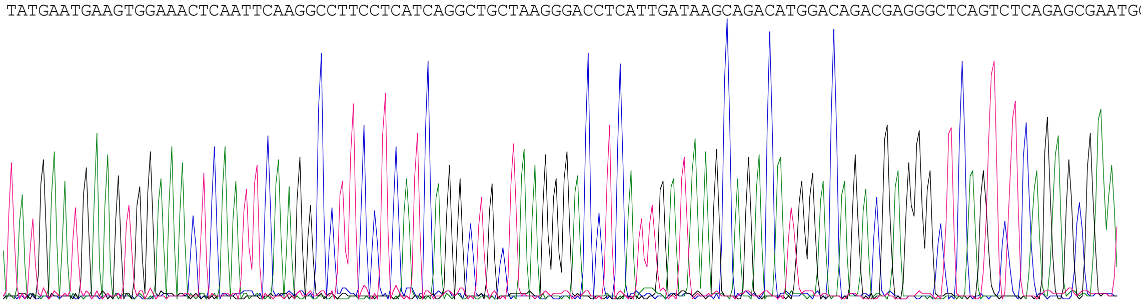

TATGAATGAAGTGGAAACTCAATTCAAGGCCTTCCTCATCAGGCTGCTAAGGGACCTCATTGATAAGCAGACATGGACAGACGAGGGCTCAGTCTCAGAGCGAAT

H10-SEQ2.ab1(1>823)

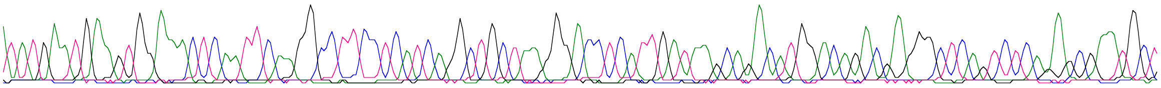

TATGAATGAAGTGGAAACTCAATTCAAGGCCTTCCTCATCAGGCTGCTAAGGGACCTCATTGATAAGCAGACATGGACAGACGAGGGCTCAGTCTCAGAGCGAATGCT

H08-pFastbac-R.ab1(1>815)

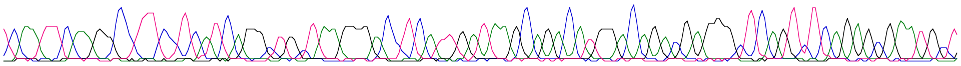

TCAATTCAAGGCCTTCCTCATCAGGCTGCTAAGGGACCTCATTGATAAGCAGACATGGACAGACGAGGGCTCAGTCTCAGAGCGAATGCT

H11-SEQ3.ab1(1>820)

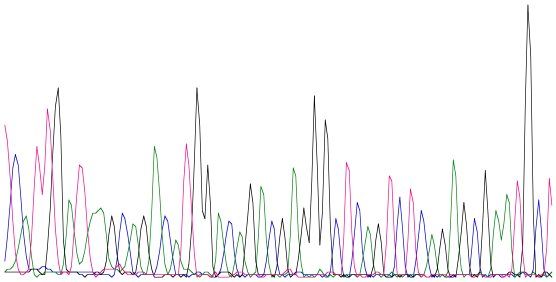

TCATTGATaAGCAGACATGGACAGACGAGGGCTCAGTCTCAGAGCGAATGCT

2,270 2,280 2,290 2,300 2,310 2,320 2,330 2,340 2,350 2,360 2,370

GCGGAGTGAGCTACTACTCCTCGCCTGTGTGCACAACTATCAGCCGTGCGTACAGAGGGCAGAAGGCTATTTCAGAAAGTGGAAGGAATCCAATGGAAACTTGAGCCT

U4947DH210-8.seq(1>2878) GCGGAGTGAGCTACTACTCCTCGCCTGTGTGCACAACTATCAGCCGTGCGTACAGAGGGCAGAAGGCTATTTCAGAAAGTGGAAGGAATCCAATGGAAACTTGAGCCT

H10-SEQ2.ab1(1>823)

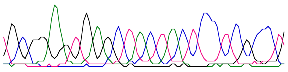

GCGGAGTGAGCTACTACTCCTCGCCTG

H08-pFastbac-R.ab1(1>815)

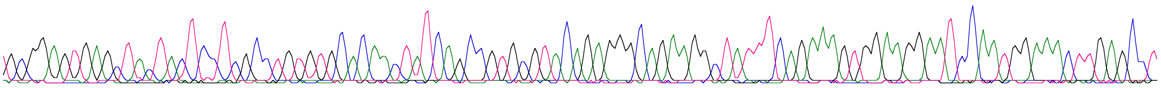

GCGGAGTGAGCTACTACTCCTCGCCTGTGTGCACAACTATCAGCCGTGCGTACAGAGGGCAGAAGGCTATTTCAGAAAGTGGAAGGAATCCAATGGAAACTTGAGCCT

Q730E

2,270 2,280 2,290 2,300 2,310 2,320 2,330 2,340 2,350 2,360 2,370

GCGGAGTGAGCTACTACTCCTCGCCTGTGTGCACAACTATCAGCCGTGCGTACAGAGGGCAGAAGGCTATTTCAGAAAGTGGAAGGAATCCAATGGAAACTTGAGCCT

H11-SEQ3.ab1(1>820)

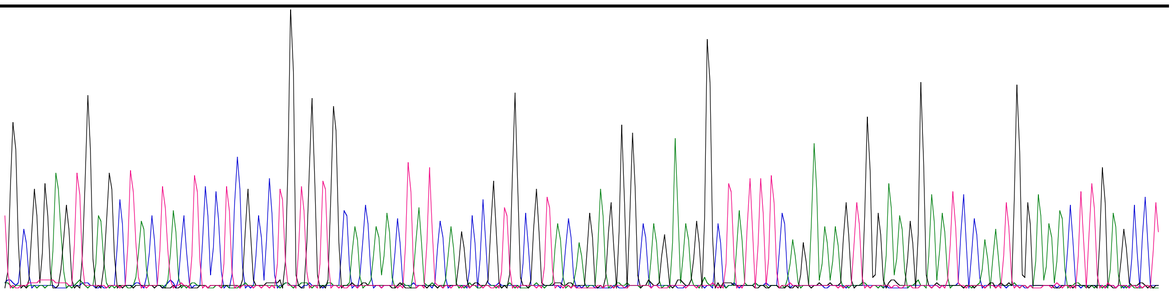

GCGGAGTGAGCTACTACTCCTCGCCTGTGTGCACAACTATCAGCCGTGCGTACAGAGGGCAGAAGGCTATTTCAGAAAGTGGAAGGAATCCAATGGAAACTTGAGCCT

2,380 2,390 2,400 2,410 2,420 2,430 2,440 2,450 2,460 2,470 2,480

GCCTGTCGACGTGACCTTGGCAGTGTTTGCTGTGGGGGCCCAGAGCACAGAAGGCTGGGATTTTCTTTATAGTAAATATCAGTTTTCTTTGTCCAGTACTGAGAAAAG

U4947DH210-8.seq(1>2878) GCCTGTCGACGTGACCTTGGCAGTGTTTGCTGTGGGGGCCCAGAGCACAGAAGGCTGGGATTTTCTTTATAGTAAATATCAGTTTTCTTTGTCCAGTACTGAGAAAAG

H08-pFastbac-R.ab1(1>815)

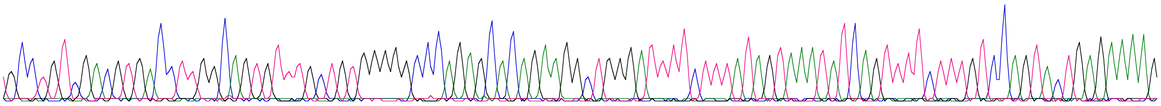

GCCTGTCGACGTGACCTTGGCAGTGTTTGCTGTGGGGGCCCAGAGCACAGAAGGCTGGGATTTTCTTTATAGTAAATATCAGTTTTCTTTGTCCAGTACTGAGAAAAG

H11-SEQ3.ab1(1>820)

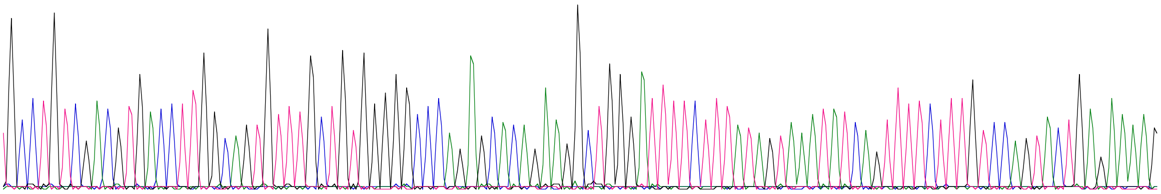

GCCTGTCGACGTGACCTTGGCAGTGTTTGCTGTGGGGGCCCAGAGCACAGAAGGCTGGGATTTTCTTTATAGTAAATATCAGTTTTCTTTGTCCAGTACTGAGAAAAG

2,490 2,500 2,510 2,520 2,530 2,540 2,550 2,560 2,570 2,580 2,590

CCAAATTGAATTTGCCCTCTGCAGAACCCAAAATAAGGAAAAGCTTCAATGGCTACTAGATGAAAGCTTTAAGGGAGATAAAATAAAAACTCAGGAGTTTCCACAAAT

U4947DH210-8.seq(1>2878) CCAAATTGAATTTGCCCTCTGCAGAACCCAAAATAAGGAAAAGCTTCAATGGCTACTAGATGAAAGCTTTAAGGGAGATAAAATAAAAACTCAGGAGTTTCCACAAAT

H08-pFastbac-R.ab1(1>815)

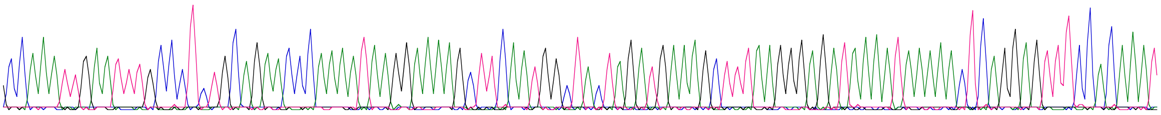

CCAAATTGAATTTGCCCTCTGCAGAACCCAAAATAAGGAAAAGCTTCAATGGCTACTAGATGAAAGCTTTAAGGGAGATAAAATAAAAACTCAGGAGTTTCCACAAAT

H11-SEQ3.ab1(1>820)

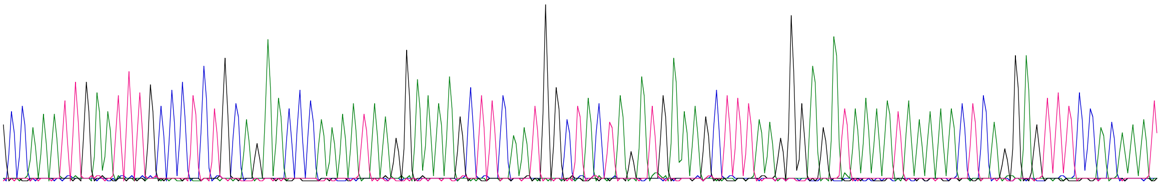

CCAAATTGAATTTGCCCTCTGCAGAACCCAAAATAAGGAAAAGCTTCAATGGCTACTAGATGAAAGCTTTAAGGGAGATAAAATAAAAACTCAGGAGTTTCCACAAAT

2,600 2,610 2,620 2,630 2,640 2,650 2,660 2,670 2,680 2,690 2,700

TCTTACACTCATTGGCAGGAACCCAGTAGGATACCCACTGGCCTGGCAATTTCTGAGGAAAAACTGGAACAAACTTGTACAAAAGTTTGAACTTGGCTCATCTTCCAT

U4947DH210-8.seq(1>2878) TCTTACACTCATTGGCAGGAACCCAGTAGGATACCCACTGGCCTGGCAATTTCTGAGGAAAAACTGGAACAAACTTGTACAAAAGTTTGAACTTGGCTCATCTTCCAT
H08-pFastbac-R.ab1(1>815)

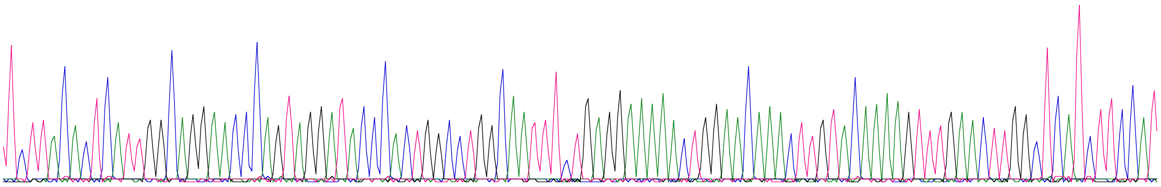

TCTTACACTCATTGGCAGGAACCCAGTAGGATACCCACTGGCCTGGCAATTTCTGAGGAAAAACTGGAACAAACTTGTACAAAAGTTTGAACTTGGCTCATCTTCCAT

H11-SEQ3.ab1(1>820)

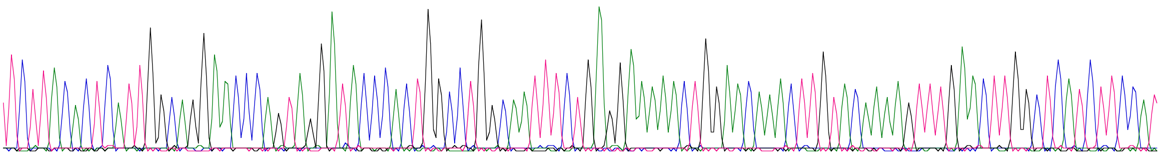

TCTTACACTCATTGGCAGGAACCCAGTAGGATACCCACTGGCCTGGCAATTTCTGAGGAAAAACTGGAACAAACTTGTACAAAAGTTTGAACTTGGCTCATCTTCCAT

2,710 2,720 2,730 2,740 2,750 2,760 2,770 2,780 2,790 2,800

AGCCCACATGGTAATGGGTACAACAAATCAATTCTCCACAAGAACACGGCTTGAAGAGGTAAAAGGATTCTTCAGCTCTTTGAAAGAAAATGGTTCTCAGCTCCGTTG

U4947DH210-8.seq(1>2878) AGCCCACATGGTAATGGGTACAACAAATCAATTCTCCACAAGAACACGGCTTGAAGAGGTAAAAGGATTCTTCAGCTCTTTGAAAGAAAATGGTTCTCAGCTCCGTTG
H08-pFastbac-R.ab1(1>815)

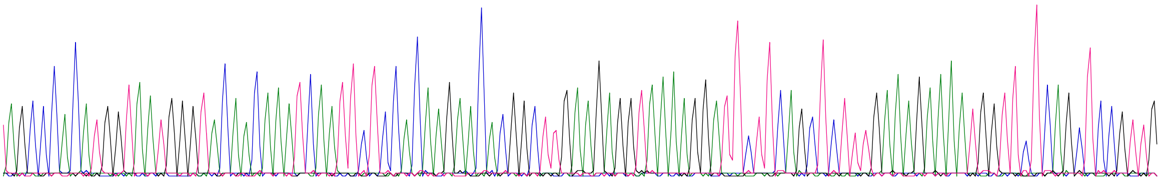

AGCCCACATGGTAATGGGTACAACAAATCAATTCTCCACAAGAACACGGCTTGAAGAGGTAAAAGGATTCTTCAGCTCTTTGAAAGAAAATGGTTCTCAGCTCCGTTG

H11-SEQ3.ab1(1>820)

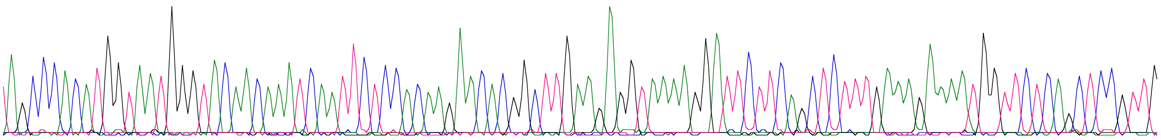

AGCCCACATGGTAATGGGTACAACAAATCAATTCTCCACAAGAACACGGCTTGAAGAGGTAAAAGGATTCTTCAGCTCTTTGAAAGAAAATGGTTCTCAGCTCCGTTG

2,810 2,820 2,830 2,840 2,850 2,860 2,870 2,880 2,890 2,900 2,910

TGTCCAACAGACAATTGAAACCATTGAAGAAAACATCGGTTGGATGGATAAGAATTTTGATAAAATCAGAGTGTGGCTGCAAAGTGAAAAGCTTGAACGCATGCATCA

U4947DH210-8.seq(1>2878) TGTCCAACAGACAATTGAAACCATTGAAGAAAACATCGGTTGGATGGATAAGAATTTTGATAAAATCAGAGTGTGGCTGCAAAGTGAAAAGCTTGAACGCATGCATCA
H08-pFastbac-R.ab1(1>815)

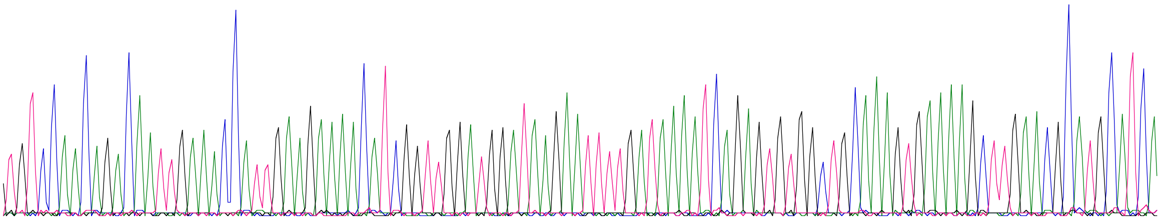

TGTCCAACAGACAATTGAAACCATTGAAGAAAACATCGGTTGGATGGATAAGAATTTTGATAAAATCAGAGTGTGGCTGCAAAGTGAAAAGCTTGAACGCATGCATCA

2,810 2,820 2,830 2,840 2,850 2,860 2,870 2,880 2,890 2,900 2,910

TGTCCAACAGACAATTGAAACCATTGAAGAAAACATCGGTTGGATGGATAAGAATTTTGATAAAATCAGAGTGTGGCTGCAAAGTGAAAAGCTTGAACGCATGCATCA

H11-SEQ3.ab1(1>820)

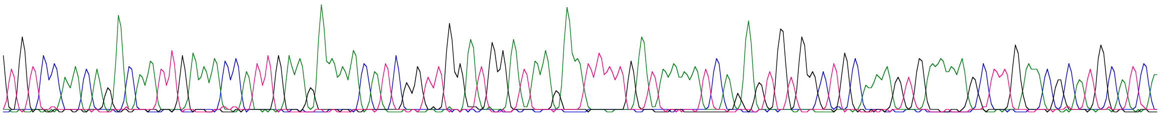

TGTCCAACAGACAATTGAAACCATTGAAGAAAACATCGGTTGGATGGATAAGAATTTTGATAAAATCAGAGTGTGGCTGCAAAGTGAAAAGCTTGAACGCATGCATCA

2,920 2,930 2,940 2,950 2,960 2,970 2,980 2,990 3,000 3,010 3,020

TCACCATCACCATTAACTCGAGGCATGCGGTACCAAGCTTGTCGAGAAGTACTAGAGGATCATAATCAGCCATACCACATTTGTAGAGGTTTTACTTGCTTTAAAAAA

U4947DH210-8.seq(1>2878) TCACCATCACCATTAACTCGAG

H08-pFastbac-R.ab1(1>815)

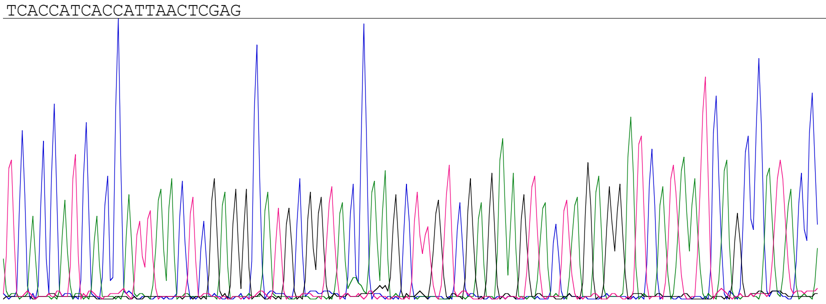

TCACCATCACCATTAACTCGAGGCATGCGGTACCAAGCTTGTCGAGAAGTACTAGAGGATCATAATCAGCCATACCA

H11-SEQ3.ab1(1>820)

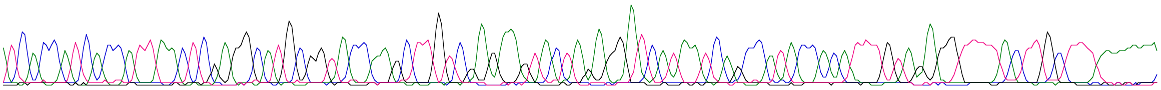

TCACCATCACCATTAACTCGAGGCATGCGGTACCAAGCTTGTCGAGAAGTACTAGAGGATCATAATCAGCCATACCACATTTGTAGAGGTTTTACTTGCTTTAAAAAA

3,030

CCTCCCACACCT

H11-SEQ3.ab1(1>820)

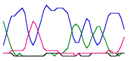

CCTCCCACACCT

Haplotype 7

10 20 30 40 50 60 70 80 90 100

ATAGATCATGGAGATAATTAAAATGATAACCATCTCGCAAATAAATAAGTATTTTACTGTTTTCGTAACAGTTTTGTAATAAAAAAACCTATAAATATTCCGGATTA

A10-SEQ1R.ab1(1>661)

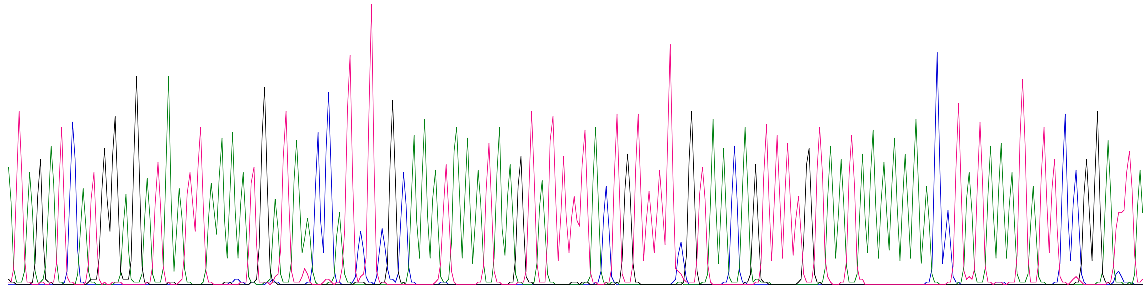

ATAGATCATGGAGATAATTAAAATGATAACCATCTCGCAAATAAATAAGTATTTTACTGTTTTCGTAACAGTTTTGTAATAAAAAAACCTATAAATATTCCGGATTA

D09-pFastbac-F.ab1(1>821)

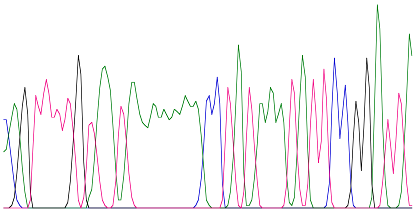

CAGTTTTGTAATAAAAAAACCTATAAATATTCCGGATTA

110 120 130 140 150 160 170 180 190 200 210

TTCATACCGTCCCACCATCGGGCGCGGATCCAACTCCTAAAAAACCGCCACCATGGTGTTTCTGCCCCTCAAATGGTCCCTTGCAACCATGTCATTTCTACTTTCCT

U3823DH140-6.seq(1>2878)
A10-SEQ1R.ab1(1>661)

GGATCCAACTCCTAAAAAACCGCCACCATGGTGTTTCTGCCCCTCAAATGGTCCCTTGCAACCATGTCATTTCTACTTTCCT

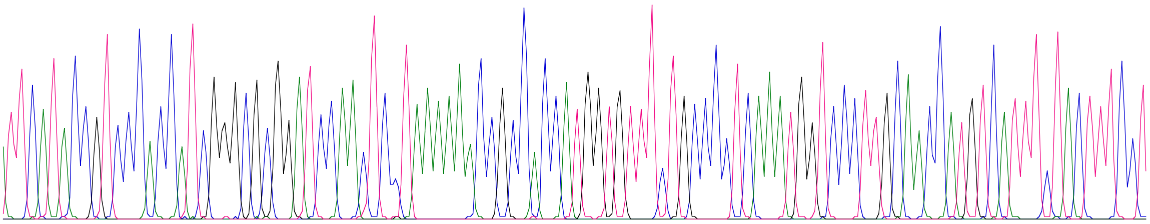

TTCATACCGTCCCACCATCGGGCGCGGATCCAACTCCTAAAAAACCGCCACCATGGTGTTTCTGCCCCTCAAATGGTCCCTTGCAACCATGTCATTTCTACTTTCCT

D09-pFastbac-F.ab1(1>821)

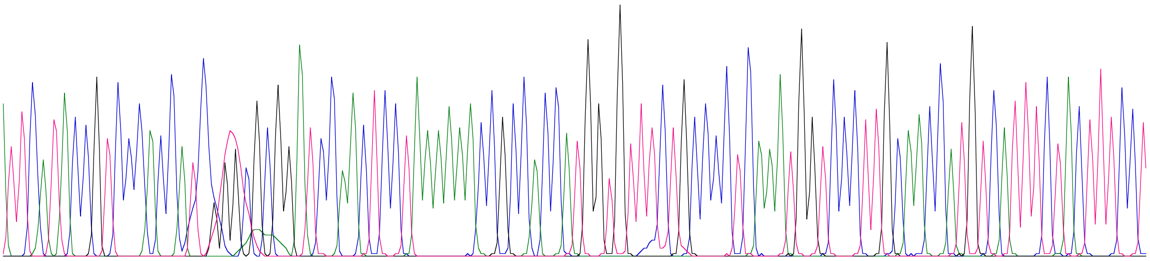

TTCATACCGTCCCACCATCGGGCGCGGATCCAACTCCTAAAAAACCGCCACCATGGTGTTTCTGCCCCTCAAATGGTCCCTTGCAACCATGTCATTTCTACTTTCCT

220 230 240 250 260 270 280 290 300 310 320

CACTGTTGGCTCTCTTAACTGTGTCCACTCCTTCATGGTGTCAGAGCACTGAAGCATCTCCAAAACGTAGTGATGGGACACCATTTCCTTGGAATAAAATACGACTT

U3823DH140-6.seq(1>2878)
A10-SEQ1R.ab1(1>661)

CACTGTTGGCTCTCTTAACTGTGTCCACTCCTTCATGGTGTCAGAGCACTGAAGCATCTCCAAAACGTAGTGATGGGACACCATTTCCTTGGAATAAAATACGACTT

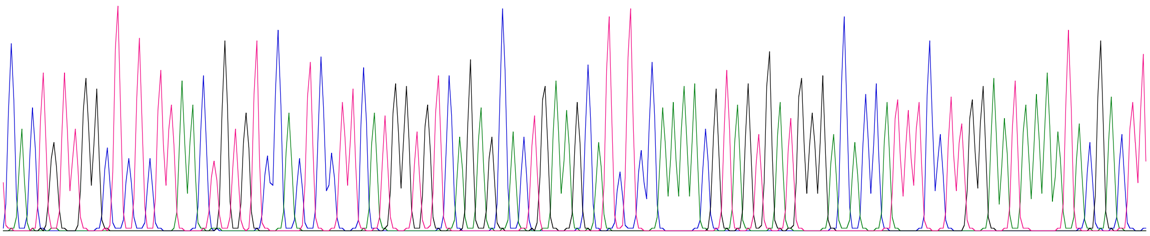

CACTGTTGGCTCTCTTAACTGTGTCCACTCCTTCATGGTGTCAGAGCACTGAAGCATCTCCAAAACGTAGTGATGGGACACCATTTCCTTGGAATAAAATACGACTT

220 230 240 250 260 270 280 290 300 310 320

CACTGTTGGCTCTCTTAACTGTGTCCACTCCTTCATGGTGTCAGAGCACTGAAGCATCTCCAAAACGTAGTGATGGGACACCATTTCCTTGGAATAAAATACGACTT

D09-pFastbac-F.ab1(1>821)

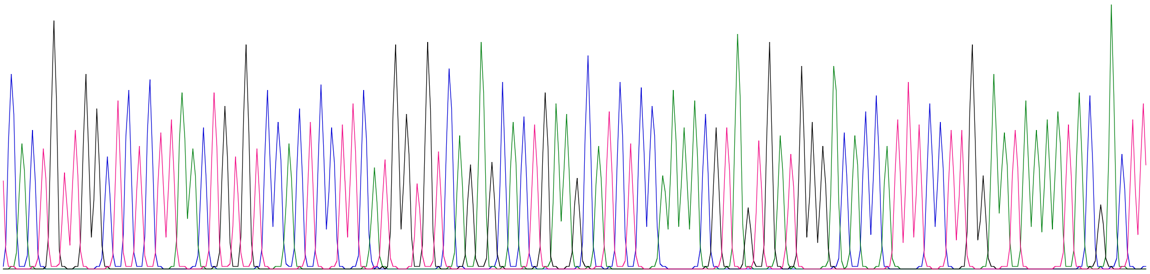

CACTGTTGGCTCTCTTAACTGTGTCCACTCCTTCATGGTGTCAGAGCACTGAAGCATCTCCAAAACGTAGTGATGGGACACCATTTCCTTGGAATAAAATACGACTT

E56K

330 340 350 360 370 380 390 400 410 420

CCTAAGTACGTCATCCCAGTTCATTATGATCTCTTGATCCATGCAAACCTTACCACGCTGACCTTCTGGGGAACCACGAAAGTAGAAATCACAGCCAGTCAGCCCAC

U3823DH140-6.seq(1>2878)
A10-SEQ1R.ab1(1>661)

CCTAAGTACGTCATCCCAGTTCATTATGATCTCTTGATCCATGCAAACCTTACCACGCTGACCTTCTGGGGAACCACGAAAGTAGAAATCACAGCCAGTCAGCCCAC

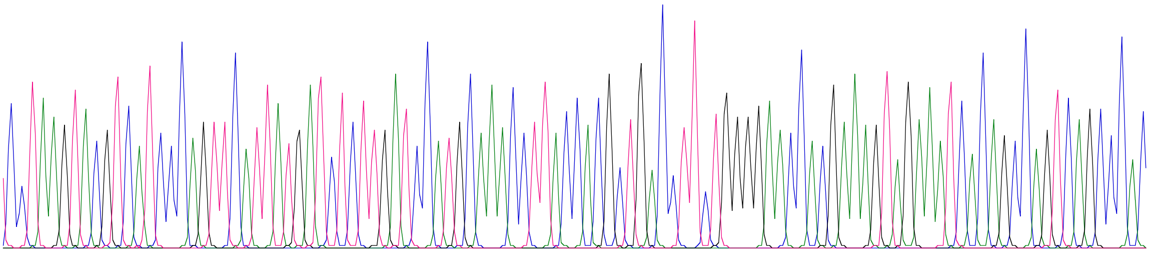

CCTAAGTACGTCATCCCAGTTCATTATGATCTCTTGATCCATGCAAACCTTACCACGCTGACCTTCTGGGGAACCACGAAAGTAGAAATCACAGCCAGTCAGCCCAC

D09-pFastbac-F.ab1(1>821)

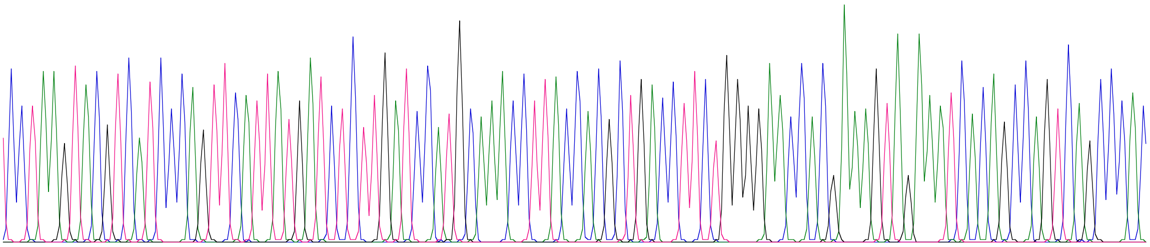

CCTAAGTACGTCATCCCAGTTCATTATGATCTCTTGATCCATGCAAACCTTACCACGCTGACCTTCTGGGGAACCACGAAAGTAGAAATCACAGCCAGTCAGCCCAC

430 440 450 460 470 480 490 500 510 520 530

CAGCACCATCATCCTGCATAGTCACCACCTGCAGATATCTAGGGCCACCCTCAGGAAGGGAGCTGGAGAGAGGCTATCGGAAGAACCCCTGCAGGTCCTGGAACACC

U3823DH140-6.seq(1>2878)
A10-SEQ1R.ab1(1>661)

CAGCACCATCATCCTGCATAGTCACCACCTGCAGATATCTAGGGCCACCCTCAGGAAGGGAGCTGGAGAGAGGCTATCGGAAGAACCCCTGCAGGTCCTGGAACACC

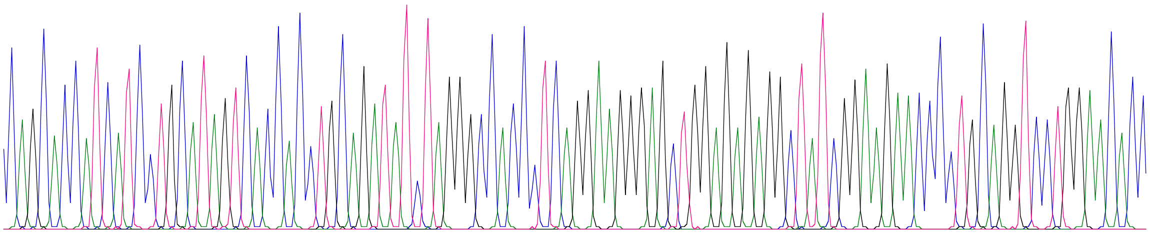

CAGCACCATCATCCTGCATAGTCACCACCTGCAGATATCTAGGGCCACCCTCAGGAAGGGAGCTGGAGAGAGGCTATCGGAAGAACCCCTGCAGGTCCTGGAACACC

D09-pFastbac-F.ab1(1>821)

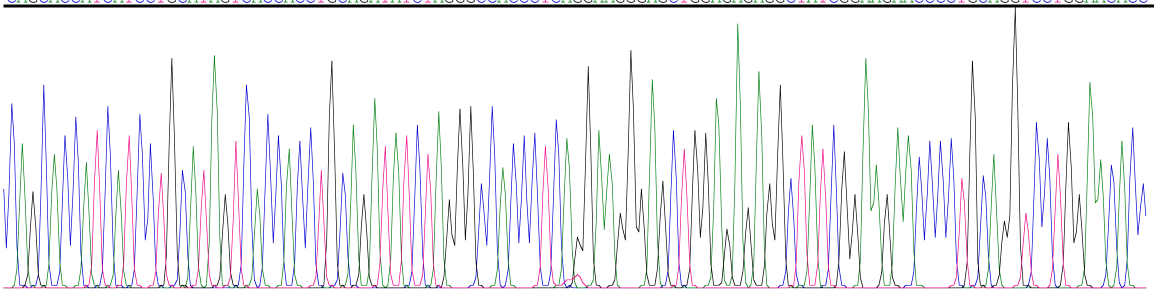

CAGCACCATCATCCTGCATAGTCACCACCTGCAGATATCTAGGGCCACCCTCAGGAAGGGAGCTGGAGAGAGGCTATCGGAAGAACCCCTGCAGGTCCTGGAACACC

430 440 450 460 470 480 490 500 510 520 530

CAGCACCATCATCCTGCATAGTCACCACCTGCAGATATCTAGGGCCACCCTCAGGAAGGGAGCTGGAGAGAGGCTATCGGAAGAACCCCTGCAGGTCCTGGAACACC

A11-SEQR.ab1(1>815)

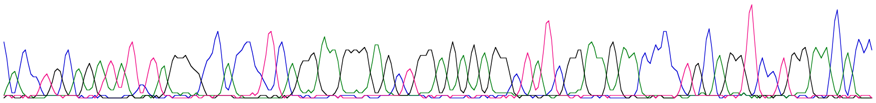

CACCTGCAGATATCTAGGGCCACCCTCAGGAAGGGAGCTGGAGAGAGGCTATCGGAAGAACCCCTGCAGGTCCTGGAACACC

D11-SEQ1.ab1(1>821)

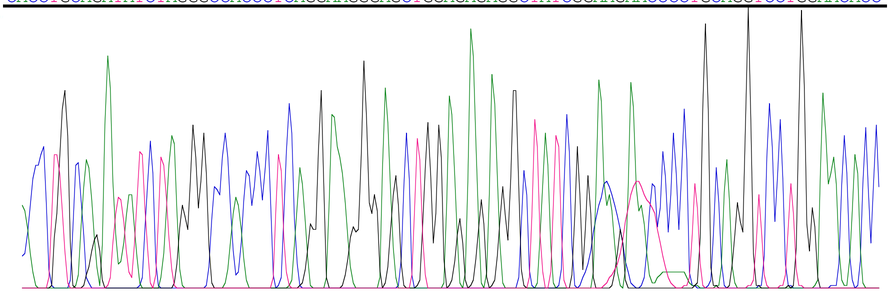

ACCTGCAGATATCTAGGGCCACCCTCAGGAAGGGAGCTGGAGAGAGGCTATCGGAAGAACCCCTGCAGGTCCTGGAACACC

540 550 560 570 580 590 600 610 620 630 640

CCCCGCAGGAGCAAATTGCACTGCTGGCTCCCGAGCCCCTCCTTGTCGGGCTCCCGTACACAGTTGTCATTCACTATGCTGGCAATCTTTCGGAGACTTTCCACGGA

U3823DH140-6.seq(1>2878)

CCCCGCAGGAGCAAATTGCACTGCTGGCTCCCGAGCCCCTCCTTGTCGGGCTCCCGTACACAGTTGTCATTCACTATGCTGGCAATCTTTCGGAGACTTTCCACGGA

A10-SEQ1R.ab1(1>661)

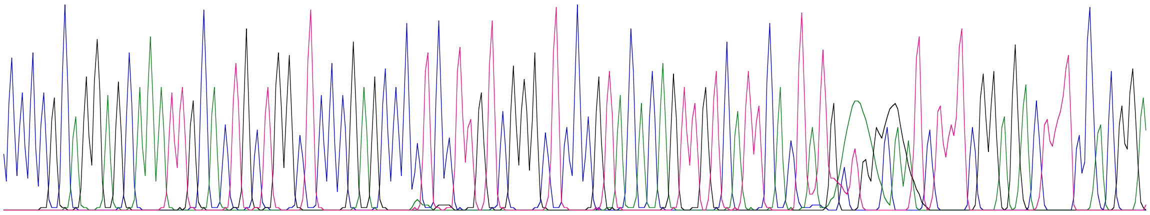

CCCCGCAGGAGCAAATTGCACTGCTGGCTCCCGAGCCCCTCCTTGTCGGGCTCCCGTACACAGTTGTCATTCACTATGCTGGCAATCTTTCGGAGACTTTCCACGGA

D09-pFastbac-F.ab1(1>821)

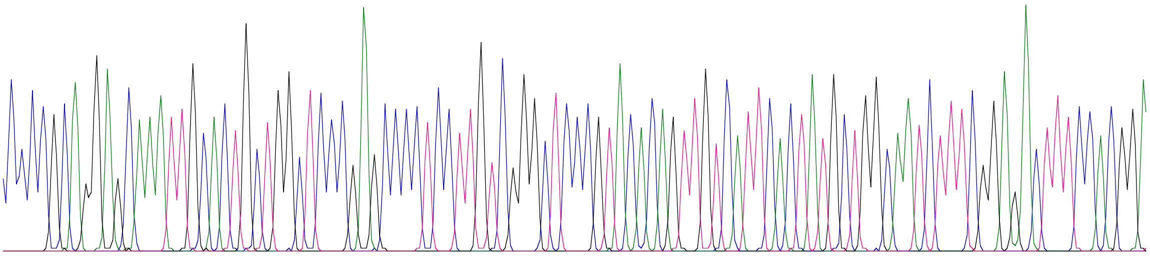

CCCCGCAGGAGCAAATTGCACTGCTGGCTCCCGAGCCCCTCCTTGTCGGGCTCCCGTACACAGTTGTCATTCACTATGCTGGCAATCTTTCGGAGACTTTCCACGGA

A11-SEQR.ab1(1>815)

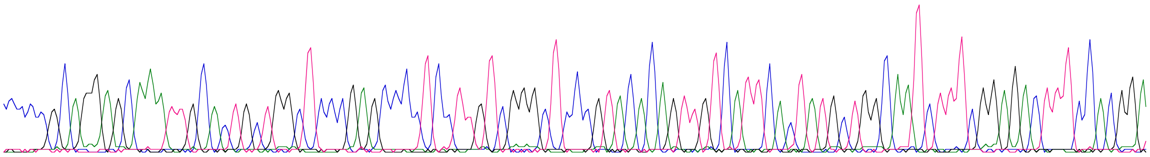

CCCCGCAGGAGCAAATTGCACTGCTGGCTCCCGAGCCCCTCCTTGTCGGGCTCCCGTACACAGTTGTCATTCACTATGCTGGCAATCTTTCGGAGACTTTCCACGGA

D11-SEQ1.ab1(1>821)

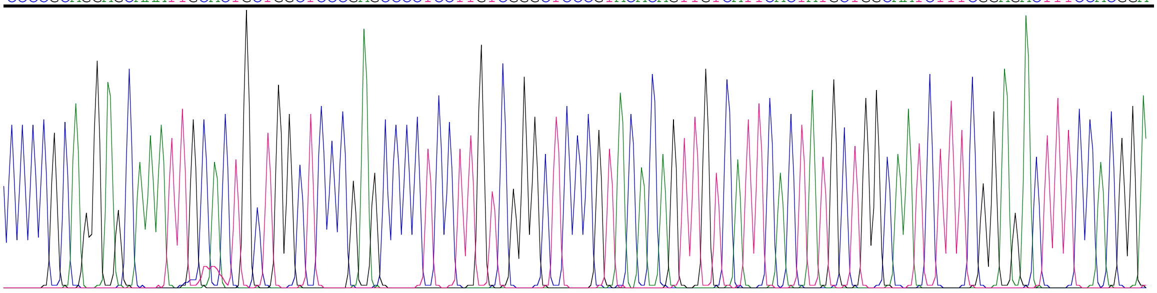

CCCCGCAGGAGCAAATTGCACTGCTGGCTCCCGAGCCCCTCCTTGTCGGGCTCCCGTACACAGTTGTCATTCACTATGCTGGCAATCTTTCGGAGACTTTCCACGGA

650 660 670 680 690 700 710 720 730 740

TTTTACAAAAGCACCTACAGAACCAAGGAAGGGGAACTGAGGATACTAGCATCAACACAATTTGAACCCACTGCAGCTAGAATGGCCTTTCCCTGCTTTGATGAACC

U3823DH140-6.seq(1>2878) → TTTTACAAAAGCACCTACAGAACCAAGGAAGGGGAACTGAGGATACTAGCATCAACACAATTTGAACCCACTGCAGCTAGAATGGCCTTTCCCTGCTTTGATGAACC

A10-SEQ1R.ab1(1>661) ←

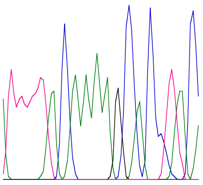

TTTTACAAAAGCACCTACA

D09-pFastbac-F.ab1(1>821) →

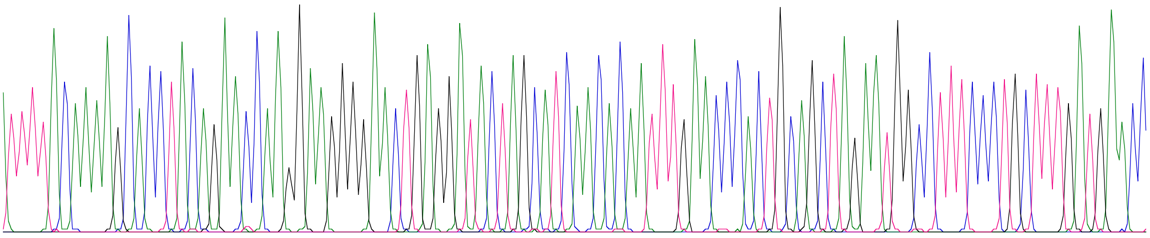

TTTTACAAAAGCACCTACAGAACCAAGGAAGGGGAACTGAGGATACTAGCATCAACACAATTTGAACCCACTGCAGCTAGAATGGCCTTTCCCTGCTTTGATGAACC

A11-SEQR.ab1(1>815) ←

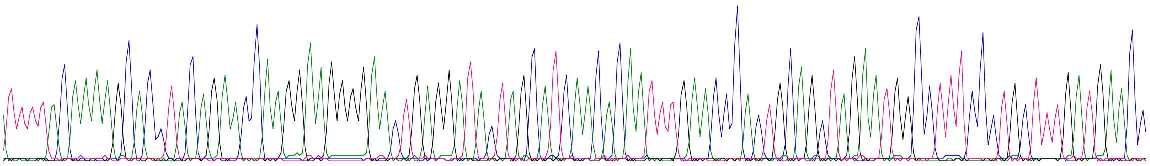

TTTTACAAAAGCACCTACAGAACCAAGGAAGGGGAACTGAGGATACTAGCATCAACACAATTTGAACCCACTGCAGCTAGAATGGCCTTTCCCTGCTTTGATGAACC

D11-SEQ1.ab1(1>821) →

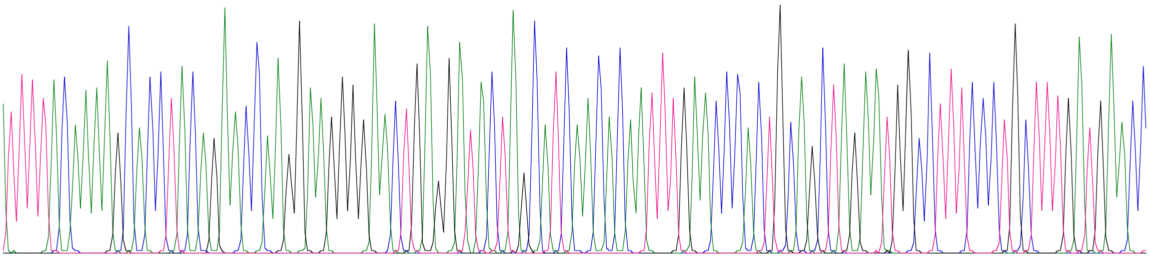

TTTTACAAAAGCACCTACAGAACCAAGGAAGGGGAACTGAGGATACTAGCATCAACACAATTTGAACCCACTGCAGCTAGAATGGCCTTTCCCTGCTTTGATGAACC

750 760 770 780 790 800 810 820 830 840 850

TGCCTTCAAAGCAAGTTTCTCAATCAAAATTAGAAGAGAGCCAAGGCACCTAGCCATCTCCAATATGCCATTGGTGAAATCTGTGACTGTTGCTGAAGGACTCATAG

U3823DH140-6.seq(1>2878) → TGCCTTCAAAGCAAGTTTCTCAATCAAAATTAGAAGAGAGCCAAGGCACCTAGCCATCTCCAATATGCCATTGGTGAAATCTGTGACTGTTGCTGAAGGACTCATAG

D09-pFastbac-F.ab1(1>821) →

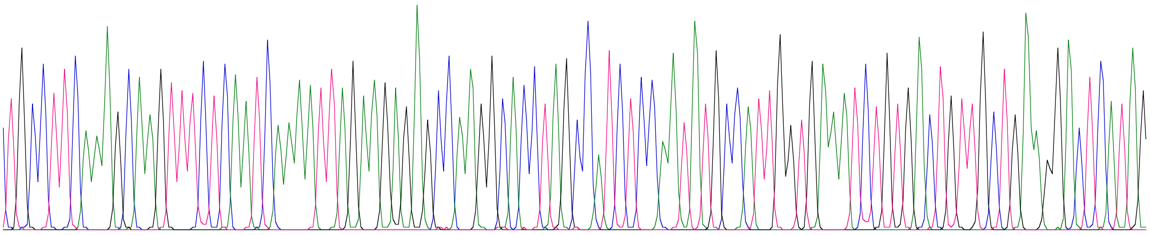

TGCCTTCAAAGCAAGTTTCTCAATCAAAATTAGAAGAGAGCCAAGGCACCTAGCCATCTCCAATATGCCATTGGTGAAATCTGTGACTGTTGCTGAAGGACTCATAG

A11-SEQR.ab1(1>815) ←

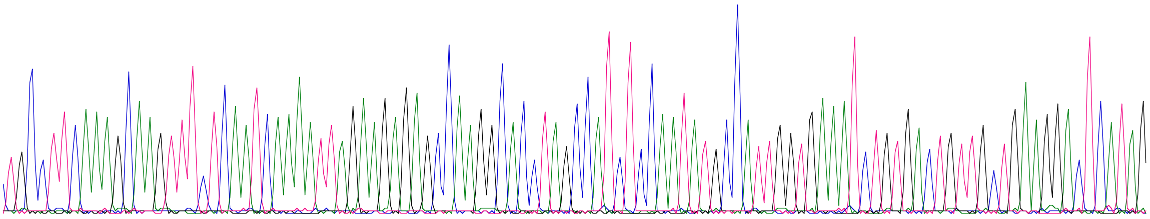

TGCCTTCAAAGCAAGTTTCTCAATCAAAATTAGAAGAGAGCCAAGGCACCTAGCCATCTCCAATATGCCATTGGTGAAATCTGTGACTGTTGCTGAAGGACTCATAG

750 760 770 780 790 800 810 820 830 840 850

TGCCTTCAAAGCAAGTTTCTCAATCAAAATTAGAAGAGAGCCAAGGCACCTAGCCATCTCCAATATGCCATTGGTGAAATCTGTGACTGTTGCTGAAGGACTCATAG

D11-SEQ1.ab1(1>821)

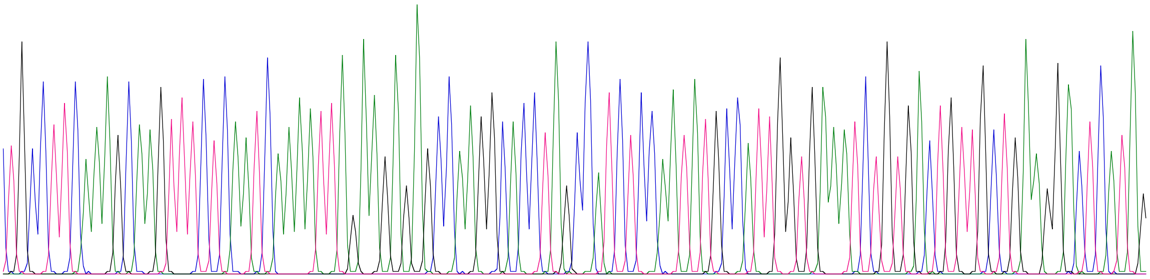

TGCCTTCAAAGCAAGTTTCTCAATCAAAATTAGAAGAGAGCCAAGGCACCTAGCCATCTCCAATATGCCATTGGTGAAATCTGTGACTGTTGCTGAAGGACTCATAG

860 870 880 890 900 910 920 930 940 950 960

AAGACCATTTTGATGTCACTGTGAAGATGAGCACCTATCTGGTGGCCTTCATCATTTCAGATTTTGAGTCTGTCAGCAAGATAACCAAGAGTGGAGTCAAGGTTTCT

U3823DH140-6.seq(1>2878) AAGACCATTTTGATGTCACTGTGAAGATGAGCACCTATCTGGTGGCCTTCATCATTTCAGATTTTGAGTCTGTCAGCAAGATAACCAAGAGTGGAGTCAAGGTTTCT

D09-pFastbac-F.ab1(1>821)

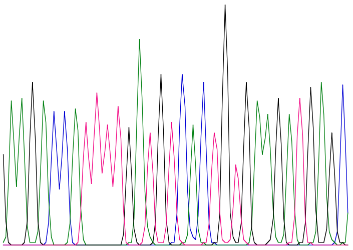

AAGACCATTTTGATGTCACTGTGAAGATGAGCA

A11-SEQR.ab1(1>815)

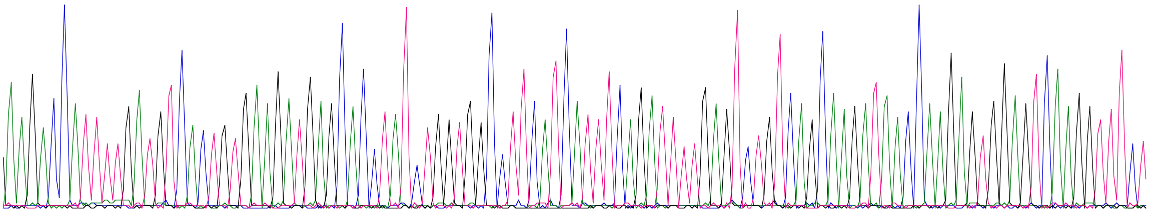

AAGACCATTTTGATGTCACTGTGAAGATGAGCACCTATCTGGTGGCCTTCATCATTTCAGATTTTGAGTCTGTCAGCAAGATAACCAAGAGTGGAGTCAAGGTTTCT

D11-SEQ1.ab1(1>821)

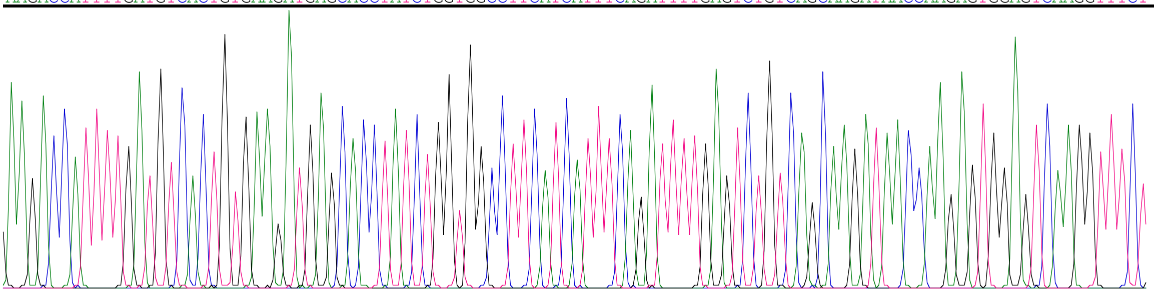

AAGACCATTTTGATGTCACTGTGAAGATGAGCACCTATCTGGTGGCCTTCATCATTTCAGATTTTGAGTCTGTCAGCAAGATAACCAAGAGTGGAGTCAAGGTTTCT

970 980 990 1,000 1,010 1,020 1,030 1,040 1,050 1,060 1,070

GTTTATGCTGTGCCAGACAAGATAAATCAAGCAGATTATGCACTGGATGCTGCGGTGACTCTTCTAGAATTTTATGAGGATTATTTCAGCATACCGTATCCCCTACC

U3823DH140-6.seq(1>2878) GTTTATGCTGTGCCAGACAAGATAAATCAAGCAGATTATGCACTGGATGCTGCGGTGACTCTTCTAGAATTTTATGAGGATTATTTCAGCATACCGTATCCCCTACC

A11-SEQR.ab1(1>815)

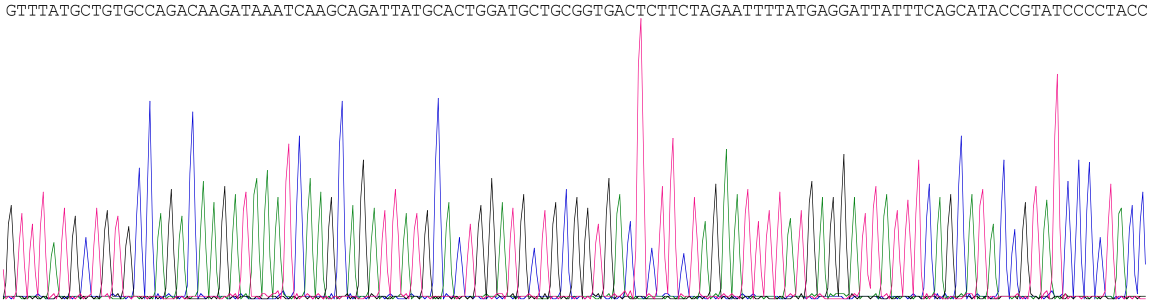

GTTTATGCTGTGCCAGACAAGATAAATCAAGCAGATTATGCACTGGATGCTGCGGTGACTCTTCTAGAATTTTATGAGGATTATTTCAGCATACCGTATCCCCTACC

970 980 990 1,000 1,010 1,020 1,030 1,040 1,050 1,060 1,070

GTTTATGCTGTGCCAGACAAGATAAATCAAGCAGATTATGCACTGGATGCTGCGGTGACTCTTCTAGAATTTTATGAGGATTATTTCAGCATACCGTATCCCCTACC

D11-SEQ1.ab1(1>821)

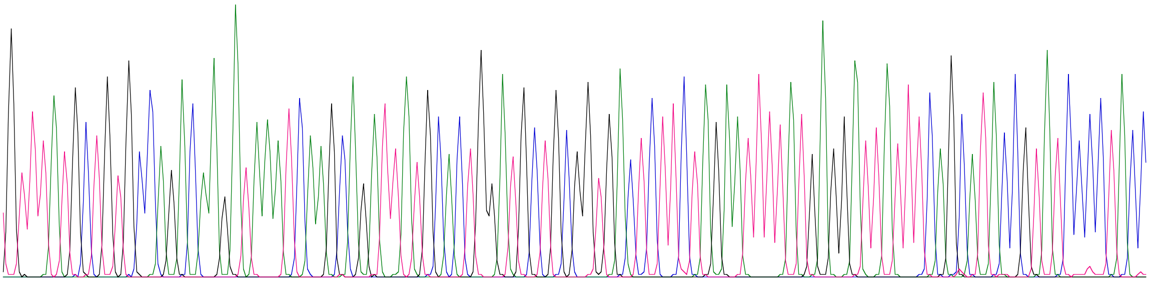

GTTTATGCTGTGCCAGACAAGATAAATCAAGCAGATTATGCACTGGATGCTGCGGTGACTCTTCTAGAATTTTATGAGGATTATTTCAGCATACCGTATCCCCTACC

A12-SEQ3R.ab1(1>811)

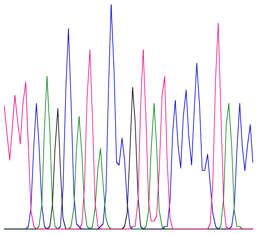

TTTCAGCATACCGTATCCCCTACC

D12-SEQ2.ab1(1>821)

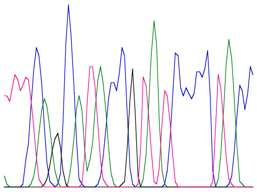

TTTCAGCATACCGTATCCCCTACC

1,080 1,090 1,100 1,110 1,120 1,130 1,140 1,150 1,160 1,170

CAAACAAGATCTTGCTGCTATTCCCGACTTTCAGTCTGGTGCTATGGAAAACTGGGGACTGACAACATATAGAGAATCTGCTCTGTTGTTTGATGCAGAAAAGTCTT

U3823DH140-6.seq(1>2878) CAAACAAGATCTTGCTGCTATTCCCGACTTTCAGTCTGGTGCTATGGAAAACTGGGGACTGACAACATATAGAGAATCTGCTCTGTTGTTTGATGCAGAAAAGTCTT

A11-SEQR.ab1(1>815)

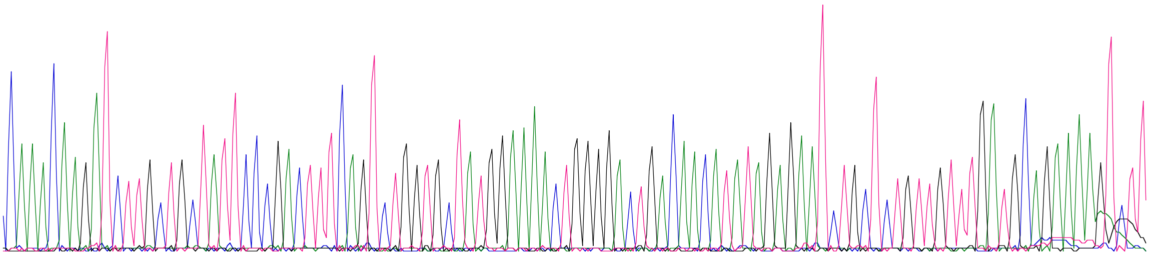

CAAACAAGATCTTGCTGCTATTCCCGACTTTCAGTCTGGTGCTATGGAAAACTGGGGACTGACAACATATAGAGAATCTGCTCTGTTGTTTGATGCAGAAAAgTCTT

D11-SEQ1.ab1(1>821)

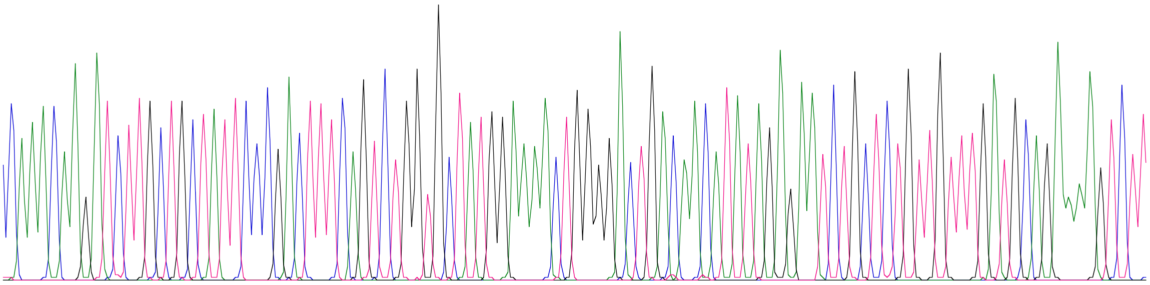

CAAACAAGATCTTGCTGCTATTCCCGACTTTCAGTCTGGTGCTATGGAAAACTGGGGACTGACAACATATAGAGAATCTGCTCTGTTGTTTGATGCAGAAAAGTCTT

A12-SEQ3R.ab1(1>811)

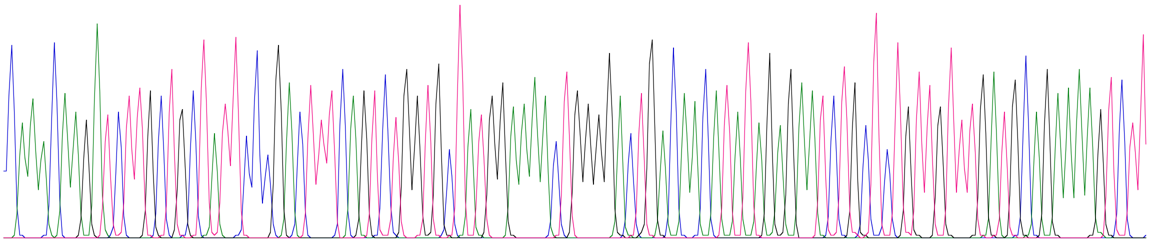

CAAACAAGATCTTGCTGCTATTCCCGACTTTCAGTCTGGTGCTATGGAAAACTGGGGACTGACAACATATAGAGAATCTGCTCTGTTGTTTGATGCAGAAAAGTCTT

1,080 1,090 1,100 1,110 1,120 1,130 1,140 1,150 1,160 1,170

CAAACAAGATCTTGCTGCTATTCCCGACTTTCAGTCTGGTGCTATGGAAAACTGGGGACTGACAACATATAGAGAATCTGCTCTGTTGTTTGATGCAGAAAAGTCTT

D12-SEQ2.ab1(1>821) →

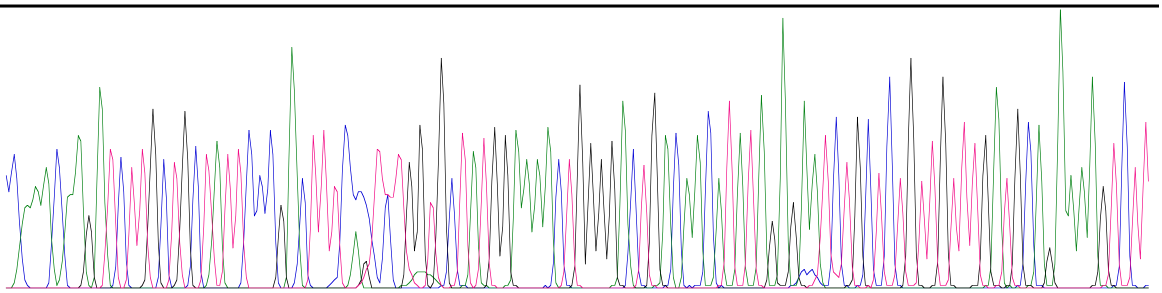

CAAACAAGATCTTGCTGCTATTCCCGACTTTCAGTCTGGTGCTATGGAAAACTGGGGACTGACAACATATAGAGAATCTGCTCTGTTGTTTGATGCAGAAAAGTCTT

1,180 1,190 1,200 1,210 1,220 1,230 1,240 1,250 1,260 1,270 1,280

CTGCATCAAGTAAGCTTGGCATCACAATGACTGTGGCCCATGAACTGGCTCACCAGTGGTTTGGGAACCTGGTCACTATGGAATGGTGGAATGATCTTTGGCTAAAT

U3823DH140-6.seq(1>2878) → CTGCATCAAGTAAGCTTGGCATCACAATGACTGTGGCCCATGAACTGGCTCACCAGTGGTTTGGGAACCTGGTCACTATGGAATGGTGGAATGATCTTTGGCTAAAT

A11-SEQR.ab1(1>815) ←

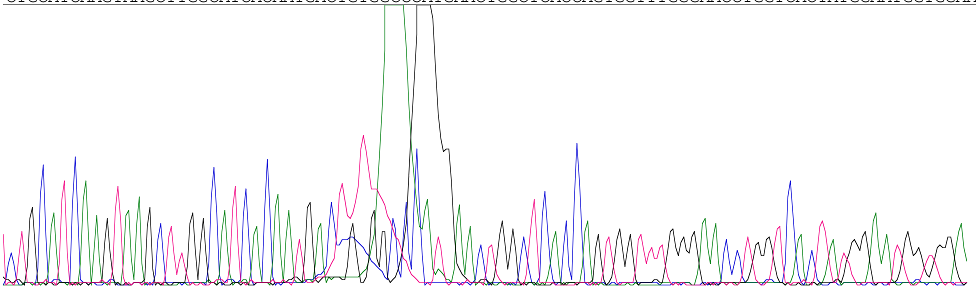

CTGCATCAAGTAAGCTTGGCATCACAATGACTGTGgcCCATGAACTGGCTCACCAGTGGTTTGGGAACCTGGTCACTATGGAATGGtGGAA

D11-SEQ1.ab1(1>821) →

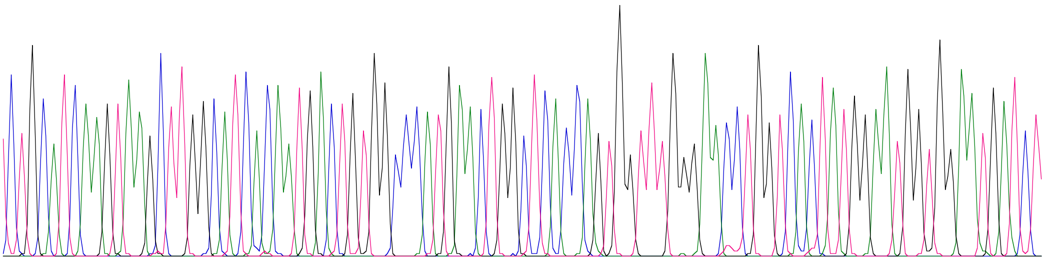

CTGCATCAAGTAAGCTTGGCATCACAATGACTGTGGCCCATGAACTGGCTCACCAGTGGTTTGGGAACCTGGTCACTATGGAATGGTGGAATGATCTT

A12-SEQ3R.ab1(1>811) ←

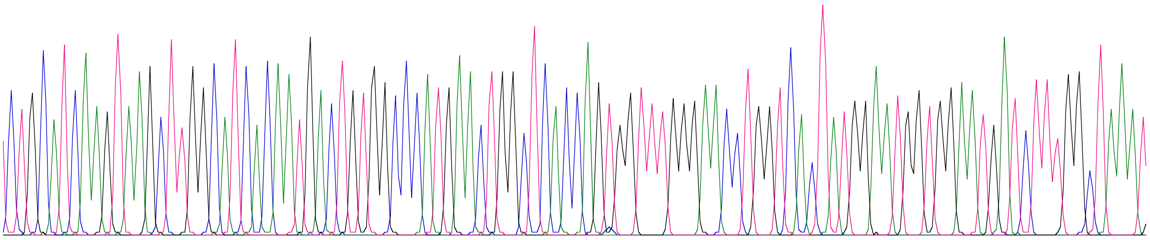

CTGCATCAAGTAAGCTTGGCATCACAATGACTGTGGCCCATGAACTGGCTCACCAGTGGTTTGGGAACCTGGTCACTATGGAATGGTGGAATGATCTTTGGCTAAAT

D12-SEQ2.ab1(1>821) →

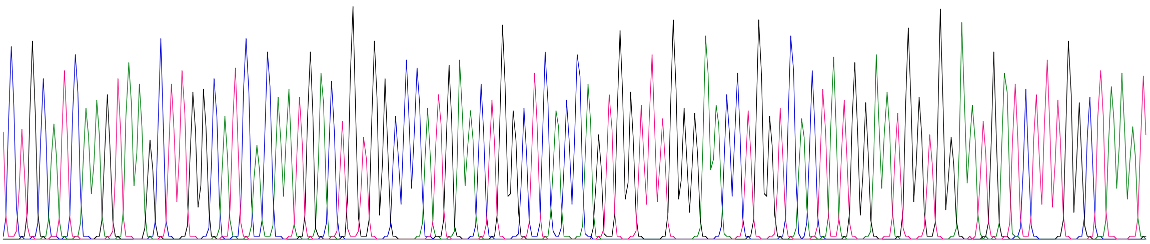

CTGCATCAAGTAAGCTTGGCATCACAATGACTGTGGCCCATGAACTGGCTCACCAGTGGTTTGGGAACCTGGTCACTATGGAATGGTGGAATGATCTTTGGCTAAAT

1,290 1,300 1,310 1,320 1,330 1,340 1,350 1,360 1,370 1,380 1,390

GAAGGATTTGCCAAATTTATGGAGTTTGTGTCTGTCAGTGTGACCCATCCTGAACTGAAAGTTGGAGATTATTTCTTTGGCAAATGTTTTGACGCAATGGAGGTAGA

U3823DH140-6.seq(1>2878) GAAGGATTTGCCAAATTTATGGAGTTTGTGTCTGTCAGTGTGACCCATCCTGAACTGAAAGTTGGAGATTATTTCTTTGGCAAATGTTTTGACGCAATGGAGGTAGA
A12-SEQ3R.ab1(1>811)

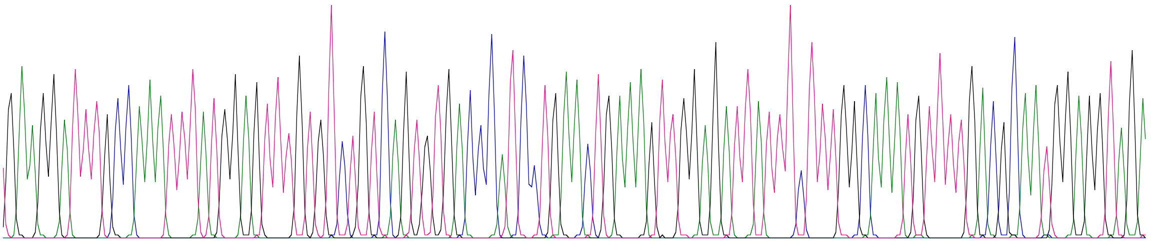

GAAGGATTTGCCAAATTTATGGAGTTTGTGTCTGTCAGTGTGACCCATCCTGAACTGAAAGTTGGAGATTATTTCTTTGGCAAATGTTTTGACGCAATGGAGGTAGA

D12-SEQ2.ab1(1>821)

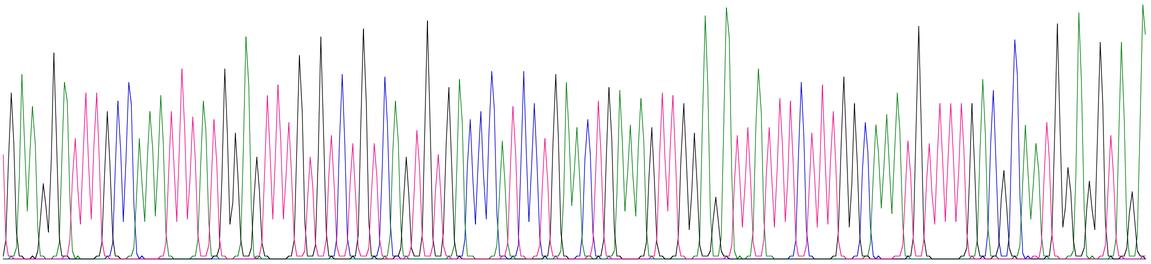

GAAGGATTTGCCAAATTTATGGAGTTTGTGTCTGTCAGTGTGACCCATCCTGAACTGAAAGTTGGAGATTATTTCTTTGGCAAATGTTTTGACGCAATGGAGGTAGA

1,400 1,410 1,420 1,430 1,440 1,450 1,460 1,470 1,480 1,490

TGCTTTAAATTCCTCACACCCTGTGTCTACACCTGTGGAAAATCCTGCTCAGATCCGGGAGATGTTTGATGATGTTTCTTATGATAAGGGAGCTTGTATTCTGAATA

U3823DH140-6.seq(1>2878) TGCTTTAAATTCCTCACACCCTGTGTCTACACCTGTGGAAAATCCTGCTCAGATCCGGGAGATGTTTGATGATGTTTCTTATGATAAGGGAGCTTGTATTCTGAATA
A12-SEQ3R.ab1(1>811)

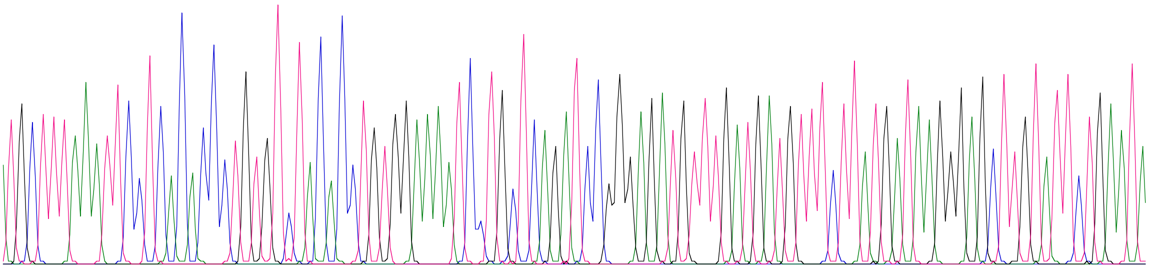

TGCTTTAAATTCCTCACACCCTGTGTCTACACCTGTGGAAAATCCTGCTCAGATCCGGGAGATGTTTGATGATGTTTCTTATGATAAGGGAGCTTGTATTCTGAATA

D12-SEQ2.ab1(1>821)

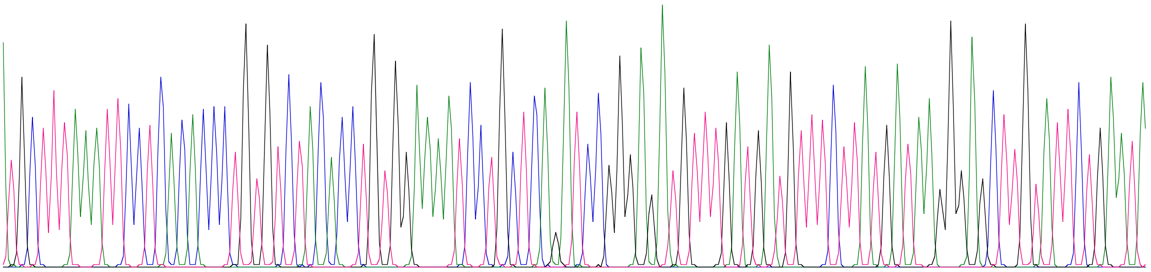

TGCTTTAAATTCCTCACACCCTGTGTCTACACCTGTGGAAAATCCTGCTCAGATCCGGGAGATGTTTGATGATGTTTCTTATGATAAGGGAGCTTGTATTCTGAATA

1,500 1,510 1,520 1,530 1,540 1,550 1,560 1,570 1,580 1,590 1,600

TGCTAAGGGAGTATCTTAGTGCTGACGCATTTAAAAGTGGTATTGTACAGTATCTCCAGAAGCATAGCTATAAAAATACAAAAAACGAGGACCTGTGGGATAGTATG

U3823DH140-6.seq(1>2878) TGCTAAGGGAGTATCTTAGTGCTGACGCATTTAAAAGTGGTATTGTACAGTATCTCCAGAAGCATAGCTATAAAAATACAAAAAACGAGGACCTGTGGGATAGTATG
A12-SEQ3R.ab1(1>811)

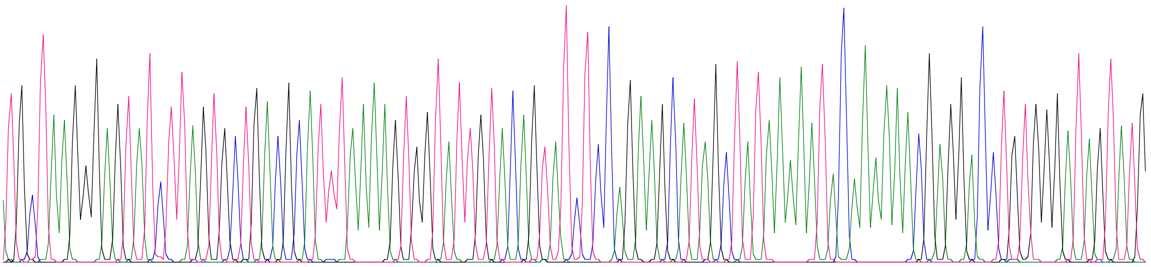

TGCTAAGGGAGTATCTTAGTGCTGACGCATTTAAAAGTGGTATTGTACAGTATCTCCAGAAGCATAGCTATAAAAATACAAAAAACGAGGACCTGTGGGATAGTATG

1,500 1,510 1,520 1,530 1,540 1,550 1,560 1,570 1,580 1,590 1,600

TGCTAAGGGAGTATCTTAGTGCTGACGCATTTAAAAGTGGTATTGTACAGTATCTCCAGAAGCATAGCTATAAAAATACAAAAAACGAGGACCTGTGGGATAGTATG

D12-SEQ2.ab1(1>821)

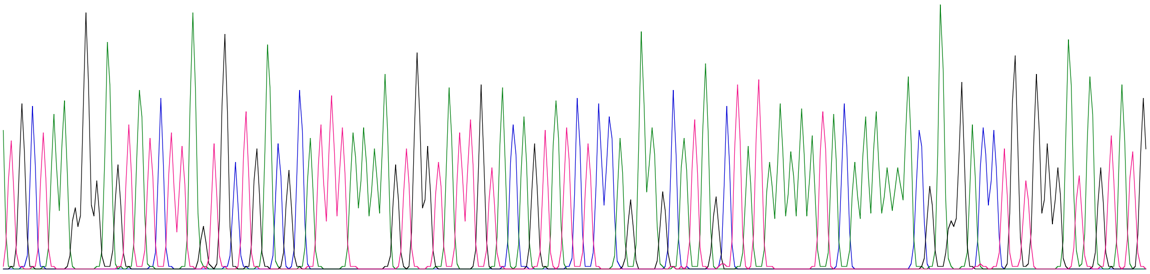

TGCTAAGGGAGTATCTTAGTGCTGACGCATTTAAAAGTGGTATTGTACAGTATCTCCAGAAGCATAGCTATAAAAATACAAAAAACGAGGACCTGTGGGATAGTATG

1,610 1,620 1,630 1,640 1,650 1,660 1,670 1,680 1,690 1,700 1,710

GCAAGTATTTGCCCTACAGATGGTGTAAAAGGGATGGATGGCTTTTGCTCTAGAAGTCAACATTCATCTTCATCCTCACATTGGCATCAGGAAGGGGTGGATGTGAA

U3823DH140-6.seq(1>2878) GCAAGTATTTGCCCTACAGATGGTGTAAAAGGGATGGATGGCTTTTGCTCTAGAAGTCAACATTCATCTTCATCCTCACATTGGCATCAGGAAGGGGTGGATGTGAA

A12-SEQ3R.ab1(1>811)

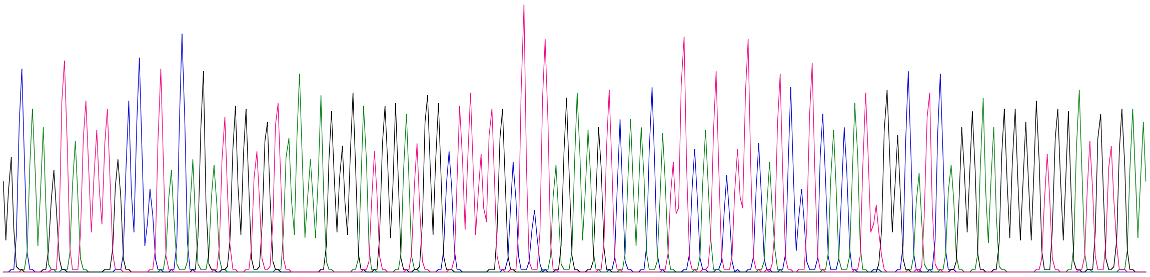

GCAAGTATTTGCCCTACAGATGGTGTAAAAGGGATGGATGGCTTTTGCTCTAGAAGTCAACATTCATCTTCATCCTCACATTGGCATCAGGAAGGGGTGGATGTGAA

D12-SEQ2.ab1(1>821)

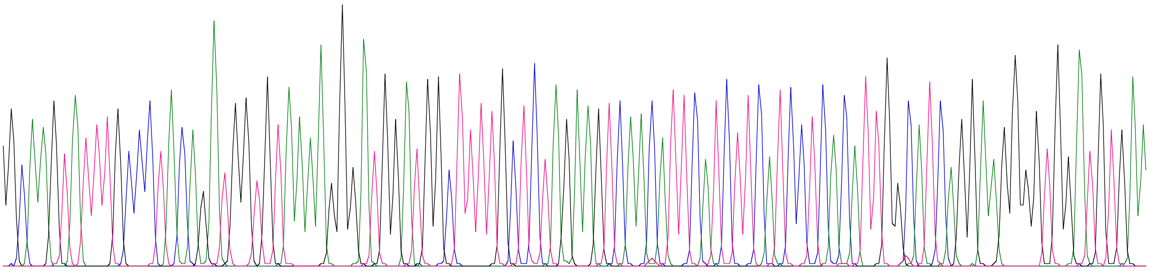

GCAAGTATTTGCCCTACAGATGGTGTAAAAGGGATGGATGGCTTTTGCTCTAGAAGTCAACATTCATCTTCATCCTCACATTGGCATCAGGAAGGGGTGGATGTGAA

B08-SEQ4RN.ab1(1>814)

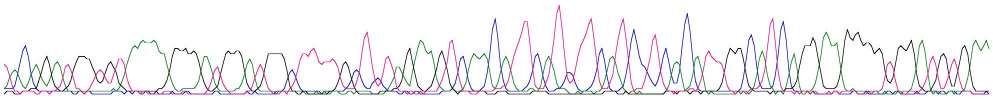

TACAGATGGTGTAAAAGGGATGGATGGCTTTTGCTCTAGAAGTCAACATTCATCTTCATCCTCACATTGGCATCAGGAAGGGGTGGATGTGAA

E01-SEQ3.ab1(1>821)

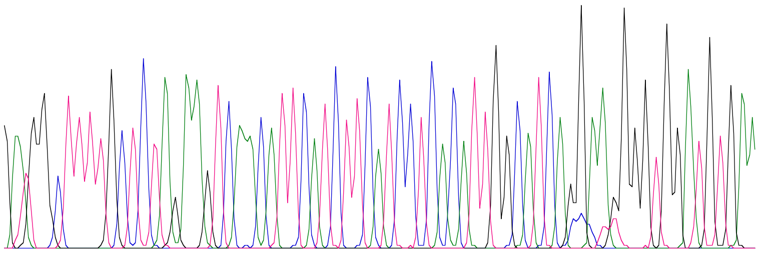

GATGGCTTTTGCTCTAGAAGTCAACATTCATCTTCATCCTCACATTGGCATCAGGAAGGGGTGGATGTGAA

K528R

1,720 1,730 1,740 1,750 1,760 1,770 1,780 1,790 1,800 1,810

AACCATGATGAACACTTGGACACTGCAGCGTGGTTTTCCCCTAATAACCATCACAGTGAGGGGGAGGAATGTACACATGAAGCAAGAGCACTACATGAAGGGCTCTG

U3823DH140-6.seq(1>2878) AACCATGATGAACACTTGGACACTGCAGCGTGGTTTTCCCCTAATAACCATCACAGTGAGGGGGAGGAATGTACACATGAAGCAAGAGCACTACATGAAGGGCTCTG

A12-SEQ3R.ab1(1>811)

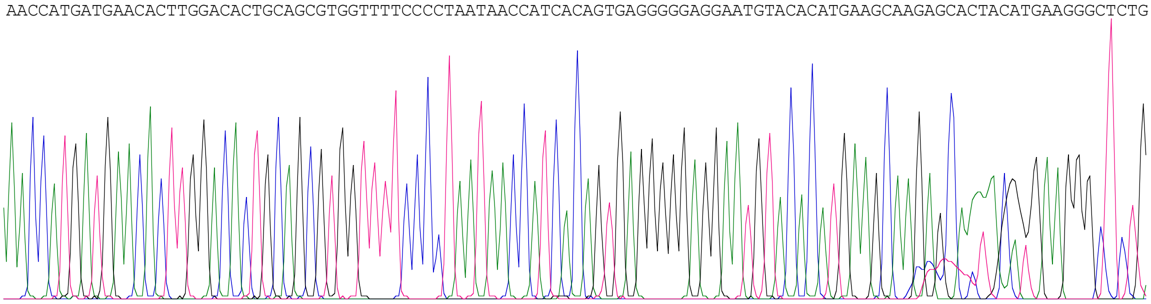

AACCATGATGAACACTTGGACACTGCAGCGTGGTTTTCCCCTAATAACCATCACAGTGAGGGGGAGGAATGTACACATGAAGCAAGAGCACTACATGAAGGGCTCTG

D12-SEQ2.ab1(1>821)

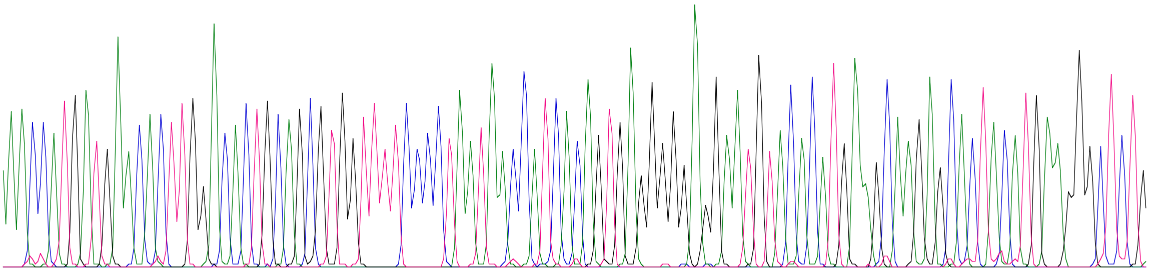

AACCATGATGAACACTTGGACACTGCAGCGTGGTTTTCCCCTAATAACCATCACAGTGAGGGGGAGGAATGTACACATGAAGCAAGAGCACTACATGAAGGGCTCTG

B08-SEQ4RN.ab1(1>814)

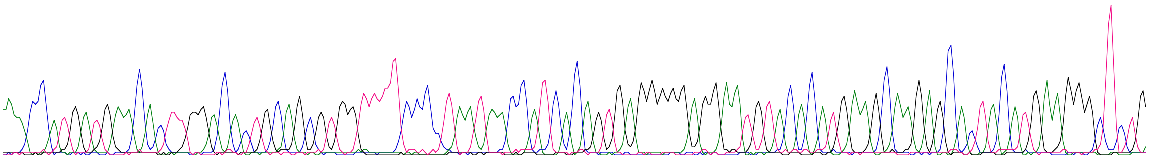

AACCATGATGAACACTTGGACACTGCAGCGTGGTTTTCCCCTAATAACCATCACAGTGAGGGGGAGGAATGTACACATGAAGCAAGAGCACTACATGAAGGGCTCTG

E01-SEQ3.ab1(1>821)

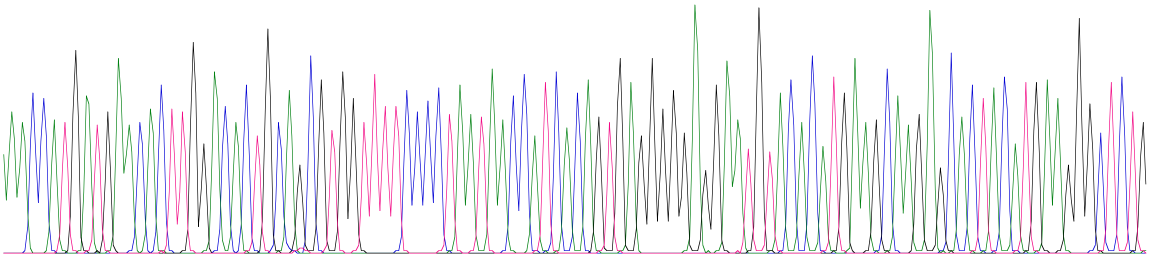

AACCATGATGAACACTTGGACACTGCAGCGTGGTTTTCCCCTAATAACCATCACAGTGAGGGGGAGGAATGTACACATGAAGCAAGAGCACTACATGAAGGGCTCTG

1,820 1,830 1,840 1,850 1,860 1,870 1,880 1,890 1,900 1,910 1,920

ACGGCGCCCCGGACACTGGGTACCTGTGGCATGTTCCATTGACATTCATCACCAGCAAATCCGACATGGTCCATCGATTTTTGCTAAAAACAAAAACAGATGTGCTC

U3823DH140-6.seq(1>2878) ACGGCGCCCCGGACACTGGGTACCTGTGGCATGTTCCATTGACATTCATCACCAGCAAATCCGACATGGTCCATCGATTTTTGCTAAAAACAAAAACAGATGTGCTC

A12-SEQ3R.ab1(1>811)

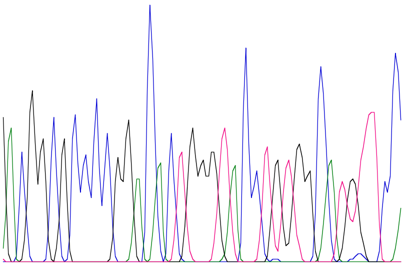

ACGGCGCCCCGGACACTGGGTACCTGTGGCATGTTCCA

D12-SEQ2.ab1(1>821)

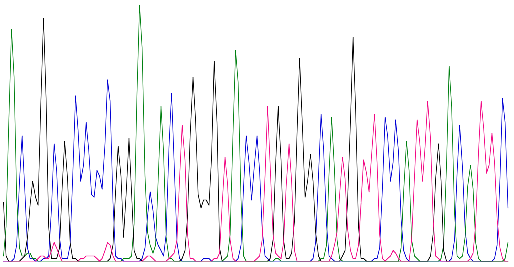

ACGGCGCCCCGGACACTGGGTACCTGTGGCATGTTCCATTGACATTCA

1,820 1,830 1,840 1,850 1,860 1,870 1,880 1,890 1,900 1,910 1,920

ACGGCGCCCCGGACACTGGGTACCTGTGGCATGTTCCATTGACATTCATCACCAGCAAATCCGACATGGTCCATCGATTTTTGCTAAAAACAAAAACAGATGTGCTC

B08-SEQ4RN.ab1(1>814)

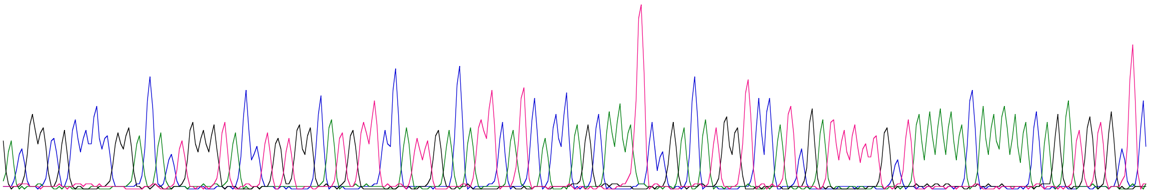

ACGGCGCCCCGGACACTGGGTACCTGTGGCATGTTCCATTGACATTCATCACCAGCAAATCCGACATGGTCCATCGATTTTTGCTAAAAACAAAAACAGATGTGCTC

E01-SEQ3.ab1(1>821)

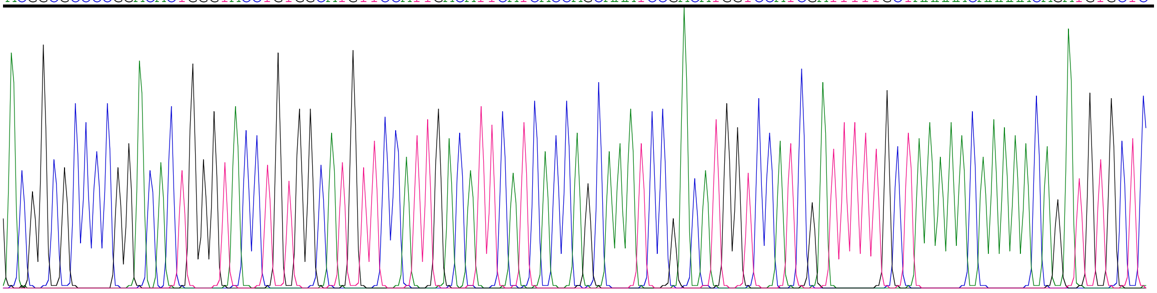

ACGGCGCCCCGGACACTGGGTACCTGTGGCATGTTCCATTGACATTCATCACCAGCAAATCCGACATGGTCCATCGATTTTTGCTAAAAACAAAAACAGATGTGCTC

1,930 1,940 1,950 1,960 1,970 1,980 1,990 2,000 2,010 2,020 2,030

ATCCTCCCAGAAGAGGTGGAATGGATCAAATTTAATGTGGGCATGAATGGCTATTACATTGTGCATTACGAGGATGATGGATGGGACTCTTTGACTGGCCTTTTAAA

U3823DH140-6.seq(1>2878)
B08-SEQ4RN.ab1(1>814)

ATCCTCCCAGAAGAGGTGGAATGGATCAAATTTAATGTGGGCATGAATGGCTATTACATTGTGCATTACGAGGATGATGGATGGGACTCTTTGACTGGCCTTTTAAA

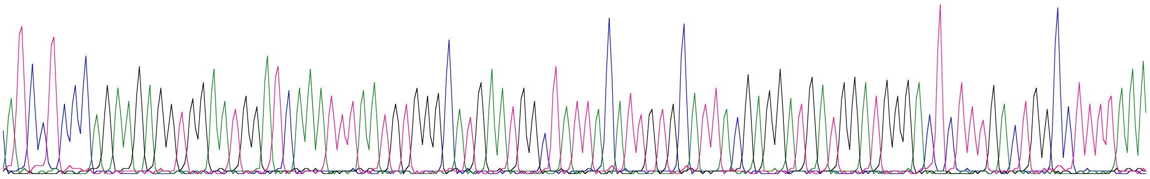

ATCCTCCCAGAAGAGGTGGAATGGATCAAATTTAATGTGGGCATGAATGGCTATTACATTGTGCATTACGAGGATGATGGATGGGACTCTTTGACTGGCCTTTTAAA

E01-SEQ3.ab1(1>821)

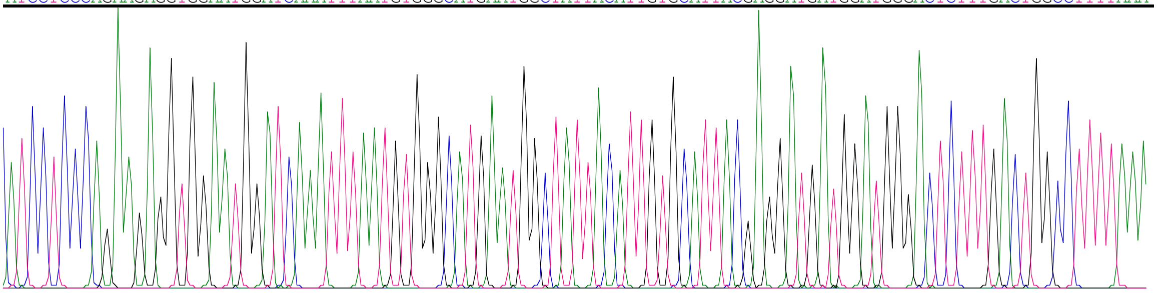

ATCCTCCCAGAAGAGGTGGAATGGATCAAATTTAATGTGGGCATGAATGGCTATTACATTGTGCATTACGAGGATGATGGATGGGACTCTTTGACTGGCCTTTTAAA

2,040 2,050 2,060 2,070 2,080 2,090 2,100 2,110 2,120 2,130 2,140

AGGAACACACACAGCAGTCAGCAGTAATGATCGGGCGAGTCTCATTAACAATGCATTTCAGCTCGTCAGCATTGGGAAGCTGTCCATTGAAAAGGCCTTGGATTTAT

U3823DH140-6.seq(1>2878)
B08-SEQ4RN.ab1(1>814)

AGGAACACACACAGCAGTCAGCAGTAATGATCGGGCGAGTCTCATTAACAATGCATTTCAGCTCGTCAGCATTGGGAAGCTGTCCATTGAAAAGGCCTTGGATTTAT

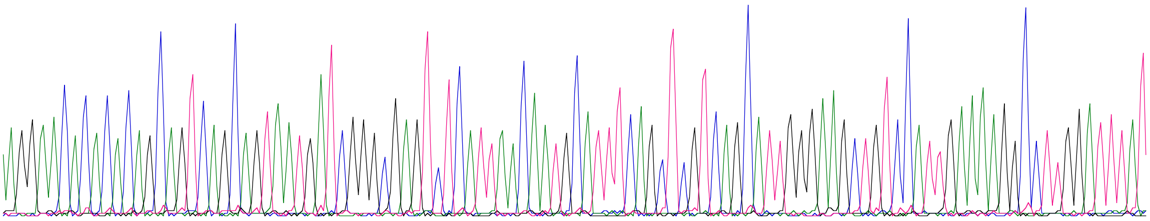

AGGAACACACACAGCAGTCAGCAGTAATGATCGGGCGAGTCTCATTAACAATGCATTTCAGCTCGTCAGCATTGGGAAGCTGTCCATTGAAAAGGCCTTGGATTTAT

2,040 2,050 2,060 2,070 2,080 2,090 2,100 2,110 2,120 2,130 2,140

AGGAACACACACAGCAGTCAGCAGTAATGATCGGGCGAGTCTCATTAACAATGCATTTCAGCTCGTCAGCATTGGGAAGCTGTCCATTGAAAAGGCCTTGGATTTAT

E01-SEQ3.ab1(1>821) →

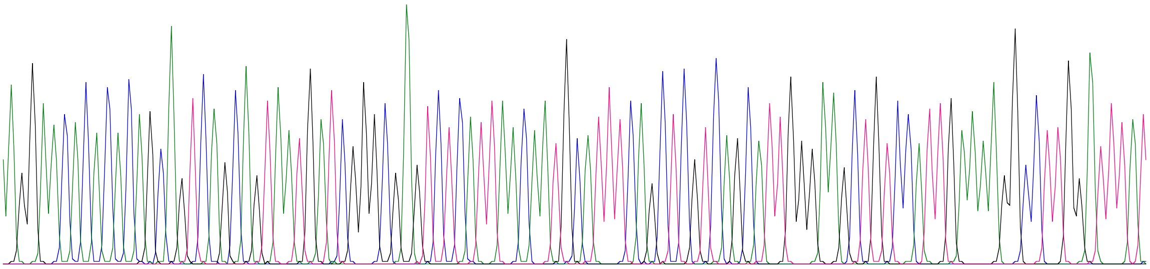

AGGAACACACACAGCAGTCAGCAGTAATGATCGGGCGAGTCTCATTAACAATGCATTTCAGCTCGTCAGCATTGGGAAGCTGTCCATTGAAAAGGCCTTGGATTTAT

2,150 2,160 2,170 2,180 2,190 2,200 2,210 2,220 2,230 2,240

CCCTGTACTTGAAACATGAAACTGAAATTATGCCCGTGTTTCAAGGTTTGAATGAGCTGATTCCTATGTATAAGTTAATGGAGAAAAGAGATATGAATGAAGTGGAA

U3823DH140-6.seq(1>2878) → CCCTGTACTTGAAACATGAAACTGAAATTATGCCCGTGTTTCAAGGTTTGAATGAGCTGATTCCTATGTATAAGTTAATGGAGAAAAGAGATATGAATGAAGTGGAA

B08-SEQ4RN.ab1(1>814) ←

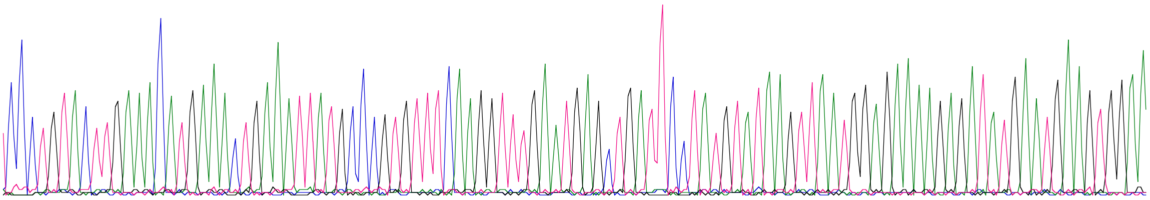

CCCTGTACTTGAAACATGAAACTGAAATTATGCCCGTGTTTCAAGGTTTGAATGAGCTGATTCCTATGTATAAGTTAATGGAGAAAAGAGATATGAATGAAGTGGAA

E01-SEQ3.ab1(1>821) →

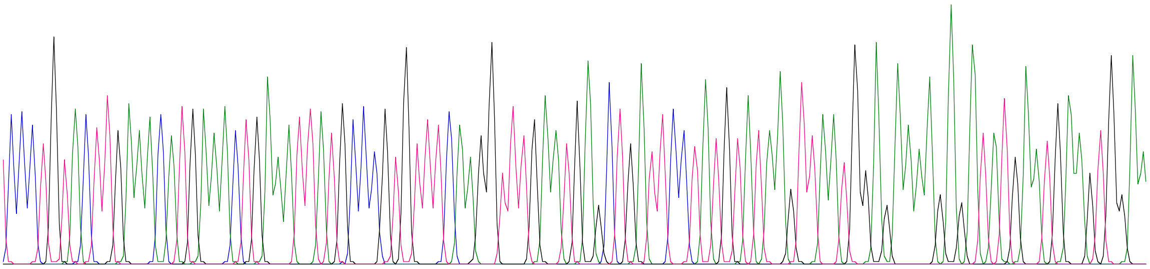

CCCTGTACTTGAAACATGAAACTGAAATTATGCCCGTGTTTCAAGGTTTGAATGAGCTGATTCCTATGTATAAGTTAATGGAGAAAAGAGATATGAATGAAGTGGAA

E02-SEQ4.ab1(1>821) →

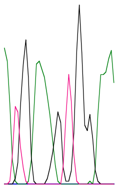

ATGAAGTGGAA

Q730E

2,250 2,260 2,270 2,280 2,290 2,300 2,310 2,320 2,330 2,340 2,350

ACTCAATTCAAGGCCTTCCTCATCAGGCTGCTAAGGGACCTCATTGATAAGCAGACATGGACAGACGAGGGCTCAGTCTCAGAGCGAATGCTGCGGAGTGAGCTACT

U3823DH140-6.seq(1>2878) → ACTCAATTCAAGGCCTTCCTCATCAGGCTGCTAAGGGACCTCATTGATAAGCAGACATGGACAGACGAGGGCTCAGTCTCAGAGCGAATGCTGCGGAGTGAGCTACT

B08-SEQ4RN.ab1(1>814) ←

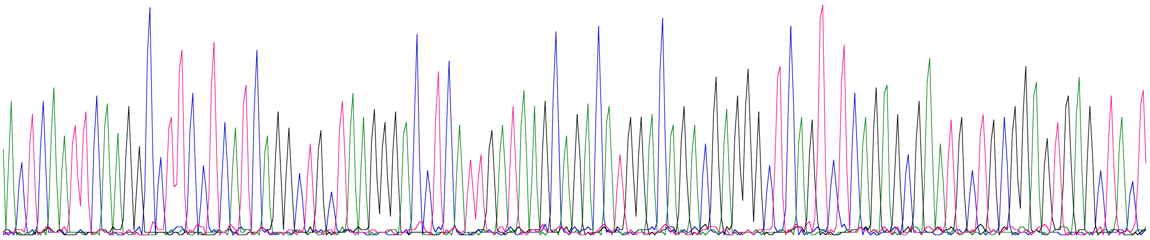

ACTCAATTCAAGGCCTTCCTCATCAGGCTGCTAAGGGACCTCATTGATAAGCAGACATGGACAGACGAGGGCTCAGTCTCAGAGCGAATGCTGCGGAGTGAGCTACT

Q730E

2,250 2,260 2,270 2,280 2,290 2,300 2,310 2,320 2,330 2,340 2,350

ACTCAATTCAAGGCCTTCCTCATCAGGCTGCTAAGGGACCTCATTGATAAGCAGACATGGACAGACGAGGGCTCAGTCTCAGAGCGAATGCTGCGGAGTGAGCTACT

E01-SEQ3.ab1(1>821)

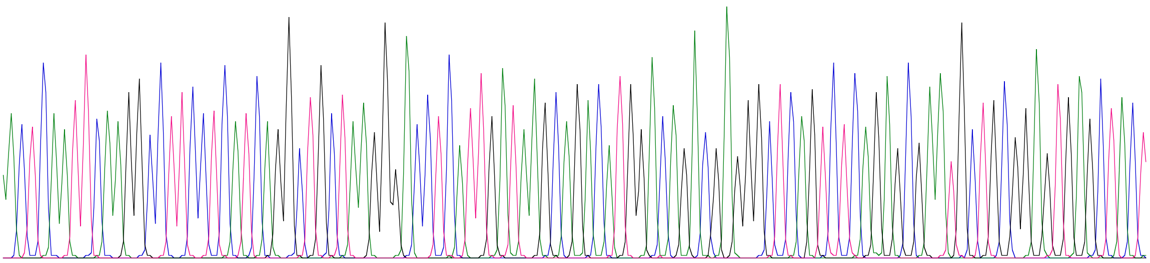

ACTCAATTCAAGGCCTTCCTCATCAGGCTGCTAAGGGACCTCATTGATAAGCAGACATGGACAGACGAGGGCTCAGTCTCAGAGCGAATGCTGCGGAGTGAGCTACT

E02-SEQ4.ab1(1>821)

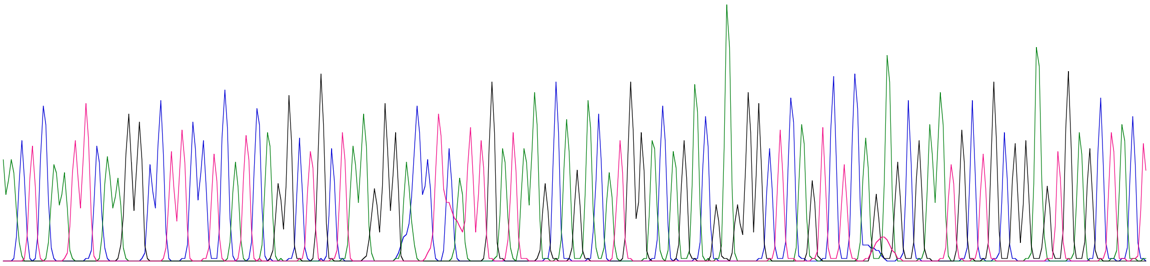

ACTCAATTCAAGGCCTTCCTCATCAGGCTGCTAAGGGACCTCATTGATAAGCAGACATGGACAGACGAGGGCTCAGTCTCAGAGCGAATGCTGCGGAGTGAGCTACT

D10-PFastBac1-R.ab1(1>821)

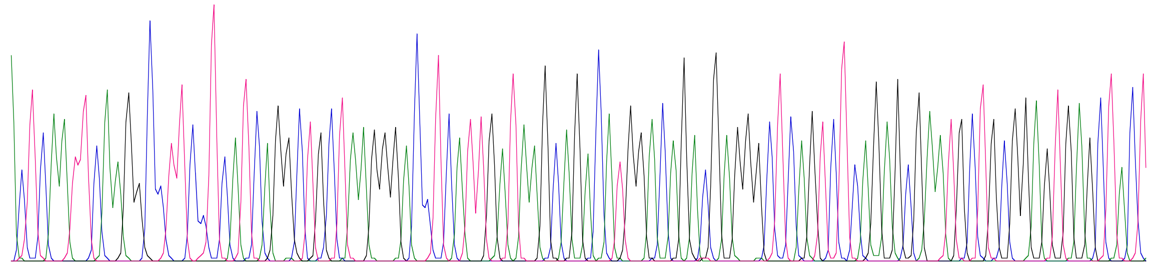

ACTCAATTCAAGGCCTTCCTCATCAGGCTGCTAAGGGACCTCATTGATAAGCAGACATGGACAGACGAGGGCTCAGTCTCAGAGCGAATGCTGCGGAGTGAGCTACT

2,360 2,370 2,380 2,390 2,400 2,410 2,420 2,430 2,440 2,450 2,460

ACTCCTCGCCTGTGTGCACAACTATCAGCCGTGCGTACAGAGGGCAGAAGGCTATTTCAGAAAGTGGAAGGAATCCAATGGAAACTTGAGCCTGCCTGTCGACGTGA

U3823DH140-6.seq(1>2878) ACTCCTCGCCTGTGTGCACAACTATCAGCCGTGCGTACAGAGGGCAGAAGGCTATTTCAGAAAGTGGAAGGAATCCAATGGAAACTTGAGCCTGCCTGTCGACGTGA

B08-SEQ4RN.ab1(1>814)

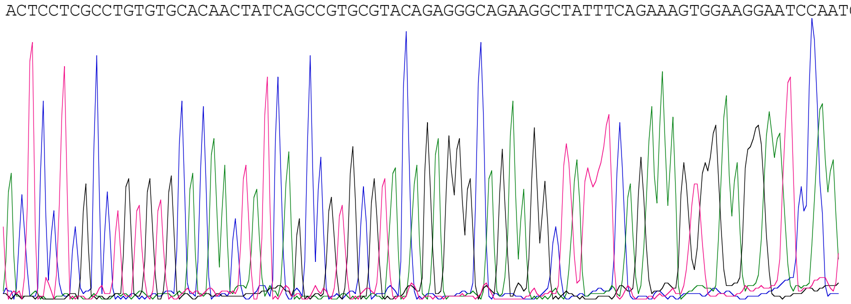

ACTCCTCGCCTGTGTGCACAACTATCAGCCGTGCGTACAGAGGGCAGAAGGCTATtTCAGAAAGtGGAAGGAATCCAAT

E01-SEQ3.ab1(1>821)

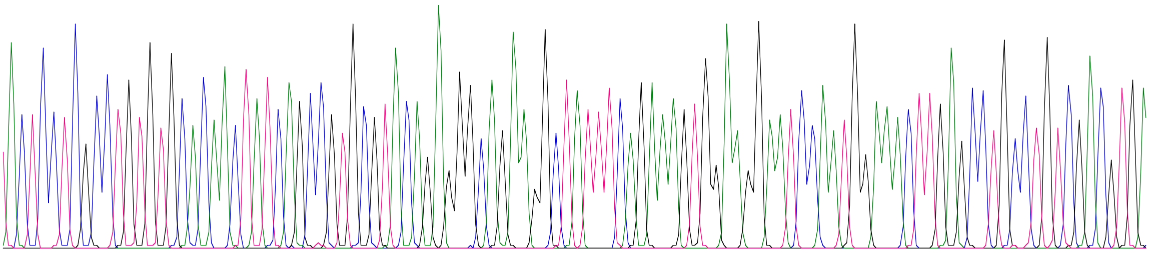

ACTCCTCGCCTGTGTGCACAACTATCAGCCGTGCGTACAGAGGGCAGAAGGCTATTTCAGAAAGTGGAAGGAATCCAATGGAAACTTGAGCCTGCCTGTCGACGTGA

E02-SEQ4.ab1(1>821)

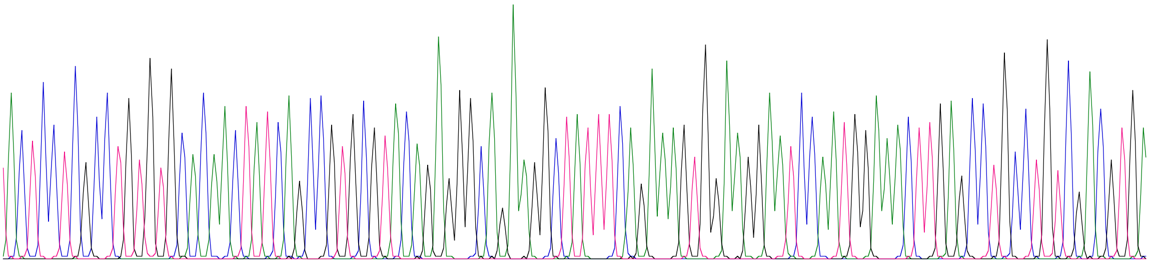

ACTCCTCGCCTGTGTGCACAACTATCAGCCGTGCGTACAGAGGGCAGAAGGCTATTTCAGAAAGTGGAAGGAATCCAATGGAAACTTGAGCCTGCCTGTCGACGTGA

2,360 2,370 2,380 2,390 2,400 2,410 2,420 2,430 2,440 2,450 2,460

ACTCCTCGCCTGTGTGCACAACTATCAGCCGTGCGTACAGAGGGCAGAAGGCTATTTCAGAAAGTGGAAGGAATCCAATGGAAACTTGAGCCTGCCTGTCGACGTGA

D10-PFastBac1-R.ab1(1>821)

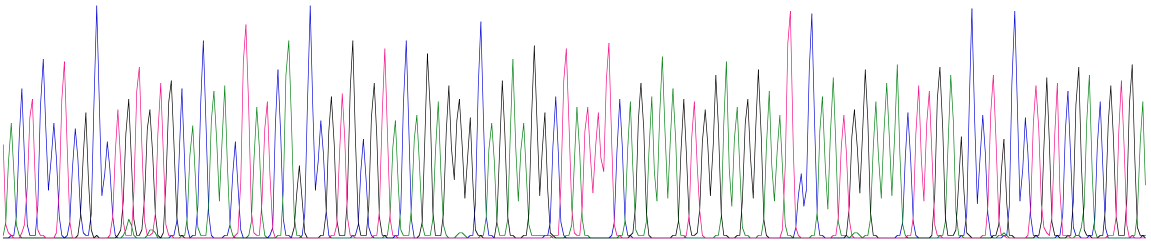

ACTCCTCGCCTGTGTGCACAACTATCAGCCGTGCGTACAGAGGGCAGAAGGCTATTTCAGAAAGTGGAAGGAATCCAATGGAAACTTGAGCCTGCCTGTCGACGTGA

2,470 2,480 2,490 2,500 2,510 2,520 2,530 2,540 2,550 2,560

CCTTGGCAGTGTTTGCTGTGGGGGCCCAGAGCACAGAAGGCTGGGATTTTCTTTATAGTAAATATCAGTTTTCTTTGTCCAGTACTGAGAAAAGCCAAATTGAATTT

U3823DH140-6.seq(1>2878) CCTTGGCAGTGTTTGCTGTGGGGGCCCAGAGCACAGAAGGCTGGGATTTTCTTTATAGTAAATATCAGTTTTCTTTGTCCAGTACTGAGAAAAGCCAAATTGAATTT

E01-SEQ3.ab1(1>821)

C

E02-SEQ4.ab1(1>821)

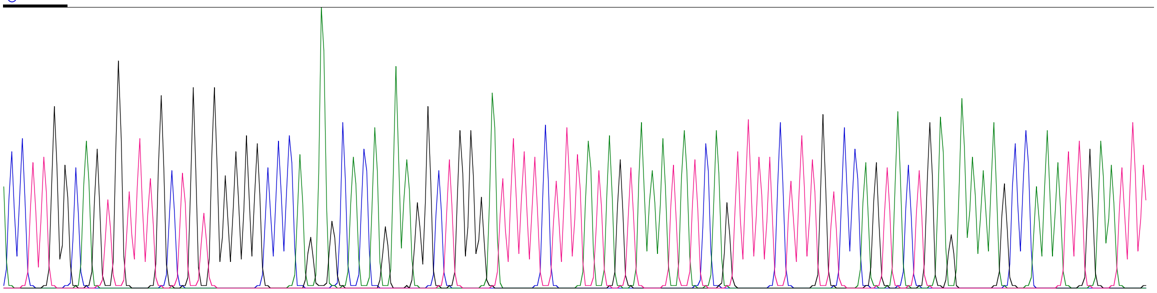

CCTTGGCAGTGTTTGCTGTGGGGGCCCAGAGCACAGAAGGCTGGGATTTTCTTTATAGTAAATATCAGTTTTCTTTGTCCAGTACTGAGAAAAGCCAAATTGAATTT

D10-PFastBac1-R.ab1(1>821)

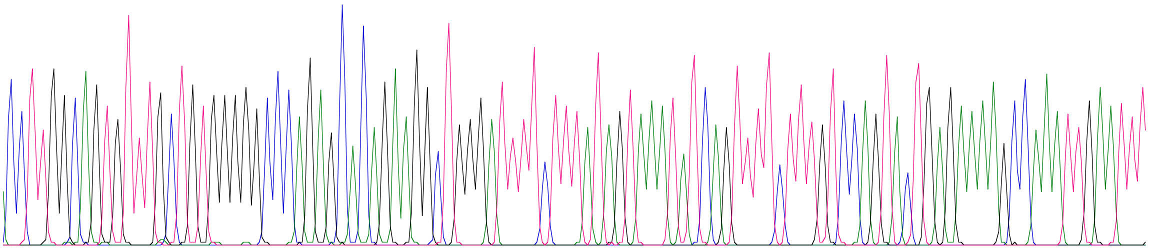

CCTTGGCAGTGTTTGCTGTGGGGGCCCAGAGCACAGAAGGCTGGGATTTTCTTTATAGTAAATATCAGTTTTCTTTGTCCAGTACTGAGAAAAGCCAAATTGAATTT

2,570 2,580 2,590 2,600 2,610 2,620 2,630 2,640 2,650 2,660 2,670

GCCCTCTGCAGAACCCAAAATAAGGAAAAGCTTCAATGGCTACTAGATGAAAGCTTTAAGGGAGATAAAATAAAAACTCAGGAGTTTCCACAAATTCTTACACTCAT

U3823DH140-6.seq(1>2878) GCCCTCTGCAGAACCCAAAATAAGGAAAAGCTTCAATGGCTACTAGATGAAAGCTTTAAGGGAGATAAAATAAAAACTCAGGAGTTTCCACAAATTCTTACACTCAT

E02-SEQ4.ab1(1>821)

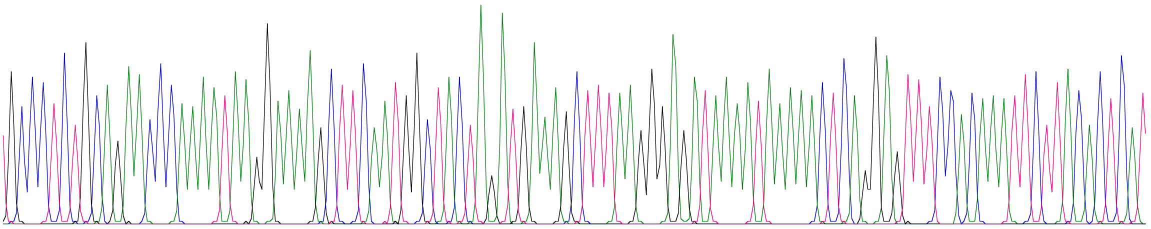

GCCCTCTGCAGAACCCAAAATAAGGAAAAGCTTCAATGGCTACTAGATGAAAGCTTTAAGGGAGATAAAATAAAAACTCAGGAGTTTCCACAAATTCTTACACTCAT

2,570 2,580 2,590 2,600 2,610 2,620 2,630 2,640 2,650 2,660 2,670

GCCCTCTGCAGAACCCAAAATAAGGAAAAGCTTCAATGGCTACTAGATGAAAGCTTTAAGGGAGATAAAATAAAAACTCAGGAGTTTCCACAAATTCTTACACTCAT

D10-PFastBac1-R.ab1(1>821)

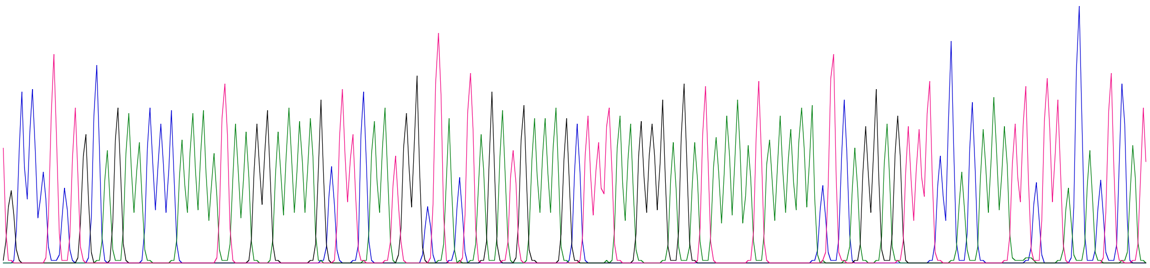

GCCCTCTGCAGAACCCAAAATAAGGAAAAGCTTCAATGGCTACTAGATGAAAGCTTTAAGGGAGATAAAATAAAAACTCAGGAGTTTCCACAAATTCTTACACTCAT

2,680 2,690 2,700 2,710 2,720 2,730 2,740 2,750 2,760 2,770 2,780

TGGCAGGAACCCAGTAGGATACCCACTGGCCTGGCAATTTCTGAGGAAAAACTGGAACAAACTTGTACAAAAGTTTGAACTTGGCTCATCTTCCATAGCCCACATGG

U3823DH140-6.seq(1>2878) TGGCAGGAACCCAGTAGGATACCCACTGGCCTGGCAATTTCTGAGGAAAAACTGGAACAAACTTGTACAAAAGTTTGAACTTGGCTCATCTTCCATAGCCCACATGG
E02-SEQ4.ab1(1>821)

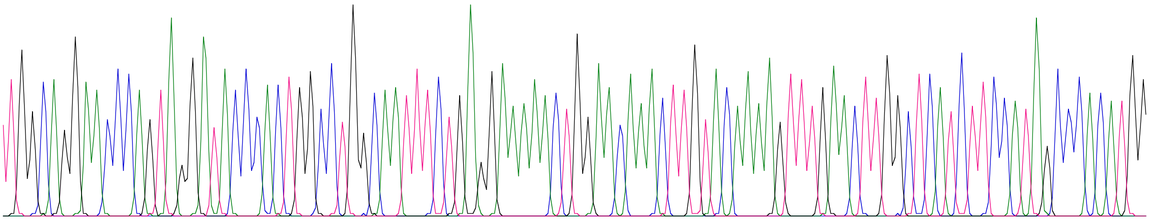

TGGCAGGAACCCAGTAGGATACCCACTGGCCTGGCAATTTCTGAGGAAAAACTGGAACAAACTTGTACAAAAGTTTGAACTTGGCTCATCTTCCATAGCCCACATGG

D10-PFastBac1-R.ab1(1>821)

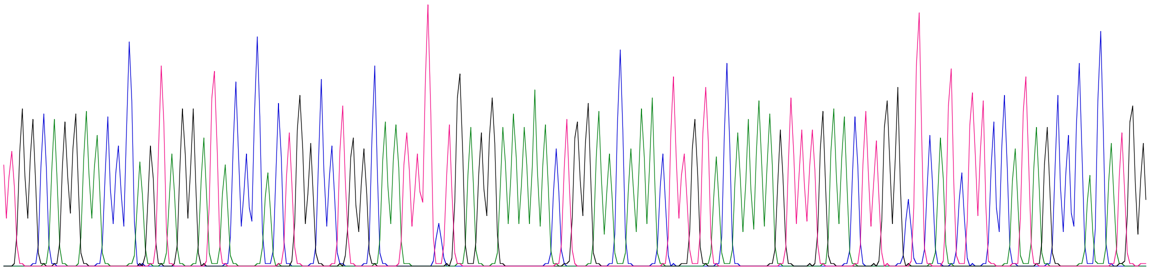

TGGCAGGAACCCAGTAGGATACCCACTGGCCTGGCAATTTCTGAGGAAAAACTGGAACAAACTTGTACAAAAGTTTGAACTTGGCTCATCTTCCATAGCCCACATGG

2,790 2,800 2,810 2,820 2,830 2,840 2,850 2,860 2,870 2,880

TAATGGGTACAACAAATCAATTCTCCACAAGAACACGGCTTGAAGAGGTAAAAGGATTCTTCAGCTCTTTGAAAGAAAATGGTTCTCAGCTCCGTTGTGTCCAACAG

U3823DH140-6.seq(1>2878) TAATGGGTACAACAAATCAATTCTCCACAAGAACACGGCTTGAAGAGGTAAAAGGATTCTTCAGCTCTTTGAAAGAAAATGGTTCTCAGCTCCGTTGTGTCCAACAG
E02-SEQ4.ab1(1>821)

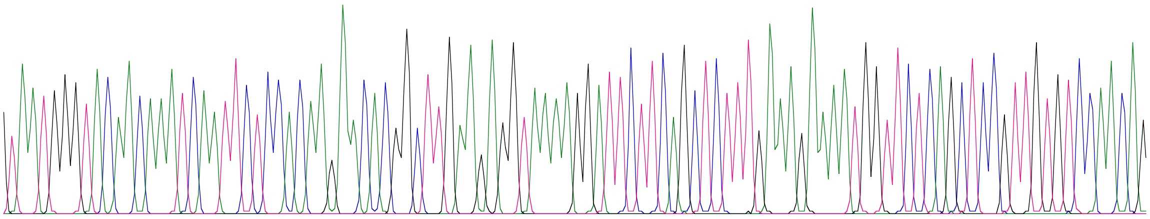

TAATGGGTACAACAAATCAATTCTCCACAAGAACACGGCTTGAAGAGGTAAAAGGATTCTTCAGCTCTTTGAAAGAAAATGGTTCTCAGCTCCGTTGTGTCCAACAG

D10-PFastBac1-R.ab1(1>821)

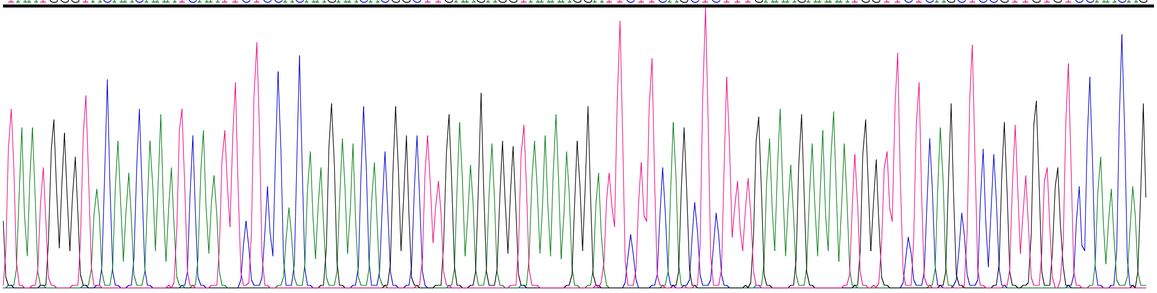

TAATGGGTACAACAAATCAATTCTCCACAAGAACACGGCTTGAAGAGGTAAAAGGATTCTTCAGCTCTTTGAAAGAAAATGGTTCTCAGCTCCGTTGTGTCCAACAG

2,790 2,800 2,810 2,820 2,830 2,840 2,850 2,860 2,870 2,880

TAATGGGTACAACAAATCAATTCTCCACAAGAACACGGCTTGAAGAGGTAAAAGGATTCTTCAGCTCTTTGAAAGAAAATGGTTCTCAGCTCCGTTGTGTCCAACAG

A09-SEQ5.ab1(1>819) →

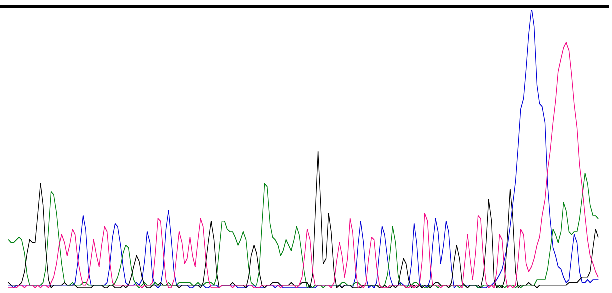

AAGGATTCTTCAGCTCTTTGAAAGAAAATGGTTCTCAGCTCCGTTGTGTCCAAcAG

2,890 2,900 2,910 2,920 2,930 2,940 2,950 2,960 2,970 2,980 2,990

ACAATTGAAACCATTGAAGAAAACATCGGTTGGATGGATAAGAATTTTGATAAAATCAGAGTGTGGCTGCAAAGTGAAAAGCTTGAACGCATGCATCATCACCATCA

U3823DH140-6.seq(1>2878) → ACAATTGAAACCATTGAAGAAAACATCGGTTGGATGGATAAGAATTTTGATAAAATCAGAGTGTGGCTGCAAAGTGAAAAGCTTGAACGCATGCATCATCACCATCA

E02-SEQ4.ab1(1>821) →

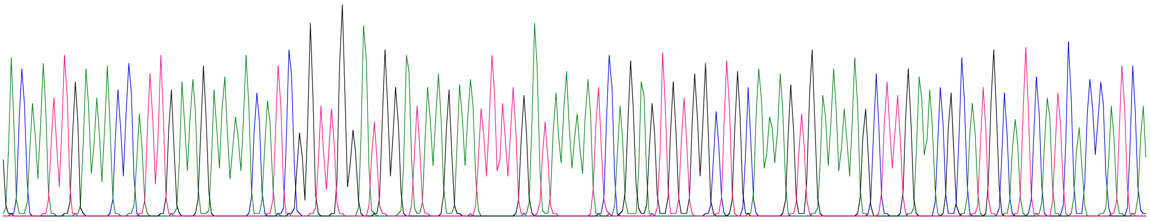

ACAATTGAAACCATTGAAGAAAACATCGGTTGGATGGATAAGAATTTTGATAAAATCAGAGTGTGGCTGCAAAGTGAAAAGCTTGAACGCATGCATCATCACCATCA

D10-PFastBac1-R.ab1(1>821) ←

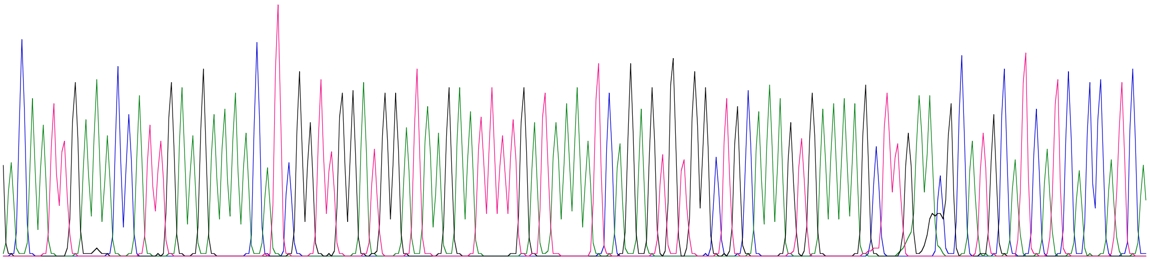

ACAATTGAAACCATTGAAGAAAACATCGGTTGGATGGATAAGAATTTTGATAAAATCAGAGTGTGGCTGCAAAGTGAAAAGCTTGAACGCATGCATCATCACCATCA

A09-SEQ5.ab1(1>819) →

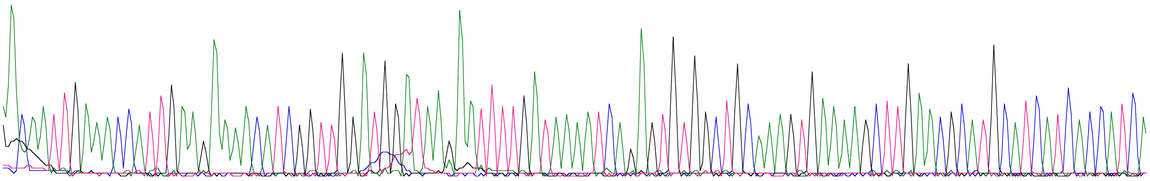

AcAATTGAAACCATTGAAGAAAACATCGGTTGGATGgaTaAgAATTTTGATAAAATCAGAGTGTGGCTGCAAAGTGAAAAGCTTGAACGCATGCATCATCACCATCA

3,000 3,010 3,020 3,030 3,040 3,050 3,060 3,070 3,080 3,090 3,100

CCATTAACTCGAGGCATGCGGTACCAAGCTTGTCGAGAAGTACTAGAGGATCATAATCAGCCATACCACATTTGTAGAGGTTTTACTTGCTTTAAAAAACCTCCCAC

U3823DH140-6.seq(1>2878) → CCATTAACTCGAG

E02-SEQ4.ab1(1>821) →

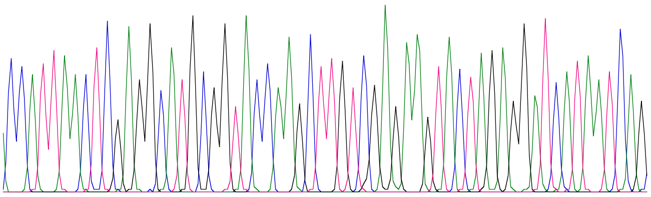

CCATTAACTCGAGGCATGCGGTACCAAGCTTGTCGAGAAGTACTAGAGGATCATAATCAGC

3,000 3,010 3,020 3,030 3,040 3,050 3,060 3,070 3,080 3,090 3,100

CCATTAACTCGAGGCATGCGGTACCAAGCTTGTCGAGAAGTACTAGAGGATCATAATCAGCCATACCACATTTGTAGAGGTTTTACTTGCTTTAAAAAACCTCCCAC

D10-PFastBac1-R.ab1(1>821)

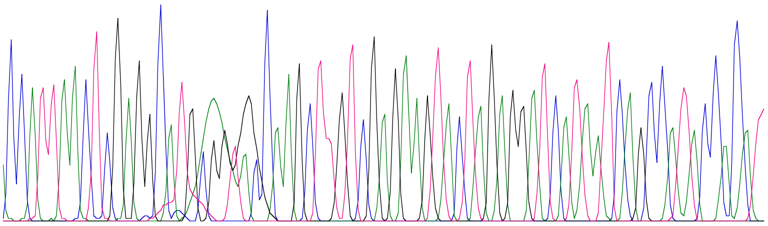

CCATTAACTCGAGGCATGCGGTACCAAGCTTGTCGAGAAGTACTAGAGGATCATAATCAGCCATACCACATT

A09-SEQ5.ab1(1>819)

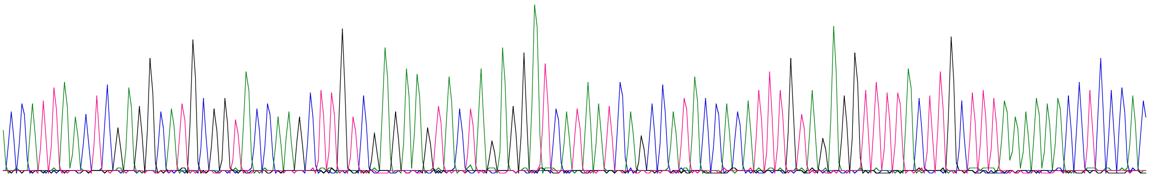

CCATTAACTCGAGGCATGCGGTACCAAGCTTGTCGAGAAGTACTAGAGGATCATAATCAGCCATACCACATTTGTAGAGGTTTTACTTGCTTTAAAAAACCTCCCAC

3,110 3,120 3,130 3,140 3,150 3,160 3,170 3,180 3,190 3,200 3,210

ACCTCCCCCTGAACCTGAAACATAAAATGAATGCAATTGTTGTTGTTAACTTGTTTATTGCAGCTTATAATGGTTACAAATAAAGCAATAGCATCACAAATTTCACA

A09-SEQ5.ab1(1>819)

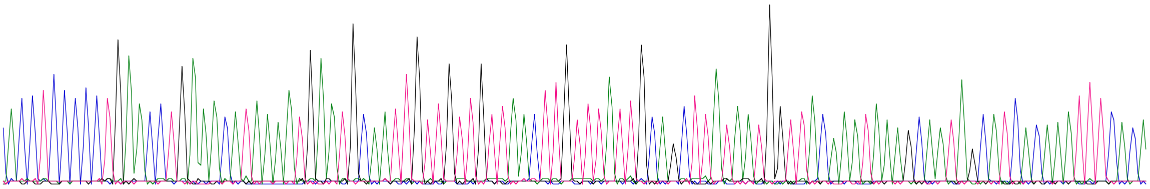

ACCTCCCCCTGAACCTGAAACATAAAATGAATGCAATTGTTGTTGTTAACTTGTTTATTGCAGCTTATAATGGTTACAAATAAAGCAATAGCATCACAAATTTCACA

3,220 3,230 3,240 3,250 3,260 3,270 3,280 3,290 3,300 3,310

AATAAAGCATTTTTTTCACTGCATTcTAGTTGTGGTTTGTCCAAACTCATCAATGTATCTTATCATGTCTGGATCTGATCACTGCTTGAGCCTAGGAGATCCGAACC

A09-SEQ5.ab1(1>819)

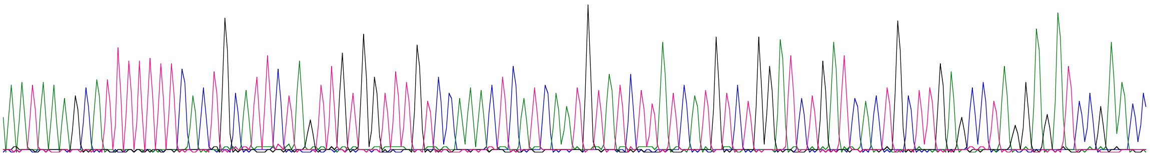

AATAAAGCATTTTTTTCACTGCATTcTAGTTGTGGTTTGTCCAAACTCATCAATGTATCTTATCATGTCTGGATCTGATCACTGCTTGAGCCTAGGAGATCCGAACC

3,320 3,330 3,340 3,350 3,360 3,370 3,380 3,390 3,400 3,410 3,420

AGATAAGTGAAATCTAGTTCCAAACTATTTTGTCATTTTTAATTTTCGTATTAGCTTACGACGCTACACCCAGTTCCCATCTATTTTGTCACTCTTCCCTAAATAAT

A09-SEQ5.ab1(1>819)

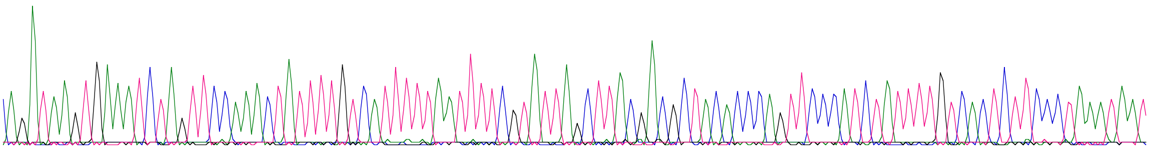

AGATAAGTGAAATCTAGTTCCAAACTATTTTGTCATTTTTAATTTTCGTATTAGCTTACGACGCTACACCCAGTTCCCATCTATTTTGTCACTCTTCCCTAAATAAT

3,430 3,440 3,450 3,460 3,470 3,480 3,490 3,500 3,510 3,520 3,530

CCTTAAAAACTCCATTTCCACCCCTCCCAGTTCCCAACTATTTTGTCCGCCCACAGCGGGGCATTTTTCTTCCTGTTATGTTTTTAATCAAACATCCTGCCAACTCC

A09-SEQ5.ab1(1>819)

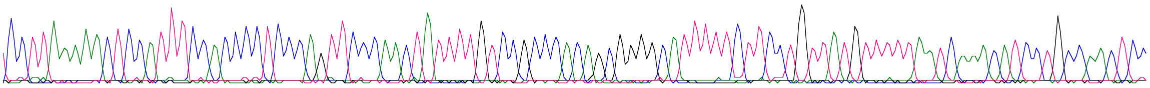

CCTTAAAAACTCCATTTCCACCCCTCCCAGTTCCCAACTATTTTGTCCGCCCACAGCGGGGCATTTTTCTTCCTGTTATGTTTTTAATCAAACATCCTGCCAACTCC

3,540 3,550 3,560 3,570 3,580 3,590 3,600 3,610 3,620 3,630

ATGTGACAAACCGTCATCTTCGGCTACTTTTTCTCTGTCACAGAATGAAAATTTTTCTGTCATCTCTTCGTTATTAATGTTTGTAATTGACTGAATATCAACGCTTA

A09-SEQ5.ab1(1>819)

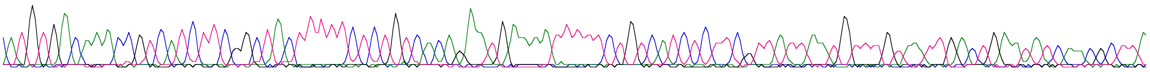

ATGTGACAAACCGTCATCTTCGGCTACTTTTTCTCTGTCACAGAATGAAAATTTTTCTGTCATCTCTTCGTTATTAATGTTTGTAATTGACTGAATATCAACGCTTA

3,640 3,650

TTTGCAGCCTGAAT

A09-SEQ5.ab1(1>819)

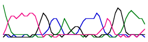

TTTGCAGCCTGAAT

Haplotype 8

10 20 30 40 50 60 70 80 90 100

GAAGACCTCGGCCGTCGCGGCGCTTGCCGGTGGTGCTGACCCCGGATGAAGTGGTTCGCATCCTCGGTTTTCTGGAAGGCGAGCATCGTTTGTTCGCCCAGGACTCT

B03-SEQ1R.ab1(1>812)

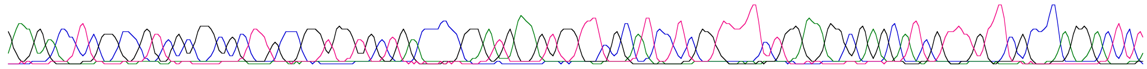

GAAGACCTCGGCCGTCGCGGCGCTTGCCGGTGGTGCTGACCCCGGATGAAGTGGTTCGCATCCTCGGTTTTCTGGAAGGCGAGCATCGTTTGTTCGCCCAGGACTCT

110 120 130 140 150 160 170 180 190 200 210

AGCTATAGTTCTAGTGGTTGGCTACGTATACTCCGGAATATTAATAGATCATGGAGATAATTAAAATGATAACCATCTCGCAAATAAATAAGTATTTTACTGTTTTC

B03-SEQ1R.ab1(1>812)

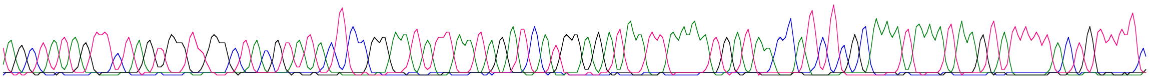

AGCTATAGTTCTAGTGGTTGGCTACGTATACTCCGGAATATTAATAGATCATGGAGATAATTAAAATGATAACCATCTCGCAAATAAATAAGTATTTTACTGTTTTC

220 230 240 250 260 270 280 290 300 310 320

GTAACAGTTTTGTAATAAAAAAACCTATAAATATTCCGGATTATTCATACCGTCCCACCATCGGGCGCGGATCCAACTCCTAAAAAACCGCCACCATGGTGTTTCTG

U3823DH140-4.seq(1>2878) GGATCCAACTCCTAAAAAACCGCCACCATGGTGTTTCTG

B03-SEQ1R.ab1(1>812)

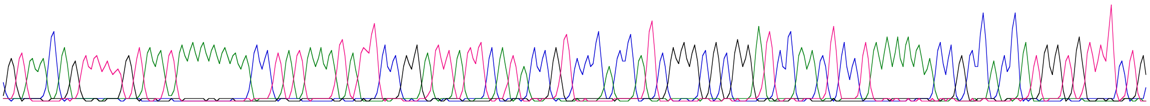

GTAACAGTTTTGTAATAAAAAAACCTATAAATATTCCGGATTATTCATACCGTCCCACCATCGGGCGCGGATCCAACTCCTAAAAAACCGCCACCATGGTGTTTCTG

C09-pFastbac-F.ab1(1>815)

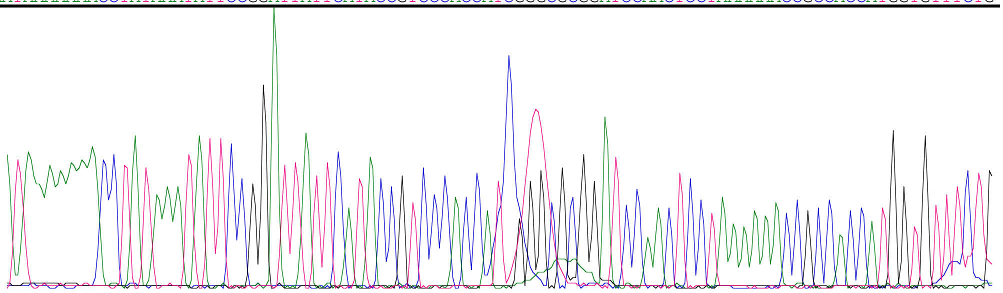

ATAAAAAAACCTATAAATATTCCGGATTATTCATACCGTCCCACCATCgGGCGcGGATCCAACTCCTAAAAAACCGCCACCATGGTGTTTcTg

330 340 350 360 370 380 390 400 410 420

CCCCTCAAATGGTCCCTTGCAACCATGTCATTTCTACTTTCCTCACTGTTGGCTCTCTTAACTGTGTCCACTCCTTCATGGTGTCAGAGCACTGAAGCATCTCCAAA

U3823DH140-4.seq(1>2878) CCCCTCAAATGGTCCCTTGCAACCATGTCATTTCTACTTTCCTCACTGTTGGCTCTCTTAACTGTGTCCACTCCTTCATGGTGTCAGAGCACTGAAGCATCTCCAAA

B03-SEQ1R.ab1(1>812)

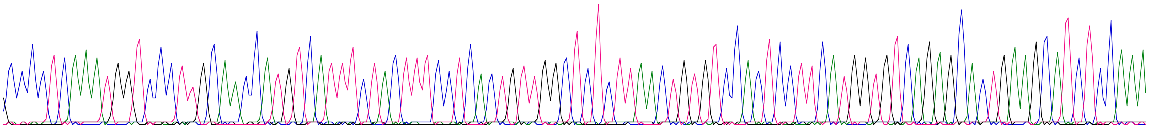

CCCCTCAAATGGTCCCTTGCAACCATGTCATTTCTACTTTCCTCACTGTTGGCTCTCTTAACTGTGTCCACTCCTTCATGGTGTCAGAGCACTGAAGCATCTCCAAA

330 340 350 360 370 380 390 400 410 420

CCCCTCAAATGGTCCCTTGCAACCATGTCATTTCTACTTTCCTCACTGTTGGCTCTCTTAACTGTGTCCACTCCTTCATGGTGTCAGAGCACTGAAGCATCTCCAAA

C09-pFastbac-F.ab1(1>815)

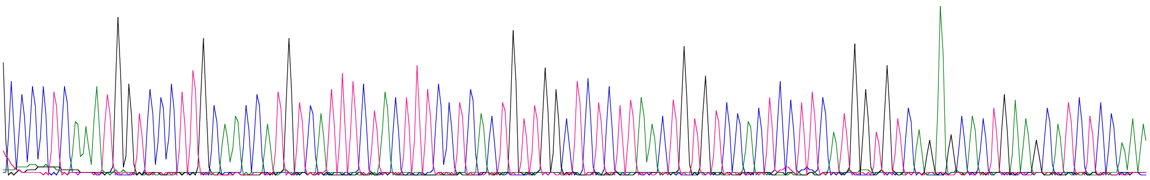

CCCCTCAAATGGTCCCTTGCAACCATGTCATTTCTACTTTCCTCACTGTTGGCTCTCTTAACTGTGTCCACTCCTTCATGGTGTCAGAGCACTGAAGCATCTCCAAA

430 440 450 460 470 480 490 500 510 520 530

ACGTAGTGATGGGACACCATTTCCTTGGAATAAAATACGACTTCCTGAGTACGTCATCCCAGTTCATTATGATCTCTTGATCCATGCAAACCTTACCACGCTGACCT

U3823DH140-4.seq(1>2878)
B03-SEQ1R.ab1(1>812)

ACGTAGTGATGGGACACCATTTCCTTGGAATAAAATACGACTTCCTGAGTACGTCATCCCAGTTCATTATGATCTCTTGATCCATGCAAACCTTACCACGCTGACCT

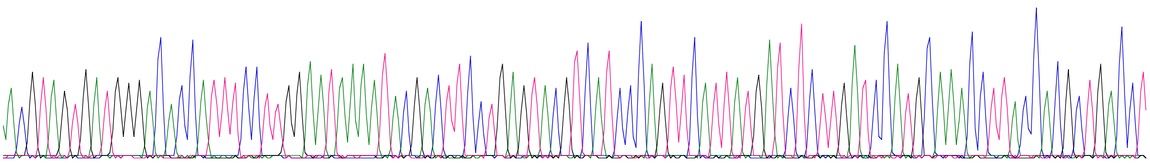

ACGTAGTGATGGGACACCATTTCCTTGGAATAAAATACGACTTCCTGAGTACGTCATCCCAGTTCATTATGATCTCTTGATCCATGCAAACCTTACCACGCTGACCT

C09-pFastbac-F.ab1(1>815)

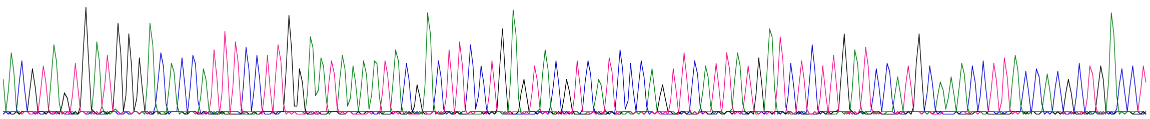

ACGTAGTGATGGGACACCATTTCCTTGGAATAAAATACGACTTCCTGAGTACGTCATCCCAGTTCATTATGATCTCTTGATCCATGCAAACCTTACCACGCTGACCT

540 550 560 570 580 590 600 610 620 630 640

TCTGGGGAACCACGAAAGTAGAAATCACAGCCAGTCAGCCCACCAGCACCATCATCCTGCATAGTCACCACCTGCAGATATCTAGGGCCACCCTCAGGAAGGGAGCT

U3823DH140-4.seq(1>2878)
B03-SEQ1R.ab1(1>812)

TCTGGGGAACCACGAAAGTAGAAATCACAGCCAGTCAGCCCACCAGCACCATCATCCTGCATAGTCACCACCTGCAGATATCTAGGGCCACCCTCAGGAAGGGAGCT

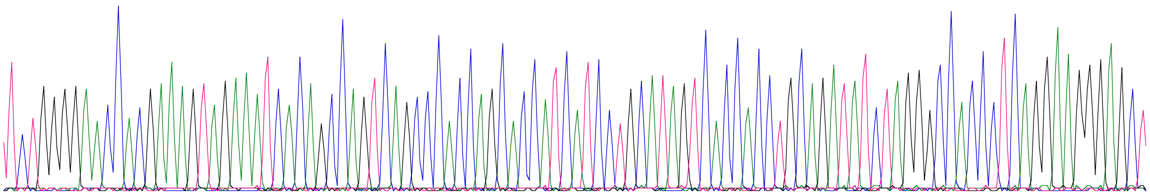

TCTGGGGAACCACGAAAGTAGAAATCACAGCCAGTCAGCCCACCAGCACCATCATCCTGCATAGTCACCACCTGCAGATATCTAGGGCCACCCTCAGGAAGGGAGCT

C09-pFastbac-F.ab1(1>815)

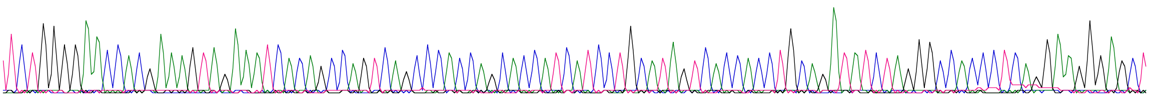

TCTGGGGAACCACGAAAGTAGAAATCACAGCCAGTCAGCCCACCAGCACCATCATCCTGCATAGTCACCACCTGCAGATATCTAGGGCCACCCTCAGGAAGGGAGCT

540 550 560 570 580 590 600 610 620 630 640

TCTGGGGAACCACGAAAGTAGAAATCACAGCCAGTCAGCCCACCAGCACCATCATCCTGCATAGTCACCACCTGCAGATATCTAGGGCCACCCTCAGGAAGGGAGCT

G05-SEQRN.ab1(1>811)

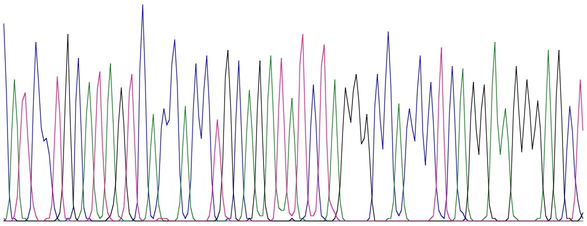

CATCCTGCATAGTCACCACCTGCAGATATCTAGGGCCACCCTCAGGAAGGGAGCT

C11-SEQ1.ab1(1>811)

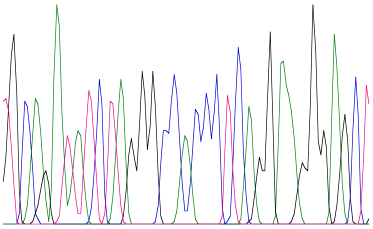

TGCAGATATCTAGGGCCACCCTCAGGAAGGGAGCT

650 660 670 680 690 700 710 720 730 740

GGAGAGAGGCTATCGGAAGAACCCCTGCAGGTCCTGGAACACCCCCCGCAGGAGCAAATTGCACTGCTGGCTCCCGAGCCCCTCCTTGTCGGGCTCCCGTACACAGT

U3823DH140-4.seq(1>2878)

GGAGAGAGGCTATCGGAAGAACCCCTGCAGGTCCTGGAACACCCCCCGCAGGAGCAAATTGCACTGCTGGCTCCCGAGCCCCTCCTTGTCGGGCTCCCGTACACAGT

B03-SEQ1R.ab1(1>812)

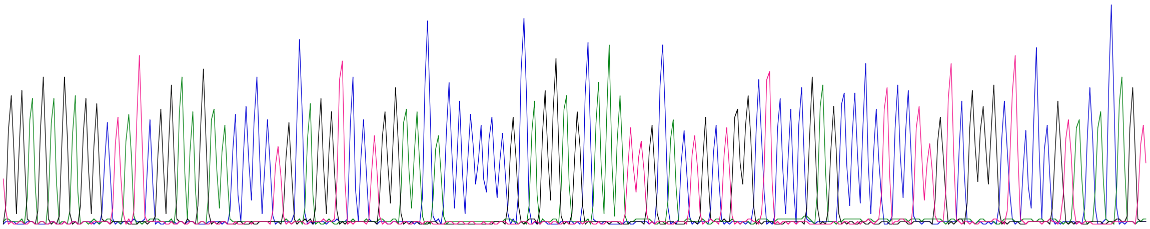

GGAGAGAGGCTATCGGAAGAACCCCTGCAGGTCCTGGAACACCCCCCGCAGGAGCAAATTGCACTGCTGGCTCCCGAGCCCCTCCTTGTCGGGCTCCCGTACACAGT

C09-pFastbac-F.ab1(1>815)

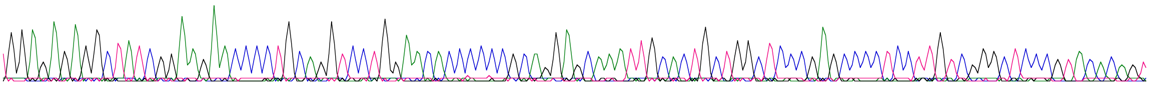

GGAGAGAGGCTATCGGAAGAACCCCTGCAGGTCCTGGAACACCCCCCGCAGGAGCAAATTGCACTGCTGGCTCCCGAGCCCCTCCTTGTCGGGCTCCCGTACACAGT

G05-SEQRN.ab1(1>811)

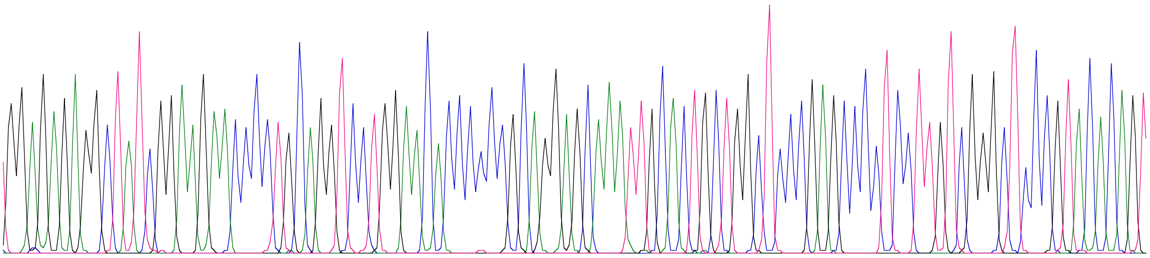

GGAGAGAGGCTATCGGAAGAACCCCTGCAGGTCCTGGAACACCCCCCGCAGGAGCAAATTGCACTGCTGGCTCCCGAGCCCCTCCTTGTCGGGCTCCCGTACACAGT

C11-SEQ1.ab1(1>811)

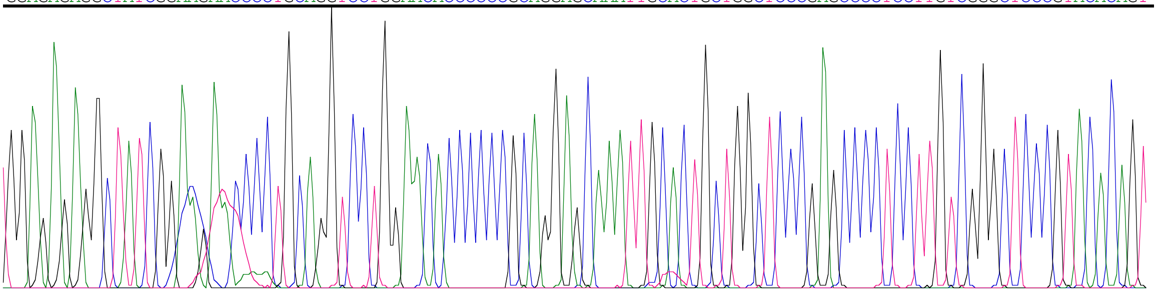

GGAGAGAGGCTATCGGAAGAACCCCTGCAGGTCCTGGAACACCCCCCGCAGGAGCAAATTGCACTGCTGGCTCCCGAGCCCCTCCTTGTCGGGCTCCCGTACACAGT

750 760 770 780 790 800 810 820 830 840 850

TGTCATTCACTATGCTGGCAATCTTTCGGAGACTTTCCACGGATTTTACAAAAGCACCTACAGAACCAAGGAAGGGGAACTGAGGATACTAGCATCAACACAATTTG

U3823DH140-4.seq(1>2878) TGTCATTCACTATGCTGGCAATCTTTCGGAGACTTTCCACGGATTTTACAAAAGCACCTACAGAACCAAGGAAGGGGAACTGAGGATACTAGCATCAACACAATTTG

B03-SEQ1R.ab1(1>812)

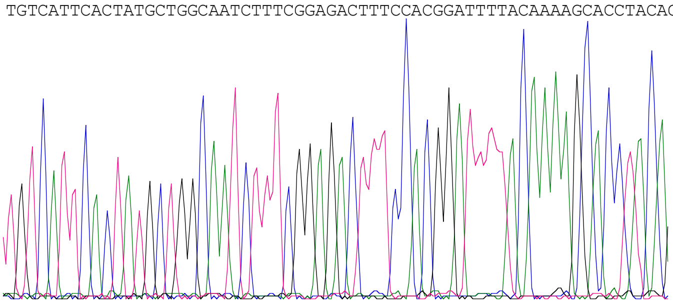

TGTCATTCACTATGCTGGCAATCTTTCGGAGACTTTCCACGGATTTTACAAAAGCACCTACAG

C09-pFastbac-F.ab1(1>815)

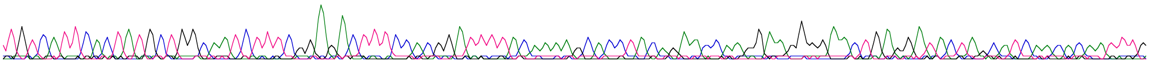

TGTCATTCACTATGCTGGCAATCTTTCGGAGACTTTCCACGGATTTTACAAAAGCACCTACAGAACCAAGGAAGGGGAACTGAGGATACTAGCATCAACACAATTTG

G05-SEQRN.ab1(1>811)

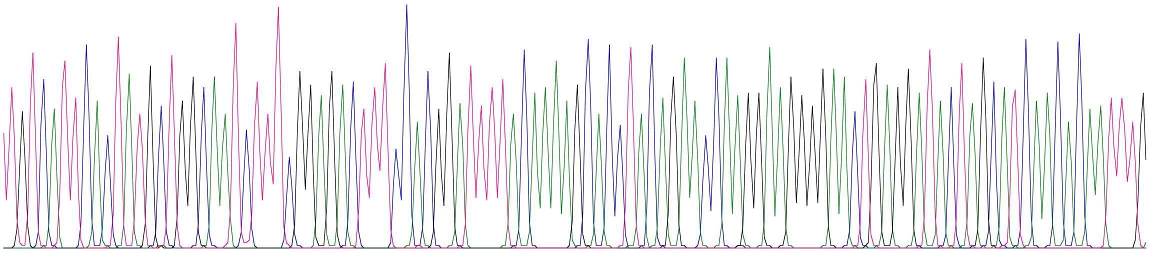

TGTCATTCACTATGCTGGCAATCTTTCGGAGACTTTCCACGGATTTTACAAAAGCACCTACAGAACCAAGGAAGGGGAACTGAGGATACTAGCATCAACACAATTTG

C11-SEQ1.ab1(1>811)

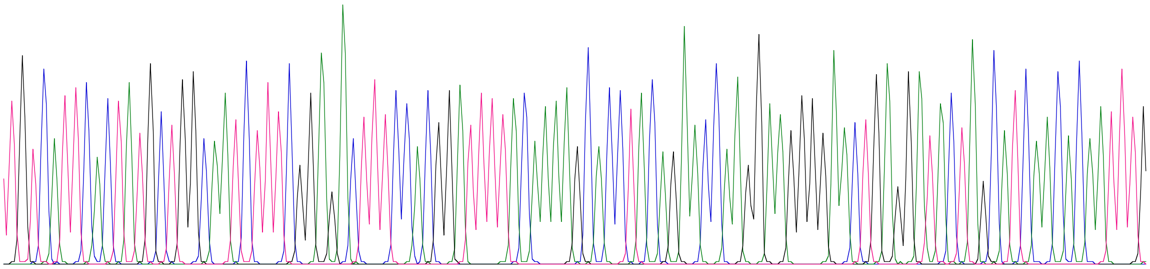

TGTCATTCACTATGCTGGCAATCTTTCGGAGACTTTCCACGGATTTTACAAAAGCACCTACAGAACCAAGGAAGGGGAACTGAGGATACTAGCATCAACACAATTTG

860 870 880 890 900 910 920 930 940 950 960

AACCCACTGCAGCTAGAATGGCCTTTCCCTGCTTTGATGAACCTGCCTTCAAAGCAAGTTTCTCAATCAAAATTAGAAGAGAGCCAAGGCACCTAGCCATCTCCAAT

U3823DH140-4.seq(1>2878) AACCCACTGCAGCTAGAATGGCCTTTCCCTGCTTTGATGAACCTGCCTTCAAAGCAAGTTTCTCAATCAAAATTAGAAGAGAGCCAAGGCACCTAGCCATCTCCAAT

C09-pFastbac-F.ab1(1>815)

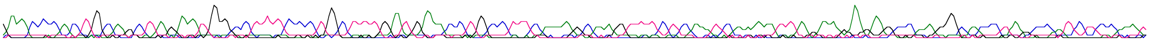

AACCCACTGCAGCTAGAATGGCCTTTCCCTGCTTTGATGAACCTGCCTTCAAAGCAAGTTTCTCAATCAAAATTAGAAGAGAGCCAAGGCACCTAGCCATCTCCAAT

G05-SEQRN.ab1(1>811)

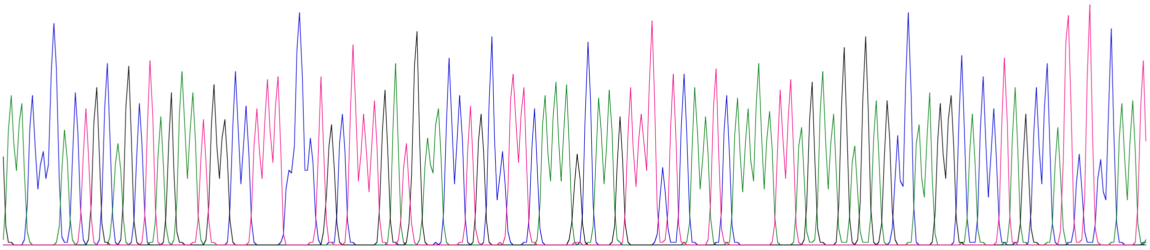

AACCCACTGCAGCTAGAATGGCCTTTCCCTGCTTTGATGAACCTGCCTTCAAAGCAAGTTTCTCAATCAAAATTAGAAGAGAGCCAAGGCACCTAGCCATCTCCAAT

860 870 880 890 900 910 920 930 940 950 960

AACCCACTGCAGCTAGAATGGCCTTTCCCTGCTTTGATGAACCTGCCTTCAAAGCAAGTTTCTCAATCAAAATTAGAAGAGAGCCAAGGCACCTAGCCATCTCCAAT

C11-SEQ1.ab1(1>811)

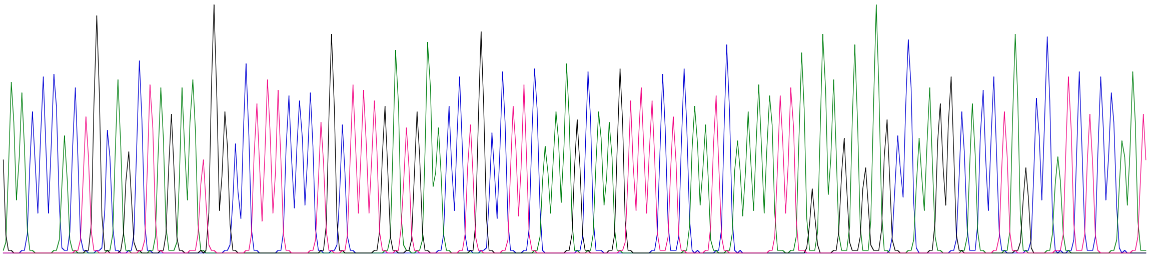

AACCCACTGCAGCTAGAATGGCCTTTCCCTGCTTTGATGAACCTGCCTTCAAAGCAAGTTTCTCAATCAAAATTAGAAGAGAGCCAAGGCACCTAGCCATCTCCAAT

970 980 990 1,000 1,010 1,020 1,030 1,040 1,050 1,060 1,070

ATGCCATTGGTGAAATCTGTGACTGTTGCTGAAGGACTCATAGAAGACCATTTTGATGTCACTGTGAAGATGAGCACCTATCTGGTGGCCTTCATCATTTCAGATTT

U3823DH140-4.seq(1>2878)
C09-pFastbac-F.ab1(1>815)

ATGCCATTGGTGAAATCTGTGACTGTTGCTGAAGGACTCATAGAAGACCATTTTGATGTCACTGTGAAGATGAGCACCTATCTGGTGGCCTTCATCATTTCAGATTT

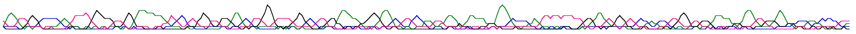

ATGCCATTGGTGAAATCTGTGACTGTTGCTGAAGGACTCATAGAAGACCATTTTGATGTCACTGTGAAGATGAGCACCTA

G05-SEQRN.ab1(1>811)

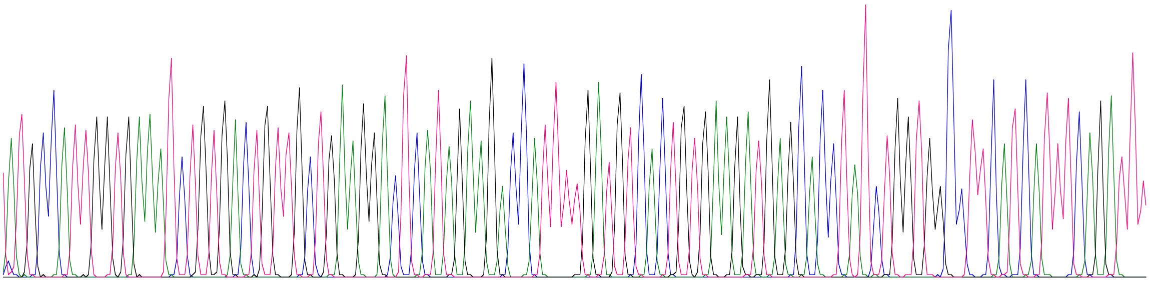

ATGCCATTGGTGAAATCTGTGACTGTTGCTGAAGGACTCATAGAAGACCATTTTGATGTCACTGTGAAGATGAGCACCTATCTGGTGGCCTTCATCATTTCAGATTT

C11-SEQ1.ab1(1>811)

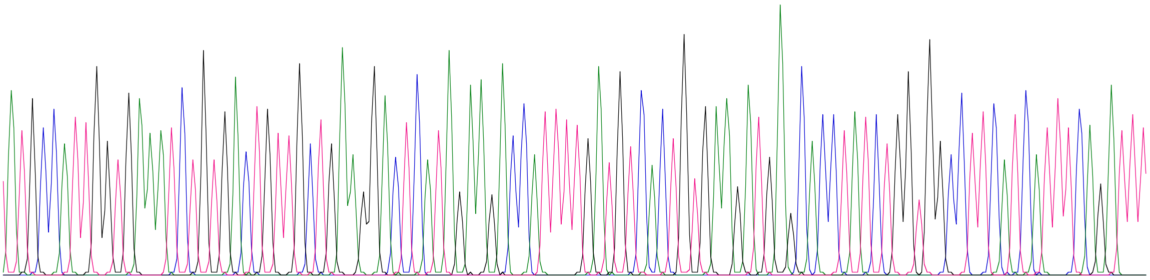

ATGCCATTGGTGAAATCTGTGACTGTTGCTGAAGGACTCATAGAAGACCATTTTGATGTCACTGTGAAGATGAGCACCTATCTGGTGGCCTTCATCATTTCAGATTT

1,080 1,090 1,100 1,110 1,120 1,130 1,140 1,150 1,160 1,170

TGAGTCTGTCAGCAAGATAACCAAGAGTGGAGTCAAGGTTTCTGTTTATGCTGTGCCAGACAAGATGAATCAAGCAGATTATGCACTGGATGCTGCGGTGACTCTTC

U3823DH140-4.seq(1>2878)
G05-SEQRN.ab1(1>811)

TGAGTCTGTCAGCAAGATAACCAAGAGTGGAGTCAAGGTTTCTGTTTATGCTGTGCCAGACAAGATGAATCAAGCAGATTATGCACTGGATGCTGCGGTGACTCTTC

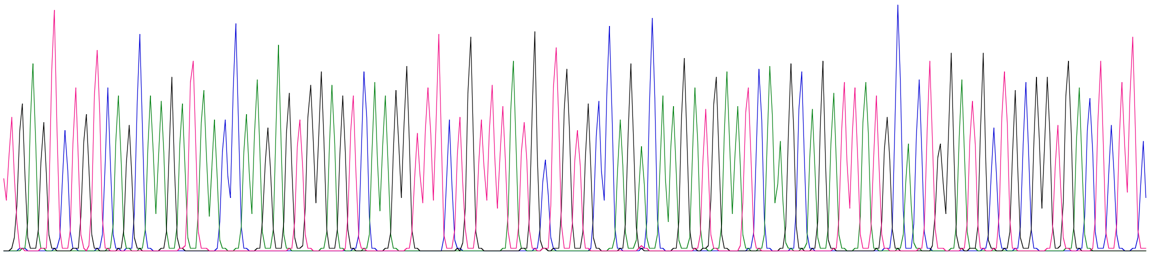

TGAGTCTGTCAGCAAGATAACCAAGAGTGGAGTCAAGGTTTCTGTTTATGCTGTGCCAGACAAGATGAATCAAGCAGATTATGCACTGGATGCTGCGGTGACTCTTC

I276M

1,080 1,090 1,100 1,110 1,120 1,130 1,140 1,150 1,160 1,170

TGAGTCTGTCAGCAAGATAACCAAGAGTGGAGTCAAGGTTTCTGTTTATGCTGTGCCAGACAAGATGAATCAAGCAGATTATGCACTGGATGCTGCGGTGACTCTTC

C11-SEQ1.ab1(1>811) →

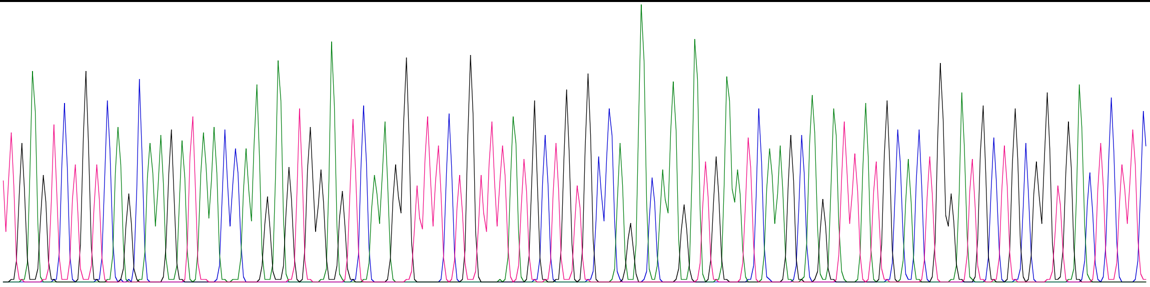

TGAGTCTGTCAGCAAGATAACCAAGAGTGGAGTCAAGGTTTCTGTTTATGCTGTGCCAGACAAGATGAATCAAGCAGATTATGCACTGGATGCTGCGGTGACTCTTC

1,180 1,190 1,200 1,210 1,220 1,230 1,240 1,250 1,260 1,270 1,280

TAGAATTTTATGAGGATTATTTCAGCATACCGTATCCCCTACCCAAACAAGATCTTGCTGCTATTCCCGACTTTCAGTCTGGTGCTATGGAAAACTGGGGACTGACA

U3823DH140-4.seq(1>2878) → TAGAATTTTATGAGGATTATTTCAGCATACCGTATCCCCTACCCAAACAAGATCTTGCTGCTATTCCCGACTTTCAGTCTGGTGCTATGGAAAACTGGGGACTGACA

G05-SEQRN.ab1(1>811) ←

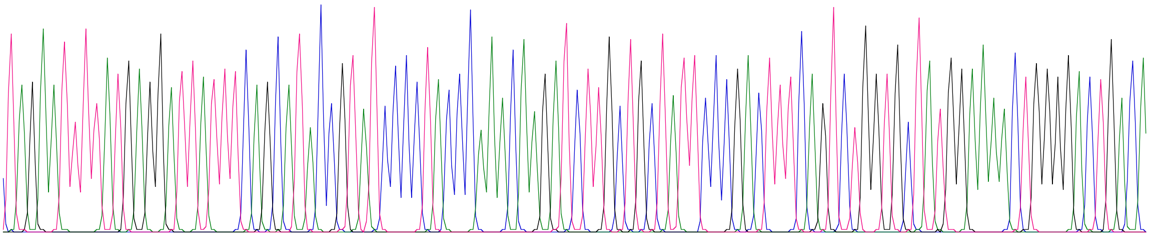

TAGAATTTTATGAGGATTATTTCAGCATACCGTATCCCCTACCCAAACAAGATCTTGCTGCTATTCCCGACTTTCAGTCTGGTGCTATGGAAAACTGGGGACTGACA

C11-SEQ1.ab1(1>811) →

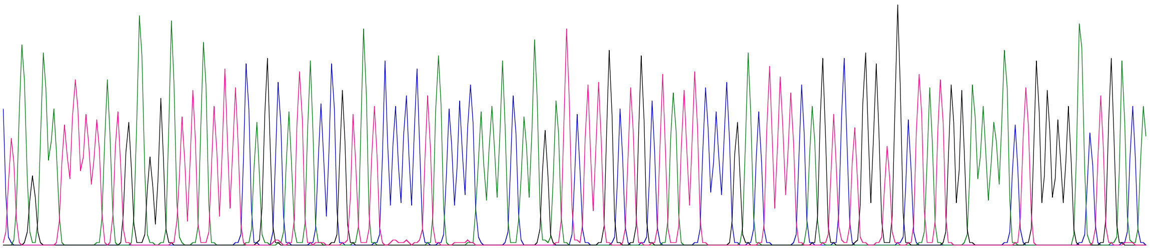

TAGAATTTTATGAGGATTATTTCAGCATACCGTATCCCCTACCCAAACAAGATCTTGCTGCTATTCCCGACTTTCAGTCTGGTGCTATGGAAAACTGGGGACTGACA

C03-SEQ3R.ab1(1>811) ←

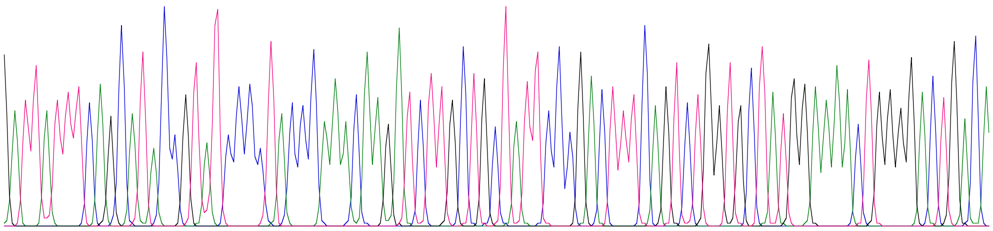

GATTATTTCAGCATACCGTATCCCCTACCCAAACAAGATCTTGCTGCTATTCCCGACTTTCAGTCTGGTGCTATGGAAAACTGGGGACTGACA

C12-SEQ2.ab1(1>811) →

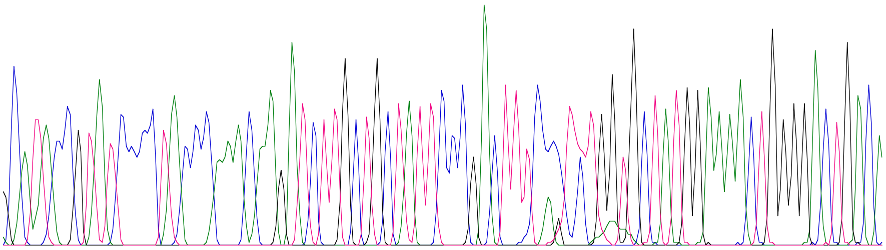

GCATACCGTATCCCCTACCCAAACAAGATCTTGCTGCTATTCCCGACTTTCAGTCTGGTGCTATGGAAAACTGGGGACTGACA

1,290 1,300 1,310 1,320 1,330 1,340 1,350 1,360 1,370 1,380 1,390

ACATATAGAGAATCTGCTCTGTTGTTTGATGCAGAAAAGTCTTCTGCATCAAGTAAGCTTGGCATCACAATGACTGTGGCCCATGAACTGGCTCACCAGTGGTTTGG

U3823DH140-4.seq(1>2878) ACATATAGAGAATCTGCTCTGTTGTTTGATGCAGAAAAGTCTTCTGCATCAAGTAAGCTTGGCATCACAATGACTGTGGCCCATGAACTGGCTCACCAGTGGTTTGG

G05-SEQRN.ab1(1>811)

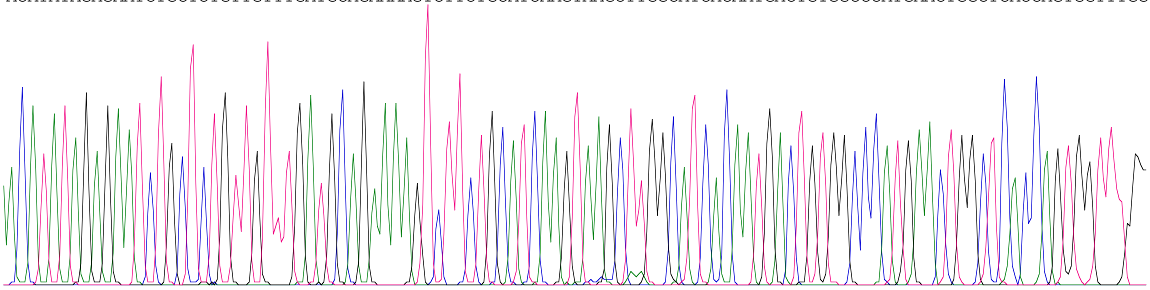

ACATATAGAGAATCTGCTCTGTTGTTTGATGCAGAAAAGTCTTCTGCATCAAGTAAGCTTGGCATCACAATGACTGTGGCCCATGAACTGGCTCACCAGTGGTTTGG

C11-SEQ1.ab1(1>811)

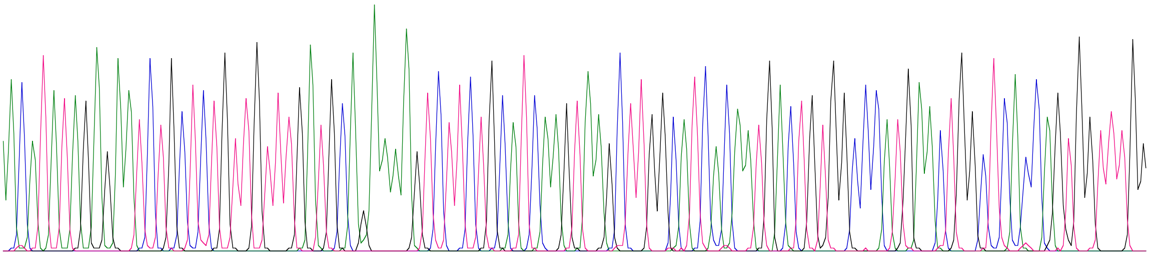

ACATATAGAGAATCTGCTCTGTTGTTTGATGCAGAAAAGTCTTCTGCATCAAGTAAGCTTGGCATCACAATGACTGTGGCCCATGAACTGGCTCACCAGTGGTTTGG

C03-SEQ3R.ab1(1>811)

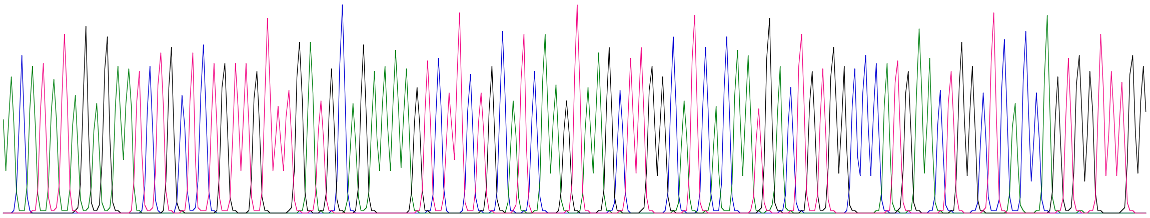

ACATATAGAGAATCTGCTCTGTTGTTTGATGCAGAAAAGTCTTCTGCATCAAGTAAGCTTGGCATCACAATGACTGTGGCCCATGAACTGGCTCACCAGTGGTTTGG

C12-SEQ2.ab1(1>811)

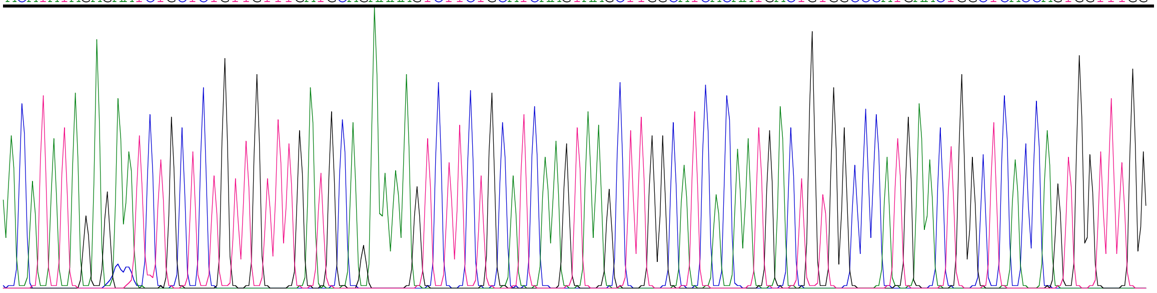

ACATATAGAGAATCTGCTCTGTTGTTTGATGCAGAAAAGTCTTCTGCATCAAGTAAGCTTGGCATCACAATGACTGTGGCCCATGAACTGGCTCACCAGTGGTTTGG

1,400 1,410 1,420 1,430 1,440 1,450 1,460 1,470 1,480 1,490

GAACCTGGTCACTATGGAATGGTGGAATGATCTTTGGCTAAATGAAGGATTTGCCAAATTTATGGAGTTTGTGTCTGTCAGTGTGACCCATCCTGAACTGAAAGTTG

U3823DH140-4.seq(1>2878) GAACCTGGTCACTATGGAATGGTGGAATGATCTTTGGCTAAATGAAGGATTTGCCAAATTTATGGAGTTTGTGTCTGTCAGTGTGACCCATCCTGAACTGAAAGTTG

G05-SEQRN.ab1(1>811)

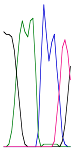

GAACCTG

C11-SEQ1.ab1(1>811)

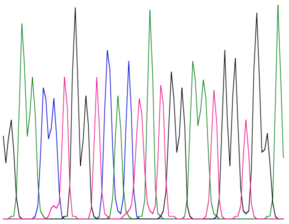

GAACCTGGTCACTATGGAATGGTGGAA

1,400 1,410 1,420 1,430 1,440 1,450 1,460 1,470 1,480 1,490

GAACCTGGTCACTATGGAATGGTGGAATGATCTTTGGCTAAATGAAGGATTTGCCAAATTTATGGAGTTTGTGTCTGTCAGTGTGACCCATCCTGAACTGAAAGTTG

C03-SEQ3R.ab1(1>811)

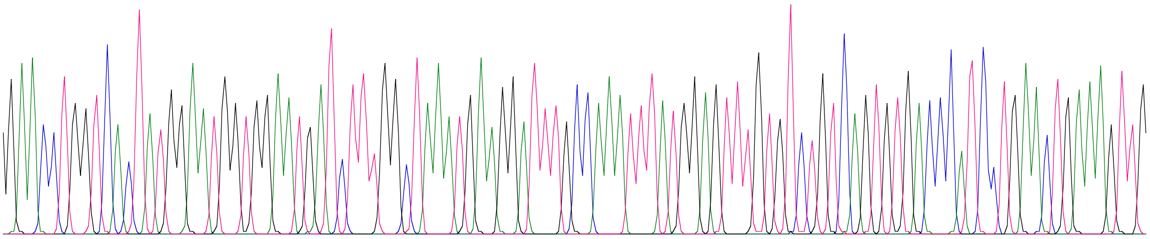

GAACCTGGTCACTATGGAATGGTGGAATGATCTTTGGCTAAATGAAGGATTTGCCAAATTTATGGAGTTTGTGTCTGTCAGTGTGACCCATCCTGAACTGAAAGTTG

C12-SEQ2.ab1(1>811)

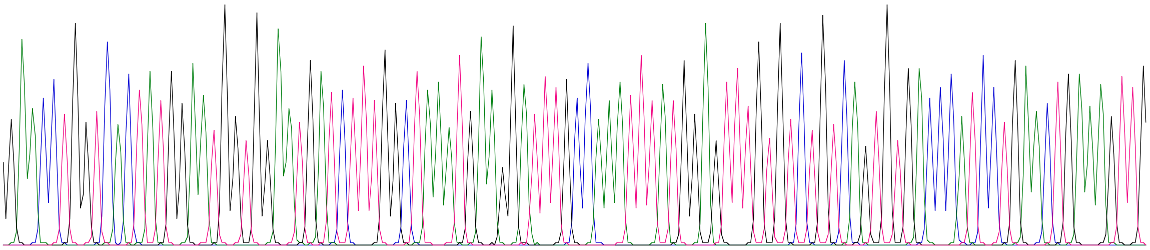

GAACCTGGTCACTATGGAATGGTGGAATGATCTTTGGCTAAATGAAGGATTTGCCAAATTTATGGAGTTTGTGTCTGTCAGTGTGACCCATCCTGAACTGAAAGTTG

1,500 1,510 1,520 1,530 1,540 1,550 1,560 1,570 1,580 1,590 1,600

GAGATTATTTCTTTGGCAAATGTTTTGACGCAATGGAGGTAGATGCTTTAAATTCCTCACACCCTGTGTCTACACCTGTGGAAAATCCTGCTCAGATCCGGGAGATG

U3823DH140-4.seq(1>2878) GAGATTATTTCTTTGGCAAATGTTTTGACGCAATGGAGGTAGATGCTTTAAATTCCTCACACCCTGTGTCTACACCTGTGGAAAATCCTGCTCAGATCCGGGAGATG
C03-SEQ3R.ab1(1>811)

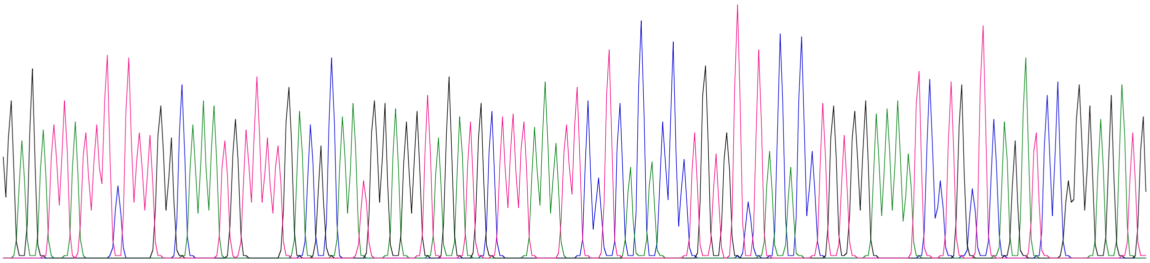

GAGATTATTTCTTTGGCAAATGTTTTGACGCAATGGAGGTAGATGCTTTAAATTCCTCACACCCTGTGTCTACACCTGTGGAAAATCCTGCTCAGATCCGGGAGATG

C12-SEQ2.ab1(1>811)

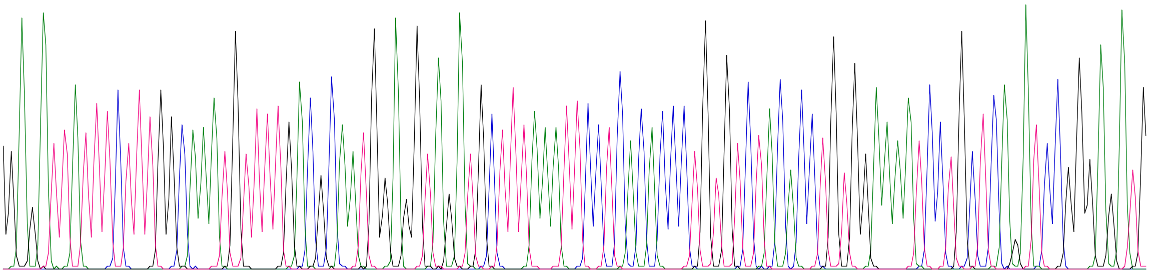

GAGATTATTTCTTTGGCAAATGTTTTGACGCAATGGAGGTAGATGCTTTAAATTCCTCACACCCTGTGTCTACACCTGTGGAAAATCCTGCTCAGATCCGGGAGATG

1,610 1,620 1,630 1,640 1,650 1,660 1,670 1,680 1,690 1,700 1,710

TTTGATGATGTTTCTTATGATAAGGGAGCTTGTATTCTGAATATGCTAAGGGAGTATCTTAGTGCTGACGCATTTAAAAGTGGTATTGTACAGTATCTCCAGAAGCA

U3823DH140-4.seq(1>2878) TTTGATGATGTTTCTTATGATAAGGGAGCTTGTATTCTGAATATGCTAAGGGAGTATCTTAGTGCTGACGCATTTAAAAGTGGTATTGTACAGTATCTCCAGAAGCA
C03-SEQ3R.ab1(1>811)

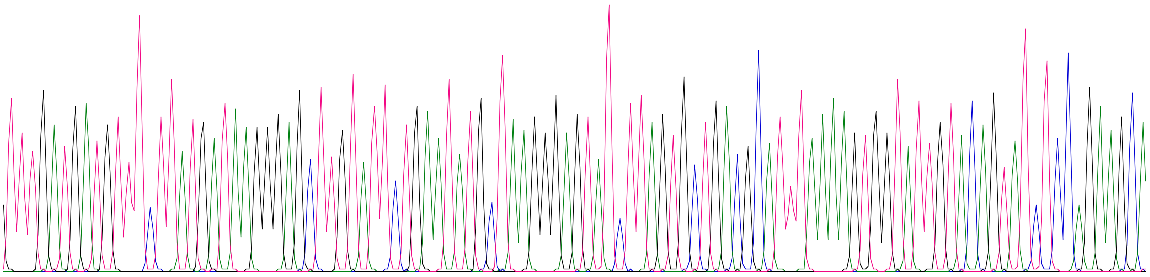

TTTGATGATGTTTCTTATGATAAGGGAGCTTGTATTCTGAATATGCTAAGGGAGTATCTTAGTGCTGACGCATTTAAAAGTGGTATTGTACAGTATCTCCAGAAGCA

1,610 1,620 1,630 1,640 1,650 1,660 1,670 1,680 1,690 1,700 1,710

TTTGATGATGTTTCTTATGATAAGGGAGCTTGTATTCTGAATATGCTAAGGGAGTATCTTAGTGCTGACGCATTTAAAAGTGGTATTGTACAGTATCTCCAGAAGCA

C12-SEQ2.ab1(1>811)

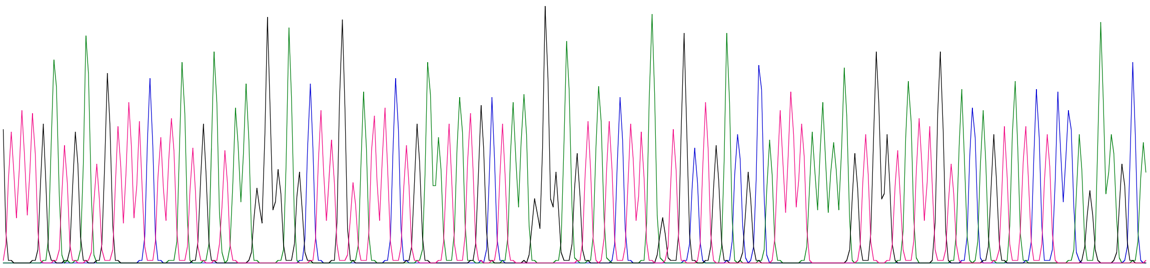

TTTGATGATGTTTCTTATGATAAGGGAGCTTGTATTCTGAATATGCTAAGGGAGTATCTTAGTGCTGACGCATTTAAAAGTGGTATTGTACAGTATCTCCAGAAGCA

1,720 1,730 1,740 1,750 1,760 1,770 1,780 1,790 1,800 1,810

TAGCTATAAAAATACAAAAAACGAGGACCTGTGGGATAGTATGGCAAGTATTTGCCCTACAGATGGTGTAAAAGGGATGGATGGCTTTTGCTCTAGAAGTCAACATT

U3823DH140-4.seq(1>2878)

TAGCTATAAAAATACAAAAAACGAGGACCTGTGGGATAGTATGGCAAGTATTTGCCCTACAGATGGTGTAAAAGGGATGGATGGCTTTTGCTCTAGAAGTCAACATT

C03-SEQ3R.ab1(1>811)

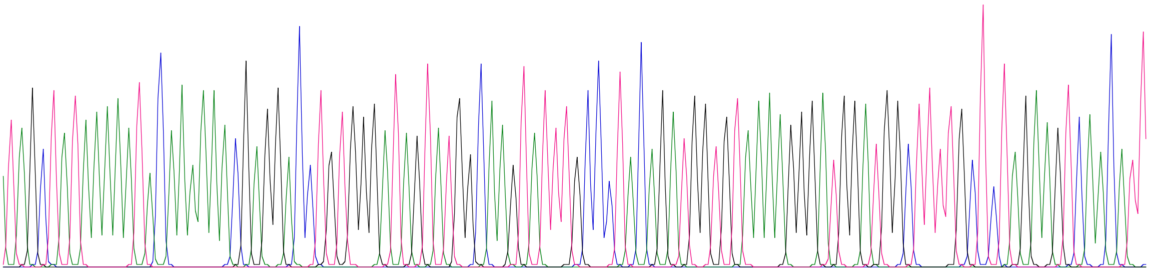

TAGCTATAAAAATACAAAAAACGAGGACCTGTGGGATAGTATGGCAAGTATTTGCCCTACAGATGGTGTAAAAGGGATGGATGGCTTTTGCTCTAGAAGTCAACATT

C12-SEQ2.ab1(1>811)

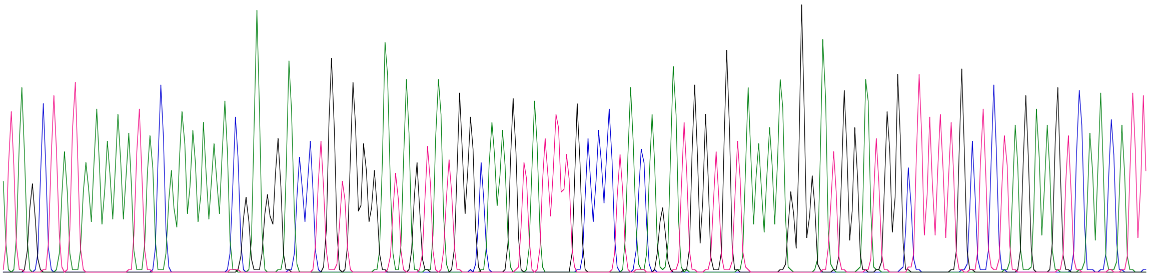

TAGCTATAAAAATACAAAAAACGAGGACCTGTGGGATAGTATGGCAAGTATTTGCCCTACAGATGGTGTAAAAGGGATGGATGGCTTTTGCTCTAGAAGTCAACATT

G04-SEQ4RN.ab1(1>811)

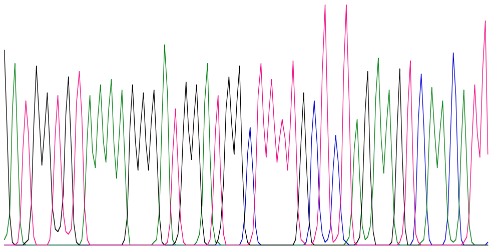

GATGGTGTAAAAGGGATGGATGGCTTTTGCTCTAGAAGTCAACATT

D01-EQ3.ab1(1>819)

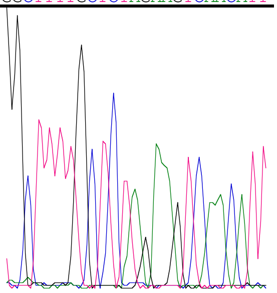

GGCTTTTGCTCTAGAAGTCAACATT

K528R

1,820 1,830 1,840 1,850 1,860 1,870 1,880 1,890 1,900 1,910 1,920

CATCTTCATCCTCACATTGGCATCAGGAAGGGGTGGATGTGAAAACCATGATGAACACTTGGACACTGCAGCGTGGTTTTCCCCTAATAACCATCACAGTGAGGGGG

U3823DH140-4.seq(1>2878) CATCTTCATCCTCACATTGGCATCAGGAAGGGGTGGATGTGAAAACCATGATGAACACTTGGACACTGCAGCGTGGTTTTCCCCTAATAACCATCACAGTGAGGGGG

C03-SEQ3R.ab1(1>811)

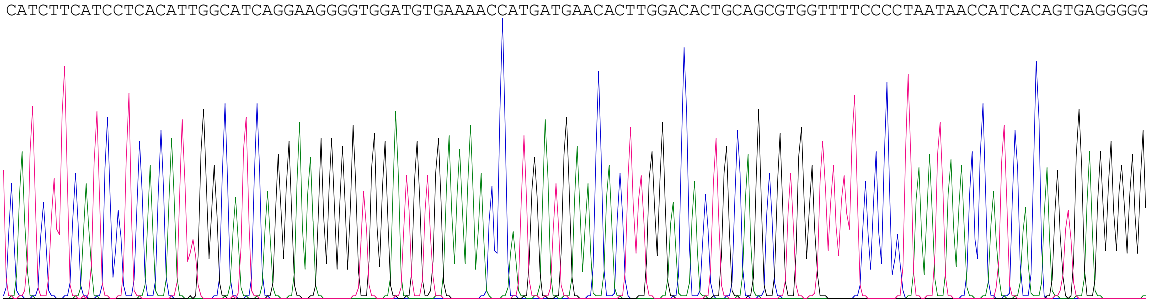

CATCTTCATCCTCACATTGGCATCAGGAAGGGGTGGATGTGAAAACCATGATGAACACTTGGACACTGCAGCGTGGTTTTCCCCTAATAACCATCACAGTGAGGGGG

C12-SEQ2.ab1(1>811)

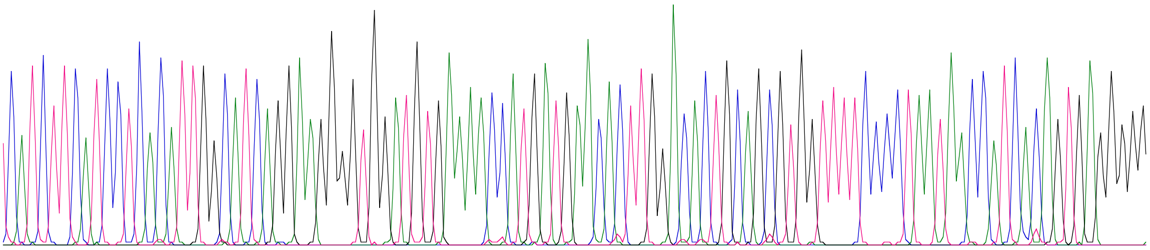

CATCTTCATCCTCACATTGGCATCAGGAAGGGGTGGATGTGAAAACCATGATGAACACTTGGACACTGCAGCGTGGTTTTCCCCTAATAACCATCACAGTGAGGGGG

G04-SEQ4RN.ab1(1>811)

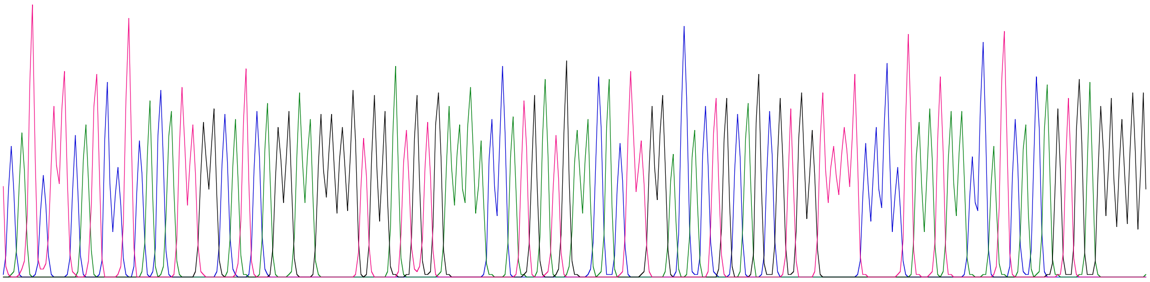

CATCTTCATCCTCACATTGGCATCAGGAAGGGGTGGATGTGAAAACCATGATGAACACTTGGACACTGCAGCGTGGTTTTCCCCTAATAACCATCACAGTGAGGGGG

D01-EQ3.ab1(1>819)

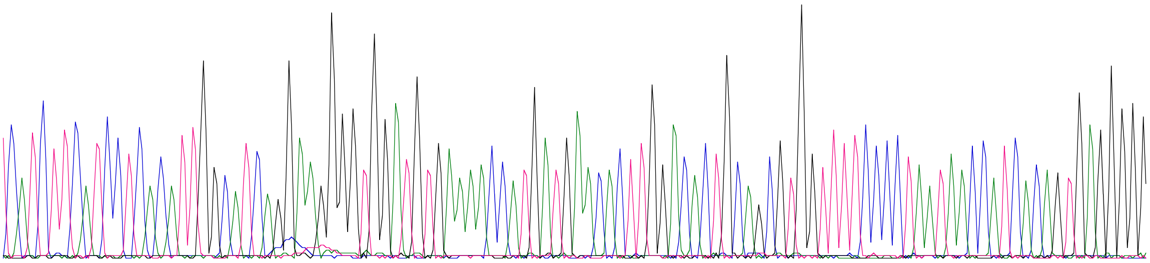

CATCTTCATCCTCACATTGGCATCAGGAAGGGGTGGATGTGAAAACCATGATGAACACTTGGACACTGCAGCGTGGTTTTCCCCTAATAACCATCACAGTGAGGGGG

1,930 1,940 1,950 1,960 1,970 1,980 1,990 2,000 2,010 2,020 2,030

AGGAATGTACACATGAAGCAAGAGCACTACATGAAGGGCTCTGACGGCGCCCCGGACACTGGGTACCTGTGGCATGTTCCATTGACATTCATCACCAGCAAATCCGA

U3823DH140-4.seq(1>2878) AGGAATGTACACATGAAGCAAGAGCACTACATGAAGGGCTCTGACGGCGCCCCGGACACTGGGTACCTGTGGCATGTTCCATTGACATTCATCACCAGCAAATCCGA

C03-SEQ3R.ab1(1>811)

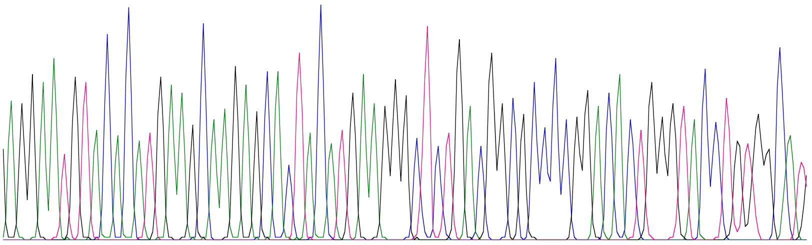

AGGAATGTACACATGAAGCAAGAGCACTACATGAAGGGCTCTGACGGCGCCCCGGACACTGGGTACCTGTGGCATG

C12-SEQ2.ab1(1>811)

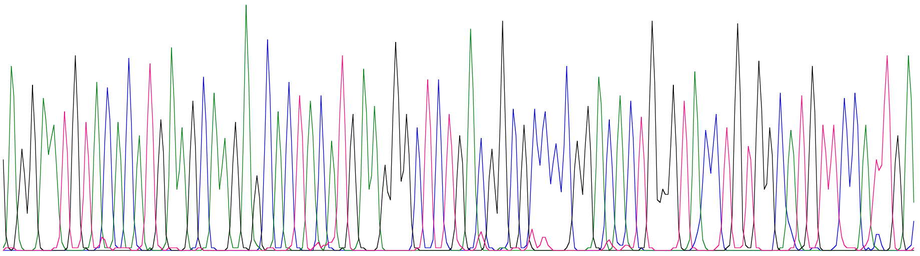

AGGAATGTACACATGAAGCAAGAGCACTACATGAAGGGCTCTGACGGCGCCCCGGACACTGGGTACCTGTGGCATGTTCCATTGAC

1,930 1,940 1,950 1,960 1,970 1,980 1,990 2,000 2,010 2,020 2,030

AGGAATGTACACATGAAGCAAGAGCACTACATGAAGGGCTCTGACGGCGCCCCGGACACTGGGTACCTGTGGCATGTTCCATTGACATTCATCACCAGCAAATCCGA

G04-SEQ4RN.ab1(1>811)

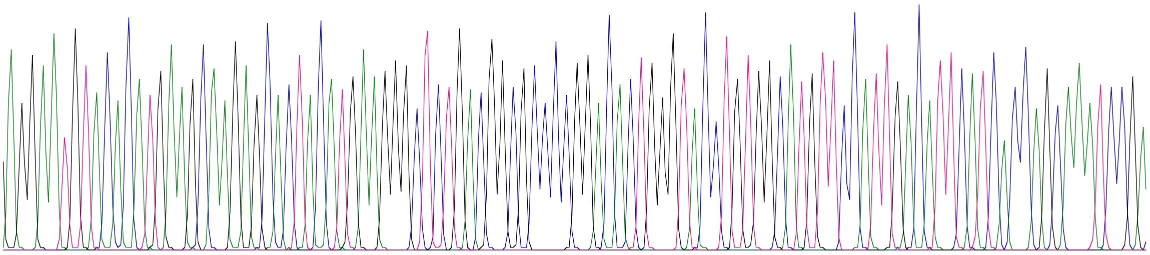

AGGAATGTACACATGAAGCAAGAGCACTACATGAAGGGCTCTGACGGCGCCCCGGACACTGGGTACCTGTGGCATGTTCCATTGACATTCATCACCAGCAAATCCGA

D01-EQ3.ab1(1>819)

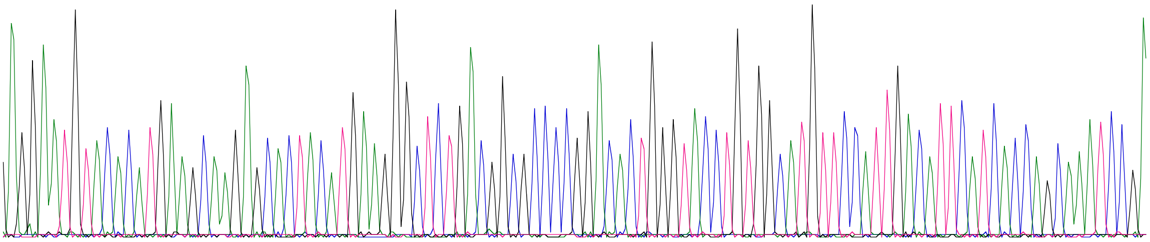

AGGAATGTACACATGAAGCAAGAGCACTACATGAAGGGCTCTGACGGCGCCCCGGACACTGGGTACCTGTGGCATGTTCCATTGACATTCATCACCAGCAAATCCGA

2,040 2,050 2,060 2,070 2,080 2,090 2,100 2,110 2,120 2,130 2,140

CATGGTCCATCGATTTTTGCTAAAAACAAAAACAGATGTGCTCATCCTCCCAGAAGAGGTGGAATGGATCAAATTTAATGTGGGCATGAATGGCTATTACATTGTGC

U3823DH140-4.seq(1>2878)
G04-SEQ4RN.ab1(1>811)

CATGGTCCATCGATTTTTGCTAAAAACAAAAACAGATGTGCTCATCCTCCCAGAAGAGGTGGAATGGATCAAATTTAATGTGGGCATGAATGGCTATTACATTGTGC

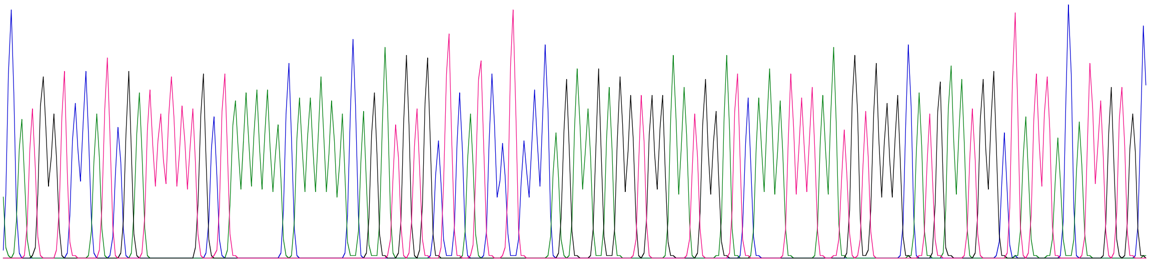

CATGGTCCATCGATTTTTGCTAAAAACAAAAACAGATGTGCTCATCCTCCCAGAAGAGGTGGAATGGATCAAATTTAATGTGGGCATGAATGGCTATTACATTGTGC

D01-EQ3.ab1(1>819)

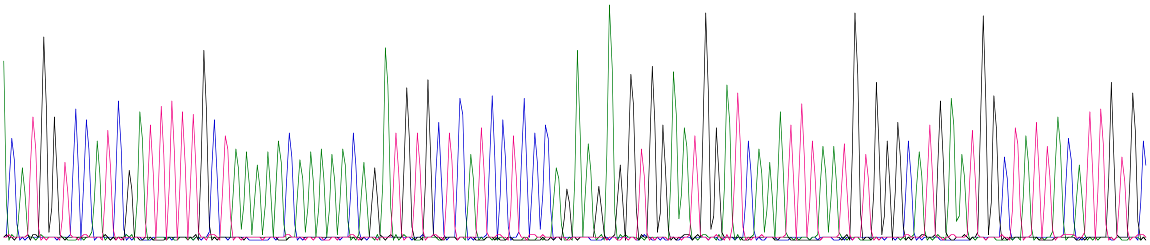

CATGGTCCATCGATTTTTGCTAAAAACAAAAACAGATGTGCTCATCCTCCCAGAAGAGGTGGAATGGATCAAATTTAATGTGGGCATGAATGGCTATTACATTGTGC

2,150 2,160 2,170 2,180 2,190 2,200 2,210 2,220 2,230 2,240

ATTACGAGGATGATGGATGGGACTCTTTGACTGGCCTTTTAAAAGGAACACACACAGCAGTCAGCAGTAATGATCGGGCGAGTCTCATTAACAATGCATTTCAGCTC

U3823DH140-4.seq(1>2878)
G04-SEQ4RN.ab1(1>811)

ATTACGAGGATGATGGATGGGACTCTTTGACTGGCCTTTTAAAAGGAACACACACAGCAGTCAGCAGTAATGATCGGGCGAGTCTCATTAACAATGCATTTCAGCTC

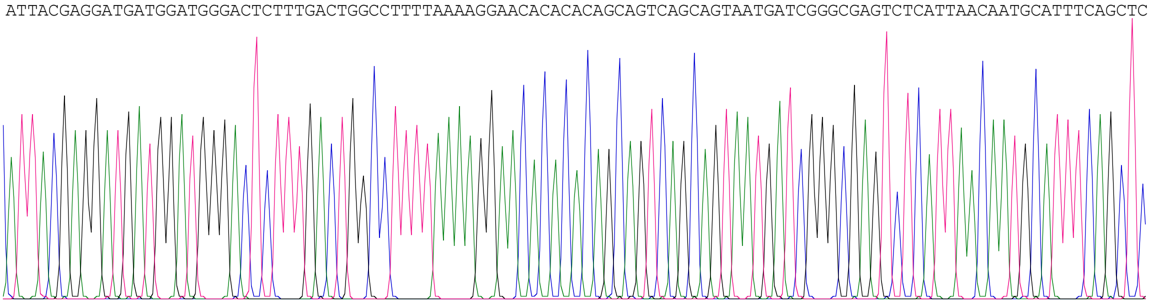

ATTACGAGGATGATGGATGGGACTCTTTGACTGGCCTTTTAAAAGGAACACACACAGCAGTCAGCAGTAATGATCGGGCGAGTCTCATTAACAATGCATTTCAGCTC

2,150 2,160 2,170 2,180 2,190 2,200 2,210 2,220 2,230 2,240

ATTACGAGGATGATGGATGGGACTCTTTGACTGGCCTTTTAAAAGGAACACACACAGCAGTCAGCAGTAATGATCGGGCGAGTCTCATTAACAATGCATTTCAGCTC

D01-EQ3.ab1(1>819)

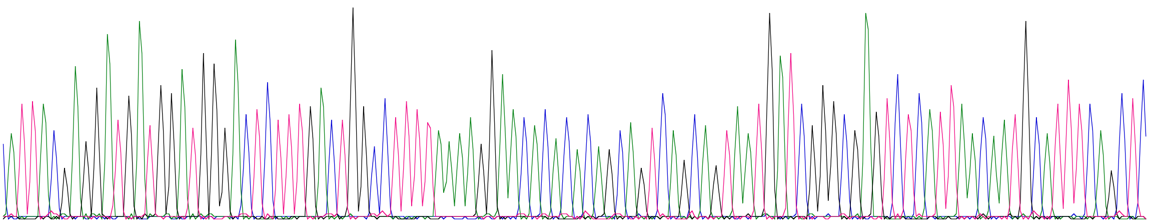

ATTACGAGGATGATGGATGGGACTCTTTGACTGGCCTTTTAAAAGGAACACACACAGCAGTCAGCAGTAATGATCGGGCGAGTCTCATTAACAATGCATTTCAGCTC

2,250 2,260 2,270 2,280 2,290 2,300 2,310 2,320 2,330 2,340 2,350

GTCAGCATTGGGAAGCTGTCCATTGAAAAGGCCTTGGATTTATCCCTGTACTTGAAACATGAAACTGAAATTATGCCCGTGTTTCAAGGTTTGAATGAGCTGATTCC

U3823DH140-4.seq(1>2878)
G04-SEQ4RN.ab1(1>811)

GTCAGCATTGGGAAGCTGTCCATTGAAAAGGCCTTGGATTTATCCCTGTACTTGAAACATGAAACTGAAATTATGCCCGTGTTTCAAGGTTTGAATGAGCTGATTCC

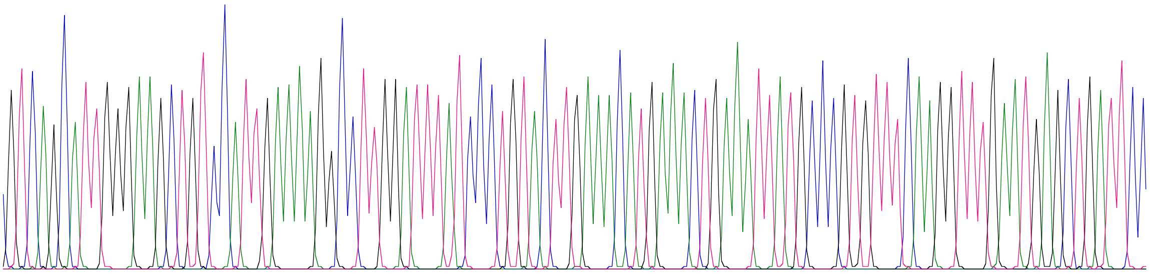

GTCAGCATTGGGAAGCTGTCCATTGAAAAGGCCTTGGATTTATCCCTGTACTTGAAACATGAAACTGAAATTATGCCCGTGTTTCAAGGTTTGAATGAGCTGATTCC

D01-EQ3.ab1(1>819)

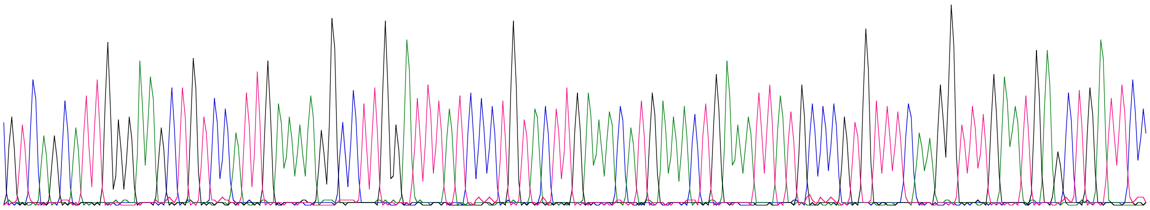

GTCAGCATTGGGAAGCTGTCCATTGAAAAGGCCTTGGATTTATCCCTGTACTTGAAACATGAAACTGAAATTATGCCCGTGTTTCAAGGTTTGAATGAGCTGATTCC

2,360 2,370 2,380 2,390 2,400 2,410 2,420 2,430 2,440 2,450 2,460

TATGTATAAGTTAATGGAGAAAAGAGATATGAATGAAGTGGAAACTCAATTCAAGGCCTTCCTCATCAGGCTGCTAAGGGACCTCATTGATAAGCAGACATGGACAG

U3823DH140-4.seq(1>2878)
G04-SEQ4RN.ab1(1>811)

TATGTATAAGTTAATGGAGAAAAGAGATATGAATGAAGTGGAAACTCAATTCAAGGCCTTCCTCATCAGGCTGCTAAGGGACCTCATTGATAAGCAGACATGGACAG

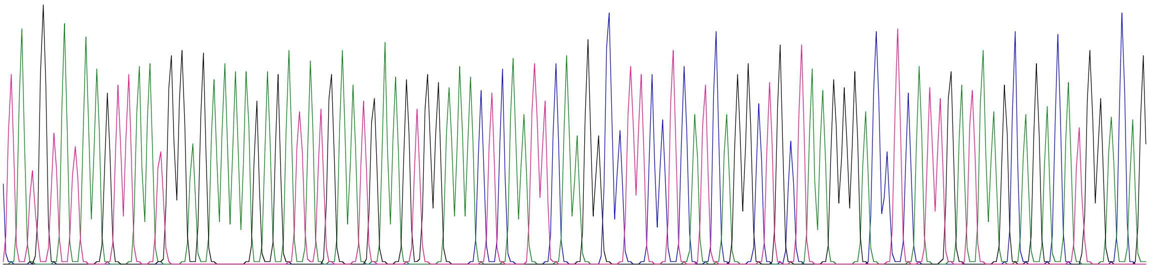

TATGTATAAGTTAATGGAGAAAAGAGATATGAATGAAGTGGAAACTCAATTCAAGGCCTTCCTCATCAGGCTGCTAAGGGACCTCATTGATAAGCAGACATGGACAG

D01-EQ3.ab1(1>819)

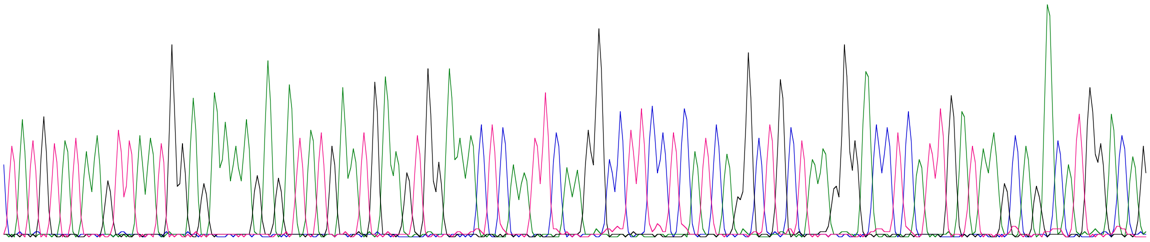

TATGTATAAGTTAATGGAGAAAAGAGATATGAATGAAGTGGAAACTCAATTCAAGGCCTTCCTCATCAGGCTGCTAAGGGACCTCATTGATAAGCAGACATGGACAG

2,360 2,370 2,380 2,390 2,400 2,410 2,420 2,430 2,440 2,450 2,460

TATGTATAAGTTAATGGAGAAAAGAGATATGAATGAAGTGGAAACTCAATTCAAGGCCTTCCTCATCAGGCTGCTAAGGGACCTCATTGATAAGCAGACATGGACAG

D02-SEQ4.ab1(1>819)

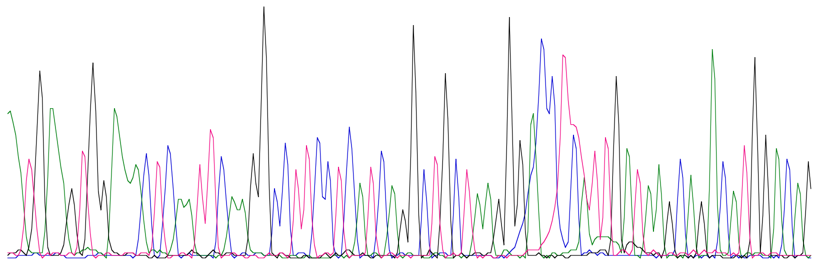

AATGAAGTGGAaACTCAATTCAAGGCCTTCCTCATCAGGCTGCTAAGGGaCCTCATTGATAAGCAGACATGGACAG

C10-PFastBac1-R.ab1(1>811)

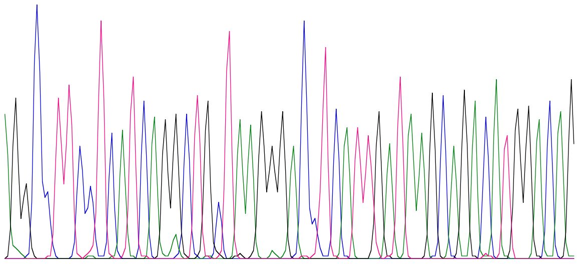

AGGCCTTCCTCATCAGGCTGCTAAGGGACCTCATTGATAAGCAGACATGGACAG

Q730E

2,470 2,480 2,490 2,500 2,510 2,520 2,530 2,540 2,550 2,560

ACGAGGGCTCAGTCTCAGAGCGAATGCTGCGGAGTGAGCTACTACTCCTCGCCTGTGTGCACAACTATCAGCCGTGCGTACAGAGGGCAGAAGGCTATTTCAGAAAG

U3823DH140-4.seq(1>2878) ACGAGGGCTCAGTCTCAGAGCGAATGCTGCGGAGTGAGCTACTACTCCTCGCCTGTGTGCACAACTATCAGCCGTGCGTACAGAGGGCAGAAGGCTATTTCAGAAAG

G04-SEQ4RN.ab1(1>811)

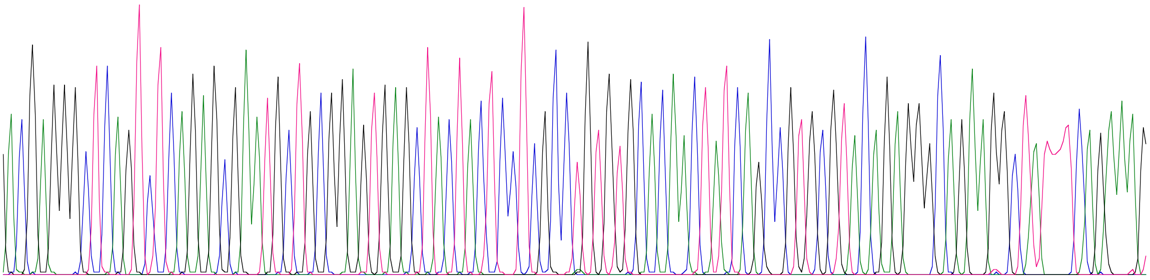

ACGAGGGCTCAGTCTCAGAGCGAATGCTGCGGAGTGAGCTACTACTCCTCGCCTGTGTGCACAACTATCAGCCGTGCGTACAGAGGGCAGAAGGCTATTTCAGAAAG

D01-EQ3.ab1(1>819)

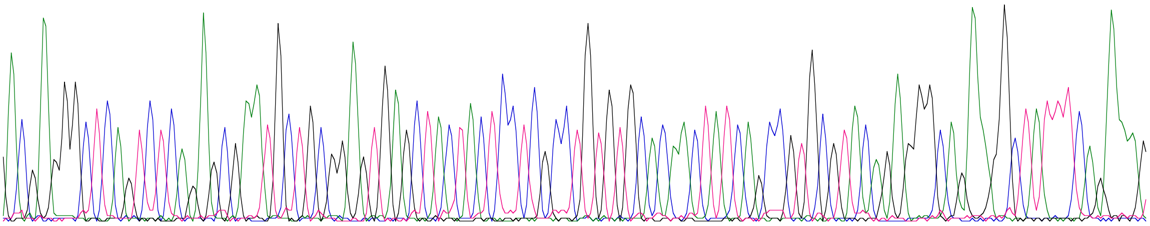

ACGAGGGCTCAGTCTCAGAGCGAATGCTGCGGAGTGAGCTACTACTCCTCGCCTGTGTGCACAACTATCAGCCGTGCGTACAGAGGGCAGAAGGCTATTTCAGAAAG

D02-SEQ4.ab1(1>819)

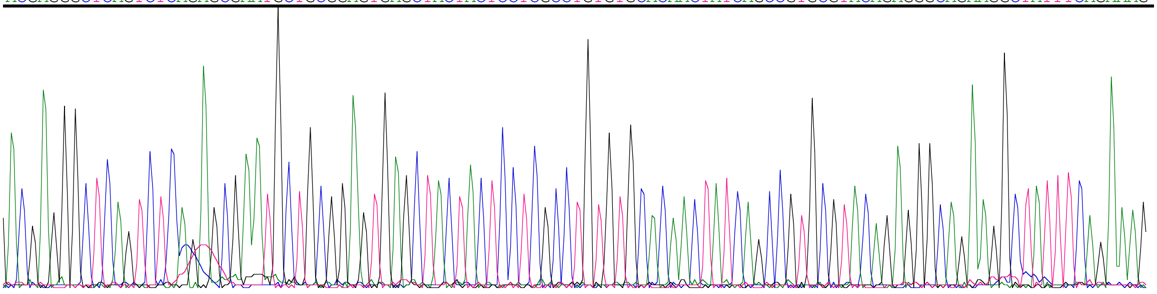

ACGAGGGCTCAGTCTCAGAGCGAATGCTGCGGAGTGAGCTACTACTCCTCGCCTGTGTGCACAACTATCAGCCGTGCGTACAGAGGGCAGAAGGCtaTTTCAGAAAG

C10-PFastBac1-R.ab1(1>811)

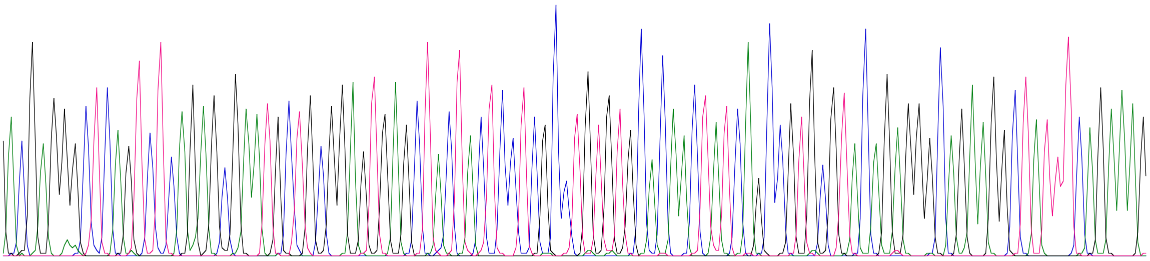

ACGAGGGCTCAGTCTCAGAGCGAATGCTGCGGAGTGAGCTACTACTCCTCGCCTGTGTGCACAACTATCAGCCGTGCGTACAGAGGGCAGAAGGCTATTTCAGAAAG

2,570 2,580 2,590 2,600 2,610 2,620 2,630 2,640 2,650 2,660 2,670

TGGAAGGAATCCAATGGAAACTTGAGCCTGCCTGTCGACGTGACCTTGGCAGTGTTTGCTGTGGGGGCCCAGAGCACAGAAGGCTGGGATTTTCTTTATAGTAAATA

U3823DH140-4.seq(1>2878) → TGGAAGGAATCCAATGGAAACTTGAGCCTGCCTGTCGACGTGACCTTGGCAGTGTTTGCTGTGGGGGCCCAGAGCACAGAAGGCTGGGATTTTCTTTATAGTAAATA

G04-SEQ4RN.ab1(1>811) ←

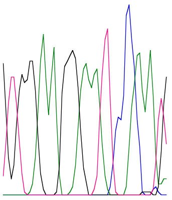

TGGAAGGAATCCAATG

D01-EQ3.ab1(1>819) →

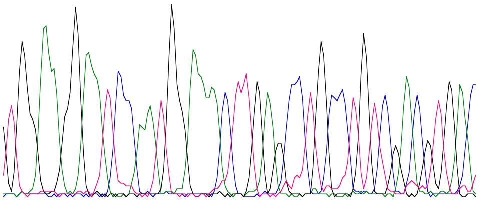

TGGAAGGAATCCAATGGAAACTTGAGCCTGCCTGTCGACGTGACC

D02-SEQ4.ab1(1>819) →

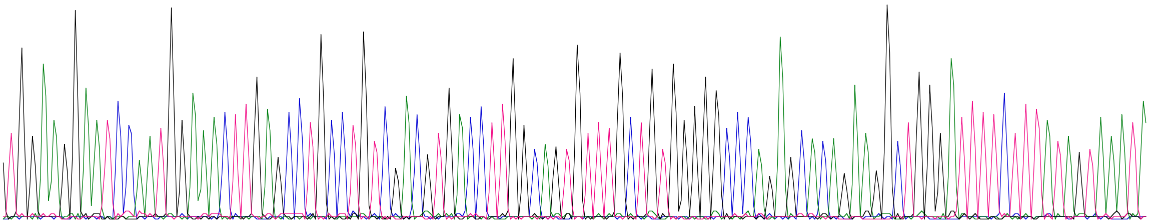

TGGAAGGAATCCAATGGAAACTTGAGCCTGCCTGTCGACGTGACCTTGGCAGTGTTTGCTGTGGGGGCCCAGAGCACAGAAGGCTGGGATTTTCTTTATAGTAAATA

C10-PFastBac1-R.ab1(1>811) ←

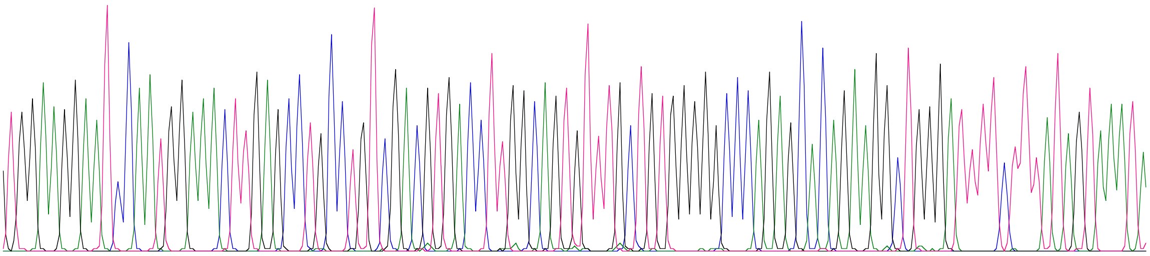

TGGAAGGAATCCAATGGAAACTTGAGCCTGCCTGTCGACGTGACCTTGGCAGTGTTTGCTGTGGGGGCCCAGAGCACAGAAGGCTGGGATTTTCTTTATAGTAAATA

2,680 2,690 2,700 2,710 2,720 2,730 2,740 2,750 2,760 2,770 2,780

TCAGTTTTCTTTGTCCAGTACTGAGAAAAGCCAAATTGAATTTGCCCTCTGCAGAACCCAAAATAAGGAAAAGCTTCAATGGCTACTAGATGAAAGCTTTAAGGGAG

U3823DH140-4.seq(1>2878) → TCAGTTTTCTTTGTCCAGTACTGAGAAAAGCCAAATTGAATTTGCCCTCTGCAGAACCCAAAATAAGGAAAAGCTTCAATGGCTACTAGATGAAAGCTTTAAGGGAG

D02-SEQ4.ab1(1>819) →

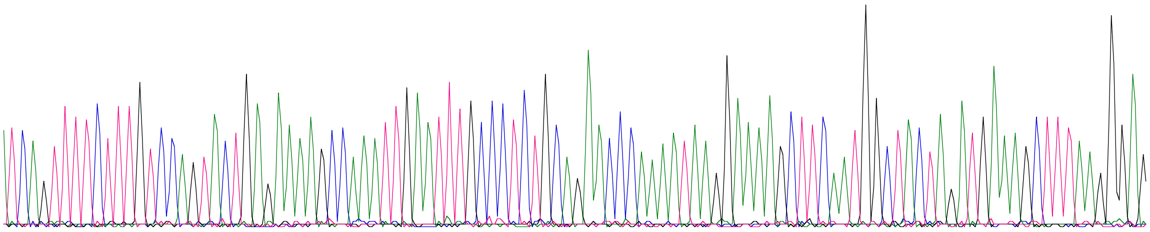

TCAGTTTTCTTTGTCCAGTACTGAGAAAAGCCAAATTGAATTTGCCCTCTGCAGAACCCAAAATAAGGAAAAGCTTCAATGGCTACTAGATGAAAGCTTTAAGGGAG

C10-PFastBac1-R.ab1(1>811) ←

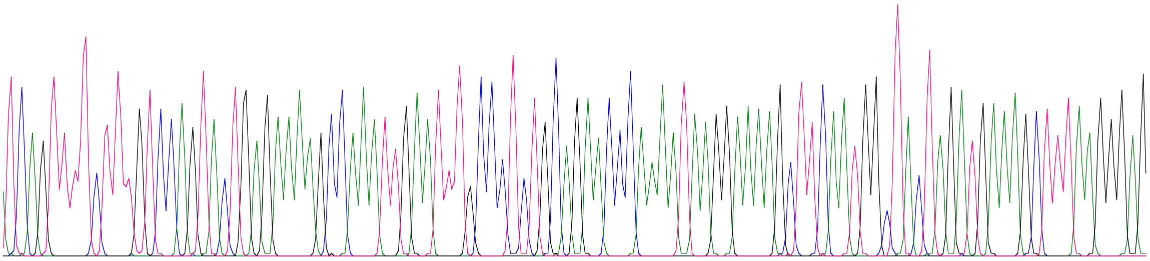

TCAGTTTTCTTTGTCCAGTACTGAGAAAAGCCAAATTGAATTTGCCCTCTGCAGAACCCAAAATAAGGAAAAGCTTCAATGGCTACTAGATGAAAGCTTTAAGGGAG

2,790 2,800 2,810 2,820 2,830 2,840 2,850 2,860 2,870 2,880

ATAAAATAAAAACTCAGGAGTTTCCACAAATTCTTACACTCATTGGCAGGAACCCAGTAGGATACCCACTGGCCTGGCAATTTCTGAGGAAAAACTGGAACAAACTT

U3823DH140-4.seq(1>2878) ATAAAATAAAAACTCAGGAGTTTCCACAAATTCTTACACTCATTGGCAGGAACCCAGTAGGATACCCACTGGCCTGGCAATTTCTGAGGAAAAACTGGAACAAACTT
D02-SEQ4.ab1(1>819)

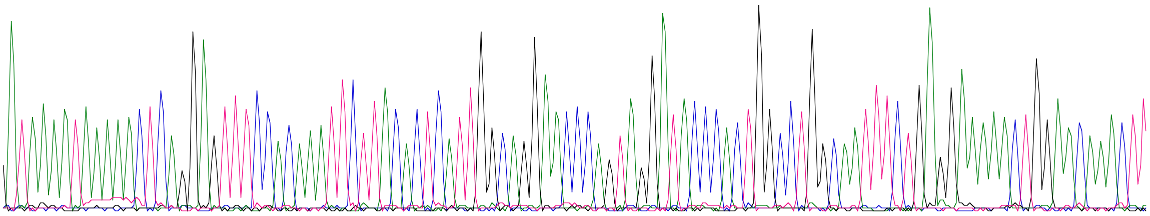

ATAAAATAAAAACTCAGGAGTTTCCACAAATTCTTACACTCATTGGCAGGAACCCAGTAGGATACCCACTGGCCTGGCAATTTCTGAGGAAAAACTGGAACAAACTT

C10-PFastBac1-R.ab1(1>811)

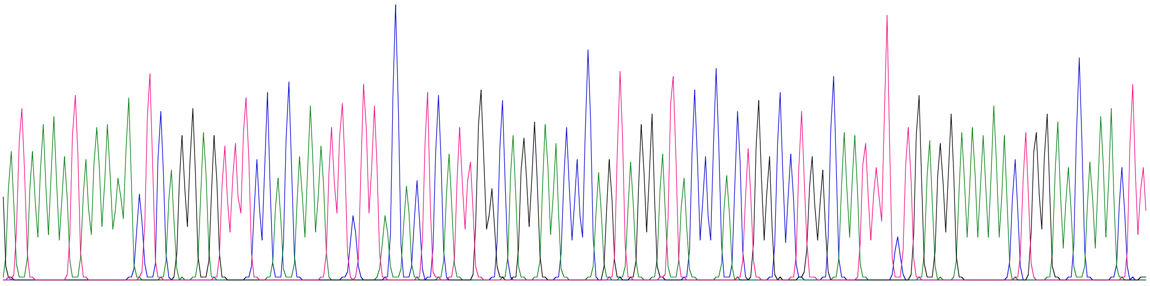

ATAAAATAAAAACTCAGGAGTTTCCACAAATTCTTACACTCATTGGCAGGAACCCAGTAGGATACCCACTGGCCTGGCAATTTCTGAGGAAAAACTGGAACAAACTT

2,890 2,900 2,910 2,920 2,930 2,940 2,950 2,960 2,970 2,980 2,990

GTACAAAAGTTTGAACTTGGCTCATCTTCCATAGCCCACATGGTAATGGGTACAACAAATCAATTCTCCACAAGAACACGGCTTGAAGAGGTAAAAGGATTCTTCAG

U3823DH140-4.seq(1>2878) GTACAAAAGTTTGAACTTGGCTCATCTTCCATAGCCCACATGGTAATGGGTACAACAAATCAATTCTCCACAAGAACACGGCTTGAAGAGGTAAAAGGATTCTTCAG
D02-SEQ4.ab1(1>819)

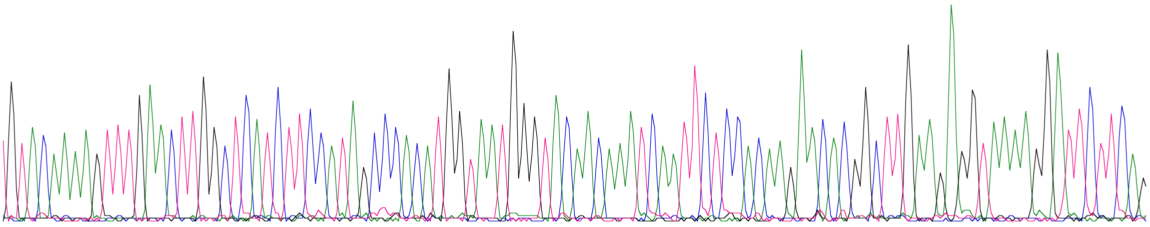

GTACAAAAGTTTGAACTTGGCTCATCTTCCATAGCCCACATGGTAATGGGTACAACAAATCAATTCTCCACAAGAACACGGCTTGAAGAGGTAAAAGGATTCTTCAG

C10-PFastBac1-R.ab1(1>811)

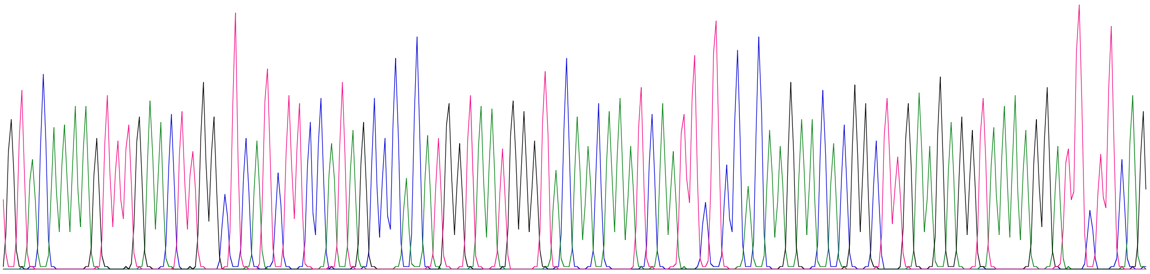

GTACAAAAGTTTGAACTTGGCTCATCTTCCATAGCCCACATGGTAATGGGTACAACAAATCAATTCTCCACAAGAACACGGCTTGAAGAGGTAAAAGGATTCTTCAG

3,000 3,010 3,020 3,030 3,040 3,050 3,060 3,070 3,080 3,090 3,100

CTCTTTGAAAGAAAATGGTTCTCAGCTCCGTTGTGTCCAACAGACAATTGAAACCATTGAAGAAAACATCGGTTGGATGGATAAGAATTTTGATAAAATCAGAGTGT

U3823DH140-4.seq(1>2878) CTCTTTGAAAGAAAATGGTTCTCAGCTCCGTTGTGTCCAACAGACAATTGAAACCATTGAAGAAAACATCGGTTGGATGGATAAGAATTTTGATAAAATCAGAGTGT
D02-SEQ4.ab1(1>819)

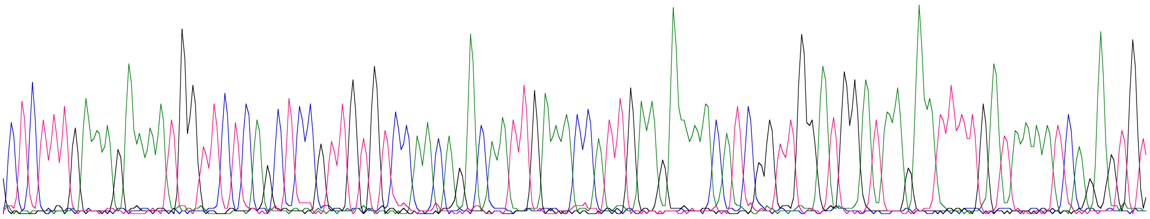

CTCTTTGAAAGAAAATGGTTCTCAGCTCCGTTGTGTCCAACAGACAATTGAAACCATTGAAGAAAACATCGGTTGGATGGATAAGAATTTTGATAAAATCAGAGTGT

3,000 3,010 3,020 3,030 3,040 3,050 3,060 3,070 3,080 3,090 3,100

CTCTTTGAAAGAAAATGGTTCTCAGCTCCGTTGTGTCCAACAGACAATTGAAACCATTGAAGAAAACATCGGTTGGATGGATAAGAATTTTGATAAAATCAGAGTGT

C10-PFastBac1-R.ab1(1>811)

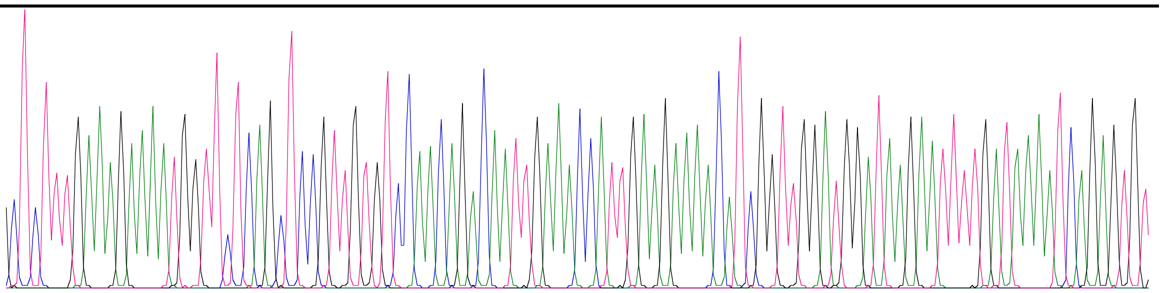

CTCTTTGAAAGAAAATGGTTCTCAGCTCCGTTGTGTCCAACAGACAATTGAAACCATTGAAGAAAACATCGGTTGGATGGATAAGAATTTTGATAAAATCAGAGTGT

3,110 3,120 3,130 3,140 3,150 3,160 3,170 3,180 3,190 3,200 3,210

GGCTGCAAAGTGAAAAGCTTGAACGCATGCATCATCACCATCACCATTAACTCGAGGCATGCGGTACCAAGCTTGTCGAGAAGTACTAGAGGATCATAATCAGCCAT

U3823DH140-4.seq(1>2878) GGCTGCAAAGTGAAAAGCTTGAACGCATGCATCATCACCATCACCATTAACTCGAG

D02-SEQ4.ab1(1>819)

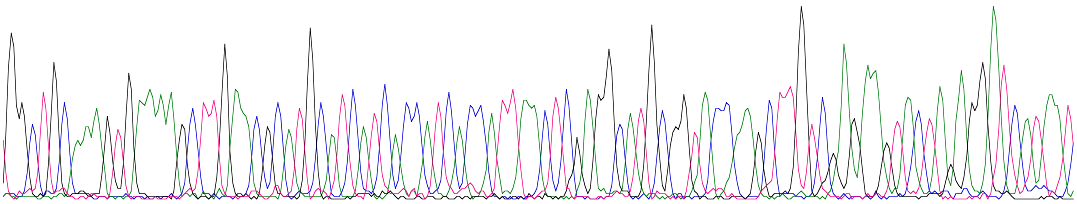

GGCTGCAAAGTGAAAAGCTTGAACGCATGCATCATCACCATCACCATTAACTCGAGGCATGCGGTACCAAGCTTGTCGAGAAGTACTAGAGGATCATAATC

C10-PFastBac1-R.ab1(1>811)

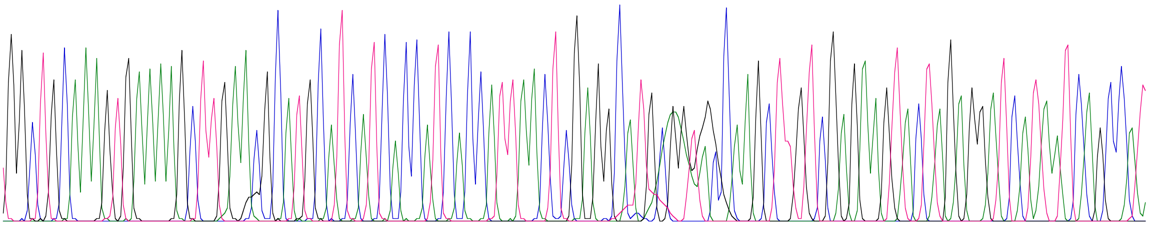

GGCTGCAAAGTGAAAAGCTTGAACGCATGCATCATCACCATCACCATTAACTCGAGGCATGCGGTACCAAGCTTGTCGAGAAGTACTAGAGGATCATAATCAGCCAT

3,220

ACCACATT

C10-PFastBac1-R.ab1(1>811)

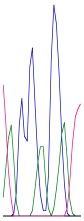

ACCACATT

Haplotype 9

10 20 30 40 50 60 70 80 90 100

GTAATAAAAAAACCTATAAATATTCCGGATTATTCATACCGTCCCACCATCGGGCGCGGATCCAACTCCTAAAAAACCGCCACCATGGTGTTTCTGCCCCTCAAATGG

U4947DH210-10.seq(1>2878) GGATCCAACTCCTAAAAAACCGCCACCATGGTGTTTCTGCCCCTCAAATGG
F02-SEQ3R.ab1(1>810)

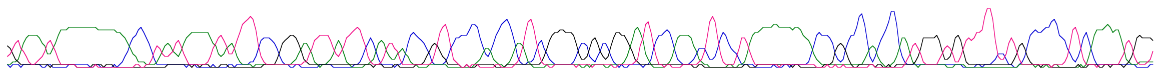

GTAATAAAAAAACCTATAAATATTCCGGATTATTCATACCGTCCCACCATCGGGCGCGGATCCAACTCCTAAAAAACCGCCACCATGGTGTTTCTGCCCCTCAAATGG

D06-pFastbac-F.ab1(1>819)

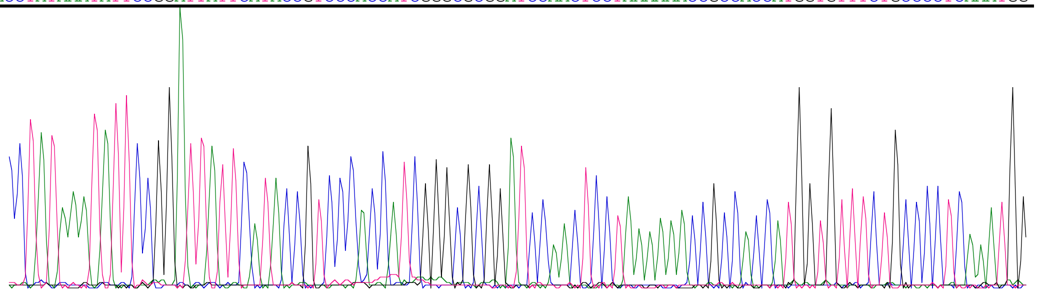

CCTATAAATATTCCGGATTATTCATACCGTCCCACCATCGGGCGCGGATCCAACTCCTAAAAAACCGCCACCATGGTGTTTCTGCCCCTCAAATGG

110 120 130 140 150 160 170 180 190 200 210

TCCCTTGCAACCATGTCATTTCTACTTTCCTCACTGTTGGCTCTCTTAACTGTGTCCACTCCTTCATGGTGTCAGAGCACTGAAGCATCTCCAAAACGTAGTGATGGG

U4947DH210-10.seq(1>2878) TCCCTTGCAACCATGTCATTTCTACTTTCCTCACTGTTGGCTCTCTTAACTGTGTCCACTCCTTCATGGTGTCAGAGCACTGAAGCATCTCCAAAACGTAGTGATGGG
F02-SEQ3R.ab1(1>810)

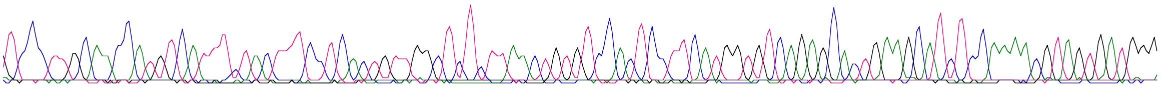

TCCCTTGCAACCATGTCATTTCTACTTTCCTCACTGTTGGCTCTCTTAACTGTGTCCACTCCTTCATGGTGTCAGAGCACTGAAGCATCTCCAAAACGTAGTGATGGG

D06-pFastbac-F.ab1(1>819)

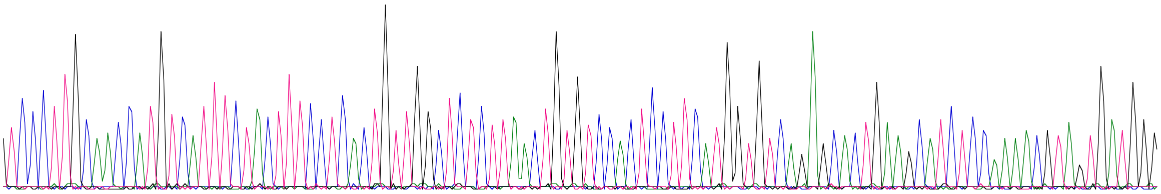

TCCCTTGCAACCATGTCATTTCTACTTTCCTCACTGTTGGCTCTCTTAACTGTGTCCACTCCTTCATGGTGTCAGAGCACTGAAGCATCTCCAAAACGTAGTGATGGG

220 230 240 250 260 270 280 290 300 310 320

ACACCATTTCCTTGGAATAAAATACGACTTCCTGAGTACGTCATCCCAGTTCATTATGATCTCTTGATCCATGCAAACCTTACCACGCTGACCTTCTGGGGAACCACG

U4947DH210-10.seq(1>2878) ACACCATTTCCTTGGAATAAAATACGACTTCCTGAGTACGTCATCCCAGTTCATTATGATCTCTTGATCCATGCAAACCTTACCACGCTGACCTTCTGGGGAACCACG
F02-SEQ3R.ab1(1>810)

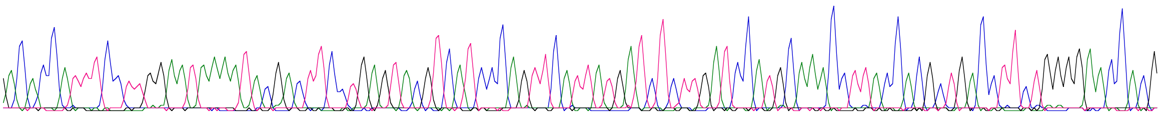

ACACCATTTCCTTGGAATAAAATACGACTTCCTGAGTACGTCATCCCAGTTCATTATGATCTCTTGATCCATGCAAACCTTACCACGCTGACCTTCTGGGGAACCACG

220 230 240 250 260 270 280 290 300 310 320

ACACCATTTCCTTGGAATAAAATACGACTTCCTGAGTACGTCATCCCAGTTCATTATGATCTCTTGATCCATGCAAACCTTACCACGCTGACCTTCTGGGGAACCACG

D06-pFastbac-F.ab1(1>819)

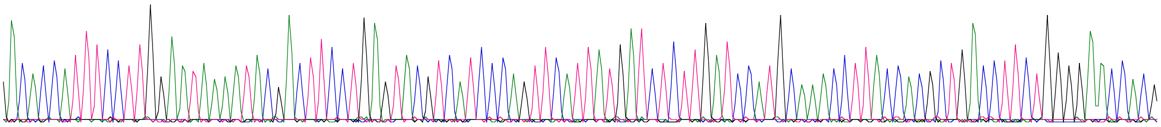

ACACCATTTCCTTGGAATAAAATACGACTTCCTGAGTACGTCATCCCAGTTCATTATGATCTCTTGATCCATGCAAACCTTACCACGCTGACCTTCTGGGGAACCACG

330 340 350 360 370 380 390 400 410 420 430

AAAGTAGAAATCACAGCCAGTCAGCCCACCAGCACCATCATCCTGCATAGTCACCACCTGCAGATATCTAGGGCCACCCTCAGGAAGGGAGCTGGAGAGAGGCTATCG

U4947DH210-10.seq(1>2878)
F02-SEQ3R.ab1(1>810)

AAAGTAGAAATCACAGCCAGTCAGCCCACCAGCACCATCATCCTGCATAGTCACCACCTGCAGATATCTAGGGCCACCCTCAGGAAGGGAGCTGGAGAGAGGCTATCG

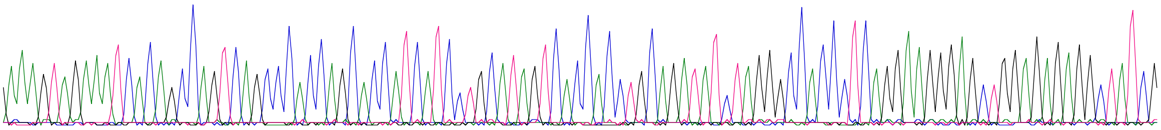

AAAGTAGAAATCACAGCCAGTCAGCCCACCAGCACCATCATCCTGCATAGTCACCACCTGCAGATATCTAGGGCCACCCTCAGGAAGGGAGCTGGAGAGAGGCTATCG

D06-pFastbac-F.ab1(1>819)

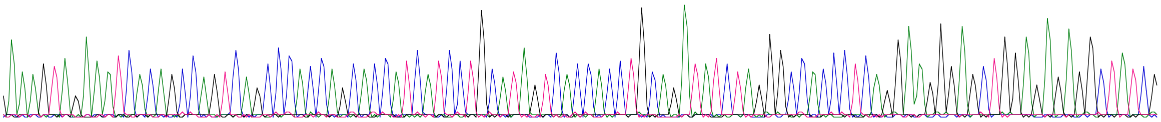

AAAGTAGAAATCACAGCCAGTCAGCCCACCAGCACCATCATCCTGCATAGTCACCACCTGCAGATATCTAGGGCCACCCTCAGGAAGGGAGCTGGAGAGAGGCTATCG

440 450 460 470 480 490 500 510 520 530 540

GAAGAACCCCTGCAGGTCCTGGAACACCCCCCGCAGGAGCAAATTGCACTGCTGGCTCCCGAGCCCCTCCTTGTCGGGCTCCCGTACACAGTTGTCATTCACTATGCT

U4947DH210-10.seq(1>2878)
F02-SEQ3R.ab1(1>810)

GAAGAACCCCTGCAGGTCCTGGAACACCCCCCGCAGGAGCAAATTGCACTGCTGGCTCCCGAGCCCCTCCTTGTCGGGCTCCCGTACACAGTTGTCATTCACTATGCT

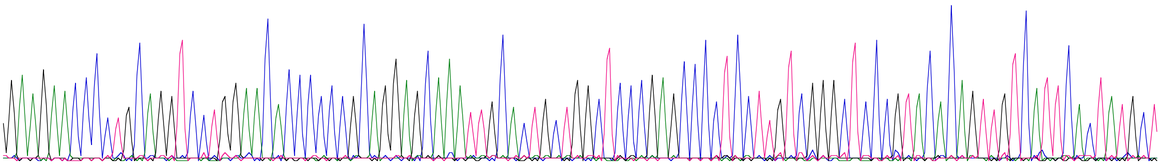

GAAGAACCCCTGCAGGTCCTGGAACACCCCCCGCAGGAGCAAATTGCACTGCTGGCTCCCGAGCCCCTCCTTGTCGGGCTCCCGTACACAGTTGTCATTCACTATGCT

D06-pFastbac-F.ab1(1>819)

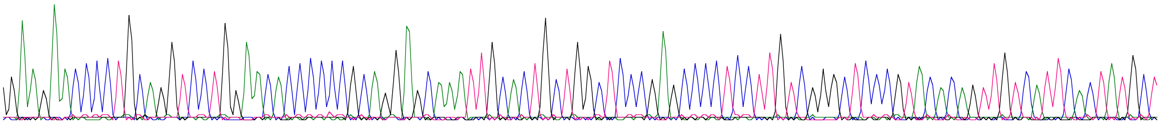

GAAGAACCCCTGCAGGTCCTGGAACACCCCCCGCAGGAGCAAATTGCACTGCTGGCTCCCGAGCCCCTCCTTGTCGGGCTCCCGTACACAGTTGTCATTCACTATGCT

550 560 570 580 590 600 610 620 630 640

GGCAATCTTTCGGAGACTTTCCACGGATTTTACAAAAGCACCTACAGAACCAAGGAAGGGGAACTGAGGATACTAGCATCAACACAATTTGAACCCACTGCAGCTAGA

U4947DH210-10.seq(1>2878) → GGCAATCTTTCGGAGACTTTCCACGGATTTTACAAAAGCACCTACAGAACCAAGGAAGGGGAACTGAGGATACTAGCATCAACACAATTTGAACCCACTGCAGCTAGA

F02-SEQ3R.ab1(1>810) ←

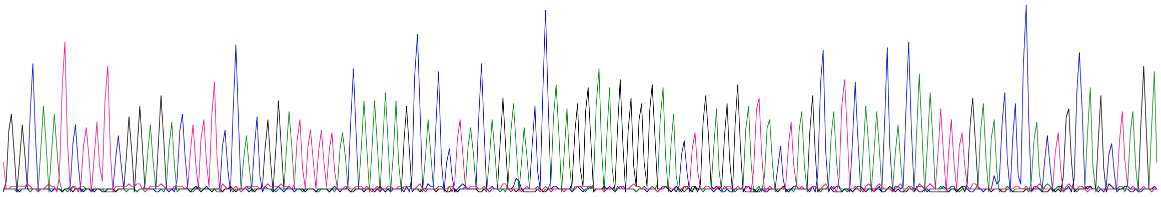

GGCAATCTTTCGGAGACTTTCCACGGATTTTACAAAAGCACCTACAGAACCAAGGAAGGGGAACTGAGGATACTAGCATCAACACAATTTGAaCCCACTGCAGCTAGA

D06-pFastbac-F.ab1(1>819) →

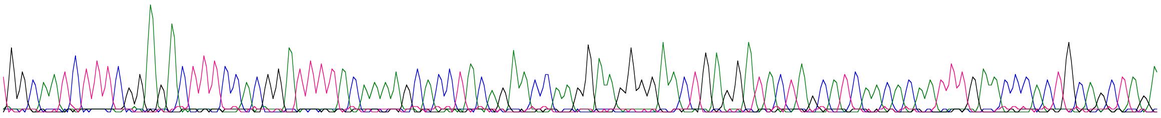

GGCAATCTTTCGGAGACTTTCCACGGATTTTACAAAAGCACCTACAGAACCAAGGAAGGGGAACTGAGGATACTAGCATCAACACAATTTGAACCCACTGCAGCTAGA

650 660 670 680 690 700 710 720 730 740 750

ATGGCCTTTCCCTGCTTTGATGAACCTGCCTTCAAAGCAAGTTTCTCAATCAAAATTAGAAGAGAGCCAAGGCACCTAGCCATCTCCAATATGCCATTGGTGAAATCT

U4947DH210-10.seq(1>2878) → ATGGCCTTTCCCTGCTTTGATGAACCTGCCTTCAAAGCAAGTTTCTCAATCAAAATTAGAAGAGAGCCAAGGCACCTAGCCATCTCCAATATGCCATTGGTGAAATCT

F02-SEQ3R.ab1(1>810) ←

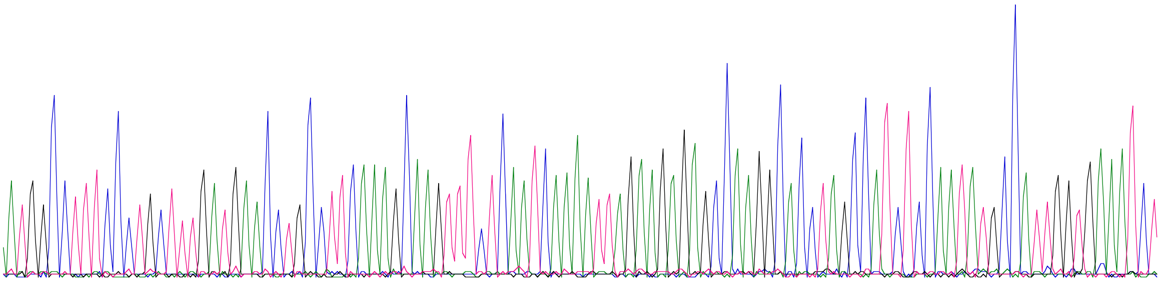

ATGGCCTTTCCCTGCTTTGATGAACCTGCCTTCAAAGCAAGTTTCTCAATCAAAATTAGAAGAGAGCCAAGGCACCTAGCCATCTCCAATATGCCATTGGTGAAATCT

D06-pFastbac-F.ab1(1>819) →

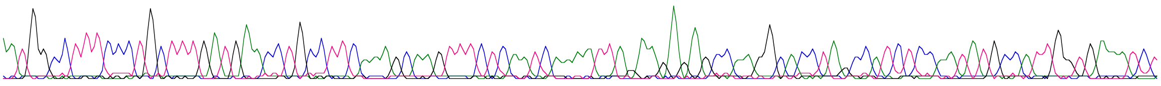

ATGGCCTTTCCCTGCTTTGATGAACCTGCCTTCAAAGCAAGTTTCTCAATCAAAATTAGAAGAGAGCCAAGGCACCTAGCCATCTCCAATATGCCATTGGTGAAATCT

A03-SEQ1.ab1(1>819) →

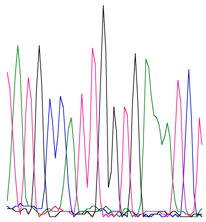

TATGCCATTGGTGAAATCT

760 770 780 790 800 810 820 830 840 850 860

GTGACTGTTGCTGAAGGACTCATAGAAGACCATTTTGATGTCACTGTGAAGATGAGCACCTATCTGGTGGCCTTCATCATTTCAGATTTTGAGTCTGTCAGCAAGATA

U4947DH210-10.seq(1>2878) → GTGACTGTTGCTGAAGGACTCATAGAAGACCATTTTGATGTCACTGTGAAGATGAGCACCTATCTGGTGGCCTTCATCATTTCAGATTTTGAGTCTGTCAGCAAGATA

F02-SEQ3R.ab1(1>810) ←

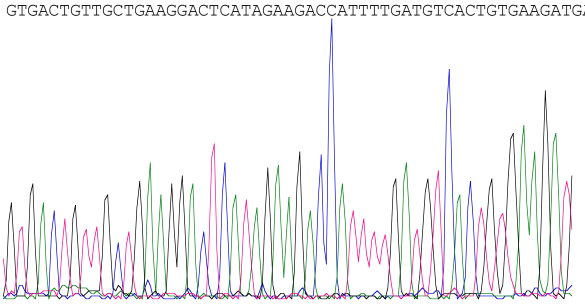

GTGACTGTTGCTGAAGGACTCATAGAAGACCATTTTGATGTCACTGTGAAGATG

D06-pFastbac-F.ab1(1>819) →

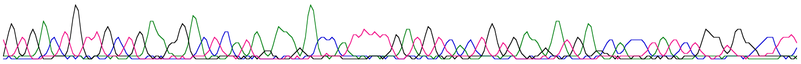

GTGACTGTTGCTGAAGGACTCATAGAAGACCATTTTGATGTCACTGTGAAGATGAGCACCTATCTGGTGGCCTTC

A03-SEQ1.ab1(1>819) →

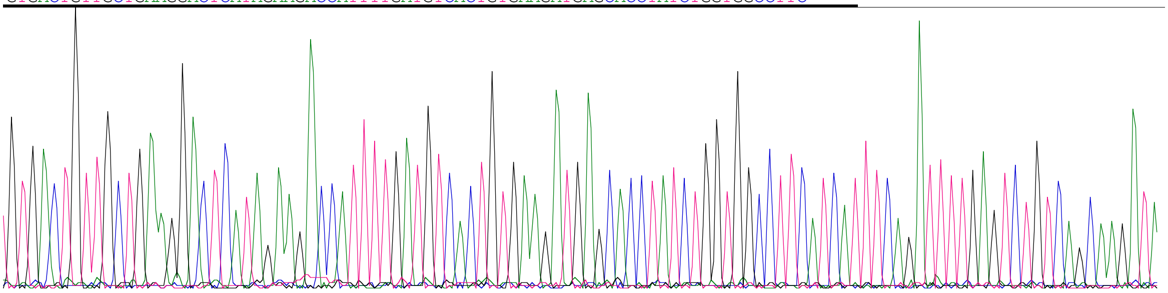

GTGACTGTTGCTGAAGGACTCATAGAAGACCATTTTGATGTCACTGTGAAGATGAGCACCTATCTGGTGGCCTTCATCATTTCAGATTTTGAGTCTGTCAGCAAGATA

F01-SEQ2R.ab1(1>781) ←

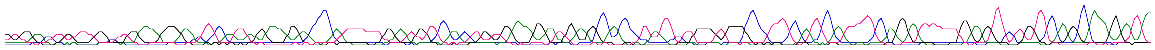

GTGACTGTTGCTGAAGGACTCATAGAAGACCATTTTGATGTCACTGTGAAGATGAGCACcTATCTGGTGGCCTTCATCATTTCAGATTTTGAGTCTGTCAGCAAGATA

I276M

870 880 890 900 910 920 930 940 950 960 970

ACCAAGAGTGGAGTCAAGGTTTCTGTTTATGCTGTGCCAGACAAGATGAATCAAGCAGATTATGCACTGGATGCTGCGGTGACTCTTCTAGAATTTTATGAGGATTAT

U4947DH210-10.seq(1>2878) → ACCAAGAGTGGAGTCAAGGTTTCTGTTTATGCTGTGCCAGACAAGATGAATCAAGCAGATTATGCACTGGATGCTGCGGTGACTCTTCTAGAATTTTATGAGGATTAT

A03-SEQ1.ab1(1>819) →

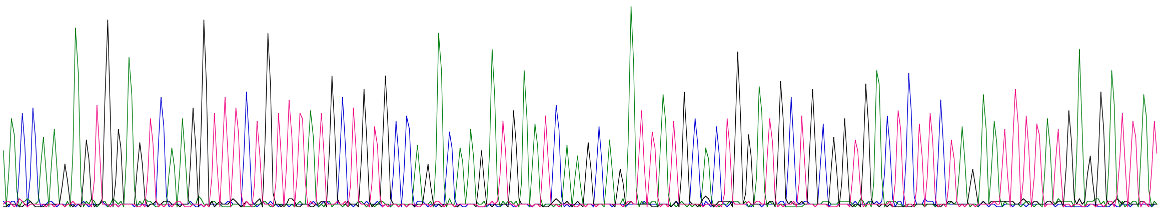

ACCAAGAGTGGAGTCAAGGTTTCTGTTTATGCTGTGCCAGACAAGATGAATCAAGCAGATTATGCACTGGATGCTGCGGTGACTCTTCTAGAATTTTATGAGGATTAT

F01-SEQ2R.ab1(1>781) ←

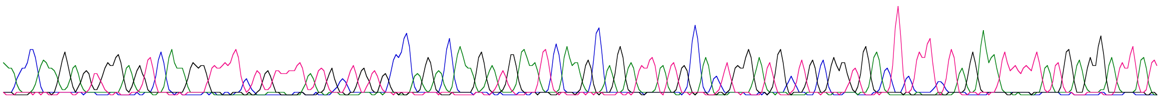

ACCAAGAGTGGAGTCAAGGTTTCTGTTTATGCTGTGCCAGACAAGATGAATCAAGCAGATTATGCACTGGATGCTGCGGTGACTCTTCTAGAATTTTATGAGGATTAT

980 990 1,000 1,010 1,020 1,030 1,040 1,050 1,060 1,070 1,080

TTCAGCATACCGTATCCCCTACCCAAACAAGATCTTGCTGCTATTCCCGACTTTCAGTCTGGTGCTATGGAAAACTGGGGACTGACAACATATAGAGAATCTGCTCTG

U4947DH210-10.seq(1>2878) TTCAGCATACCGTATCCCCTACCCAAACAAGATCTTGCTGCTATTCCCGACTTTCAGTCTGGTGCTATGGAAAACTGGGGACTGACAACATATAGAGAATCTGCTCTG

A03-SEQ1.ab1(1>819)

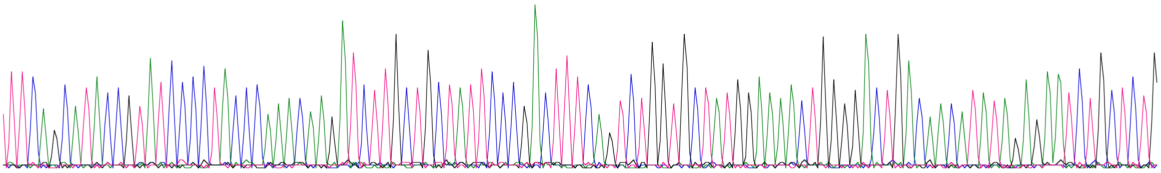

F01-SEQ2R.ab1(1>781)

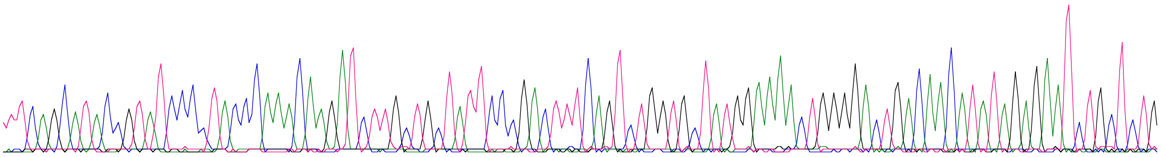

1,090 1,100 1,110 1,120 1,130 1,140 1,150 1,160 1,170 1,180

TTGTTTGATGCAGAAAAGTCTTCTGCATCAAGTAAGCTTGGCATCACAATGACTGTGGCCCATGAACTGGCTCACCAGTGGTTTGGGAACCTGGTCACTATGGAATGG

U4947DH210-10.seq(1>2878) TTGTTTGATGCAGAAAAGTCTTCTGCATCAAGTAAGCTTGGCATCACAATGACTGTGGCCCATGAACTGGCTCACCAGTGGTTTGGGAACCTGGTCACTATGGAATGG

A03-SEQ1.ab1(1>819)

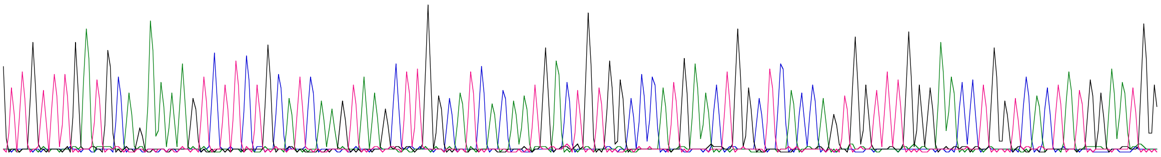

F01-SEQ2R.ab1(1>781)

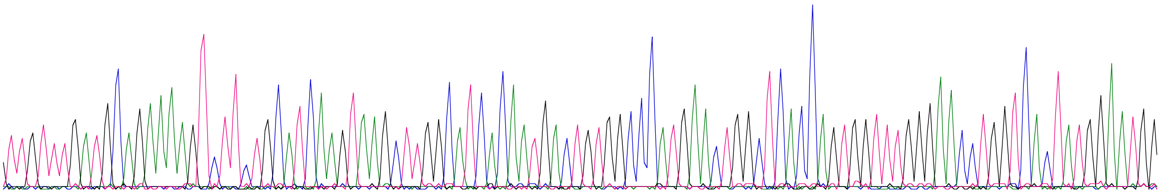

1,190 1,200 1,210 1,220 1,230 1,240 1,250 1,260 1,270 1,280 1,290

TGGAATGATCTTTGGCTAAATGAAGGATTTGCCAAATTTATGGAGTTTGTGTCTGTCAGTGTGACCCATCCTGAACTGAAAGTTGGAGATTATTTCTTTGGCAAATGT

U4947DH210-10.seq(1>2878) TGGAATGATCTTTGGCTAAATGAAGGATTTGCCAAATTTATGGAGTTTGTGTCTGTCAGTGTGACCCATCCTGAACTGAAAGTTGGAGATTATTTCTTTGGCAAATGT

A03-SEQ1.ab1(1>819)

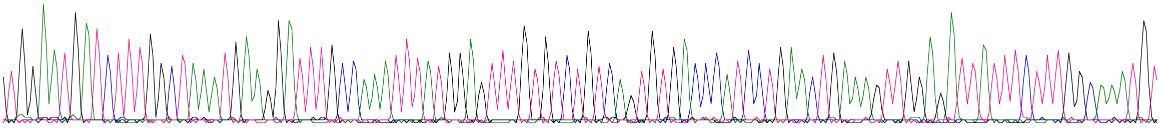

1,190 1,200 1,210 1,220 1,230 1,240 1,250 1,260 1,270 1,280 1,290

TGGAATGATCTTTGGCTAAATGAAGGATTTGCCAAATTTATGGAGTTTGTGTCTGTCAGTGTGACCCATCCTGAACTGAAAGTTGGAGATTATTTCTTTGGCAAATGT

F01-SEQ2R.ab1(1>781) ←

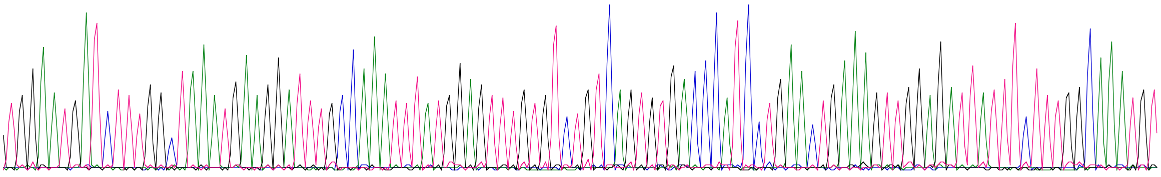

TGGAATGATCTTTGGCTAAATGAAGGATTTGCCAAATTTATGGAGTTTGTGTCTGTCAGTGTGACCCATCCTGAACTGAAAGTTGGAGATTATTTCTTTGGCAAATGT

1,300 1,310 1,320 1,330 1,340 1,350 1,360 1,370 1,380 1,390 1,400

TTTGACGCAATGGAGGTAGATGCTTTAAATTCCTCACACCCTGTGTCTACACCTGTGGAAAATCCTGCTCAGATCCGGGAGATGTTTGATGATGTTTCTTATGATAAG

U4947DH210-10.seq(1>2878) → TTTGACGCAATGGAGGTAGATGCTTTAAATTCCTCACACCCTGTGTCTACACCTGTGGAAAATCCTGCTCAGATCCGGGAGATGTTTGATGATGTTTCTTATGATAAG

A03-SEQ1.ab1(1>819) →

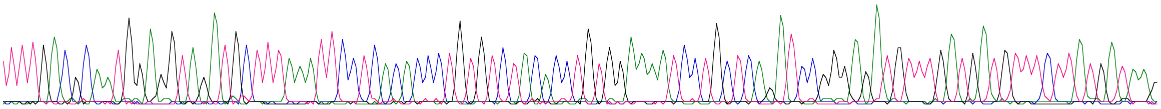

TTTGACGCAATGGAGGTAGATGCTTTAAATTCCTCACACCCTGTGTCTACACCTGTGGAAAATCCTGCTCAGATCCGGGAGATGTTTGATGATGTTTCTTATGATAAG

F01-SEQ2R.ab1(1>781) ←

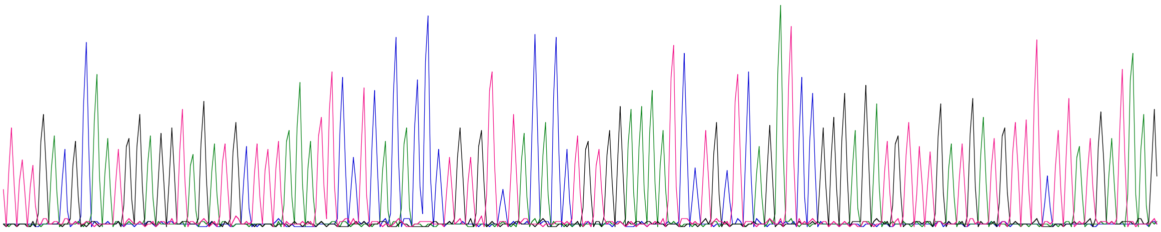

TTTGACGCAATGGAGGTAGATGCTTTAAATTCCTCACACCCTGTGTCTACACCTGTGGAAAATCCTGCTCAGATCCGGGAGATGTTTGATGATGTTTCTTATGATAAG

1,410 1,420 1,430 1,440 1,450 1,460 1,470 1,480 1,490 1,500 1,510

GGAGCTTGTATTCTGAATATGCTAAGGGAGTATCTTAGTGCTGACGCATTTAAAAGTGGTATTGTACAGTATCTCCAGAAGCATAGCTATAAAAATACAAAAAACGAG

U4947DH210-10.seq(1>2878) → GGAGCTTGTATTCTGAATATGCTAAGGGAGTATCTTAGTGCTGACGCATTTAAAAGTGGTATTGTACAGTATCTCCAGAAGCATAGCTATAAAAATACAAAAAACGAG

A03-SEQ1.ab1(1>819) →

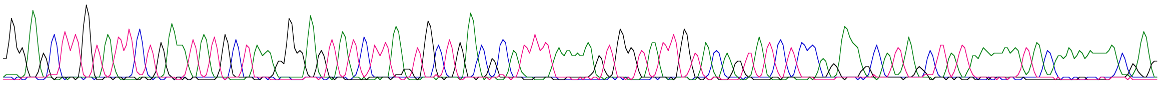

GGAGCTTGTATTCTGAATATGCTAAGGGAGTATCTTAGTGCTGACGCATTTAAAAGTGGTATTGTACAGTATCTCCAGAAGCATAGCTATAAAAATACAAAAAACGAG

F01-SEQ2R.ab1(1>781) ←

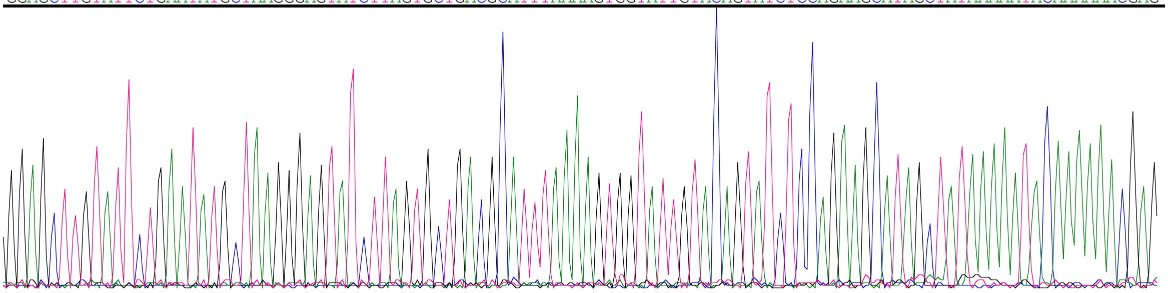

GGAGCTTGTATTCTGAATATGCTAAGGGAGTATCTTAGTGCTGACGCATTTAAAAGTGGTATTGTACAGTATCTCCAGAAGCATAGCTATAAAAATACAAAAAACGAG

1,410 1,420 1,430 1,440 1,450 1,460 1,470 1,480 1,490 1,500 1,510

GGAGCTTGTATTCTGAATATGCTAAGGGAGTATCTTAGTGCTGACGCATTTAAAAGTGGTATTGTACAGTATCTCCAGAAGCATAGCTATAAAAATACAAAAAACGAG

E12-SEQ1R.ab1(1>825)

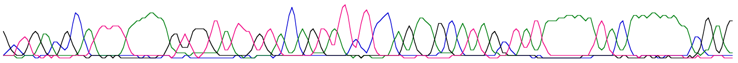

GCTGACGCATTTAAAAGTGGTATTGTACAGTATCTCCAGAAGCATAGCTATAAAAATACAAAAAACGAG

A04-SEQ2.ab1(1>821)

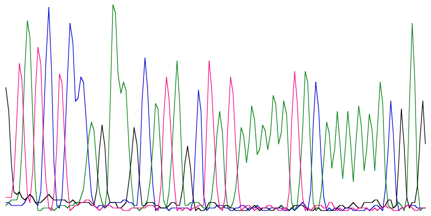

GtATCTCCAGAAGCATAGCTATAAAAATACAAAAAACGAG

1,520 1,530 1,540 1,550 1,560 1,570 1,580 1,590 1,600 1,610 1,620

GACCTGTGGGATAGTATGGCAAGTATTTGCCCTACAGATGGTGTAAAAGGGATGGATGGCTTTTGCTCTAGAAGTCAACATTCATCTTCATCCTCACATTGGCATCAG

U4947DH210-10.seq(1>2878) GACCTGTGGGATAGTATGGCAAGTATTTGCCCTACAGATGGTGTAAAAGGGATGGATGGCTTTTGCTCTAGAAGTCAACATTCATCTTCATCCTCACATTGGCATCAG

A03-SEQ1.ab1(1>819)

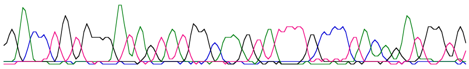

GACCTGTGGGATAGTATGGCAAGTATTTGCCCTACAGATGGTGT

F01-SEQ2R.ab1(1>781)

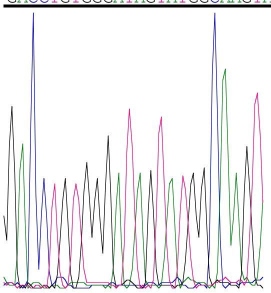

GACCTGTGGGATAGTATGGCAAGTA

E12-SEQ1R.ab1(1>825)

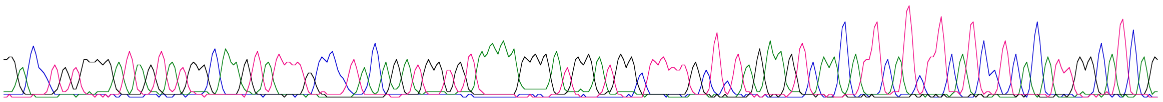

GACCTGTGGGATAGTATGGCAAGTATTTGCCCTACAGATGGTGTAAAAGGGATGGATGGCTTTTGCTCTAGAAGTCAACATTCATCTTCATCCTCACATTGGCATCAG

A04-SEQ2.ab1(1>821)

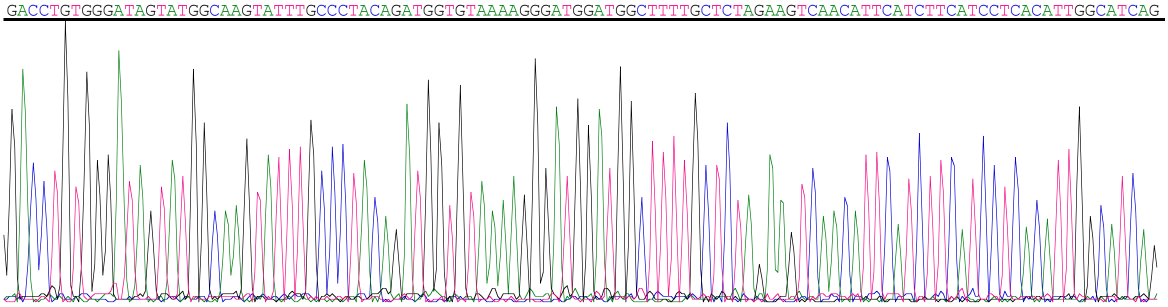

GACCTGTGGGATAGTATGGCAAGTATTTGCCCTACAGATGGTGTAAAAGGGATGGATGGcTTTTGCTCTAGAAGTCAACATTCATCTTCATCCTCACATTGGCATCAG

K528R

1,630 1,640 1,650 1,660 1,670 1,680 1,690 1,700 1,710 1,720

GAAGGGGTGGATGTGAAAACCATGATGAACACTTGGACACTGCAGCGTGGTTTTCCCCTAATAACCATCACAGTGAGGGGGAGGAATGTACACATGAAGCAAGAGCAC

U4947DH210-10.seq(1>2878) GAAGGGGTGGATGTGAAAACCATGATGAACACTTGGACACTGCAGCGTGGTTTTCCCCTAATAACCATCACAGTGAGGGGGAGGAATGTACACATGAAGCAAGAGCAC

E12-SEQ1R.ab1(1>825)

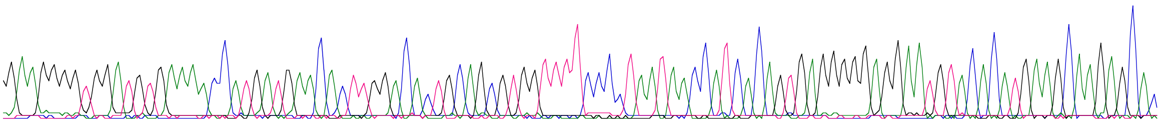

GAAGGGGTGGATGTGAAAACCATGATGAACACTTGGACACTGCAGCGTGGTTTTCCCCTAATAACCATCACAGTGAGGGGGAGGAATGTACACATGAAGCAAGAGCAC

A04-SEQ2.ab1(1>821)

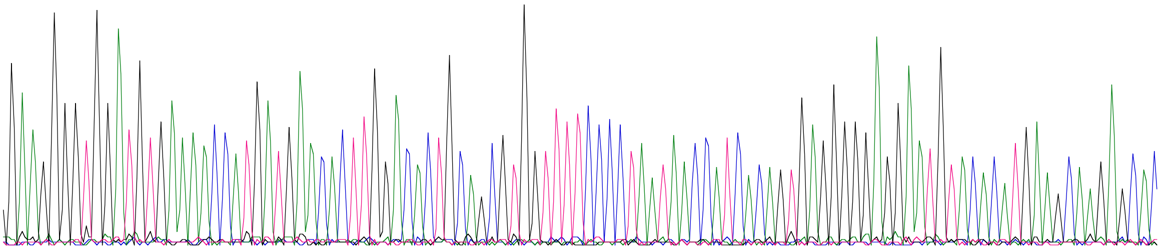

GAAGGGGtGGATGTGAAAACCATGATGAACACTTGGACACTGCAGCGTGGTTTTCCCCTAATAACCATCACAGTGAGGGGGAGGAATGTACACATGAAGCAAGAGCAC

D575N

1,730 1,740 1,750 1,760 1,770 1,780 1,790 1,800 1,810 1,820 1,830

TACATGAAGGGCTCTGACGGCGCCCCGGACACTGGGTACCTGTGGCATGTTCCATTGACATTCATCACCAGCAAATCCAACATGGTCCATCGATTTTTGCTAAAAACA

U4947DH210-10.seq(1>2878) TACATGAAGGGCTCTGACGGCGCCCCGGACACTGGGTACCTGTGGCATGTTCCATTGACATTCATCACCAGCAAATCCAACATGGTCCATCGATTTTTGCTAAAAACA

E12-SEQ1R.ab1(1>825)

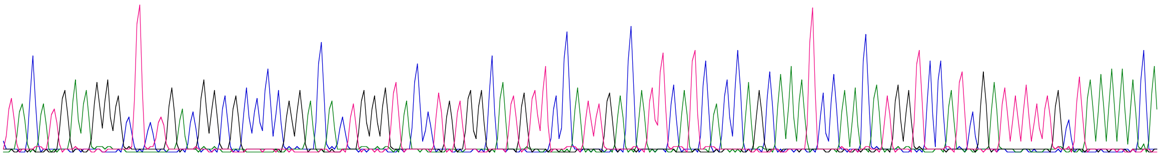

TACATGAAGGGCTCTGACGGCGCCCCGGACACTGGGTACCTGTGGCATGTTCCATTGACATTCATCACCAGCAAATCCAACATGGTCCATCGATTTTTGCTAAAAACA

A04-SEQ2.ab1(1>821)

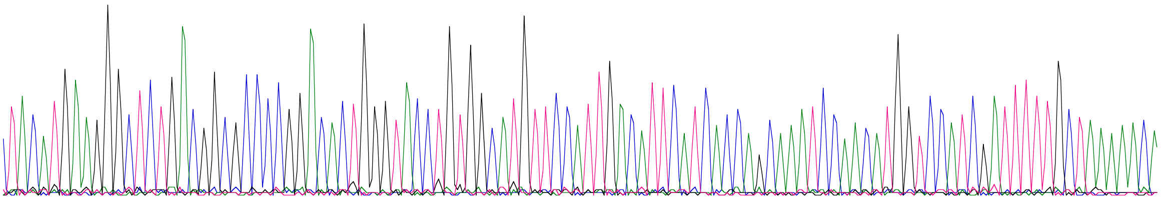

TACATGAAGGGCTCTGACGGCgCCCCGGACACTGGGTACCTGTGGCATGTTCCATTGACATTCATCACCAGCAAATCCAACATGGTCCATCGATTTTTGCTAAAAACA

1,840 1,850 1,860 1,870 1,880 1,890 1,900 1,910 1,920 1,930 1,940

AAAACAGATGTGCTCATCCTCCCAGAAGAGGTGGAATGGATCAAATTTAATGTGGGCATGAATGGCTATTACATTGTGCATTACGAGGATGATGGATGGGACTCTTTG

U4947DH210-10.seq(1>2878) AAAACAGATGTGCTCATCCTCCCAGAAGAGGTGGAATGGATCAAATTTAATGTGGGCATGAATGGCTATTACATTGTGCATTACGAGGATGATGGATGGGACTCTTTG

E12-SEQ1R.ab1(1>825)

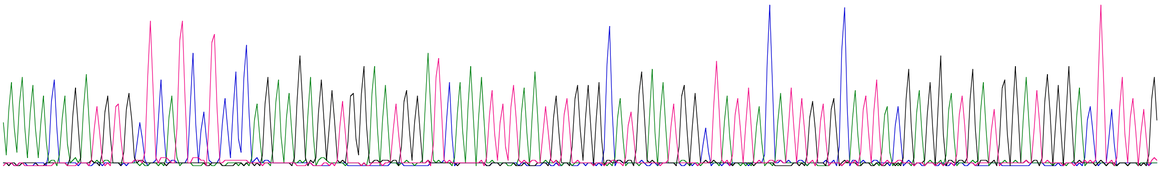

AAAACAGATGTGCTCATCCTCCCAGAAGAGGTGGAATGGATCAAATTTAATGTGGGCATGAATGGCTATTACATTGTGCATTACGAGGATGATGGATGGGACTCTTTG

1,840 1,850 1,860 1,870 1,880 1,890 1,900 1,910 1,920 1,930 1,940

AAAACAGATGTGCTCATCCTCCCAGAAGAGGTGGAATGGATCAAATTTAATGTGGGCATGAATGGCTATTACATTGTGCATTACGAGGATGATGGATGGGACTCTTTG

A04-SEQ2.ab1(1>821)

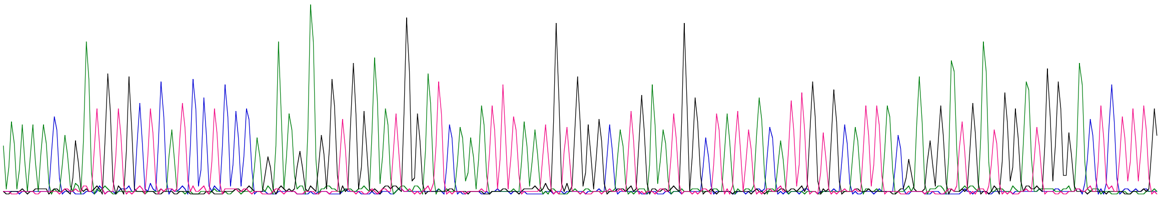

AAAACAGATGTGCTCATCCTCCCAGAAGAGGTGGAATGGATCAAATTTAATGTGGGCATGAATGGCTATTACATTGTGCATTACGAGGATGATGGATGGGACTCTTTG

1,950 1,960 1,970 1,980 1,990 2,000 2,010 2,020 2,030 2,040 2,050

ACTGGCCTTTTAAAAGGAACACACACAGCAGTCAGCAGTAATGATCGGGCGAGTCTCATTAACAATGCATTTCAGCTCGTCAGCATTGGGAAGCTGTCCATTGAAAAG

U4947DH210-10.seq(1>2878) ACTGGCCTTTTAAAAGGAACACACACAGCAGTCAGCAGTAATGATCGGGCGAGTCTCATTAACAATGCATTTCAGCTCGTCAGCATTGGGAAGCTGTCCATTGAAAAG
E12-SEQ1R.ab1(1>825)

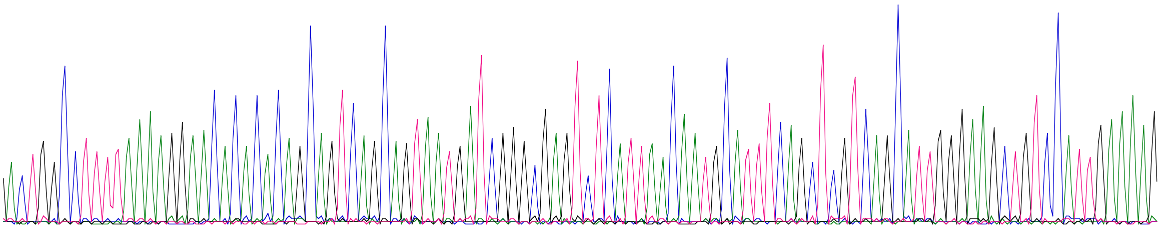

ACTGGCCTTTTAAAAGGAACACACACAGCAGTCAGCAGTAATGATCGGGCGAGTCTCATTAACAATGCATTTCAGCTCGTCAGCATTGGGAAGCTGTCCATTGAAAAG

A04-SEQ2.ab1(1>821)

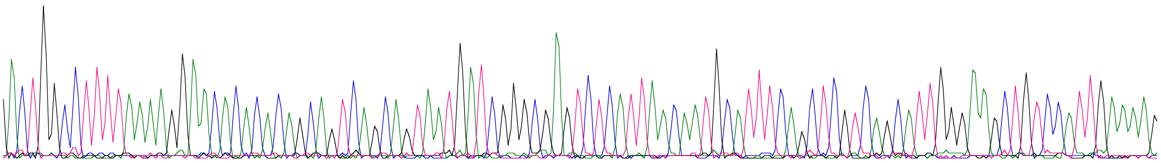

ACTGGCCTTTTAAAAGGAACACACACAGCAGTCAGCAGTAATGATCGGGCGAGTCTCATTAACAATGCATTTCAGCTCGTCAGCATTGGGAAGCTGTCCATTGAAAAG

2,060 2,070 2,080 2,090 2,100 2,110 2,120 2,130 2,140 2,150 2,160

GCCTTGGATTTATCCCTGTACTTGAAACATGAAACTGAAATTATGCCCGTGTTTCAAGGTTTGAATGAGCTGATTCCTATGTATAAGTTAATGGAGAAAAGAGATATG

U4947DH210-10.seq(1>2878) GCCTTGGATTTATCCCTGTACTTGAAACATGAAACTGAAATTATGCCCGTGTTTCAAGGTTTGAATGAGCTGATTCCTATGTATAAGTTAATGGAGAAAAGAGATATG
E12-SEQ1R.ab1(1>825)

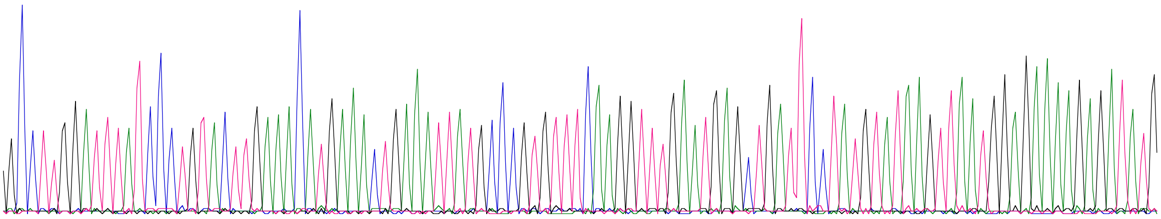

GCCTTGGATTTATCCCTGTACTTGAAACATGAAACTGAAATTATGCCCGTGTTTCAAGGTTTGAATGAGCTGATTCCTATGTATAAGTTAATGGAGAAAAGAGATATG

A04-SEQ2.ab1(1>821)

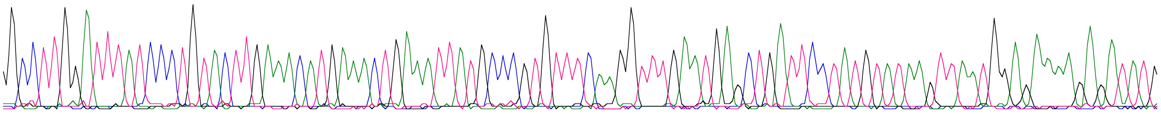

GCCTTGGATTTATCCCTGTACTTGAAACATGAAACTGAAATTATGCCCGTGTTTCAAGGTTTGAATGAGCTGATTCCTATGTATAAGTTAATGGAGAAAAGAGATATG

2,170 2,180 2,190 2,200 2,210 2,220 2,230 2,240 2,250 2,260

AATGAAGTGGAAACTCAATTCAAGGCCTTCCTCATCAGGCTGCTAAGGGACCTCATTGATAAGCAGACATGGACAGACGAGGGCTCAGTCTCAGAGCGAATGCTGCGG

U4947DH210-10.seq(1>2878) AATGAAGTGGAAACTCAATTCAAGGCCTTCCTCATCAGGCTGCTAAGGGACCTCATTGATAAGCAGACATGGACAGACGAGGGCTCAGTCTCAGAGCGAATGCTGCGG

E12-SEQ1R.ab1(1>825)

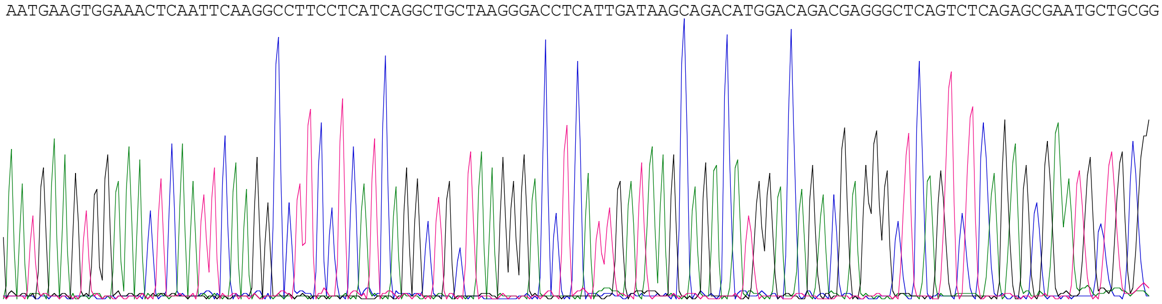

AATGAAGTGGAAACTCAATTCAAGGCCTTCCTCATCAGGCTGCTAAGGGACCTCATTGATAAGCAGACATGGACAGACGAGGGCTCAGTCTCAGAGCGAATGCTGCGG

A04-SEQ2.ab1(1>821)

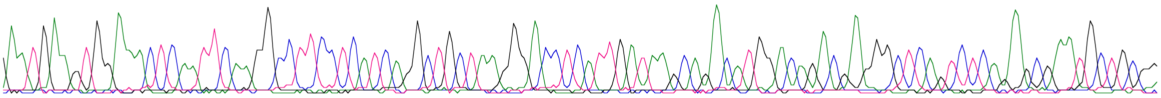

AATGAAGTGGAAACTCAATTCAAGGCCTTCCTCATCAGGCTGCTAAGGGACCTCATTGATAAGCAGACATGGACAGACGAGGGCTCAGTCTCAGAGCGAATGCTGCGG

A02-pFastbac-R.ab1(1>817)

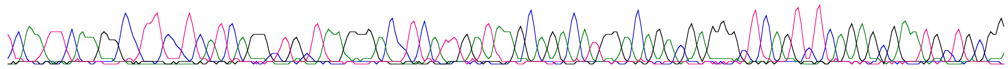

TCAATTCAAGGCCTTCCTCATCAGGCTGCTAAGGGACCTCATTGATAAGCAGACATGGACAGACGAGGGCTCAGTCTCAGAGCGAATGCTGCGG

A05-SEQ3.ab1(1>826)

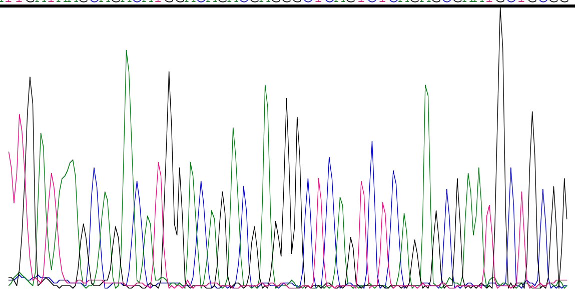

TTGATAAGCAGACATGGACAGACGAGGGCTCAGTCTCAGAGCGAAtGCTGCGG

2,270 2,280 2,290 2,300 2,310 2,320 2,330 2,340 2,350 2,360 2,370

AGTGAGCTACTACTCCTCGCCTGTGTGCACAACTATCAGCCGTGCGTACAGAGGGCAGAAGGCTATTTCAGAAAGTGGAAGGAATCCAATGGAAACTTGAGCCTGCCT

U4947DH210-10.seq(1>2878) AGTGAGCTACTACTCCTCGCCTGTGTGCACAACTATCAGCCGTGCGTACAGAGGGCAGAAGGCTATTTCAGAAAGTGGAAGGAATCCAATGGAAACTTGAGCCTGCCT

A04-SEQ2.ab1(1>821)

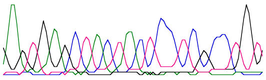

AGTGAGCTACTACTCCTCGCCTGTG

A02-pFastbac-R.ab1(1>817)

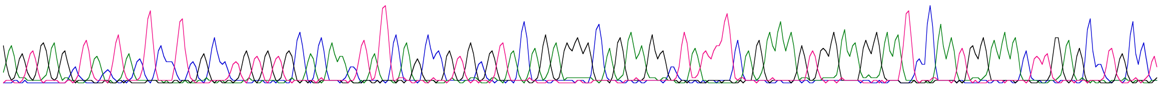

AGTGAGCTACTACTCCTCGCCTGTGTGCACAACTATCAGCCGTGCGTACAGAGGGCAGAAGGCTATTTCAGAAAGTGGAAGGAATCCAATGGAAACTTGAGCCTGCCT

Q730E

2,270 2,280 2,290 2,300 2,310 2,320 2,330 2,340 2,350 2,360 2,370

AGTGAGCTACTACTCCTCGCCTGTGTGCACAACTATCAGCCGTGCGTACAGAGGGCAGAAGGCTATTTCAGAAAGTGGAAGGAATCCAATGGAAACTTGAGCCTGCCT

A05-SEQ3.ab1(1>826)

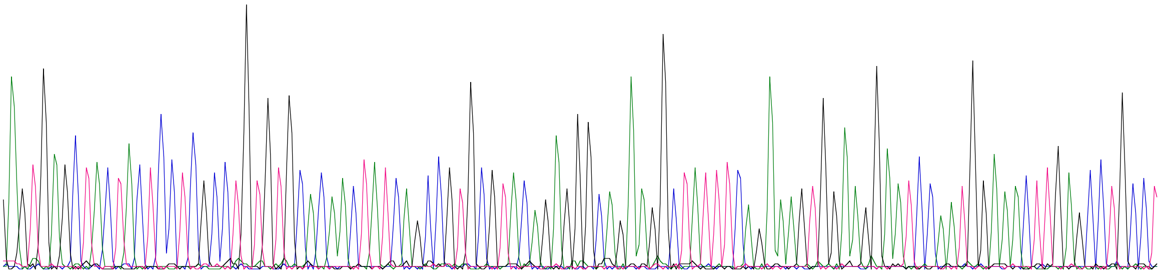

AGTGAGCTACTACTCCTCGCCTGTGTGCACAACTATCAGCCGTGCGTACAGAGGGCAGAAGGCTATTTCAGAAAGTGGAAGGAATCCAATGGAAACTTGAGCCTGCCT

2,380 2,390 2,400 2,410 2,420 2,430 2,440 2,450 2,460 2,470 2,480

GTCGACGTGACCTTGGCAGTGTTTGCTGTGGGGGCCCAGAGCACAGAAGGCTGGGATTTTCTTTATAGTAAATATCAGTTTTCTTTGTCCAGTACTGAGAAAAGCCAA

U4947DH210-10.seq(1>2878)
A02-pFastbac-R.ab1(1>817)

GTCGACGTGACCTTGGCAGTGTTTGCTGTGGGGGCCCAGAGCACAGAAGGCTGGGATTTTCTTTATAGTAAATATCAGTTTTCTTTGTCCAGTACTGAGAAAAGCCAA

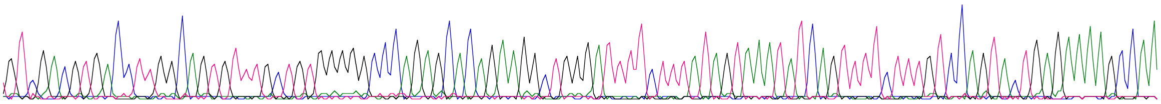

GTCGACGTGACCTTGGCAGTGTTTGCTGTGGGGGCCCAGAGCACAGAAGGCTGGGATTTTCTTTATAGTAAATATCAGTTTTCTTTGTCCAGTACTGAGAAAAGCCAA

A05-SEQ3.ab1(1>826)

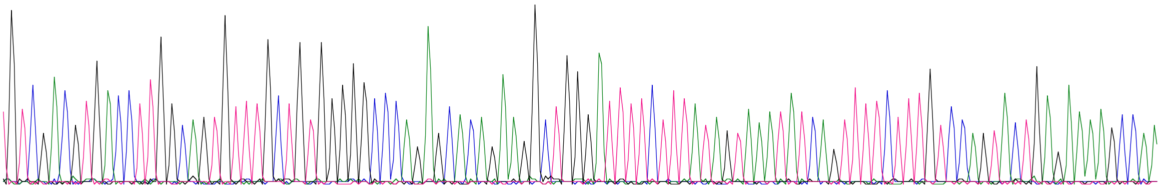

GTCGACGTGACCTTGGCAGTGTTTGCTGTGGGGGCCCAGAGCACAGAAGGCTGGGATTTTCTTTATAGTAAATATCAGTTTTCTTTGTCCAGTACTGAGAAAAGCCAA

2,490 2,500 2,510 2,520 2,530 2,540 2,550 2,560 2,570 2,580 2,590

ATTGAATTTGCCCTCTGCAGAACCCAAAATAAGGAAAAGCTTCAATGGCTACTAGATGAAAGCTTTAAGGGAGATAAAATAAAAACTCAGGAGTTTCCACAAATTCTT

U4947DH210-10.seq(1>2878)
A02-pFastbac-R.ab1(1>817)

ATTGAATTTGCCCTCTGCAGAACCCAAAATAAGGAAAAGCTTCAATGGCTACTAGATGAAAGCTTTAAGGGAGATAAAATAAAAACTCAGGAGTTTCCACAAATTCTT

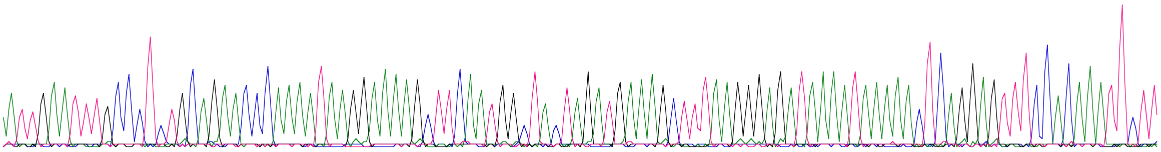

ATTGAATTTGCCCTCTGCAGAACCCAAAATAAGGAAAAGCTTCAATGGCTACTAGATGAAAGCTTTAAGGGAGATAAAATAAAAACTCAGGAGTTTCCACAAATTCTT

A05-SEQ3.ab1(1>826)

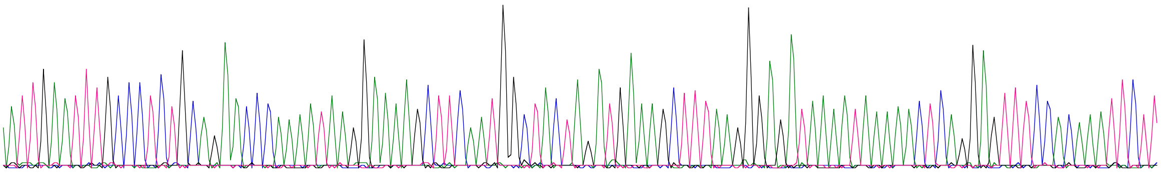

ATTGAATTTGCCCTCTGCAGAACCCAAAATAAGGAAAAGCTTCAATGGCTACTAGATGAAAGCTTTAAGGGAGATAAAATAAAAACTCAGGAGTTTCCACAAATTCTT

2,600 2,610 2,620 2,630 2,640 2,650 2,660 2,670 2,680 2,690 2,700

ACACTCATTGGCAGGAACCCAGTAGGATACCCACTGGCCTGGCAATTTCTGAGGAAAAACTGGAACAAACTTGTACAAAAGTTTGAACTTGGCTCATCTTCCATAGCC

U4947DH210-10.seq(1>2878) ACACTCATTGGCAGGAACCCAGTAGGATACCCACTGGCCTGGCAATTTCTGAGGAAAAACTGGAACAAACTTGTACAAAAGTTTGAACTTGGCTCATCTTCCATAGCC
A02-pFastbac-R.ab1(1>817)

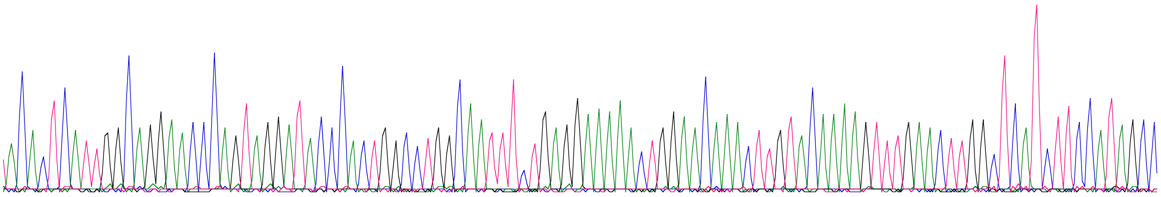

ACACTCATTGGCAGGAACCCAGTAGGATACCCACTGGCCTGGCAATTTCTGAGGAAAAACTGGAACAAACTTGTACAAAAGTTTGAACTTGGCTCATCTTCCATAGCC

A05-SEQ3.ab1(1>826)

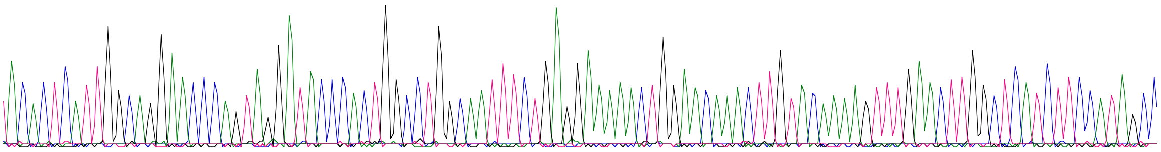

ACACTCATTGGCAGGAACCCAGTAGGATACCCACTGGCCTGGCAATTTCTGAGGAAAAACTGGAACAAACTTGTACAAAAGTTTGAACTTGGCTCATCTTCCATAGCC

2,710 2,720 2,730 2,740 2,750 2,760 2,770 2,780 2,790 2,800

CACATGGTAATGGGTACAACAAATCAATTCTCCACAAGAACACGGCTTGAAGAGGTAAAAGGATTCTTCAGCTCTTTGAAAGAAAATGGTTCTCAGCTCCGTTGTGTC

U4947DH210-10.seq(1>2878) CACATGGTAATGGGTACAACAAATCAATTCTCCACAAGAACACGGCTTGAAGAGGTAAAAGGATTCTTCAGCTCTTTGAAAGAAAATGGTTCTCAGCTCCGTTGTGTC
A02-pFastbac-R.ab1(1>817)

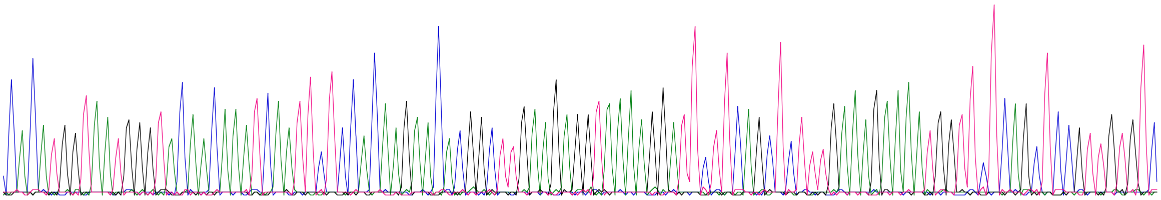

CACATGGTAATGGGTACAACAAATCAATTCTCCACAAGAACACGGCTTGAAGAGGTAAAAGGATTCTTCAGCTCTTTGAAAgAAAATGGTTcTCAGCTCCGTTGTGTC

A05-SEQ3.ab1(1>826)

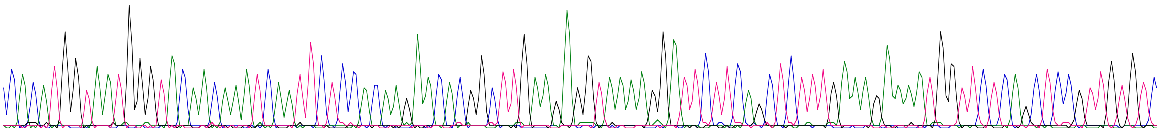

CACATGGTAATGGGTACAACAAATCAATTCTCCACAAGAACACGGCTTGAAGAGGTAAAAGGATTCTTCAGCTCTTTGAAAGAAAATGGTTCTCAGCTCCGTTGTGTC

2,810 2,820 2,830 2,840 2,850 2,860 2,870 2,880 2,890 2,900 2,910

CAACAGACAATTGAAACCATTGAAGAAAACATCGGTTGGATGGATAAGAATTTTGATAAAATCAGAGTGTGGCTGCAAAGTGAAAAGCTTGAACGCATGCATCATCAC

U4947DH210-10.seq(1>2878) CAACAGACAATTGAAACCATTGAAGAAAACATCGGTTGGATGGATAAGAATTTTGATAAAATCAGAGTGTGGCTGCAAAGTGAAAAGCTTGAACGCATGCATCATCAC
A02-pFastbac-R.ab1(1>817)

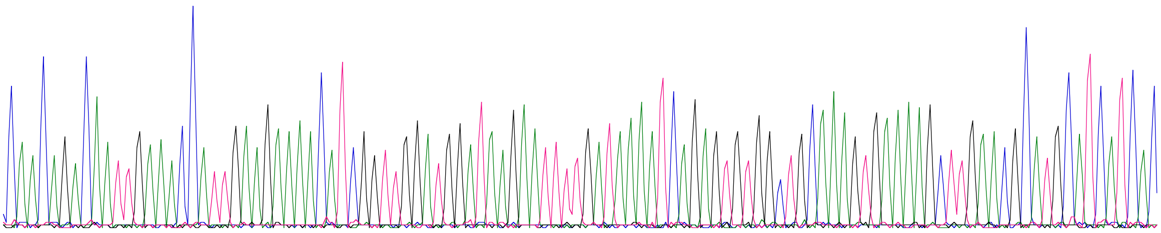

CAACAGACAATTGAAACCATTGAAGAAAACATCGGTTGGATGGATAAGAATTTTGATAAAATCAGAGTGTGGCTGCAAAGTGAAAAGCTTGAACGCATGCATCATCAC

2,810 2,820 2,830 2,840 2,850 2,860 2,870 2,880 2,890 2,900 2,910

CAACAGACAATTGAAACCATTGAAGAAAACATCGGTTGGATGGATAAGAATTTTGATAAAATCAGAGTGTGGCTGCAAAGTGAAAAGCTTGAACGCATGCATCATCAC

A05-SEQ3.ab1(1>826)

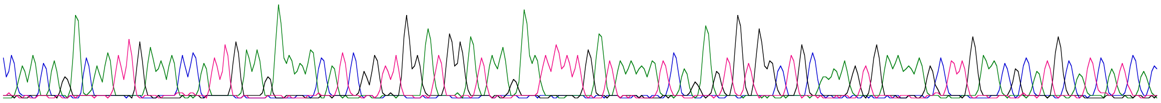

CAACAGACAATTGAAACCATTGAAGAAAACATCGGTTGGATGGATAAGAATTTTGATAAAATCAGAGTGTGGCTGCAAAGTGAAAAGCTTGAACGCATGCATCATCAC

2,920 2,930 2,940 2,950 2,960 2,970 2,980 2,990 3,000 3,010 3,020

CATCACCATTAACTCGAGGCATGCGGTACCAAGCTTGTCGAGAAGTACTAGAGGATCATAATCAGCCATACCACATTTGTAGAGGTTTTACTTGCTTTAAAAAACCTC

U4947DH210-10.seq(1>2878) CATCACCATTAACTCGAG

A02-pFastbac-R.ab1(1>817)

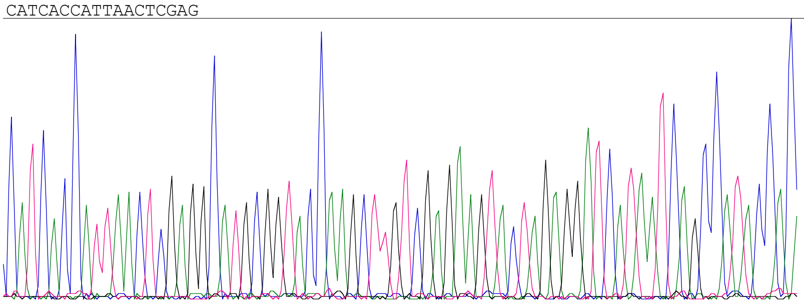

CATCACCATTAACTCGAGGCATGCGGTACCAAGCTTGTCGAGAAGTACTAGAGGATCATAATCAGCCATACCACA

A05-SEQ3.ab1(1>826)

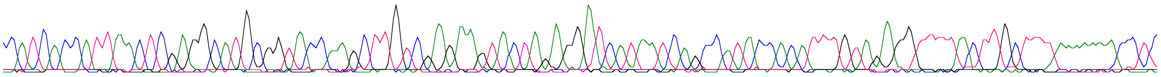

CATCACCATTAACTCGAGGCATGCGGTACCAAGCTTGTCGAGAAGTACTAGAGGATCATAATCAGCCATACCACATTTGTAGAGGTTTTACTTGCTTTAAAAAACCTC

3,030 3,040

CCACACCTCCCCCTGAA

A05-SEQ3.ab1(1>826)

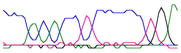

CCACACCTCCCCCTGAA

10 20 30 40 50 60 70 80 90 100

ATCGGAAGACCTCGGCCGTCGCGGCGCTTGCCGGTGGTGCTGACCCCGGATGAAGTGGTTCGCATCCTCGGTTTTCTGGAAGGCGAGCATCGTTTGTTCGCCCAGGA

A05-SEQ1R.ab1(1>821)

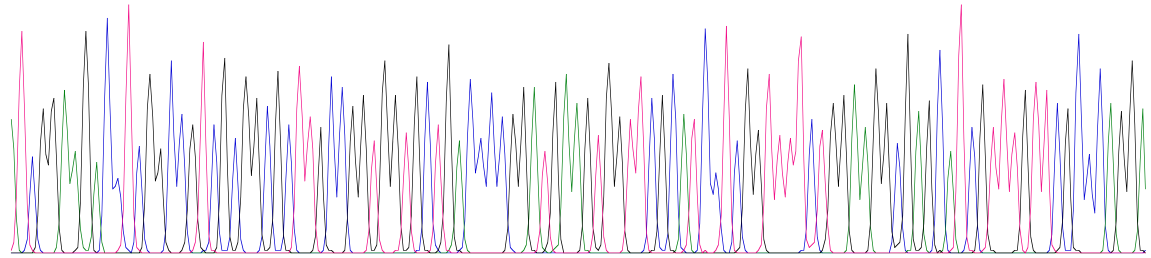

ATCGGAAGACCTCGGCCGTCGCGGCGCTTGCCGGTGGTGCTGACCCCGGATGAAGTGGTTCGCATCCTCGGTTTTCTGGAAGGCGAGCATCGTTTGTTCGCCCAGGA

110 120 130 140 150 160 170 180 190 200 210

CTCTAGCTATAGTTCTAGTGGTTGGCTACGTATACTCCGGAATATTAATAGATCATGGAGATAATTAAAATGATAACCATCTCGCAAATAAATAAGTATTTTACTGT

A05-SEQ1R.ab1(1>821)

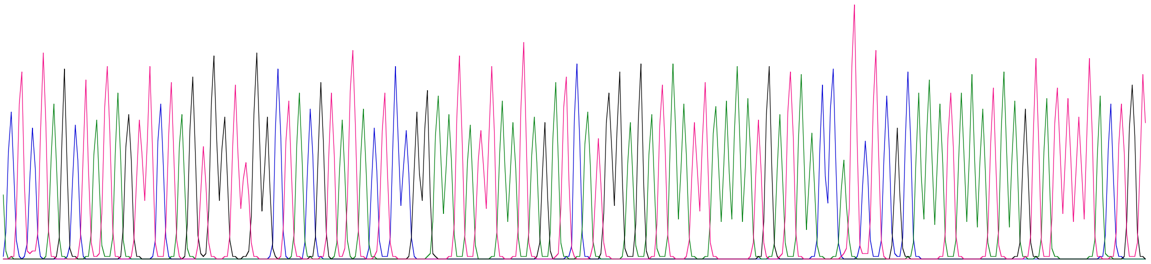

CTCTAGCTATAGTTCTAGTGGTTGGCTACGTATACTCCGGAATATTAATAGATCATGGAGATAATTAAAATGATAACCATCTCGCAAATAAATAAGTATTTTACTGT

220 230 240 250 260 270 280 290 300 310 320

TTTCGTAACAGTTTTGTAATAAAAAAAACCTATAAATATTCCGGATTATTCATACCGTCCCACCATCGGGCGCGGATCCAACTCCTAAAAAAACCGCCACCATGGTGTT

U3823DH140-2.seq(1>2878)
A05-SEQ1R.ab1(1>821)

GGATCCAACTCCTAAAAAAACCGCCACCATGGTGTT

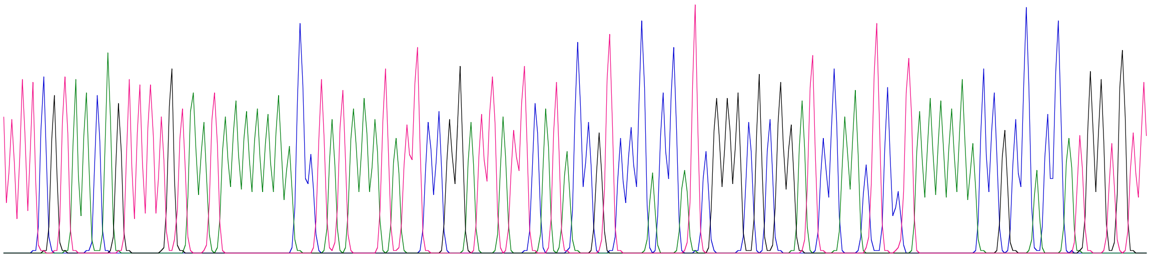

TTTCGTAACAGTTTTGTAATAAAAAAAACCTATAAATATTCCGGATTATTCATACCGTCCCACCATCGGGCGCGGATCCAACTCCTAAAAAAACCGCCACCATGGTGTT

A09-pFastbac-F.ab1(1>824)

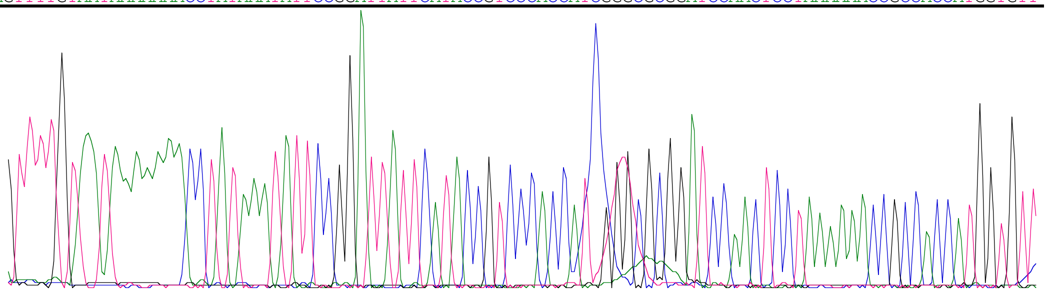

GTTTTGTaATAAAAAAAACCTATAAATATTCCGGATTATTCATACCGTCCCACCATCGGGCGCGGATCCAACTCCTAAAAAAACCGCCACCATGGTGTT

330 340 350 360 370 380 390 400 410 420

TCTGCCCCTCAAATGGTCCCTTGCAACCATGTCATTTCTACTTTCCTCACTGTTGGCTCTCTTAACTGTGTCCACTCCTTCATGGTGTCAGAGCACTGAAGCATCTC

U3823DH140-2.seq(1>2878)
A05-SEQ1R.ab1(1>821)

TCTGCCCCTCAAATGGTCCCTTGCAACCATGTCATTTCTACTTTCCTCACTGTTGGCTCTCTTAACTGTGTCCACTCCTTCATGGTGTCAGAGCACTGAAGCATCTC

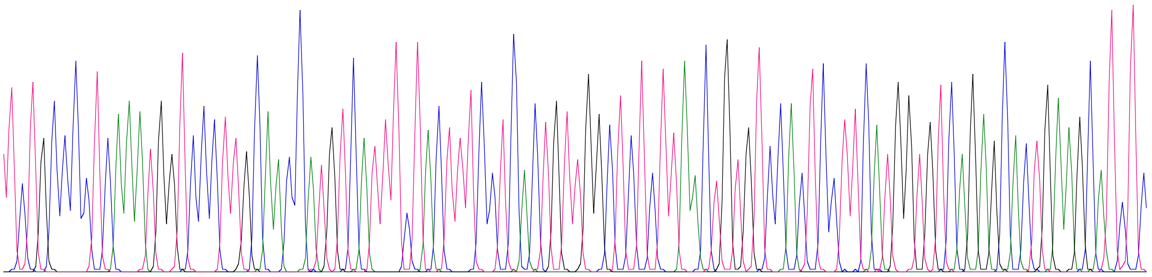

TCTGCCCCTCAAATGGTCCCTTGCAACCATGTCATTTCTACTTTCCTCACTGTTGGCTCTCTTAACTGTGTCCACTCCTTCATGGTGTCAGAGCACTGAAGCATCTC

330 340 350 360 370 380 390 400 410 420

TCTGCCCCTCAAATGGTCCCTTGCAACCATGTCATTTCTACTTTCCTCACTGTTGGCTCTCTTAACTGTGTCCACTCCTTCATGGTGTCAGAGCACTGAAGCATCTC

A09-pFastbac-F.ab1(1>824) →

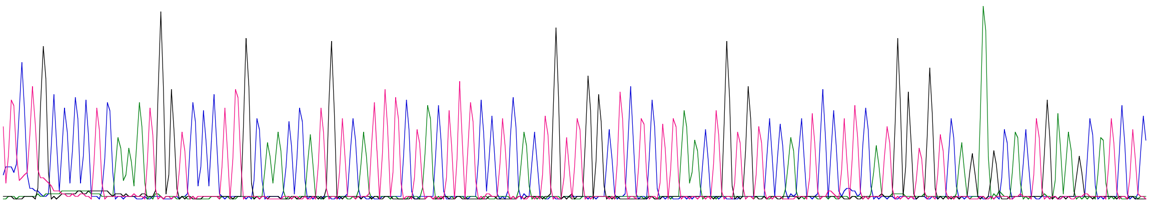

TCTGCCCCTCAAATGGTCCCTTGCAACCATGTCATTTCTACTTTCCTCACTGTTGGCTCTCTTAACTGTGTCCACTCCTTCATGGTGTCAGAGCACTGAAGCATCTC

430 440 450 460 470 480 490 500 510 520 530

CAAAACGTAGTGATGGGACACCATTTCCTTGGAATAAAATACGACTTCCTGAGTACGTCATCCCAGTTCATTATGATCTCTTGATCCATGCAAACCTTACCACGCTG

U3823DH140-2.seq(1>2878) → CAAAACGTAGTGATGGGACACCATTTCCTTGGAATAAAATACGACTTCCTGAGTACGTCATCCCAGTTCATTATGATCTCTTGATCCATGCAAACCTTACCACGCTG

A05-SEQ1R.ab1(1>821) ←

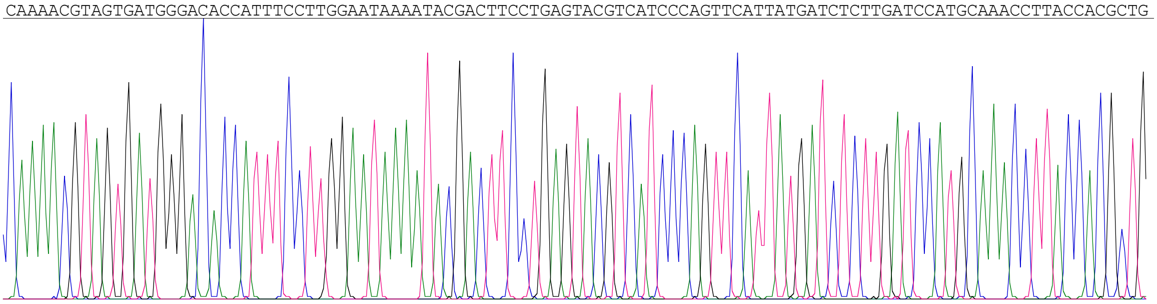

CAAAACGTAGTGATGGGACACCATTTCCTTGGAATAAAATACGACTTCCTGAGTACGTCATCCCAGTTCATTATGATCTCTTGATCCATGCAAACCTTACCACGCTG

A09-pFastbac-F.ab1(1>824) →

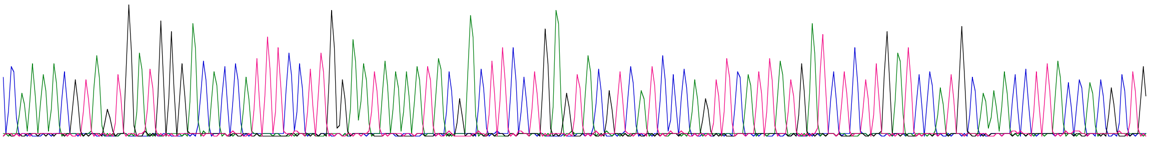

CAAAACGTAGTGATGGGACACCATTTCCTTGGAATAAAATACGACTTCCTGAGTACGTCATCCCAGTTCATTATGATCTCTTGATCCATGCAAACCTTACCACGCTG

540 550 560 570 580 590 600 610 620 630 640

ACCTTCTGGGGAACCACGAAAGTAGAAATCACAGCCAGTCAGCCCACCAGCACCATCATCCTGCATAGTCACCACCTGCAGATATCTAGGGCCACCCTCAGGAAGGG

U3823DH140-2.seq(1>2878) → ACCTTCTGGGGAACCACGAAAGTAGAAATCACAGCCAGTCAGCCCACCAGCACCATCATCCTGCATAGTCACCACCTGCAGATATCTAGGGCCACCCTCAGGAAGGG

A05-SEQ1R.ab1(1>821) ←

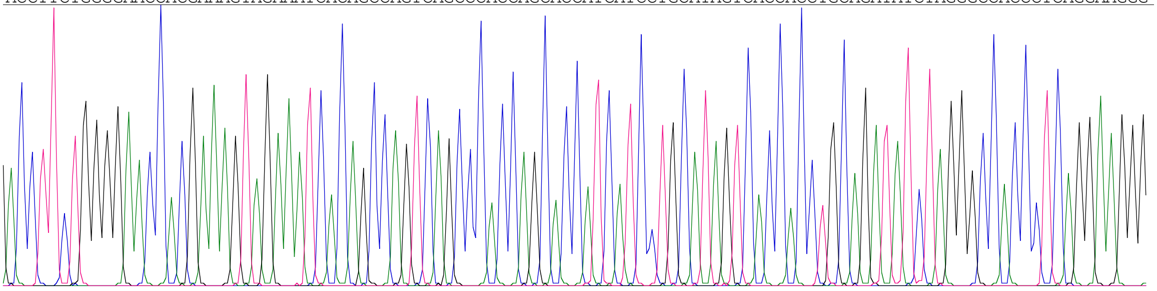

ACCTTCTGGGGAACCACGAAAGTAGAAATCACAGCCAGTCAGCCCACCAGCACCATCATCCTGCATAGTCACCACCTGCAGATATCTAGGGCCACCCTCAGGAAGGG

A09-pFastbac-F.ab1(1>824) →

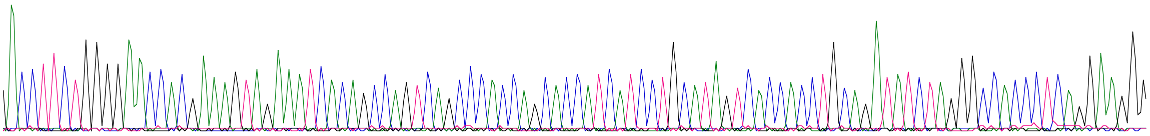

ACCTTCTGGGGAACCACGAAAGTAGAAATCACAGCCAGTCAGCCCACCAGCACCATCATCCTGCATAGTCACCACCTGCAGATATCTAGGGCCACCCTCAGGAAGGG

540 550 560 570 580 590 600 610 620 630 640

ACCTTCTGGGGAACCACGAAAGTAGAAATCACAGCCAGTCAGCCCACCAGCACCATCATCCTGCATAGTCACCACCTGCAGATATCTAGGGCCACCCTCAGGAAGGG

A06-SEQR.ab1(1>815)

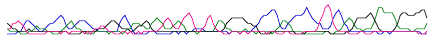

GTCACCACcTGCAGATATcTAGGGCCACCCTCAGGAAGGG

A11-SEQ1.ab1(1>821)

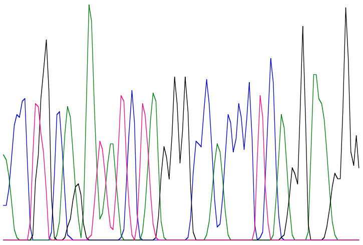

ACCTGCAGATATCTAGGGCCACCCTCAGGAAGGG

650 660 670 680 690 700 710 720 730 740

AGCTGGAGAGAGGCTATCGGAAGAACCCCTGCAGGTCCTGGAACACCCCCCGCAGGAGCAAATTGCACTGCTGGCTCCCGAGCCCCTCCTTGTCGGGCTCCCGTACA

U3823DH140-2.seq(1>2878)

AGCTGGAGAGAGGCTATCGGAAGAACCCCTGCAGGTCCTGGAACACCCCCCGCAGGAGCAAATTGCACTGCTGGCTCCCGAGCCCCTCCTTGTCGGGCTCCCGTACA

A05-SEQ1R.ab1(1>821)

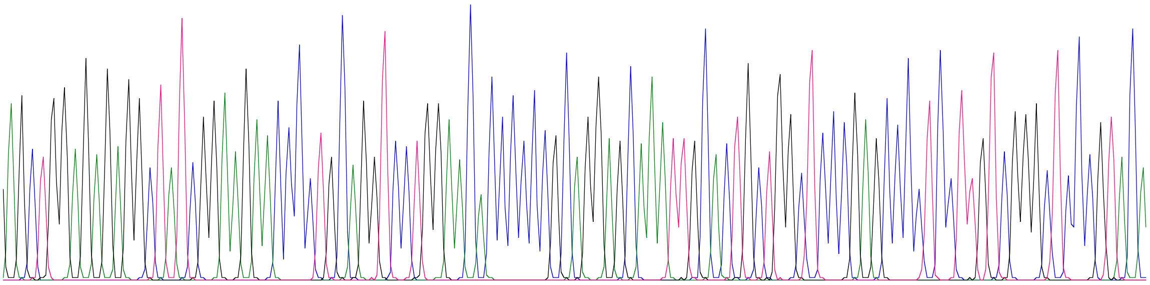

AGCTGGAGAGAGGCTATCGGAAGAACCCCTGCAGGTCCTGGAACACCCCCCGCAGGAGCAAATTGCACTGCTGGCTCCCGAGCCCCTCCTTGTCGGGCTCCCGTACA

A09-pFastbac-F.ab1(1>824)

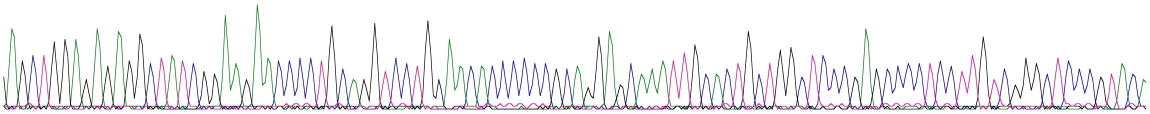

AGCTGGAGAGAGGCTATCGGAAGAACCCCTGCAGGTCCTGGAACACCCCCCGCAGGAGCAAATTGCACTGCTGGCTCCCGAGCCCCTCCTTGTCGGGCTCCCGTACA

A06-SEQR.ab1(1>815)

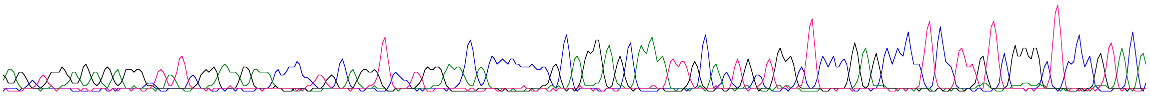

AGCTGGAGAGAGGCTATCGGAAGAACCCCTGCAGGTCCTGGAACACCCCCCGCAGGAGCAAATTGCACTGCTGGCTCCCGAGCCCCTCCTTgTCGgGCTCCCGTACA

A11-SEQ1.ab1(1>821)

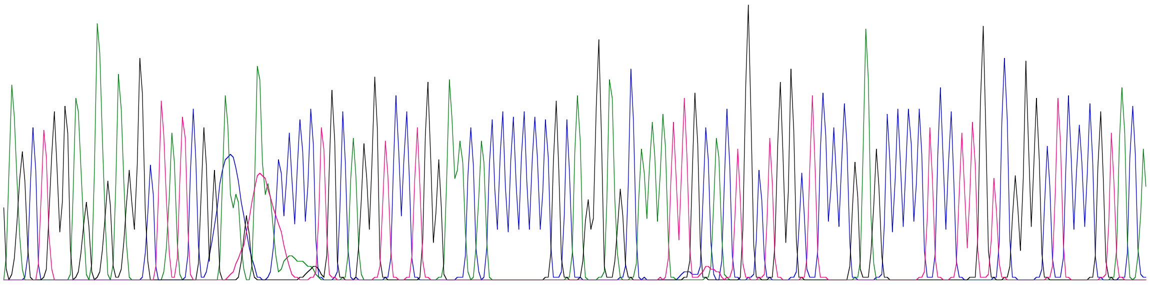

AGCTGGAGAGAGGCTATCGGAAGAACCCCTGCAGGTCCTGGAACACCCCCCGCAGGAGCAAATTGCACTGCTGGCTCCCGAGCCCCTCCTTGTCGGGCTCCCGTACA

750 760 770 780 790 800 810 820 830 840 850

CAGTTGTCATTCACTATGCTGGCAATCTTTCGGAGACTTTCCACGGATTTTACAAAAGCACCTACAGAACCAAGGAAGGGGAACTGAGGATACTAGCATCAACACAA

U3823DH140-2.seq(1>2878) → CAGTTGTCATTCACTATGCTGGCAATCTTTCGGAGACTTTCCACGGATTTTACAAAAGCACCTACAGAACCAAGGAAGGGGAACTGAGGATACTAGCATCAACACAA

A05-SEQ1R.ab1(1>821) ←

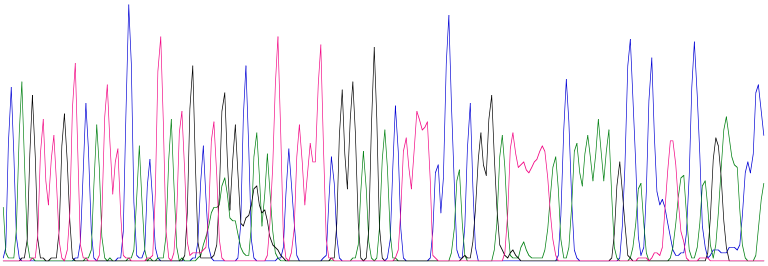

CAGTTGTCATTCACTATGCTGGCAATCTTTCGGAGACTTTCCACGGATTTTACAAAAGCACCTACAGAACCA

A09-pFastbac-F.ab1(1>824) →

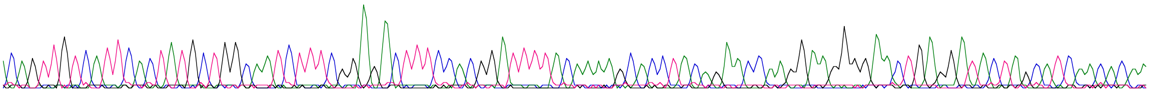

CAGTTGTCATTCACTATGCTGGCAATCTTTCGGAGACTTTCCACGGATTTTACAAAAGCACCTACAGAACCAAGGAAGGGGAACTGAGGATACTAGCATCAACACAA

A06-SEQR.ab1(1>815) ←

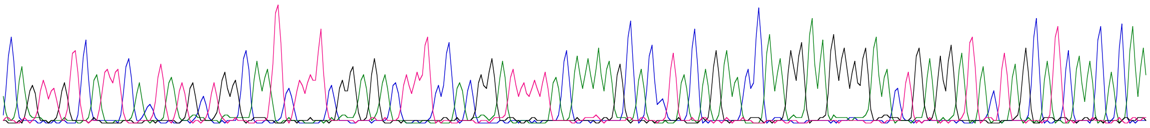

CAGTTGTCATTCACTATGCTGGCAATCTTTCGGAGACTTTCCACGGATTTTACAAAAGCACCTACAGAACCAAGGAAGGGGAACTGAGGATACTAGCATCAACACAA

A11-SEQ1.ab1(1>821) →

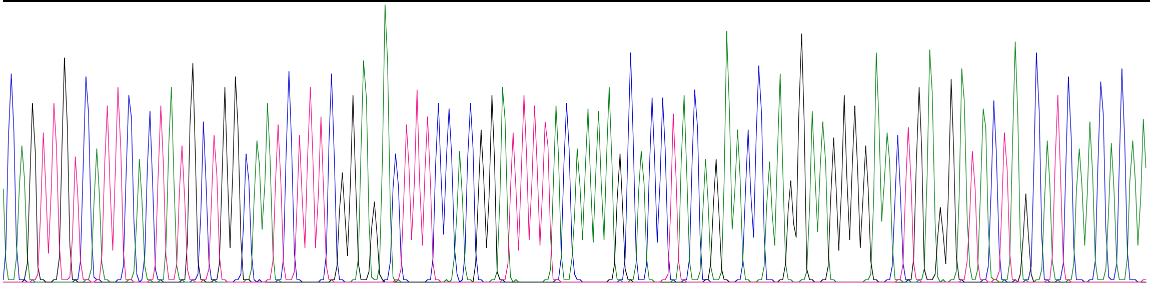

CAGTTGTCATTCACTATGCTGGCAATCTTTCGGAGACTTTCCACGGATTTTACAAAAGCACCTACAGAACCAAGGAAGGGGAACTGAGGATACTAGCATCAACACAA

860 870 880 890 900 910 920 930 940 950 960

TTTGAACCCACTGCAGCTAGAATGGCCTTTCCCTGCTTTGATGAACCTGCCTTCAAAGCAAGTTTCTCAATCAAAATTAGAAGAGAGCCAAGGCACCTAGCCATCTC

U3823DH140-2.seq(1>2878) → TTTGAACCCACTGCAGCTAGAATGGCCTTTCCCTGCTTTGATGAACCTGCCTTCAAAGCAAGTTTCTCAATCAAAATTAGAAGAGAGCCAAGGCACCTAGCCATCTC

A09-pFastbac-F.ab1(1>824) →

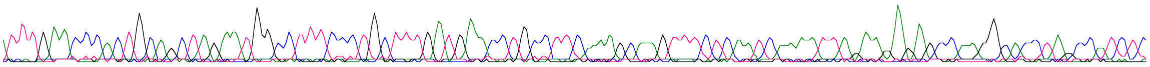

TTTGAACCCACTGCAGCTAGAATGGCCTTTCCCTGCTTTGATGAACCTGCCTTCAAAGCAAGTTTCTCAATCAAAATTAGAAGAGAGCCAAGGCACCTAGCCATCTC

A06-SEQR.ab1(1>815) ←

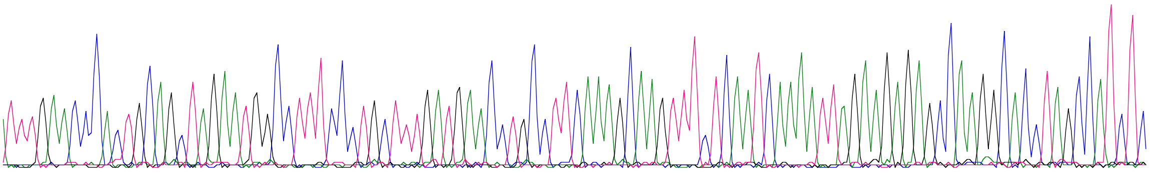

TTTGAACCCACTGCAGCTAGAATGGCCTTTCCCTGCTTTGATGAACCTGCCTTCAAAGCAAGTTTCTCAATCAAAATTAGAAGAGAGCCAAGGCACCTAGCCATCTC

860 870 880 890 900 910 920 930 940 950 960

TTTGAACCCACTGCAGCTAGAATGGCCTTTCCCTGCTTTGATGAACCTGCCTTCAAAGCAAGTTTCTCAATCAAAATTAGAAGAGAGCCAAGGCACCTAGCCATCTC

A11-SEQ1.ab1(1>821) →

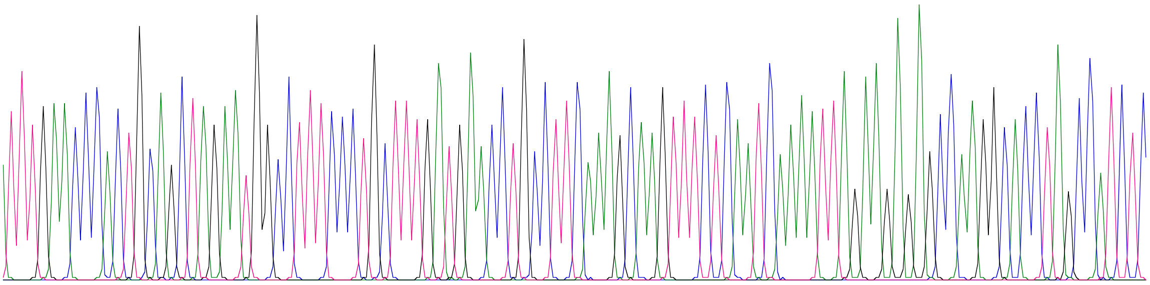

TTTGAACCCACTGCAGCTAGAATGGCCTTTCCCTGCTTTGATGAACCTGCCTTCAAAGCAAGTTTCTCAATCAAAATTAGAAGAGAGCCAAGGCACCTAGCCATCTC

970 980 990 1,000 1,010 1,020 1,030 1,040 1,050 1,060 1,070

CAATATGCCATTGGTGAAATCTGTGACTGTTGCTGAAGGACTCATAGAAGACCATTTTGATGTCACTGTGAAGATGAGCACCTATCTGGTGGCCTTCATCATTTCAG

U3823DH140-2.seq(1>2878) → CAATATGCCATTGGTGAAATCTGTGACTGTTGCTGAAGGACTCATAGAAGACCATTTTGATGTCACTGTGAAGATGAGCACCTATCTGGTGGCCTTCATCATTTCAG

A09-pFastbac-F.ab1(1>824) →

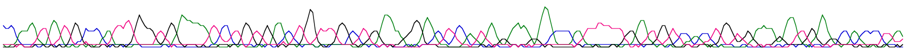

CAATATGCCATTGGTGAAATCTGTGACTGTTGCTGAAGGACTCATAGAAGACCATTTTGATGTCACTGTGAAGATGAGCACCTAT

A06-SEQR.ab1(1>815) ←

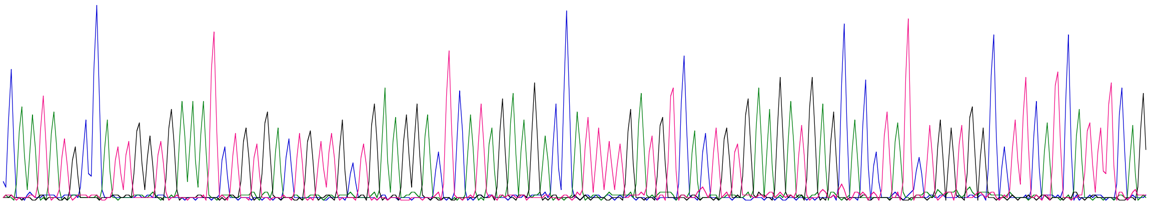

CAATATGCCATTGGTGAAATCTGTGACTGTTGCTGAAGGACTCATAGAAGACCATTTTGATGTCACTGTGAAGATGAGCACCTATCTGGTGGCCTTCATCATTTCAG

A11-SEQ1.ab1(1>821) →

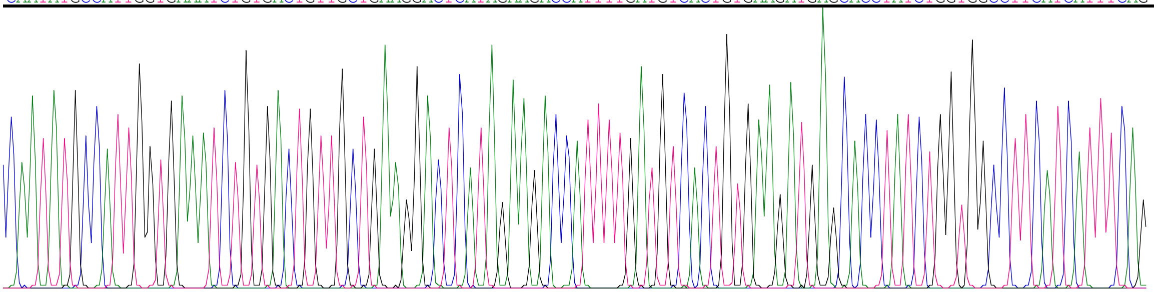

CAATATGCCATTGGTGAAATCTGTGACTGTTGCTGAAGGACTCATAGAAGACCATTTTGATGTCACTGTGAAGATGAGCACCTATCTGGTGGCCTTCATCATTTCAG

1,080 1,090 1,100 1,110 1,120 1,130 1,140 1,150 1,160 1,170

ATTTTGAGTCTGTCAGCAAGATAACCAAGAGTGGAGTCAAGGTTTCTGTTTATGCTGTGCCAGACAAGATAAATCAAGCAGATTATGCACTGGATGCTGCGGTGACT

U3823DH140-2.seq(1>2878) → ATTTTGAGTCTGTCAGCAAGATAACCAAGAGTGGAGTCAAGGTTTCTGTTTATGCTGTGCCAGACAAGATAAATCAAGCAGATTATGCACTGGATGCTGCGGTGACT

A06-SEQR.ab1(1>815) ←

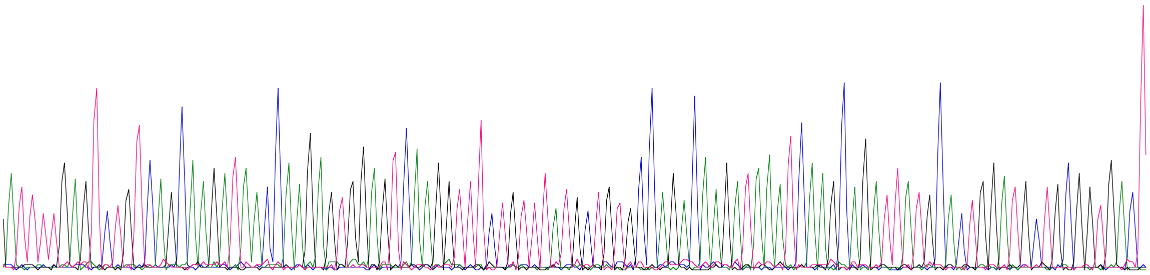

ATTTTGAGTCTGTCAGCAAGATAACCAAGAGTGGAGTCAAGGTTTCTGTTTATGCTGTGCCAGACAAGATAAATCAAGCAGATTATGCACTGGATGCTGCGGTGACT

1,080 1,090 1,100 1,110 1,120 1,130 1,140 1,150 1,160 1,170

ATTTTGAGTCTGTCAGCAAGATAACCAAGAGTGGAGTCAAGGTTTCTGTTTATGCTGTGCCAGACAAGATAAATCAAGCAGATTATGCACTGGATGCTGCGGTGACT

A11-SEQ1.ab1(1>821) →

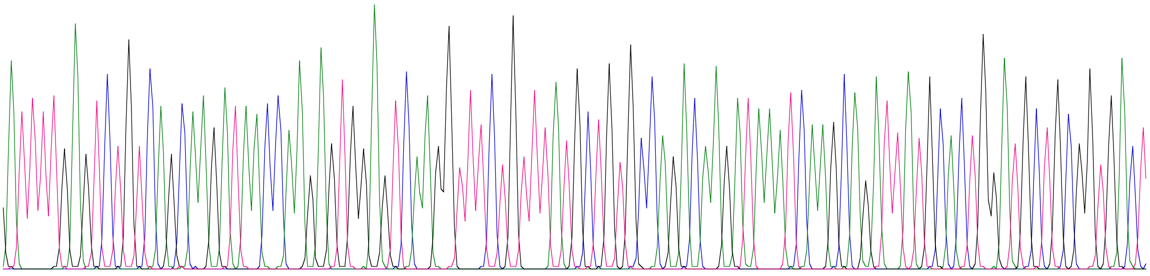

ATTTTGAGTCTGTCAGCAAGATAACCAAGAGTGGAGTCAAGGTTTCTGTTTATGCTGTGCCAGACAAGATAAATCAAGCAGATTATGCACTGGATGCTGCGGTGACT

1,180 1,190 1,200 1,210 1,220 1,230 1,240 1,250 1,260 1,270 1,280

CTTCTAGAATTTTATGAGGATTATTTCAGCATACCGTATCCCCTACCCAAACAAGATCTTGCTGCTATTCCCGACTTTCAGTCTGGTGCTATGGAAAACTGGGGACT

U3823DH140-2.seq(1>2878) → CTTCTAGAATTTTATGAGGATTATTTCAGCATACCGTATCCCCTACCCAAACAAGATCTTGCTGCTATTCCCGACTTTCAGTCTGGTGCTATGGAAAACTGGGGACT

A06-SEQR.ab1(1>815) ←

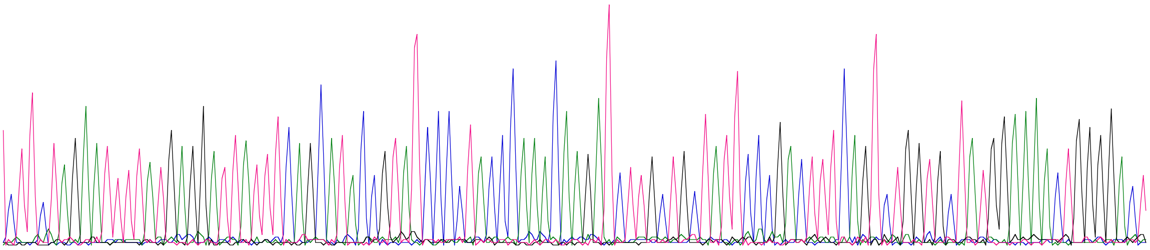

CTTCTAGAATTTTATGAGGATTATTTCAGCATACCGTATCCCCTACCCAAACAAGATCTTGCTGCTATTCCCGACTTTCAGTCTGGTGCTATGGAAAACTGGGGACT

A11-SEQ1.ab1(1>821) →

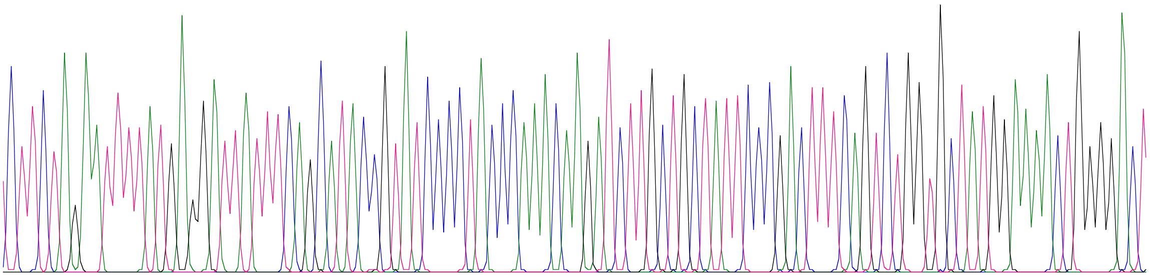

CTTCTAGAATTTTATGAGGATTATTTCAGCATACCGTATCCCCTACCCAAACAAGATCTTGCTGCTATTCCCGACTTTCAGTCTGGTGCTATGGAAAACTGGGGACT

A07-SEQ3R.ab1(1>810) ←

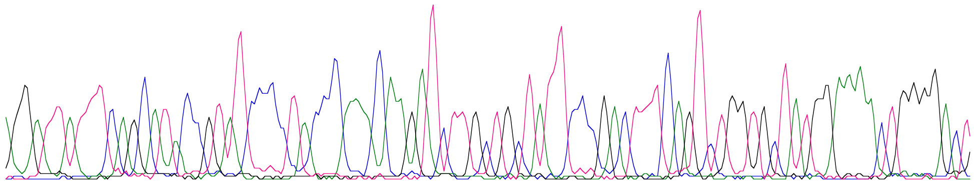

AGGATTATTTCAGCATACCGTATCCCCTACCCAAACAAGATCTTGCTGCTATTCCCGACTTTCAGTCTGGTGCTATGGAAAACTGGGGACT

A12-SEQ2.ab1(1>821) →

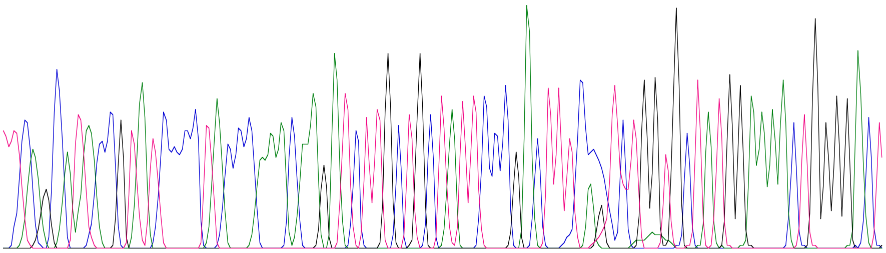

TTCAGCATACCGTATCCCCTACCCAAACAAGATCTTGCTGCTATTCCCGACTTTCAGTCTGGTGCTATGGAAAACTGGGGACT

M349V

1,290 1,300 1,310 1,320 1,330 1,340 1,350 1,360 1,370 1,380 1,390

GACAACATATAGAGAATCTGCTCTGTTGTTTGATGCAGAAAAGTCTTCTGCATCAAGTAAGCTTGGCATCACAGTGACTGTGGCCCATGAACTGGCTCACCAGTGGT

U3823DH140-2.seq(1>2878) GACAACATATAGAGAATCTGCTCTGTTGTTTGATGCAGAAAAGTCTTCTGCATCAAGTAAGCTTGGCATCACAGTGACTGTGGCCCATGAACTGGCTCACCAGTGGT
A06-SEQR.ab1(1>815)

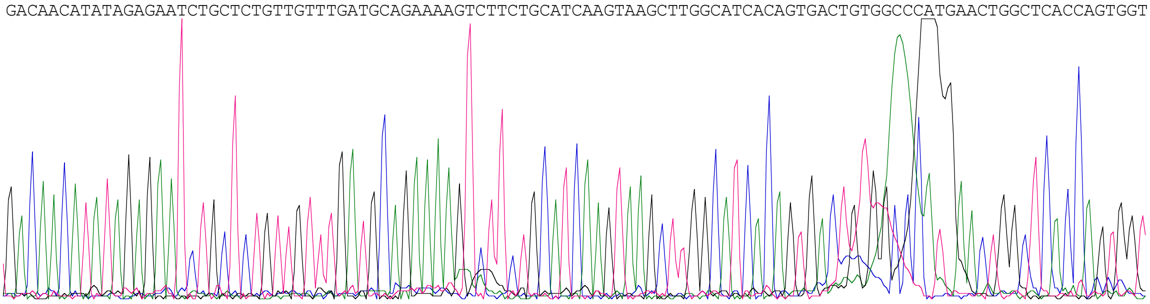

GACAACATATAGAGAATCTGCTCTGTTGTTTGATGCAGAAAAGTcTTCTGCATCAAGTAAGCTTGGCATCACAGTGACTgTggcCCATGAACTGGCTCACCAgtGGT

A11-SEQ1.ab1(1>821)

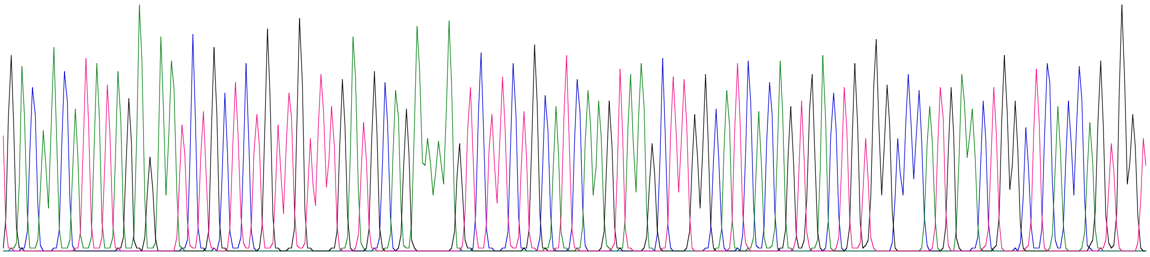

GACAACATATAGAGAATCTGCTCTGTTGTTTGATGCAGAAAAGTCTTCTGCATCAAGTAAGCTTGGCATCACAGTGACTGTGGCCCATGAACTGGCTCACCAGTGGT

A07-SEQ3R.ab1(1>810)

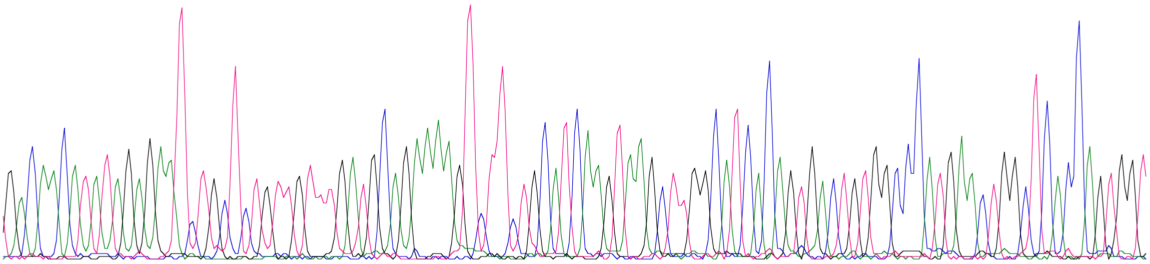

GACAACATATAGAGAATCTGCTCTGTTGTTTGATGCAGAAAAGTCTTCTGCATCAAGTAAGCTTGGCATCACAGTGACTGTGGCCCATGAACTGGCTCACCAGTGGT

A12-SEQ2.ab1(1>821)

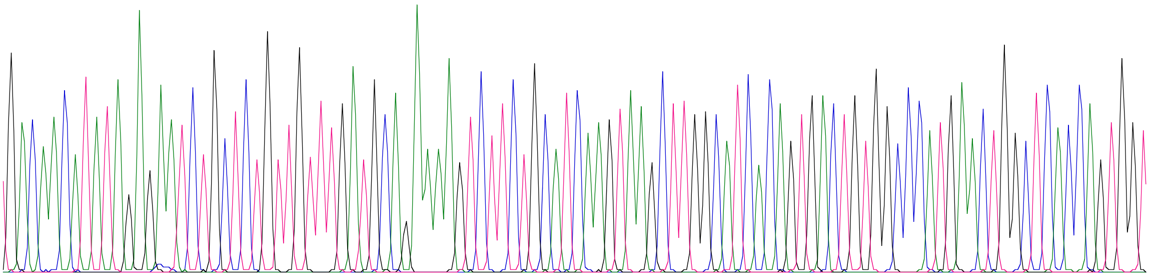

GACAACATATAGAGAATCTGCTCTGTTGTTTGATGCAGAAAAGTCTTCTGCATCAAGTAAGCTTGGCATCACAGTGACTGTGGCCCATGAACTGGCTCACCAGTGGT

1,400 1,410 1,420 1,430 1,440 1,450 1,460 1,470 1,480 1,490

TTGGGAACCTGGTCACTATGGAATGGTGGAATGATCTTTGGCTAAATGAAGGATTTGCCAAATTTATGGAGTTTGTGTCTGTCAGTGTGACCCATCCTGAACTGAAA

U3823DH140-2.seq(1>2878) TTGGGAACCTGGTCACTATGGAATGGTGGAATGATCTTTGGCTAAATGAAGGATTTGCCAAATTTATGGAGTTTGTGTCTGTCAGTGTGACCCATCCTGAACTGAAA
A06-SEQR.ab1(1>815)

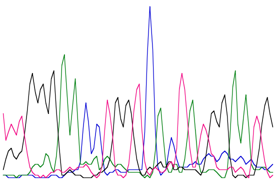

tTGGGAACctGGTCACTATGGAATGG

A11-SEQ1.ab1(1>821)

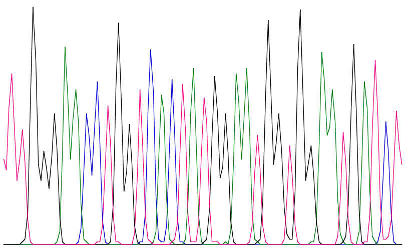

TTGGGAACCTGGTCACTATGGAATGGTGGAATGATCTT

1,400 1,410 1,420 1,430 1,440 1,450 1,460 1,470 1,480 1,490

TTGGGAACCTGGTCACTATGGAATGGTGGAATGATCTTTGGCTAAATGAAGGATTTGCCAAATTTATGGAGTTTGTGTCTGTCAGTGTGACCCATCCTGAACTGAAA

A07-SEQ3R.ab1(1>810) ←

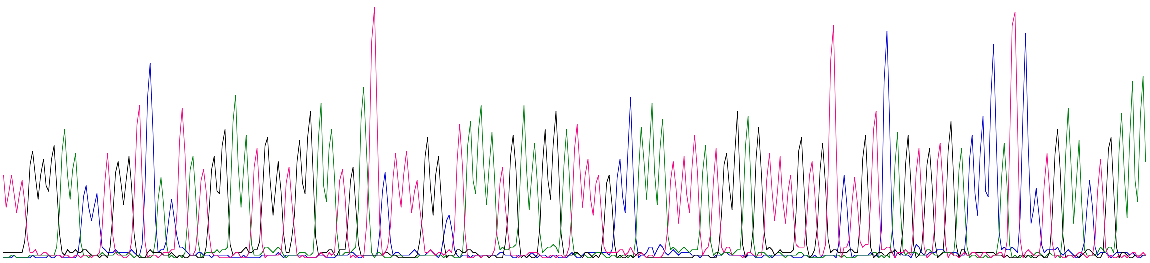

TTGGGAACCTGGTCACTATGGAATGGTGGAATGATCTTTGGCTAAATGAAGGATTTGCCAAATTTATGGAGTTTGTGTCTGTCAGTGTGACCCATCCTGAACTGAAA

A12-SEQ2.ab1(1>821) →

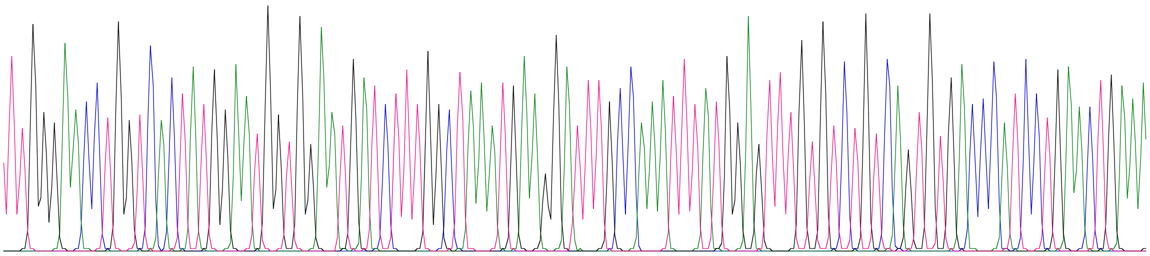

TTGGGAACCTGGTCACTATGGAATGGTGGAATGATCTTTGGCTAAATGAAGGATTTGCCAAATTTATGGAGTTTGTGTCTGTCAGTGTGACCCATCCTGAACTGAAA

1,500 1,510 1,520 1,530 1,540 1,550 1,560 1,570 1,580 1,590 1,600

GTTGGAGATTATTTCTTTGGCAAATGTTTTGACGCAATGGAGGTAGATGCTTTAAATTCCTCACACCCTGTGTCTACACCTGTGGAAAATCCTGCTCAGATCCGGGA

U3823DH140-2.seq(1>2878) → GTTGGAGATTATTTCTTTGGCAAATGTTTTGACGCAATGGAGGTAGATGCTTTAAATTCCTCACACCCTGTGTCTACACCTGTGGAAAATCCTGCTCAGATCCGGGA

A07-SEQ3R.ab1(1>810) ←

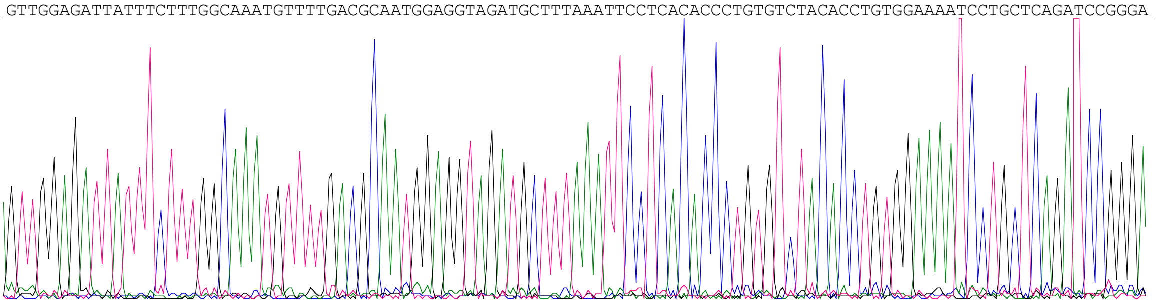

GTTGGAGATTATTTCTTTGGCAAATGTTTTGACGCAATGGAGGTAGATGCTTTAAATTCCTCACACCCTGTGTCTACACCTGTGGAAAATCCTGCTCAGATCCGGGA

A12-SEQ2.ab1(1>821) →

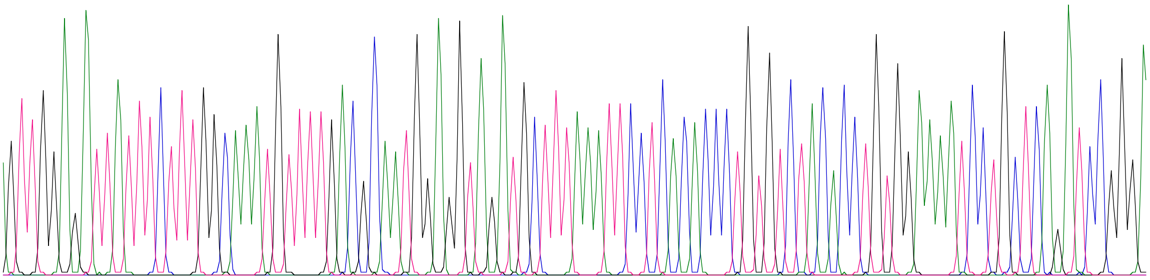

GTTGGAGATTATTTCTTTGGCAAATGTTTTGACGCAATGGAGGTAGATGCTTTAAATTCCTCACACCCTGTGTCTACACCTGTGGAAAATCCTGCTCAGATCCGGGA

1,610 1,620 1,630 1,640 1,650 1,660 1,670 1,680 1,690 1,700 1,710

GATGTTTGATGATGTTTCTTATGATAAGGGAGCTTGTATTCTGAATATGCTAAGGGAGTATCTTAGTGCTGACGCATTTAAAAGTGGTATTGTACAGTATCTCCAGA

U3823DH140-2.seq(1>2878) → GATGTTTGATGATGTTTCTTATGATAAGGGAGCTTGTATTCTGAATATGCTAAGGGAGTATCTTAGTGCTGACGCATTTAAAAGTGGTATTGTACAGTATCTCCAGA

A07-SEQ3R.ab1(1>810) ←

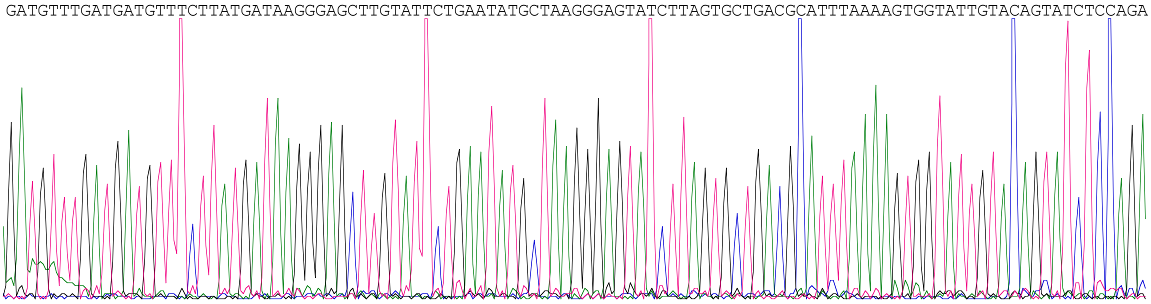

GAtGTTTGATGATGTTTCTTATGATAAGGGAGCTTGTATTCTGAATATGCTAAGGGAGTATCTTAGTGCTGACGCATTTAAAAGTGGTATTGTACAGTATCTCCAGA

1,610 1,620 1,630 1,640 1,650 1,660 1,670 1,680 1,690 1,700 1,710

GATGTTTGATGATGTTTCTTATGATAAGGGAGCTTGTATTCTGAATATGCTAAGGGAGTATCTTAGTGCTGACGCATTTAAAAGTGGTATTGTACAGTATCTCCAGA

A12-SEQ2.ab1(1>821)

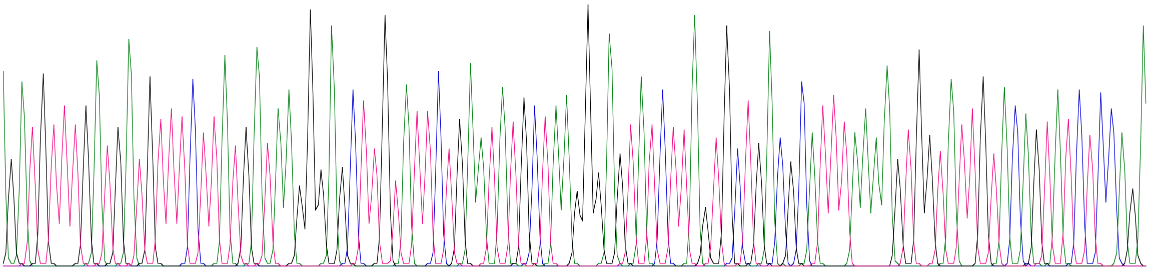

GATGTTTGATGATGTTTCTTATGATAAGGGAGCTTGTATTCTGAATATGCTAAGGGAGTATCTTAGTGCTGACGCATTTAAAAGTGGTATTGTACAGTATCTCCAGA

1,720 1,730 1,740 1,750 1,760 1,770 1,780 1,790 1,800 1,810

AGCATAGCTATAAAAATACAAAAAACGAGGACCTGTGGGATAGTATGGCAAGTATTTGCCCTACAGATGGTGTAAAAGGGATGGATGGCTTTTGCTCTAGAAGTCAA

U3823DH140-2.seq(1>2878)

AGCATAGCTATAAAAATACAAAAAACGAGGACCTGTGGGATAGTATGGCAAGTATTTGCCCTACAGATGGTGTAAAAGGGATGGATGGCTTTTGCTCTAGAAGTCAA

A07-SEQ3R.ab1(1>810)

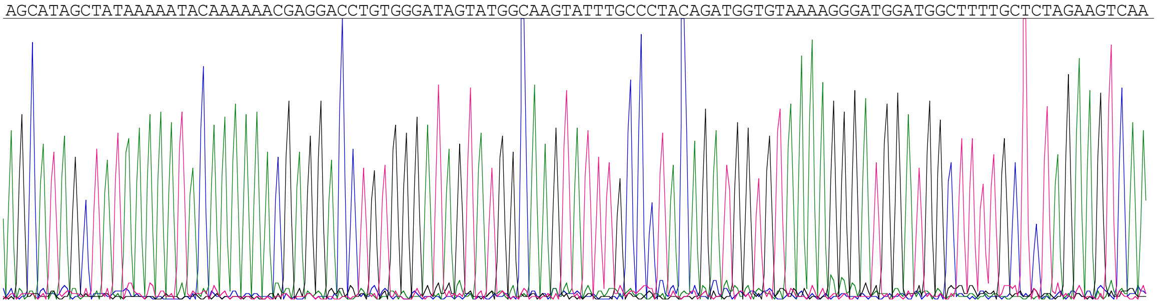

AGCATAGCTATAAAAATACAAAAAACGAGGACCTGTGGGATAGTATGGCAAGTATTTGCCCTACAGAtGGTGTAAAAGGGATGGATGGCTTTTGCTCTAGAAGTCAA

A12-SEQ2.ab1(1>821)

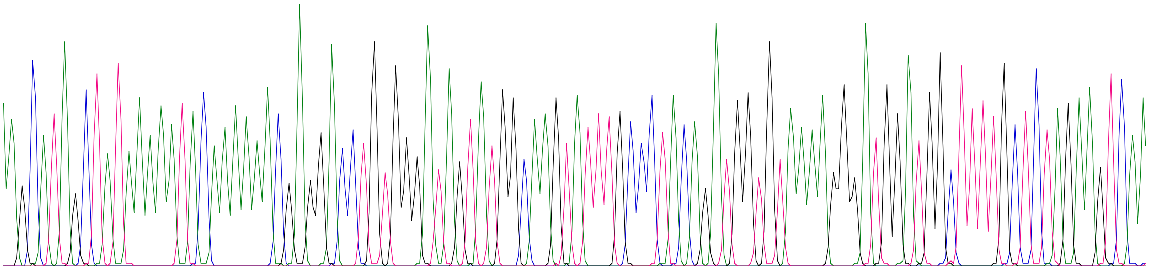

AGCATAGCTATAAAAATACAAAAAACGAGGACCTGTGGGATAGTATGGCAAGTATTTGCCCTACAGATGGTGTAAAAGGGATGGATGGCTTTTGCTCTAGAAGTCAA

B07-SEQ4RN.ab1(1>821)

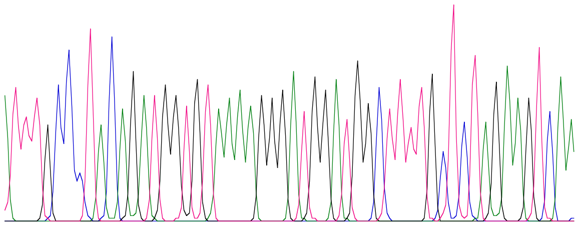

ATTTGCCCTACAGATGGTGTAAAAGGGATGGATGGCTTTTGCTCTAGAAGTCAA

B01-SEQ3.ab1(1>821)

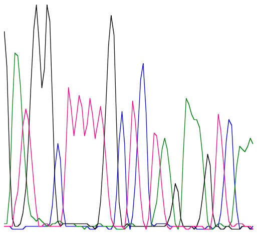

GATGGCTTTTGCTCTAGAAGTCAA

K528R

1,820 1,830 1,840 1,850 1,860 1,870 1,880 1,890 1,900 1,910 1,920

CATTCATCTTCATCCTCACATTGGCATCAGGAAGGGGTGGATGTGAAAACCATGATGAACACTTGGACACTGCAGCGTGGTTTTCCCCTAATAACCATCACAGTGAG

U3823DH140-2.seq(1>2878) → CATTCATCTTCATCCTCACATTGGCATCAGGAAGGGGTGGATGTGAAAACCATGATGAACACTTGGACACTGCAGCGTGGTTTTCCCCTAATAACCATCACAGTGAG

A07-SEQ3R.ab1(1>810) ←

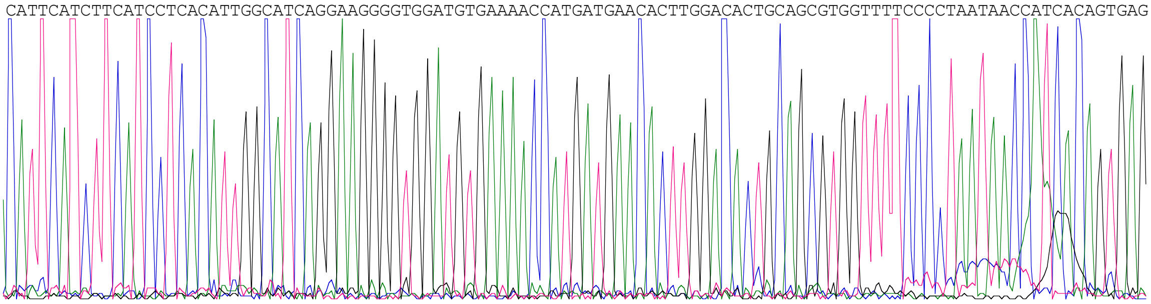

CATTCATCTTCATCCTCACATTGGCATCAGGAAGGGGTGGATGTGAAAACCATGATGAACACTTGGACACTGCAGCGTGGTTTTCCCCTAATAACCATCACAGTGAG

A12-SEQ2.ab1(1>821) →

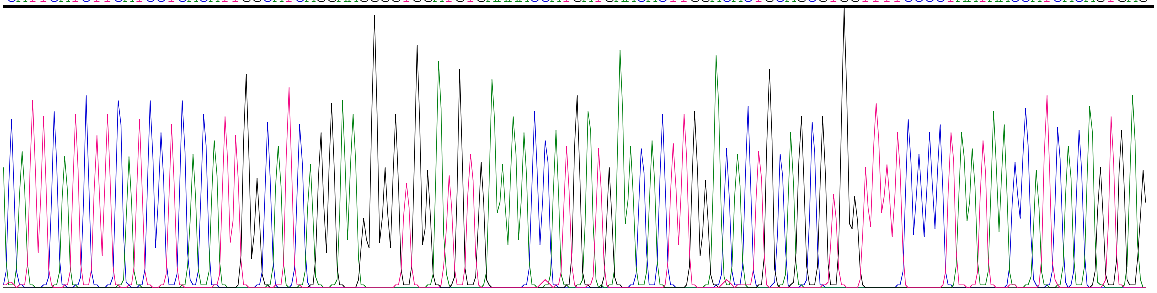

CATTCATCTTCATCCTCACATTGGCATCAGGAAGGGGTGGATGTGAAAACCATGATGAACACTTGGACACTGCAGCGTGGTTTTCCCCTAATAACCATCACAGTGAG

B07-SEQ4RN.ab1(1>821) ←

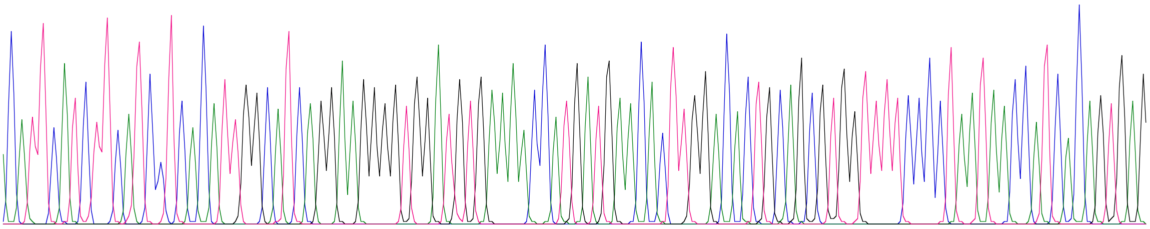

CATTCATCTTCATCCTCACATTGGCATCAGGAAGGGGTGGATGTGAAAACCATGATGAACACTTGGACACTGCAGCGTGGTTTTCCCCTAATAACCATCACAGTGAG

B01-SEQ3.ab1(1>821) →

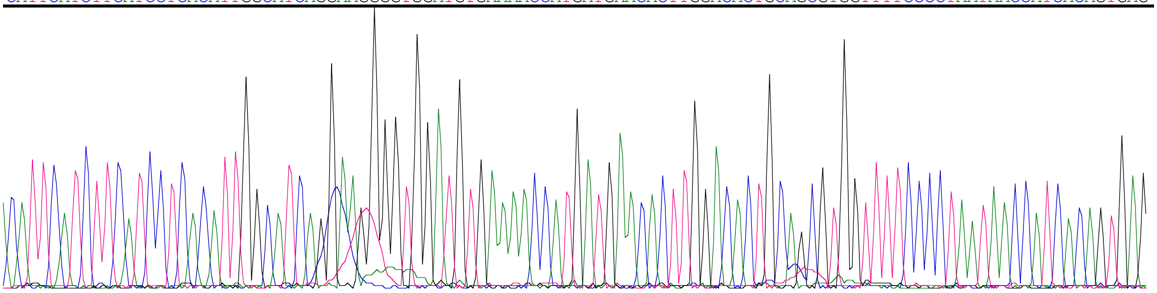

CATTCATCTTCATCCTCACATTGGCATCAGGAAgGGGTGGATGTGAAAACCATGATGAACACTTGGACACTGCAgCGTGGTTTTCCCCTAATAACCATCACAGTGAG

1,930 1,940 1,950 1,960 1,970 1,980 1,990 2,000 2,010 2,020 2,030

GGGGAGGAATGTACACATGAAGCAAGAGCACTACATGAAGGGCTCTGACGGCGCCCCGGACACTGGGTACCTGTGGCATGTTCCATTGACATTCATCACCAGCAAAT

U3823DH140-2.seq(1>2878) → GGGGAGGAATGTACACATGAAGCAAGAGCACTACATGAAGGGCTCTGACGGCGCCCCGGACACTGGGTACCTGTGGCATGTTCCATTGACATTCATCACCAGCAAAT

A07-SEQ3R.ab1(1>810) ←

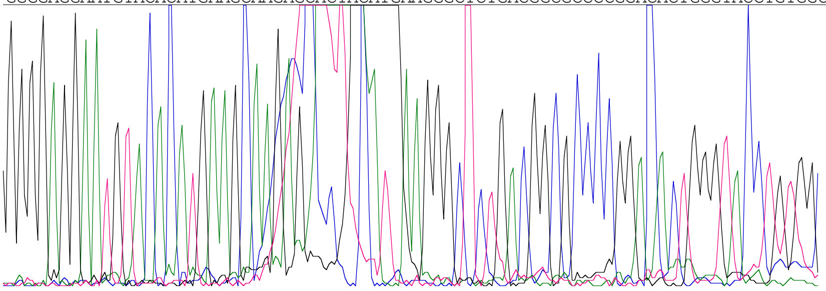

GGGGAGGAATGTACACATGAAGCAAGAGCaCTaCATGAAGGGCTCTGACGGCGCCCCGGACACTGGGTACCTGTGGC

A12-SEQ2.ab1(1>821) →

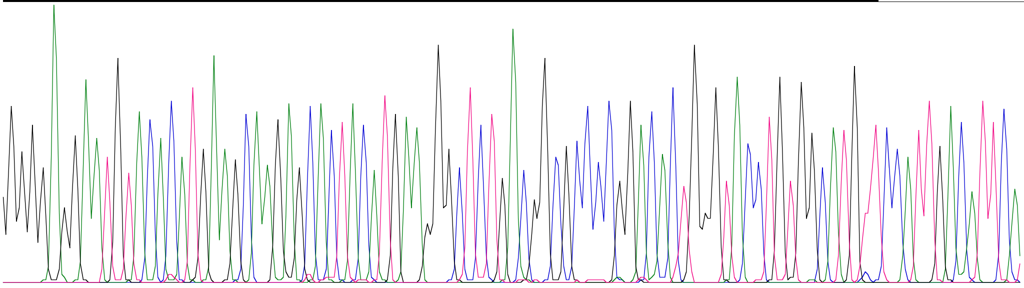

GGGGAGGAATGTACACATGAAGCAAGAGCACTACATGAAGGGCTCTGACGGCGCCCCGGACACTGGGTACCTGTGGCATGTTCCATTGACATTCAT

1,930 1,940 1,950 1,960 1,970 1,980 1,990 2,000 2,010 2,020 2,030

GGGGAGGAATGTACACATGAAGCAAGAGCACTACATGAAGGGCTCTGACGGCGCCCCGGACACTGGGTACCTGTGGCATGTTCCATTGACATTCATCACCAGCAAAT

B07-SEQ4RN.ab1(1>821)

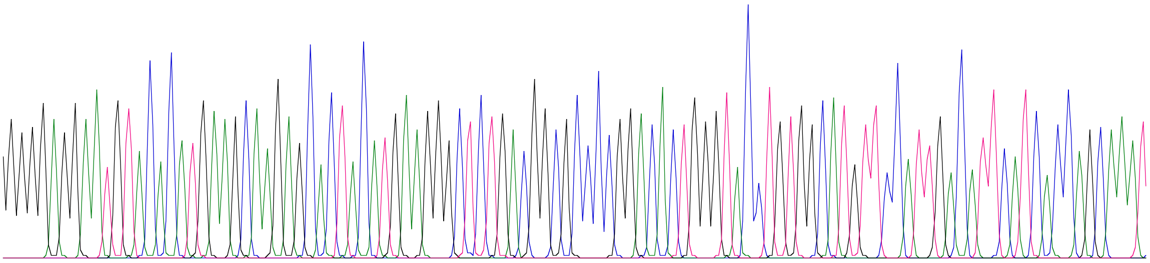

GGGGAGGAATGTACACATGAAGCAAGAGCACTACATGAAGGGCTCTGACGGCGCCCCGGACACTGGGTACCTGTGGCATGTTCCATTGACATTCATCACCAGCAAAT

B01-SEQ3.ab1(1>821)

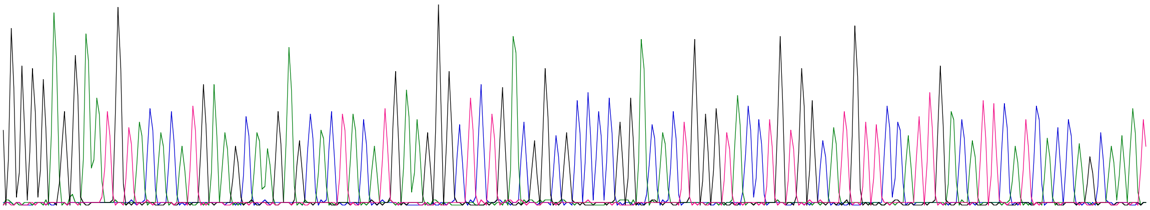

GGGGAGGAATGTACACATGAAGCAAGAGCACTACATGAAGGGCTCTGACGGCGCCCCGGACACTGGGTACCTGTGGCATGTTCCATTGACATTCATCACCAGCAAAT

D575N

2,040 2,050 2,060 2,070 2,080 2,090 2,100 2,110 2,120 2,130 2,140

CCAACATGGTCCATCGATTTTTGCTAAAAACAAAAACAGATGTGCTCATCCTCCCAGAAGAGGTGGAATGGATCAAATTTAATGTGGGCATGAATGGCTATTACATT

U3823DH140-2.seq(1>2878) CCAACATGGTCCATCGATTTTTGCTAAAAACAAAAACAGATGTGCTCATCCTCCCAGAAGAGGTGGAATGGATCAAATTTAATGTGGGCATGAATGGCTATTACATT

B07-SEQ4RN.ab1(1>821)

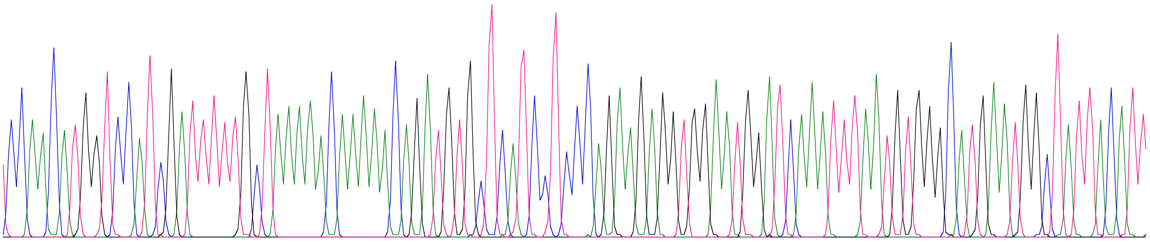

CCAACATGGTCCATCGATTTTTGCTAAAAACAAAAACAGATGTGCTCATCCTCCCAGAAGAGGTGGAATGGATCAAATTTAATGTGGGCATGAATGGCTATTACATT

B01-SEQ3.ab1(1>821)

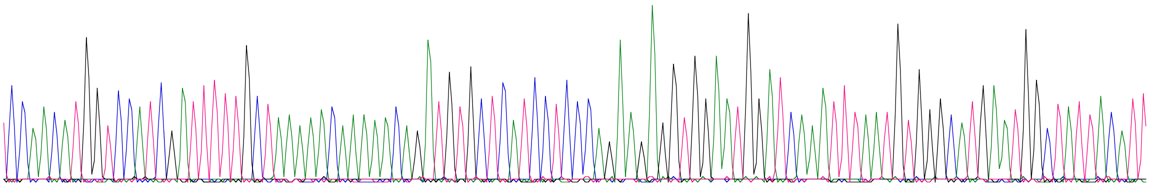

CCAACATGGTCCATCGATTTTTGCTAAAAACAAAAACAGATGTGCTCATCCTCCCAGAAGAGGTGGAATGGATCAAATTTAATGTGGGCATGAATGGCTATTACATT

2,150 2,160 2,170 2,180 2,190 2,200 2,210 2,220 2,230 2,240

GTGCATTACGAGGATGATGGATGGGACTCTTTGACTGGCCTTTTAAAAGGAACACACACAGCAGTCAGCAGTAATGATCGGGCGAGTCTCATTAACAATGCATTTCA

U3823DH140-2.seq(1>2878) GTGCATTACGAGGATGATGGATGGGACTCTTTGACTGGCCTTTTAAAAGGAACACACACAGCAGTCAGCAGTAATGATCGGGCGAGTCTCATTAACAATGCATTTCA

B07-SEQ4RN.ab1(1>821)

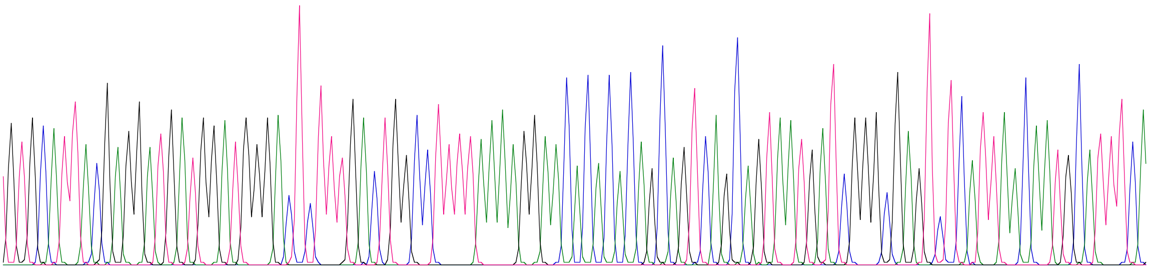

GTGCATTACGAGGATGATGGATGGGACTCTTTGACTGGCCTTTTAAAAGGAACACACACAGCAGTCAGCAGTAATGATCGGGCGAGTCTCATTAACAATGCATTTCA

2,150 2,160 2,170 2,180 2,190 2,200 2,210 2,220 2,230 2,240

GTGCATTACGAGGATGATGGATGGGACTCTTTGACTGGCCTTTTAAAAGGAACACACACAGCAGTCAGCAGTAATGATCGGGCGAGTCTCATTAACAATGCATTTCA

B01-SEQ3.ab1(1>821)

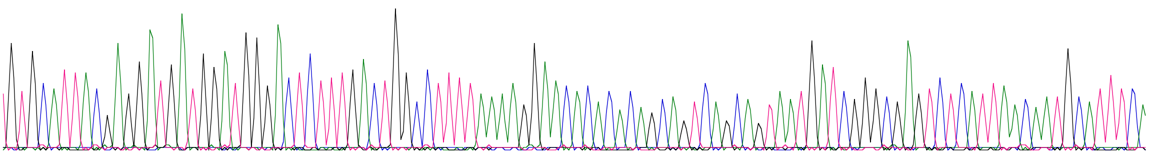

GTGCATTACGAGGATGATGGATGGGACTCTTTGACTGGCCTTTTAAAAGGAACACACACAGCAGTCAGCAGTAATGATCGGGCGAGTCTCATTAACAATGCATTTCA

2,250 2,260 2,270 2,280 2,290 2,300 2,310 2,320 2,330 2,340 2,350

GCTCGTCAGCATTGGGAAGCTGTCCATTGAAAAGGCCTTGGATTTATCCCTGTACTTGAAACATGAAACTGAAATTATGCCCGTGTTTCAAGGTTTGAATGAGCTGA

U3823DH140-2.seq(1>2878) GCTCGTCAGCATTGGGAAGCTGTCCATTGAAAAGGCCTTGGATTTATCCCTGTACTTGAAACATGAAACTGAAATTATGCCCGTGTTTCAAGGTTTGAATGAGCTGA

B07-SEQ4RN.ab1(1>821)

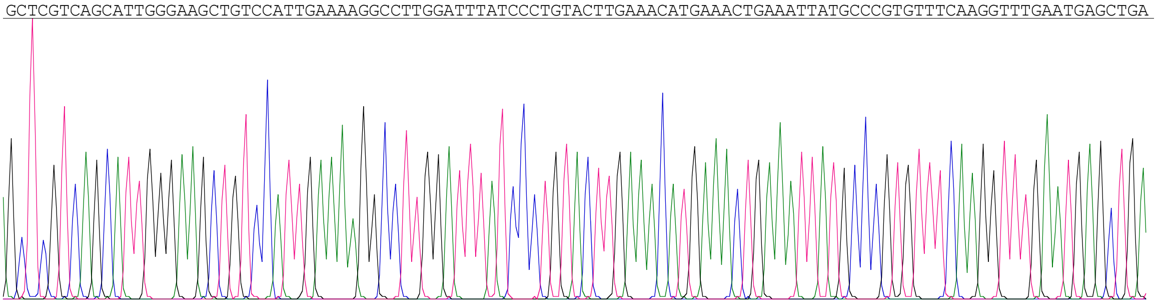

GCTCGTCAGCATTGGGAAGCTGTCCATTGAAAAGGCCTTGGATTTATCCCTGTACTTGAAACATGAAACTGAAATTATGCCCGTGTTTCAAGGTTTGAATGAGCTGA

B01-SEQ3.ab1(1>821)

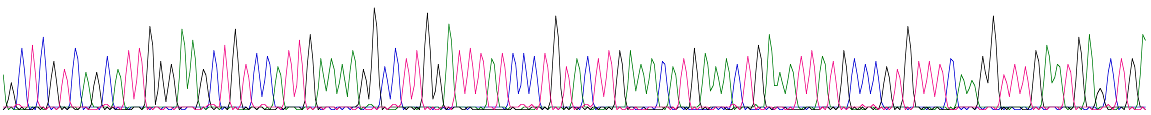

GCTCGTCAGCATTGGGAAGCTGTCCATTGAAAAGGCCTTGGATTTATCCCTGTACTTGAAACATGAAACTGAAATTATGCCCGTGTTTCAAGGTTTGAATGAGCTGA

2,360 2,370 2,380 2,390 2,400 2,410 2,420 2,430 2,440 2,450 2,460

TTCCTATGTATAAGTTAATGGAGAAAAGAGATATGAATGAAGTGGAAACTCAATTCAAGGCCTTCCTCATCAGGCTGCTAAGGGACCTCATTGATAAGCAGACATGG

U3823DH140-2.seq(1>2878) TTCCTATGTATAAGTTAATGGAGAAAAGAGATATGAATGAAGTGGAAACTCAATTCAAGGCCTTCCTCATCAGGCTGCTAAGGGACCTCATTGATAAGCAGACATGG

B07-SEQ4RN.ab1(1>821)

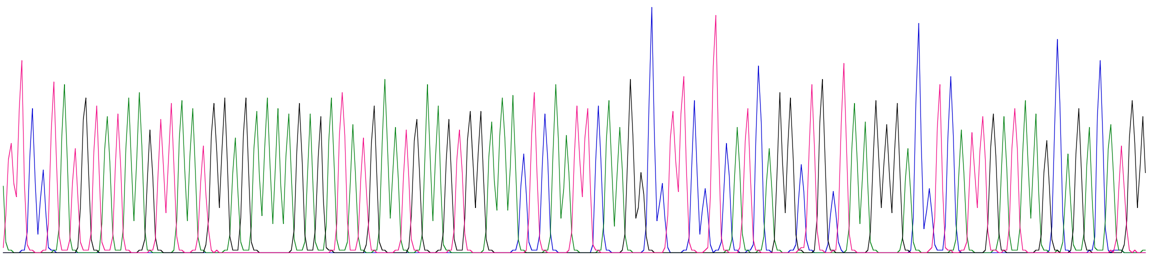

TTCCTATGTATAAGTTAATGGAGAAAAGAGATATGAATGAAGTGGAAACTCAATTCAAGGCCTTCCTCATCAGGCTGCTAAGGGACCTCATTGATAAGCAGACATGG

B01-SEQ3.ab1(1>821)

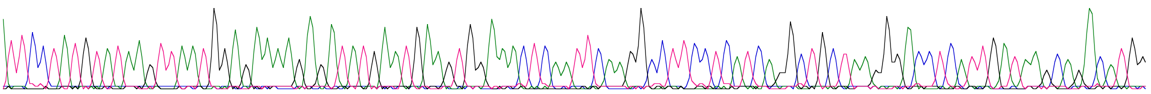

TTCCTATGTATAAGTTAATGGAGAAAAGAGATATGAATGAAGTGGAAACTCAATTCAAGGCCTTCCTCATCAGGCTGCTAAGGGACCTCATTGATAAGCAGACATGG

2,360 2,370 2,380 2,390 2,400 2,410 2,420 2,430 2,440 2,450 2,460

TTCCTATGTATAAGTTAATGGAGAAAAGAGATATGAATGAAGTGGAAACTCAATTCAAGGCCTTCCTCATCAGGCTGCTAAGGGACCTCATTGATAAGCAGACATGG

A07-SEQ4.ab1(1>819)

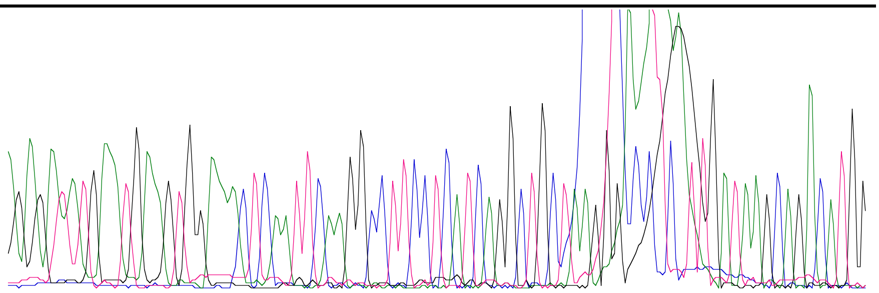

AGAGATATGAATGAAGTGGAAACTCAATTCAAGGCCTTCCTCATCAGGCTGCTAAGGGAcCtCaTTGATAAGCAGACATGG

A10-PFastBac1-R.ab1(1>821)

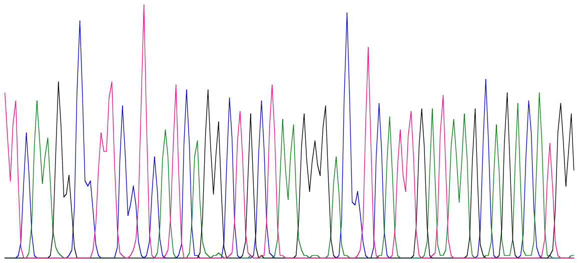

TTCAAGGCCTTCCTCATCAGGCTGCTAAGGGACCTCATTGATAAGCAGACATGG

R725Q Q730E

2,470 2,480 2,490 2,500 2,510 2,520 2,530 2,540 2,550 2,560

ACAGACGAGGGCTCAGTCTCAGAGCAGATGCTGCGGAGTGAGCTACTACTCCTCGCCTGTGTGCACAACTATCAGCCGTGCGTACAGAGGGCAGAAGGCTATTTCAG

U3823DH140-2.seq(1>2878) ACAGACGAGGGCTCAGTCTCAGAGCAGATGCTGCGGAGTGAGCTACTACTCCTCGCCTGTGTGCACAACTATCAGCCGTGCGTACAGAGGGCAGAAGGCTATTTCAG

B07-SEQ4RN.ab1(1>821)

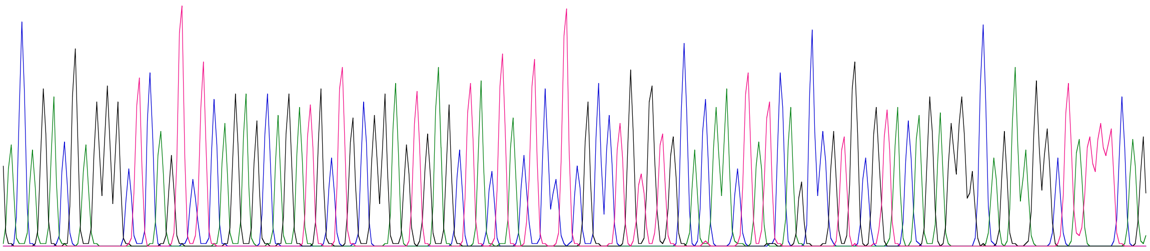

ACAGACGAGGGCTCAGTCTCAGAGCAGATGCTGCGGAGTGAGCTACTACTCCTCGCCTGTGTGCACAACTATCAGCCGTGCGTACAGAGGGCAGAAGGCTATTTCAG

B01-SEQ3.ab1(1>821)

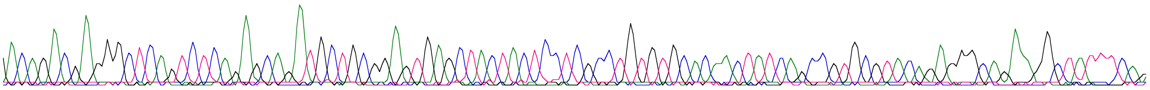

ACAGACGAGGGCTCAGTCTCAGAGCAGATGCTGCGGAGTGAGCTACTACTCCTCGCCTGTGTGCACAACTATCAGCCGTGCGTACAGAGGGCAGAAGGCTATTTCAG

A07-SEQ4.ab1(1>819)

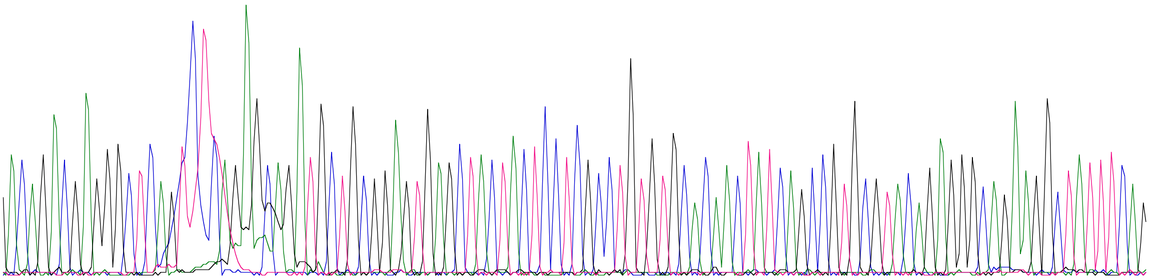

ACAGACGAGGGCTCAGTCTCAgAGCAGATGCTGCGGAGTGAGCTACTACTCCTCGCCTGTGTGCACAACTATCAGCCGTGCGTACAGAGGGCAGAAGGCTATTTCAG

A10-PFastBac1-R.ab1(1>821)

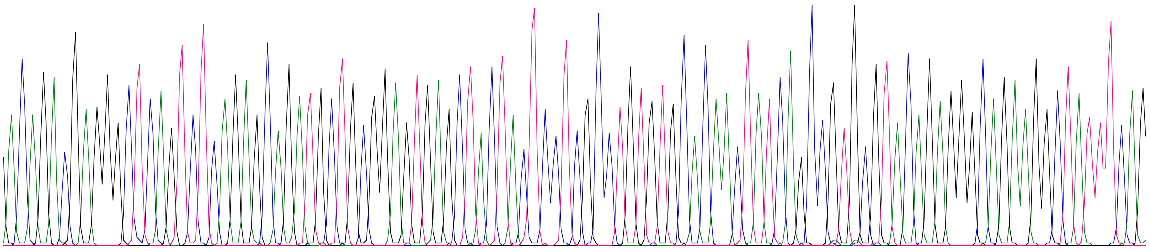

ACAGACGAGGGCTCAGTCTCAGAGCAGATGCTGCGGAGTGAGCTACTACTCCTCGCCTGTGTGCACAACTATCAGCCGTGCGTACAGAGGGCAGAAGGCTATTTCAG

2,570 2,580 2,590 2,600 2,610 2,620 2,630 2,640 2,650 2,660 2,670

AAAGTGGAAGGAATCCAATGGAAACTTGAGCCTGCCTGTCGACGTGACCTTGGCAGTGTTTGCTGTGGGGGCCCAGAGCACAGAAGGCTGGGATTTTCTTTATAGTA

U3823DH140-2.seq(1>2878) → AAAGTGGAAGGAATCCAATGGAAACTTGAGCCTGCCTGTCGACGTGACCTTGGCAGTGTTTGCTGTGGGGGCCCAGAGCACAGAAGGCTGGGATTTTCTTTATAGTA

B07-SEQ4RN.ab1(1>821) ←

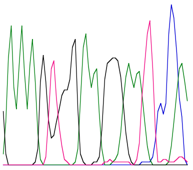

AAAGTGGAAGGAATCCAA

B01-SEQ3.ab1(1>821) →

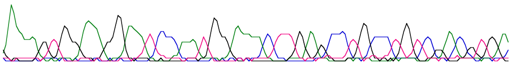

AAAGTGGAAGGAATCCAATGGAAACTTGAGCCTGCCTGTCGACGTGAC

A07-SEQ4.ab1(1>819) →

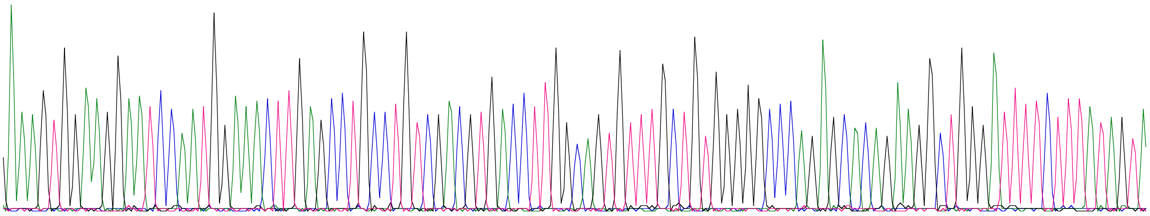

AAAGTGGAAGGAATCCAATGGAAACTTGAGCCTGCCTGTCGACGTGACCTTGGCAGTGTTTGCTGTGGGGGCCCAGAGCACAGAAGGCTGGGATTTTCTTTATAGTA

A10-PFastBac1-R.ab1(1>821) ←

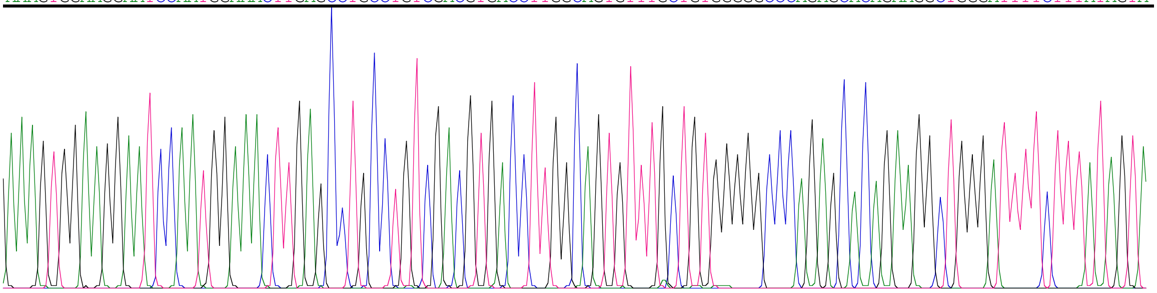

AAAGTGGAAGGAATCCAATGGAAACTTGAGCCTGCCTGTCGACGTGACCTTGGCAGTGTTTGCTGTGGGGGCCCAGAGCACAGAAGGCTGGGATTTTCTTTATAGTA

2,680 2,690 2,700 2,710 2,720 2,730 2,740 2,750 2,760 2,770 2,780

AATATCAGTTTTCTTTGTCCAGTACTGAGAAAAGCCAAATTGAATTTGCCCTCTGCAGAACCCAAAATAAGGAAAAGCTTCAATGGCTACTAGATGAAAGCTTTAAG

U3823DH140-2.seq(1>2878) → AATATCAGTTTTCTTTGTCCAGTACTGAGAAAAGCCAAATTGAATTTGCCCTCTGCAGAACCCAAAATAAGGAAAAGCTTCAATGGCTACTAGATGAAAGCTTTAAG

A07-SEQ4.ab1(1>819) →

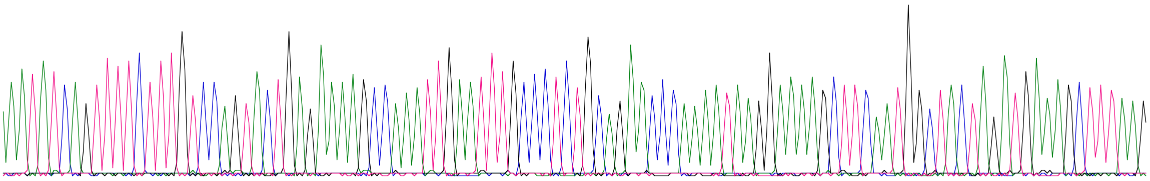

AATATCAGTTTTCTTTGTCCAGTACTGAGAAAAGCCAAATTGAATTTGCCCTCTGCAGAACCCAAAATAAGGAAAAGCTTCAATGGCTACTAGATGAAAGCTTTAAG

A10-PFastBac1-R.ab1(1>821) ←

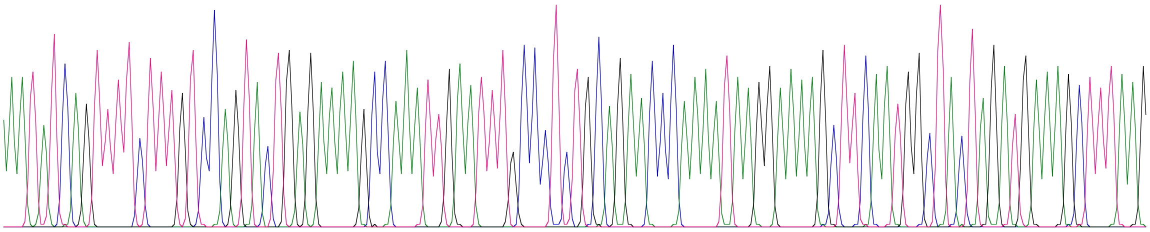

AATATCAGTTTTCTTTGTCCAGTACTGAGAAAAGCCAAATTGAATTTGCCCTCTGCAGAACCCAAAATAAGGAAAAGCTTCAATGGCTACTAGATGAAAGCTTTAAG

2,790 2,800 2,810 2,820 2,830 2,840 2,850 2,860 2,870 2,880

GGAGATAAAATAAAAACTCAGGAGTTTCCACAAATTCTTACACTCATTGGCAGGAACCCAGTAGGATACCCACTGGCCTGGCAATTTCTGAGGAAAAACTGGAACAA

U3823DH140-2.seq(1>2878) GGAGATAAAATAAAAACTCAGGAGTTTCCACAAATTCTTACACTCATTGGCAGGAACCCAGTAGGATACCCACTGGCCTGGCAATTTCTGAGGAAAAACTGGAACAA

A07-SEQ4.ab1(1>819)

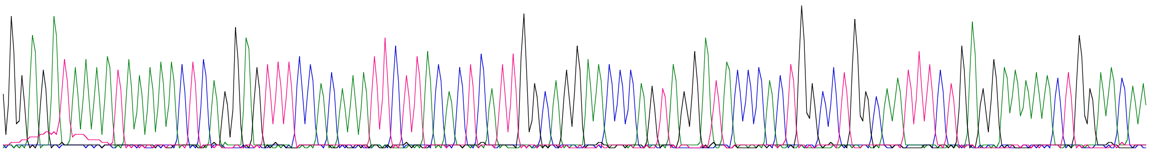

GGAGATAAAATAAAAACTCAGGAGTTTCCACAAATTCTTACACTCATTGGCAGGAACCCAGTAGGATACCCACTGGCCTGGCAATTTCTGAGGAAAAACTGGAACAA

A10-PFastBac1-R.ab1(1>821)

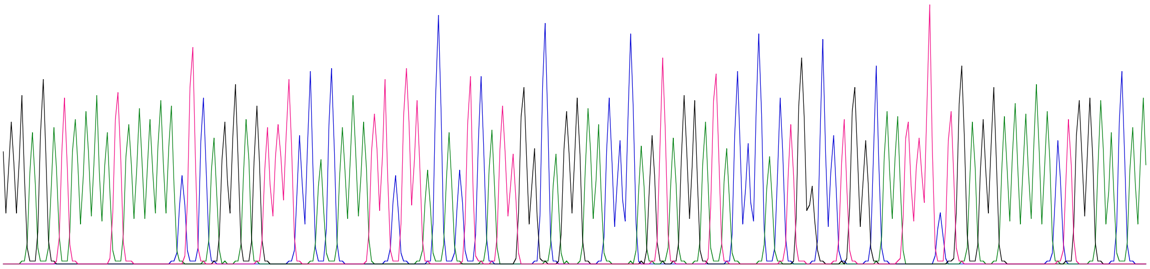

GGAGATAAAATAAAAACTCAGGAGTTTCCACAAATTCTTACACTCATTGGCAGGAACCCAGTAGGATACCCACTGGCCTGGCAATTTCTGAGGAAAAACTGGAACAA

2,890 2,900 2,910 2,920 2,930 2,940 2,950 2,960 2,970 2,980 2,990

ACTTGTACAAAAGTTTGAACTTGGCTCATCTTCCATAGCCCACATGGTAATGGGTACAACAAATCAATTCTCCACAAGAACACGGCTTGAAGAGGTAAAAGGATTCT

U3823DH140-2.seq(1>2878) ACTTGTACAAAAGTTTGAACTTGGCTCATCTTCCATAGCCCACATGGTAATGGGTACAACAAATCAATTCTCCACAAGAACACGGCTTGAAGAGGTAAAAGGATTCT

A07-SEQ4.ab1(1>819)

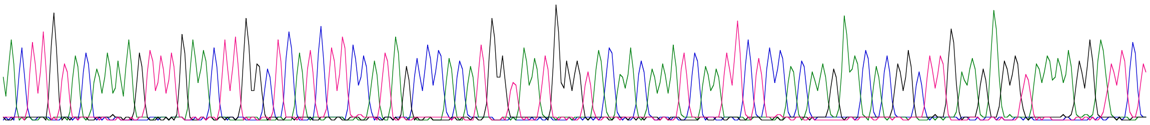

ACTTGTACAAAAGTTTGAACTTGGCTCATCTTCCATAGCCCACATGGTAATGGGTACAACAAATCAATTCTCCACAAGAACACGGCTTGAAGAGGTAAAAGGATTCT

A10-PFastBac1-R.ab1(1>821)

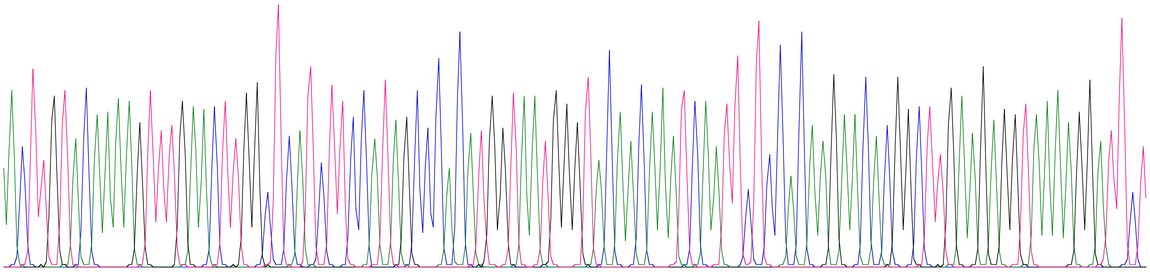

ACTTGTACAAAAGTTTGAACTTGGCTCATCTTCCATAGCCCACATGGTAATGGGTACAACAAATCAATTCTCCACAAGAACACGGCTTGAAGAGGTAAAAGGATTCT

A04-SEQ5.ab1(1>821)

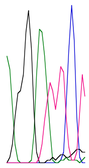

AGGATTCT

3,000 3,010 3,020 3,030 3,040 3,050 3,060 3,070 3,080 3,090 3,100

TCAGCTCTTTGAAAGAAAATGGTTCTCAGCTCCGTTGTGTCCAACAGACAATTGAAACCATTGAAGAAAACATCGGTTGGATGGATAAGAATTTTGATAAAATCAGA

U3823DH140-2.seq(1>2878) → TCAGCTCTTTGAAAGAAAATGGTTCTCAGCTCCGTTGTGTCCAACAGACAATTGAAACCATTGAAGAAAACATCGGTTGGATGGATAAGAATTTTGATAAAATCAGA

A07-SEQ4.ab1(1>819) →

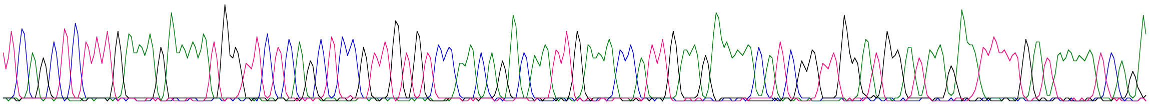

TCAGCTCTTTGAAAGAAAATGGTTCTCAGCTCCGTTGTGTCCAACAGACAATTGAAACCATTGAAGAAAACATCGGTTGGATGGATAAGAATTTTGATAAAATCAGA

A10-PFastBac1-R.ab1(1>821) ←

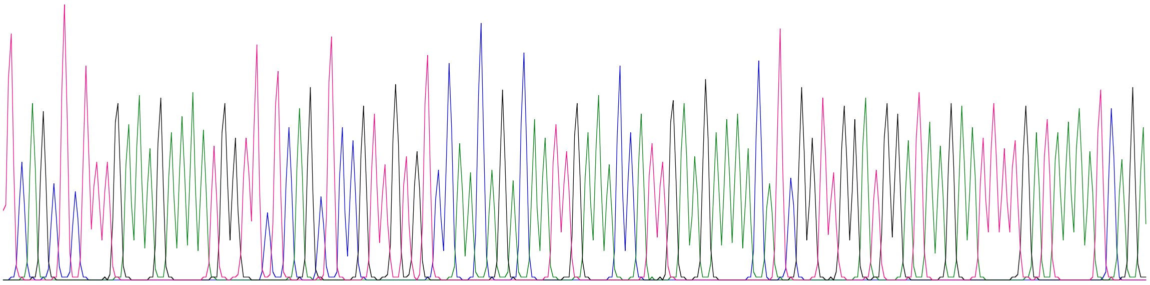

TCAGCTCTTTGAAAGAAAATGGTTCTCAGCTCCGTTGTGTCCAACAGACAATTGAAACCATTGAAGAAAACATCGGTTGGATGGATAAGAATTTTGATAAAATCAGA

A04-SEQ5.ab1(1>821) →

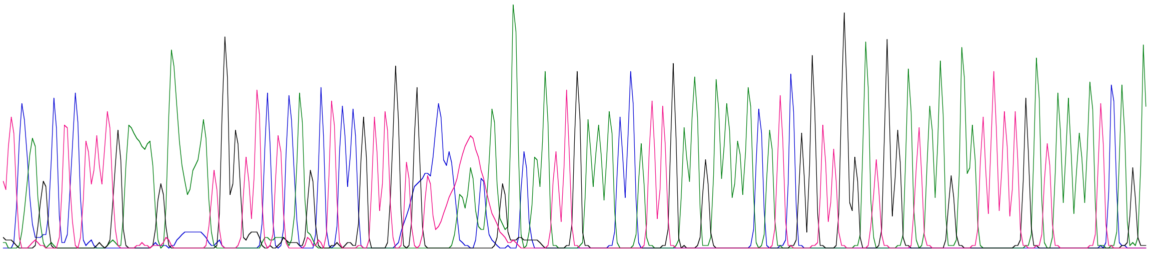

TCAGCTCTTTGAAAGAAAATGGTTCTCAGCTCCGTTGTGTCCAACAGACAATTGAAACCATTGAAGAAAACATCGGTTGGATGGATAAGAATTTTGATAAAATCAGA

3,110 3,120 3,130 3,140 3,150 3,160 3,170 3,180 3,190 3,200 3,210

GTGTGGCTGCAAAGTGAAAAGCTTGAACGCATGCATCATCACCATCACCATTAACTCGAGGCATGCGGTACCAAGCTTGTCGAGAAGTACTAGAGGATCATAATCAG

U3823DH140-2.seq(1>2878) → GTGTGGCTGCAAAGTGAAAAGCTTGAACGCATGCATCATCACCATCACCATTAACTCGAG

A07-SEQ4.ab1(1>819) →

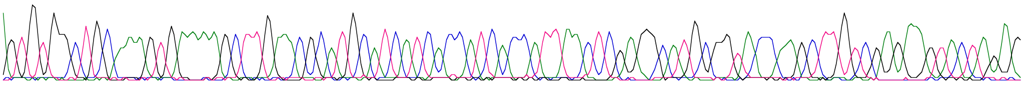

GTGTGGCTGCAAAGTGAAAAGCTTGAACGCATGCATCATCACCATCACCATTAACTCGAGGCATGCGGTACCAAGCTTGTCGAGAAGTACTAGAGG

A10-PFastBac1-R.ab1(1>821) ←

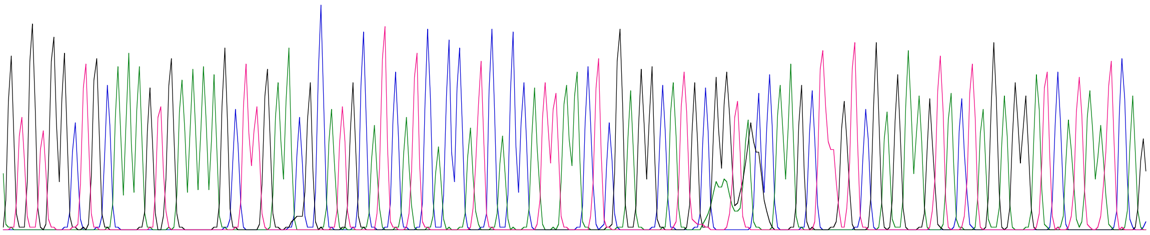

GTGTGGCTGCAAAGTGAAAAGCTTGAACGCATGCATCATCACCATCACCATTAACTCGAGGCATGCGGTACCAAGCTTGTCGAGAAGTACTAGAGGATCATAATCAG

A04-SEQ5.ab1(1>821) →

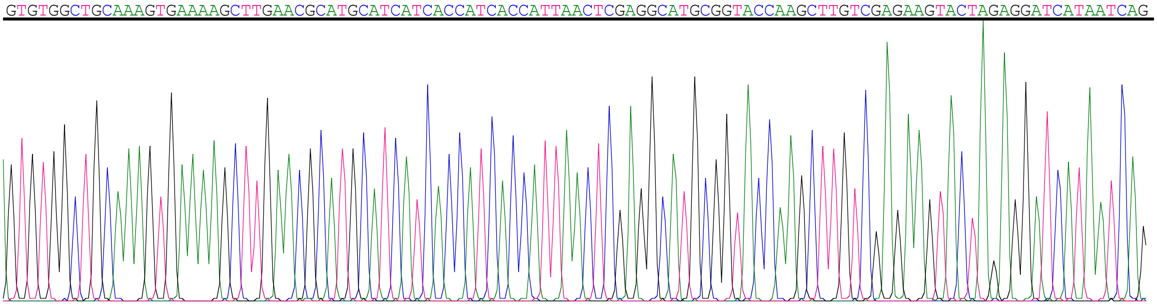

GTGTGGCTGCAAAGTGAAAAGCTTGAACGCATGCATCATCACCATCACCATTAACTCGAGGCATGCGGTACCAAGCTTGTCGAGAAGTACTAGAGGATCATAATCAG

3,220 3,230 3,240 3,250 3,260 3,270 3,280 3,290 3,300 3,310

CCATACCACATTTGTAGAGGTTTTACTTGCTTTAAAAAACCTCCCACACCTCCCCCTGAACCTGAAACATAAAATGAATGCAATTGTTGTTGTTAACTTGTTTATTG

A10-PFastBac1-R.ab1(1>821)

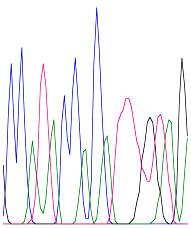

CCATACCACATTTGTAGA

A04-SEQ5.ab1(1>821)

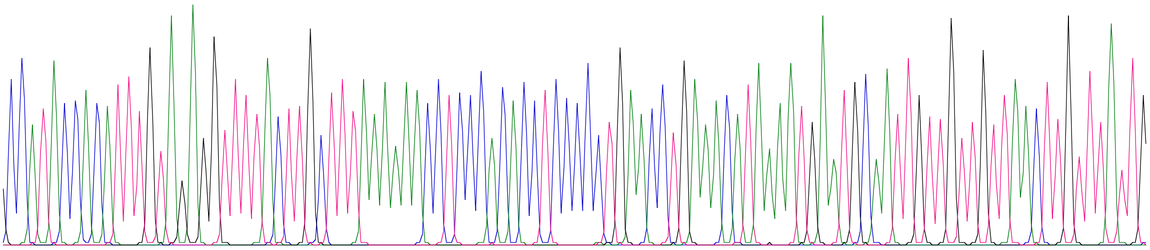

CCATACCACATTTGTAGAGGTTTTACTTGCTTTAAAAAACCTCCCACACCTCCCCCTGAACCTGAAACATAAAATGAATGCAATTGTTGTTGTTAACTTGTTTATTG

3,320 3,330 3,340 3,350 3,360 3,370 3,380 3,390 3,400 3,410 3,420

CAGCTTATAATGGTTACAAATAAAGCAATAGCATCACAAATTTCACAAATAAAGCATTTTTTTCACTGCATTCTAGTTGTGGTTTGTCCAAACTCATCAATGTATCT

A04-SEQ5.ab1(1>821)

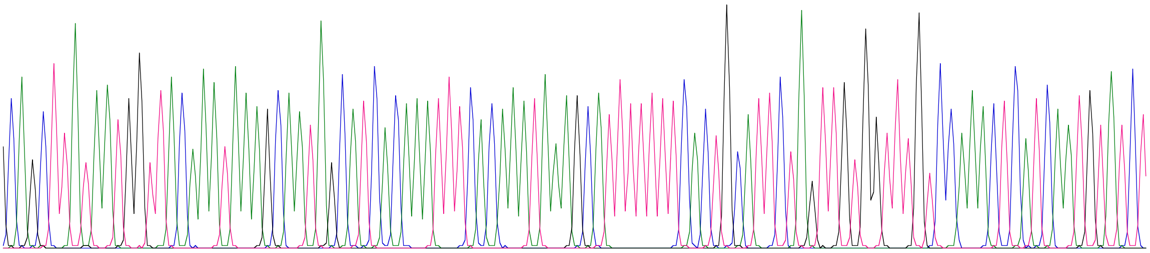

CAGCTTATAATGGTTACAAATAAAGCAATAGCATCACAAATTTCACAAATAAAGCATTTTTTTCACTGCATTCTAGTTGTGGTTTGTCCAAACTCATCAATGTATCT

3,430 3,440 3,450 3,460 3,470 3,480 3,490 3,500 3,510 3,520 3,530

TATCATGTCTGGATCTGATCACTGCTTGAGCCTAGGAGATCCGAACCAGATAAGTGAAATCTAGTTCCAAACTATTTTGTCATTTTTAATTTTCGTATTAGCTTACG

A04-SEQ5.ab1(1>821)

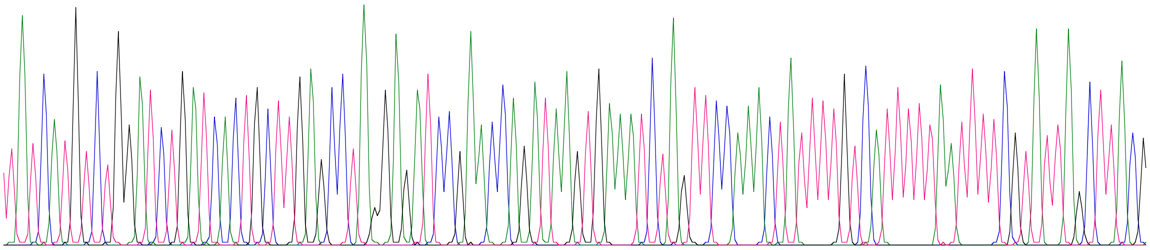

TATCATGTCTGGATCTGATCACTGCTTGAGCCTAGGAGATCCGAACCAGATAAGTGAAATCTAGTTCCAAACTATTTTGTCATTTTTAATTTTCGTATTAGCTTACG

3,540 3,550 3,560 3,570 3,580 3,590 3,600 3,610 3,620 3,630

ACGCTACACCCAGTTCCCATCTATTTTGTCACTCTTCCCTAAATAATCCTTAAAAACTCCATTTCCACCCCTCCCAGTTCCCAACTATTTTGTCCGCCCACAGCGGG

A04-SEQ5.ab1(1>821)

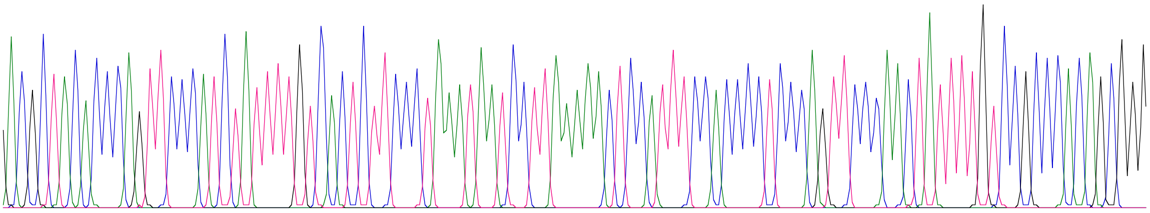

ACGCTACACCCAGTTCCCATCTATTTTGTCACTCTTCCCTAAATAATCCTTAAAAACTCCATTTCCACCCCTCCCAGTTCCCAACTATTTTGTCCGCCCACAGCGGG

3,640 3,650 3,660 3,670 3,680 3,690 3,700 3,710 3,720 3,730 3,740

GCATTTTTCTTCCTGTTATGTTTTTAATCAAACATCCTGCCAACTCCATGTGACAAACCGTCATCTTCGGCTACTTTTTCTCTGTCACAGAATGAAAATTTTTCTGT

A04-SEQ5.ab1(1>821)

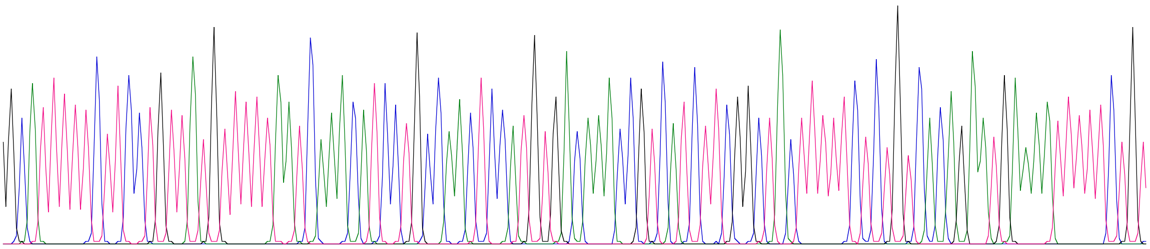

GCATTTTTCTTCCTGTTATGTTTTTAATCAAACATCCTGCCAACTCCATGTGACAAACCGTCATCTTCGGCTACTTTTTCTCTGTCACAGAATGAAAATTTTTCTGT

3,750 3,760 3,770 3,780 3,790 3,800

CATCTCTTCGTTATTAATGTTTGTAATTGACTGAATATCAACGCTTATTTGCAGCCTGAATGGC

A04-SEQ5.ab1(1>821)

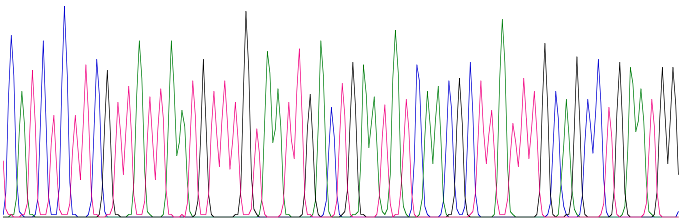

CATCTCTTCGTTATTAATGTTTGTAATTGACTGAATATCAACGCTTATTTGCAGCCTGAATGGC
